# Supplementary figures and images for: Bistability in fatty-acid oxidation resulting from substrate inhibition
Source: PLoS Comput Biol. 2021 Aug 12;17(8):e1009259. doi: 10.1371/journal.pcbi.1009259 (PMC8396765; doi:10.1371/journal.pcbi.1009259)

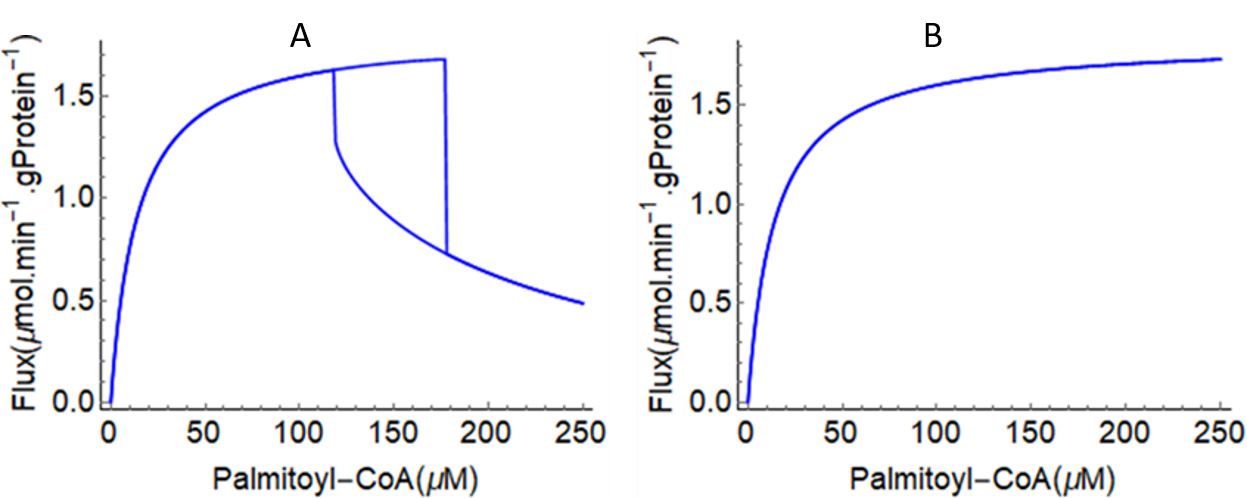

Supplement: S1 Fig — (A) Artificially excluding substrate inhibition of C6-acylCoA from MCKAT resulting a minor shift in the bistability region to higher palmitoyl-CoA. (B) Artificially excluding substrate inhibition of C4-acylCoA from MCKAT resulting the model to exhibit a saturation kinetics and the bistability disappeared, i.e., the low-flux state disappeared at least up to 250 μM of palmitoyl-CoA. (TIF) [file pcbi.1009259.s001.tif]

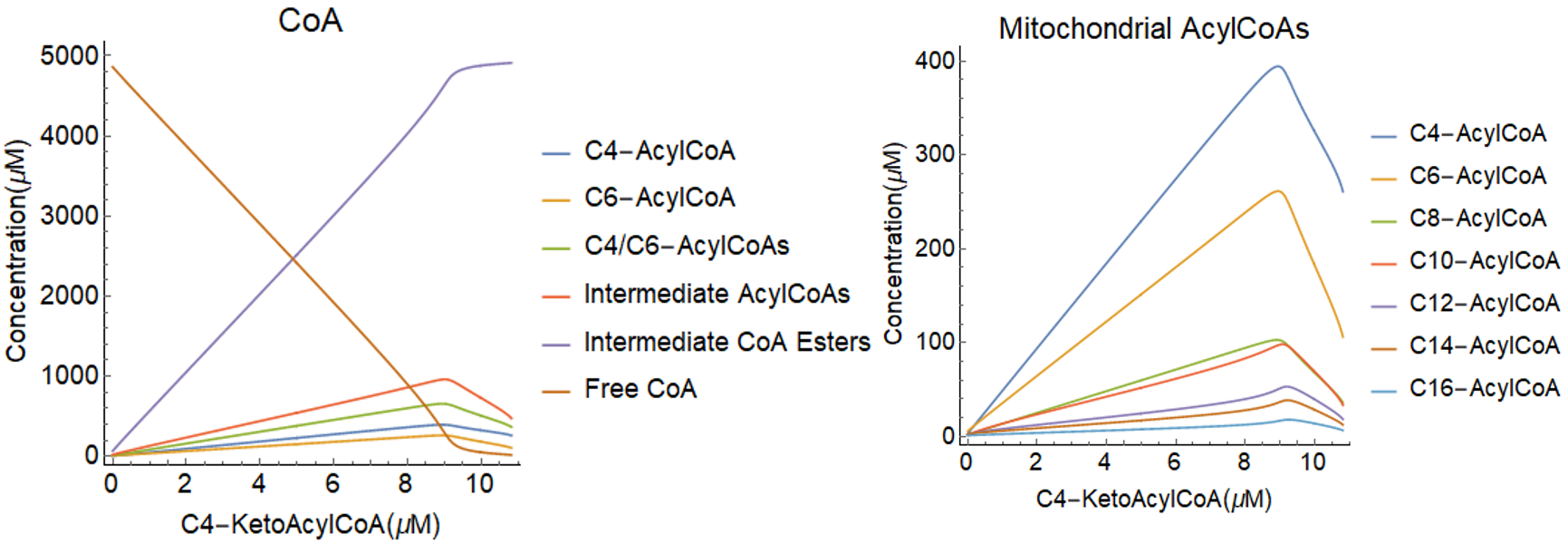

Supplement: S2 Fig — The plots show the depletion of free CoA and accumulation of C4 –C16 intermediate CoA esters with increasing C4-KetoAcylCoA. (TIF) [file pcbi.1009259.s002.tif]

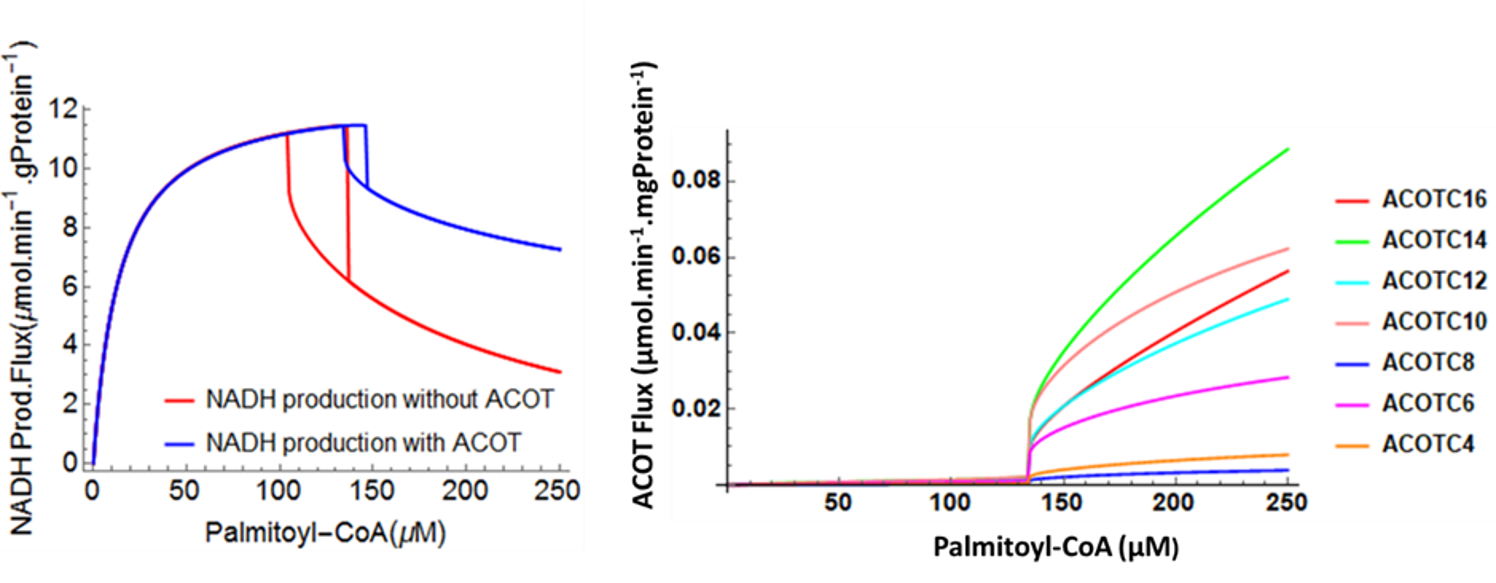

Supplement: S3 Fig — The left panel shows the NADH production with and without including ACOT in the model. The right panel shows carbon-chain specific activities of ACOT. (TIF) [file pcbi.1009259.s003.tif]

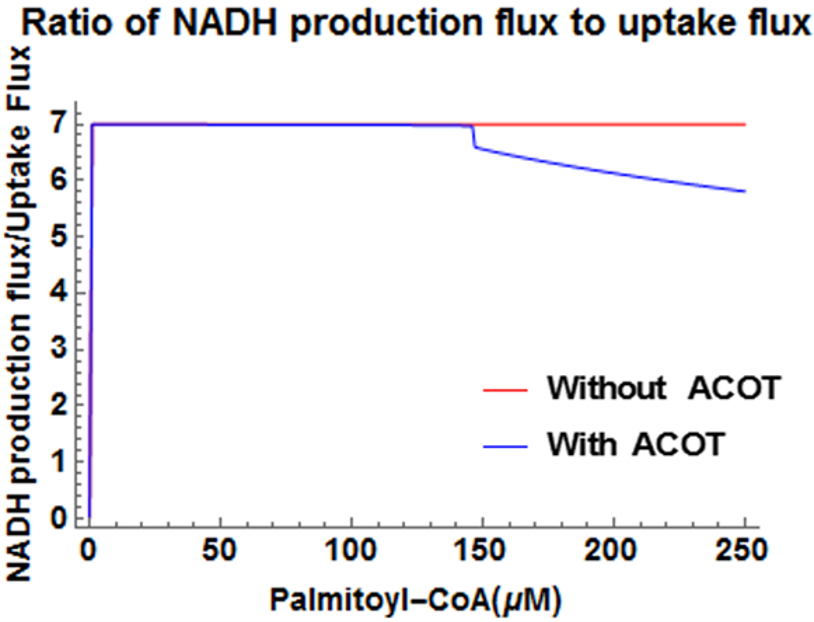

Supplement: S4 Fig — (TIF) [file pcbi.1009259.s004.tif]

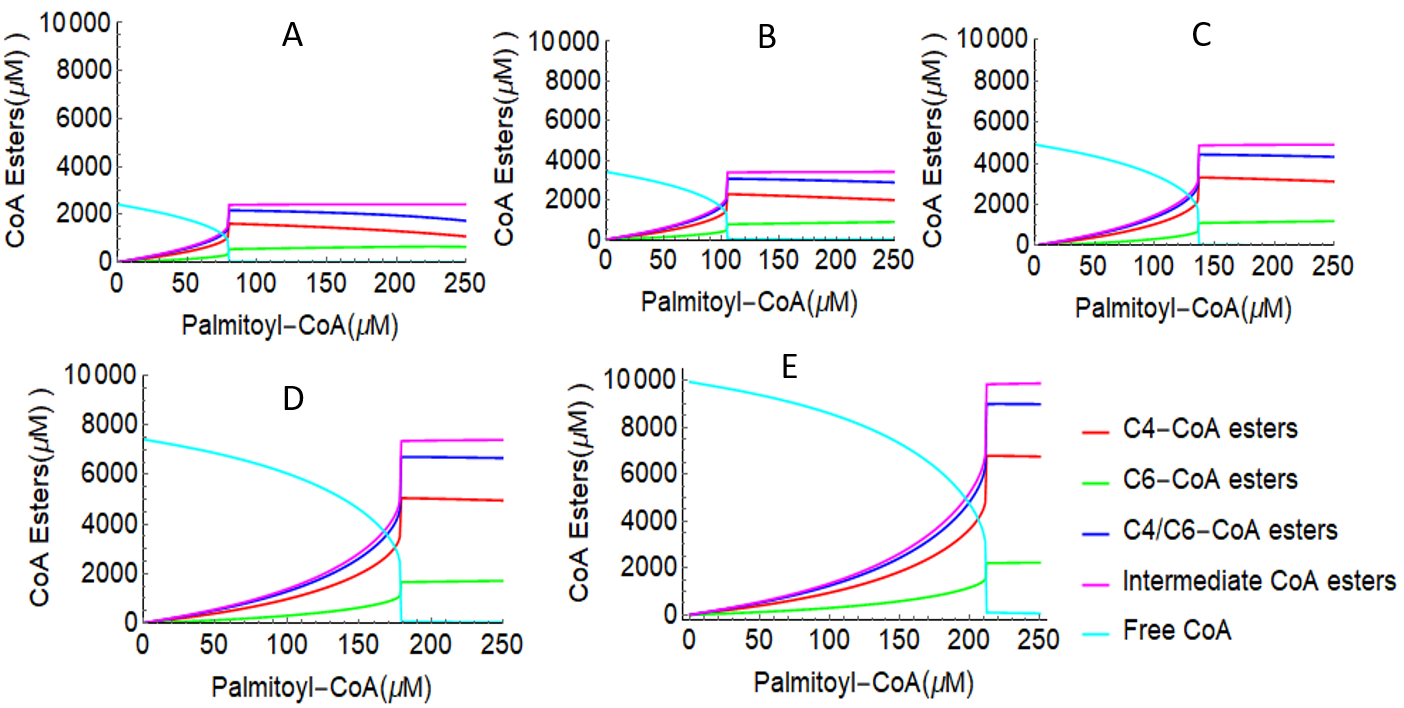

Supplement: S5 Fig — Total CoA pool (A) 2500 μM, (B) 3500 μM, (C) 5000 μM, (D) 7500 μM, (E) 10000 μM. Each plot shows as the concentration of palmitoyl-CoA increases, the depletion of free CoA and accumulation of intermediate CoA esters. The sharp decline in free CoA is shifted to higher palmitoyl-CoA concentration with increasing total CoA pool from 2500 to 10000 μM. (TIF) [file pcbi.1009259.s005.tif]

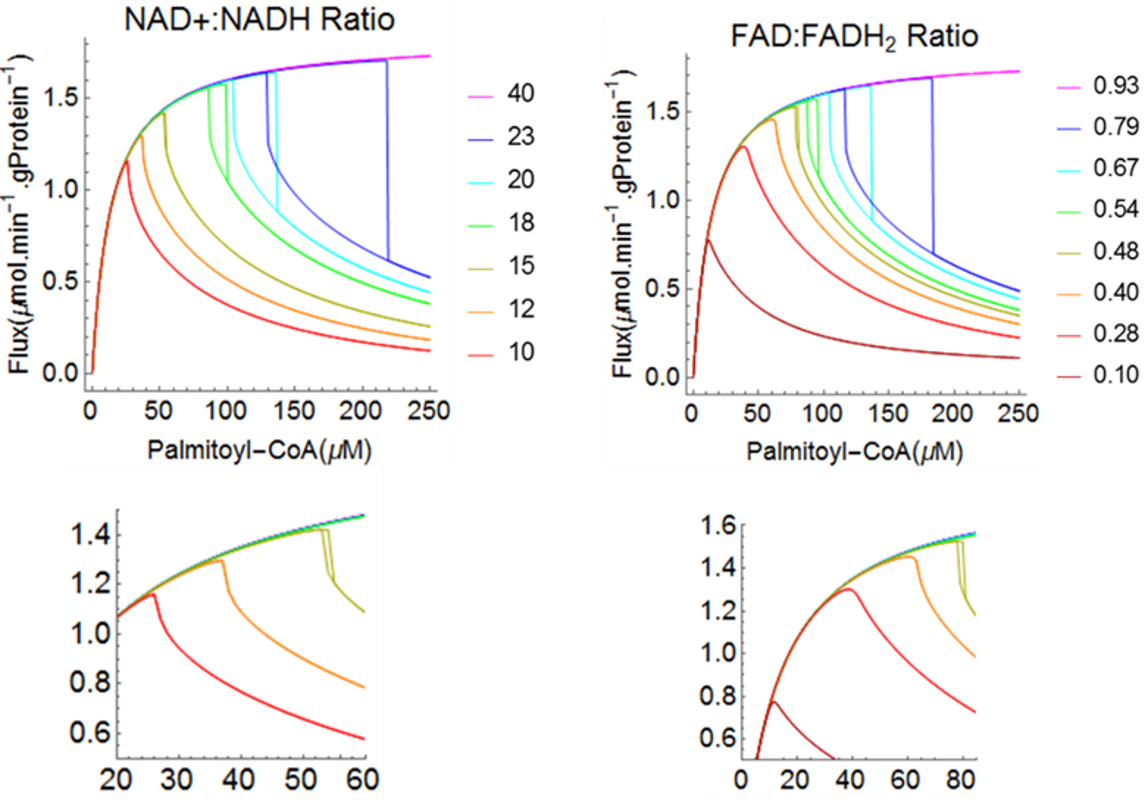

Supplement: S6 Fig — (TIF) [file pcbi.1009259.s006.tif]

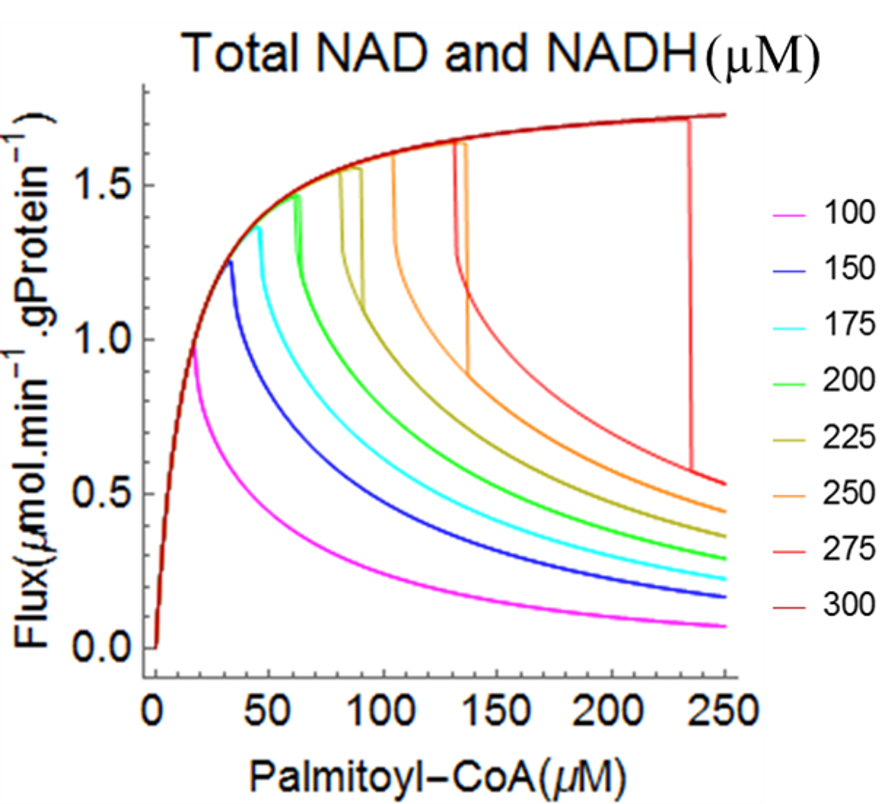

Supplement: S7 Fig — (TIF) [file pcbi.1009259.s007.tif]

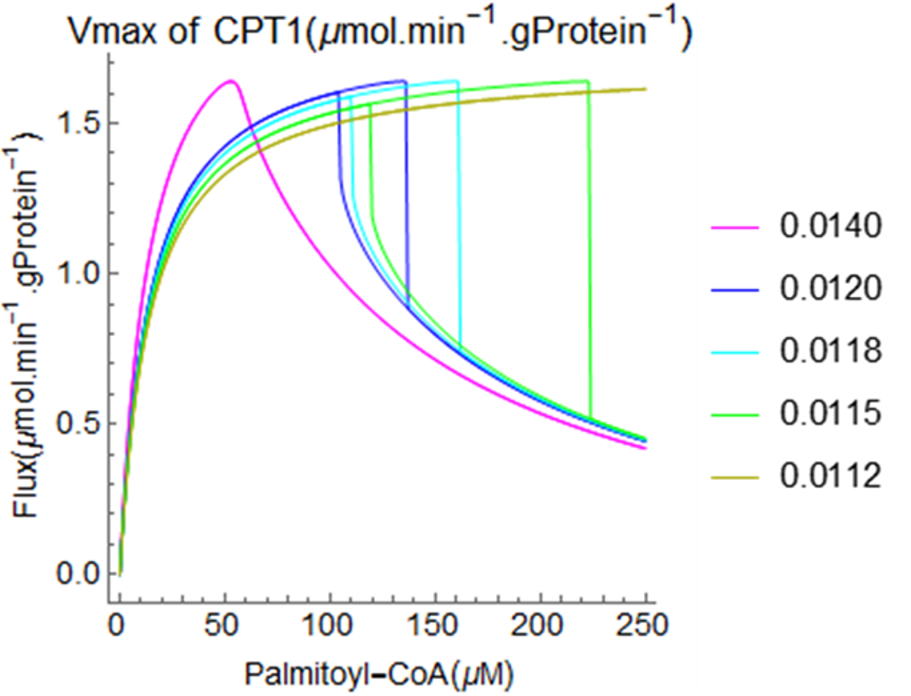

Supplement: S8 Fig — With decreasing Vmax of CPT1, bistability set in at a higher palmitoyl-CoA concentration. (TIF) [file pcbi.1009259.s008.tif]

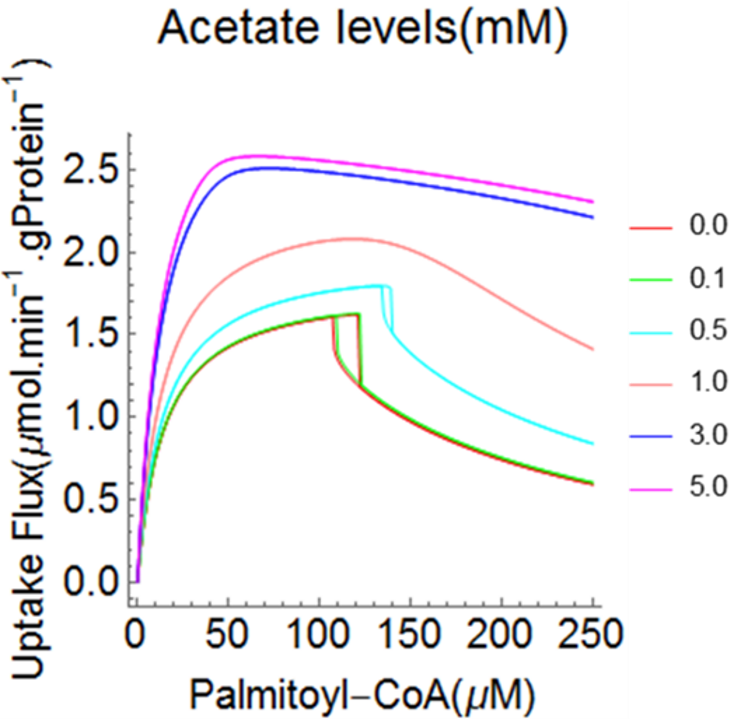

Supplement: S9 Fig — With increasing levels of acetate from 0 to 5 mM, the bistability behavior and flux decline eventually vanished. (TIF) [file pcbi.1009259.s009.tif]
