# Supplementary material for: Bistability in fatty-acid oxidation resulting from substrate inhibition
Source: PLoS Comput Biol. 2021 Aug 12;17(8):e1009259. doi: 10.1371/journal.pcbi.1009259 (PMC8396765; doi:10.1371/journal.pcbi.1009259)
Supplement: S1 Text — (PDF) [file pcbi.1009259.s010.pdf]

S1 Text: Ordinary differential equations and rate equations in the extended mFAO models

1. Ordinary differential equation and rate equations for NADH concentration in the mitochondria

$$\frac{dNADH}{dt} = \frac{1}{VMAT}(vmtpC16 + vmtpC14 + vmtpC12 + vmtpC10 + vmtpC8 + vmschadC16 + vmschadC14 + vmschadC12 + vmschadC10 + vmschadC8 + vmschadC6 + vmschadC4 + vpdhnad - voxphnadh)$$

where the rate equations $vpdhnad$ and $voxphnadh$ are given by

$$vpoxphnadh = \frac{\boldsymbol{VmaxOxPh} \cdot (\boldsymbol{1} + \boldsymbol{\alpha}) \cdot \left(\frac{[\boldsymbol{NADH}]}{\boldsymbol{Km}_{\text{nadhOxPh}}}\right)}{\left(\boldsymbol{1} + \frac{[\boldsymbol{NADH}]}{\boldsymbol{Km}_{\text{nadhOxPh}}} + \frac{[\boldsymbol{Nt} - \boldsymbol{NADH}]}{\boldsymbol{Km}_{\text{nadOxPh}}}\right)}$$

$$vpdhnad = \frac{\boldsymbol{VmaxPDH} \cdot \left(\frac{[\boldsymbol{Nt} - \boldsymbol{NADH}]}{\boldsymbol{Km}_{\text{nadPDH}}}\right)}{\left(\boldsymbol{1} + \frac{[\boldsymbol{Nt} - \boldsymbol{NADH}]}{\boldsymbol{Km}_{\text{nadPDH}}} + \frac{[\boldsymbol{NADH}]}{\boldsymbol{Km}_{\text{nadhPDH}}}\right)}$$

The remaining rate equations ($vmtpC16, vmtpC14, vmtpC12, vmtpC10, vmtpC8, vmschadC16, vmschadC14, vmschadC12, vmschadC10, vmschadC8, vmschadC6, vmschadC4$) are as given in [1]. The value of **α** is given below and Nt is the total pool of NAD and NADH.

2. Parameter values for enzyme reactions (ACOT, OxPh and PDH) included in the Extended mFAO model.

ACOT (Acyl-CoA thioesterases):

Parameter	Values	Unit	Reference
sfacotC16	0.70		[2]
sfacotC14	0.86		[2]
sfacotC12	0.68		[2]
sfacotC10	1.00		[2]
sfacotC8	0.21		Estimated
sfacotC6	0.55		[2]
sfacotC4	0.70		Estimated
Vmax_acot	0.00371	μmol.min^{-1}.mgProtein^{-1}	[3], Adapted
KmacotC16CoA	10	μM	[2]
KmacotC14CoA	15.5	μM	[2]
KmacotC12CoA	27.5	μM	[2]
KmacotC10CoA	47.2	μM	[2]
KmacotC8CoA	150.89	μM	Estimated
KmacotC6CoA	138.2	μM	[2]
KmacotC4CoA	1036.29	μM	Estimated

KmacotC16FFA	2.7E4	μM	Estimated
KmacotC14FFA	1.3E5	μM	Estimated
KmacotC12FFA	2.3E5	μM	Estimated
KmacotC10FFA	4E5	μM	Estimated
KmacotC8FFA	1.3E6	μM	Estimated
KmacotC6FFA	1.2E6	μM	Estimated
KmacotC4FFA	3.5E8	μM	Estimated
Km_CoA	9	μM	[4]
KeqacotC16	2.4E4		Estimated
KeqacotC14	7.6E4		Estimated
KeqacotC12	7.6E4		Estimated
KeqacotC10	7.6E4		Estimated
KeqacotC8	7.6E4		Estimated
KeqacotC6	7.6E4		Estimated
KeqacotC4	3.0E6		Estimated
**Oxidative Phosphorylation (OxPh)**			
KmnadhOxPh	4.3	μM	[5,6]
KmnadOxPh	780	μM	[5,6]
VmaxOxPh	0.155	$\mu mol.min^{-1}.mgProtein^{-1}$	Adapted
**PDH (Pruyvate Dehydrogenase)**			
KmnadhPDH	40	μM	[7]
KmnadPDH	60.7	μM	[7]
VmaxPDH	0.127	$\mu mol.min^{-1}.mgProtein^{-1}$	[7]

3. Modified parameters

a. MCKAT inhibition by p46Shc protein

The inhibitory effect of p46Shc protein on MCKAT enzyme is expressed by its modified Vmax value given by

$$Vmax\_mckat_{modified} = Vmax\_mckat * (1 + \beta) \qquad \text{(E1)}$$

where $Vmax\_mckat$ = 0.377 is the Vmax of MCKAT in the model we considered [1], and β= 0.30, which was a value used to mimic 30% increase in palmitate oxidation capacity for purified mitochondria from ShcKO (20% residual activity of p46Shc protein) mice compared to controls (WT levels of p46Shc) [8].

b. Short chain fatty acids contribution to CPT1 and Oxidative Phosphorylation activities

The Vmax of CPT1 and oxidative phosphorylation (OxPh) were expressed as a function of acetate. For j= CPT1 or OxPh, their modified Vmax is given by.

$$Vmax, j_{modified} = Vmax, j * (1 + \alpha) \quad \text{(E2)}$$

where $\alpha = 0.9$ if acetate concentration is 3 mM which was fixed to mimic about a two-fold increase in CPT1 activity and UPC2 expression after long term acetate treatment [9]. Their study demonstrated that i) short chain fatty acid (3 mM acetate) feeding led to a two-fold increase in the enzyme activity of CPT1 in liver (i.e., the Vmax of CPT1 increased from 0.012 $\mu mol \cdot min^{-1} \cdot mgProtien^{-1}$ for controls which is a value in the model we considered to about 0.023 $\mu mol \cdot min^{-1} \cdot mgProtien^{-1}$) and ii) short chain fatty acid (3 mM acetate) feeding led to a two-fold increase in the expression of UCP2 over controls suggesting proton leak via UCP2 that could be responsible for the uncoupling of mitochondrial oxidative phosphorylation.

4. Computational tools for model simulations

A. Mathematica simulation codes for steady state analysis (S1-S6 Appendix)

B. Matlab for the bistability analysis (Figure 2 and Figure 4 in the main text): we used *matcont GUI version* in matlab on the mitochondrial fatty-acid oxidation model [1] code available through the JWS online database (http://jjj.biochem.sun.ac.za/models/vaneunen6). All the model parameters are unchanged except the NADH value changed from 16 µM to 12µM that was due to a change in the [NAD+]:[NADH] ratio from 15 to ~20 [10].

References

1. van Eunen K, Simons SMJ, Gerding A, Bleeker A, den Besten G, Touw CML, et al. Biochemical competition makes fatty-acid β-oxidation vulnerable to substrate overload. PLoS Comput Biol. 2013;9: e1003186. doi:10.1371/journal.pcbi.1003186

2. Wei J, Kang HW, Cohen DE. Thioesterase superfamily member 2 (Them2)/acyl-CoA thioesterase 13 (Acot13): a homotetrameric hotdog fold thioesterase with selectivity for long-chain fatty acyl-CoAs. Biochem J. 2009;421: 311–322. doi:10.1042/BJ20090039

3. Kang HW, Niepel MW, Han S, Kawano Y, Cohen DE. Thioesterase superfamily member 2/acyl-CoA thioesterase 13 (Them2/Acot13) regulates hepatic lipid and glucose metabolism. FASEB J. 2012;26: 2209–2221. doi:10.1096/fj.11-202853

4. Cao J, Xu H, Zhao H, Gong W, Dunaway-Mariano D. The Mechanisms of Human Hotdog-fold Thioesterase 2 (hTHEM2) Substrate Recognition and Catalysis Illuminated by a Structure and Function Based Analysis,. Biochemistry. 2009;48: 1293–1304. doi:10.1021/bi801879z

5. Heiske M, Nazaret C, Mazat J-P. Modeling the respiratory chain complexes with biothermokinetic equations - the case of complex I. Biochim Biophys Acta. 2014;1837: 1707–1716. doi:10.1016/j.bbabio.2014.07.013

6. Heiske M, Letellier T, Klipp E. Comprehensive mathematical model of oxidative phosphorylation valid for physiological and pathological conditions. FEBS J. 2017;284: 2802–2828. doi:https://doi.org/10.1111/febs.14151

7. Wu F, Yang F, Vinnakota KC, Beard DA. Computer modeling of mitochondrial tricarboxylic acid cycle, oxidative phosphorylation, metabolite transport, and electrophysiology. J Biol Chem. 2007;282: 24525–24537. doi:10.1074/jbc.M701024200

8. Tomilov A, Tomilova N, Shan Y, Hagopian K, Bettaieb A, Kim K, et al. p46Shc Inhibits Thiolase and Lipid Oxidation in Mitochondria. J Biol Chem. 2016;291: 12575–12585. doi:10.1074/jbc.M115.695577

9. den Besten G, Bleeker A, Gerding A, van Eunen K, Havinga R, van Dijk TH, et al. Short-Chain Fatty Acids Protect Against High-Fat Diet-Induced Obesity via a PPARγ-Dependent Switch From Lipogenesis to Fat Oxidation. Diabetes. 2015;64: 2398–2408. doi:10.2337/db14-1213

10. Siess EA, Brocks DG, Wieland OH. Subcellular distribution of key metabolites in isolated liver cells from fasted rats. FEBS Lett. 1976;69: 265–271. doi:10.1016/0014-5793(76)80701-6
