# Supplementary material for: Bistability in fatty-acid oxidation resulting from substrate inhibition
Source: PLoS Comput Biol. 2021 Aug 12;17(8):e1009259. doi: 10.1371/journal.pcbi.1009259 (PMC8396765; doi:10.1371/journal.pcbi.1009259)
Supplement: S2 Text — (PDF) [file pcbi.1009259.s011.pdf]

S2 Text: Analytical derivation of steady states in one dimensional ODE for a reduced mFAO model

In order to analytically investigate bistability, we considered a reduced mFAO pathway around C4-KetoAcylCoA. The ODE related to the rate of change in the concentration C4-Ketoacyl-CoA over time is given by

$$VMAT\frac{dC4KetoacylCoAMAT}{dt} = vMSCHADC4 - vMCKATC4$$

where the rate equations are given by

$$vMSCHADC4 = \frac{sf_{vmschad}V_{max,mschad}\left(\frac{[C4HydroxyacylCoA][Nt-NADH]}{Km_{MSCHAD,C4hydroxylCoA}Km_N} - \frac{[C4KetoacylCoA][NADH]}{Km_{MSCHAD,C4hydroxylCoA}Km_N K_{eq,MSCHAD}}\right)}{\left(1+\frac{[C4HydroxyacylCoA]}{Km_{MSCHAD,C4hydroxylCoA}} + \underbrace{\frac{[C4KetoacylCoA]}{Km_{MSCHAD,C4hydroxylCoA}}}_{inhibition\ by\ reaction\ product} + \underbrace{\sum_{n=6}^{16}\left\{\frac{[CnHydroxyacylCoA]}{Km_{MSCHAD,CnhydroxylCoA}} + \frac{[CnKetoacylCoA]}{Km_{MSCHAD,CnketoacylCoA}}\right\}}_{inhibition\ by\ substrates\ and\ products\ of\ other\ chain\ length}\right)\left(1+\frac{[Nt-NADH]}{Km_N}+\frac{[NADH]}{Km_{NADH}}\right)}$$

(A1)

$$vMCKATC4 = \frac{sf_{vmckat}V_{max,mckat}\left(\frac{[C4KetoacylCoA][FreeCoA]}{Km_{MCKAT,C4ketoacyCoA}Km_{CoA}} - \frac{[AcetylCoA][AcetylCoA]}{Km_{MCKAT,C4ketoacyCoA}Km_{CoA}K_{eq,MCKAT}}\right)}{\left(1+\frac{[C4acylCoA]}{Km_{MCKAT,C4acyCoA}} + \underbrace{\frac{[AcetylCoA]}{Km_{AcetylCoA}}}_{inhibition\ by\ reaction\ product} + \underbrace{\sum_{n=6}^{16}\left\{\frac{[CnKetoacylCoA]}{Km_{MCKAT,CnketoacyCoA}} + \frac{[CnAcylCoA]}{Km_{MCKAT,CnacyCoA}}\right\}}_{inhibition\ by\ substrates\ and\ products\ of\ other\ chain\ length}\right)\left(1+\frac{[FreeCoA]}{Km_{CoA}}+\frac{[AcetylCoA]}{Km_{AcetylCoA}}\right)}$$

(A2)

The following notations are used:

M=C4-Ketoacyl-CoAMAT

Mn=Cn-Ketoacyl-CoAMAT, n=6, 8, 10, 12, 14, 16

Hn= Cn-Hydroxyacyl-CoAMAT, n=4, 6, 8, 10, 12, 14, 16

T= total CoA pool

S = the sum of all mitochondrial CoA esters except C4-Ketoacyl-CoAMAT (M)

Nt= total of NAD+ and NADH (ND)

Acetyl =Acetyl-CoA

$vmschad = sf_{vmschad}V_{max,mschad}$

$vmckat = sf_{vmckat}V_{max,mckat}$

Then the rate equations can be rewritten as

$$vMSCHADC4 = \frac{vmschad\left(\frac{H4(Nt-N_D)}{K_{H4}K_N} - \frac{M\,N_D}{K_{H4}K_N K_{eqMSCHAD}}\right)}{\left(1+\frac{H4}{K_{H4}}+\frac{M}{K_{Msc4}}+\frac{Q_1}{K_{Q1}}\right)\left(1+\frac{Nt-N_D}{K_N}+\frac{N_D}{K_{N_D}}\right)} \qquad (A3)$$

$$vMCKATC4 = \frac{vmckat\left(\frac{M(T-S-M)}{K_{Mc4}K_{CoA}} - \frac{Acetyl \times Acetyl}{K_{Mc4}K_{CoA}K_{eqMCKAT}}\right)}{\left(1+\frac{M}{K_{Mc4}}+\frac{Acetyl}{K_{ACoA}}+\frac{Q_2}{K_{Q2}}\right)\left(1+\frac{T-S-M}{K_{CoA}}+\frac{Acetyl}{K_{ACoA}}\right)} \qquad (A4)$$

Where the competition part in (A3) can be expressed as

$$\frac{Q_1}{K_{Q1}} = \frac{H6}{K_{H6}} + \frac{H8}{K_{H8}} + \frac{H10}{K_{H10}} + \frac{H12}{K_{H12}} + \frac{H14}{K_{H14}} + \frac{H16}{K_{H16}} + \frac{M6}{K_{M6}} + \frac{M8}{K_{M8}} + \frac{M10}{K_{M10}} + \frac{M12}{K_{M12}} + \frac{M14}{K_{M14}} + \frac{M16}{K_{M16}}$$

Such that

$$\frac{Q_1}{K_{Q1}} = \frac{\sum_j H_j \prod_{i\neq j} K_{Hi} \prod_l K_{Ml} + \sum_l M_l \prod_j K_{Hj} \prod_{n\neq l} K_{Mn}}{\prod_j K_{Hj} \prod_l K_{Ml}} \quad , j, i, l, n \in \{6, 8, 10, 12, 14, 16\};$$

Similarly, the competition part in (A4) can be expressed as

$$\frac{Q_2}{K_{Q2}} = \frac{M6}{K_{M4}} + \frac{M8}{K_{M8}} + \frac{M10}{K_{M10}} + \frac{M12}{K_{M12}} + \frac{M14}{K_{M14}} + \frac{M16}{K_{M16}} + \frac{A4}{K_{A4}} + \frac{A6}{K_{A6}} + \frac{A8}{K_{A8}} + \frac{A10}{K_{A10}} + \frac{A12}{K_{A12}} + \frac{A14}{K_{A14}} + \frac{A16}{K_{A16}}$$

Such that

$$\frac{Q_2}{K_{Q2}} = \frac{\sum_j M_j \prod_{i\neq j} K_{Mi} \prod_l K_{Al} + \sum_l A_l \prod_j K_{Mj} \prod_{n\neq l} K_{An}}{\prod_j K_{Mj} \prod_l K_{Al}}, j, i \in \{6, 8, 10, 12, 14, 16\}; \quad l, n \in \{4, 6, 8, 10, 12, 14, 16\}$$

At steady state

$$VMAT\frac{dM}{dt} = 0$$

implying

$$vMSCHADC4 - vMCKATC4 = 0 \qquad (A5)$$

Substituting (A3) and (A4) into (A5) we have

$$\frac{\text{Vmschad}\left(\frac{\text{H4}(\text{Nt}-\text{N}_\text{D})}{\text{K}_{\text{H4}}\text{K}_\text{N}}-\frac{\text{M N}_\text{D}}{\text{K}_{\text{H4}}\text{K}_\text{N}\text{K}_{\text{eqMSCHAD}}}\right)}{\left(1+\frac{\text{H4}}{\text{K}_{\text{H4}}}+\frac{\text{M}}{\text{K}_{\text{Msc4}}}+\frac{\text{Q}_1}{\text{K}_{\text{Q1}}}\right)\left(1+\frac{\text{Nt}-\text{N}_\text{D}}{\text{K}_\text{N}}+\frac{\text{N}_\text{D}}{\text{K}_{\text{N}_\text{D}}}\right)}-\frac{\text{Vmckat}\left(\frac{\text{M}(\text{T}-\text{S}-\text{M})}{\text{K}_{\text{Mc4}}\text{K}_{\text{CoA}}}-\frac{\text{Acetyl}\ \times\text{Acetyl}}{\text{K}_{\text{Mc4}}\text{K}_{\text{CoA}}\text{K}_{\text{eqMCKAT}}}\right)}{\left(1+\frac{\text{M}}{\text{K}_{\text{Mc4}}}+\frac{\text{Acetyl}}{\text{K}_{\text{ACoA}}}+\frac{\text{Q}_2}{\text{K}_{\text{Q2}}}\right)\left(1+\frac{\text{T}-\text{S}-\text{M}}{\text{K}_{\text{CoA}}}+\frac{\text{Acetyl}}{\text{K}_{\text{ACoA}}}\right)} = 0$$

This can be simplified to a cubic polynomial of the form

$$\text{a M}^3+\text{b M}^2+\text{c M}+\text{d}=0 \tag{A6}$$

where

$a = a_1 + a_2$

$b = b_1 - b_2 - b_3 + b_4$

$c = c_1 - c_2 + c_3 - c_4$

$d = d_1 + d_2$

$$a_1 = \frac{\text{Vmschad}\ \ \text{N}_\text{D}}{\text{K}_{\text{H4}}\text{K}_\text{N}\text{K}_{\text{Mc4}}\text{K}_{\text{CoA}}\text{K}_{\text{eqMSCHAD}}}$$

$$b_1 = \frac{\text{Vmschad}\ \ \text{H4}\ (\text{Nt}-\text{N}_\text{D})}{\text{K}_{\text{H4}}\text{K}_\text{N}\text{K}_{\text{Mc4}}\text{K}_{\text{CoA}}}$$

$$b_2 = \frac{\text{Vmschad}\ \ \text{N}_\text{D}}{\text{K}_{\text{H4}}\text{K}_\text{N}\text{K}_{\text{eqMSCHAD}}}\times w_2$$

$$c_1 = \frac{\text{Vmschad}\ \ \text{H4}\ (\text{Nt}-\text{N}_\text{D})}{\text{K}_{\text{H4}}\text{K}_\text{N}}\times w_2$$

$$c_2 = \frac{\text{Vmschad}\ \ \text{N}_\text{D}}{\text{K}_{\text{H4}}\text{K}_\text{N}\text{K}_{\text{eqMSCHAD}}}\times w_1$$

$$d_1 = \frac{\text{Vmschad}\ \ \text{H4}\ (\text{Nt}-\text{N}_\text{D})}{\text{K}_{\text{H4}}\text{K}_\text{N}}\times w_1$$

$$a_2 = \frac{\text{Vmckat}}{\text{K}_{\text{Mc4}}\text{K}_{\text{CoA}}\text{K}_{\text{Msc4}}}\times y_2,$$

$$b_3 = \frac{\text{Vmckat}\ (\text{T}-\text{S})}{\text{K}_{\text{Mc4}}\text{K}_{\text{CoA}}\text{K}_{\text{eqMCKAT}}\text{K}_{\text{Msc4}}}\times y_2,$$

$$b_4 = \frac{\text{Vmckat}}{\text{K}_{\text{Mc4}}\text{K}_{\text{CoA}}}\times y_1,$$

$$c_3 = \frac{\text{Vmckat AcetylCoA}\times\ \text{Acetyl}}{\text{K}_{\text{Mc4}}\text{K}_{\text{CoA}}\text{K}_{\text{eqMCKAT}}\text{K}_{\text{Msc4}}}\times y_2,$$

$$c_4 = \frac{\text{Vmckat}\ (\text{T}-\text{S})}{\text{K}_{\text{Mc4}}\text{K}_{\text{CoA}}}\times y_1,$$

$$d_2 = \frac{\mathrm{Vmckat\ Acetyl} \times \mathrm{Acetyl}}{K_{Mc4} K_{CoA} K_{eqMCKAT}} \times y_1,$$

With

$$w_1 = 1 + \frac{T - S}{K_{CoA}} + \frac{\mathrm{Acetyl}}{K_A} + \frac{\mathrm{Acetyl}}{K_A} + \frac{\mathrm{Acetyl} \times (T - S)}{K_A K_{CoA}} + \frac{\mathrm{Acetyl} \times \mathrm{Acetyl}}{K_A \times K_A} + \frac{Q_2}{K_{Q2}} + \frac{Q_2 (T - S)}{K_{Q2} K_{CoA}} + \frac{Q_2 \times \mathrm{Acetyl}}{K_{Q2} K_A}$$

$$w_2 = \frac{1}{K_{Mc4}} - \frac{1}{K_{CoA}} + \frac{T - S}{K_{Mc4} K_{CoA}} + \frac{\mathrm{Acetyl}}{K_{Mc4} K_A} - \frac{\mathrm{Acetyl}}{K_A K_{CoA}} - \frac{Q_2}{K_{Q2} K_{CoA}}$$

$$y_1 = 1 + \frac{Nt - N_D}{K_N} + \frac{N_D}{K_D} + \frac{H_4}{K_{H4}} + \frac{H_4 \times (Nt - N_D)}{K_{H4} K_N} + \frac{H_4 \times N_D}{K_{H4} K_D} + \frac{Q_1}{K_{Q1}} + \frac{Q_1 (Nt - N_D)}{K_{Q1} K_N} + \frac{Q_1 \times N_D}{K_{Q1} K_D}$$

$$y_2 = 1 + \frac{Nt - N_D}{K_N} + \frac{N_D}{K_D}$$
