# Supplementary material for: Bistability in fatty-acid oxidation resulting from substrate inhibition
Source: PLoS Comput Biol. 2021 Aug 12;17(8):e1009259. doi: 10.1371/journal.pcbi.1009259 (PMC8396765; doi:10.1371/journal.pcbi.1009259)

1.Mitochondria Fatty acid Oxidation Kinetic Model - origin and cause of bistability by varying the concentration of C4-KetoacylCoAMAT or C4-AcetoacylCoAMAT where Palmitoyl-CoA is fixed at 120 μM

Definitions of the various functions

In[∘]:= CPT1[sf_, V_, Kms1_, Kms2_, Kmp1_, Kmp2_, Ki1_, Keq_, S1_, S2_, P1_, P2_, I1_, n_] :=

$$\frac{sf*V*\left(\frac{S1*S2}{Kms1*Kms2}-\frac{P1*P2}{Kms1*Kms2*Keq}\right)}{\left(1+\frac{S1}{Kms1}+\frac{P1}{Kmp1}+\left(\frac{I1}{Ki1}\right)^{n}\right)*\left(1+\frac{S2}{Kms2}+\frac{P2}{Kmp2}\right)}$$

In[∘]:= CACT[Vf_, Vr_, Kms1_, Kms2_, Kmp1_, Kmp2_, Kis1_, Kip2_, Keq_, S1_, S2_, P1_, P2_] :=

$$\frac{Vf*\left(S1*S2-\frac{P1*P2}{Keq}\right)}{S1*S2+Kms2*S1+Kms1*S2*\left(1+\frac{P2}{Kip2}\right)+\frac{Vf}{Vr*Keq}*\left(Kmp2*P1*\left(1+\frac{S1}{Kis1}\right)+P2*(Kmp1+P1)\right)}$$

In[∘]:= CPT2[sf_, V_, Kms1_, Kms2_, Kms3_, Kms4_, Kms5_, Kms6_, Kms7_, Kms8_, Kmp1_, Kmp2_, Kmp3_, Kmp4_, Kmp5_, Kmp6_, Kmp7_, Kmp8_, Keq_, S1_, S2_, S3_, S4_, S5_, S6_, S7_, S8_, P1_, P2_, P3_, P4_, P5_, P6_, P7_, P8_] :=

$$\left(sf*V*\left(\frac{S1*S8}{Kms1*Kms8}-\frac{P1*P8}{Kms1*Kms8*Keq}\right)\right)\Big/\left(\left(1+\frac{S1}{Kms1}+\frac{P1}{Kmp1}+\frac{S2}{Kms2}+\frac{P2}{Kmp2}+\frac{S3}{Kms3}+\frac{P3}{Kmp3}+\frac{S4}{Kms4}+\frac{P4}{Kmp4}+\frac{S5}{Kms5}+\frac{P5}{Kmp5}+\frac{S6}{Kms6}+\frac{P6}{Kmp6}+\frac{S7}{Kms7}+\frac{P7}{Kmp7}\right)*\left(1+\frac{S8}{Kms8}+\frac{P8}{Kmp8}\right)\right)$$

In[∘]:= VLCAD[sf_, V_, Kms1_, Kms2_, Kms3_, Kms4_, Kmp1_, Kmp2_, Kmp3_, Kmp4_, Keq_, S1_, S2_, S3_, S4_, P1_, P2_, P3_, P4_] :=

$$\frac{sf*V*\left(\frac{S1*(S4-P4)}{Kms1*Kms4}-\frac{P1*P4}{Kms1*Kms4*Keq}\right)}{\left(1+\frac{S1}{Kms1}+\frac{P1}{Kmp1}+\frac{S2}{Kms2}+\frac{P2}{Kmp2}+\frac{S3}{Kms3}+\frac{P3}{Kmp3}\right)*\left(1+\frac{(S4-P4)}{Kms4}+\frac{P4}{Kmp4}\right)}$$

In[∘]:= LCAD[sf_, V_, Kms1_, Kms2_, Kms3_, Kms4_, Kms5_, Kms6_, Kmp1_, Kmp2_, Kmp3_, Kmp4_, Kmp5_, Kmp6_, Keq_, S1_, S2_, S3_, S4_, S5_, S6_, P1_, P2_, P3_, P4_, P5_, P6_] :=

$$\frac{sf*V*\left(\frac{S1*(S6-P6)}{Kms1*Kms6}-\frac{P1*P6}{Kms1*Kms6*Keq}\right)}{\left(1+\frac{S1}{Kms1}+\frac{P1}{Kmp1}+\frac{S2}{Kms2}+\frac{P2}{Kmp2}+\frac{S3}{Kms3}+\frac{P3}{Kmp3}+\frac{S4}{Kms4}+\frac{P4}{Kmp4}+\frac{S5}{Kms5}+\frac{P5}{Kmp5}\right)*\left(1+\frac{(S6-P6)}{Kms6}+\frac{P6}{Kmp6}\right)}$$

In[∘]:= MCAD[sf_, V_, Kms1_, Kms2_, Kms3_, Kms4_, Kms5_, Kms6_, Kmp1_, Kmp2_, Kmp3_, Kmp4_, Kmp5_, Kmp6_, Keq_, S1_, S2_, S3_, S4_, S5_, S6_, P1_, P2_, P3_, P4_, P5_, P6_] :=

$$\frac{sf*V*\left(\frac{S1*(S6-P6)}{Kms1*Kms6}-\frac{P1*P6}{Kms1*Kms6*Keq}\right)}{\left(1+\frac{S1}{Kms1}+\frac{P1}{Kmp1}+\frac{S2}{Kms2}+\frac{P2}{Kmp2}+\frac{S3}{Kms3}+\frac{P3}{Kmp3}+\frac{S4}{Kms4}+\frac{P4}{Kmp4}+\frac{S5}{Kms5}+\frac{P5}{Kmp5}\right)*\left(1+\frac{(S6-P6)}{Kms6}+\frac{P6}{Kmp6}\right)}$$

In[∘]:= SCAD[sf_, V_, Kms1_, Kms2_, Kms3_, Kmp1_, Kmp2_, Kmp3_, Keq_, S1_, S2_, S3_, P1_, P2_, P3_] :=

$$\frac{sf*V*\left(\frac{S1*(S3-P3)}{Kms1*Kms3}-\frac{P1*P3}{Kms1*Kms3*Keq}\right)}{\left(1+\frac{S1}{Kms1}+\frac{P1}{Kmp1}+\frac{S2}{Kms2}+\frac{P2}{Kmp2}\right)*\left(1+\frac{(S3-P3)}{Kms3}+\frac{P3}{Kmp3}\right)}$$

In[∘]:= CROT[sf_, V_, Kms1_, Kms2_, Kms3_, Kms4_, Kms5_, Kms6_, Kms7_, Kmp1_, Kmp2_, Kmp3_, Kmp4_, Kmp5_, Kmp6_, Kmp7_, Ki1_, Keq_, S1_, S2_, S3_, S4_, S5_, S6_, S7_, P1_, P2_, P3_, P4_, P5_, P6_, P7_, I1_] :=

$$\frac{sf*V*\left(\frac{S1}{Kms1}-\frac{P1}{Kms1*Keq}\right)}{1+\frac{S1}{Kms1}+\frac{P1}{Kmp1}+\frac{S2}{Kms2}+\frac{P2}{Kmp2}+\frac{S3}{Kms3}+\frac{P3}{Kmp3}+\frac{S4}{Kms4}+\frac{P4}{Kmp4}+\frac{S5}{Kms5}+\frac{P5}{Kmp5}+\frac{S6}{Kms6}+\frac{P6}{Kmp6}+\frac{S7}{Kms7}+\frac{P7}{Kmp7}+\frac{I1}{Ki1}}$$

In[∘]:= MSCHAD[sf_, V_, Kms1_, Kms2_, Kms3_, Kms4_, Kms5_, Kms6_, Kms7_, Kms8_, Kmp1_, Kmp2_, Kmp3_, Kmp4_, Kmp5_, Kmp6_, Kmp7_, Kmp8_, Keq_, S1_, S2_, S3_, S4_, S5_, S6_, S7_, S8_, P1_, P2_, P3_, P4_, P5_, P6_, P7_, P8_] :=

$$\left(sf*V*\left(\frac{S1*(S8-P8)}{Kms1*Kms8}-\frac{P1*P8}{Kms1*Kms8*Keq}\right)\right)\Big/\left(\left(1+\frac{S1}{Kms1}+\frac{P1}{Kmp1}+\frac{S2}{Kms2}+\frac{P2}{Kmp2}+\frac{S3}{Kms3}+\frac{P3}{Kmp3}+\frac{S4}{Kms4}+\frac{P4}{Kmp4}+\frac{S5}{Kms5}+\frac{P5}{Kmp5}+\frac{S6}{Kms6}+\frac{P6}{Kmp6}+\frac{S7}{Kms7}+\frac{P7}{Kmp7}\right)*\left(1+\frac{(S8-P8)}{Kms8}+\frac{P8}{Kmp8}\right)\right)$$

In[∘]:= MCKATA[sf_, V_, Kms1_, Kms2_, Kms3_, Kms4_, Kms5_, Kms6_, Kms7_, Kms8_, Kmp1_, Kmp2_, Kmp3_, Kmp4_, Kmp5_, Kmp6_, Kmp7_, Kmp8_, Keq_, S1_, S2_, S3_, S4_, S5_, S6_, S7_, S8_, P1_, P2_, P3_, P4_, P5_, P6_, P7_, P8_] :=

$$\left(sf*V*\left(\frac{S1*S8}{Kms1*Kms8}-\frac{P1*P8}{Kms1*Kms8*Keq}\right)\right)\Big/\left(\left(1+\frac{S1}{Kms1}+\frac{P1}{Kmp1}+\frac{S2}{Kms2}+\frac{P2}{Kmp2}+\frac{S3}{Kms3}+\frac{P3}{Kmp3}+\frac{S4}{Kms4}+\frac{P4}{Kmp4}+\frac{S5}{Kms5}+\frac{P5}{Kmp5}+\frac{S6}{Kms6}+\frac{P6}{Kmp6}+\frac{S7}{Kms7}+\frac{P7}{Kmp7}+\frac{P8}{Kmp8}\right)*\left(1+\frac{S8}{Kms8}+\frac{P8}{Kmp8}\right)\right)$$

In[∘]:= MCKATB[sf_, V_, Kms1_, Kms2_, Kms3_, Kms4_, Kms5_, Kms6_, Kms7_, Kms8_, Kmp1_, Kmp2_, Kmp3_, Kmp4_, Kmp5_, Kmp6_, Kmp7_, Kmp8_, Keq_, S1_, S2_, S3_, S4_, S5_, S6_, S7_, S8_, P1_, P2_, P3_, P4_, P5_, P6_, P7_, P8_] :=

$$\left(sf*V*\left(\frac{S1*S8}{Kms1*Kms8}-\frac{P8*P8}{Kms1*Kms8*Keq}\right)\right)\Big/\left(\left(1+\frac{S1}{Kms1}+\frac{P1}{Kmp1}+\frac{S2}{Kms2}+\frac{P2}{Kmp2}+\frac{S3}{Kms3}+\frac{P3}{Kmp3}+\frac{S4}{Kms4}+\frac{P4}{Kmp4}+\frac{S5}{Kms5}+\frac{P5}{Kmp5}+\frac{S6}{Kms6}+\frac{P6}{Kmp6}+\frac{S7}{Kms7}+\frac{P7}{Kmp7}+\frac{P8}{Kmp8}\right)*\left(1+\frac{S8}{Kms8}+\frac{P8}{Kmp8}\right)\right)$$

In[•]:=
```
MTP[sf_, V_, Kms1_, Kms2_, Kms3_, Kms4_, Kms5_, Kms7_, Kms8_, Kmp1_,
  Kmp2_, Kmp3_, Kmp4_, Kmp5_, Kmp6_, Kmp7_, Kmp8_, Ki1_, Keq_, S1_, S2_,
  S3_, S4_, S5_, S7_, S8_, P1_, P2_, P3_, P4_, P5_, P6_, P7_, P8_, I1_] :=
```

$$\left(sf * V * \left(\frac{S1 * (S7 - P7) * S8}{Kms1 * Kms7 * Kms8} - \frac{P1 * P7 * P8}{Kms1 * Kms7 * Kms8 * Keq}\right)\right) \Big/ \left(\left(1 + \frac{S1}{Kms1} + \frac{P1}{Kmp1} + \frac{S2}{Kms2} + \frac{P2}{Kmp2} + \frac{S3}{Kms3} + \frac{P3}{Kmp3} + \frac{S4}{Kms4} + \frac{P4}{Kmp4} + \frac{S5}{Kms5} + \frac{P5}{Kmp5} + \frac{P6}{Kmp6} + \frac{I1}{Ki1}\right) * \left(1 + \frac{(S7 - P7)}{Kms7} + \frac{P7}{Kmp7}\right) * \left(1 + \frac{S8}{Kms8} + \frac{P8}{Kmp8}\right)\right)$$

In[•]:=
```
RES[Ks_, S_, K1_] := Ks * (S - K1)
```

Define the differential equations

In[•]:= Odes = {

$$C16AcylCarCYT'[t] == \frac{vcpt1C16 - vcactC16}{VCYT},$$

$$C16AcylCarMAT'[t] == \frac{vcactC16 - vcpt2C16}{VMAT},$$

$$C16AcylCoAMAT'[t] == \frac{vcpt2C16 - vvlcadC16 - vlcadC16}{VMAT},$$

$$C16EnoylCoAMAT'[t] == \frac{vvlcadC16 + vlcadC16 - vcrotC16 - vmtpC16}{VMAT},$$

$$C16HydroxyacylCoAMAT'[t] == \frac{vcrotC16 - vmschadC16}{VMAT},$$

$$C16KetoacylCoAMAT'[t] == \frac{vmschadC16 - vmckatC16}{VMAT},$$

$$C14AcylCarCYT'[t] == \frac{-vcactC14}{VCYT},$$

$$C14AcylCarMAT'[t] == \frac{vcactC14 - vcpt2C14}{VMAT},$$

$$C14AcylCoAMAT'[t] == \frac{vcpt2C14 + vmtpC16 + vmckatC16 - vvlcadC14 - vlcadC14}{VMAT},$$

$$C14EnoylCoAMAT'[t] == \frac{vvlcadC14 + vlcadC14 - vcrotC14 - vmtpC14}{VMAT},$$

$$C14HydroxyacylCoAMAT'[t] == \frac{vcrotC14 - vmschadC14}{VMAT},$$

$$C14KetoacylCoAMAT'[t] == \frac{vmschadC14 - vmckatC14}{VMAT},$$

$$C12AcylCarCYT'[t] == \frac{-vcactC12}{VCYT},$$

$$C12AcylCarMAT'[t] == \frac{vcactC12 - vcpt2C12}{VMAT},$$

$$C12AcylCoAMAT'[t] == \frac{vcpt2C12 + vmtpC14 + vmckatC14 - vvlcadC12 - vlcadC12 - vmcadC12}{VMAT},$$

$$C12EnoylCoAMAT'[t] == \frac{vvlcadC12 + vlcadC12 + vmcadC12 - vcrotC12 - vmtpC12}{VMAT},$$

$$C12HydroxyacylCoAMAT'[t] == \frac{vcrotC12 - vmschadC12}{VMAT},$$

$$C12KetoacylCoAMAT'[t] == \frac{vmschadC12 - vmckatC12}{VMAT},$$

$$C10AcylCarCYT'[t] == \frac{-vcactC10}{VCYT},$$

$$C10AcylCarMAT'[t] == \frac{vcactC10 - vcpt2C10}{VMAT},$$

$$C10AcylCoAMAT'[t] == \frac{vcpt2C10 + vmtpC12 + vmckatC12 - vlcadC10 - vmcadC10}{VMAT},$$

$$C10EnoylCoAMAT'[t] == \frac{vlcadC10 + vmcadC10 - vcrotC10 - vmtpC10}{VMAT},$$

$$C10HydroxyacylCoAMAT'[t] == \frac{vcrotC10 - vmschadC10}{VMAT},$$

$$C10KetoacylCoAMAT'[t] == \frac{vmschadC10 - vmckatC10}{VMAT},$$

$$C8AcylCarCYT'[t] == \frac{-vcactC8}{VCYT},$$

$$C8AcylCarMAT'[t] == \frac{vcactC8 - vcpt2C8}{VMAT},$$

$$C8AcylCoAMAT'[t] == \frac{vcpt2C8 + vmtpC10 + vmckatC10 - vlcadC8 - vmcadC8}{VMAT},$$

$$C8EnoylCoAMAT'[t] == \frac{vlcadC8 + vmcadC8 - vcrotC8 - vmtpC8}{VMAT},$$

$$C8HydroxyacylCoAMAT'[t] == \frac{vcrotC8 - vmschadC8}{VMAT},$$

$$C8KetoacylCoAMAT'[t] == \frac{vmschadC8 - vmckatC8}{VMAT},$$

$$C6AcylCarCYT'[t] == \frac{-vcactC6}{VCYT},$$

$$C6AcylCarMAT'[t] == \frac{vcactC6 - vcpt2C6}{VMAT},$$

$$C6AcylCoAMAT'[t] == \frac{vcpt2C6 + vmtpC8 + vmckatC8 - vmcadC6 - vscadC6}{VMAT},$$

$$C6EnoylCoAMAT'[t] == \frac{vmcadC6 + vscadC6 - vcrotC6}{VMAT},$$

$$C6HydroxyacylCoAMAT'[t] == \frac{vcrotC6 - vmschadC6}{VMAT},$$

$$C6KetoacylCoAMAT'[t] == \frac{vmschadC6 - vmckatC6}{VMAT},$$

$$C4AcylCarCYT'[t] == \frac{-vcactC4}{VCYT},$$

$$C4AcylCarMAT'[t] == \frac{vcactC4 - vcpt2C4}{VMAT},$$

$$C4AcylCoAMAT'[t] == \frac{vcpt2C4 + vmckatC6 - vmcadC4 - vscadC4}{VMAT},$$

```
C4EnoylCoAMAT'[t] == (vmcadC4 + vscadC4 - vcrotC4)/VMAT,
C4HydroxyacylCoAMAT'[t] == (vcrotC4 - vmschadC4)/VMAT}; (*,*)
(*C4AcetoacylCoAMAT'[t] == (vmschadC4-vmckatC4)/VMAT};*)

RateEqs = {vcpt1C16 → CPT1[sfcpt1C16, Vcpt1, Kmcpt1C16AcylCoACYT,
    Kmcpt1CarCYT, Kmcpt1C16AcylCarCYT, Kmcpt1CoACYT, Kicpt1MalCoACYT, Keqcpt1,
    C16AcylCoACYT, CarCYT, C16AcylCarCYT[t], CoACYT, MalCoACYT, ncpt1],
  vcactC16 → CACT[Vfcact, Vrcact, KmcactC16AcylCarCYT, KmcactCarMAT,
    KmcactC16AcylCarMAT, KmcactCarCYT, KicactC16AcylCarCYT, KicactCarCYT,
    Keqcact, C16AcylCarCYT[t], CarMAT, C16AcylCarMAT[t], CarCYT],
  vcactC14 → CACT[Vfcact, Vrcact, KmcactC14AcylCarCYT, KmcactCarMAT,
    KmcactC14AcylCarMAT, KmcactCarCYT, KicactC14AcylCarCYT, KicactCarCYT,
    Keqcact, C14AcylCarCYT[t], CarMAT, C14AcylCarMAT[t], CarCYT],
  vcactC12 → CACT[Vfcact, Vrcact, KmcactC12AcylCarCYT, KmcactCarMAT,
    KmcactC12AcylCarMAT, KmcactCarCYT, KicactC12AcylCarCYT, KicactCarCYT,
    Keqcact, C12AcylCarCYT[t], CarMAT, C12AcylCarMAT[t], CarCYT],
  vcactC10 → CACT[Vfcact, Vrcact, KmcactC10AcylCarCYT, KmcactCarMAT,
    KmcactC10AcylCarMAT, KmcactCarCYT, KicactC10AcylCarCYT, KicactCarCYT,
    Keqcact, C10AcylCarCYT[t], CarMAT, C10AcylCarMAT[t], CarCYT],
  vcactC8 → CACT[Vfcact, Vrcact, KmcactC8AcylCarCYT, KmcactCarMAT,
    KmcactC8AcylCarMAT, KmcactCarCYT, KicactC8AcylCarCYT, KicactCarCYT,
    Keqcact, C8AcylCarCYT[t], CarMAT, C8AcylCarMAT[t], CarCYT],
  vcactC6 → CACT[Vfcact, Vrcact, KmcactC6AcylCarCYT, KmcactCarMAT,
    KmcactC6AcylCarMAT, KmcactCarCYT, KicactC6AcylCarCYT, KicactCarCYT,
    Keqcact, C6AcylCarCYT[t], CarMAT, C6AcylCarMAT[t], CarCYT],
  vcactC4 → CACT[Vfcact, Vrcact, KmcactC4AcylCarCYT, KmcactCarMAT,
    KmcactC4AcylCarMAT, KmcactCarCYT, KicactC4AcylCarCYT, KicactCarCYT,
    Keqcact, C4AcylCarCYT[t], CarMAT, C4AcylCarMAT[t], CarCYT],
  vcpt2C16 → CPT2[sfcpt2C16, Vcpt2, Kmcpt2C16AcylCarMAT, Kmcpt2C14AcylCarMAT,
    Kmcpt2C12AcylCarMAT, Kmcpt2C10AcylCarMAT, Kmcpt2C8AcylCarMAT, Kmcpt2C6AcylCarMAT,
    Kmcpt2C4AcylCarMAT, Kmcpt2CoAMAT, Kmcpt2C16AcylCoAMAT, Kmcpt2C14AcylCoAMAT,
    Kmcpt2C12AcylCoAMAT, Kmcpt2C10AcylCoAMAT, Kmcpt2C8AcylCoAMAT, Kmcpt2C6AcylCoAMAT,
    Kmcpt2C4AcylCoAMAT, Kmcpt2CarMAT, Keqcpt2, C16AcylCarMAT[t], C14AcylCarMAT[t],
    C12AcylCarMAT[t], C10AcylCarMAT[t], C8AcylCarMAT[t], C6AcylCarMAT[t],
    C4AcylCarMAT[t], CoAMAT, C16AcylCoAMAT[t], C14AcylCoAMAT[t], C12AcylCoAMAT[t],
    C10AcylCoAMAT[t], C8AcylCoAMAT[t], C6AcylCoAMAT[t], C4AcylCoAMAT[t], CarMAT],
  vcpt2C14 → CPT2[sfcpt2C14, Vcpt2, Kmcpt2C14AcylCarMAT, Kmcpt2C16AcylCarMAT,
    Kmcpt2C12AcylCarMAT, Kmcpt2C10AcylCarMAT, Kmcpt2C8AcylCarMAT, Kmcpt2C6AcylCarMAT,
    Kmcpt2C4AcylCoAMAT, Kmcpt2CoAMAT, Kmcpt2C14AcylCoAMAT, Kmcpt2C16AcylCoAMAT,
    Kmcpt2C12AcylCoAMAT, Kmcpt2C10AcylCoAMAT, Kmcpt2C8AcylCoAMAT, Kmcpt2C6AcylCoAMAT,
    Kmcpt2C4AcylCoAMAT, Kmcpt2CarMAT, Keqcpt2, C14AcylCarMAT[t], C16AcylCarMAT[t],
    C12AcylCarMAT[t], C10AcylCarMAT[t], C8AcylCarMAT[t], C6AcylCarMAT[t],
    C4AcylCarMAT[t], CoAMAT, C14AcylCoAMAT[t], C16AcylCoAMAT[t], C12AcylCoAMAT[t],
    C10AcylCoAMAT[t], C8AcylCoAMAT[t], C6AcylCoAMAT[t], C4AcylCoAMAT[t], CarMAT],
  vcpt2C12 → CPT2[sfcpt2C12, Vcpt2, Kmcpt2C12AcylCarMAT, Kmcpt2C16AcylCarMAT,
    Kmcpt2C14AcylCarMAT, Kmcpt2C10AcylCarMAT, Kmcpt2C8AcylCarMAT, Kmcpt2C6AcylCarMAT,
    Kmcpt2C4AcylCarMAT, Kmcpt2CoAMAT, Kmcpt2C12AcylCoAMAT, Kmcpt2C16AcylCoAMAT,
    Kmcpt2C14AcylCoAMAT, Kmcpt2C10AcylCoAMAT, Kmcpt2C8AcylCoAMAT, Kmcpt2C6AcylCoAMAT,
    Kmcpt2C4AcylCoAMAT, Kmcpt2CarMAT, Keqcpt2, C12AcylCarMAT[t], C16AcylCarMAT[t],
    C14AcylCarMAT[t], C10AcylCarMAT[t], C8AcylCarMAT[t], C6AcylCarMAT[t],
    C4AcylCarMAT[t], CoAMAT, C12AcylCoAMAT[t], C16AcylCoAMAT[t], C14AcylCoAMAT[t],
    C10AcylCoAMAT[t], C8AcylCoAMAT[t], C6AcylCoAMAT[t], C4AcylCoAMAT[t], CarMAT],
```

```
vcpt2C10 → CPT2[sfcpt2C10, Vcpt2, Kmcpt2C10AcylCarMAT, Kmcpt2C16AcylCarMAT,
  Kmcpt2C14AcylCarMAT, Kmcpt2C12AcylCarMAT, Kmcpt2C8AcylCarMAT, Kmcpt2C6AcylCarMAT,
  Kmcpt2C4AcylCarMAT, Kmcpt2CoAMAT, Kmcpt2C10AcylCoAMAT, Kmcpt2C16AcylCoAMAT,
  Kmcpt2C14AcylCoAMAT, Kmcpt2C12AcylCoAMAT, Kmcpt2C8AcylCoAMAT, Kmcpt2C6AcylCoAMAT,
  Kmcpt2C4AcylCoAMAT, Kmcpt2CarMAT, Keqcpt2, C10AcylCarMAT[t], C16AcylCarMAT[t],
  C14AcylCarMAT[t], C12AcylCarMAT[t], C8AcylCarMAT[t], C6AcylCarMAT[t],
  C4AcylCarMAT[t], CoAMAT, C10AcylCoAMAT[t], C16AcylCoAMAT[t], C14AcylCoAMAT[t],
  C12AcylCoAMAT[t], C8AcylCoAMAT[t], C6AcylCoAMAT[t], C4AcylCoAMAT[t], CarMAT],
vcpt2C8 → CPT2[sfcpt2C8, Vcpt2, Kmcpt2C8AcylCarMAT, Kmcpt2C16AcylCarMAT,
  Kmcpt2C14AcylCarMAT, Kmcpt2C12AcylCarMAT, Kmcpt2C10AcylCarMAT, Kmcpt2C6AcylCarMAT,
  Kmcpt2C4AcylCarMAT, Kmcpt2CoAMAT, Kmcpt2C8AcylCoAMAT, Kmcpt2C16AcylCoAMAT,
  Kmcpt2C14AcylCoAMAT, Kmcpt2C12AcylCoAMAT, Kmcpt2C10AcylCoAMAT, Kmcpt2C6AcylCoAMAT,
  Kmcpt2C4AcylCoAMAT, Kmcpt2CarMAT, Keqcpt2, C8AcylCarMAT[t], C16AcylCarMAT[t],
  C14AcylCarMAT[t], C12AcylCarMAT[t], C10AcylCarMAT[t], C6AcylCarMAT[t],
  C4AcylCarMAT[t], CoAMAT, C8AcylCoAMAT[t], C16AcylCoAMAT[t], C14AcylCoAMAT[t],
  C12AcylCoAMAT[t], C10AcylCoAMAT[t], C6AcylCoAMAT[t], C4AcylCoAMAT[t], CarMAT],
vcpt2C6 → CPT2[sfcpt2C6, Vcpt2, Kmcpt2C6AcylCarMAT, Kmcpt2C16AcylCarMAT,
  Kmcpt2C14AcylCarMAT, Kmcpt2C12AcylCarMAT, Kmcpt2C10AcylCarMAT, Kmcpt2C8AcylCarMAT,
  Kmcpt2C4AcylCarMAT, Kmcpt2CoAMAT, Kmcpt2C6AcylCoAMAT, Kmcpt2C16AcylCoAMAT,
  Kmcpt2C14AcylCoAMAT, Kmcpt2C12AcylCoAMAT, Kmcpt2C10AcylCoAMAT, Kmcpt2C8AcylCoAMAT,
  Kmcpt2C4AcylCoAMAT, Kmcpt2CarMAT, Keqcpt2, C6AcylCarMAT[t], C16AcylCarMAT[t],
  C14AcylCarMAT[t], C12AcylCarMAT[t], C10AcylCarMAT[t], C8AcylCarMAT[t],
  C4AcylCarMAT[t], CoAMAT, C6AcylCoAMAT[t], C16AcylCoAMAT[t], C14AcylCoAMAT[t],
  C12AcylCoAMAT[t], C10AcylCoAMAT[t], C8AcylCoAMAT[t], C4AcylCoAMAT[t], CarMAT],
vcpt2C4 → CPT2[sfcpt2C4, Vcpt2, Kmcpt2C4AcylCarMAT, Kmcpt2C16AcylCarMAT,
  Kmcpt2C14AcylCarMAT, Kmcpt2C12AcylCarMAT, Kmcpt2C10AcylCarMAT, Kmcpt2C8AcylCarMAT,
  Kmcpt2C6AcylCarMAT, Kmcpt2CoAMAT, Kmcpt2C4AcylCoAMAT, Kmcpt2C16AcylCoAMAT,
  Kmcpt2C14AcylCoAMAT, Kmcpt2C12AcylCoAMAT, Kmcpt2C10AcylCoAMAT, Kmcpt2C8AcylCoAMAT,
  Kmcpt2C6AcylCoAMAT, Kmcpt2CarMAT, Keqcpt2, C4AcylCarMAT[t], C16AcylCarMAT[t],
  C14AcylCarMAT[t], C12AcylCarMAT[t], C10AcylCarMAT[t], C8AcylCarMAT[t],
  C6AcylCarMAT[t], CoAMAT, C4AcylCoAMAT[t], C16AcylCoAMAT[t], C14AcylCoAMAT[t],
  C12AcylCoAMAT[t], C10AcylCoAMAT[t], C8AcylCoAMAT[t], C6AcylCoAMAT[t], CarMAT],
vvlcadC16 → VLCAD[sfvlcadC16, Vvlcad, KmvlcadC16AcylCoAMAT, KmvlcadC14AcylCoAMAT,
  KmvlcadC12AcylCoAMAT, KmvlcadFAD, KmvlcadC16EnoylCoAMAT,
  KmvlcadC14EnoylCoAMAT, KmvlcadC12EnoylCoAMAT, KmvlcadFADH, Keqvlcad,
  C16AcylCoAMAT[t], C14AcylCoAMAT[t], C12AcylCoAMAT[t], FADtMAT,
  C16EnoylCoAMAT[t], C14EnoylCoAMAT[t], C12EnoylCoAMAT[t], FADHMAT],
vvlcadC14 → VLCAD[sfvlcadC14, Vvlcad, KmvlcadC14AcylCoAMAT, KmvlcadC16AcylCoAMAT,
  KmvlcadC12AcylCoAMAT, KmvlcadFAD, KmvlcadC14EnoylCoAMAT,
  KmvlcadC16EnoylCoAMAT, KmvlcadC12EnoylCoAMAT, KmvlcadFADH, Keqvlcad,
  C14AcylCoAMAT[t], C16AcylCoAMAT[t], C12AcylCoAMAT[t], FADtMAT,
  C14EnoylCoAMAT[t], C16EnoylCoAMAT[t], C12EnoylCoAMAT[t], FADHMAT],
vvlcadC12 → VLCAD[sfvlcadC12, Vvlcad, KmvlcadC12AcylCoAMAT, KmvlcadC16AcylCoAMAT,
  KmvlcadC14AcylCoAMAT, KmvlcadFAD, KmvlcadC12EnoylCoAMAT,
  KmvlcadC16EnoylCoAMAT, KmvlcadC14EnoylCoAMAT, KmvlcadFADH, Keqvlcad,
  C12AcylCoAMAT[t], C16AcylCoAMAT[t], C14AcylCoAMAT[t], FADtMAT,
  C12EnoylCoAMAT[t], C16EnoylCoAMAT[t], C14EnoylCoAMAT[t], FADHMAT],
vlcadC16 → LCAD[sflcadC16, Vlcad, KmlcadC16AcylCoAMAT, KmlcadC14AcylCoAMAT,
  KmlcadC12AcylCoAMAT, KmlcadC10AcylCoAMAT, KmlcadC8AcylCoAMAT, KmlcadFAD,
  KmlcadC16EnoylCoAMAT, KmlcadC14EnoylCoAMAT, KmlcadC12EnoylCoAMAT,
  KmlcadC10EnoylCoAMAT, KmlcadC8EnoylCoAMAT, KmlcadFADH, Keqlcad,
  C16AcylCoAMAT[t], C14AcylCoAMAT[t], C12AcylCoAMAT[t], C10AcylCoAMAT[t],
  C8AcylCoAMAT[t], FADtMAT, C16EnoylCoAMAT[t], C14EnoylCoAMAT[t],
  C12EnoylCoAMAT[t], C10EnoylCoAMAT[t], C8EnoylCoAMAT[t], FADHMAT],
vlcadC14 → LCAD[sflcadC14, Vlcad, KmlcadC14AcylCoAMAT, KmlcadC16AcylCoAMAT,
```

```
   KmlcadC12AcylCoAMAT, KmlcadC10AcylCoAMAT, KmlcadC8AcylCoAMAT, KmlcadFAD,
   KmlcadC14EnoylCoAMAT, KmlcadC16EnoylCoAMAT, KmlcadC12EnoylCoAMAT,
   KmlcadC10EnoylCoAMAT, KmlcadC8EnoylCoAMAT, KmlcadFADH, Keqlcad,
   C14AcylCoAMAT[t], C16AcylCoAMAT[t], C12AcylCoAMAT[t], C10AcylCoAMAT[t],
   C8AcylCoAMAT[t], FADtMAT, C14EnoylCoAMAT[t], C16EnoylCoAMAT[t],
   C12EnoylCoAMAT[t], C10EnoylCoAMAT[t], C8EnoylCoAMAT[t], FADHMAT],
vlcadC12 → LCAD[sflcadC12, Vlcad, KmlcadC12AcylCoAMAT, KmlcadC16AcylCoAMAT,
   KmlcadC14AcylCoAMAT, KmlcadC10AcylCoAMAT, KmlcadC8AcylCoAMAT, KmlcadFAD,
   KmlcadC12EnoylCoAMAT, KmlcadC16EnoylCoAMAT, KmlcadC14EnoylCoAMAT,
   KmlcadC10EnoylCoAMAT, KmlcadC8EnoylCoAMAT, KmlcadFADH, Keqlcad,
   C12AcylCoAMAT[t], C16AcylCoAMAT[t], C14AcylCoAMAT[t], C10AcylCoAMAT[t],
   C8AcylCoAMAT[t], FADtMAT, C14EnoylCoAMAT[t], C16EnoylCoAMAT[t],
   C14EnoylCoAMAT[t], C10EnoylCoAMAT[t], C8EnoylCoAMAT[t], FADHMAT],
vlcadC10 → LCAD[sflcadC10, Vlcad, KmlcadC10AcylCoAMAT, KmlcadC16AcylCoAMAT,
   KmlcadC14AcylCoAMAT, KmlcadC12AcylCoAMAT, KmlcadC8AcylCoAMAT, KmlcadFAD,
   KmlcadC10EnoylCoAMAT, KmlcadC16EnoylCoAMAT, KmlcadC14EnoylCoAMAT,
   KmlcadC12EnoylCoAMAT, KmlcadC8EnoylCoAMAT, KmlcadFADH, Keqlcad,
   C10AcylCoAMAT[t], C16AcylCoAMAT[t], C14AcylCoAMAT[t], C12AcylCoAMAT[t],
   C8AcylCoAMAT[t], FADtMAT, C10EnoylCoAMAT[t], C16EnoylCoAMAT[t],
   C14EnoylCoAMAT[t], C12EnoylCoAMAT[t], C8EnoylCoAMAT[t], FADHMAT],
vlcadC8 → LCAD[sflcadC8, Vlcad, KmlcadC8AcylCoAMAT, KmlcadC16AcylCoAMAT,
   KmlcadC14AcylCoAMAT, KmlcadC12AcylCoAMAT, KmlcadC10AcylCoAMAT, KmlcadFAD,
   KmlcadC8EnoylCoAMAT, KmlcadC16EnoylCoAMAT, KmlcadC14EnoylCoAMAT,
   KmlcadC12EnoylCoAMAT, KmlcadC10EnoylCoAMAT, KmlcadFADH, Keqlcad,
   C8AcylCoAMAT[t], C16AcylCoAMAT[t], C14AcylCoAMAT[t], C12AcylCoAMAT[t],
   C10AcylCoAMAT[t], FADtMAT, C8EnoylCoAMAT[t], C16EnoylCoAMAT[t],
   C14EnoylCoAMAT[t], C12EnoylCoAMAT[t], C10EnoylCoAMAT[t], FADHMAT],
vmcadC12 → MCAD[sfmcadC12, Vmcad, KmmcadC12AcylCoAMAT, KmmcadC10AcylCoAMAT,
   KmmcadC8AcylCoAMAT, KmmcadC6AcylCoAMAT, KmmcadC4AcylCoAMAT, KmmcadFAD,
   KmmcadC12EnoylCoAMAT, KmmcadC10EnoylCoAMAT, KmmcadC8EnoylCoAMAT,
   KmmcadC6EnoylCoAMAT, KmmcadC4EnoylCoAMAT, KmmcadFADH, Keqmcad,
   C12AcylCoAMAT[t], C10AcylCoAMAT[t], C8AcylCoAMAT[t], C6AcylCoAMAT[t],
   C4AcylCoAMAT[t], FADtMAT, C12EnoylCoAMAT[t], C10EnoylCoAMAT[t],
   C8EnoylCoAMAT[t], C6EnoylCoAMAT[t], C4EnoylCoAMAT[t], FADHMAT],
vmcadC10 → MCAD[sfmcadC10, Vmcad, KmmcadC10AcylCoAMAT, KmmcadC12AcylCoAMAT,
   KmmcadC8AcylCoAMAT, KmmcadC6AcylCoAMAT, KmmcadC4AcylCoAMAT, KmmcadFAD,
   KmmcadC10EnoylCoAMAT, KmmcadC12EnoylCoAMAT, KmmcadC8EnoylCoAMAT,
   KmmcadC6EnoylCoAMAT, KmmcadC4EnoylCoAMAT, KmmcadFADH, Keqmcad,
   C10AcylCoAMAT[t], C12AcylCoAMAT[t], C8AcylCoAMAT[t], C6AcylCoAMAT[t],
   C4AcylCoAMAT[t], FADtMAT, C10EnoylCoAMAT[t], C12EnoylCoAMAT[t],
   C8EnoylCoAMAT[t], C6EnoylCoAMAT[t], C4EnoylCoAMAT[t], FADHMAT],
vmcadC8 → MCAD[sfmcadC8, Vmcad, KmmcadC8AcylCoAMAT, KmmcadC12AcylCoAMAT,
   KmmcadC10AcylCoAMAT, KmmcadC6AcylCoAMAT, KmmcadC4AcylCoAMAT, KmmcadFAD,
   KmmcadC8EnoylCoAMAT, KmmcadC12EnoylCoAMAT, KmmcadC10EnoylCoAMAT,
   KmmcadC6EnoylCoAMAT, KmmcadC4EnoylCoAMAT, KmmcadFADH, Keqmcad,
   C8AcylCoAMAT[t], C12AcylCoAMAT[t], C10AcylCoAMAT[t], C6AcylCoAMAT[t],
   C4AcylCoAMAT[t], FADtMAT, C8EnoylCoAMAT[t], C12EnoylCoAMAT[t],
   C10EnoylCoAMAT[t], C6EnoylCoAMAT[t], C4EnoylCoAMAT[t], FADHMAT],
vmcadC6 → MCAD[sfmcadC6, Vmcad, KmmcadC6AcylCoAMAT, KmmcadC12AcylCoAMAT,
   KmmcadC10AcylCoAMAT, KmmcadC8AcylCoAMAT, KmmcadC4AcylCoAMAT, KmmcadFAD,
   KmmcadC6EnoylCoAMAT, KmmcadC12EnoylCoAMAT, KmmcadC10EnoylCoAMAT,
   KmmcadC8EnoylCoAMAT, KmmcadC4EnoylCoAMAT, KmmcadFADH, Keqmcad,
   C6AcylCoAMAT[t], C12AcylCoAMAT[t], C10AcylCoAMAT[t], C8AcylCoAMAT[t],
   C4AcylCoAMAT[t], FADtMAT, C6EnoylCoAMAT[t], C12EnoylCoAMAT[t],
   C10EnoylCoAMAT[t], C8EnoylCoAMAT[t], C4EnoylCoAMAT[t], FADHMAT],
```

```
vmcadC4 → MCAD[sfmcadC4, Vmcad, KmmcadC4AcylCoAMAT, KmmcadC12AcylCoAMAT,
  KmmcadC10AcylCoAMAT, KmmcadC8AcylCoAMAT, KmmcadC6AcylCoAMAT, KmmcadFAD,
  KmmcadC4EnoylCoAMAT, KmmcadC12EnoylCoAMAT, KmmcadC10EnoylCoAMAT,
  KmmcadC8EnoylCoAMAT, KmmcadC6EnoylCoAMAT, KmmcadFADH, Keqmcad,
  C4AcylCoAMAT[t], C12AcylCoAMAT[t], C10AcylCoAMAT[t], C8AcylCoAMAT[t],
  C6AcylCoAMAT[t], FADtMAT, C4EnoylCoAMAT[t], C12EnoylCoAMAT[t],
  C10EnoylCoAMAT[t], C8EnoylCoAMAT[t], C6EnoylCoAMAT[t], FADHMAT],
vscadC6 → SCAD[sfscadC6, Vscad, KmscadC6AcylCoAMAT, KmscadC4AcylCoAMAT, KmscadFAD,
  KmscadC6EnoylCoAMAT, KmscadC4EnoylCoAMAT, KmscadFADH, Keqscad, C6AcylCoAMAT[t],
  C4AcylCoAMAT[t], FADtMAT, C6EnoylCoAMAT[t], C4EnoylCoAMAT[t], FADHMAT],
vscadC4 → SCAD[sfscadC4, Vscad, KmscadC4AcylCoAMAT, KmscadC6AcylCoAMAT, KmscadFAD,
  KmscadC4EnoylCoAMAT, KmscadC6EnoylCoAMAT, KmscadFADH, Keqscad, C4AcylCoAMAT[t],
  C6AcylCoAMAT[t], FADtMAT, C4EnoylCoAMAT[t], C6EnoylCoAMAT[t], FADHMAT],
vcrotC16 → CROT[sfcrotC16, Vcrot, KmcrotC16EnoylCoAMAT, KmcrotC14EnoylCoAMAT,
  KmcrotC12EnoylCoAMAT, KmcrotC10EnoylCoAMAT, KmcrotC8EnoylCoAMAT,
  KmcrotC6EnoylCoAMAT, KmcrotC4EnoylCoAMAT, KmcrotC16HydroxyacylCoAMAT,
  KmcrotC14HydroxyacylCoAMAT, KmcrotC12HydroxyacylCoAMAT,
  KmcrotC10HydroxyacylCoAMAT, KmcrotC8HydroxyacylCoAMAT,
  KmcrotC6HydroxyacylCoAMAT, KmcrotC4HydroxyacylCoAMAT, KicrotC4AcetoacylCoA,
  Keqcrot, C16EnoylCoAMAT[t], C14EnoylCoAMAT[t], C12EnoylCoAMAT[t],
  C10EnoylCoAMAT[t], C8EnoylCoAMAT[t], C6EnoylCoAMAT[t],
  C4EnoylCoAMAT[t], C16HydroxyacylCoAMAT[t], C14HydroxyacylCoAMAT[t],
  C12HydroxyacylCoAMAT[t], C10HydroxyacylCoAMAT[t], C8HydroxyacylCoAMAT[t],
  C6HydroxyacylCoAMAT[t], C4HydroxyacylCoAMAT[t], C4AcetoacylCoAMAT],
vcrotC14 → CROT[sfcrotC14, Vcrot, KmcrotC14EnoylCoAMAT, KmcrotC16EnoylCoAMAT,
  KmcrotC12EnoylCoAMAT, KmcrotC10EnoylCoAMAT, KmcrotC8EnoylCoAMAT,
  KmcrotC6EnoylCoAMAT, KmcrotC4EnoylCoAMAT, KmcrotC14HydroxyacylCoAMAT,
  KmcrotC16HydroxyacylCoAMAT, KmcrotC12HydroxyacylCoAMAT,
  KmcrotC10HydroxyacylCoAMAT, KmcrotC8HydroxyacylCoAMAT,
  KmcrotC6HydroxyacylCoAMAT, KmcrotC4HydroxyacylCoAMAT, KicrotC4AcetoacylCoA,
  Keqcrot, C14EnoylCoAMAT[t], C16EnoylCoAMAT[t], C12EnoylCoAMAT[t],
  C10EnoylCoAMAT[t], C8EnoylCoAMAT[t], C6EnoylCoAMAT[t],
  C4EnoylCoAMAT[t], C14HydroxyacylCoAMAT[t], C16HydroxyacylCoAMAT[t],
  C12HydroxyacylCoAMAT[t], C10HydroxyacylCoAMAT[t], C8HydroxyacylCoAMAT[t],
  C6HydroxyacylCoAMAT[t], C4HydroxyacylCoAMAT[t], C4AcetoacylCoAMAT],
vcrotC12 → CROT[sfcrotC12, Vcrot, KmcrotC12EnoylCoAMAT, KmcrotC16EnoylCoAMAT,
  KmcrotC14EnoylCoAMAT, KmcrotC10EnoylCoAMAT, KmcrotC8EnoylCoAMAT,
  KmcrotC6EnoylCoAMAT, KmcrotC4EnoylCoAMAT, KmcrotC12HydroxyacylCoAMAT,
  KmcrotC16HydroxyacylCoAMAT, KmcrotC14HydroxyacylCoAMAT,
  KmcrotC10HydroxyacylCoAMAT, KmcrotC8HydroxyacylCoAMAT,
  KmcrotC6HydroxyacylCoAMAT, KmcrotC4HydroxyacylCoAMAT, KicrotC4AcetoacylCoA,
  Keqcrot, C12EnoylCoAMAT[t], C16EnoylCoAMAT[t], C14EnoylCoAMAT[t],
  C10EnoylCoAMAT[t], C8EnoylCoAMAT[t], C6EnoylCoAMAT[t],
  C4EnoylCoAMAT[t], C12HydroxyacylCoAMAT[t], C16HydroxyacylCoAMAT[t],
  C14HydroxyacylCoAMAT[t], C10HydroxyacylCoAMAT[t], C8HydroxyacylCoAMAT[t],
  C6HydroxyacylCoAMAT[t], C4HydroxyacylCoAMAT[t], C4AcetoacylCoAMAT],
vcrotC10 → CROT[sfcrotC10, Vcrot, KmcrotC10EnoylCoAMAT, KmcrotC16EnoylCoAMAT,
  KmcrotC14EnoylCoAMAT, KmcrotC12EnoylCoAMAT, KmcrotC8EnoylCoAMAT,
  KmcrotC6EnoylCoAMAT, KmcrotC4EnoylCoAMAT, KmcrotC10HydroxyacylCoAMAT,
  KmcrotC16HydroxyacylCoAMAT, KmcrotC14HydroxyacylCoAMAT,
  KmcrotC12HydroxyacylCoAMAT, KmcrotC8HydroxyacylCoAMAT,
  KmcrotC6HydroxyacylCoAMAT, KmcrotC4HydroxyacylCoAMAT, KicrotC4AcetoacylCoA,
  Keqcrot, C10EnoylCoAMAT[t], C16EnoylCoAMAT[t], C14EnoylCoAMAT[t],
  C12EnoylCoAMAT[t], C8EnoylCoAMAT[t], C6EnoylCoAMAT[t],
  C4EnoylCoAMAT[t], C10HydroxyacylCoAMAT[t], C16HydroxyacylCoAMAT[t],
```

```
    C14HydroxyacylCoAMAT[t], C12HydroxyacylCoAMAT[t], C8HydroxyacylCoAMAT[t],
    C6HydroxyacylCoAMAT[t], C4HydroxyacylCoAMAT[t], C4AcetoacylCoAMAT],
  vcrotC8 → CROT[sfcrotC8, Vcrot, KmcrotC8EnoylCoAMAT, KmcrotC16EnoylCoAMAT,
    KmcrotC14EnoylCoAMAT, KmcrotC12EnoylCoAMAT, KmcrotC10EnoylCoAMAT,
    KmcrotC6EnoylCoAMAT, KmcrotC4EnoylCoAMAT, KmcrotC8HydroxyacylCoAMAT,
    KmcrotC16HydroxyacylCoAMAT, KmcrotC14HydroxyacylCoAMAT,
    KmcrotC12HydroxyacylCoAMAT, KmcrotC10HydroxyacylCoAMAT,
    KmcrotC6HydroxyacylCoAMAT, KmcrotC4HydroxyacylCoAMAT, KicrotC4AcetoacylCoA,
    Keqcrot, C8EnoylCoAMAT[t], C16EnoylCoAMAT[t], C14EnoylCoAMAT[t],
    C12EnoylCoAMAT[t], C10EnoylCoAMAT[t], C6EnoylCoAMAT[t],
    C4EnoylCoAMAT[t], C8HydroxyacylCoAMAT[t], C16HydroxyacylCoAMAT[t],
    C14HydroxyacylCoAMAT[t], C12HydroxyacylCoAMAT[t], C10HydroxyacylCoAMAT[t],
    C6HydroxyacylCoAMAT[t], C4HydroxyacylCoAMAT[t], C4AcetoacylCoAMAT],
  vcrotC6 → CROT[sfcrotC6, Vcrot, KmcrotC6EnoylCoAMAT, KmcrotC16EnoylCoAMAT,
    KmcrotC14EnoylCoAMAT, KmcrotC12EnoylCoAMAT, KmcrotC10EnoylCoAMAT,
    KmcrotC8EnoylCoAMAT, KmcrotC4EnoylCoAMAT, KmcrotC6HydroxyacylCoAMAT,
    KmcrotC16HydroxyacylCoAMAT, KmcrotC14HydroxyacylCoAMAT,
    KmcrotC12HydroxyacylCoAMAT, KmcrotC10HydroxyacylCoAMAT,
    KmcrotC8HydroxyacylCoAMAT, KmcrotC4HydroxyacylCoAMAT, KicrotC4AcetoacylCoA,
    Keqcrot, C6EnoylCoAMAT[t], C16EnoylCoAMAT[t], C14EnoylCoAMAT[t],
    C12EnoylCoAMAT[t], C10EnoylCoAMAT[t], C8EnoylCoAMAT[t],
    C4EnoylCoAMAT[t], C6HydroxyacylCoAMAT[t], C16HydroxyacylCoAMAT[t],
    C14HydroxyacylCoAMAT[t], C12HydroxyacylCoAMAT[t], C10HydroxyacylCoAMAT[t],
    C8HydroxyacylCoAMAT[t], C4HydroxyacylCoAMAT[t], C4AcetoacylCoAMAT],
  vcrotC4 → CROT[sfcrotC4, Vcrot, KmcrotC4EnoylCoAMAT, KmcrotC16EnoylCoAMAT,
    KmcrotC14EnoylCoAMAT, KmcrotC12EnoylCoAMAT, KmcrotC10EnoylCoAMAT,
    KmcrotC8EnoylCoAMAT, KmcrotC6EnoylCoAMAT, KmcrotC4HydroxyacylCoAMAT,
    KmcrotC16HydroxyacylCoAMAT, KmcrotC14HydroxyacylCoAMAT,
    KmcrotC12HydroxyacylCoAMAT, KmcrotC10HydroxyacylCoAMAT,
    KmcrotC8HydroxyacylCoAMAT, KmcrotC6HydroxyacylCoAMAT, KicrotC4AcetoacylCoA,
    Keqcrot, C4EnoylCoAMAT[t], C16EnoylCoAMAT[t], C14EnoylCoAMAT[t],
    C12EnoylCoAMAT[t], C10EnoylCoAMAT[t], C8EnoylCoAMAT[t],
    C6EnoylCoAMAT[t], C4HydroxyacylCoAMAT[t], C16HydroxyacylCoAMAT[t],
    C14HydroxyacylCoAMAT[t], C12HydroxyacylCoAMAT[t], C10HydroxyacylCoAMAT[t],
    C8HydroxyacylCoAMAT[t], C6HydroxyacylCoAMAT[t], C4AcetoacylCoAMAT],
  vmschadC16 → MSCHAD[sfmschadC16, Vmschad, KmmschadC16HydroxyacylCoAMAT,
    KmmschadC14HydroxyacylCoAMAT, KmmschadC12HydroxyacylCoAMAT,
    KmmschadC10HydroxyacylCoAMAT, KmmschadC8HydroxyacylCoAMAT,
    KmmschadC6HydroxyacylCoAMAT, KmmschadC4HydroxyacylCoAMAT,
    KmmschadNADMAT, KmmschadC16KetoacylCoAMAT, KmmschadC14KetoacylCoAMAT,
    KmmschadC12KetoacylCoAMAT, KmmschadC10KetoacylCoAMAT, KmmschadC8KetoacylCoAMAT,
    KmmschadC6KetoacylCoAMAT, KmmschadC4AcetoacylCoAMAT, KmmschadNADHMAT,
    Keqmschad, C16HydroxyacylCoAMAT[t], C14HydroxyacylCoAMAT[t],
    C12HydroxyacylCoAMAT[t], C10HydroxyacylCoAMAT[t], C8HydroxyacylCoAMAT[t],
    C6HydroxyacylCoAMAT[t], C4HydroxyacylCoAMAT[t], NADtMAT, C16KetoacylCoAMAT[t],
    C14KetoacylCoAMAT[t], C12KetoacylCoAMAT[t], C10KetoacylCoAMAT[t],
    C8KetoacylCoAMAT[t], C6KetoacylCoAMAT[t], C4AcetoacylCoAMAT, NADHMAT],
  vmschadC14 → MSCHAD[sfmschadC14, Vmschad, KmmschadC14HydroxyacylCoAMAT,
    KmmschadC16HydroxyacylCoAMAT, KmmschadC12HydroxyacylCoAMAT,
    KmmschadC10HydroxyacylCoAMAT, KmmschadC8HydroxyacylCoAMAT,
    KmmschadC6HydroxyacylCoAMAT, KmmschadC4HydroxyacylCoAMAT,
    KmmschadNADMAT, KmmschadC14KetoacylCoAMAT, KmmschadC16KetoacylCoAMAT,
    KmmschadC12KetoacylCoAMAT, KmmschadC10KetoacylCoAMAT, KmmschadC8KetoacylCoAMAT,
    KmmschadC6KetoacylCoAMAT, KmmschadC4AcetoacylCoAMAT, KmmschadNADHMAT,
    Keqmschad, C14HydroxyacylCoAMAT[t], C16HydroxyacylCoAMAT[t],
```

```
    C12HydroxyacylCoAMAT[t], C10HydroxyacylCoAMAT[t], C8HydroxyacylCoAMAT[t],
    C6HydroxyacylCoAMAT[t], C4HydroxyacylCoAMAT[t], NADtMAT, C14KetoacylCoAMAT[t],
    C16KetoacylCoAMAT[t], C12KetoacylCoAMAT[t], C10KetoacylCoAMAT[t],
    C8KetoacylCoAMAT[t], C6KetoacylCoAMAT[t], C4AcetoacylCoAMAT, NADHMAT],
  vmschadC12 → MSCHAD[sfmschadC12, Vmschad, KmmschadC12HydroxyacylCoAMAT,
    KmmschadC16HydroxyacylCoAMAT, KmmschadC14HydroxyacylCoAMAT,
    KmmschadC10HydroxyacylCoAMAT, KmmschadC8HydroxyacylCoAMAT,
    KmmschadC6HydroxyacylCoAMAT, KmmschadC4HydroxyacylCoAMAT,
    KmmschadNADMAT, KmmschadC12KetoacylCoAMAT, KmmschadC16KetoacylCoAMAT,
    KmmschadC14KetoacylCoAMAT, KmmschadC10KetoacylCoAMAT, KmmschadC8KetoacylCoAMAT,
    KmmschadC6KetoacylCoAMAT, KmmschadC4AcetoacylCoAMAT, KmmschadNADHMAT,
    Keqmschad, C12HydroxyacylCoAMAT[t], C16HydroxyacylCoAMAT[t],
    C14HydroxyacylCoAMAT[t], C10HydroxyacylCoAMAT[t], C8HydroxyacylCoAMAT[t],
    C6HydroxyacylCoAMAT[t], C4HydroxyacylCoAMAT[t], NADtMAT, C12KetoacylCoAMAT[t],
    C16KetoacylCoAMAT[t], C14KetoacylCoAMAT[t], C10KetoacylCoAMAT[t],
    C8KetoacylCoAMAT[t], C6KetoacylCoAMAT[t], C4AcetoacylCoAMAT, NADHMAT],
  vmschadC10 → MSCHAD[sfmschadC10, Vmschad, KmmschadC10HydroxyacylCoAMAT,
    KmmschadC16HydroxyacylCoAMAT, KmmschadC14HydroxyacylCoAMAT,
    KmmschadC12HydroxyacylCoAMAT, KmmschadC8HydroxyacylCoAMAT,
    KmmschadC6HydroxyacylCoAMAT, KmmschadC4HydroxyacylCoAMAT,
    KmmschadNADMAT, KmmschadC10KetoacylCoAMAT, KmmschadC16KetoacylCoAMAT,
    KmmschadC14KetoacylCoAMAT, KmmschadC12KetoacylCoAMAT, KmmschadC8KetoacylCoAMAT,
    KmmschadC6KetoacylCoAMAT, KmmschadC4AcetoacylCoAMAT, KmmschadNADHMAT,
    Keqmschad, C10HydroxyacylCoAMAT[t], C16HydroxyacylCoAMAT[t],
    C14HydroxyacylCoAMAT[t], C12HydroxyacylCoAMAT[t], C8HydroxyacylCoAMAT[t],
    C6HydroxyacylCoAMAT[t], C4HydroxyacylCoAMAT[t], NADtMAT, C10KetoacylCoAMAT[t],
    C16KetoacylCoAMAT[t], C14KetoacylCoAMAT[t], C12KetoacylCoAMAT[t],
    C8KetoacylCoAMAT[t], C6KetoacylCoAMAT[t], C4AcetoacylCoAMAT, NADHMAT],
  vmschadC8 → MSCHAD[sfmschadC8, Vmschad, KmmschadC8HydroxyacylCoAMAT,
    KmmschadC16HydroxyacylCoAMAT, KmmschadC14HydroxyacylCoAMAT,
    KmmschadC12HydroxyacylCoAMAT, KmmschadC10HydroxyacylCoAMAT,
    KmmschadC6HydroxyacylCoAMAT, KmmschadC4HydroxyacylCoAMAT,
    KmmschadNADMAT, KmmschadC8KetoacylCoAMAT, KmmschadC16KetoacylCoAMAT,
    KmmschadC14KetoacylCoAMAT, KmmschadC12KetoacylCoAMAT, KmmschadC10KetoacylCoAMAT,
    KmmschadC6KetoacylCoAMAT, KmmschadC4AcetoacylCoAMAT, KmmschadNADHMAT,
    Keqmschad, C8HydroxyacylCoAMAT[t], C16HydroxyacylCoAMAT[t],
    C14HydroxyacylCoAMAT[t], C12HydroxyacylCoAMAT[t], C10HydroxyacylCoAMAT[t],
    C6HydroxyacylCoAMAT[t], C4HydroxyacylCoAMAT[t], NADtMAT, C8KetoacylCoAMAT[t],
    C16KetoacylCoAMAT[t], C14KetoacylCoAMAT[t], C12KetoacylCoAMAT[t],
    C10KetoacylCoAMAT[t], C6KetoacylCoAMAT[t], C4AcetoacylCoAMAT, NADHMAT],
  vmschadC6 → MSCHAD[sfmschadC6, Vmschad, KmmschadC6HydroxyacylCoAMAT,
    KmmschadC16HydroxyacylCoAMAT, KmmschadC14HydroxyacylCoAMAT,
    KmmschadC12HydroxyacylCoAMAT, KmmschadC10HydroxyacylCoAMAT,
    KmmschadC8HydroxyacylCoAMAT, KmmschadC4HydroxyacylCoAMAT,
    KmmschadNADMAT, KmmschadC6KetoacylCoAMAT, KmmschadC16KetoacylCoAMAT,
    KmmschadC14KetoacylCoAMAT, KmmschadC12KetoacylCoAMAT, KmmschadC10KetoacylCoAMAT,
    KmmschadC8KetoacylCoAMAT, KmmschadC4AcetoacylCoAMAT, KmmschadNADHMAT,
    Keqmschad, C6HydroxyacylCoAMAT[t], C16HydroxyacylCoAMAT[t],
    C14HydroxyacylCoAMAT[t], C12HydroxyacylCoAMAT[t], C10HydroxyacylCoAMAT[t],
    C8HydroxyacylCoAMAT[t], C4HydroxyacylCoAMAT[t], NADtMAT, C6KetoacylCoAMAT[t],
    C16KetoacylCoAMAT[t], C14KetoacylCoAMAT[t], C12KetoacylCoAMAT[t],
    C10KetoacylCoAMAT[t], C8KetoacylCoAMAT[t], C4AcetoacylCoAMAT, NADHMAT],
  vmschadC4 → MSCHAD[sfmschadC4, Vmschad, KmmschadC4HydroxyacylCoAMAT,
    KmmschadC16HydroxyacylCoAMAT, KmmschadC14HydroxyacylCoAMAT,
    KmmschadC12HydroxyacylCoAMAT, KmmschadC10HydroxyacylCoAMAT,
```

```
    KmmschadC8HydroxyacylCoAMAT, KmmschadC6HydroxyacylCoAMAT,
    KmmschadNADMAT, KmmschadC4AcetoacylCoAMAT, KmmschadC16KetoacylCoAMAT,
    KmmschadC14KetoacylCoAMAT, KmmschadC12KetoacylCoAMAT, KmmschadC10KetoacylCoAMAT,
    KmmschadC8KetoacylCoAMAT, KmmschadC6KetoacylCoAMAT, KmmschadNADHMAT,
    Keqmschad, C4HydroxyacylCoAMAT[t], C16HydroxyacylCoAMAT[t],
    C14HydroxyacylCoAMAT[t], C12HydroxyacylCoAMAT[t], C10HydroxyacylCoAMAT[t],
    C8HydroxyacylCoAMAT[t], C6HydroxyacylCoAMAT[t], NADtMAT, C4AcetoacylCoAMAT,
    C16KetoacylCoAMAT[t], C14KetoacylCoAMAT[t], C12KetoacylCoAMAT[t],
    C10KetoacylCoAMAT[t], C8KetoacylCoAMAT[t], C6KetoacylCoAMAT[t], NADHMAT],
  vmckatC16 → MCKATA[sfmckatC16, Vmckat, KmmckatC16KetoacylCoAMAT,
    KmmckatC14KetoacylCoAMAT, KmmckatC12KetoacylCoAMAT, KmmckatC10KetoacylCoAMAT,
    KmmckatC8KetoacylCoAMAT, KmmckatC6KetoacylCoAMAT, KmmckatC4AcetoacylCoAMAT,
    KmmckatCoAMAT, KmmckatC14AcylCoAMAT, KmmckatC16AcylCoAMAT, KmmckatC12AcylCoAMAT,
    KmmckatC10AcylCoAMAT, KmmckatC8AcylCoAMAT, KmmckatC6AcylCoAMAT,
    KmmckatC4AcylCoAMAT, KmmckatAcetylCoAMAT, Keqmckat, C16KetoacylCoAMAT[t],
    C14KetoacylCoAMAT[t], C12KetoacylCoAMAT[t], C10KetoacylCoAMAT[t],
    C8KetoacylCoAMAT[t], C6KetoacylCoAMAT[t], C4AcetoacylCoAMAT, CoAMAT,
    C14AcylCoAMAT[t], C16AcylCoAMAT[t], C12AcylCoAMAT[t], C10AcylCoAMAT[t],
    C8AcylCoAMAT[t], C6AcylCoAMAT[t], C4AcylCoAMAT[t], AcetylCoAMAT],
  vmckatC14 → MCKATA[sfmckatC14, Vmckat, KmmckatC14KetoacylCoAMAT,
    KmmckatC16KetoacylCoAMAT, KmmckatC12KetoacylCoAMAT, KmmckatC10KetoacylCoAMAT,
    KmmckatC8KetoacylCoAMAT, KmmckatC6KetoacylCoAMAT, KmmckatC4AcetoacylCoAMAT,
    KmmckatCoAMAT, KmmckatC12AcylCoAMAT, KmmckatC16AcylCoAMAT, KmmckatC14AcylCoAMAT,
    KmmckatC10AcylCoAMAT, KmmckatC8AcylCoAMAT, KmmckatC6AcylCoAMAT,
    KmmckatC4AcylCoAMAT, KmmckatAcetylCoAMAT, Keqmckat, C14KetoacylCoAMAT[t],
    C16KetoacylCoAMAT[t], C12KetoacylCoAMAT[t], C10KetoacylCoAMAT[t],
    C8KetoacylCoAMAT[t], C6KetoacylCoAMAT[t], C4AcetoacylCoAMAT, CoAMAT,
    C12AcylCoAMAT[t], C16AcylCoAMAT[t], C14AcylCoAMAT[t], C10AcylCoAMAT[t],
    C8AcylCoAMAT[t], C6AcylCoAMAT[t], C4AcylCoAMAT[t], AcetylCoAMAT],
  vmckatC12 → MCKATA[sfmckatC12, Vmckat, KmmckatC12KetoacylCoAMAT,
    KmmckatC16KetoacylCoAMAT, KmmckatC14KetoacylCoAMAT, KmmckatC10KetoacylCoAMAT,
    KmmckatC8KetoacylCoAMAT, KmmckatC6KetoacylCoAMAT, KmmckatC4AcetoacylCoAMAT,
    KmmckatCoAMAT, KmmckatC10AcylCoAMAT, KmmckatC16AcylCoAMAT, KmmckatC14AcylCoAMAT,
    KmmckatC12AcylCoAMAT, KmmckatC8AcylCoAMAT, KmmckatC6AcylCoAMAT,
    KmmckatC4AcylCoAMAT, KmmckatAcetylCoAMAT, Keqmckat, C12KetoacylCoAMAT[t],
    C16KetoacylCoAMAT[t], C14KetoacylCoAMAT[t], C10KetoacylCoAMAT[t],
    C8KetoacylCoAMAT[t], C6KetoacylCoAMAT[t], C4AcetoacylCoAMAT, CoAMAT,
    C10AcylCoAMAT[t], C16AcylCoAMAT[t], C14AcylCoAMAT[t], C12AcylCoAMAT[t],
    C8AcylCoAMAT[t], C6AcylCoAMAT[t], C4AcylCoAMAT[t], AcetylCoAMAT],
  vmckatC10 → MCKATA[sfmckatC10, Vmckat, KmmckatC10KetoacylCoAMAT,
    KmmckatC16KetoacylCoAMAT, KmmckatC14KetoacylCoAMAT, KmmckatC12KetoacylCoAMAT,
    KmmckatC8KetoacylCoAMAT, KmmckatC6KetoacylCoAMAT, KmmckatC4AcetoacylCoAMAT,
    KmmckatCoAMAT, KmmckatC8AcylCoAMAT, KmmckatC16AcylCoAMAT, KmmckatC14AcylCoAMAT,
    KmmckatC12AcylCoAMAT, KmmckatC10AcylCoAMAT, KmmckatC6AcylCoAMAT,
    KmmckatC4AcylCoAMAT, KmmckatAcetylCoAMAT, Keqmckat, C10KetoacylCoAMAT[t],
    C16KetoacylCoAMAT[t], C14KetoacylCoAMAT[t], C12KetoacylCoAMAT[t],
    C8KetoacylCoAMAT[t], C6KetoacylCoAMAT[t], C4AcetoacylCoAMAT, CoAMAT,
    C8AcylCoAMAT[t], C16AcylCoAMAT[t], C14AcylCoAMAT[t], C12AcylCoAMAT[t],
    C10AcylCoAMAT[t], C6AcylCoAMAT[t], C4AcylCoAMAT[t], AcetylCoAMAT],
  vmckatC8 → MCKATA[sfmckatC8, Vmckat, KmmckatC8KetoacylCoAMAT,
    KmmckatC16KetoacylCoAMAT, KmmckatC14KetoacylCoAMAT, KmmckatC12KetoacylCoAMAT,
    KmmckatC10KetoacylCoAMAT, KmmckatC6KetoacylCoAMAT, KmmckatC4AcetoacylCoAMAT,
    KmmckatCoAMAT, KmmckatC6AcylCoAMAT, KmmckatC16AcylCoAMAT, KmmckatC14AcylCoAMAT,
    KmmckatC12AcylCoAMAT, KmmckatC10AcylCoAMAT, KmmckatC8AcylCoAMAT,
    KmmckatC4AcylCoAMAT, KmmckatAcetylCoAMAT, Keqmckat, C8KetoacylCoAMAT[t],
```

```
    C16KetoacylCoAMAT[t], C14KetoacylCoAMAT[t], C12KetoacylCoAMAT[t],
    C10KetoacylCoAMAT[t], C6KetoacylCoAMAT[t], C4AcetoacylCoAMAT, CoAMAT,
    C6AcylCoAMAT[t], C16AcylCoAMAT[t], C14AcylCoAMAT[t], C12AcylCoAMAT[t],
    C10AcylCoAMAT[t], C8AcylCoAMAT[t], C4AcylCoAMAT[t], AcetylCoAMAT],
 vmckatC6 → MCKATA[sfmckatC6, Vmckat, KmmckatC6KetoacylCoAMAT,
    KmmckatC16KetoacylCoAMAT, KmmckatC14KetoacylCoAMAT,
    KmmckatC12KetoacylCoAMAT, KmmckatC10KetoacylCoAMAT, KmmckatC8KetoacylCoAMAT,
    KmmckatC4AcetoacylCoAMAT, KmmckatCoAMAT, KmmckatC4AcylCoAMAT,
    KmmckatC16AcylCoAMAT, KmmckatC14AcylCoAMAT, KmmckatC12AcylCoAMAT,
    KmmckatC10AcylCoAMAT, KmmckatC8AcylCoAMAT, KmmckatC6AcylCoAMAT,
    KmmckatAcetylCoAMAT, Keqmckat, C6KetoacylCoAMAT[t], C16KetoacylCoAMAT[t],
    C14KetoacylCoAMAT[t], C12KetoacylCoAMAT[t], C10KetoacylCoAMAT[t],
    C8KetoacylCoAMAT[t], C4AcetoacylCoAMAT, CoAMAT, C4AcylCoAMAT[t],
    C16AcylCoAMAT[t], C14AcylCoAMAT[t], C12AcylCoAMAT[t], C10AcylCoAMAT[t],
    C8AcylCoAMAT[t], C6AcylCoAMAT[t], AcetylCoAMAT], (*C6AcylCoAMAT[t]*)
 vmckatC4 → MCKATB[sfmckatC4, Vmckat, KmmckatC4AcetoacylCoAMAT,
    KmmckatC16KetoacylCoAMAT, KmmckatC14KetoacylCoAMAT,
    KmmckatC12KetoacylCoAMAT, KmmckatC10KetoacylCoAMAT, KmmckatC8KetoacylCoAMAT,
    KmmckatC6KetoacylCoAMAT, KmmckatCoAMAT, KmmckatC4AcylCoAMAT,
    KmmckatC16AcylCoAMAT, KmmckatC14AcylCoAMAT, KmmckatC12AcylCoAMAT,
    KmmckatC10AcylCoAMAT, KmmckatC8AcylCoAMAT, KmmckatC6AcylCoAMAT,
    KmmckatAcetylCoAMAT, Keqmckat, C4AcetoacylCoAMAT, C16KetoacylCoAMAT[t],
    C14KetoacylCoAMAT[t], C12KetoacylCoAMAT[t], C10KetoacylCoAMAT[t],
    C8KetoacylCoAMAT[t], C6KetoacylCoAMAT[t], CoAMAT, C4AcylCoAMAT[t],
    C16AcylCoAMAT[t], C14AcylCoAMAT[t], C12AcylCoAMAT[t], C10AcylCoAMAT[t],
    C8AcylCoAMAT[t], C6AcylCoAMAT[t], AcetylCoAMAT], (*C4AcylCoAMAT[t]*)
 vmtpC16 → MTP[sfmtpC16, Vmtp, KmmtpC16EnoylCoAMAT, KmmtpC14EnoylCoAMAT,
    KmmtpC12EnoylCoAMAT, KmmtpC10EnoylCoAMAT, KmmtpC8EnoylCoAMAT,
    KmmtpNADMAT, KmmtpCoAMAT, KmmtpC14AcylCoAMAT, KmmtpC16AcylCoAMAT,
    KmmtpC12AcylCoAMAT, KmmtpC10AcylCoAMAT, KmmtpC8AcylCoAMAT,
    KmmtpC6AcylCoAMAT, KmmtpNADHMAT, KmmtpAcetylCoAMAT, KicrotC4AcetoacylCoA,
    Keqmtp, C16EnoylCoAMAT[t], C14EnoylCoAMAT[t], C12EnoylCoAMAT[t],
    C10EnoylCoAMAT[t], C8EnoylCoAMAT[t], NADtMAT, CoAMAT, C14AcylCoAMAT[t],
    C16AcylCoAMAT[t], C12AcylCoAMAT[t], C10AcylCoAMAT[t], C8AcylCoAMAT[t],
    C6AcylCoAMAT[t], NADHMAT, AcetylCoAMAT, C4AcetoacylCoAMAT],
 vmtpC14 → MTP[sfmtpC14, Vmtp, KmmtpC14EnoylCoAMAT, KmmtpC16EnoylCoAMAT,
    KmmtpC12EnoylCoAMAT, KmmtpC10EnoylCoAMAT, KmmtpC8EnoylCoAMAT,
    KmmtpNADMAT, KmmtpCoAMAT, KmmtpC12AcylCoAMAT, KmmtpC16AcylCoAMAT,
    KmmtpC14AcylCoAMAT, KmmtpC10AcylCoAMAT, KmmtpC8AcylCoAMAT,
    KmmtpC6AcylCoAMAT, KmmtpNADHMAT, KmmtpAcetylCoAMAT, KicrotC4AcetoacylCoA,
    Keqmtp, C14EnoylCoAMAT[t], C16EnoylCoAMAT[t], C12EnoylCoAMAT[t],
    C10EnoylCoAMAT[t], C8EnoylCoAMAT[t], NADtMAT, CoAMAT, C12AcylCoAMAT[t],
    C16AcylCoAMAT[t], C14AcylCoAMAT[t], C10AcylCoAMAT[t], C8AcylCoAMAT[t],
    C6AcylCoAMAT[t], NADHMAT, AcetylCoAMAT, C4AcetoacylCoAMAT],
 vmtpC12 → MTP[sfmtpC12, Vmtp, KmmtpC12EnoylCoAMAT, KmmtpC16EnoylCoAMAT,
    KmmtpC14EnoylCoAMAT, KmmtpC10EnoylCoAMAT, KmmtpC8EnoylCoAMAT,
    KmmtpNADMAT, KmmtpCoAMAT, KmmtpC10AcylCoAMAT, KmmtpC16AcylCoAMAT,
    KmmtpC14AcylCoAMAT, KmmtpC12AcylCoAMAT, KmmtpC8AcylCoAMAT,
    KmmtpC6AcylCoAMAT, KmmtpNADHMAT, KmmtpAcetylCoAMAT, KicrotC4AcetoacylCoA,
    Keqmtp, C12EnoylCoAMAT[t], C16EnoylCoAMAT[t], C14EnoylCoAMAT[t],
    C10EnoylCoAMAT[t], C8EnoylCoAMAT[t], NADtMAT, CoAMAT, C10AcylCoAMAT[t],
    C16AcylCoAMAT[t], C14AcylCoAMAT[t], C12AcylCoAMAT[t], C8AcylCoAMAT[t],
    C6AcylCoAMAT[t], NADHMAT, AcetylCoAMAT, C4AcetoacylCoAMAT],
 vmtpC10 → MTP[sfmtpC10, Vmtp, KmmtpC10EnoylCoAMAT, KmmtpC16EnoylCoAMAT,
    KmmtpC14EnoylCoAMAT, KmmtpC12EnoylCoAMAT, KmmtpC8EnoylCoAMAT,
```

```
      KmmtpNADMAT, KmmtpCoAMAT, KmmtpC8AcylCoAMAT, KmmtpC16AcylCoAMAT,
      KmmtpC14AcylCoAMAT, KmmtpC12AcylCoAMAT, KmmtpC10AcylCoAMAT,
      KmmtpC6AcylCoAMAT, KmmtpNADHMAT, KmmtpAcetylCoAMAT, KicrotC4AcetoacylCoA,
      Keqmtp, C10EnoylCoAMAT[t], C16EnoylCoAMAT[t], C14EnoylCoAMAT[t],
      C12EnoylCoAMAT[t], C8EnoylCoAMAT[t], NADtMAT, CoAMAT, C8AcylCoAMAT[t],
      C16AcylCoAMAT[t], C14AcylCoAMAT[t], C12AcylCoAMAT[t], C10AcylCoAMAT[t],
      C6AcylCoAMAT[t], NADHMAT, AcetylCoAMAT, C4AcetoacylCoAMAT],
    vmtpC8 → MTP[sfmtpC8, Vmtp, KmmtpC8EnoylCoAMAT, KmmtpC16EnoylCoAMAT,
      KmmtpC14EnoylCoAMAT, KmmtpC12EnoylCoAMAT, KmmtpC10EnoylCoAMAT,
      KmmtpNADMAT, KmmtpCoAMAT, KmmtpC6AcylCoAMAT, KmmtpC16AcylCoAMAT,
      KmmtpC14AcylCoAMAT, KmmtpC12AcylCoAMAT, KmmtpC10AcylCoAMAT,
      KmmtpC8AcylCoAMAT, KmmtpNADHMAT, KmmtpAcetylCoAMAT, KicrotC4AcetoacylCoA,
      Keqmtp, C8EnoylCoAMAT[t], C16EnoylCoAMAT[t], C14EnoylCoAMAT[t],
      C12EnoylCoAMAT[t], C10EnoylCoAMAT[t], NADtMAT, CoAMAT, C6AcylCoAMAT[t],
      C16AcylCoAMAT[t], C14AcylCoAMAT[t], C12AcylCoAMAT[t], C10AcylCoAMAT[t],
      C8AcylCoAMAT[t], NADHMAT, AcetylCoAMAT, C4AcetoacylCoAMAT],
    vacesink → RES[Ksacesink, AcetylCoAMAT, K1acesink],
    vfadhsink → RES[Ksfadhsink, FADHMAT, K1fadhsink],
    vnadhsink → RES[Ksnadhsink, NADHMAT, K1nadhsink]};

CoAMATX =
   {CoAMAT → CoAMATt - C16AcylCoAMAT[t] - C16EnoylCoAMAT[t] - C16HydroxyacylCoAMAT[t] -
      C16KetoacylCoAMAT[t] - C14AcylCoAMAT[t] - C14EnoylCoAMAT[t] -
      C14HydroxyacylCoAMAT[t] - C14KetoacylCoAMAT[t] - C12AcylCoAMAT[t] -
      C12EnoylCoAMAT[t] - C12HydroxyacylCoAMAT[t] - C12KetoacylCoAMAT[t] -
      C10AcylCoAMAT[t] - C10EnoylCoAMAT[t] - C10HydroxyacylCoAMAT[t] -
      C10KetoacylCoAMAT[t] - C8AcylCoAMAT[t] - C8EnoylCoAMAT[t] -
      C8HydroxyacylCoAMAT[t] - C8KetoacylCoAMAT[t] - C6AcylCoAMAT[t] - C6EnoylCoAMAT[t] -
      C6HydroxyacylCoAMAT[t] - C6KetoacylCoAMAT[t] - C4AcylCoAMAT[t] -
      C4EnoylCoAMAT[t] - C4HydroxyacylCoAMAT[t] - C4AcetoacylCoAMAT - AcetylCoAMAT};

Parm = {
    sfcpt1C16 → 1, Vcpt1 → 0.012, Kmcpt1C16AcylCoACYT → 13.8,
    Kmcpt1CarCYT → 250, Kmcpt1C16AcylCarCYT → 136, Kmcpt1CoACYT → 40.7,
    Kicpt1MalCoACYT → 9.1, Keqcpt1 → 0.45,  ncpt1 → 2.4799,
    Vfcact → 0.42, Vrcact → 0.42, KmcactC16AcylCarCYT → 15,
    KmcactC14AcylCarCYT → 15, KmcactC12AcylCarCYT → 15, KmcactC10AcylCarCYT → 15,
    KmcactC8AcylCarCYT → 15, KmcactC6AcylCarCYT → 15, KmcactC4AcylCarCYT → 15,
    KmcactCarMAT → 130, KmcactC16AcylCarMAT → 15, KmcactC14AcylCarMAT → 15,
    KmcactC12AcylCarMAT → 15, KmcactC10AcylCarMAT → 15, KmcactC8AcylCarMAT → 15,
    KmcactC6AcylCarMAT → 15, KmcactC4AcylCarMAT → 15, KmcactCarCYT → 130,
    KicactC16AcylCarCYT → 56, KicactC14AcylCarCYT → 56, KicactC12AcylCarCYT → 56,
    KicactC10AcylCarCYT → 56, KicactC8AcylCarCYT → 56, KicactC6AcylCarCYT → 56,
    KicactC4AcylCarCYT → 56, KicactCarCYT → 200, Keqcact → 1,
    sfcpt2C16 → 0.85, sfcpt2C14 → 1, sfcpt2C12 → 0.95, sfcpt2C10 → 0.95,
    sfcpt2C8 → 0.35, sfcpt2C6 → 0.15, sfcpt2C4 → 0.01, Vcpt2 → 0.391,
    Kmcpt2C16AcylCarMAT → 51, Kmcpt2C14AcylCarMAT → 51, Kmcpt2C12AcylCarMAT → 51,
    Kmcpt2C10AcylCarMAT → 51, Kmcpt2C8AcylCarMAT → 51, Kmcpt2C6AcylCarMAT → 51,
    Kmcpt2C4AcylCarMAT → 51, Kmcpt2CoAMAT → 30, Kmcpt2C16AcylCoAMAT → 38,
    Kmcpt2C14AcylCoAMAT → 38, Kmcpt2C12AcylCoAMAT → 38,
    Kmcpt2C10AcylCoAMAT → 38, Kmcpt2C8AcylCoAMAT → 38, Kmcpt2C6AcylCoAMAT → 1000,
    Kmcpt2C4AcylCoAMAT → 1000000, Kmcpt2CarMAT → 350, Keqcpt2 → 2.22,
    sfvlcadC16 → 1, sfvlcadC14 → 0.42, sfvlcadC12 → 0.11, Vvlcad → 0.008,
    KmvlcadC16AcylCoAMAT → 6.5, KmvlcadC14AcylCoAMAT → 4, KmvlcadC12AcylCoAMAT → 2.7,
    KmvlcadFAD → 0.12, KmvlcadC16EnoylCoAMAT → 1.08, KmvlcadC14EnoylCoAMAT → 1.08,
```

```
KmvlcadC12EnoylCoAMAT → 1.08, KmvlcadFADH → 24.2, Keqvlcad → 6,
sflcadC16 → 0.9, sflcadC14 → 1, sflcadC12 → 0.9, sflcadC10 → 0.75, sflcadC8 → 0.4,
Vlcad → 0.01, KmlcadC16AcylCoAMAT → 2.5, KmlcadC14AcylCoAMAT → 7.4,
KmlcadC12AcylCoAMAT → 9, KmlcadC10AcylCoAMAT → 24.3, KmlcadC8AcylCoAMAT → 123,
KmlcadFAD → 0.12, KmlcadC16EnoylCoAMAT → 1.08, KmlcadC14EnoylCoAMAT → 1.08,
KmlcadC12EnoylCoAMAT → 1.08, KmlcadC10EnoylCoAMAT → 1.08,
KmlcadC8EnoylCoAMAT → 1.08, KmlcadFADH → 24.2, Keqlcad → 6,
sfmcadC12 → 0.38, sfmcadC10 → 0.8, sfmcadC8 → 0.87, sfmcadC6 → 1, sfmcadC4 → 0.12,
Vmcad → 0.081, KmmcadC12AcylCoAMAT → 5.7, KmmcadC10AcylCoAMAT → 5.4,
KmmcadC8AcylCoAMAT → 4, KmmcadC6AcylCoAMAT → 9.4, KmmcadC4AcylCoAMAT → 135,
KmmcadFAD → 0.12, KmmcadC12EnoylCoAMAT → 1.08, KmmcadC10EnoylCoAMAT → 1.08,
KmmcadC8EnoylCoAMAT → 1.08, KmmcadC6EnoylCoAMAT → 1.08,
KmmcadC4EnoylCoAMAT → 1.08, KmmcadFADH → 24.2, Keqmcad → 6,
sfscadC6 → 0.3, sfscadC4 → 1, Vscad → 0.081, KmscadC6AcylCoAMAT → 285,
KmscadC4AcylCoAMAT → 10.7, KmscadFAD → 0.12, KmscadC6EnoylCoAMAT → 1.08,
KmscadC4EnoylCoAMAT → 1.08, KmscadFADH → 24.2, Keqscad -> 6,
sfcrotC16 → 0.13, sfcrotC14 → 0.2, sfcrotC12 → 0.25, sfcrotC10 → 0.33, sfcrotC8 → 0.58,
sfcrotC6 → 0.83, sfcrotC4 → 1, Vcrot → 3.6, KmcrotC16EnoylCoAMAT → 150,
KmcrotC14EnoylCoAMAT → 100, KmcrotC12EnoylCoAMAT → 25, KmcrotC10EnoylCoAMAT → 25,
KmcrotC8EnoylCoAMAT → 25, KmcrotC6EnoylCoAMAT → 25, KmcrotC4EnoylCoAMAT → 40,
KmcrotC16HydroxyacylCoAMAT → 45, KmcrotC14HydroxyacylCoAMAT → 45,
KmcrotC12HydroxyacylCoAMAT → 45, KmcrotC10HydroxyacylCoAMAT → 45,
KmcrotC8HydroxyacylCoAMAT → 45, KmcrotC6HydroxyacylCoAMAT → 45,
KmcrotC4HydroxyacylCoAMAT → 45, KicrotC4AcetoacylCoA → 1.6, Keqcrot → 3.13,
sfmschadC16 → 0.6, sfmschadC14 → 0.5, sfmschadC12 → 0.43, sfmschadC10 → 0.64,
sfmschadC8 → 0.89, sfmschadC6 → 1, sfmschadC4 → 0.67, Vmschad → 1,
KmmschadC16HydroxyacylCoAMAT → 1.5, KmmschadC14HydroxyacylCoAMAT → 1.8,
KmmschadC12HydroxyacylCoAMAT → 3.7, KmmschadC10HydroxyacylCoAMAT → 8.8,
KmmschadC8HydroxyacylCoAMAT → 16.3, KmmschadC6HydroxyacylCoAMAT → 28.6,
KmmschadC4HydroxyacylCoAMAT → 69.9, KmmschadNADMAT → 58.5,
KmmschadC16KetoacylCoAMAT → 1.4, KmmschadC14KetoacylCoAMAT → 1.4,
KmmschadC12KetoacylCoAMAT → 1.6, KmmschadC10KetoacylCoAMAT → 2.3,
KmmschadC8KetoacylCoAMAT → 4.1, KmmschadC6KetoacylCoAMAT → 5.8,
KmmschadC4AcetoacylCoAMAT → 16.9, KmmschadNADHMAT → 5.4, Keqmschad → 2.17 * 10^-4,
sfmckatC16 → 0, sfmckatC14 → 0.2, sfmckatC12 → 0.38, sfmckatC10 → 0.65,
sfmckatC8 → 0.81, sfmckatC6 → 1, sfmckatC4 → 0.49, Vmckat → 0.377,
KmmckatC16KetoacylCoAMAT → 1.1, KmmckatC14KetoacylCoAMAT → 1.2,
KmmckatC12KetoacylCoAMAT → 1.3, KmmckatC10KetoacylCoAMAT → 2.1,
KmmckatC8KetoacylCoAMAT → 3.2, KmmckatC6KetoacylCoAMAT → 6.7,
KmmckatC4AcetoacylCoAMAT → 12.4, KmmckatCoAMAT → 26.6,
KmmckatC14AcylCoAMAT → 13.83, KmmckatC16AcylCoAMAT → 13.83,
KmmckatC12AcylCoAMAT → 13.83, KmmckatC10AcylCoAMAT → 13.83,
KmmckatC8AcylCoAMAT → 13.83, KmmckatC6AcylCoAMAT → 13.83,
KmmckatC4AcylCoAMAT → 13.83, KmmckatAcetylCoAMAT → 30, Keqmckat → 1051,
sfmtpC16 → 1, sfmtpC14 → 0.9, sfmtpC12 → 0.81, sfmtpC10 → 0.73, sfmtpC8 → 0.34,
Vmtp → 2.84, KmmtpC16EnoylCoAMAT → 25, KmmtpC14EnoylCoAMAT → 25,
KmmtpC12EnoylCoAMAT → 25, KmmtpC10EnoylCoAMAT → 25, KmmtpC8EnoylCoAMAT → 25,
KmmtpNADMAT → 60, KmmtpCoAMAT → 30, KmmtpC14AcylCoAMAT → 13.83,
KmmtpC16AcylCoAMAT → 13.83, KmmtpC12AcylCoAMAT → 13.83,
KmmtpC10AcylCoAMAT → 13.83, KmmtpC8AcylCoAMAT → 13.83, KmmtpC6AcylCoAMAT → 13.83,
KmmtpNADHMAT → 50, KmmtpAcetylCoAMAT → 30, Keqmtp → 0.71,
Ksacesink → 6 000 000, K1acesink → 70, Ksfadhsink → 6 000 000,
K1fadhsink → 0.46, Ksnadhsink → 6 000 000, K1nadhsink → 12,
C16AcylCoACYT → 25, CarCYT → 200, CoACYT → 140, MalCoACYT → 0,
CarMAT -> 950, FADtMAT → 0.77, NADtMAT → 250, CoAMATt → 5000,
```

```
   VCYT → 2.2 * 10^-6, VMAT → 1.8 * 10^-6, AcetylCoAMAT → 70,
   FADHMAT → 0.46, NADHMAT → 12, C4AcetoacylCoAMAT → 9.50};

InitialConditions = {
   C16AcylCarCYT[0] == 0, C16AcylCarMAT[0] == 0, C16AcylCoAMAT[0] == 0,
   C16EnoylCoAMAT[0] == 0, C16HydroxyacylCoAMAT[0] == 0, C16KetoacylCoAMAT[0] == 0,
   C14AcylCarCYT[0] == 0, C14AcylCarMAT[0] == 0, C14AcylCoAMAT[0] == 0,
   C14EnoylCoAMAT[0] == 0, C14HydroxyacylCoAMAT[0] == 0, C14KetoacylCoAMAT[0] == 0,
   C12AcylCarCYT[0] == 0, C12AcylCarMAT[0] == 0, C12AcylCoAMAT[0] == 0,
   C12EnoylCoAMAT[0] == 0, C12HydroxyacylCoAMAT[0] == 0, C12KetoacylCoAMAT[0] == 0,
   C10AcylCarCYT[0] == 0, C10AcylCarMAT[0] == 0, C10AcylCoAMAT[0] == 0,
   C10EnoylCoAMAT[0] == 0, C10HydroxyacylCoAMAT[0] == 0, C10KetoacylCoAMAT[0] == 0,
   C8AcylCarCYT[0] == 0, C8AcylCarMAT[0] == 0, C8AcylCoAMAT[0] == 0,
   C8EnoylCoAMAT[0] == 0, C8HydroxyacylCoAMAT[0] == 0, C8KetoacylCoAMAT[0] == 0,
   C6AcylCarCYT[0] == 0, C6AcylCarMAT[0] == 0, C6AcylCoAMAT[0] == 0,
   C6EnoylCoAMAT[0] == 0, C6HydroxyacylCoAMAT[0] == 0, C6KetoacylCoAMAT[0] == 0,
   C4AcylCarCYT[0] == 0, C4AcylCarMAT[0] == 0, C4AcylCoAMAT[0] == 0, C4EnoylCoAMAT[0] == 0,
   C4HydroxyacylCoAMAT[0] == 0 }; (*,C4AcetoacylCoAMAT[0]==0};*)

Vars = {
   C16AcylCarCYT, C16AcylCarMAT, C16AcylCoAMAT,
   C16EnoylCoAMAT, C16HydroxyacylCoAMAT, C16KetoacylCoAMAT,
   C14AcylCarCYT, C14AcylCarMAT, C14AcylCoAMAT, C14EnoylCoAMAT,
   C14HydroxyacylCoAMAT, C14KetoacylCoAMAT,
   C12AcylCarCYT, C12AcylCarMAT, C12AcylCoAMAT, C12EnoylCoAMAT,
   C12HydroxyacylCoAMAT, C12KetoacylCoAMAT,
   C10AcylCarCYT, C10AcylCarMAT, C10AcylCoAMAT, C10EnoylCoAMAT,
   C10HydroxyacylCoAMAT, C10KetoacylCoAMAT,
   C8AcylCarCYT, C8AcylCarMAT, C8AcylCoAMAT, C8EnoylCoAMAT,
   C8HydroxyacylCoAMAT, C8KetoacylCoAMAT,
   C6AcylCarCYT, C6AcylCarMAT, C6AcylCoAMAT, C6EnoylCoAMAT,
   C6HydroxyacylCoAMAT, C6KetoacylCoAMAT,
   C4AcylCarCYT, C4AcylCarMAT, C4AcylCoAMAT, C4EnoylCoAMAT, C4HydroxyacylCoAMAT};
```

```
In[]:= TableForm[Odes];
TableForm[RateEqs];
TableForm[Odes /. RateEqs /. CoAMATX /. Parm ];
TableForm[RateEqs /. Parm ];
TableForm[InitialConditions];
```

```
In[]:=
tsol = NDSolve[Join[Odes /. RateEqs /. CoAMATX /. Parm , InitialConditions],
   Vars, {t, 0, 1000000000}];
```

In[ ]:=
```
Table[{Vars[[i]][t], (Vars[[i]][900000000] /. tsol)[[1]]}, {i, 1, Length[Vars]}]
```

Out[ ]=
```
{{C16AcylCarCYT[t], 0.756046}, {C16AcylCarMAT[t], 3.14533},
 {C16AcylCoAMAT[t], 3.33431}, {C16EnoylCoAMAT[t], 0.421291},
 {C16HydroxyacylCoAMAT[t], 1.31864}, {C16KetoacylCoAMAT[t], 0.00567521},
 {C14AcylCarCYT[t], 0.617941}, {C14AcylCarMAT[t], 2.93522},
 {C14AcylCoAMAT[t], 7.09155}, {C14EnoylCoAMAT[t], 0.481954},
 {C14HydroxyacylCoAMAT[t], 1.29848}, {C14KetoacylCoAMAT[t], 0.00558118},
 {C12AcylCarCYT[t], 0.977987}, {C12AcylCarMAT[t], 4.64544},
 {C12AcylCoAMAT[t], 11.2235}, {C12EnoylCoAMAT[t], 0.640721},
 {C12HydroxyacylCoAMAT[t], 1.91516}, {C12KetoacylCoAMAT[t], 0.00820524},
 {C10AcylCarCYT[t], 3.04502}, {C10AcylCarMAT[t], 14.4639},
 {C10AcylCoAMAT[t], 34.945}, {C10EnoylCoAMAT[t], 0.818145},
 {C10HydroxyacylCoAMAT[t], 2.47985}, {C10KetoacylCoAMAT[t], 0.0106023},
 {C8AcylCarCYT[t], 5.34126}, {C8AcylCarMAT[t], 25.371}, {C8AcylCoAMAT[t], 61.2968},
 {C8EnoylCoAMAT[t], 1.88227}, {C8HydroxyacylCoAMAT[t], 5.80935},
 {C8KetoacylCoAMAT[t], 0.0248349}, {C6AcylCarCYT[t], 13.9584},
 {C6AcylCarMAT[t], 66.3026}, {C6AcylCoAMAT[t], 160.188}, {C6EnoylCoAMAT[t], 81.0406},
 {C6HydroxyacylCoAMAT[t], 251.211}, {C6KetoacylCoAMAT[t], 1.07002},
 {C4AcylCarCYT[t], 28.9327}, {C4AcylCarMAT[t], 137.43}, {C4AcylCoAMAT[t], 332.034},
 {C4EnoylCoAMAT[t], 709.276}, {C4HydroxyacylCoAMAT[t], 2216.79}}
```

In[ ]:=

In[ ]:=
```
ParmScan[X_] := {
    sfcpt1C16 → 1, Vcpt1 → 0.012, Kmcpt1C16AcylCoACYT → 13.8,
    Kmcpt1CarCYT → 250, Kmcpt1C16AcylCarCYT → 136, Kmcpt1CoACYT → 40.7,
    Kicpt1MalCoACYT → 9.1, Keqcpt1 → 0.45, ncpt1 → 2.4799,
    Vfcact → 0.42, Vrcact → 0.42, KmcactC16AcylCarCYT → 15,
    KmcactC14AcylCarCYT → 15, KmcactC12AcylCarCYT → 15, KmcactC10AcylCarCYT → 15,
    KmcactC8AcylCarCYT → 15, KmcactC6AcylCarCYT → 15, KmcactC4AcylCarCYT → 15,
    KmcactCarMAT → 130, KmcactC16AcylCarMAT → 15, KmcactC14AcylCarMAT → 15,
    KmcactC12AcylCarMAT → 15, KmcactC10AcylCarMAT → 15, KmcactC8AcylCarMAT → 15,
    KmcactC6AcylCarMAT → 15, KmcactC4AcylCarMAT → 15, KmcactCarCYT → 130,
    KicactC16AcylCarCYT → 56, KicactC14AcylCarCYT → 56, KicactC12AcylCarCYT → 56,
    KicactC10AcylCarCYT → 56, KicactC8AcylCarCYT → 56, KicactC6AcylCarCYT → 56,
    KicactC4AcylCarCYT → 56, KicactCarCYT → 200, Keqcact → 1,
    sfcpt2C16 → 0.85, sfcpt2C14 → 1, sfcpt2C12 → 0.95, sfcpt2C10 → 0.95,
    sfcpt2C8 → 0.35, sfcpt2C6 → 0.15, sfcpt2C4 → 0.01, Vcpt2 → 0.391,
    Kmcpt2C16AcylCarMAT → 51, Kmcpt2C14AcylCarMAT → 51, Kmcpt2C12AcylCarMAT → 51,
    Kmcpt2C10AcylCarMAT → 51, Kmcpt2C8AcylCarMAT → 51, Kmcpt2C6AcylCarMAT → 51,
    Kmcpt2C4AcylCarMAT → 51, Kmcpt2CoAMAT → 30, Kmcpt2C16AcylCoAMAT → 38,
    Kmcpt2C14AcylCoAMAT → 38, Kmcpt2C12AcylCoAMAT → 38,
    Kmcpt2C10AcylCoAMAT → 38, Kmcpt2C8AcylCoAMAT → 38, Kmcpt2C6AcylCoAMAT → 1000,
    Kmcpt2C4AcylCoAMAT → 1000000, Kmcpt2CarMAT → 350, Keqcpt2 → 2.22,
    sfvlcadC16 → 1, sfvlcadC14 → 0.42, sfvlcadC12 → 0.11, Vvlcad → 0.008,
    KmvlcadC16AcylCoAMAT → 6.5, KmvlcadC14AcylCoAMAT → 4, KmvlcadC12AcylCoAMAT → 2.7,
    KmvlcadFAD → 0.12, KmvlcadC16EnoylCoAMAT → 1.08, KmvlcadC14EnoylCoAMAT → 1.08,
    KmvlcadC12EnoylCoAMAT → 1.08, KmvlcadFADH → 24.2, Keqvlcad → 6,
    sflcadC16 → 0.9, sflcadC14 → 1, sflcadC12 → 0.9, sflcadC10 → 0.75, sflcadC8 → 0.4,
    Vlcad → 0.01, KmlcadC16AcylCoAMAT → 2.5, KmlcadC14AcylCoAMAT → 7.4,
    KmlcadC12AcylCoAMAT → 9, KmlcadC10AcylCoAMAT → 24.3, KmlcadC8AcylCoAMAT → 123,
```

```
KmlcadFAD → 0.12, KmlcadC16EnoylCoAMAT → 1.08, KmlcadC14EnoylCoAMAT → 1.08,
KmlcadC12EnoylCoAMAT → 1.08, KmlcadC10EnoylCoAMAT → 1.08,
KmlcadC8EnoylCoAMAT → 1.08, KmlcadFADH → 24.2, Keqlcad → 6,
sfmcadC12 → 0.38, sfmcadC10 → 0.8, sfmcadC8 → 0.87, sfmcadC6 → 1, sfmcadC4 → 0.12,
Vmcad → 0.081, KmmcadC12AcylCoAMAT → 5.7, KmmcadC10AcylCoAMAT → 5.4,
KmmcadC8AcylCoAMAT → 4, KmmcadC6AcylCoAMAT → 9.4, KmmcadC4AcylCoAMAT → 135,
KmmcadFAD → 0.12, KmmcadC12EnoylCoAMAT → 1.08, KmmcadC10EnoylCoAMAT → 1.08,
KmmcadC8EnoylCoAMAT → 1.08, KmmcadC6EnoylCoAMAT → 1.08,
KmmcadC4EnoylCoAMAT → 1.08, KmmcadFADH → 24.2, Keqmcad → 6,
sfscadC6 → 0.3, sfscadC4 → 1, Vscad → 0.081, KmscadC6AcylCoAMAT → 285,
KmscadC4AcylCoAMAT → 10.7, KmscadFAD → 0.12, KmscadC6EnoylCoAMAT → 1.08,
KmscadC4EnoylCoAMAT → 1.08, KmscadFADH → 24.2, Keqscad -> 6,
sfcrotC16 → 0.13, sfcrotC14 → 0.2, sfcrotC12 → 0.25, sfcrotC10 → 0.33, sfcrotC8 → 0.58,
sfcrotC6 → 0.83, sfcrotC4 → 1, Vcrot → 3.6, KmcrotC16EnoylCoAMAT → 150,
KmcrotC14EnoylCoAMAT → 100, KmcrotC12EnoylCoAMAT → 25, KmcrotC10EnoylCoAMAT → 25,
KmcrotC8EnoylCoAMAT → 25, KmcrotC6EnoylCoAMAT → 25, KmcrotC4EnoylCoAMAT → 40,
KmcrotC16HydroxyacylCoAMAT → 45, KmcrotC14HydroxyacylCoAMAT → 45,
KmcrotC12HydroxyacylCoAMAT → 45, KmcrotC10HydroxyacylCoAMAT → 45,
KmcrotC8HydroxyacylCoAMAT → 45, KmcrotC6HydroxyacylCoAMAT → 45,
KmcrotC4HydroxyacylCoAMAT → 45, KicrotC4AcetoacylCoA → 1.6, Keqcrot → 3.13,
sfmschadC16 → 0.6, sfmschadC14 → 0.5, sfmschadC12 → 0.43, sfmschadC10 → 0.64,
sfmschadC8 → 0.89, sfmschadC6 → 1, sfmschadC4 → 0.67, Vmschad → 1,
KmmschadC16HydroxyacylCoAMAT → 1.5, KmmschadC14HydroxyacylCoAMAT → 1.8,
KmmschadC12HydroxyacylCoAMAT → 3.7, KmmschadC10HydroxyacylCoAMAT → 8.8,
KmmschadC8HydroxyacylCoAMAT → 16.3, KmmschadC6HydroxyacylCoAMAT → 28.6,
KmmschadC4HydroxyacylCoAMAT → 69.9, KmmschadNADMAT → 58.5,
KmmschadC16KetoacylCoAMAT → 1.4, KmmschadC14KetoacylCoAMAT → 1.4,
KmmschadC12KetoacylCoAMAT → 1.6, KmmschadC10KetoacylCoAMAT → 2.3,
KmmschadC8KetoacylCoAMAT → 4.1, KmmschadC6KetoacylCoAMAT → 5.8,
KmmschadC4AcetoacylCoAMAT → 16.9, KmmschadNADHMAT → 5.4, Keqmschad → 2.17 * 10^-4,
sfmckatC16 → 0, sfmckatC14 → 0.2, sfmckatC12 → 0.38, sfmckatC10 → 0.65,
sfmckatC8 → 0.81, sfmckatC6 → 1, sfmckatC4 → 0.49, Vmckat → 0.377,
KmmckatC16KetoacylCoAMAT → 1.1, KmmckatC14KetoacylCoAMAT → 1.2,
KmmckatC12KetoacylCoAMAT → 1.3, KmmckatC10KetoacylCoAMAT → 2.1,
KmmckatC8KetoacylCoAMAT → 3.2, KmmckatC6KetoacylCoAMAT → 6.7,
KmmckatC4AcetoacylCoAMAT → 12.4, KmmckatCoAMAT → 26.6,
KmmckatC14AcylCoAMAT → 13.83, KmmckatC16AcylCoAMAT → 13.83,
KmmckatC12AcylCoAMAT → 13.83, KmmckatC10AcylCoAMAT → 13.83,
KmmckatC8AcylCoAMAT → 13.83, KmmckatC6AcylCoAMAT → 13.83,
KmmckatC4AcylCoAMAT → 13.83, KmmckatAcetylCoAMAT → 30, Keqmckat → 1051,
sfmtpC16 → 1, sfmtpC14 → 0.9, sfmtpC12 → 0.81, sfmtpC10 → 0.73, sfmtpC8 → 0.34,
Vmtp → 2.84, KmmtpC16EnoylCoAMAT → 25, KmmtpC14EnoylCoAMAT → 25,
KmmtpC12EnoylCoAMAT → 25, KmmtpC10EnoylCoAMAT → 25, KmmtpC8EnoylCoAMAT → 25,
KmmtpNADMAT → 60, KmmtpCoAMAT → 30, KmmtpC14AcylCoAMAT → 13.83,
KmmtpC16AcylCoAMAT → 13.83, KmmtpC12AcylCoAMAT → 13.83,
KmmtpC10AcylCoAMAT → 13.83, KmmtpC8AcylCoAMAT → 13.83, KmmtpC6AcylCoAMAT → 13.83,
KmmtpNADHMAT → 50, KmmtpAcetylCoAMAT → 30, Keqmtp → 0.71,
Ksacesink → 6000000, K1acesink → 70, Ksfadhsink → 6000000,
K1fadhsink → 0.46, Ksnadhsink → 6000000, K1nadhsink → 12,
C16AcylCoACYT → 120, CarCYT → 200, CoACYT → 140, MalCoACYT → 0,
CarMAT -> 950, FADtMAT → 0.77, NADtMAT → 250, CoAMATt → 5000,
VCYT → 2.2 * 10^-6, VMAT → 1.8 * 10^-6, AcetylCoAMAT → 70,
FADHMAT → 0.46, NADHMAT → 12, C4AcetoacylCoAMAT → X};

tsolScan[X_] :=
```

```
  NDSolve[Join[Odes /. RateEqs /. CoAMATX /. ParmScan[X], InitialConditions],
   Vars, {t, 0, 1000000000}];

SsScan[X_] := Module[{SSGuess},
  SSGuess := Table[{Vars[[i]][t],
     (Vars[[i]][900000000] /. tsolScan[X])[[1]]}, {i, 1, Length[Vars]}];
  FindRoot[Table[Odes[[i, 2]] == 0, {i, 1, Length[Odes]}] /. RateEqs /. CoAMATX /.
    ParmScan[X], SSGuess]]

ScanDownNDS[Xstart_, dX_, Xend_] := Monitor[Module[{SS, SSGuess},
   DataDownNDSfluxMCKATC4 = {};
   DataDownNDSfluxMSCHADC4 = {};
   DataDownNDSflux10md = {};
   DataDownNDSc4acylcoad = {};
   DataDownNDSc6acylcoad = {};
   DataDownNDSc4c6acylcoad = {};
   DataDownNDScintermedcoad = {};
   DataDownNDSfreecoad = {};
   DataDownNDScintermedacylcoad = {};
   tsolStart = tsolScan[Xend];
   SSGuess = Table[{Vars[[i]][t],
      (Vars[[i]][900000000] /. tsolStart)[[1]]}, {i, 1, Length[Vars]}];
   SSGuess1 = SSGuess[[All, 1]];
   SSGuess2 = SSGuess[[All, 2]];
   SSGuess1int = SSGuess1 /. t → 0;
   InitialConditionsUD = Thread[SSGuess1int == SSGuess2];

   For[X = Xend, X ≥ Xstart,


    tsolScanNDS = NDSolve[Join[Odes /. RateEqs /. CoAMATX /. ParmScan[X],
       InitialConditionsUD], Vars, {t, 0, 1000000000}];
    SSGuess = Table[{Vars[[i]][t], (Vars[[i]][900000000] /. tsolScanNDS)[[1]]},
      {i, 1, Length[Vars]}];
    SSGuess1 = SSGuess[[All, 1]];
    SSGuess2 = SSGuess[[All, 2]];
    SSGuess1int = SSGuess1 /. t → 0;
    InitialConditionsUD = Thread[SSGuess1int == SSGuess2];
    SS = Thread[SSGuess1 → SSGuess2];

    AppendTo[DataDownNDSfluxMCKATC4,
     {X, 10^3 vmckatC4 /. RateEqs /. CoAMATX /. ParmScan[X] /. SS}];
    AppendTo[DataDownNDSfluxMSCHADC4,
     {X, 10^3 vmschadC4 /. RateEqs /. CoAMATX /. ParmScan[X] /. SS}];
    c4acylcoa = SSGuess2[[39]] ;
    c6acylcoa = SSGuess2[[33]] ;
    c4c6acylcoa = c4acylcoa + c6acylcoa;
    intermediatecoa = SSGuess2[[3]] + SSGuess2[[4]] + SSGuess2[[5]] +
      SSGuess2[[6]] + SSGuess2[[9]] + SSGuess2[[10]] + SSGuess2[[11]] +
      SSGuess2[[12]] + SSGuess2[[15]] + SSGuess2[[16]] + SSGuess2[[17]] +
      SSGuess2[[18]] + SSGuess2[[21]] + SSGuess2[[22]] + SSGuess2[[23]] +
      SSGuess2[[24]] + SSGuess2[[27]] + SSGuess2[[28]] + SSGuess2[[29]] +
      SSGuess2[[30]] + SSGuess2[[33]] + SSGuess2[[34]] + SSGuess2[[35]] +
      SSGuess2[[36]] + SSGuess2[[39]] + SSGuess2[[40]] + SSGuess2[[41]] + X;
```

```
      freeCoa = 5000 - intermediatecoa - 70;
      intermediateacylcoa = SSGuess2[[39]] + SSGuess2[[33]] + SSGuess2[[27]] +
        SSGuess2[[21]] + SSGuess2[[15]] + SSGuess2[[9]] + SSGuess2[[3]];

      AppendTo[DataDownNDSflux10md,
       {X, 10^3 vcactC16 /. RateEqs /. CoAMATX /. ParmScan[X] /. SS}];

      AppendTo[DataDownNDSfluxMCKATC4,
       {X, 10^3 vmckatC4 /. RateEqs /. CoAMATX /. ParmScan[X] /. SS}];
      AppendTo[DataDownNDSfluxMSCHADC4,
       {X, 10^3 vmschadC4 /. RateEqs /. CoAMATX /. ParmScan[X] /. SS}];
      AppendTo[DataDownNDSc4acylcoad, {X, c4acylcoa}];
      AppendTo[DataDownNDSc6acylcoad, {X, c6acylcoa}];
      AppendTo[DataDownNDSc4c6acylcoad, {X, c4c6acylcoa}];
      AppendTo[DataDownNDScintermedcoad, {X, intermediatecoa}];
      AppendTo[DataDownNDSfreecoad, {X, freeCoa}];
      AppendTo[DataDownNDScintermedacylcoad, {X, intermediateacylcoa}];

      X = X - dX;];
    ], ProgressIndicator[X, {Xstart, Xend}]]
```

In[]:=
```
ScanDownNDS[0, 0.01, 10.82]
```

```
ScanUpNDS[Xstart_, dX_, Xend_] := Monitor[Module[{SS, SSGuess},
    DataUpNDSfluxMCKATC4 = {};
    DataUpNDSfluxMSCHADC4 = {};
    DataUpNDSflux10md = {};
    DataUpNDSfluxMCKATC4 = {};
    DataUpNDSfluxMSCHADC4 = {};
    DataUpNDSc4acylcoad = {};
    DataUpNDSc6acylcoad = {};
    DataUpNDSc4c6acylcoad = {};
    DataUpNDScintermedcoad = {};
    DataUpNDSfreecoad = {};
    DataUpNDScintermedacylcoad = {};
    DataUpNDSc8acylcoad = {};
    DataUpNDSc10acylcoad = {};
    DataUpNDSc12acylcoad = {};
    DataUpNDSc14acylcoad = {};
    DataUpNDSc16acylcoad = {};

    tsolStart = tsolScan[Xstart];
    SSGuess = Table[{Vars[[i]][t],
       (Vars[[i]][900 000 000] /. tsolStart)[[1]]}, {i, 1, Length[Vars]}];
    SSGuess1 = SSGuess[[All, 1]];
    SSGuess2 = SSGuess[[All, 2]];
    SSGuess1int = SSGuess1 /. t → 0;
    InitialConditionsUD = Thread[SSGuess1int == SSGuess2];
```

```
For[X = Xstart, X ≤ Xend,


 tsolScanNDS = NDSolve[Join[Odes /. RateEqs /. CoAMATX /. ParmScan[X],
    InitialConditionsUD], Vars, {t, 0, 1000000000}];
 SSGuess = Table[{Vars[[i]][t], (Vars[[i]][900000000] /. tsolScanNDS)[[1]]},
   {i, 1, Length[Vars]}];
 SSGuess1 = SSGuess[[All, 1]];
 SSGuess2 = SSGuess[[All, 2]];
 SSGuess1int = SSGuess1 /. t → 0;
 InitialConditionsUD = Thread[SSGuess1int == SSGuess2];
 SS = Thread[SSGuess1 → SSGuess2];
 c4acylcoa = SSGuess2[[39]] ;
 c6acylcoa = SSGuess2[[33]] ;
 c8acylcoa = SSGuess2[[27]] ;
 c10acylcoa = SSGuess2[[21]] ;
 c12acylcoa = SSGuess2[[15]] ;
 c14acylcoa = SSGuess2[[9]] ;
 c16acylcoa = SSGuess2[[3]] ;

 c4c6acylcoa = c4acylcoa + c6acylcoa;
 intermediatecoa = SSGuess2[[3]] + SSGuess2[[4]] + SSGuess2[[5]] +
   SSGuess2[[6]] + SSGuess2[[9]] + SSGuess2[[10]] + SSGuess2[[11]] +
   SSGuess2[[12]] + SSGuess2[[15]] + SSGuess2[[16]] + SSGuess2[[17]] +
   SSGuess2[[18]] + SSGuess2[[21]] + SSGuess2[[22]] + SSGuess2[[23]] +
   SSGuess2[[24]] + SSGuess2[[27]] + SSGuess2[[28]] + SSGuess2[[29]] +
   SSGuess2[[30]] + SSGuess2[[33]] + SSGuess2[[34]] + SSGuess2[[35]] +
   SSGuess2[[36]] + SSGuess2[[39]] + SSGuess2[[40]] + SSGuess2[[41]] + X;
 freeCoa = 5000 - intermediatecoa - 70;
 intermediateacylcoa = SSGuess2[[39]] + SSGuess2[[33]] + SSGuess2[[27]] +
   SSGuess2[[21]] + SSGuess2[[15]] + SSGuess2[[9]] + SSGuess2[[3]];

 AppendTo[DataUpNDSflux10md,
  {X, 10^3 vcactC16 /. RateEqs /. CoAMATX /. ParmScan[X] /. SS}];

 AppendTo[DataUpNDSfluxMCKATC4,
  {X, 10^3 vmckatC4 /. RateEqs /. CoAMATX /. ParmScan[X] /. SS}];
 AppendTo[DataUpNDSfluxMSCHADC4,
  {X, 10^3 vmschadC4 /. RateEqs /. CoAMATX /. ParmScan[X] /. SS}];
 AppendTo[DataUpNDSc4acylcoad, {X, c4acylcoa}];
 AppendTo[DataUpNDSc6acylcoad, {X, c6acylcoa}];
 AppendTo[DataUpNDSc8acylcoad, {X, c8acylcoa}];
 AppendTo[DataUpNDSc10acylcoad, {X, c10acylcoa}];
 AppendTo[DataUpNDSc12acylcoad, {X, c12acylcoa}];
 AppendTo[DataUpNDSc14acylcoad, {X, c14acylcoa}];
 AppendTo[DataUpNDSc16acylcoad, {X, c16acylcoa}];

 AppendTo[DataUpNDSc4c6acylcoad, {X, c4c6acylcoa}];
 AppendTo[DataUpNDScintermedcoad, {X, intermediatecoa}];
 AppendTo[DataUpNDSfreecoad, {X, freeCoa}];
 AppendTo[DataUpNDScintermedacylcoad, {X, intermediateacylcoa}];

 X = X + dX;];
```

```
], ProgressIndicator[X, {Xstart, Xend}]]
```

In[∘]:=
```
ScanUpNDS[0, 0.01, 10.82]
```

In[∘]:=

In[∘]:=
```
ListLinePlot[{DataDownNDSfluxMCKATC4, DataDownNDSfluxMSCHADC4}, PlotRange → All,
 PlotStyle → {Blue, Green}, PlotLegends → {"vMCKATC4", "vMSCHADC4"}]
```

Out[∘]=

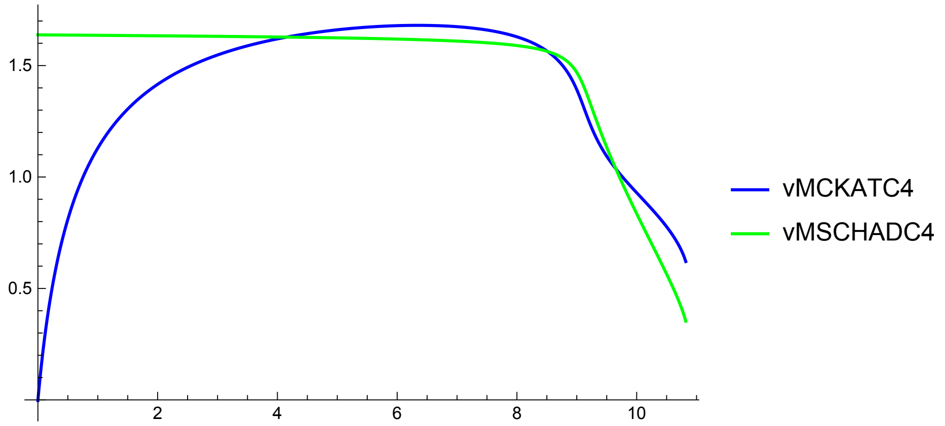


```
(*Plots of AcylCoas and Free CoA*)
```

In[∘]:=
```
t1 = ListLinePlot[
  {DataUpNDSc4acylcoad, DataUpNDSc6acylcoad, DataUpNDSc8acylcoad, DataUpNDSc10acylcoad,
   DataUpNDSc12acylcoad, DataUpNDSc14acylcoad, DataUpNDSc16acylcoad},
  PlotRange → All, PlotStyle → Automatic, AxesStyle → Directive[Black, 18],
  LabelStyle → Directive[Black, 18], PlotLegends →
   {"C4-AcylCoA", "C6-AcylCoA", "C8-AcylCoA", "C10-AcylCoA", "C12-AcylCoA",
    "C14-AcylCoA", "C16-AcylCoA"}, PlotLabel → "Mitochondrial AcylCoAs",
  Frame → {{True, False}, {True, False}}, FrameLabel →
   {{"Concentration(μM)", None}, {"C4-KetoAcylCoA(μM)", None}},
  BaseStyle → {FontSize → 18, FontWeight → ""}, FrameStyle → Thickness[0.00005],
  ImageSize → Scaled[0.25], AspectRatio → 1]
```

Out[∘]=

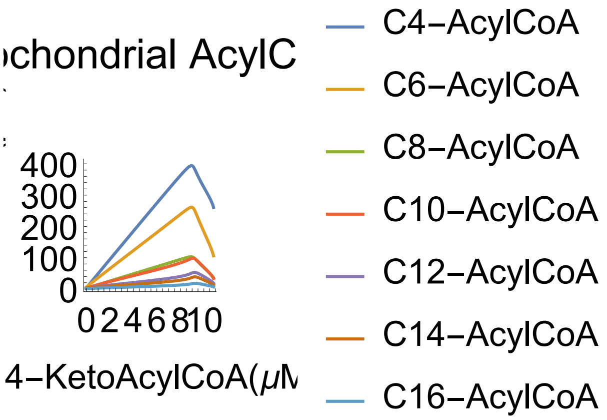

In[ ]:=
```
q1 = ListLinePlot[{DataUpNDSc4acylcoad, DataUpNDSc6acylcoad, DataUpNDSc4c6acylcoad,
   DataUpNDScintermedacylcoad, DataUpNDScintermedcoad, DataUpNDSfreecoad},
  PlotRange → All, PlotStyle → Automatic, AxesStyle → Directive[Black, 18],
  LabelStyle → Directive[Black, 18], PlotLegends →
   {"C4-AcylCoA", "C6-AcylCoA", "C4/C6-AcylCoAs", "Intermediate AcylCoAs",
    "Intermediate CoA Esters", "Free CoA"}, PlotLabel → "CoA",
  Frame → {{True, False}, {True, False}}, FrameLabel →
   {{"Concentration(μM)", None}, {"C4-KetoAcylCoA(μM)", None}},
  BaseStyle → {FontSize → 18, FontWeight → ""}, FrameStyle → Thickness[0.00005],
  ImageSize → Scaled[0.25], AspectRatio → 1]
```

Out[ ]=

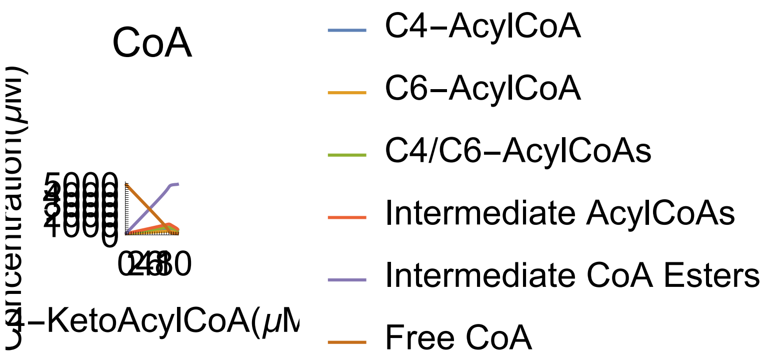

2.Mitochondria Fatty acid Oxidation Kinetic Model - origin and cause of bistability by varying the concentration of C4-KetoacylCoAMAT or C4-AcetoacylCoAMAT where Palmitoyl-CoA is fixed at 50 μM

2.Mitochondria Fatty acid Oxidation Kinetic Model - origin and cause of bistability by varying the concentration of C4-KetoacylCoAMAT or C4-AcetoacylCoAMAT where Palmitoyl-CoA is fixed at 120 μM and inhibition by C4-acylCoAMAT is removed from MCKATC4

Definitions of the various functions

In[∘]:= CPT1[sf_, V_, Kms1_, Kms2_, Kmp1_, Kmp2_, Ki1_, Keq_, S1_, S2_, P1_, P2_, I1_, n_] :=

$$\frac{sf * V * \left(\frac{S1*S2}{Kms1*Kms2} - \frac{P1*P2}{Kms1*Kms2*Keq}\right)}{\left(1 + \frac{S1}{Kms1} + \frac{P1}{Kmp1} + \left(\frac{I1}{Ki1}\right)^{n}\right) * \left(1 + \frac{S2}{Kms2} + \frac{P2}{Kmp2}\right)} \quad (*\left(1-\left(\frac{I1^{n}}{I1^{n}+ Ki1^{n}}\right)\right)*+\left(\frac{I1}{Ki1}\right)^{n}*)$$

In[∘]:= CACT[Vf_, Vr_, Kms1_, Kms2_, Kmp1_, Kmp2_, Kis1_, Kip2_, Keq_, S1_, S2_, P1_, P2_] :=

$$\frac{Vf * \left(S1 * S2 - \frac{P1*P2}{Keq}\right)}{S1 * S2 + Kms2 * S1 + Kms1 * S2 * \left(1 + \frac{P2}{Kip2}\right) + \frac{Vf}{Vr*Keq} * \left(Kmp2 * P1 * \left(1 + \frac{S1}{Kis1}\right) + P2 * (Kmp1 + P1)\right)}$$

In[∘]:= CPT2[sf_, V_, Kms1_, Kms2_, Kms3_, Kms4_, Kms5_, Kms6_, Kms7_, Kms8_, Kmp1_, Kmp2_, Kmp3_, Kmp4_, Kmp5_, Kmp6_, Kmp7_, Kmp8_, Keq_, S1_, S2_, S3_, S4_, S5_, S6_, S7_, S8_, P1_, P2_, P3_, P4_, P5_, P6_, P7_, P8_] :=

$$\left(sf * V * \left(\frac{S1 * S8}{Kms1 * Kms8} - \frac{P1 * P8}{Kms1 * Kms8 * Keq}\right)\right) \Big/ \left(\left(1 + \frac{S1}{Kms1} + \frac{P1}{Kmp1} + \frac{S2}{Kms2} + \frac{P2}{Kmp2} + \frac{S3}{Kms3} + \frac{P3}{Kmp3} + \frac{S4}{Kms4} + \frac{P4}{Kmp4} + \frac{S5}{Kms5} + \frac{P5}{Kmp5} + \frac{S6}{Kms6} + \frac{P6}{Kmp6} + \frac{S7}{Kms7} + \frac{P7}{Kmp7}\right) * \left(1 + \frac{S8}{Kms8} + \frac{P8}{Kmp8}\right)\right)$$

In[∘]:= VLCAD[sf_, V_, Kms1_, Kms2_, Kms3_, Kms4_, Kmp1_, Kmp2_, Kmp3_, Kmp4_, Keq_, S1_, S2_, S3_, S4_, P1_, P2_, P3_, P4_] :=

$$\frac{sf * V * \left(\frac{S1*(S4-P4)}{Kms1*Kms4} - \frac{P1*P4}{Kms1*Kms4*Keq}\right)}{\left(1 + \frac{S1}{Kms1} + \frac{P1}{Kmp1} + \frac{S2}{Kms2} + \frac{P2}{Kmp2} + \frac{S3}{Kms3} + \frac{P3}{Kmp3}\right) * \left(1 + \frac{(S4-P4)}{Kms4} + \frac{P4}{Kmp4}\right)}$$

In[∘]:= LCAD[sf_, V_, Kms1_, Kms2_, Kms3_, Kms4_, Kms5_, Kms6_, Kmp1_, Kmp2_, Kmp3_, Kmp4_, Kmp5_, Kmp6_, Keq_, S1_, S2_, S3_, S4_, S5_, S6_, P1_, P2_, P3_, P4_, P5_, P6_] :=

$$\frac{sf * V * \left(\frac{S1*(S6-P6)}{Kms1*Kms6} - \frac{P1*P6}{Kms1*Kms6*Keq}\right)}{\left(1 + \frac{S1}{Kms1} + \frac{P1}{Kmp1} + \frac{S2}{Kms2} + \frac{P2}{Kmp2} + \frac{S3}{Kms3} + \frac{P3}{Kmp3} + \frac{S4}{Kms4} + \frac{P4}{Kmp4} + \frac{S5}{Kms5} + \frac{P5}{Kmp5}\right) * \left(1 + \frac{(S6-P6)}{Kms6} + \frac{P6}{Kmp6}\right)}$$

In[∘]:= MCAD[sf_, V_, Kms1_, Kms2_, Kms3_, Kms4_, Kms5_, Kms6_, Kmp1_, Kmp2_, Kmp3_, Kmp4_, Kmp5_, Kmp6_, Keq_, S1_, S2_, S3_, S4_, S5_, S6_, P1_, P2_, P3_, P4_, P5_, P6_] :=

$$\frac{sf * V * \left(\frac{S1*(S6-P6)}{Kms1*Kms6} - \frac{P1*P6}{Kms1*Kms6*Keq}\right)}{\left(1 + \frac{S1}{Kms1} + \frac{P1}{Kmp1} + \frac{S2}{Kms2} + \frac{P2}{Kmp2} + \frac{S3}{Kms3} + \frac{P3}{Kmp3} + \frac{S4}{Kms4} + \frac{P4}{Kmp4} + \frac{S5}{Kms5} + \frac{P5}{Kmp5}\right) * \left(1 + \frac{(S6-P6)}{Kms6} + \frac{P6}{Kmp6}\right)}$$

In[∘]:= SCAD[sf_, V_, Kms1_, Kms2_, Kms3_, Kmp1_, Kmp2_, Kmp3_, Keq_, S1_, S2_, S3_, P1_, P2_, P3_] :=

$$\frac{sf * V * \left(\frac{S1*(S3-P3)}{Kms1*Kms3} - \frac{P1*P3}{Kms1*Kms3*Keq}\right)}{\left(1 + \frac{S1}{Kms1} + \frac{P1}{Kmp1} + \frac{S2}{Kms2} + \frac{P2}{Kmp2}\right) * \left(1 + \frac{(S3-P3)}{Kms3} + \frac{P3}{Kmp3}\right)}$$

In[∘]:= CROT[sf_, V_, Kms1_, Kms2_, Kms3_, Kms4_, Kms5_, Kms6_, Kms7_, Kmp1_, Kmp2_, Kmp3_, Kmp4_, Kmp5_, Kmp6_, Kmp7_, Ki1_, Keq_, S1_, S2_, S3_, S4_, S5_, S6_, S7_, P1_, P2_, P3_, P4_, P5_, P6_, P7_, I1_] :=

$$\frac{sf * V * \left(\frac{S1}{Kms1} - \frac{P1}{Kms1*Keq}\right)}{1 + \frac{S1}{Kms1} + \frac{P1}{Kmp1} + \frac{S2}{Kms2} + \frac{P2}{Kmp2} + \frac{S3}{Kms3} + \frac{P3}{Kmp3} + \frac{S4}{Kms4} + \frac{P4}{Kmp4} + \frac{S5}{Kms5} + \frac{P5}{Kmp5} + \frac{S6}{Kms6} + \frac{P6}{Kmp6} + \frac{S7}{Kms7} + \frac{P7}{Kmp7} + \frac{I1}{Ki1}}$$

```
MSCHAD[sf_, V_, Kms1_, Kms2_, Kms3_, Kms4_, Kms5_, Kms6_, Kms7_, Kms8_, Kmp1_, Kmp2_,
  Kmp3_, Kmp4_, Kmp5_, Kmp6_, Kmp7_, Kmp8_, Keq_, S1_, S2_, S3_, S4_, S5_, S6_, S7_, S8_,
  P1_, P2_, P3_, P4_, P5_, P6_, P7_, P8_] := (sf * V * ((S1 * (S8 - P8))/(Kms1 * Kms8) - (P1 * P8)/(Kms1 * Kms8 * Keq))) /
  ((1 + S1/Kms1 + P1/Kmp1 + S2/Kms2 + P2/Kmp2 + S3/Kms3 + P3/Kmp3 + S4/Kms4 + P4/Kmp4 +
      S5/Kms5 + P5/Kmp5 + S6/Kms6 + P6/Kmp6 + S7/Kms7 + P7/Kmp7) * (1 + (S8 - P8)/Kms8 + P8/Kmp8))
```

```
MCKATA[sf_, V_, Kms1_, Kms2_, Kms3_, Kms4_, Kms5_, Kms6_, Kms7_, Kms8_,
  Kmp1_, Kmp2_, Kmp3_, Kmp4_, Kmp5_, Kmp6_, Kmp7_, Kmp8_, Keq_, S1_, S2_,
  S3_, S4_, S5_, S6_, S7_, S8_, P1_, P2_, P3_, P4_, P5_, P6_, P7_, P8_] :=
 (sf * V * ((S1 * S8)/(Kms1 * Kms8) - (P1 * P8)/(Kms1 * Kms8 * Keq))) / ((1 + S1/Kms1 + P1/Kmp1 + S2/Kms2 + P2/Kmp2 + S3/Kms3 + P3/Kmp3 +
      S4/Kms4 + P4/Kmp4 + S5/Kms5 + P5/Kmp5 + S6/Kms6 + P6/Kmp6 + S7/Kms7 + P7/Kmp7 + P8/Kmp8) * (1 + S8/Kms8 + P8/Kmp8))
```

```
MCKATB[sf_, V_, Kms1_, Kms2_, Kms3_, Kms4_, Kms5_, Kms6_, Kms7_, Kms8_,
  Kmp1_, Kmp2_, Kmp3_, Kmp4_, Kmp5_, Kmp6_, Kmp7_, Kmp8_, Keq_, S1_, S2_,
  S3_, S4_, S5_, S6_, S7_, S8_, P1_, P2_, P3_, P4_, P5_, P6_, P7_, P8_] :=
 (sf * V * ((S1 * S8)/(Kms1 * Kms8) - (P8 * P8)/(Kms1 * Kms8 * Keq))) / ((1 + S1/Kms1 + P1/Kmp1 + S2/Kms2 + P2/Kmp2 + S3/Kms3 + P3/Kmp3 +
      S4/Kms4 + P4/Kmp4 + S5/Kms5 + P5/Kmp5 + S6/Kms6 + P6/Kmp6 + S7/Kms7 + P7/Kmp7 + P8/Kmp8) * (1 + S8/Kms8 + P8/Kmp8))
```

```
MTP[sf_, V_, Kms1_, Kms2_, Kms3_, Kms4_, Kms5_, Kms7_, Kms8_, Kmp1_,
  Kmp2_, Kmp3_, Kmp4_, Kmp5_, Kmp6_, Kmp7_, Kmp8_, Ki1_, Keq_, S1_, S2_,
  S3_, S4_, S5_, S7_, S8_, P1_, P2_, P3_, P4_, P5_, P6_, P7_, P8_, I1_] :=
 (sf * V * ((S1 * (S7 - P7) * S8)/(Kms1 * Kms7 * Kms8) - (P1 * P7 * P8)/(Kms1 * Kms7 * Kms8 * Keq))) /
  ((1 + S1/Kms1 + P1/Kmp1 + S2/Kms2 + P2/Kmp2 + S3/Kms3 + P3/Kmp3 + S4/Kms4 + P4/Kmp4 + S5/Kms5 + P5/Kmp5 + P6/Kmp6 + I1/Ki1) *
    (1 + (S7 - P7)/Kms7 + P7/Kmp7) * (1 + S8/Kms8 + P8/Kmp8))
```

```
RES[Ks_, S_, K1_] := Ks * (S - K1)
```

Define the differential equations

```
Odes = {
   C16AcylCarCYT'[t] == (vcpt1C16 - vcactC16)/VCYT,
   C16AcylCarMAT'[t] == (vcactC16 - vcpt2C16)/VMAT,
   C16AcylCoAMAT'[t] == (vcpt2C16 - vvlcadC16 - vlcadC16)/VMAT,
   C16EnoylCoAMAT'[t] == (vvlcadC16 + vlcadC16 - vcrotC16 - vmtpC16)/VMAT,
   C16HydroxyacylCoAMAT'[t] == (vcrotC16 - vmschadC16)/VMAT,
```

$$C16KetoacylCoAMAT'[t] == \frac{vmschadC16 - vmckatC16}{VMAT},$$

$$C14AcylCarCYT'[t] == \frac{-vcactC14}{VCYT},$$

$$C14AcylCarMAT'[t] == \frac{vcactC14 - vcpt2C14}{VMAT},$$

$$C14AcylCoAMAT'[t] == \frac{vcpt2C14 + vmtpC16 + vmckatC16 - vvlcadC14 - vlcadC14}{VMAT},$$

$$C14EnoylCoAMAT'[t] == \frac{vvlcadC14 + vlcadC14 - vcrotC14 - vmtpC14}{VMAT},$$

$$C14HydroxyacylCoAMAT'[t] == \frac{vcrotC14 - vmschadC14}{VMAT},$$

$$C14KetoacylCoAMAT'[t] == \frac{vmschadC14 - vmckatC14}{VMAT},$$

$$C12AcylCarCYT'[t] == \frac{-vcactC12}{VCYT},$$

$$C12AcylCarMAT'[t] == \frac{vcactC12 - vcpt2C12}{VMAT},$$

$$C12AcylCoAMAT'[t] == \frac{vcpt2C12 + vmtpC14 + vmckatC14 - vvlcadC12 - vlcadC12 - vmcadC12}{VMAT},$$

$$C12EnoylCoAMAT'[t] == \frac{vvlcadC12 + vlcadC12 + vmcadC12 - vcrotC12 - vmtpC12}{VMAT},$$

$$C12HydroxyacylCoAMAT'[t] == \frac{vcrotC12 - vmschadC12}{VMAT},$$

$$C12KetoacylCoAMAT'[t] == \frac{vmschadC12 - vmckatC12}{VMAT},$$

$$C10AcylCarCYT'[t] == \frac{-vcactC10}{VCYT},$$

$$C10AcylCarMAT'[t] == \frac{vcactC10 - vcpt2C10}{VMAT},$$

$$C10AcylCoAMAT'[t] == \frac{vcpt2C10 + vmtpC12 + vmckatC12 - vlcadC10 - vmcadC10}{VMAT},$$

$$C10EnoylCoAMAT'[t] == \frac{vlcadC10 + vmcadC10 - vcrotC10 - vmtpC10}{VMAT},$$

$$C10HydroxyacylCoAMAT'[t] == \frac{vcrotC10 - vmschadC10}{VMAT},$$

$$C10KetoacylCoAMAT'[t] == \frac{vmschadC10 - vmckatC10}{VMAT},$$

$$C8AcylCarCYT'[t] == \frac{-vcactC8}{VCYT},$$

$$C8AcylCarMAT'[t] == \frac{vcactC8 - vcpt2C8}{VMAT},$$

$$C8AcylCoAMAT'[t] == \frac{vcpt2C8 + vmtpC10 + vmckatC10 - vlcadC8 - vmcadC8}{VMAT},$$

$$C8EnoylCoAMAT'[t] == \frac{vlcadC8 + vmcadC8 - vcrotC8 - vmtpC8}{VMAT},$$

```
  C8HydroxyacylCoAMAT'[t] == (vcrotC8 - vmschadC8)/VMAT,
  C8KetoacylCoAMAT'[t] == (vmschadC8 - vmckatC8)/VMAT,
  C6AcylCarCYT'[t] == (-vcactC6)/VCYT,
  C6AcylCarMAT'[t] == (vcactC6 - vcpt2C6)/VMAT,
  C6AcylCoAMAT'[t] == (vcpt2C6 + vmtpC8 + vmckatC8 - vmcadC6 - vscadC6)/VMAT,
  C6EnoylCoAMAT'[t] == (vmcadC6 + vscadC6 - vcrotC6)/VMAT,
  C6HydroxyacylCoAMAT'[t] == (vcrotC6 - vmschadC6)/VMAT,
  C6KetoacylCoAMAT'[t] == (vmschadC6 - vmckatC6)/VMAT,
  C4AcylCarCYT'[t] == (-vcactC4)/VCYT,
  C4AcylCarMAT'[t] == (vcactC4 - vcpt2C4)/VMAT,
  C4AcylCoAMAT'[t] == (vcpt2C4 + vmckatC6 - vmcadC4 - vscadC4)/VMAT,
  C4EnoylCoAMAT'[t] == (vmcadC4 + vscadC4 - vcrotC4)/VMAT,
  C4HydroxyacylCoAMAT'[t] == (vcrotC4 - vmschadC4)/VMAT}; (*,*)
(*C4AcetoacylCoAMAT'[t] == (vmschadC4-vmckatC4)/VMAT};*)

RateEqs = {vcpt1C16 → CPT1[sfcpt1C16, Vcpt1, Kmcpt1C16AcylCoACYT,
     Kmcpt1CarCYT, Kmcpt1C16AcylCarCYT, Kmcpt1CoACYT, Kicpt1MalCoACYT, Keqcpt1,
     C16AcylCoACYT, CarCYT, C16AcylCarCYT[t], CoACYT, MalCoACYT, ncpt1],
   vcactC16 → CACT[Vfcact, Vrcact, KmcactC16AcylCarCYT, KmcactCarMAT,
     KmcactC16AcylCarMAT, KmcactCarCYT, KicactC16AcylCarCYT, KicactCarCYT,
     Keqcact, C16AcylCarCYT[t], CarMAT, C16AcylCarMAT[t], CarCYT],
   vcactC14 → CACT[Vfcact, Vrcact, KmcactC14AcylCarCYT, KmcactCarMAT,
     KmcactC14AcylCarMAT, KmcactCarCYT, KicactC14AcylCarCYT, KicactCarCYT,
     Keqcact, C14AcylCarCYT[t], CarMAT, C14AcylCarMAT[t], CarCYT],
   vcactC12 → CACT[Vfcact, Vrcact, KmcactC12AcylCarCYT, KmcactCarMAT,
     KmcactC12AcylCarMAT, KmcactCarCYT, KicactC12AcylCarCYT, KicactCarCYT,
     Keqcact, C12AcylCarCYT[t], CarMAT, C12AcylCarMAT[t], CarCYT],
   vcactC10 → CACT[Vfcact, Vrcact, KmcactC10AcylCarCYT, KmcactCarMAT,
     KmcactC10AcylCarMAT, KmcactCarCYT, KicactC10AcylCarCYT, KicactCarCYT,
     Keqcact, C10AcylCarCYT[t], CarMAT, C10AcylCarMAT[t], CarCYT],
   vcactC8 → CACT[Vfcact, Vrcact, KmcactC8AcylCarCYT, KmcactCarMAT,
     KmcactC8AcylCarMAT, KmcactCarCYT, KicactC8AcylCarCYT, KicactCarCYT,
     Keqcact, C8AcylCarCYT[t], CarMAT, C8AcylCarMAT[t], CarCYT],
   vcactC6 → CACT[Vfcact, Vrcact, KmcactC6AcylCarCYT, KmcactCarMAT,
     KmcactC6AcylCarMAT, KmcactCarCYT, KicactC6AcylCarCYT, KicactCarCYT,
     Keqcact, C6AcylCarCYT[t], CarMAT, C6AcylCarMAT[t], CarCYT],
   vcactC4 → CACT[Vfcact, Vrcact, KmcactC4AcylCarCYT, KmcactCarMAT,
```

```
    KmcactC4AcylCarMAT, KmcactCarCYT, KicactC4AcylCarCYT, KicactCarCYT,
    Keqcact, C4AcylCarCYT[t], CarMAT, C4AcylCarMAT[t], CarCYT],
  vcpt2C16 → CPT2[sfcpt2C16, Vcpt2, Kmcpt2C16AcylCarMAT, Kmcpt2C14AcylCarMAT,
    Kmcpt2C12AcylCarMAT, Kmcpt2C10AcylCarMAT, Kmcpt2C8AcylCarMAT, Kmcpt2C6AcylCarMAT,
    Kmcpt2C4AcylCarMAT, Kmcpt2CoAMAT, Kmcpt2C16AcylCoAMAT, Kmcpt2C14AcylCoAMAT,
    Kmcpt2C12AcylCoAMAT, Kmcpt2C10AcylCoAMAT, Kmcpt2C8AcylCoAMAT, Kmcpt2C6AcylCoAMAT,
    Kmcpt2C4AcylCoAMAT, Kmcpt2CarMAT, Keqcpt2, C16AcylCarMAT[t], C14AcylCarMAT[t],
    C12AcylCarMAT[t], C10AcylCarMAT[t], C8AcylCarMAT[t], C6AcylCarMAT[t],
    C4AcylCarMAT[t], CoAMAT, C16AcylCoAMAT[t], C14AcylCoAMAT[t], C12AcylCoAMAT[t],
    C10AcylCoAMAT[t], C8AcylCoAMAT[t], C6AcylCoAMAT[t], C4AcylCoAMAT[t], CarMAT],
  vcpt2C14 → CPT2[sfcpt2C14, Vcpt2, Kmcpt2C14AcylCarMAT, Kmcpt2C16AcylCarMAT,
    Kmcpt2C12AcylCarMAT, Kmcpt2C10AcylCarMAT, Kmcpt2C8AcylCarMAT, Kmcpt2C6AcylCarMAT,
    Kmcpt2C4AcylCoAMAT, Kmcpt2CoAMAT, Kmcpt2C14AcylCoAMAT, Kmcpt2C16AcylCoAMAT,
    Kmcpt2C12AcylCoAMAT, Kmcpt2C10AcylCoAMAT, Kmcpt2C8AcylCoAMAT, Kmcpt2C6AcylCoAMAT,
    Kmcpt2C4AcylCoAMAT, Kmcpt2CarMAT, Keqcpt2, C14AcylCarMAT[t], C16AcylCarMAT[t],
    C12AcylCarMAT[t], C10AcylCarMAT[t], C8AcylCarMAT[t], C6AcylCarMAT[t],
    C4AcylCarMAT[t], CoAMAT, C14AcylCoAMAT[t], C16AcylCoAMAT[t], C12AcylCoAMAT[t],
    C10AcylCoAMAT[t], C8AcylCoAMAT[t], C6AcylCoAMAT[t], C4AcylCoAMAT[t], CarMAT],
  vcpt2C12 → CPT2[sfcpt2C12, Vcpt2, Kmcpt2C12AcylCarMAT, Kmcpt2C16AcylCarMAT,
    Kmcpt2C14AcylCarMAT, Kmcpt2C10AcylCarMAT, Kmcpt2C8AcylCarMAT, Kmcpt2C6AcylCarMAT,
    Kmcpt2C4AcylCarMAT, Kmcpt2CoAMAT, Kmcpt2C12AcylCoAMAT, Kmcpt2C16AcylCoAMAT,
    Kmcpt2C14AcylCoAMAT, Kmcpt2C10AcylCoAMAT, Kmcpt2C8AcylCoAMAT, Kmcpt2C6AcylCoAMAT,
    Kmcpt2C4AcylCoAMAT, Kmcpt2CarMAT, Keqcpt2, C12AcylCarMAT[t], C16AcylCarMAT[t],
    C14AcylCarMAT[t], C10AcylCarMAT[t], C8AcylCarMAT[t], C6AcylCarMAT[t],
    C4AcylCarMAT[t], CoAMAT, C12AcylCoAMAT[t], C16AcylCoAMAT[t], C14AcylCoAMAT[t],
    C10AcylCoAMAT[t], C8AcylCoAMAT[t], C6AcylCoAMAT[t], C4AcylCoAMAT[t], CarMAT],
  vcpt2C10 → CPT2[sfcpt2C10, Vcpt2, Kmcpt2C10AcylCarMAT, Kmcpt2C16AcylCarMAT,
    Kmcpt2C14AcylCarMAT, Kmcpt2C12AcylCarMAT, Kmcpt2C8AcylCarMAT, Kmcpt2C6AcylCarMAT,
    Kmcpt2C4AcylCarMAT, Kmcpt2CoAMAT, Kmcpt2C10AcylCoAMAT, Kmcpt2C16AcylCoAMAT,
    Kmcpt2C14AcylCoAMAT, Kmcpt2C12AcylCoAMAT, Kmcpt2C8AcylCoAMAT, Kmcpt2C6AcylCoAMAT,
    Kmcpt2C4AcylCoAMAT, Kmcpt2CarMAT, Keqcpt2, C10AcylCarMAT[t], C16AcylCarMAT[t],
    C14AcylCarMAT[t], C12AcylCarMAT[t], C8AcylCarMAT[t], C6AcylCarMAT[t],
    C4AcylCarMAT[t], CoAMAT, C10AcylCoAMAT[t], C16AcylCoAMAT[t], C14AcylCoAMAT[t],
    C12AcylCoAMAT[t], C8AcylCoAMAT[t], C6AcylCoAMAT[t], C4AcylCoAMAT[t], CarMAT],
  vcpt2C8 → CPT2[sfcpt2C8, Vcpt2, Kmcpt2C8AcylCarMAT, Kmcpt2C16AcylCarMAT,
    Kmcpt2C14AcylCarMAT, Kmcpt2C12AcylCarMAT, Kmcpt2C10AcylCarMAT, Kmcpt2C6AcylCarMAT,
    Kmcpt2C4AcylCarMAT, Kmcpt2CoAMAT, Kmcpt2C8AcylCoAMAT, Kmcpt2C16AcylCoAMAT,
    Kmcpt2C14AcylCoAMAT, Kmcpt2C12AcylCoAMAT, Kmcpt2C10AcylCoAMAT, Kmcpt2C6AcylCoAMAT,
    Kmcpt2C4AcylCoAMAT, Kmcpt2CarMAT, Keqcpt2, C8AcylCarMAT[t], C16AcylCarMAT[t],
    C14AcylCarMAT[t], C12AcylCarMAT[t], C10AcylCarMAT[t], C6AcylCarMAT[t],
    C4AcylCarMAT[t], CoAMAT, C8AcylCoAMAT[t], C16AcylCoAMAT[t], C14AcylCoAMAT[t],
    C12AcylCoAMAT[t], C10AcylCoAMAT[t], C6AcylCoAMAT[t], C4AcylCoAMAT[t], CarMAT],
  vcpt2C6 → CPT2[sfcpt2C6, Vcpt2, Kmcpt2C6AcylCarMAT, Kmcpt2C16AcylCarMAT,
    Kmcpt2C14AcylCarMAT, Kmcpt2C12AcylCarMAT, Kmcpt2C10AcylCarMAT, Kmcpt2C8AcylCarMAT,
    Kmcpt2C4AcylCarMAT, Kmcpt2CoAMAT, Kmcpt2C6AcylCoAMAT, Kmcpt2C16AcylCoAMAT,
    Kmcpt2C14AcylCoAMAT, Kmcpt2C12AcylCoAMAT, Kmcpt2C10AcylCoAMAT, Kmcpt2C8AcylCoAMAT,
    Kmcpt2C4AcylCoAMAT, Kmcpt2CarMAT, Keqcpt2, C6AcylCarMAT[t], C16AcylCarMAT[t],
    C14AcylCarMAT[t], C12AcylCarMAT[t], C10AcylCarMAT[t], C8AcylCarMAT[t],
    C4AcylCarMAT[t], CoAMAT, C6AcylCoAMAT[t], C16AcylCoAMAT[t], C14AcylCoAMAT[t],
    C12AcylCoAMAT[t], C10AcylCoAMAT[t], C8AcylCoAMAT[t], C4AcylCoAMAT[t], CarMAT],
  vcpt2C4 → CPT2[sfcpt2C4, Vcpt2, Kmcpt2C4AcylCarMAT, Kmcpt2C16AcylCarMAT,
    Kmcpt2C14AcylCarMAT, Kmcpt2C12AcylCarMAT, Kmcpt2C10AcylCarMAT, Kmcpt2C8AcylCarMAT,
    Kmcpt2C6AcylCarMAT, Kmcpt2CoAMAT, Kmcpt2C4AcylCoAMAT, Kmcpt2C16AcylCoAMAT,
    Kmcpt2C14AcylCoAMAT, Kmcpt2C12AcylCoAMAT, Kmcpt2C10AcylCoAMAT, Kmcpt2C8AcylCoAMAT,
    Kmcpt2C6AcylCoAMAT, Kmcpt2CarMAT, Keqcpt2, C4AcylCarMAT[t], C16AcylCarMAT[t],
```

```
    C14AcylCarMAT[t], C12AcylCarMAT[t], C10AcylCarMAT[t], C8AcylCarMAT[t],
    C6AcylCarMAT[t], CoAMAT, C4AcylCoAMAT[t], C16AcylCoAMAT[t], C14AcylCoAMAT[t],
    C12AcylCoAMAT[t], C10AcylCoAMAT[t], C8AcylCoAMAT[t], C6AcylCoAMAT[t], CarMAT],
  vvlcadC16 → VLCAD[sfvlcadC16, Vvlcad, KmvlcadC16AcylCoAMAT, KmvlcadC14AcylCoAMAT,
    KmvlcadC12AcylCoAMAT, KmvlcadFAD, KmvlcadC16EnoylCoAMAT,
    KmvlcadC14EnoylCoAMAT, KmvlcadC12EnoylCoAMAT, KmvlcadFADH, Keqvlcad,
    C16AcylCoAMAT[t], C14AcylCoAMAT[t], C12AcylCoAMAT[t], FADtMAT,
    C16EnoylCoAMAT[t], C14EnoylCoAMAT[t], C12EnoylCoAMAT[t], FADHMAT],
  vvlcadC14 → VLCAD[sfvlcadC14, Vvlcad, KmvlcadC14AcylCoAMAT, KmvlcadC16AcylCoAMAT,
    KmvlcadC12AcylCoAMAT, KmvlcadFAD, KmvlcadC14EnoylCoAMAT,
    KmvlcadC16EnoylCoAMAT, KmvlcadC12EnoylCoAMAT, KmvlcadFADH, Keqvlcad,
    C14AcylCoAMAT[t], C16AcylCoAMAT[t], C12AcylCoAMAT[t], FADtMAT,
    C14EnoylCoAMAT[t], C16EnoylCoAMAT[t], C12EnoylCoAMAT[t], FADHMAT],
  vvlcadC12 → VLCAD[sfvlcadC12, Vvlcad, KmvlcadC12AcylCoAMAT, KmvlcadC16AcylCoAMAT,
    KmvlcadC14AcylCoAMAT, KmvlcadFAD, KmvlcadC12EnoylCoAMAT,
    KmvlcadC16EnoylCoAMAT, KmvlcadC14EnoylCoAMAT, KmvlcadFADH, Keqvlcad,
    C12AcylCoAMAT[t], C16AcylCoAMAT[t], C14AcylCoAMAT[t], FADtMAT,
    C12EnoylCoAMAT[t], C16EnoylCoAMAT[t], C14EnoylCoAMAT[t], FADHMAT],
  vlcadC16 → LCAD[sflcadC16, Vlcad, KmlcadC16AcylCoAMAT, KmlcadC14AcylCoAMAT,
    KmlcadC12AcylCoAMAT, KmlcadC10AcylCoAMAT, KmlcadC8AcylCoAMAT, KmlcadFAD,
    KmlcadC16EnoylCoAMAT, KmlcadC14EnoylCoAMAT, KmlcadC12EnoylCoAMAT,
    KmlcadC10EnoylCoAMAT, KmlcadC8EnoylCoAMAT, KmlcadFADH, Keqlcad,
    C16AcylCoAMAT[t], C14AcylCoAMAT[t], C12AcylCoAMAT[t], C10AcylCoAMAT[t],
    C8AcylCoAMAT[t], FADtMAT, C16EnoylCoAMAT[t], C14EnoylCoAMAT[t],
    C12EnoylCoAMAT[t], C10EnoylCoAMAT[t], C8EnoylCoAMAT[t], FADHMAT],
  vlcadC14 → LCAD[sflcadC14, Vlcad, KmlcadC14AcylCoAMAT, KmlcadC16AcylCoAMAT,
    KmlcadC12AcylCoAMAT, KmlcadC10AcylCoAMAT, KmlcadC8AcylCoAMAT, KmlcadFAD,
    KmlcadC14EnoylCoAMAT, KmlcadC16EnoylCoAMAT, KmlcadC12EnoylCoAMAT,
    KmlcadC10EnoylCoAMAT, KmlcadC8EnoylCoAMAT, KmlcadFADH, Keqlcad,
    C14AcylCoAMAT[t], C16AcylCoAMAT[t], C12AcylCoAMAT[t], C10AcylCoAMAT[t],
    C8AcylCoAMAT[t], FADtMAT, C14EnoylCoAMAT[t], C16EnoylCoAMAT[t],
    C12EnoylCoAMAT[t], C10EnoylCoAMAT[t], C8EnoylCoAMAT[t], FADHMAT],
  vlcadC12 → LCAD[sflcadC12, Vlcad, KmlcadC12AcylCoAMAT, KmlcadC16AcylCoAMAT,
    KmlcadC14AcylCoAMAT, KmlcadC10AcylCoAMAT, KmlcadC8AcylCoAMAT, KmlcadFAD,
    KmlcadC12EnoylCoAMAT, KmlcadC16EnoylCoAMAT, KmlcadC14EnoylCoAMAT,
    KmlcadC10EnoylCoAMAT, KmlcadC8EnoylCoAMAT, KmlcadFADH, Keqlcad,
    C12AcylCoAMAT[t], C16AcylCoAMAT[t], C14AcylCoAMAT[t], C10AcylCoAMAT[t],
    C8AcylCoAMAT[t], FADtMAT, C14EnoylCoAMAT[t], C16EnoylCoAMAT[t],
    C14EnoylCoAMAT[t], C10EnoylCoAMAT[t], C8EnoylCoAMAT[t], FADHMAT],
  vlcadC10 → LCAD[sflcadC10, Vlcad, KmlcadC10AcylCoAMAT, KmlcadC16AcylCoAMAT,
    KmlcadC14AcylCoAMAT, KmlcadC12AcylCoAMAT, KmlcadC8AcylCoAMAT, KmlcadFAD,
    KmlcadC10EnoylCoAMAT, KmlcadC16EnoylCoAMAT, KmlcadC14EnoylCoAMAT,
    KmlcadC12EnoylCoAMAT, KmlcadC8EnoylCoAMAT, KmlcadFADH, Keqlcad,
    C10AcylCoAMAT[t], C16AcylCoAMAT[t], C14AcylCoAMAT[t], C12AcylCoAMAT[t],
    C8AcylCoAMAT[t], FADtMAT, C10EnoylCoAMAT[t], C16EnoylCoAMAT[t],
    C14EnoylCoAMAT[t], C12EnoylCoAMAT[t], C8EnoylCoAMAT[t], FADHMAT],
  vlcadC8 → LCAD[sflcadC8, Vlcad, KmlcadC8AcylCoAMAT, KmlcadC16AcylCoAMAT,
    KmlcadC14AcylCoAMAT, KmlcadC12AcylCoAMAT, KmlcadC10AcylCoAMAT, KmlcadFAD,
    KmlcadC8EnoylCoAMAT, KmlcadC16EnoylCoAMAT, KmlcadC14EnoylCoAMAT,
    KmlcadC12EnoylCoAMAT, KmlcadC10EnoylCoAMAT, KmlcadFADH, Keqlcad,
    C8AcylCoAMAT[t], C16AcylCoAMAT[t], C14AcylCoAMAT[t], C12AcylCoAMAT[t],
    C10AcylCoAMAT[t], FADtMAT, C8EnoylCoAMAT[t], C16EnoylCoAMAT[t],
    C14EnoylCoAMAT[t], C12EnoylCoAMAT[t], C10EnoylCoAMAT[t], FADHMAT],
  vmcadC12 → MCAD[sfmcadC12, Vmcad, KmmcadC12AcylCoAMAT, KmmcadC10AcylCoAMAT,
    KmmcadC8AcylCoAMAT, KmmcadC6AcylCoAMAT, KmmcadC4AcylCoAMAT, KmmcadFAD,
```

```
   KmmcadC12EnoylCoAMAT, KmmcadC10EnoylCoAMAT, KmmcadC8EnoylCoAMAT,
   KmmcadC6EnoylCoAMAT, KmmcadC4EnoylCoAMAT, KmmcadFADH, Keqmcad,
   C12AcylCoAMAT[t], C10AcylCoAMAT[t], C8AcylCoAMAT[t], C6AcylCoAMAT[t],
   C4AcylCoAMAT[t], FADtMAT, C12EnoylCoAMAT[t], C10EnoylCoAMAT[t],
   C8EnoylCoAMAT[t], C6EnoylCoAMAT[t], C4EnoylCoAMAT[t], FADHMAT],
vmcadC10 → MCAD[sfmcadC10, Vmcad, KmmcadC10AcylCoAMAT, KmmcadC12AcylCoAMAT,
   KmmcadC8AcylCoAMAT, KmmcadC6AcylCoAMAT, KmmcadC4AcylCoAMAT, KmmcadFAD,
   KmmcadC10EnoylCoAMAT, KmmcadC12EnoylCoAMAT, KmmcadC8EnoylCoAMAT,
   KmmcadC6EnoylCoAMAT, KmmcadC4EnoylCoAMAT, KmmcadFADH, Keqmcad,
   C10AcylCoAMAT[t], C12AcylCoAMAT[t], C8AcylCoAMAT[t], C6AcylCoAMAT[t],
   C4AcylCoAMAT[t], FADtMAT, C10EnoylCoAMAT[t], C12EnoylCoAMAT[t],
   C8EnoylCoAMAT[t], C6EnoylCoAMAT[t], C4EnoylCoAMAT[t], FADHMAT],
vmcadC8 → MCAD[sfmcadC8, Vmcad, KmmcadC8AcylCoAMAT, KmmcadC12AcylCoAMAT,
   KmmcadC10AcylCoAMAT, KmmcadC6AcylCoAMAT, KmmcadC4AcylCoAMAT, KmmcadFAD,
   KmmcadC8EnoylCoAMAT, KmmcadC12EnoylCoAMAT, KmmcadC10EnoylCoAMAT,
   KmmcadC6EnoylCoAMAT, KmmcadC4EnoylCoAMAT, KmmcadFADH, Keqmcad,
   C8AcylCoAMAT[t], C12AcylCoAMAT[t], C10AcylCoAMAT[t], C6AcylCoAMAT[t],
   C4AcylCoAMAT[t], FADtMAT, C8EnoylCoAMAT[t], C12EnoylCoAMAT[t],
   C10EnoylCoAMAT[t], C6EnoylCoAMAT[t], C4EnoylCoAMAT[t], FADHMAT],
vmcadC6 → MCAD[sfmcadC6, Vmcad, KmmcadC6AcylCoAMAT, KmmcadC12AcylCoAMAT,
   KmmcadC10AcylCoAMAT, KmmcadC8AcylCoAMAT, KmmcadC4AcylCoAMAT, KmmcadFAD,
   KmmcadC6EnoylCoAMAT, KmmcadC12EnoylCoAMAT, KmmcadC10EnoylCoAMAT,
   KmmcadC8EnoylCoAMAT, KmmcadC4EnoylCoAMAT, KmmcadFADH, Keqmcad,
   C6AcylCoAMAT[t], C12AcylCoAMAT[t], C10AcylCoAMAT[t], C8AcylCoAMAT[t],
   C4AcylCoAMAT[t], FADtMAT, C6EnoylCoAMAT[t], C12EnoylCoAMAT[t],
   C10EnoylCoAMAT[t], C8EnoylCoAMAT[t], C4EnoylCoAMAT[t], FADHMAT],
vmcadC4 → MCAD[sfmcadC4, Vmcad, KmmcadC4AcylCoAMAT, KmmcadC12AcylCoAMAT,
   KmmcadC10AcylCoAMAT, KmmcadC8AcylCoAMAT, KmmcadC6AcylCoAMAT, KmmcadFAD,
   KmmcadC4EnoylCoAMAT, KmmcadC12EnoylCoAMAT, KmmcadC10EnoylCoAMAT,
   KmmcadC8EnoylCoAMAT, KmmcadC6EnoylCoAMAT, KmmcadFADH, Keqmcad,
   C4AcylCoAMAT[t], C12AcylCoAMAT[t], C10AcylCoAMAT[t], C8AcylCoAMAT[t],
   C6AcylCoAMAT[t], FADtMAT, C4EnoylCoAMAT[t], C12EnoylCoAMAT[t],
   C10EnoylCoAMAT[t], C8EnoylCoAMAT[t], C6EnoylCoAMAT[t], FADHMAT],
vscadC6 → SCAD[sfscadC6, Vscad, KmscadC6AcylCoAMAT, KmscadC4AcylCoAMAT, KmscadFAD,
   KmscadC6EnoylCoAMAT, KmscadC4EnoylCoAMAT, KmscadFADH, Keqscad, C6AcylCoAMAT[t],
   C4AcylCoAMAT[t], FADtMAT, C6EnoylCoAMAT[t], C4EnoylCoAMAT[t], FADHMAT],
vscadC4 → SCAD[sfscadC4, Vscad, KmscadC4AcylCoAMAT, KmscadC6AcylCoAMAT, KmscadFAD,
   KmscadC4EnoylCoAMAT, KmscadC6EnoylCoAMAT, KmscadFADH, Keqscad, C4AcylCoAMAT[t],
   C6AcylCoAMAT[t], FADtMAT, C4EnoylCoAMAT[t], C6EnoylCoAMAT[t], FADHMAT],
vcrotC16 → CROT[sfcrotC16, Vcrot, KmcrotC16EnoylCoAMAT, KmcrotC14EnoylCoAMAT,
   KmcrotC12EnoylCoAMAT, KmcrotC10EnoylCoAMAT, KmcrotC8EnoylCoAMAT,
   KmcrotC6EnoylCoAMAT, KmcrotC4EnoylCoAMAT, KmcrotC16HydroxyacylCoAMAT,
   KmcrotC14HydroxyacylCoAMAT, KmcrotC12HydroxyacylCoAMAT,
   KmcrotC10HydroxyacylCoAMAT, KmcrotC8HydroxyacylCoAMAT,
   KmcrotC6HydroxyacylCoAMAT, KmcrotC4HydroxyacylCoAMAT, KicrotC4AcetoacylCoA,
   Keqcrot, C16EnoylCoAMAT[t], C14EnoylCoAMAT[t], C12EnoylCoAMAT[t],
   C10EnoylCoAMAT[t], C8EnoylCoAMAT[t], C6EnoylCoAMAT[t],
   C4EnoylCoAMAT[t], C16HydroxyacylCoAMAT[t], C14HydroxyacylCoAMAT[t],
   C12HydroxyacylCoAMAT[t], C10HydroxyacylCoAMAT[t], C8HydroxyacylCoAMAT[t],
   C6HydroxyacylCoAMAT[t], C4HydroxyacylCoAMAT[t], C4AcetoacylCoAMAT],
vcrotC14 → CROT[sfcrotC14, Vcrot, KmcrotC14EnoylCoAMAT, KmcrotC16EnoylCoAMAT,
   KmcrotC12EnoylCoAMAT, KmcrotC10EnoylCoAMAT, KmcrotC8EnoylCoAMAT,
   KmcrotC6EnoylCoAMAT, KmcrotC4EnoylCoAMAT, KmcrotC14HydroxyacylCoAMAT,
   KmcrotC16HydroxyacylCoAMAT, KmcrotC12HydroxyacylCoAMAT,
   KmcrotC10HydroxyacylCoAMAT, KmcrotC8HydroxyacylCoAMAT,
```

```
    KmcrotC6HydroxyacylCoAMAT, KmcrotC4HydroxyacylCoAMAT, KicrotC4AcetoacylCoA,
    Keqcrot, C14EnoylCoAMAT[t], C16EnoylCoAMAT[t], C12EnoylCoAMAT[t],
    C10EnoylCoAMAT[t], C8EnoylCoAMAT[t], C6EnoylCoAMAT[t],
    C4EnoylCoAMAT[t], C14HydroxyacylCoAMAT[t], C16HydroxyacylCoAMAT[t],
    C12HydroxyacylCoAMAT[t], C10HydroxyacylCoAMAT[t], C8HydroxyacylCoAMAT[t],
    C6HydroxyacylCoAMAT[t], C4HydroxyacylCoAMAT[t], C4AcetoacylCoAMAT],
  vcrotC12 → CROT[sfcrotC12, Vcrot, KmcrotC12EnoylCoAMAT, KmcrotC16EnoylCoAMAT,
    KmcrotC14EnoylCoAMAT, KmcrotC10EnoylCoAMAT, KmcrotC8EnoylCoAMAT,
    KmcrotC6EnoylCoAMAT, KmcrotC4EnoylCoAMAT, KmcrotC12HydroxyacylCoAMAT,
    KmcrotC16HydroxyacylCoAMAT, KmcrotC14HydroxyacylCoAMAT,
    KmcrotC10HydroxyacylCoAMAT, KmcrotC8HydroxyacylCoAMAT,
    KmcrotC6HydroxyacylCoAMAT, KmcrotC4HydroxyacylCoAMAT, KicrotC4AcetoacylCoA,
    Keqcrot, C12EnoylCoAMAT[t], C16EnoylCoAMAT[t], C14EnoylCoAMAT[t],
    C10EnoylCoAMAT[t], C8EnoylCoAMAT[t], C6EnoylCoAMAT[t],
    C4EnoylCoAMAT[t], C12HydroxyacylCoAMAT[t], C16HydroxyacylCoAMAT[t],
    C14HydroxyacylCoAMAT[t], C10HydroxyacylCoAMAT[t], C8HydroxyacylCoAMAT[t],
    C6HydroxyacylCoAMAT[t], C4HydroxyacylCoAMAT[t], C4AcetoacylCoAMAT],
  vcrotC10 → CROT[sfcrotC10, Vcrot, KmcrotC10EnoylCoAMAT, KmcrotC16EnoylCoAMAT,
    KmcrotC14EnoylCoAMAT, KmcrotC12EnoylCoAMAT, KmcrotC8EnoylCoAMAT,
    KmcrotC6EnoylCoAMAT, KmcrotC4EnoylCoAMAT, KmcrotC10HydroxyacylCoAMAT,
    KmcrotC16HydroxyacylCoAMAT, KmcrotC14HydroxyacylCoAMAT,
    KmcrotC12HydroxyacylCoAMAT, KmcrotC8HydroxyacylCoAMAT,
    KmcrotC6HydroxyacylCoAMAT, KmcrotC4HydroxyacylCoAMAT, KicrotC4AcetoacylCoA,
    Keqcrot, C10EnoylCoAMAT[t], C16EnoylCoAMAT[t], C14EnoylCoAMAT[t],
    C12EnoylCoAMAT[t], C8EnoylCoAMAT[t], C6EnoylCoAMAT[t],
    C4EnoylCoAMAT[t], C10HydroxyacylCoAMAT[t], C16HydroxyacylCoAMAT[t],
    C14HydroxyacylCoAMAT[t], C12HydroxyacylCoAMAT[t], C8HydroxyacylCoAMAT[t],
    C6HydroxyacylCoAMAT[t], C4HydroxyacylCoAMAT[t], C4AcetoacylCoAMAT],
  vcrotC8 → CROT[sfcrotC8, Vcrot, KmcrotC8EnoylCoAMAT, KmcrotC16EnoylCoAMAT,
    KmcrotC14EnoylCoAMAT, KmcrotC12EnoylCoAMAT, KmcrotC10EnoylCoAMAT,
    KmcrotC6EnoylCoAMAT, KmcrotC4EnoylCoAMAT, KmcrotC8HydroxyacylCoAMAT,
    KmcrotC16HydroxyacylCoAMAT, KmcrotC14HydroxyacylCoAMAT,
    KmcrotC12HydroxyacylCoAMAT, KmcrotC10HydroxyacylCoAMAT,
    KmcrotC6HydroxyacylCoAMAT, KmcrotC4HydroxyacylCoAMAT, KicrotC4AcetoacylCoA,
    Keqcrot, C8EnoylCoAMAT[t], C16EnoylCoAMAT[t], C14EnoylCoAMAT[t],
    C12EnoylCoAMAT[t], C10EnoylCoAMAT[t], C6EnoylCoAMAT[t],
    C4EnoylCoAMAT[t], C8HydroxyacylCoAMAT[t], C16HydroxyacylCoAMAT[t],
    C14HydroxyacylCoAMAT[t], C12HydroxyacylCoAMAT[t], C10HydroxyacylCoAMAT[t],
    C6HydroxyacylCoAMAT[t], C4HydroxyacylCoAMAT[t], C4AcetoacylCoAMAT],
  vcrotC6 → CROT[sfcrotC6, Vcrot, KmcrotC6EnoylCoAMAT, KmcrotC16EnoylCoAMAT,
    KmcrotC14EnoylCoAMAT, KmcrotC12EnoylCoAMAT, KmcrotC10EnoylCoAMAT,
    KmcrotC8EnoylCoAMAT, KmcrotC4EnoylCoAMAT, KmcrotC6HydroxyacylCoAMAT,
    KmcrotC16HydroxyacylCoAMAT, KmcrotC14HydroxyacylCoAMAT,
    KmcrotC12HydroxyacylCoAMAT, KmcrotC10HydroxyacylCoAMAT,
    KmcrotC8HydroxyacylCoAMAT, KmcrotC4HydroxyacylCoAMAT, KicrotC4AcetoacylCoA,
    Keqcrot, C6EnoylCoAMAT[t], C16EnoylCoAMAT[t], C14EnoylCoAMAT[t],
    C12EnoylCoAMAT[t], C10EnoylCoAMAT[t], C8EnoylCoAMAT[t],
    C4EnoylCoAMAT[t], C6HydroxyacylCoAMAT[t], C16HydroxyacylCoAMAT[t],
    C14HydroxyacylCoAMAT[t], C12HydroxyacylCoAMAT[t], C10HydroxyacylCoAMAT[t],
    C8HydroxyacylCoAMAT[t], C4HydroxyacylCoAMAT[t], C4AcetoacylCoAMAT],
  vcrotC4 → CROT[sfcrotC4, Vcrot, KmcrotC4EnoylCoAMAT, KmcrotC16EnoylCoAMAT,
    KmcrotC14EnoylCoAMAT, KmcrotC12EnoylCoAMAT, KmcrotC10EnoylCoAMAT,
    KmcrotC8EnoylCoAMAT, KmcrotC6EnoylCoAMAT, KmcrotC4HydroxyacylCoAMAT,
    KmcrotC16HydroxyacylCoAMAT, KmcrotC14HydroxyacylCoAMAT,
    KmcrotC12HydroxyacylCoAMAT, KmcrotC10HydroxyacylCoAMAT,
```

```
   KmcrotC8HydroxyacylCoAMAT, KmcrotC6HydroxyacylCoAMAT, KicrotC4AcetoacylCoA,
   Keqcrot, C4EnoylCoAMAT[t], C16EnoylCoAMAT[t], C14EnoylCoAMAT[t],
   C12EnoylCoAMAT[t], C10EnoylCoAMAT[t], C8EnoylCoAMAT[t],
   C6EnoylCoAMAT[t], C4HydroxyacylCoAMAT[t], C16HydroxyacylCoAMAT[t],
   C14HydroxyacylCoAMAT[t], C12HydroxyacylCoAMAT[t], C10HydroxyacylCoAMAT[t],
   C8HydroxyacylCoAMAT[t], C6HydroxyacylCoAMAT[t], C4AcetoacylCoAMAT],
vmschadC16 → MSCHAD[sfmschadC16, Vmschad, KmmschadC16HydroxyacylCoAMAT,
   KmmschadC14HydroxyacylCoAMAT, KmmschadC12HydroxyacylCoAMAT,
   KmmschadC10HydroxyacylCoAMAT, KmmschadC8HydroxyacylCoAMAT,
   KmmschadC6HydroxyacylCoAMAT, KmmschadC4HydroxyacylCoAMAT,
   KmmschadNADMAT, KmmschadC16KetoacylCoAMAT, KmmschadC14KetoacylCoAMAT,
   KmmschadC12KetoacylCoAMAT, KmmschadC10KetoacylCoAMAT, KmmschadC8KetoacylCoAMAT,
   KmmschadC6KetoacylCoAMAT, KmmschadC4AcetoacylCoAMAT, KmmschadNADHMAT,
   Keqmschad, C16HydroxyacylCoAMAT[t], C14HydroxyacylCoAMAT[t],
   C12HydroxyacylCoAMAT[t], C10HydroxyacylCoAMAT[t], C8HydroxyacylCoAMAT[t],
   C6HydroxyacylCoAMAT[t], C4HydroxyacylCoAMAT[t], NADtMAT, C16KetoacylCoAMAT[t],
   C14KetoacylCoAMAT[t], C12KetoacylCoAMAT[t], C10KetoacylCoAMAT[t],
   C8KetoacylCoAMAT[t], C6KetoacylCoAMAT[t], C4AcetoacylCoAMAT, NADHMAT],
vmschadC14 → MSCHAD[sfmschadC14, Vmschad, KmmschadC14HydroxyacylCoAMAT,
   KmmschadC16HydroxyacylCoAMAT, KmmschadC12HydroxyacylCoAMAT,
   KmmschadC10HydroxyacylCoAMAT, KmmschadC8HydroxyacylCoAMAT,
   KmmschadC6HydroxyacylCoAMAT, KmmschadC4HydroxyacylCoAMAT,
   KmmschadNADMAT, KmmschadC14KetoacylCoAMAT, KmmschadC16KetoacylCoAMAT,
   KmmschadC12KetoacylCoAMAT, KmmschadC10KetoacylCoAMAT, KmmschadC8KetoacylCoAMAT,
   KmmschadC6KetoacylCoAMAT, KmmschadC4AcetoacylCoAMAT, KmmschadNADHMAT,
   Keqmschad, C14HydroxyacylCoAMAT[t], C16HydroxyacylCoAMAT[t],
   C12HydroxyacylCoAMAT[t], C10HydroxyacylCoAMAT[t], C8HydroxyacylCoAMAT[t],
   C6HydroxyacylCoAMAT[t], C4HydroxyacylCoAMAT[t], NADtMAT, C14KetoacylCoAMAT[t],
   C16KetoacylCoAMAT[t], C12KetoacylCoAMAT[t], C10KetoacylCoAMAT[t],
   C8KetoacylCoAMAT[t], C6KetoacylCoAMAT[t], C4AcetoacylCoAMAT, NADHMAT],
vmschadC12 → MSCHAD[sfmschadC12, Vmschad, KmmschadC12HydroxyacylCoAMAT,
   KmmschadC16HydroxyacylCoAMAT, KmmschadC14HydroxyacylCoAMAT,
   KmmschadC10HydroxyacylCoAMAT, KmmschadC8HydroxyacylCoAMAT,
   KmmschadC6HydroxyacylCoAMAT, KmmschadC4HydroxyacylCoAMAT,
   KmmschadNADMAT, KmmschadC12KetoacylCoAMAT, KmmschadC16KetoacylCoAMAT,
   KmmschadC14KetoacylCoAMAT, KmmschadC10KetoacylCoAMAT, KmmschadC8KetoacylCoAMAT,
   KmmschadC6KetoacylCoAMAT, KmmschadC4AcetoacylCoAMAT, KmmschadNADHMAT,
   Keqmschad, C12HydroxyacylCoAMAT[t], C16HydroxyacylCoAMAT[t],
   C14HydroxyacylCoAMAT[t], C10HydroxyacylCoAMAT[t], C8HydroxyacylCoAMAT[t],
   C6HydroxyacylCoAMAT[t], C4HydroxyacylCoAMAT[t], NADtMAT, C12KetoacylCoAMAT[t],
   C16KetoacylCoAMAT[t], C14KetoacylCoAMAT[t], C10KetoacylCoAMAT[t],
   C8KetoacylCoAMAT[t], C6KetoacylCoAMAT[t], C4AcetoacylCoAMAT, NADHMAT],
vmschadC10 → MSCHAD[sfmschadC10, Vmschad, KmmschadC10HydroxyacylCoAMAT,
   KmmschadC16HydroxyacylCoAMAT, KmmschadC14HydroxyacylCoAMAT,
   KmmschadC12HydroxyacylCoAMAT, KmmschadC8HydroxyacylCoAMAT,
   KmmschadC6HydroxyacylCoAMAT, KmmschadC4HydroxyacylCoAMAT,
   KmmschadNADMAT, KmmschadC10KetoacylCoAMAT, KmmschadC16KetoacylCoAMAT,
   KmmschadC14KetoacylCoAMAT, KmmschadC12KetoacylCoAMAT, KmmschadC8KetoacylCoAMAT,
   KmmschadC6KetoacylCoAMAT, KmmschadC4AcetoacylCoAMAT, KmmschadNADHMAT,
   Keqmschad, C10HydroxyacylCoAMAT[t], C16HydroxyacylCoAMAT[t],
   C14HydroxyacylCoAMAT[t], C12HydroxyacylCoAMAT[t], C8HydroxyacylCoAMAT[t],
   C6HydroxyacylCoAMAT[t], C4HydroxyacylCoAMAT[t], NADtMAT, C10KetoacylCoAMAT[t],
   C16KetoacylCoAMAT[t], C14KetoacylCoAMAT[t], C12KetoacylCoAMAT[t],
   C8KetoacylCoAMAT[t], C6KetoacylCoAMAT[t], C4AcetoacylCoAMAT, NADHMAT],
vmschadC8 → MSCHAD[sfmschadC8, Vmschad, KmmschadC8HydroxyacylCoAMAT,
```

```
    KmmschadC16HydroxyacylCoAMAT, KmmschadC14HydroxyacylCoAMAT,
    KmmschadC12HydroxyacylCoAMAT, KmmschadC10HydroxyacylCoAMAT,
    KmmschadC6HydroxyacylCoAMAT, KmmschadC4HydroxyacylCoAMAT,
    KmmschadNADMAT, KmmschadC8KetoacylCoAMAT, KmmschadC16KetoacylCoAMAT,
    KmmschadC14KetoacylCoAMAT, KmmschadC12KetoacylCoAMAT, KmmschadC10KetoacylCoAMAT,
    KmmschadC6KetoacylCoAMAT, KmmschadC4AcetoacylCoAMAT, KmmschadNADHMAT,
    Keqmschad, C8HydroxyacylCoAMAT[t], C16HydroxyacylCoAMAT[t],
    C14HydroxyacylCoAMAT[t], C12HydroxyacylCoAMAT[t], C10HydroxyacylCoAMAT[t],
    C6HydroxyacylCoAMAT[t], C4HydroxyacylCoAMAT[t], NADtMAT, C8KetoacylCoAMAT[t],
    C16KetoacylCoAMAT[t], C14KetoacylCoAMAT[t], C12KetoacylCoAMAT[t],
    C10KetoacylCoAMAT[t], C6KetoacylCoAMAT[t], C4AcetoacylCoAMAT, NADHMAT],
  vmschadC6 → MSCHAD[sfmschadC6, Vmschad, KmmschadC6HydroxyacylCoAMAT,
    KmmschadC16HydroxyacylCoAMAT, KmmschadC14HydroxyacylCoAMAT,
    KmmschadC12HydroxyacylCoAMAT, KmmschadC10HydroxyacylCoAMAT,
    KmmschadC8HydroxyacylCoAMAT, KmmschadC4HydroxyacylCoAMAT,
    KmmschadNADMAT, KmmschadC6KetoacylCoAMAT, KmmschadC16KetoacylCoAMAT,
    KmmschadC14KetoacylCoAMAT, KmmschadC12KetoacylCoAMAT, KmmschadC10KetoacylCoAMAT,
    KmmschadC8KetoacylCoAMAT, KmmschadC4AcetoacylCoAMAT, KmmschadNADHMAT,
    Keqmschad, C6HydroxyacylCoAMAT[t], C16HydroxyacylCoAMAT[t],
    C14HydroxyacylCoAMAT[t], C12HydroxyacylCoAMAT[t], C10HydroxyacylCoAMAT[t],
    C8HydroxyacylCoAMAT[t], C4HydroxyacylCoAMAT[t], NADtMAT, C6KetoacylCoAMAT[t],
    C16KetoacylCoAMAT[t], C14KetoacylCoAMAT[t], C12KetoacylCoAMAT[t],
    C10KetoacylCoAMAT[t], C8KetoacylCoAMAT[t], C4AcetoacylCoAMAT, NADHMAT],
  vmschadC4 → MSCHAD[sfmschadC4, Vmschad, KmmschadC4HydroxyacylCoAMAT,
    KmmschadC16HydroxyacylCoAMAT, KmmschadC14HydroxyacylCoAMAT,
    KmmschadC12HydroxyacylCoAMAT, KmmschadC10HydroxyacylCoAMAT,
    KmmschadC8HydroxyacylCoAMAT, KmmschadC6HydroxyacylCoAMAT,
    KmmschadNADMAT, KmmschadC4AcetoacylCoAMAT, KmmschadC16KetoacylCoAMAT,
    KmmschadC14KetoacylCoAMAT, KmmschadC12KetoacylCoAMAT, KmmschadC10KetoacylCoAMAT,
    KmmschadC8KetoacylCoAMAT, KmmschadC6KetoacylCoAMAT, KmmschadNADHMAT,
    Keqmschad, C4HydroxyacylCoAMAT[t], C16HydroxyacylCoAMAT[t],
    C14HydroxyacylCoAMAT[t], C12HydroxyacylCoAMAT[t], C10HydroxyacylCoAMAT[t],
    C8HydroxyacylCoAMAT[t], C6HydroxyacylCoAMAT[t], NADtMAT, C4AcetoacylCoAMAT,
    C16KetoacylCoAMAT[t], C14KetoacylCoAMAT[t], C12KetoacylCoAMAT[t],
    C10KetoacylCoAMAT[t], C8KetoacylCoAMAT[t], C6KetoacylCoAMAT[t], NADHMAT],
  vmckatC16 → MCKATA[sfmckatC16, Vmckat, KmmckatC16KetoacylCoAMAT,
    KmmckatC14KetoacylCoAMAT, KmmckatC12KetoacylCoAMAT, KmmckatC10KetoacylCoAMAT,
    KmmckatC8KetoacylCoAMAT, KmmckatC6KetoacylCoAMAT, KmmckatC4AcetoacylCoAMAT,
    KmmckatCoAMAT, KmmckatC14AcylCoAMAT, KmmckatC16AcylCoAMAT, KmmckatC12AcylCoAMAT,
    KmmckatC10AcylCoAMAT, KmmckatC8AcylCoAMAT, KmmckatC6AcylCoAMAT,
    KmmckatC4AcylCoAMAT, KmmckatAcetylCoAMAT, Keqmckat, C16KetoacylCoAMAT[t],
    C14KetoacylCoAMAT[t], C12KetoacylCoAMAT[t], C10KetoacylCoAMAT[t],
    C8KetoacylCoAMAT[t], C6KetoacylCoAMAT[t], C4AcetoacylCoAMAT, CoAMAT,
    C14AcylCoAMAT[t], C16AcylCoAMAT[t], C12AcylCoAMAT[t], C10AcylCoAMAT[t],
    C8AcylCoAMAT[t], C6AcylCoAMAT[t], C4AcylCoAMAT[t], AcetylCoAMAT],
  vmckatC14 → MCKATA[sfmckatC14, Vmckat, KmmckatC14KetoacylCoAMAT,
    KmmckatC16KetoacylCoAMAT, KmmckatC12KetoacylCoAMAT, KmmckatC10KetoacylCoAMAT,
    KmmckatC8KetoacylCoAMAT, KmmckatC6KetoacylCoAMAT, KmmckatC4AcetoacylCoAMAT,
    KmmckatCoAMAT, KmmckatC12AcylCoAMAT, KmmckatC16AcylCoAMAT, KmmckatC14AcylCoAMAT,
    KmmckatC10AcylCoAMAT, KmmckatC8AcylCoAMAT, KmmckatC6AcylCoAMAT,
    KmmckatC4AcylCoAMAT, KmmckatAcetylCoAMAT, Keqmckat, C14KetoacylCoAMAT[t],
    C16KetoacylCoAMAT[t], C12KetoacylCoAMAT[t], C10KetoacylCoAMAT[t],
    C8KetoacylCoAMAT[t], C6KetoacylCoAMAT[t], C4AcetoacylCoAMAT, CoAMAT,
    C12AcylCoAMAT[t], C16AcylCoAMAT[t], C14AcylCoAMAT[t], C10AcylCoAMAT[t],
    C8AcylCoAMAT[t], C6AcylCoAMAT[t], C4AcylCoAMAT[t], AcetylCoAMAT],
```

```
vmckatC12 → MCKATA[sfmckatC12, Vmckat, KmmckatC12KetoacylCoAMAT,
   KmmckatC16KetoacylCoAMAT, KmmckatC14KetoacylCoAMAT, KmmckatC10KetoacylCoAMAT,
   KmmckatC8KetoacylCoAMAT, KmmckatC6KetoacylCoAMAT, KmmckatC4AcetoacylCoAMAT,
   KmmckatCoAMAT, KmmckatC10AcylCoAMAT, KmmckatC16AcylCoAMAT, KmmckatC14AcylCoAMAT,
   KmmckatC12AcylCoAMAT, KmmckatC8AcylCoAMAT, KmmckatC6AcylCoAMAT,
   KmmckatC4AcylCoAMAT, KmmckatAcetylCoAMAT, Keqmckat, C12KetoacylCoAMAT[t],
   C16KetoacylCoAMAT[t], C14KetoacylCoAMAT[t], C10KetoacylCoAMAT[t],
   C8KetoacylCoAMAT[t], C6KetoacylCoAMAT[t], C4AcetoacylCoAMAT, CoAMAT,
   C10AcylCoAMAT[t], C16AcylCoAMAT[t], C14AcylCoAMAT[t], C12AcylCoAMAT[t],
   C8AcylCoAMAT[t], C6AcylCoAMAT[t], C4AcylCoAMAT[t], AcetylCoAMAT],
vmckatC10 → MCKATA[sfmckatC10, Vmckat, KmmckatC10KetoacylCoAMAT,
   KmmckatC16KetoacylCoAMAT, KmmckatC14KetoacylCoAMAT, KmmckatC12KetoacylCoAMAT,
   KmmckatC8KetoacylCoAMAT, KmmckatC6KetoacylCoAMAT, KmmckatC4AcetoacylCoAMAT,
   KmmckatCoAMAT, KmmckatC8AcylCoAMAT, KmmckatC16AcylCoAMAT, KmmckatC14AcylCoAMAT,
   KmmckatC12AcylCoAMAT, KmmckatC10AcylCoAMAT, KmmckatC6AcylCoAMAT,
   KmmckatC4AcylCoAMAT, KmmckatAcetylCoAMAT, Keqmckat, C10KetoacylCoAMAT[t],
   C16KetoacylCoAMAT[t], C14KetoacylCoAMAT[t], C12KetoacylCoAMAT[t],
   C8KetoacylCoAMAT[t], C6KetoacylCoAMAT[t], C4AcetoacylCoAMAT, CoAMAT,
   C8AcylCoAMAT[t], C16AcylCoAMAT[t], C14AcylCoAMAT[t], C12AcylCoAMAT[t],
   C10AcylCoAMAT[t], C6AcylCoAMAT[t], C4AcylCoAMAT[t], AcetylCoAMAT],
vmckatC8 → MCKATA[sfmckatC8, Vmckat, KmmckatC8KetoacylCoAMAT,
   KmmckatC16KetoacylCoAMAT, KmmckatC14KetoacylCoAMAT, KmmckatC12KetoacylCoAMAT,
   KmmckatC10KetoacylCoAMAT, KmmckatC6KetoacylCoAMAT, KmmckatC4AcetoacylCoAMAT,
   KmmckatCoAMAT, KmmckatC6AcylCoAMAT, KmmckatC16AcylCoAMAT, KmmckatC14AcylCoAMAT,
   KmmckatC12AcylCoAMAT, KmmckatC10AcylCoAMAT, KmmckatC8AcylCoAMAT,
   KmmckatC4AcylCoAMAT, KmmckatAcetylCoAMAT, Keqmckat, C8KetoacylCoAMAT[t],
   C16KetoacylCoAMAT[t], C14KetoacylCoAMAT[t], C12KetoacylCoAMAT[t],
   C10KetoacylCoAMAT[t], C6KetoacylCoAMAT[t], C4AcetoacylCoAMAT, CoAMAT,
   C6AcylCoAMAT[t], C16AcylCoAMAT[t], C14AcylCoAMAT[t], C12AcylCoAMAT[t],
   C10AcylCoAMAT[t], C8AcylCoAMAT[t], C4AcylCoAMAT[t], AcetylCoAMAT],
vmckatC6 → MCKATA[sfmckatC6, Vmckat, KmmckatC6KetoacylCoAMAT,
   KmmckatC16KetoacylCoAMAT, KmmckatC14KetoacylCoAMAT,
   KmmckatC12KetoacylCoAMAT, KmmckatC10KetoacylCoAMAT, KmmckatC8KetoacylCoAMAT,
   KmmckatC4AcetoacylCoAMAT, KmmckatCoAMAT, KmmckatC4AcylCoAMAT,
   KmmckatC16AcylCoAMAT, KmmckatC14AcylCoAMAT, KmmckatC12AcylCoAMAT,
   KmmckatC10AcylCoAMAT, KmmckatC8AcylCoAMAT, KmmckatC6AcylCoAMAT,
   KmmckatAcetylCoAMAT, Keqmckat, C6KetoacylCoAMAT[t], C16KetoacylCoAMAT[t],
   C14KetoacylCoAMAT[t], C12KetoacylCoAMAT[t], C10KetoacylCoAMAT[t],
   C8KetoacylCoAMAT[t], C4AcetoacylCoAMAT, CoAMAT, C4AcylCoAMAT[t],
   C16AcylCoAMAT[t], C14AcylCoAMAT[t], C12AcylCoAMAT[t], C10AcylCoAMAT[t],
   C8AcylCoAMAT[t], C6AcylCoAMAT[t], AcetylCoAMAT], (*C6AcylCoAMAT[t]*)
vmckatC4 → MCKATB[sfmckatC4, Vmckat, KmmckatC4AcetoacylCoAMAT,
   KmmckatC16KetoacylCoAMAT, KmmckatC14KetoacylCoAMAT, KmmckatC12KetoacylCoAMAT,
   KmmckatC10KetoacylCoAMAT, KmmckatC8KetoacylCoAMAT, KmmckatC6KetoacylCoAMAT,
   KmmckatCoAMAT, KmmckatC4AcylCoAMAT, KmmckatC16AcylCoAMAT, KmmckatC14AcylCoAMAT,
   KmmckatC12AcylCoAMAT, KmmckatC10AcylCoAMAT, KmmckatC8AcylCoAMAT,
   KmmckatC6AcylCoAMAT, KmmckatAcetylCoAMAT, Keqmckat, C4AcetoacylCoAMAT,
   C16KetoacylCoAMAT[t], C14KetoacylCoAMAT[t], C12KetoacylCoAMAT[t],
   C10KetoacylCoAMAT[t], C8KetoacylCoAMAT[t], C6KetoacylCoAMAT[t], CoAMAT, 0,
   C16AcylCoAMAT[t], C14AcylCoAMAT[t], C12AcylCoAMAT[t], C10AcylCoAMAT[t],
   C8AcylCoAMAT[t], C6AcylCoAMAT[t], AcetylCoAMAT], (*C4AcylCoAMAT[t]*)
vmtpC16 → MTP[sfmtpC16, Vmtp, KmmtpC16EnoylCoAMAT, KmmtpC14EnoylCoAMAT,
   KmmtpC12EnoylCoAMAT, KmmtpC10EnoylCoAMAT, KmmtpC8EnoylCoAMAT,
   KmmtpNADMAT, KmmtpCoAMAT, KmmtpC14AcylCoAMAT, KmmtpC16AcylCoAMAT,
   KmmtpC12AcylCoAMAT, KmmtpC10AcylCoAMAT, KmmtpC8AcylCoAMAT,
```

```
      KmmtpC6AcylCoAMAT, KmmtpNADHMAT, KmmtpAcetylCoAMAT, KicrotC4AcetoacylCoA,
      Keqmtp, C16EnoylCoAMAT[t], C14EnoylCoAMAT[t], C12EnoylCoAMAT[t],
      C10EnoylCoAMAT[t], C8EnoylCoAMAT[t], NADtMAT, CoAMAT, C14AcylCoAMAT[t],
      C16AcylCoAMAT[t], C12AcylCoAMAT[t], C10AcylCoAMAT[t], C8AcylCoAMAT[t],
      C6AcylCoAMAT[t], NADHMAT, AcetylCoAMAT, C4AcetoacylCoAMAT],
    vmtpC14 → MTP[sfmtpC14, Vmtp, KmmtpC14EnoylCoAMAT, KmmtpC16EnoylCoAMAT,
      KmmtpC12EnoylCoAMAT, KmmtpC10EnoylCoAMAT, KmmtpC8EnoylCoAMAT,
      KmmtpNADMAT, KmmtpCoAMAT, KmmtpC12AcylCoAMAT, KmmtpC16AcylCoAMAT,
      KmmtpC14AcylCoAMAT, KmmtpC10AcylCoAMAT, KmmtpC8AcylCoAMAT,
      KmmtpC6AcylCoAMAT, KmmtpNADHMAT, KmmtpAcetylCoAMAT, KicrotC4AcetoacylCoA,
      Keqmtp, C14EnoylCoAMAT[t], C16EnoylCoAMAT[t], C12EnoylCoAMAT[t],
      C10EnoylCoAMAT[t], C8EnoylCoAMAT[t], NADtMAT, CoAMAT, C12AcylCoAMAT[t],
      C16AcylCoAMAT[t], C14AcylCoAMAT[t], C10AcylCoAMAT[t], C8AcylCoAMAT[t],
      C6AcylCoAMAT[t], NADHMAT, AcetylCoAMAT, C4AcetoacylCoAMAT],
    vmtpC12 → MTP[sfmtpC12, Vmtp, KmmtpC12EnoylCoAMAT, KmmtpC16EnoylCoAMAT,
      KmmtpC14EnoylCoAMAT, KmmtpC10EnoylCoAMAT, KmmtpC8EnoylCoAMAT,
      KmmtpNADMAT, KmmtpCoAMAT, KmmtpC10AcylCoAMAT, KmmtpC16AcylCoAMAT,
      KmmtpC14AcylCoAMAT, KmmtpC12AcylCoAMAT, KmmtpC8AcylCoAMAT,
      KmmtpC6AcylCoAMAT, KmmtpNADHMAT, KmmtpAcetylCoAMAT, KicrotC4AcetoacylCoA,
      Keqmtp, C12EnoylCoAMAT[t], C16EnoylCoAMAT[t], C14EnoylCoAMAT[t],
      C10EnoylCoAMAT[t], C8EnoylCoAMAT[t], NADtMAT, CoAMAT, C10AcylCoAMAT[t],
      C16AcylCoAMAT[t], C14AcylCoAMAT[t], C12AcylCoAMAT[t], C8AcylCoAMAT[t],
      C6AcylCoAMAT[t], NADHMAT, AcetylCoAMAT, C4AcetoacylCoAMAT],
    vmtpC10 → MTP[sfmtpC10, Vmtp, KmmtpC10EnoylCoAMAT, KmmtpC16EnoylCoAMAT,
      KmmtpC14EnoylCoAMAT, KmmtpC12EnoylCoAMAT, KmmtpC8EnoylCoAMAT,
      KmmtpNADMAT, KmmtpCoAMAT, KmmtpC8AcylCoAMAT, KmmtpC16AcylCoAMAT,
      KmmtpC14AcylCoAMAT, KmmtpC12AcylCoAMAT, KmmtpC10AcylCoAMAT,
      KmmtpC6AcylCoAMAT, KmmtpNADHMAT, KmmtpAcetylCoAMAT, KicrotC4AcetoacylCoA,
      Keqmtp, C10EnoylCoAMAT[t], C16EnoylCoAMAT[t], C14EnoylCoAMAT[t],
      C12EnoylCoAMAT[t], C8EnoylCoAMAT[t], NADtMAT, CoAMAT, C8AcylCoAMAT[t],
      C16AcylCoAMAT[t], C14AcylCoAMAT[t], C12AcylCoAMAT[t], C10AcylCoAMAT[t],
      C6AcylCoAMAT[t], NADHMAT, AcetylCoAMAT, C4AcetoacylCoAMAT],
    vmtpC8 → MTP[sfmtpC8, Vmtp, KmmtpC8EnoylCoAMAT, KmmtpC16EnoylCoAMAT,
      KmmtpC14EnoylCoAMAT, KmmtpC12EnoylCoAMAT, KmmtpC10EnoylCoAMAT,
      KmmtpNADMAT, KmmtpCoAMAT, KmmtpC6AcylCoAMAT, KmmtpC16AcylCoAMAT,
      KmmtpC14AcylCoAMAT, KmmtpC12AcylCoAMAT, KmmtpC10AcylCoAMAT,
      KmmtpC8AcylCoAMAT, KmmtpNADHMAT, KmmtpAcetylCoAMAT, KicrotC4AcetoacylCoA,
      Keqmtp, C8EnoylCoAMAT[t], C16EnoylCoAMAT[t], C14EnoylCoAMAT[t],
      C12EnoylCoAMAT[t], C10EnoylCoAMAT[t], NADtMAT, CoAMAT, C6AcylCoAMAT[t],
      C16AcylCoAMAT[t], C14AcylCoAMAT[t], C12AcylCoAMAT[t], C10AcylCoAMAT[t],
      C8AcylCoAMAT[t], NADHMAT, AcetylCoAMAT, C4AcetoacylCoAMAT],
    vacesink → RES[Ksacesink, AcetylCoAMAT, K1acesink],
    vfadhsink → RES[Ksfadhsink, FADHMAT, K1fadhsink],
    vnadhsink → RES[Ksnadhsink, NADHMAT, K1nadhsink]};

CoAMATX =
  {CoAMAT → CoAMATt - C16AcylCoAMAT[t] - C16EnoylCoAMAT[t] - C16HydroxyacylCoAMAT[t] -
      C16KetoacylCoAMAT[t] - C14AcylCoAMAT[t] - C14EnoylCoAMAT[t] -
      C14HydroxyacylCoAMAT[t] - C14KetoacylCoAMAT[t] - C12AcylCoAMAT[t] -
      C12EnoylCoAMAT[t] - C12HydroxyacylCoAMAT[t] - C12KetoacylCoAMAT[t] -
      C10AcylCoAMAT[t] - C10EnoylCoAMAT[t] - C10HydroxyacylCoAMAT[t] -
      C10KetoacylCoAMAT[t] - C8AcylCoAMAT[t] - C8EnoylCoAMAT[t] -
      C8HydroxyacylCoAMAT[t] - C8KetoacylCoAMAT[t] - C6AcylCoAMAT[t] - C6EnoylCoAMAT[t] -
      C6HydroxyacylCoAMAT[t] - C6KetoacylCoAMAT[t] - C4AcylCoAMAT[t] -
      C4EnoylCoAMAT[t] - C4HydroxyacylCoAMAT[t] - C4AcetoacylCoAMAT - AcetylCoAMAT};
```

```
Parm = {
   sfcpt1C16 → 1, Vcpt1 → 0.012, Kmcpt1C16AcylCoACYT → 13.8,
   Kmcpt1CarCYT → 250, Kmcpt1C16AcylCarCYT → 136, Kmcpt1CoACYT → 40.7,
   Kicpt1MalCoACYT → 9.1, Keqcpt1 → 0.45, ncpt1 → 2.4799,
   Vfcact → 0.42, Vrcact → 0.42, KmcactC16AcylCarCYT → 15,
   KmcactC14AcylCarCYT → 15, KmcactC12AcylCarCYT → 15, KmcactC10AcylCarCYT → 15,
   KmcactC8AcylCarCYT → 15, KmcactC6AcylCarCYT → 15, KmcactC4AcylCarCYT → 15,
   KmcactCarMAT → 130, KmcactC16AcylCarMAT → 15, KmcactC14AcylCarMAT → 15,
   KmcactC12AcylCarMAT → 15, KmcactC10AcylCarMAT → 15, KmcactC8AcylCarMAT → 15,
   KmcactC6AcylCarMAT → 15, KmcactC4AcylCarMAT → 15, KmcactCarCYT → 130,
   KicactC16AcylCarCYT → 56, KicactC14AcylCarCYT → 56, KicactC12AcylCarCYT → 56,
   KicactC10AcylCarCYT → 56, KicactC8AcylCarCYT → 56, KicactC6AcylCarCYT → 56,
   KicactC4AcylCarCYT → 56, KicactCarCYT → 200, Keqcact → 1,
   sfcpt2C16 → 0.85, sfcpt2C14 → 1, sfcpt2C12 → 0.95, sfcpt2C10 → 0.95,
   sfcpt2C8 → 0.35, sfcpt2C6 → 0.15, sfcpt2C4 → 0.01, Vcpt2 → 0.391,
   Kmcpt2C16AcylCarMAT → 51, Kmcpt2C14AcylCarMAT → 51, Kmcpt2C12AcylCarMAT → 51,
   Kmcpt2C10AcylCarMAT → 51, Kmcpt2C8AcylCarMAT → 51, Kmcpt2C6AcylCarMAT → 51,
   Kmcpt2C4AcylCarMAT → 51, Kmcpt2CoAMAT → 30, Kmcpt2C16AcylCoAMAT → 38,
   Kmcpt2C14AcylCoAMAT → 38, Kmcpt2C12AcylCoAMAT → 38,
   Kmcpt2C10AcylCoAMAT → 38, Kmcpt2C8AcylCoAMAT → 38, Kmcpt2C6AcylCoAMAT → 1000,
   Kmcpt2C4AcylCoAMAT → 1000000, Kmcpt2CarMAT → 350, Keqcpt2 → 2.22,
   sfvlcadC16 → 1, sfvlcadC14 → 0.42, sfvlcadC12 → 0.11, Vvlcad → 0.008,
   KmvlcadC16AcylCoAMAT → 6.5, KmvlcadC14AcylCoAMAT → 4, KmvlcadC12AcylCoAMAT → 2.7,
   KmvlcadFAD → 0.12, KmvlcadC16EnoylCoAMAT → 1.08, KmvlcadC14EnoylCoAMAT → 1.08,
   KmvlcadC12EnoylCoAMAT → 1.08, KmvlcadFADH → 24.2, Keqvlcad → 6,
   sflcadC16 → 0.9, sflcadC14 → 1, sflcadC12 → 0.9, sflcadC10 → 0.75, sflcadC8 → 0.4,
   Vlcad → 0.01, KmlcadC16AcylCoAMAT → 2.5, KmlcadC14AcylCoAMAT → 7.4,
   KmlcadC12AcylCoAMAT → 9, KmlcadC10AcylCoAMAT → 24.3, KmlcadC8AcylCoAMAT → 123,
   KmlcadFAD → 0.12, KmlcadC16EnoylCoAMAT → 1.08, KmlcadC14EnoylCoAMAT → 1.08,
   KmlcadC12EnoylCoAMAT → 1.08, KmlcadC10EnoylCoAMAT → 1.08,
   KmlcadC8EnoylCoAMAT → 1.08, KmlcadFADH → 24.2, Keqlcad → 6,
   sfmcadC12 → 0.38, sfmcadC10 → 0.8, sfmcadC8 → 0.87, sfmcadC6 → 1, sfmcadC4 → 0.12,
   Vmcad → 0.081, KmmcadC12AcylCoAMAT → 5.7, KmmcadC10AcylCoAMAT → 5.4,
   KmmcadC8AcylCoAMAT → 4, KmmcadC6AcylCoAMAT → 9.4, KmmcadC4AcylCoAMAT → 135,
   KmmcadFAD → 0.12, KmmcadC12EnoylCoAMAT → 1.08, KmmcadC10EnoylCoAMAT → 1.08,
   KmmcadC8EnoylCoAMAT → 1.08, KmmcadC6EnoylCoAMAT → 1.08,
   KmmcadC4EnoylCoAMAT → 1.08, KmmcadFADH → 24.2, Keqmcad → 6,
   sfscadC6 → 0.3, sfscadC4 → 1, Vscad → 0.081, KmscadC6AcylCoAMAT → 285,
   KmscadC4AcylCoAMAT → 10.7, KmscadFAD → 0.12, KmscadC6EnoylCoAMAT → 1.08,
   KmscadC4EnoylCoAMAT → 1.08, KmscadFADH → 24.2, Keqscad -> 6,
   sfcrotC16 → 0.13, sfcrotC14 → 0.2, sfcrotC12 → 0.25, sfcrotC10 → 0.33, sfcrotC8 → 0.58,
   sfcrotC6 → 0.83, sfcrotC4 → 1, Vcrot → 3.6, KmcrotC16EnoylCoAMAT → 150,
   KmcrotC14EnoylCoAMAT → 100, KmcrotC12EnoylCoAMAT → 25, KmcrotC10EnoylCoAMAT → 25,
   KmcrotC8EnoylCoAMAT → 25, KmcrotC6EnoylCoAMAT → 25, KmcrotC4EnoylCoAMAT → 40,
   KmcrotC16HydroxyacylCoAMAT → 45, KmcrotC14HydroxyacylCoAMAT → 45,
   KmcrotC12HydroxyacylCoAMAT → 45, KmcrotC10HydroxyacylCoAMAT → 45,
   KmcrotC8HydroxyacylCoAMAT → 45, KmcrotC6HydroxyacylCoAMAT → 45,
   KmcrotC4HydroxyacylCoAMAT → 45, KicrotC4AcetoacylCoA → 1.6, Keqcrot → 3.13,
   sfmschadC16 → 0.6, sfmschadC14 → 0.5, sfmschadC12 → 0.43, sfmschadC10 → 0.64,
   sfmschadC8 → 0.89, sfmschadC6 → 1, sfmschadC4 → 0.67, Vmschad → 1,
   KmmschadC16HydroxyacylCoAMAT → 1.5, KmmschadC14HydroxyacylCoAMAT → 1.8,
   KmmschadC12HydroxyacylCoAMAT → 3.7, KmmschadC10HydroxyacylCoAMAT → 8.8,
   KmmschadC8HydroxyacylCoAMAT → 16.3, KmmschadC6HydroxyacylCoAMAT → 28.6,
   KmmschadC4HydroxyacylCoAMAT → 69.9, KmmschadNADMAT → 58.5,
```

```
    KmmschadC16KetoacylCoAMAT → 1.4, KmmschadC14KetoacylCoAMAT → 1.4,
    KmmschadC12KetoacylCoAMAT → 1.6, KmmschadC10KetoacylCoAMAT → 2.3,
    KmmschadC8KetoacylCoAMAT → 4.1, KmmschadC6KetoacylCoAMAT → 5.8,
    KmmschadC4AcetoacylCoAMAT → 16.9, KmmschadNADHMAT → 5.4, Keqmschad → 2.17 * 10^-4,
    sfmckatC16 → 0, sfmckatC14 → 0.2, sfmckatC12 → 0.38, sfmckatC10 → 0.65,
    sfmckatC8 → 0.81, sfmckatC6 → 1, sfmckatC4 → 0.49, Vmckat → 0.377,
    KmmckatC16KetoacylCoAMAT → 1.1, KmmckatC14KetoacylCoAMAT → 1.2,
    KmmckatC12KetoacylCoAMAT → 1.3, KmmckatC10KetoacylCoAMAT → 2.1,
    KmmckatC8KetoacylCoAMAT → 3.2, KmmckatC6KetoacylCoAMAT → 6.7,
    KmmckatC4AcetoacylCoAMAT → 12.4, KmmckatCoAMAT → 26.6,
    KmmckatC14AcylCoAMAT → 13.83, KmmckatC16AcylCoAMAT → 13.83,
    KmmckatC12AcylCoAMAT → 13.83, KmmckatC10AcylCoAMAT → 13.83,
    KmmckatC8AcylCoAMAT → 13.83, KmmckatC6AcylCoAMAT → 13.83,
    KmmckatC4AcylCoAMAT → 13.83, KmmckatAcetylCoAMAT → 30, Keqmckat → 1051,
    sfmtpC16 → 1, sfmtpC14 → 0.9, sfmtpC12 → 0.81, sfmtpC10 → 0.73, sfmtpC8 → 0.34,
    Vmtp → 2.84, KmmtpC16EnoylCoAMAT → 25, KmmtpC14EnoylCoAMAT → 25,
    KmmtpC12EnoylCoAMAT → 25, KmmtpC10EnoylCoAMAT → 25, KmmtpC8EnoylCoAMAT → 25,
    KmmtpNADMAT → 60, KmmtpCoAMAT → 30, KmmtpC14AcylCoAMAT → 13.83,
    KmmtpC16AcylCoAMAT → 13.83, KmmtpC12AcylCoAMAT → 13.83,
    KmmtpC10AcylCoAMAT → 13.83, KmmtpC8AcylCoAMAT → 13.83, KmmtpC6AcylCoAMAT → 13.83,
    KmmtpNADHMAT → 50, KmmtpAcetylCoAMAT → 30, Keqmtp → 0.71,
    Ksacesink → 6000000, K1acesink → 70, Ksfadhsink → 6000000,
    K1fadhsink → 0.46, Ksnadhsink → 6000000, K1nadhsink → 12,
    C16AcylCoACYT → 25, CarCYT → 200, CoACYT → 140, MalCoACYT → 0,
    CarMAT -> 950, FADtMAT → 0.77, NADtMAT → 250, CoAMATt → 5000,
    VCYT → 2.2 * 10^-6, VMAT → 1.8 * 10^-6, AcetylCoAMAT → 70,
    FADHMAT → 0.46, NADHMAT → 12, C4AcetoacylCoAMAT → 12.50};

InitialConditions = {
    C16AcylCarCYT[0] == 0, C16AcylCarMAT[0] == 0, C16AcylCoAMAT[0] == 0,
    C16EnoylCoAMAT[0] == 0, C16HydroxyacylCoAMAT[0] == 0, C16KetoacylCoAMAT[0] == 0,
    C14AcylCarCYT[0] == 0, C14AcylCarMAT[0] == 0, C14AcylCoAMAT[0] == 0,
    C14EnoylCoAMAT[0] == 0, C14HydroxyacylCoAMAT[0] == 0, C14KetoacylCoAMAT[0] == 0,
    C12AcylCarCYT[0] == 0, C12AcylCarMAT[0] == 0, C12AcylCoAMAT[0] == 0,
    C12EnoylCoAMAT[0] == 0, C12HydroxyacylCoAMAT[0] == 0, C12KetoacylCoAMAT[0] == 0,
    C10AcylCarCYT[0] == 0, C10AcylCarMAT[0] == 0, C10AcylCoAMAT[0] == 0,
    C10EnoylCoAMAT[0] == 0, C10HydroxyacylCoAMAT[0] == 0, C10KetoacylCoAMAT[0] == 0,
    C8AcylCarCYT[0] == 0, C8AcylCarMAT[0] == 0, C8AcylCoAMAT[0] == 0,
    C8EnoylCoAMAT[0] == 0, C8HydroxyacylCoAMAT[0] == 0, C8KetoacylCoAMAT[0] == 0,
    C6AcylCarCYT[0] == 0, C6AcylCarMAT[0] == 0, C6AcylCoAMAT[0] == 0,
    C6EnoylCoAMAT[0] == 0, C6HydroxyacylCoAMAT[0] == 0, C6KetoacylCoAMAT[0] == 0,
    C4AcylCarCYT[0] == 0, C4AcylCarMAT[0] == 0, C4AcylCoAMAT[0] == 0, C4EnoylCoAMAT[0] == 0,
    C4HydroxyacylCoAMAT[0] == 0 }; (*,C4AcetoacylCoAMAT[0]==0};*)

Vars = {
    C16AcylCarCYT, C16AcylCarMAT, C16AcylCoAMAT,
    C16EnoylCoAMAT, C16HydroxyacylCoAMAT, C16KetoacylCoAMAT,
    C14AcylCarCYT, C14AcylCarMAT, C14AcylCoAMAT, C14EnoylCoAMAT,
    C14HydroxyacylCoAMAT, C14KetoacylCoAMAT,
    C12AcylCarCYT, C12AcylCarMAT, C12AcylCoAMAT, C12EnoylCoAMAT,
    C12HydroxyacylCoAMAT, C12KetoacylCoAMAT,
    C10AcylCarCYT, C10AcylCarMAT, C10AcylCoAMAT, C10EnoylCoAMAT,
    C10HydroxyacylCoAMAT, C10KetoacylCoAMAT,
    C8AcylCarCYT, C8AcylCarMAT, C8AcylCoAMAT, C8EnoylCoAMAT,
    C8HydroxyacylCoAMAT, C8KetoacylCoAMAT,
```

```
    C6AcylCarCYT, C6AcylCarMAT, C6AcylCoAMAT, C6EnoylCoAMAT,
    C6HydroxyacylCoAMAT, C6KetoacylCoAMAT,
    C4AcylCarCYT, C4AcylCarMAT, C4AcylCoAMAT, C4EnoylCoAMAT, C4HydroxyacylCoAMAT};
```

In[∘]:=
```
TableForm[Odes];
TableForm[RateEqs];
TableForm[Odes /. RateEqs /. CoAMATX /. Parm ];
TableForm[RateEqs /. Parm ];
TableForm[InitialConditions];
```

In[∘]:=
```
tsol = NDSolve[Join[Odes /. RateEqs /. CoAMATX /. Parm , InitialConditions],
    Vars, {t, 0, 1000000000}];
```

In[∘]:=
```
Table[{Vars[[i]][t], (Vars[[i]][900000000] /. tsol)[[1]]}, {i, 1, Length[Vars]}]
```

Out[∘]=
```
{{C16AcylCarCYT[t], 9.11471}, {C16AcylCarMAT[t], 42.9596}, {C16AcylCoAMAT[t], 2.94733},
 {C16EnoylCoAMAT[t], 0.913049}, {C16HydroxyacylCoAMAT[t], 2.85784},
 {C16KetoacylCoAMAT[t], 0.0122997}, {C14AcylCarCYT[t], 9.51902},
 {C14AcylCarMAT[t], 45.2154}, {C14AcylCoAMAT[t], 5.70439}, {C14EnoylCoAMAT[t], 1.2966},
 {C14HydroxyacylCoAMAT[t], 3.91616}, {C14KetoacylCoAMAT[t], 0.0168491},
 {C12AcylCarCYT[t], 15.7357}, {C12AcylCarMAT[t], 74.7445},
 {C12AcylCoAMAT[t], 9.42979}, {C12EnoylCoAMAT[t], 3.17582},
 {C12HydroxyacylCoAMAT[t], 9.88026}, {C12KetoacylCoAMAT[t], 0.0424959},
 {C10AcylCarCYT[t], 48.9878}, {C10AcylCarMAT[t], 232.692},
 {C10AcylCoAMAT[t], 29.3566}, {C10EnoylCoAMAT[t], 4.16939},
 {C10HydroxyacylCoAMAT[t], 12.9962}, {C10KetoacylCoAMAT[t], 0.0558822},
 {C8AcylCarCYT[t], 66.0779}, {C8AcylCarMAT[t], 313.87}, {C8AcylCoAMAT[t], 39.598},
 {C8EnoylCoAMAT[t], 10.8578}, {C8HydroxyacylCoAMAT[t], 33.9303},
 {C8KetoacylCoAMAT[t], 0.145908}, {C6AcylCarCYT[t], 176.654},
 {C6AcylCarMAT[t], 839.106}, {C6AcylCoAMAT[t], 105.862}, {C6EnoylCoAMAT[t], 102.987},
 {C6HydroxyacylCoAMAT[t], 320.908}, {C6KetoacylCoAMAT[t], 1.37395},
 {C4AcylCarCYT[t], 534.203}, {C4AcylCarMAT[t], 2537.46}, {C4AcylCoAMAT[t], 320.127},
 {C4EnoylCoAMAT[t], 930.476}, {C4HydroxyacylCoAMAT[t], 2910.48}}
```

In[∘]:=

In[∘]:=
```
ParmScan[X_] := {
    sfcpt1C16 → 1, Vcpt1 → 0.012, Kmcpt1C16AcylCoACYT → 13.8,
    Kmcpt1CarCYT → 250, Kmcpt1C16AcylCarCYT → 136, Kmcpt1CoACYT → 40.7,
    Kicpt1MalCoACYT → 9.1, Keqcpt1 → 0.45,  ncpt1 → 2.4799,
    Vfcact → 0.42, Vrcact → 0.42, KmcactC16AcylCarCYT → 15,
    KmcactC14AcylCarCYT → 15, KmcactC12AcylCarCYT → 15, KmcactC10AcylCarCYT → 15,
    KmcactC8AcylCarCYT → 15, KmcactC6AcylCarCYT → 15, KmcactC4AcylCarCYT → 15,
    KmcactCarMAT → 130, KmcactC16AcylCarMAT → 15, KmcactC14AcylCarMAT → 15,
    KmcactC12AcylCarMAT → 15, KmcactC10AcylCarMAT → 15, KmcactC8AcylCarMAT → 15,
    KmcactC6AcylCarMAT → 15, KmcactC4AcylCarMAT → 15, KmcactCarCYT → 130,
    KicactC16AcylCarCYT → 56, KicactC14AcylCarCYT → 56, KicactC12AcylCarCYT → 56,
    KicactC10AcylCarCYT → 56, KicactC8AcylCarCYT → 56, KicactC6AcylCarCYT → 56,
    KicactC4AcylCarCYT → 56, KicactCarCYT → 200, Keqcact → 1,
    sfcpt2C16 → 0.85, sfcpt2C14 → 1, sfcpt2C12 → 0.95, sfcpt2C10 → 0.95,
    sfcpt2C8 → 0.35, sfcpt2C6 → 0.15, sfcpt2C4 → 0.01, Vcpt2 → 0.391,
    Kmcpt2C16AcylCarMAT → 51, Kmcpt2C14AcylCarMAT → 51, Kmcpt2C12AcylCarMAT → 51,
    Kmcpt2C10AcylCarMAT → 51, Kmcpt2C8AcylCarMAT → 51, Kmcpt2C6AcylCarMAT → 51,
    Kmcpt2C4AcylCarMAT → 51, Kmcpt2CoAMAT → 30, Kmcpt2C16AcylCoAMAT → 38,
```

```
Kmcpt2C14AcylCoAMAT → 38, Kmcpt2C12AcylCoAMAT → 38,
Kmcpt2C10AcylCoAMAT → 38, Kmcpt2C8AcylCoAMAT → 38, Kmcpt2C6AcylCoAMAT → 1000,
Kmcpt2C4AcylCoAMAT → 1 000 000, Kmcpt2CarMAT → 350, Keqcpt2 → 2.22,
sfvlcadC16 → 1, sfvlcadC14 → 0.42, sfvlcadC12 → 0.11, Vvlcad → 0.008,
KmvlcadC16AcylCoAMAT → 6.5, KmvlcadC14AcylCoAMAT → 4, KmvlcadC12AcylCoAMAT → 2.7,
KmvlcadFAD → 0.12, KmvlcadC16EnoylCoAMAT → 1.08, KmvlcadC14EnoylCoAMAT → 1.08,
KmvlcadC12EnoylCoAMAT → 1.08, KmvlcadFADH → 24.2, Keqvlcad → 6,
sflcadC16 → 0.9, sflcadC14 → 1, sflcadC12 → 0.9, sflcadC10 → 0.75, sflcadC8 → 0.4,
Vlcad → 0.01, KmlcadC16AcylCoAMAT → 2.5, KmlcadC14AcylCoAMAT → 7.4,
KmlcadC12AcylCoAMAT → 9, KmlcadC10AcylCoAMAT → 24.3, KmlcadC8AcylCoAMAT → 123,
KmlcadFAD → 0.12, KmlcadC16EnoylCoAMAT → 1.08, KmlcadC14EnoylCoAMAT → 1.08,
KmlcadC12EnoylCoAMAT → 1.08, KmlcadC10EnoylCoAMAT → 1.08,
KmlcadC8EnoylCoAMAT → 1.08, KmlcadFADH → 24.2, Keqlcad → 6,
sfmcadC12 → 0.38, sfmcadC10 → 0.8, sfmcadC8 → 0.87, sfmcadC6 → 1, sfmcadC4 → 0.12,
Vmcad → 0.081, KmmcadC12AcylCoAMAT → 5.7, KmmcadC10AcylCoAMAT → 5.4,
KmmcadC8AcylCoAMAT → 4, KmmcadC6AcylCoAMAT → 9.4, KmmcadC4AcylCoAMAT → 135,
KmmcadFAD → 0.12, KmmcadC12EnoylCoAMAT → 1.08, KmmcadC10EnoylCoAMAT → 1.08,
KmmcadC8EnoylCoAMAT → 1.08, KmmcadC6EnoylCoAMAT → 1.08,
KmmcadC4EnoylCoAMAT → 1.08, KmmcadFADH → 24.2, Keqmcad → 6,
sfscadC6 → 0.3, sfscadC4 → 1, Vscad → 0.081, KmscadC6AcylCoAMAT → 285,
KmscadC4AcylCoAMAT → 10.7, KmscadFAD → 0.12, KmscadC6EnoylCoAMAT → 1.08,
KmscadC4EnoylCoAMAT → 1.08, KmscadFADH → 24.2, Keqscad -> 6,
sfcrotC16 → 0.13, sfcrotC14 → 0.2, sfcrotC12 → 0.25, sfcrotC10 → 0.33, sfcrotC8 → 0.58,
sfcrotC6 → 0.83, sfcrotC4 → 1, Vcrot → 3.6, KmcrotC16EnoylCoAMAT → 150,
KmcrotC14EnoylCoAMAT → 100, KmcrotC12EnoylCoAMAT → 25, KmcrotC10EnoylCoAMAT → 25,
KmcrotC8EnoylCoAMAT → 25, KmcrotC6EnoylCoAMAT → 25, KmcrotC4EnoylCoAMAT → 40,
KmcrotC16HydroxyacylCoAMAT → 45, KmcrotC14HydroxyacylCoAMAT → 45,
KmcrotC12HydroxyacylCoAMAT → 45, KmcrotC10HydroxyacylCoAMAT → 45,
KmcrotC8HydroxyacylCoAMAT → 45, KmcrotC6HydroxyacylCoAMAT → 45,
KmcrotC4HydroxyacylCoAMAT → 45, KicrotC4AcetoacylCoA → 1.6, Keqcrot → 3.13,
sfmschadC16 → 0.6, sfmschadC14 → 0.5, sfmschadC12 → 0.43, sfmschadC10 → 0.64,
sfmschadC8 → 0.89, sfmschadC6 → 1, sfmschadC4 → 0.67, Vmschad → 1,
KmmschadC16HydroxyacylCoAMAT → 1.5, KmmschadC14HydroxyacylCoAMAT → 1.8,
KmmschadC12HydroxyacylCoAMAT → 3.7, KmmschadC10HydroxyacylCoAMAT → 8.8,
KmmschadC8HydroxyacylCoAMAT → 16.3, KmmschadC6HydroxyacylCoAMAT → 28.6,
KmmschadC4HydroxyacylCoAMAT → 69.9, KmmschadNADMAT → 58.5,
KmmschadC16KetoacylCoAMAT → 1.4, KmmschadC14KetoacylCoAMAT → 1.4,
KmmschadC12KetoacylCoAMAT → 1.6, KmmschadC10KetoacylCoAMAT → 2.3,
KmmschadC8KetoacylCoAMAT → 4.1, KmmschadC6KetoacylCoAMAT → 5.8,
KmmschadC4AcetoacylCoAMAT → 16.9, KmmschadNADHMAT → 5.4, Keqmschad → 2.17 * 10^-4,
sfmckatC16 → 0, sfmckatC14 → 0.2, sfmckatC12 → 0.38, sfmckatC10 → 0.65,
sfmckatC8 → 0.81, sfmckatC6 → 1, sfmckatC4 → 0.49, Vmckat → 0.377,
KmmckatC16KetoacylCoAMAT → 1.1, KmmckatC14KetoacylCoAMAT → 1.2,
KmmckatC12KetoacylCoAMAT → 1.3, KmmckatC10KetoacylCoAMAT → 2.1,
KmmckatC8KetoacylCoAMAT → 3.2, KmmckatC6KetoacylCoAMAT → 6.7,
KmmckatC4AcetoacylCoAMAT → 12.4, KmmckatCoAMAT → 26.6,
KmmckatC14AcylCoAMAT → 13.83, KmmckatC16AcylCoAMAT → 13.83,
KmmckatC12AcylCoAMAT → 13.83, KmmckatC10AcylCoAMAT → 13.83,
KmmckatC8AcylCoAMAT → 13.83, KmmckatC6AcylCoAMAT → 13.83,
KmmckatC4AcylCoAMAT → 13.83, KmmckatAcetylCoAMAT → 30, Keqmckat → 1051,
sfmtpC16 → 1, sfmtpC14 → 0.9, sfmtpC12 → 0.81, sfmtpC10 → 0.73, sfmtpC8 → 0.34,
Vmtp → 2.84, KmmtpC16EnoylCoAMAT → 25, KmmtpC14EnoylCoAMAT → 25,
KmmtpC12EnoylCoAMAT → 25, KmmtpC10EnoylCoAMAT → 25, KmmtpC8EnoylCoAMAT → 25,
KmmtpNADMAT → 60, KmmtpCoAMAT → 30, KmmtpC14AcylCoAMAT → 13.83,
KmmtpC16AcylCoAMAT → 13.83, KmmtpC12AcylCoAMAT → 13.83,
```

```
KmmtpC10AcylCoAMAT → 13.83, KmmtpC8AcylCoAMAT → 13.83, KmmtpC6AcylCoAMAT → 13.83,
KmmtpNADHMAT → 50, KmmtpAcetylCoAMAT → 30, Keqmtp → 0.71,
Ksacesink → 6000000, K1acesink → 70, Ksfadhsink → 6000000,
K1fadhsink → 0.46, Ksnadhsink → 6000000, K1nadhsink → 12,
C16AcylCoACYT → 120, CarCYT → 200, CoACYT → 140, MalCoACYT → 0,
CarMAT -> 950, FADtMAT → 0.77, NADtMAT → 250, CoAMATt → 5000,
VCYT → 2.2 * 10^-6, VMAT → 1.8 * 10^-6, AcetylCoAMAT → 70,
FADHMAT → 0.46, NADHMAT → 12, C4AcetoacylCoAMAT → X};

tsolScan[X_] :=
  NDSolve[Join[Odes /. RateEqs /. CoAMATX /. ParmScan[X], InitialConditions],
   Vars, {t, 0, 1000000000}];

SsScan[X_] := Module[{SSGuess},
  SSGuess := Table[{Vars[[i]][t],
     (Vars[[i]][900000000] /. tsolScan[X])[[1]]}, {i, 1, Length[Vars]}];
  FindRoot[Table[Odes[[i, 2]] == 0, {i, 1, Length[Odes]}] /. RateEqs /. CoAMATX /.
    ParmScan[X], SSGuess]]

In[•]:= ScanDownNDS[Xstart_, dX_, Xend_] := Monitor[Module[{SS, SSGuess},
   DataDownNDSfluxMCKATC4 = {};
   DataDownNDSfluxMSCHADC4 = {};
   DataDownNDSflux10md = {};
   DataDownNDSc4acylcoad = {};
   DataDownNDSc6acylcoad = {};
   DataDownNDSc4c6acylcoad = {};
   DataDownNDScintermedcoad = {};
   DataDownNDSfreecoad = {};
   DataDownNDScintermedacylcoad = {};
   tsolStart = tsolScan[Xend];
   SSGuess = Table[{Vars[[i]][t],
      (Vars[[i]][900000000] /. tsolStart)[[1]]}, {i, 1, Length[Vars]}];
   SSGuess1 = SSGuess[[All, 1]];
   SSGuess2 = SSGuess[[All, 2]];
   SSGuess1int = SSGuess1 /. t → 0;
   InitialConditionsUD = Thread[SSGuess1int == SSGuess2];

   For[X = Xend, X ≥ Xstart,


    tsolScanNDS = NDSolve[Join[Odes /. RateEqs /. CoAMATX /. ParmScan[X],
       InitialConditionsUD], Vars, {t, 0, 1000000000}];
    SSGuess = Table[{Vars[[i]][t], (Vars[[i]][900000000] /. tsolScanNDS)[[1]]},
      {i, 1, Length[Vars]}];
    SSGuess1 = SSGuess[[All, 1]];
    SSGuess2 = SSGuess[[All, 2]];
    SSGuess1int = SSGuess1 /. t → 0;
    InitialConditionsUD = Thread[SSGuess1int == SSGuess2];
    SS = Thread[SSGuess1 → SSGuess2];

    AppendTo[DataDownNDSfluxMCKATC4,
     {X, 10^3 vmckatC4 /. RateEqs /. CoAMATX /. ParmScan[X] /. SS}];
    AppendTo[DataDownNDSfluxMSCHADC4,
     {X, 10^3 vmschadC4 /. RateEqs /. CoAMATX /. ParmScan[X] /. SS}];
```

```
      c4acylcoa = SSGuess2[[39]];
      c6acylcoa = SSGuess2[[33]];
      c4c6acylcoa = c4acylcoa + c6acylcoa;
      intermediatecoa = SSGuess2[[3]] + SSGuess2[[4]] + SSGuess2[[5]] +
        SSGuess2[[6]] + SSGuess2[[9]] + SSGuess2[[10]] + SSGuess2[[11]] +
        SSGuess2[[12]] + SSGuess2[[15]] + SSGuess2[[16]] + SSGuess2[[17]] +
        SSGuess2[[18]] + SSGuess2[[21]] + SSGuess2[[22]] + SSGuess2[[23]] +
        SSGuess2[[24]] + SSGuess2[[27]] + SSGuess2[[28]] + SSGuess2[[29]] +
        SSGuess2[[30]] + SSGuess2[[33]] + SSGuess2[[34]] + SSGuess2[[35]] +
        SSGuess2[[36]] + SSGuess2[[39]] + SSGuess2[[40]] + SSGuess2[[41]] + X;
      freeCoa = 5000 - intermediatecoa - 70;
      intermediateacylcoa = SSGuess2[[39]] + SSGuess2[[33]] + SSGuess2[[27]] +
        SSGuess2[[21]] + SSGuess2[[15]] + SSGuess2[[9]] + SSGuess2[[3]];

      AppendTo[DataDownNDSflux10md,
       {X, 10^3 vcactC16 /. RateEqs /. CoAMATX /. ParmScan[X] /. SS}];

      AppendTo[DataDownNDSfluxMCKATC4,
       {X, 10^3 vmckatC4 /. RateEqs /. CoAMATX /. ParmScan[X] /. SS}];
      AppendTo[DataDownNDSfluxMSCHADC4,
       {X, 10^3 vmschadC4 /. RateEqs /. CoAMATX /. ParmScan[X] /. SS}];
      AppendTo[DataDownNDSc4acylcoad, {X, c4acylcoa}];
      AppendTo[DataDownNDSc6acylcoad, {X, c6acylcoa}];
      AppendTo[DataDownNDSc4c6acylcoad, {X, c4c6acylcoa}];
      AppendTo[DataDownNDScintermedcoad, {X, intermediatecoa}];
      AppendTo[DataDownNDSfreecoad, {X, freeCoa}];
      AppendTo[DataDownNDScintermedacylcoad, {X, intermediateacylcoa}];

      X = X - dX;];
    ], ProgressIndicator[X, {Xstart, Xend}]]
```

```
In[•]:= ScanDownNDS[0, 0.01, 10.82]
```

```
In[•]:= ScanUpNDS[Xstart_, dX_, Xend_] := Monitor[Module[{SS, SSGuess},
     DataUpNDSfluxMCKATC4 = {};
     DataUpNDSfluxMSCHADC4 = {};
     DataUpNDSflux10md = {};
     DataUpNDSfluxMCKATC4 = {};
     DataUpNDSfluxMSCHADC4 = {};
     DataUpNDSc4acylcoad = {};
     DataUpNDSc6acylcoad = {};
     DataUpNDSc4c6acylcoad = {};
     DataUpNDScintermedcoad = {};
     DataUpNDSfreecoad = {};
     DataUpNDScintermedacylcoad = {};
     DataUpNDSc8acylcoad = {};
     DataUpNDSc10acylcoad = {};
     DataUpNDSc12acylcoad = {};
     DataUpNDSc14acylcoad = {};
```

```
DataUpNDSc16acylcoad = {};

tsolStart = tsolScan[Xstart];
SSGuess = Table[{Vars[[i]][t],
    (Vars[[i]][900000000] /. tsolStart)[[1]]}, {i, 1, Length[Vars]}];
SSGuess1 = SSGuess[[All, 1]];
SSGuess2 = SSGuess[[All, 2]];
SSGuess1int = SSGuess1 /. t → 0;
InitialConditionsUD = Thread[SSGuess1int == SSGuess2];

For[X = Xstart, X ≤ Xend,


  tsolScanNDS = NDSolve[Join[Odes /. RateEqs /. CoAMATX /. ParmScan[X],
      InitialConditionsUD], Vars, {t, 0, 1000000000}];
  SSGuess = Table[{Vars[[i]][t], (Vars[[i]][900000000] /. tsolScanNDS)[[1]]},
    {i, 1, Length[Vars]}];
  SSGuess1 = SSGuess[[All, 1]];
  SSGuess2 = SSGuess[[All, 2]];
  SSGuess1int = SSGuess1 /. t → 0;
  InitialConditionsUD = Thread[SSGuess1int == SSGuess2];
  SS = Thread[SSGuess1 → SSGuess2];
  c4acylcoa = SSGuess2[[39]] ;
  c6acylcoa = SSGuess2[[33]] ;
  c8acylcoa = SSGuess2[[27]] ;
  c10acylcoa = SSGuess2[[21]] ;
  c12acylcoa = SSGuess2[[15]] ;
  c14acylcoa = SSGuess2[[9]] ;
  c16acylcoa = SSGuess2[[3]] ;

  c4c6acylcoa = c4acylcoa + c6acylcoa;
  intermediatecoa = SSGuess2[[3]] + SSGuess2[[4]] + SSGuess2[[5]] +
    SSGuess2[[6]] + SSGuess2[[9]] + SSGuess2[[10]] + SSGuess2[[11]] +
    SSGuess2[[12]] + SSGuess2[[15]] + SSGuess2[[16]] + SSGuess2[[17]] +
    SSGuess2[[18]] + SSGuess2[[21]] + SSGuess2[[22]] + SSGuess2[[23]] +
    SSGuess2[[24]] + SSGuess2[[27]] + SSGuess2[[28]] + SSGuess2[[29]] +
    SSGuess2[[30]] + SSGuess2[[33]] + SSGuess2[[34]] + SSGuess2[[35]] +
    SSGuess2[[36]] + SSGuess2[[39]] + SSGuess2[[40]] + SSGuess2[[41]] + X;
  freeCoa = 5000 - intermediatecoa - 70;
  intermediateacylcoa = SSGuess2[[39]] + SSGuess2[[33]] + SSGuess2[[27]] +
    SSGuess2[[21]] + SSGuess2[[15]] + SSGuess2[[9]] + SSGuess2[[3]];

  AppendTo[DataUpNDSflux10md,
   {X, 10^3 vcactC16 /. RateEqs /. CoAMATX /. ParmScan[X] /. SS}];

  AppendTo[DataUpNDSfluxMCKATC4,
   {X, 10^3 vmckatC4 /. RateEqs /. CoAMATX /. ParmScan[X] /. SS}];
  AppendTo[DataUpNDSfluxMSCHADC4,
   {X, 10^3 vmschadC4 /. RateEqs /. CoAMATX /. ParmScan[X] /. SS}];
  AppendTo[DataUpNDSc4acylcoad, {X, c4acylcoa}];
  AppendTo[DataUpNDSc6acylcoad, {X, c6acylcoa}];
  AppendTo[DataUpNDSc8acylcoad, {X, c8acylcoa}];
  AppendTo[DataUpNDSc10acylcoad, {X, c10acylcoa}];
  AppendTo[DataUpNDSc12acylcoad, {X, c12acylcoa}];
```

```
      AppendTo[DataUpNDSc14acylcoad, {X, c14acylcoa}];
      AppendTo[DataUpNDSc16acylcoad, {X, c16acylcoa}];

      AppendTo[DataUpNDSc4c6acylcoad, {X, c4c6acylcoa}];
      AppendTo[DataUpNDScintermedcoad, {X, intermediatecoa}];
      AppendTo[DataUpNDSfreecoad, {X, freeCoa}];
      AppendTo[DataUpNDScintermedacylcoad, {X, intermediateacylcoa}];

      X = X + dX;];
    ], ProgressIndicator[X, {Xstart, Xend}]]
```

In[∘]:=
```
ScanUpNDS[0, 0.01, 10.82]
```

In[∘]:=
```
ListLinePlot[{DataDownNDSfluxMCKATC4, DataDownNDSfluxMSCHADC4}, PlotRange → All,
  PlotStyle → {Blue, Green}, PlotLegends → {"vMCKATC4", "vMSCHADC4"}]
```

Out[∘]=

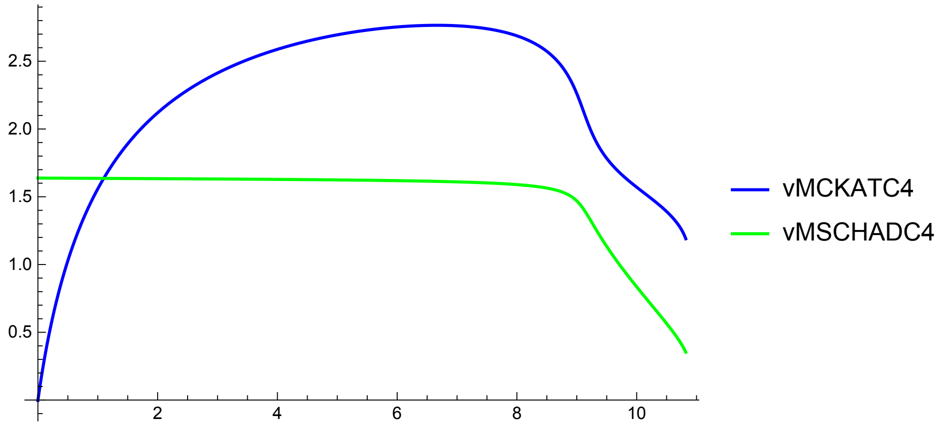

Supplement: S1 Appendix — (ZIP) [file pcbi.1009259.s012.zip › MFAOOriginandCauseofBistability.pdf]
