# Supplementary material for: Bistability in fatty-acid oxidation resulting from substrate inhibition
Source: PLoS Comput Biol. 2021 Aug 12;17(8):e1009259. doi: 10.1371/journal.pcbi.1009259 (PMC8396765; doi:10.1371/journal.pcbi.1009259)
Supplement: S2 Appendix — (ZIP) [file pcbi.1009259.s013.zip › MFAOmodelwithVaryingParametersFin.pdf]

Mitochondrial Fatty Acid Oxidation Kinetic model with Varying Parameters:NAD:NADH ratio, FAD:FADH ratio, Malonyl-CoA concentration, total CoA pool, total NAD and NADH, Vmax of CPT1 and MCKAT.

Definitions of the various functions

In[∘]:= CPT1[sf_, V_, Kms1_, Kms2_, Kmp1_, Kmp2_, Ki1_, Keq_, S1_, S2_, P1_, P2_, I1_, n_] :=

$$\frac{sf*V*\left(\frac{S1*S2}{Kms1*Kms2}-\frac{P1*P2}{Kms1*Kms2*Keq}\right)}{\left(1+\frac{S1}{Kms1}+\frac{P1}{Kmp1}+\left(\frac{I1}{Ki1}\right)^{n}\right)*\left(1+\frac{S2}{Kms2}+\frac{P2}{Kmp2}\right)} \text{ (* } \left(1-\left(\frac{I1^{n}}{I1^{n}+Ki1^{n}}\right)\right) \text{ *+ } \left(\frac{I1}{Ki1}\right)^{n} \text{ *)}$$

In[∘]:= CACT[Vf_, Vr_, Kms1_, Kms2_, Kmp1_, Kmp2_, Kis1_, Kip2_, Keq_, S1_, S2_, P1_, P2_] :=

$$\frac{Vf*\left(S1*S2-\frac{P1*P2}{Keq}\right)}{S1*S2+Kms2*S1+Kms1*S2*\left(1+\frac{P2}{Kip2}\right)+\frac{Vf}{Vr*Keq}*\left(Kmp2*P1*\left(1+\frac{S1}{Kis1}\right)+P2*(Kmp1+P1)\right)}$$

In[∘]:= CPT2[sf_, V_, Kms1_, Kms2_, Kms3_, Kms4_, Kms5_, Kms6_, Kms7_, Kms8_, Kmp1_, Kmp2_, Kmp3_, Kmp4_, Kmp5_, Kmp6_, Kmp7_, Kmp8_, Keq_, S1_, S2_, S3_, S4_, S5_, S6_, S7_, S8_, P1_, P2_, P3_, P4_, P5_, P6_, P7_, P8_] :=

$$\left(sf*V*\left(\frac{S1*S8}{Kms1*Kms8}-\frac{P1*P8}{Kms1*Kms8*Keq}\right)\right)\Big/\left(\left(1+\frac{S1}{Kms1}+\frac{P1}{Kmp1}+\frac{S2}{Kms2}+\frac{P2}{Kmp2}+\frac{S3}{Kms3}+\frac{P3}{Kmp3}+\frac{S4}{Kms4}+\frac{P4}{Kmp4}+\frac{S5}{Kms5}+\frac{P5}{Kmp5}+\frac{S6}{Kms6}+\frac{P6}{Kmp6}+\frac{S7}{Kms7}+\frac{P7}{Kmp7}\right)*\left(1+\frac{S8}{Kms8}+\frac{P8}{Kmp8}\right)\right)$$

In[∘]:= VLCAD[sf_, V_, Kms1_, Kms2_, Kms3_, Kms4_, Kmp1_, Kmp2_, Kmp3_, Kmp4_, Keq_, S1_, S2_, S3_, S4_, P1_, P2_, P3_, P4_] :=

$$\frac{sf*V*\left(\frac{S1*(S4-P4)}{Kms1*Kms4}-\frac{P1*P4}{Kms1*Kms4*Keq}\right)}{\left(1+\frac{S1}{Kms1}+\frac{P1}{Kmp1}+\frac{S2}{Kms2}+\frac{P2}{Kmp2}+\frac{S3}{Kms3}+\frac{P3}{Kmp3}\right)*\left(1+\frac{(S4-P4)}{Kms4}+\frac{P4}{Kmp4}\right)}$$

In[∘]:= LCAD[sf_, V_, Kms1_, Kms2_, Kms3_, Kms4_, Kms5_, Kms6_, Kmp1_, Kmp2_, Kmp3_, Kmp4_, Kmp5_, Kmp6_, Keq_, S1_, S2_, S3_, S4_, S5_, S6_, P1_, P2_, P3_, P4_, P5_, P6_] :=

$$\frac{sf*V*\left(\frac{S1*(S6-P6)}{Kms1*Kms6}-\frac{P1*P6}{Kms1*Kms6*Keq}\right)}{\left(1+\frac{S1}{Kms1}+\frac{P1}{Kmp1}+\frac{S2}{Kms2}+\frac{P2}{Kmp2}+\frac{S3}{Kms3}+\frac{P3}{Kmp3}+\frac{S4}{Kms4}+\frac{P4}{Kmp4}+\frac{S5}{Kms5}+\frac{P5}{Kmp5}\right)*\left(1+\frac{(S6-P6)}{Kms6}+\frac{P6}{Kmp6}\right)}$$

In[ ]:= MCAD[sf_, V_, Kms1_, Kms2_, Kms3_, Kms4_, Kms5_, Kms6_, Kmp1_, Kmp2_, Kmp3_, Kmp4_, Kmp5_, Kmp6_, Keq_, S1_, S2_, S3_, S4_, S5_, S6_, P1_, P2_, P3_, P4_, P5_, P6_] :=

$$\frac{sf*V*\left(\frac{S1*(S6-P6)}{Kms1*Kms6}-\frac{P1*P6}{Kms1*Kms6*Keq}\right)}{\left(1+\frac{S1}{Kms1}+\frac{P1}{Kmp1}+\frac{S2}{Kms2}+\frac{P2}{Kmp2}+\frac{S3}{Kms3}+\frac{P3}{Kmp3}+\frac{S4}{Kms4}+\frac{P4}{Kmp4}+\frac{S5}{Kms5}+\frac{P5}{Kmp5}\right)*\left(1+\frac{(S6-P6)}{Kms6}+\frac{P6}{Kmp6}\right)}$$

In[ ]:= SCAD[sf_, V_, Kms1_, Kms2_, Kms3_, Kmp1_, Kmp2_, Kmp3_, Keq_, S1_, S2_, S3_, P1_, P2_, P3_] :=

$$\frac{sf*V*\left(\frac{S1*(S3-P3)}{Kms1*Kms3}-\frac{P1*P3}{Kms1*Kms3*Keq}\right)}{\left(1+\frac{S1}{Kms1}+\frac{P1}{Kmp1}+\frac{S2}{Kms2}+\frac{P2}{Kmp2}\right)*\left(1+\frac{(S3-P3)}{Kms3}+\frac{P3}{Kmp3}\right)}$$

In[ ]:= CROT[sf_, V_, Kms1_, Kms2_, Kms3_, Kms4_, Kms5_, Kms6_, Kms7_, Kmp1_, Kmp2_, Kmp3_, Kmp4_, Kmp5_, Kmp6_, Kmp7_, Ki1_, Keq_, S1_, S2_, S3_, S4_, S5_, S6_, S7_, P1_, P2_, P3_, P4_, P5_, P6_, P7_, I1_] :=

$$\frac{sf*V*\left(\frac{S1}{Kms1}-\frac{P1}{Kms1*Keq}\right)}{1+\frac{S1}{Kms1}+\frac{P1}{Kmp1}+\frac{S2}{Kms2}+\frac{P2}{Kmp2}+\frac{S3}{Kms3}+\frac{P3}{Kmp3}+\frac{S4}{Kms4}+\frac{P4}{Kmp4}+\frac{S5}{Kms5}+\frac{P5}{Kmp5}+\frac{S6}{Kms6}+\frac{P6}{Kmp6}+\frac{S7}{Kms7}+\frac{P7}{Kmp7}+\frac{I1}{Ki1}}$$

In[ ]:= MSCHAD[sf_, V_, Kms1_, Kms2_, Kms3_, Kms4_, Kms5_, Kms6_, Kms7_, Kms8_, Kmp1_, Kmp2_, Kmp3_, Kmp4_, Kmp5_, Kmp6_, Kmp7_, Kmp8_, Keq_, S1_, S2_, S3_, S4_, S5_, S6_, S7_, S8_, P1_, P2_, P3_, P4_, P5_, P6_, P7_, P8_] :=

$$\left(sf*V*\left(\frac{S1*(S8-P8)}{Kms1*Kms8}-\frac{P1*P8}{Kms1*Kms8*Keq}\right)\right)\Big/\left(\left(1+\frac{S1}{Kms1}+\frac{P1}{Kmp1}+\frac{S2}{Kms2}+\frac{P2}{Kmp2}+\frac{S3}{Kms3}+\frac{P3}{Kmp3}+\frac{S4}{Kms4}+\frac{P4}{Kmp4}+\frac{S5}{Kms5}+\frac{P5}{Kmp5}+\frac{S6}{Kms6}+\frac{P6}{Kmp6}+\frac{S7}{Kms7}+\frac{P7}{Kmp7}\right)*\left(1+\frac{(S8-P8)}{Kms8}+\frac{P8}{Kmp8}\right)\right)$$

In[ ]:= MCKATA[sf_, V_, Kms1_, Kms2_, Kms3_, Kms4_, Kms5_, Kms6_, Kms7_, Kms8_, Kmp1_, Kmp2_, Kmp3_, Kmp4_, Kmp5_, Kmp6_, Kmp7_, Kmp8_, Keq_, S1_, S2_, S3_, S4_, S5_, S6_, S7_, S8_, P1_, P2_, P3_, P4_, P5_, P6_, P7_, P8_] :=

$$\left(sf*V*\left(\frac{S1*S8}{Kms1*Kms8}-\frac{P1*P8}{Kms1*Kms8*Keq}\right)\right)\Big/\left(\left(1+\frac{S1}{Kms1}+\frac{P1}{Kmp1}+\frac{S2}{Kms2}+\frac{P2}{Kmp2}+\frac{S3}{Kms3}+\frac{P3}{Kmp3}+\frac{S4}{Kms4}+\frac{P4}{Kmp4}+\frac{S5}{Kms5}+\frac{P5}{Kmp5}+\frac{S6}{Kms6}+\frac{P6}{Kmp6}+\frac{S7}{Kms7}+\frac{P7}{Kmp7}+\frac{P8}{Kmp8}\right)*\left(1+\frac{S8}{Kms8}+\frac{P8}{Kmp8}\right)\right)$$

In[ ]:= MCKATB[sf_, V_, Kms1_, Kms2_, Kms3_, Kms4_, Kms5_, Kms6_, Kms7_, Kms8_, Kmp1_, Kmp2_, Kmp3_, Kmp4_, Kmp5_, Kmp6_, Kmp7_, Kmp8_, Keq_, S1_, S2_, S3_, S4_, S5_, S6_, S7_, S8_, P1_, P2_, P3_, P4_, P5_, P6_, P7_, P8_] :=

$$\left(sf*V*\left(\frac{S1*S8}{Kms1*Kms8}-\frac{P8*P8}{Kms1*Kms8*Keq}\right)\right)\Big/\left(\left(1+\frac{S1}{Kms1}+\frac{P1}{Kmp1}+\frac{S2}{Kms2}+\frac{P2}{Kmp2}+\frac{S3}{Kms3}+\frac{P3}{Kmp3}+\frac{S4}{Kms4}+\frac{P4}{Kmp4}+\frac{S5}{Kms5}+\frac{P5}{Kmp5}+\frac{S6}{Kms6}+\frac{P6}{Kmp6}+\frac{S7}{Kms7}+\frac{P7}{Kmp7}+\frac{P8}{Kmp8}\right)*\left(1+\frac{S8}{Kms8}+\frac{P8}{Kmp8}\right)\right)$$

In[ ]:=
```
MTP[sf_, V_, Kms1_, Kms2_, Kms3_, Kms4_, Kms5_, Kms7_, Kms8_, Kmp1_,
  Kmp2_, Kmp3_, Kmp4_, Kmp5_, Kmp6_, Kmp7_, Kmp8_, Ki1_, Keq_, S1_, S2_,
  S3_, S4_, S5_, S7_, S8_, P1_, P2_, P3_, P4_, P5_, P6_, P7_, P8_, I1_] :=
 (sf * V * (S1 * (S7 - P7) * S8 / (Kms1 * Kms7 * Kms8) - P1 * P7 * P8 / (Kms1 * Kms7 * Kms8 * Keq))) /
  ((1 + S1/Kms1 + P1/Kmp1 + S2/Kms2 + P2/Kmp2 + S3/Kms3 + P3/Kmp3 + S4/Kms4 + P4/Kmp4 + S5/Kms5 + P5/Kmp5 + P6/Kmp6 + I1/Ki1) *
   (1 + (S7 - P7)/Kms7 + P7/Kmp7) * (1 + S8/Kms8 + P8/Kmp8))
```

In[ ]:=
```
RES[Ks_, S_, K1_] := Ks * (S - K1)
```

Define the differential equations

In[ ]:=
```
Odes = {
  C16AcylCarCYT'[t] == (vcpt1C16 - vcactC16)/VCYT,
  C16AcylCarMAT'[t] == (vcactC16 - vcpt2C16)/VMAT,
  C16AcylCoAMAT'[t] == (vcpt2C16 - vvlcadC16 - vlcadC16)/VMAT,
  C16EnoylCoAMAT'[t] == (vvlcadC16 + vlcadC16 - vcrotC16 - vmtpC16)/VMAT,
  C16HydroxyacylCoAMAT'[t] == (vcrotC16 - vmschadC16)/VMAT,
  C16KetoacylCoAMAT'[t] == (vmschadC16 - vmckatC16)/VMAT,
  C14AcylCarCYT'[t] == (-vcactC14)/VCYT,
  C14AcylCarMAT'[t] == (vcactC14 - vcpt2C14)/VMAT,
  C14AcylCoAMAT'[t] == (vcpt2C14 + vmtpC16 + vmckatC16 - vvlcadC14 - vlcadC14)/VMAT,
  C14EnoylCoAMAT'[t] == (vvlcadC14 + vlcadC14 - vcrotC14 - vmtpC14)/VMAT,
  C14HydroxyacylCoAMAT'[t] == (vcrotC14 - vmschadC14)/VMAT,
  C14KetoacylCoAMAT'[t] == (vmschadC14 - vmckatC14)/VMAT,
  C12AcylCarCYT'[t] == (-vcactC12)/VCYT,
  C12AcylCarMAT'[t] == (vcactC12 - vcpt2C12)/VMAT,
  C12AcylCoAMAT'[t] ==
   (vcpt2C12 + vmtpC14 + vmckatC14 - vvlcadC12 - vlcadC12 - vmcadC12)/VMAT,
  C12EnoylCoAMAT'[t] == (vvlcadC12 + vlcadC12 + vmcadC12 - vcrotC12 - vmtpC12)/VMAT,
```

$$\text{C12HydroxyacylCoAMAT}'[t] == \frac{\text{vcrotC12} - \text{vmschadC12}}{\text{VMAT}},$$

$$\text{C12KetoacylCoAMAT}'[t] == \frac{\text{vmschadC12} - \text{vmckatC12}}{\text{VMAT}},$$

$$\text{C10AcylCarCYT}'[t] == \frac{-\text{vcactC10}}{\text{VCYT}},$$

$$\text{C10AcylCarMAT}'[t] == \frac{\text{vcactC10} - \text{vcpt2C10}}{\text{VMAT}},$$

$$\text{C10AcylCoAMAT}'[t] == \frac{\text{vcpt2C10} + \text{vmtpC12} + \text{vmckatC12} - \text{vlcadC10} - \text{vmcadC10}}{\text{VMAT}},$$

$$\text{C10EnoylCoAMAT}'[t] == \frac{\text{vlcadC10} + \text{vmcadC10} - \text{vcrotC10} - \text{vmtpC10}}{\text{VMAT}},$$

$$\text{C10HydroxyacylCoAMAT}'[t] == \frac{\text{vcrotC10} - \text{vmschadC10}}{\text{VMAT}},$$

$$\text{C10KetoacylCoAMAT}'[t] == \frac{\text{vmschadC10} - \text{vmckatC10}}{\text{VMAT}},$$

$$\text{C8AcylCarCYT}'[t] == \frac{-\text{vcactC8}}{\text{VCYT}},$$

$$\text{C8AcylCarMAT}'[t] == \frac{\text{vcactC8} - \text{vcpt2C8}}{\text{VMAT}},$$

$$\text{C8AcylCoAMAT}'[t] == \frac{\text{vcpt2C8} + \text{vmtpC10} + \text{vmckatC10} - \text{vlcadC8} - \text{vmcadC8}}{\text{VMAT}},$$

$$\text{C8EnoylCoAMAT}'[t] == \frac{\text{vlcadC8} + \text{vmcadC8} - \text{vcrotC8} - \text{vmtpC8}}{\text{VMAT}},$$

$$\text{C8HydroxyacylCoAMAT}'[t] == \frac{\text{vcrotC8} - \text{vmschadC8}}{\text{VMAT}},$$

$$\text{C8KetoacylCoAMAT}'[t] == \frac{\text{vmschadC8} - \text{vmckatC8}}{\text{VMAT}},$$

$$\text{C6AcylCarCYT}'[t] == \frac{-\text{vcactC6}}{\text{VCYT}},$$

$$\text{C6AcylCarMAT}'[t] == \frac{\text{vcactC6} - \text{vcpt2C6}}{\text{VMAT}},$$

$$\text{C6AcylCoAMAT}'[t] == \frac{\text{vcpt2C6} + \text{vmtpC8} + \text{vmckatC8} - \text{vmcadC6} - \text{vscadC6}}{\text{VMAT}},$$

$$\text{C6EnoylCoAMAT}'[t] == \frac{\text{vmcadC6} + \text{vscadC6} - \text{vcrotC6}}{\text{VMAT}},$$

$$\text{C6HydroxyacylCoAMAT}'[t] == \frac{\text{vcrotC6} - \text{vmschadC6}}{\text{VMAT}},$$

$$\text{C6KetoacylCoAMAT}'[t] == \frac{\text{vmschadC6} - \text{vmckatC6}}{\text{VMAT}},$$

$$\text{C4AcylCarCYT}'[t] == \frac{-\text{vcactC4}}{\text{VCYT}},$$

$$\text{C4AcylCarMAT}'[t] == \frac{\text{vcactC4} - \text{vcpt2C4}}{\text{VMAT}},$$

$$\text{C4AcylCoAMAT}'[t] == \frac{\text{vcpt2C4} + \text{vmckatC6} - \text{vmcadC4} - \text{vscadC4}}{\text{VMAT}},$$

```
C4EnoylCoAMAT'[t] == (vmcadC4 + vscadC4 - vcrotC4)/VMAT,
C4HydroxyacylCoAMAT'[t] == (vcrotC4 - vmschadC4)/VMAT,
C4AcetoacylCoAMAT'[t] == (vmschadC4 - vmckatC4)/VMAT};

RateEqs = {vcpt1C16 → CPT1[sfcpt1C16, Vcpt1, Kmcpt1C16AcylCoACYT,
     Kmcpt1CarCYT, Kmcpt1C16AcylCarCYT, Kmcpt1CoACYT, Kicpt1MalCoACYT, Keqcpt1,
     C16AcylCoACYT, CarCYT, C16AcylCarCYT[t], CoACYT, MalCoACYT, ncpt1],
   vcactC16 → CACT[Vfcact, Vrcact, KmcactC16AcylCarCYT, KmcactCarMAT,
     KmcactC16AcylCarMAT, KmcactCarCYT, KicactC16AcylCarCYT, KicactCarCYT,
     Keqcact, C16AcylCarCYT[t], CarMAT, C16AcylCarMAT[t], CarCYT],
   vcactC14 → CACT[Vfcact, Vrcact, KmcactC14AcylCarCYT, KmcactCarMAT,
     KmcactC14AcylCarMAT, KmcactCarCYT, KicactC14AcylCarCYT, KicactCarCYT,
     Keqcact, C14AcylCarCYT[t], CarMAT, C14AcylCarMAT[t], CarCYT],
   vcactC12 → CACT[Vfcact, Vrcact, KmcactC12AcylCarCYT, KmcactCarMAT,
     KmcactC12AcylCarMAT, KmcactCarCYT, KicactC12AcylCarCYT, KicactCarCYT,
     Keqcact, C12AcylCarCYT[t], CarMAT, C12AcylCarMAT[t], CarCYT],
   vcactC10 → CACT[Vfcact, Vrcact, KmcactC10AcylCarCYT, KmcactCarMAT,
     KmcactC10AcylCarMAT, KmcactCarCYT, KicactC10AcylCarCYT, KicactCarCYT,
     Keqcact, C10AcylCarCYT[t], CarMAT, C10AcylCarMAT[t], CarCYT],
   vcactC8 → CACT[Vfcact, Vrcact, KmcactC8AcylCarCYT, KmcactCarMAT,
     KmcactC8AcylCarMAT, KmcactCarCYT, KicactC8AcylCarCYT, KicactCarCYT,
     Keqcact, C8AcylCarCYT[t], CarMAT, C8AcylCarMAT[t], CarCYT],
   vcactC6 → CACT[Vfcact, Vrcact, KmcactC6AcylCarCYT, KmcactCarMAT,
     KmcactC6AcylCarMAT, KmcactCarCYT, KicactC6AcylCarCYT, KicactCarCYT,
     Keqcact, C6AcylCarCYT[t], CarMAT, C6AcylCarMAT[t], CarCYT],
   vcactC4 → CACT[Vfcact, Vrcact, KmcactC4AcylCarCYT, KmcactCarMAT,
     KmcactC4AcylCarMAT, KmcactCarCYT, KicactC4AcylCarCYT, KicactCarCYT,
     Keqcact, C4AcylCarCYT[t], CarMAT, C4AcylCarMAT[t], CarCYT],
   vcpt2C16 → CPT2[sfcpt2C16, Vcpt2, Kmcpt2C16AcylCarMAT, Kmcpt2C14AcylCarMAT,
     Kmcpt2C12AcylCarMAT, Kmcpt2C10AcylCarMAT, Kmcpt2C8AcylCarMAT, Kmcpt2C6AcylCarMAT,
     Kmcpt2C4AcylCarMAT, Kmcpt2CoAMAT, Kmcpt2C16AcylCoAMAT, Kmcpt2C14AcylCoAMAT,
     Kmcpt2C12AcylCoAMAT, Kmcpt2C10AcylCoAMAT, Kmcpt2C8AcylCoAMAT, Kmcpt2C6AcylCoAMAT,
     Kmcpt2C4AcylCoAMAT, Kmcpt2CarMAT, Keqcpt2, C16AcylCarMAT[t], C14AcylCarMAT[t],
     C12AcylCarMAT[t], C10AcylCarMAT[t], C8AcylCarMAT[t], C6AcylCarMAT[t],
     C4AcylCarMAT[t], CoAMAT, C16AcylCoAMAT[t], C14AcylCoAMAT[t], C12AcylCoAMAT[t],
     C10AcylCoAMAT[t], C8AcylCoAMAT[t], C6AcylCoAMAT[t], C4AcylCoAMAT[t], CarMAT],
   vcpt2C14 → CPT2[sfcpt2C14, Vcpt2, Kmcpt2C14AcylCarMAT, Kmcpt2C16AcylCarMAT,
     Kmcpt2C12AcylCarMAT, Kmcpt2C10AcylCarMAT, Kmcpt2C8AcylCarMAT, Kmcpt2C6AcylCarMAT,
     Kmcpt2C4AcylCoAMAT, Kmcpt2CoAMAT, Kmcpt2C14AcylCoAMAT, Kmcpt2C16AcylCoAMAT,
     Kmcpt2C12AcylCoAMAT, Kmcpt2C10AcylCoAMAT, Kmcpt2C8AcylCoAMAT, Kmcpt2C6AcylCoAMAT,
     Kmcpt2C4AcylCoAMAT, Kmcpt2CarMAT, Keqcpt2, C14AcylCarMAT[t], C16AcylCarMAT[t],
     C12AcylCarMAT[t], C10AcylCarMAT[t], C8AcylCarMAT[t], C6AcylCarMAT[t],
     C4AcylCarMAT[t], CoAMAT, C14AcylCoAMAT[t], C16AcylCoAMAT[t], C12AcylCoAMAT[t],
     C10AcylCoAMAT[t], C8AcylCoAMAT[t], C6AcylCoAMAT[t], C4AcylCoAMAT[t], CarMAT],
   vcpt2C12 → CPT2[sfcpt2C12, Vcpt2, Kmcpt2C12AcylCarMAT, Kmcpt2C16AcylCarMAT,
     Kmcpt2C14AcylCarMAT, Kmcpt2C10AcylCarMAT, Kmcpt2C8AcylCarMAT, Kmcpt2C6AcylCarMAT,
     Kmcpt2C4AcylCarMAT, Kmcpt2CoAMAT, Kmcpt2C12AcylCoAMAT, Kmcpt2C16AcylCoAMAT,
     Kmcpt2C14AcylCoAMAT, Kmcpt2C10AcylCoAMAT, Kmcpt2C8AcylCoAMAT, Kmcpt2C6AcylCoAMAT,
     Kmcpt2C4AcylCoAMAT, Kmcpt2CarMAT, Keqcpt2, C12AcylCarMAT[t], C16AcylCarMAT[t],
     C14AcylCarMAT[t], C10AcylCarMAT[t], C8AcylCarMAT[t], C6AcylCarMAT[t],
     C4AcylCarMAT[t], CoAMAT, C12AcylCoAMAT[t], C16AcylCoAMAT[t], C14AcylCoAMAT[t],
```

```
    C10AcylCoAMAT[t], C8AcylCoAMAT[t], C6AcylCoAMAT[t], C4AcylCoAMAT[t], CarMAT],
  vcpt2C10 → CPT2[sfcpt2C10, Vcpt2, Kmcpt2C10AcylCarMAT, Kmcpt2C16AcylCarMAT,
    Kmcpt2C14AcylCarMAT, Kmcpt2C12AcylCarMAT, Kmcpt2C8AcylCarMAT, Kmcpt2C6AcylCarMAT,
    Kmcpt2C4AcylCarMAT, Kmcpt2CoAMAT, Kmcpt2C10AcylCoAMAT, Kmcpt2C16AcylCoAMAT,
    Kmcpt2C14AcylCoAMAT, Kmcpt2C12AcylCoAMAT, Kmcpt2C8AcylCoAMAT, Kmcpt2C6AcylCoAMAT,
    Kmcpt2C4AcylCoAMAT, Kmcpt2CarMAT, Keqcpt2, C10AcylCarMAT[t], C16AcylCarMAT[t],
    C14AcylCarMAT[t], C12AcylCarMAT[t], C8AcylCarMAT[t], C6AcylCarMAT[t],
    C4AcylCarMAT[t], CoAMAT, C10AcylCoAMAT[t], C16AcylCoAMAT[t], C14AcylCoAMAT[t],
    C12AcylCoAMAT[t], C8AcylCoAMAT[t], C6AcylCoAMAT[t], C4AcylCoAMAT[t], CarMAT],
  vcpt2C8 → CPT2[sfcpt2C8, Vcpt2, Kmcpt2C8AcylCarMAT, Kmcpt2C16AcylCarMAT,
    Kmcpt2C14AcylCarMAT, Kmcpt2C12AcylCarMAT, Kmcpt2C10AcylCarMAT, Kmcpt2C6AcylCarMAT,
    Kmcpt2C4AcylCarMAT, Kmcpt2CoAMAT, Kmcpt2C8AcylCoAMAT, Kmcpt2C16AcylCoAMAT,
    Kmcpt2C14AcylCoAMAT, Kmcpt2C12AcylCoAMAT, Kmcpt2C10AcylCoAMAT, Kmcpt2C6AcylCoAMAT,
    Kmcpt2C4AcylCoAMAT, Kmcpt2CarMAT, Keqcpt2, C8AcylCarMAT[t], C16AcylCarMAT[t],
    C14AcylCarMAT[t], C12AcylCarMAT[t], C10AcylCarMAT[t], C6AcylCarMAT[t],
    C4AcylCarMAT[t], CoAMAT, C8AcylCoAMAT[t], C16AcylCoAMAT[t], C14AcylCoAMAT[t],
    C12AcylCoAMAT[t], C10AcylCoAMAT[t], C6AcylCoAMAT[t], C4AcylCoAMAT[t], CarMAT],
  vcpt2C6 → CPT2[sfcpt2C6, Vcpt2, Kmcpt2C6AcylCarMAT, Kmcpt2C16AcylCarMAT,
    Kmcpt2C14AcylCarMAT, Kmcpt2C12AcylCarMAT, Kmcpt2C10AcylCarMAT, Kmcpt2C8AcylCarMAT,
    Kmcpt2C4AcylCarMAT, Kmcpt2CoAMAT, Kmcpt2C6AcylCoAMAT, Kmcpt2C16AcylCoAMAT,
    Kmcpt2C14AcylCoAMAT, Kmcpt2C12AcylCoAMAT, Kmcpt2C10AcylCoAMAT, Kmcpt2C8AcylCoAMAT,
    Kmcpt2C4AcylCoAMAT, Kmcpt2CarMAT, Keqcpt2, C6AcylCarMAT[t], C16AcylCarMAT[t],
    C14AcylCarMAT[t], C12AcylCarMAT[t], C10AcylCarMAT[t], C8AcylCarMAT[t],
    C4AcylCarMAT[t], CoAMAT, C6AcylCoAMAT[t], C16AcylCoAMAT[t], C14AcylCoAMAT[t],
    C12AcylCoAMAT[t], C10AcylCoAMAT[t], C8AcylCoAMAT[t], C4AcylCoAMAT[t], CarMAT],
  vcpt2C4 → CPT2[sfcpt2C4, Vcpt2, Kmcpt2C4AcylCarMAT, Kmcpt2C16AcylCarMAT,
    Kmcpt2C14AcylCarMAT, Kmcpt2C12AcylCarMAT, Kmcpt2C10AcylCarMAT, Kmcpt2C8AcylCarMAT,
    Kmcpt2C6AcylCarMAT, Kmcpt2CoAMAT, Kmcpt2C4AcylCoAMAT, Kmcpt2C16AcylCoAMAT,
    Kmcpt2C14AcylCoAMAT, Kmcpt2C12AcylCoAMAT, Kmcpt2C10AcylCoAMAT, Kmcpt2C8AcylCoAMAT,
    Kmcpt2C6AcylCoAMAT, Kmcpt2CarMAT, Keqcpt2, C4AcylCarMAT[t], C16AcylCarMAT[t],
    C14AcylCarMAT[t], C12AcylCarMAT[t], C10AcylCarMAT[t], C8AcylCarMAT[t],
    C6AcylCarMAT[t], CoAMAT, C4AcylCoAMAT[t], C16AcylCoAMAT[t], C14AcylCoAMAT[t],
    C12AcylCoAMAT[t], C10AcylCoAMAT[t], C8AcylCoAMAT[t], C6AcylCoAMAT[t], CarMAT],
  vvlcadC16 → VLCAD[sfvlcadC16, Vvlcad, KmvlcadC16AcylCoAMAT, KmvlcadC14AcylCoAMAT,
    KmvlcadC12AcylCoAMAT, KmvlcadFAD, KmvlcadC16EnoylCoAMAT,
    KmvlcadC14EnoylCoAMAT, KmvlcadC12EnoylCoAMAT, KmvlcadFADH, Keqvlcad,
    C16AcylCoAMAT[t], C14AcylCoAMAT[t], C12AcylCoAMAT[t], FADtMAT,
    C16EnoylCoAMAT[t], C14EnoylCoAMAT[t], C12EnoylCoAMAT[t], FADHMAT],
  vvlcadC14 → VLCAD[sfvlcadC14, Vvlcad, KmvlcadC14AcylCoAMAT, KmvlcadC16AcylCoAMAT,
    KmvlcadC12AcylCoAMAT, KmvlcadFAD, KmvlcadC14EnoylCoAMAT,
    KmvlcadC16EnoylCoAMAT, KmvlcadC12EnoylCoAMAT, KmvlcadFADH, Keqvlcad,
    C14AcylCoAMAT[t], C16AcylCoAMAT[t], C12AcylCoAMAT[t], FADtMAT,
    C14EnoylCoAMAT[t], C16EnoylCoAMAT[t], C12EnoylCoAMAT[t], FADHMAT],
  vvlcadC12 → VLCAD[sfvlcadC12, Vvlcad, KmvlcadC12AcylCoAMAT, KmvlcadC16AcylCoAMAT,
    KmvlcadC14AcylCoAMAT, KmvlcadFAD, KmvlcadC12EnoylCoAMAT,
    KmvlcadC16EnoylCoAMAT, KmvlcadC14EnoylCoAMAT, KmvlcadFADH, Keqvlcad,
    C12AcylCoAMAT[t], C16AcylCoAMAT[t], C14AcylCoAMAT[t], FADtMAT,
    C12EnoylCoAMAT[t], C16EnoylCoAMAT[t], C14EnoylCoAMAT[t], FADHMAT],
  vlcadC16 → LCAD[sflcadC16, Vlcad, KmlcadC16AcylCoAMAT, KmlcadC14AcylCoAMAT,
    KmlcadC12AcylCoAMAT, KmlcadC10AcylCoAMAT, KmlcadC8AcylCoAMAT, KmlcadFAD,
    KmlcadC16EnoylCoAMAT, KmlcadC14EnoylCoAMAT, KmlcadC12EnoylCoAMAT,
    KmlcadC10EnoylCoAMAT, KmlcadC8EnoylCoAMAT, KmlcadFADH, Keqlcad,
    C16AcylCoAMAT[t], C14AcylCoAMAT[t], C12AcylCoAMAT[t], C10AcylCoAMAT[t],
    C8AcylCoAMAT[t], FADtMAT, C16EnoylCoAMAT[t], C14EnoylCoAMAT[t],
    C12EnoylCoAMAT[t], C10EnoylCoAMAT[t], C8EnoylCoAMAT[t], FADHMAT],
```

```
vlcadC14 → LCAD[sflcadC14, Vlcad, KmlcadC14AcylCoAMAT, KmlcadC16AcylCoAMAT,
   KmlcadC12AcylCoAMAT, KmlcadC10AcylCoAMAT, KmlcadC8AcylCoAMAT, KmlcadFAD,
   KmlcadC14EnoylCoAMAT, KmlcadC16EnoylCoAMAT, KmlcadC12EnoylCoAMAT,
   KmlcadC10EnoylCoAMAT, KmlcadC8EnoylCoAMAT, KmlcadFADH, Keqlcad,
   C14AcylCoAMAT[t], C16AcylCoAMAT[t], C12AcylCoAMAT[t], C10AcylCoAMAT[t],
   C8AcylCoAMAT[t], FADtMAT, C14EnoylCoAMAT[t], C16EnoylCoAMAT[t],
   C12EnoylCoAMAT[t], C10EnoylCoAMAT[t], C8EnoylCoAMAT[t], FADHMAT],
vlcadC12 → LCAD[sflcadC12, Vlcad, KmlcadC12AcylCoAMAT, KmlcadC16AcylCoAMAT,
   KmlcadC14AcylCoAMAT, KmlcadC10AcylCoAMAT, KmlcadC8AcylCoAMAT, KmlcadFAD,
   KmlcadC12EnoylCoAMAT, KmlcadC16EnoylCoAMAT, KmlcadC14EnoylCoAMAT,
   KmlcadC10EnoylCoAMAT, KmlcadC8EnoylCoAMAT, KmlcadFADH, Keqlcad,
   C12AcylCoAMAT[t], C16AcylCoAMAT[t], C14AcylCoAMAT[t], C10AcylCoAMAT[t],
   C8AcylCoAMAT[t], FADtMAT, C14EnoylCoAMAT[t], C16EnoylCoAMAT[t],
   C14EnoylCoAMAT[t], C10EnoylCoAMAT[t], C8EnoylCoAMAT[t], FADHMAT],
vlcadC10 → LCAD[sflcadC10, Vlcad, KmlcadC10AcylCoAMAT, KmlcadC16AcylCoAMAT,
   KmlcadC14AcylCoAMAT, KmlcadC12AcylCoAMAT, KmlcadC8AcylCoAMAT, KmlcadFAD,
   KmlcadC10EnoylCoAMAT, KmlcadC16EnoylCoAMAT, KmlcadC14EnoylCoAMAT,
   KmlcadC12EnoylCoAMAT, KmlcadC8EnoylCoAMAT, KmlcadFADH, Keqlcad,
   C10AcylCoAMAT[t], C16AcylCoAMAT[t], C14AcylCoAMAT[t], C12AcylCoAMAT[t],
   C8AcylCoAMAT[t], FADtMAT, C10EnoylCoAMAT[t], C16EnoylCoAMAT[t],
   C14EnoylCoAMAT[t], C12EnoylCoAMAT[t], C8EnoylCoAMAT[t], FADHMAT],
vlcadC8 → LCAD[sflcadC8, Vlcad, KmlcadC8AcylCoAMAT, KmlcadC16AcylCoAMAT,
   KmlcadC14AcylCoAMAT, KmlcadC12AcylCoAMAT, KmlcadC10AcylCoAMAT, KmlcadFAD,
   KmlcadC8EnoylCoAMAT, KmlcadC16EnoylCoAMAT, KmlcadC14EnoylCoAMAT,
   KmlcadC12EnoylCoAMAT, KmlcadC10EnoylCoAMAT, KmlcadFADH, Keqlcad,
   C8AcylCoAMAT[t], C16AcylCoAMAT[t], C14AcylCoAMAT[t], C12AcylCoAMAT[t],
   C10AcylCoAMAT[t], FADtMAT, C8EnoylCoAMAT[t], C16EnoylCoAMAT[t],
   C14EnoylCoAMAT[t], C12EnoylCoAMAT[t], C10EnoylCoAMAT[t], FADHMAT],
vmcadC12 → MCAD[sfmcadC12, Vmcad, KmmcadC12AcylCoAMAT, KmmcadC10AcylCoAMAT,
   KmmcadC8AcylCoAMAT, KmmcadC6AcylCoAMAT, KmmcadC4AcylCoAMAT, KmmcadFAD,
   KmmcadC12EnoylCoAMAT, KmmcadC10EnoylCoAMAT, KmmcadC8EnoylCoAMAT,
   KmmcadC6EnoylCoAMAT, KmmcadC4EnoylCoAMAT, KmmcadFADH, Keqmcad,
   C12AcylCoAMAT[t], C10AcylCoAMAT[t], C8AcylCoAMAT[t], C6AcylCoAMAT[t],
   C4AcylCoAMAT[t], FADtMAT, C12EnoylCoAMAT[t], C10EnoylCoAMAT[t],
   C8EnoylCoAMAT[t], C6EnoylCoAMAT[t], C4EnoylCoAMAT[t], FADHMAT],
vmcadC10 → MCAD[sfmcadC10, Vmcad, KmmcadC10AcylCoAMAT, KmmcadC12AcylCoAMAT,
   KmmcadC8AcylCoAMAT, KmmcadC6AcylCoAMAT, KmmcadC4AcylCoAMAT, KmmcadFAD,
   KmmcadC10EnoylCoAMAT, KmmcadC12EnoylCoAMAT, KmmcadC8EnoylCoAMAT,
   KmmcadC6EnoylCoAMAT, KmmcadC4EnoylCoAMAT, KmmcadFADH, Keqmcad,
   C10AcylCoAMAT[t], C12AcylCoAMAT[t], C8AcylCoAMAT[t], C6AcylCoAMAT[t],
   C4AcylCoAMAT[t], FADtMAT, C10EnoylCoAMAT[t], C12EnoylCoAMAT[t],
   C8EnoylCoAMAT[t], C6EnoylCoAMAT[t], C4EnoylCoAMAT[t], FADHMAT],
vmcadC8 → MCAD[sfmcadC8, Vmcad, KmmcadC8AcylCoAMAT, KmmcadC12AcylCoAMAT,
   KmmcadC10AcylCoAMAT, KmmcadC6AcylCoAMAT, KmmcadC4AcylCoAMAT, KmmcadFAD,
   KmmcadC8EnoylCoAMAT, KmmcadC12EnoylCoAMAT, KmmcadC10EnoylCoAMAT,
   KmmcadC6EnoylCoAMAT, KmmcadC4EnoylCoAMAT, KmmcadFADH, Keqmcad,
   C8AcylCoAMAT[t], C12AcylCoAMAT[t], C10AcylCoAMAT[t], C6AcylCoAMAT[t],
   C4AcylCoAMAT[t], FADtMAT, C8EnoylCoAMAT[t], C12EnoylCoAMAT[t],
   C10EnoylCoAMAT[t], C6EnoylCoAMAT[t], C4EnoylCoAMAT[t], FADHMAT],
vmcadC6 → MCAD[sfmcadC6, Vmcad, KmmcadC6AcylCoAMAT, KmmcadC12AcylCoAMAT,
   KmmcadC10AcylCoAMAT, KmmcadC8AcylCoAMAT, KmmcadC4AcylCoAMAT, KmmcadFAD,
   KmmcadC6EnoylCoAMAT, KmmcadC12EnoylCoAMAT, KmmcadC10EnoylCoAMAT,
   KmmcadC8EnoylCoAMAT, KmmcadC4EnoylCoAMAT, KmmcadFADH, Keqmcad,
   C6AcylCoAMAT[t], C12AcylCoAMAT[t], C10AcylCoAMAT[t], C8AcylCoAMAT[t],
   C4AcylCoAMAT[t], FADtMAT, C6EnoylCoAMAT[t], C12EnoylCoAMAT[t],
```

```
    C10EnoylCoAMAT[t], C8EnoylCoAMAT[t], C4EnoylCoAMAT[t], FADHMAT],
  vmcadC4 → MCAD[sfmcadC4, Vmcad, KmmcadC4AcylCoAMAT, KmmcadC12AcylCoAMAT,
    KmmcadC10AcylCoAMAT, KmmcadC8AcylCoAMAT, KmmcadC6AcylCoAMAT, KmmcadFAD,
    KmmcadC4EnoylCoAMAT, KmmcadC12EnoylCoAMAT, KmmcadC10EnoylCoAMAT,
    KmmcadC8EnoylCoAMAT, KmmcadC6EnoylCoAMAT, KmmcadFADH, Keqmcad,
    C4AcylCoAMAT[t], C12AcylCoAMAT[t], C10AcylCoAMAT[t], C8AcylCoAMAT[t],
    C6AcylCoAMAT[t], FADtMAT, C4EnoylCoAMAT[t], C12EnoylCoAMAT[t],
    C10EnoylCoAMAT[t], C8EnoylCoAMAT[t], C6EnoylCoAMAT[t], FADHMAT],
  vscadC6 → SCAD[sfscadC6, Vscad, KmscadC6AcylCoAMAT, KmscadC4AcylCoAMAT, KmscadFAD,
    KmscadC6EnoylCoAMAT, KmscadC4EnoylCoAMAT, KmscadFADH, Keqscad, C6AcylCoAMAT[t],
    C4AcylCoAMAT[t], FADtMAT, C6EnoylCoAMAT[t], C4EnoylCoAMAT[t], FADHMAT],
  vscadC4 → SCAD[sfscadC4, Vscad, KmscadC4AcylCoAMAT, KmscadC6AcylCoAMAT, KmscadFAD,
    KmscadC4EnoylCoAMAT, KmscadC6EnoylCoAMAT, KmscadFADH, Keqscad, C4AcylCoAMAT[t],
    C6AcylCoAMAT[t], FADtMAT, C4EnoylCoAMAT[t], C6EnoylCoAMAT[t], FADHMAT],
  vcrotC16 → CROT[sfcrotC16, Vcrot, KmcrotC16EnoylCoAMAT, KmcrotC14EnoylCoAMAT,
    KmcrotC12EnoylCoAMAT, KmcrotC10EnoylCoAMAT, KmcrotC8EnoylCoAMAT,
    KmcrotC6EnoylCoAMAT, KmcrotC4EnoylCoAMAT, KmcrotC16HydroxyacylCoAMAT,
    KmcrotC14HydroxyacylCoAMAT, KmcrotC12HydroxyacylCoAMAT,
    KmcrotC10HydroxyacylCoAMAT, KmcrotC8HydroxyacylCoAMAT,
    KmcrotC6HydroxyacylCoAMAT, KmcrotC4HydroxyacylCoAMAT, KicrotC4AcetoacylCoA,
    Keqcrot, C16EnoylCoAMAT[t], C14EnoylCoAMAT[t], C12EnoylCoAMAT[t],
    C10EnoylCoAMAT[t], C8EnoylCoAMAT[t], C6EnoylCoAMAT[t],
    C4EnoylCoAMAT[t], C16HydroxyacylCoAMAT[t], C14HydroxyacylCoAMAT[t],
    C12HydroxyacylCoAMAT[t], C10HydroxyacylCoAMAT[t], C8HydroxyacylCoAMAT[t],
    C6HydroxyacylCoAMAT[t], C4HydroxyacylCoAMAT[t], C4AcetoacylCoAMAT[t]],
  vcrotC14 → CROT[sfcrotC14, Vcrot, KmcrotC14EnoylCoAMAT, KmcrotC16EnoylCoAMAT,
    KmcrotC12EnoylCoAMAT, KmcrotC10EnoylCoAMAT, KmcrotC8EnoylCoAMAT,
    KmcrotC6EnoylCoAMAT, KmcrotC4EnoylCoAMAT, KmcrotC14HydroxyacylCoAMAT,
    KmcrotC16HydroxyacylCoAMAT, KmcrotC12HydroxyacylCoAMAT,
    KmcrotC10HydroxyacylCoAMAT, KmcrotC8HydroxyacylCoAMAT,
    KmcrotC6HydroxyacylCoAMAT, KmcrotC4HydroxyacylCoAMAT, KicrotC4AcetoacylCoA,
    Keqcrot, C14EnoylCoAMAT[t], C16EnoylCoAMAT[t], C12EnoylCoAMAT[t],
    C10EnoylCoAMAT[t], C8EnoylCoAMAT[t], C6EnoylCoAMAT[t],
    C4EnoylCoAMAT[t], C14HydroxyacylCoAMAT[t], C16HydroxyacylCoAMAT[t],
    C12HydroxyacylCoAMAT[t], C10HydroxyacylCoAMAT[t], C8HydroxyacylCoAMAT[t],
    C6HydroxyacylCoAMAT[t], C4HydroxyacylCoAMAT[t], C4AcetoacylCoAMAT[t]],
  vcrotC12 → CROT[sfcrotC12, Vcrot, KmcrotC12EnoylCoAMAT, KmcrotC16EnoylCoAMAT,
    KmcrotC14EnoylCoAMAT, KmcrotC10EnoylCoAMAT, KmcrotC8EnoylCoAMAT,
    KmcrotC6EnoylCoAMAT, KmcrotC4EnoylCoAMAT, KmcrotC12HydroxyacylCoAMAT,
    KmcrotC16HydroxyacylCoAMAT, KmcrotC14HydroxyacylCoAMAT,
    KmcrotC10HydroxyacylCoAMAT, KmcrotC8HydroxyacylCoAMAT,
    KmcrotC6HydroxyacylCoAMAT, KmcrotC4HydroxyacylCoAMAT, KicrotC4AcetoacylCoA,
    Keqcrot, C12EnoylCoAMAT[t], C16EnoylCoAMAT[t], C14EnoylCoAMAT[t],
    C10EnoylCoAMAT[t], C8EnoylCoAMAT[t], C6EnoylCoAMAT[t],
    C4EnoylCoAMAT[t], C12HydroxyacylCoAMAT[t], C16HydroxyacylCoAMAT[t],
    C14HydroxyacylCoAMAT[t], C10HydroxyacylCoAMAT[t], C8HydroxyacylCoAMAT[t],
    C6HydroxyacylCoAMAT[t], C4HydroxyacylCoAMAT[t], C4AcetoacylCoAMAT[t]],
  vcrotC10 → CROT[sfcrotC10, Vcrot, KmcrotC10EnoylCoAMAT, KmcrotC16EnoylCoAMAT,
    KmcrotC14EnoylCoAMAT, KmcrotC12EnoylCoAMAT, KmcrotC8EnoylCoAMAT,
    KmcrotC6EnoylCoAMAT, KmcrotC4EnoylCoAMAT, KmcrotC10HydroxyacylCoAMAT,
    KmcrotC16HydroxyacylCoAMAT, KmcrotC14HydroxyacylCoAMAT,
    KmcrotC12HydroxyacylCoAMAT, KmcrotC8HydroxyacylCoAMAT,
    KmcrotC6HydroxyacylCoAMAT, KmcrotC4HydroxyacylCoAMAT, KicrotC4AcetoacylCoA,
    Keqcrot, C10EnoylCoAMAT[t], C16EnoylCoAMAT[t], C14EnoylCoAMAT[t],
    C12EnoylCoAMAT[t], C8EnoylCoAMAT[t], C6EnoylCoAMAT[t],
```

```
    C4EnoylCoAMAT[t], C10HydroxyacylCoAMAT[t], C16HydroxyacylCoAMAT[t],
    C14HydroxyacylCoAMAT[t], C12HydroxyacylCoAMAT[t], C8HydroxyacylCoAMAT[t],
    C6HydroxyacylCoAMAT[t], C4HydroxyacylCoAMAT[t], C4AcetoacylCoAMAT[t]],
  vcrotC8 → CROT[sfcrotC8, Vcrot, KmcrotC8EnoylCoAMAT, KmcrotC16EnoylCoAMAT,
    KmcrotC14EnoylCoAMAT, KmcrotC12EnoylCoAMAT, KmcrotC10EnoylCoAMAT,
    KmcrotC6EnoylCoAMAT, KmcrotC4EnoylCoAMAT, KmcrotC8HydroxyacylCoAMAT,
    KmcrotC16HydroxyacylCoAMAT, KmcrotC14HydroxyacylCoAMAT,
    KmcrotC12HydroxyacylCoAMAT, KmcrotC10HydroxyacylCoAMAT,
    KmcrotC6HydroxyacylCoAMAT, KmcrotC4HydroxyacylCoAMAT, KicrotC4AcetoacylCoA,
    Keqcrot, C8EnoylCoAMAT[t], C16EnoylCoAMAT[t], C14EnoylCoAMAT[t],
    C12EnoylCoAMAT[t], C10EnoylCoAMAT[t], C6EnoylCoAMAT[t],
    C4EnoylCoAMAT[t], C8HydroxyacylCoAMAT[t], C16HydroxyacylCoAMAT[t],
    C14HydroxyacylCoAMAT[t], C12HydroxyacylCoAMAT[t], C10HydroxyacylCoAMAT[t],
    C6HydroxyacylCoAMAT[t], C4HydroxyacylCoAMAT[t], C4AcetoacylCoAMAT[t]],
  vcrotC6 → CROT[sfcrotC6, Vcrot, KmcrotC6EnoylCoAMAT, KmcrotC16EnoylCoAMAT,
    KmcrotC14EnoylCoAMAT, KmcrotC12EnoylCoAMAT, KmcrotC10EnoylCoAMAT,
    KmcrotC8EnoylCoAMAT, KmcrotC4EnoylCoAMAT, KmcrotC6HydroxyacylCoAMAT,
    KmcrotC16HydroxyacylCoAMAT, KmcrotC14HydroxyacylCoAMAT,
    KmcrotC12HydroxyacylCoAMAT, KmcrotC10HydroxyacylCoAMAT,
    KmcrotC8HydroxyacylCoAMAT, KmcrotC4HydroxyacylCoAMAT, KicrotC4AcetoacylCoA,
    Keqcrot, C6EnoylCoAMAT[t], C16EnoylCoAMAT[t], C14EnoylCoAMAT[t],
    C12EnoylCoAMAT[t], C10EnoylCoAMAT[t], C8EnoylCoAMAT[t],
    C4EnoylCoAMAT[t], C6HydroxyacylCoAMAT[t], C16HydroxyacylCoAMAT[t],
    C14HydroxyacylCoAMAT[t], C12HydroxyacylCoAMAT[t], C10HydroxyacylCoAMAT[t],
    C8HydroxyacylCoAMAT[t], C4HydroxyacylCoAMAT[t], C4AcetoacylCoAMAT[t]],
  vcrotC4 → CROT[sfcrotC4, Vcrot, KmcrotC4EnoylCoAMAT, KmcrotC16EnoylCoAMAT,
    KmcrotC14EnoylCoAMAT, KmcrotC12EnoylCoAMAT, KmcrotC10EnoylCoAMAT,
    KmcrotC8EnoylCoAMAT, KmcrotC6EnoylCoAMAT, KmcrotC4HydroxyacylCoAMAT,
    KmcrotC16HydroxyacylCoAMAT, KmcrotC14HydroxyacylCoAMAT,
    KmcrotC12HydroxyacylCoAMAT, KmcrotC10HydroxyacylCoAMAT,
    KmcrotC8HydroxyacylCoAMAT, KmcrotC6HydroxyacylCoAMAT, KicrotC4AcetoacylCoA,
    Keqcrot, C4EnoylCoAMAT[t], C16EnoylCoAMAT[t], C14EnoylCoAMAT[t],
    C12EnoylCoAMAT[t], C10EnoylCoAMAT[t], C8EnoylCoAMAT[t],
    C6EnoylCoAMAT[t], C4HydroxyacylCoAMAT[t], C16HydroxyacylCoAMAT[t],
    C14HydroxyacylCoAMAT[t], C12HydroxyacylCoAMAT[t], C10HydroxyacylCoAMAT[t],
    C8HydroxyacylCoAMAT[t], C6HydroxyacylCoAMAT[t], C4AcetoacylCoAMAT[t]],
  vmschadC16 → MSCHAD[sfmschadC16, Vmschad, KmmschadC16HydroxyacylCoAMAT,
    KmmschadC14HydroxyacylCoAMAT, KmmschadC12HydroxyacylCoAMAT,
    KmmschadC10HydroxyacylCoAMAT, KmmschadC8HydroxyacylCoAMAT,
    KmmschadC6HydroxyacylCoAMAT, KmmschadC4HydroxyacylCoAMAT,
    KmmschadNADMAT, KmmschadC16KetoacylCoAMAT, KmmschadC14KetoacylCoAMAT,
    KmmschadC12KetoacylCoAMAT, KmmschadC10KetoacylCoAMAT, KmmschadC8KetoacylCoAMAT,
    KmmschadC6KetoacylCoAMAT, KmmschadC4AcetoacylCoAMAT, KmmschadNADHMAT,
    Keqmschad, C16HydroxyacylCoAMAT[t], C14HydroxyacylCoAMAT[t],
    C12HydroxyacylCoAMAT[t], C10HydroxyacylCoAMAT[t], C8HydroxyacylCoAMAT[t],
    C6HydroxyacylCoAMAT[t], C4HydroxyacylCoAMAT[t], NADtMAT, C16KetoacylCoAMAT[t],
    C14KetoacylCoAMAT[t], C12KetoacylCoAMAT[t], C10KetoacylCoAMAT[t],
    C8KetoacylCoAMAT[t], C6KetoacylCoAMAT[t], C4AcetoacylCoAMAT[t], NADHMAT],
  vmschadC14 → MSCHAD[sfmschadC14, Vmschad, KmmschadC14HydroxyacylCoAMAT,
    KmmschadC16HydroxyacylCoAMAT, KmmschadC12HydroxyacylCoAMAT,
    KmmschadC10HydroxyacylCoAMAT, KmmschadC8HydroxyacylCoAMAT,
    KmmschadC6HydroxyacylCoAMAT, KmmschadC4HydroxyacylCoAMAT,
    KmmschadNADMAT, KmmschadC14KetoacylCoAMAT, KmmschadC16KetoacylCoAMAT,
    KmmschadC12KetoacylCoAMAT, KmmschadC10KetoacylCoAMAT, KmmschadC8KetoacylCoAMAT,
    KmmschadC6KetoacylCoAMAT, KmmschadC4AcetoacylCoAMAT, KmmschadNADHMAT,
```

```
    Keqmschad, C14HydroxyacylCoAMAT[t], C16HydroxyacylCoAMAT[t],
    C12HydroxyacylCoAMAT[t], C10HydroxyacylCoAMAT[t], C8HydroxyacylCoAMAT[t],
    C6HydroxyacylCoAMAT[t], C4HydroxyacylCoAMAT[t], NADtMAT, C14KetoacylCoAMAT[t],
    C16KetoacylCoAMAT[t], C12KetoacylCoAMAT[t], C10KetoacylCoAMAT[t],
    C8KetoacylCoAMAT[t], C6KetoacylCoAMAT[t], C4AcetoacylCoAMAT[t], NADHMAT],
  vmschadC12 → MSCHAD[sfmschadC12, Vmschad, KmmschadC12HydroxyacylCoAMAT,
    KmmschadC16HydroxyacylCoAMAT, KmmschadC14HydroxyacylCoAMAT,
    KmmschadC10HydroxyacylCoAMAT, KmmschadC8HydroxyacylCoAMAT,
    KmmschadC6HydroxyacylCoAMAT, KmmschadC4HydroxyacylCoAMAT,
    KmmschadNADMAT, KmmschadC12KetoacylCoAMAT, KmmschadC16KetoacylCoAMAT,
    KmmschadC14KetoacylCoAMAT, KmmschadC10KetoacylCoAMAT, KmmschadC8KetoacylCoAMAT,
    KmmschadC6KetoacylCoAMAT, KmmschadC4AcetoacylCoAMAT, KmmschadNADHMAT,
    Keqmschad, C12HydroxyacylCoAMAT[t], C16HydroxyacylCoAMAT[t],
    C14HydroxyacylCoAMAT[t], C10HydroxyacylCoAMAT[t], C8HydroxyacylCoAMAT[t],
    C6HydroxyacylCoAMAT[t], C4HydroxyacylCoAMAT[t], NADtMAT, C12KetoacylCoAMAT[t],
    C16KetoacylCoAMAT[t], C14KetoacylCoAMAT[t], C10KetoacylCoAMAT[t],
    C8KetoacylCoAMAT[t], C6KetoacylCoAMAT[t], C4AcetoacylCoAMAT[t], NADHMAT],
  vmschadC10 → MSCHAD[sfmschadC10, Vmschad, KmmschadC10HydroxyacylCoAMAT,
    KmmschadC16HydroxyacylCoAMAT, KmmschadC14HydroxyacylCoAMAT,
    KmmschadC12HydroxyacylCoAMAT, KmmschadC8HydroxyacylCoAMAT,
    KmmschadC6HydroxyacylCoAMAT, KmmschadC4HydroxyacylCoAMAT,
    KmmschadNADMAT, KmmschadC10KetoacylCoAMAT, KmmschadC16KetoacylCoAMAT,
    KmmschadC14KetoacylCoAMAT, KmmschadC12KetoacylCoAMAT, KmmschadC8KetoacylCoAMAT,
    KmmschadC6KetoacylCoAMAT, KmmschadC4AcetoacylCoAMAT, KmmschadNADHMAT,
    Keqmschad, C10HydroxyacylCoAMAT[t], C16HydroxyacylCoAMAT[t],
    C14HydroxyacylCoAMAT[t], C12HydroxyacylCoAMAT[t], C8HydroxyacylCoAMAT[t],
    C6HydroxyacylCoAMAT[t], C4HydroxyacylCoAMAT[t], NADtMAT, C10KetoacylCoAMAT[t],
    C16KetoacylCoAMAT[t], C14KetoacylCoAMAT[t], C12KetoacylCoAMAT[t],
    C8KetoacylCoAMAT[t], C6KetoacylCoAMAT[t], C4AcetoacylCoAMAT[t], NADHMAT],
  vmschadC8 → MSCHAD[sfmschadC8, Vmschad, KmmschadC8HydroxyacylCoAMAT,
    KmmschadC16HydroxyacylCoAMAT, KmmschadC14HydroxyacylCoAMAT,
    KmmschadC12HydroxyacylCoAMAT, KmmschadC10HydroxyacylCoAMAT,
    KmmschadC6HydroxyacylCoAMAT, KmmschadC4HydroxyacylCoAMAT,
    KmmschadNADMAT, KmmschadC8KetoacylCoAMAT, KmmschadC16KetoacylCoAMAT,
    KmmschadC14KetoacylCoAMAT, KmmschadC12KetoacylCoAMAT, KmmschadC10KetoacylCoAMAT,
    KmmschadC6KetoacylCoAMAT, KmmschadC4AcetoacylCoAMAT, KmmschadNADHMAT,
    Keqmschad, C8HydroxyacylCoAMAT[t], C16HydroxyacylCoAMAT[t],
    C14HydroxyacylCoAMAT[t], C12HydroxyacylCoAMAT[t], C10HydroxyacylCoAMAT[t],
    C6HydroxyacylCoAMAT[t], C4HydroxyacylCoAMAT[t], NADtMAT, C8KetoacylCoAMAT[t],
    C16KetoacylCoAMAT[t], C14KetoacylCoAMAT[t], C12KetoacylCoAMAT[t],
    C10KetoacylCoAMAT[t], C6KetoacylCoAMAT[t], C4AcetoacylCoAMAT[t], NADHMAT],
  vmschadC6 → MSCHAD[sfmschadC6, Vmschad, KmmschadC6HydroxyacylCoAMAT,
    KmmschadC16HydroxyacylCoAMAT, KmmschadC14HydroxyacylCoAMAT,
    KmmschadC12HydroxyacylCoAMAT, KmmschadC10HydroxyacylCoAMAT,
    KmmschadC8HydroxyacylCoAMAT, KmmschadC4HydroxyacylCoAMAT,
    KmmschadNADMAT, KmmschadC6KetoacylCoAMAT, KmmschadC16KetoacylCoAMAT,
    KmmschadC14KetoacylCoAMAT, KmmschadC12KetoacylCoAMAT, KmmschadC10KetoacylCoAMAT,
    KmmschadC8KetoacylCoAMAT, KmmschadC4AcetoacylCoAMAT, KmmschadNADHMAT,
    Keqmschad, C6HydroxyacylCoAMAT[t], C16HydroxyacylCoAMAT[t],
    C14HydroxyacylCoAMAT[t], C12HydroxyacylCoAMAT[t], C10HydroxyacylCoAMAT[t],
    C8HydroxyacylCoAMAT[t], C4HydroxyacylCoAMAT[t], NADtMAT, C6KetoacylCoAMAT[t],
    C16KetoacylCoAMAT[t], C14KetoacylCoAMAT[t], C12KetoacylCoAMAT[t],
    C10KetoacylCoAMAT[t], C8KetoacylCoAMAT[t], C4AcetoacylCoAMAT[t], NADHMAT],
  vmschadC4 → MSCHAD[sfmschadC4, Vmschad, KmmschadC4HydroxyacylCoAMAT,
    KmmschadC16HydroxyacylCoAMAT, KmmschadC14HydroxyacylCoAMAT,
```

```
   KmmschadC12HydroxyacylCoAMAT, KmmschadC10HydroxyacylCoAMAT,
   KmmschadC8HydroxyacylCoAMAT, KmmschadC6HydroxyacylCoAMAT,
   KmmschadNADMAT, KmmschadC4AcetoacylCoAMAT, KmmschadC16KetoacylCoAMAT,
   KmmschadC14KetoacylCoAMAT, KmmschadC12KetoacylCoAMAT, KmmschadC10KetoacylCoAMAT,
   KmmschadC8KetoacylCoAMAT, KmmschadC6KetoacylCoAMAT, KmmschadNADHMAT,
   Keqmschad, C4HydroxyacylCoAMAT[t], C16HydroxyacylCoAMAT[t],
   C14HydroxyacylCoAMAT[t], C12HydroxyacylCoAMAT[t], C10HydroxyacylCoAMAT[t],
   C8HydroxyacylCoAMAT[t], C6HydroxyacylCoAMAT[t], NADtMAT, C4AcetoacylCoAMAT[t],
   C16KetoacylCoAMAT[t], C14KetoacylCoAMAT[t], C12KetoacylCoAMAT[t],
   C10KetoacylCoAMAT[t], C8KetoacylCoAMAT[t], C6KetoacylCoAMAT[t], NADHMAT],
vmckatC16 → MCKATA[sfmckatC16, Vmckat, KmmckatC16KetoacylCoAMAT,
   KmmckatC14KetoacylCoAMAT, KmmckatC12KetoacylCoAMAT, KmmckatC10KetoacylCoAMAT,
   KmmckatC8KetoacylCoAMAT, KmmckatC6KetoacylCoAMAT, KmmckatC4AcetoacylCoAMAT,
   KmmckatCoAMAT, KmmckatC14AcylCoAMAT, KmmckatC16AcylCoAMAT, KmmckatC12AcylCoAMAT,
   KmmckatC10AcylCoAMAT, KmmckatC8AcylCoAMAT, KmmckatC6AcylCoAMAT,
   KmmckatC4AcylCoAMAT, KmmckatAcetylCoAMAT, Keqmckat, C16KetoacylCoAMAT[t],
   C14KetoacylCoAMAT[t], C12KetoacylCoAMAT[t], C10KetoacylCoAMAT[t],
   C8KetoacylCoAMAT[t], C6KetoacylCoAMAT[t], C4AcetoacylCoAMAT[t], CoAMAT,
   C14AcylCoAMAT[t], C16AcylCoAMAT[t], C12AcylCoAMAT[t], C10AcylCoAMAT[t],
   C8AcylCoAMAT[t], C6AcylCoAMAT[t], C4AcylCoAMAT[t], AcetylCoAMAT],
vmckatC14 → MCKATA[sfmckatC14, Vmckat, KmmckatC14KetoacylCoAMAT,
   KmmckatC16KetoacylCoAMAT, KmmckatC12KetoacylCoAMAT, KmmckatC10KetoacylCoAMAT,
   KmmckatC8KetoacylCoAMAT, KmmckatC6KetoacylCoAMAT, KmmckatC4AcetoacylCoAMAT,
   KmmckatCoAMAT, KmmckatC12AcylCoAMAT, KmmckatC16AcylCoAMAT, KmmckatC14AcylCoAMAT,
   KmmckatC10AcylCoAMAT, KmmckatC8AcylCoAMAT, KmmckatC6AcylCoAMAT,
   KmmckatC4AcylCoAMAT, KmmckatAcetylCoAMAT, Keqmckat, C14KetoacylCoAMAT[t],
   C16KetoacylCoAMAT[t], C12KetoacylCoAMAT[t], C10KetoacylCoAMAT[t],
   C8KetoacylCoAMAT[t], C6KetoacylCoAMAT[t], C4AcetoacylCoAMAT[t], CoAMAT,
   C12AcylCoAMAT[t], C16AcylCoAMAT[t], C14AcylCoAMAT[t], C10AcylCoAMAT[t],
   C8AcylCoAMAT[t], C6AcylCoAMAT[t], C4AcylCoAMAT[t], AcetylCoAMAT],
vmckatC12 → MCKATA[sfmckatC12, Vmckat, KmmckatC12KetoacylCoAMAT,
   KmmckatC16KetoacylCoAMAT, KmmckatC14KetoacylCoAMAT, KmmckatC10KetoacylCoAMAT,
   KmmckatC8KetoacylCoAMAT, KmmckatC6KetoacylCoAMAT, KmmckatC4AcetoacylCoAMAT,
   KmmckatCoAMAT, KmmckatC10AcylCoAMAT, KmmckatC16AcylCoAMAT, KmmckatC14AcylCoAMAT,
   KmmckatC12AcylCoAMAT, KmmckatC8AcylCoAMAT, KmmckatC6AcylCoAMAT,
   KmmckatC4AcylCoAMAT, KmmckatAcetylCoAMAT, Keqmckat, C12KetoacylCoAMAT[t],
   C16KetoacylCoAMAT[t], C14KetoacylCoAMAT[t], C10KetoacylCoAMAT[t],
   C8KetoacylCoAMAT[t], C6KetoacylCoAMAT[t], C4AcetoacylCoAMAT[t], CoAMAT,
   C10AcylCoAMAT[t], C16AcylCoAMAT[t], C14AcylCoAMAT[t], C12AcylCoAMAT[t],
   C8AcylCoAMAT[t], C6AcylCoAMAT[t], C4AcylCoAMAT[t], AcetylCoAMAT],
vmckatC10 → MCKATA[sfmckatC10, Vmckat, KmmckatC10KetoacylCoAMAT,
   KmmckatC16KetoacylCoAMAT, KmmckatC14KetoacylCoAMAT, KmmckatC12KetoacylCoAMAT,
   KmmckatC8KetoacylCoAMAT, KmmckatC6KetoacylCoAMAT, KmmckatC4AcetoacylCoAMAT,
   KmmckatCoAMAT, KmmckatC8AcylCoAMAT, KmmckatC16AcylCoAMAT, KmmckatC14AcylCoAMAT,
   KmmckatC12AcylCoAMAT, KmmckatC10AcylCoAMAT, KmmckatC6AcylCoAMAT,
   KmmckatC4AcylCoAMAT, KmmckatAcetylCoAMAT, Keqmckat, C10KetoacylCoAMAT[t],
   C16KetoacylCoAMAT[t], C14KetoacylCoAMAT[t], C12KetoacylCoAMAT[t],
   C8KetoacylCoAMAT[t], C6KetoacylCoAMAT[t], C4AcetoacylCoAMAT[t], CoAMAT,
   C8AcylCoAMAT[t], C16AcylCoAMAT[t], C14AcylCoAMAT[t], C12AcylCoAMAT[t],
   C10AcylCoAMAT[t], C6AcylCoAMAT[t], C4AcylCoAMAT[t], AcetylCoAMAT],
vmckatC8 → MCKATA[sfmckatC8, Vmckat, KmmckatC8KetoacylCoAMAT,
   KmmckatC16KetoacylCoAMAT, KmmckatC14KetoacylCoAMAT, KmmckatC12KetoacylCoAMAT,
   KmmckatC10KetoacylCoAMAT, KmmckatC6KetoacylCoAMAT, KmmckatC4AcetoacylCoAMAT,
   KmmckatCoAMAT, KmmckatC6AcylCoAMAT, KmmckatC16AcylCoAMAT, KmmckatC14AcylCoAMAT,
   KmmckatC12AcylCoAMAT, KmmckatC10AcylCoAMAT, KmmckatC8AcylCoAMAT,
```

```
    KmmckatC4AcylCoAMAT, KmmckatAcetylCoAMAT, Keqmckat, C8KetoacylCoAMAT[t],
    C16KetoacylCoAMAT[t], C14KetoacylCoAMAT[t], C12KetoacylCoAMAT[t],
    C10KetoacylCoAMAT[t], C6KetoacylCoAMAT[t], C4AcetoacylCoAMAT[t], CoAMAT,
    C6AcylCoAMAT[t], C16AcylCoAMAT[t], C14AcylCoAMAT[t], C12AcylCoAMAT[t],
    C10AcylCoAMAT[t], C8AcylCoAMAT[t], C4AcylCoAMAT[t], AcetylCoAMAT],
  vmckatC6 → MCKATA[sfmckatC6, Vmckat, KmmckatC6KetoacylCoAMAT,
    KmmckatC16KetoacylCoAMAT, KmmckatC14KetoacylCoAMAT, KmmckatC12KetoacylCoAMAT,
    KmmckatC10KetoacylCoAMAT, KmmckatC8KetoacylCoAMAT, KmmckatC4AcetoacylCoAMAT,
    KmmckatCoAMAT, KmmckatC4AcylCoAMAT, KmmckatC16AcylCoAMAT, KmmckatC14AcylCoAMAT,
    KmmckatC12AcylCoAMAT, KmmckatC10AcylCoAMAT, KmmckatC8AcylCoAMAT,
    KmmckatC6AcylCoAMAT, KmmckatAcetylCoAMAT, Keqmckat, C6KetoacylCoAMAT[t],
    C16KetoacylCoAMAT[t], C14KetoacylCoAMAT[t], C12KetoacylCoAMAT[t],
    C10KetoacylCoAMAT[t], C8KetoacylCoAMAT[t], C4AcetoacylCoAMAT[t], CoAMAT,
    C4AcylCoAMAT[t], C16AcylCoAMAT[t], C14AcylCoAMAT[t], C12AcylCoAMAT[t],
    C10AcylCoAMAT[t], C8AcylCoAMAT[t], C6AcylCoAMAT[t], AcetylCoAMAT],
  vmckatC4 → MCKATB[sfmckatC4, Vmckat, KmmckatC4AcetoacylCoAMAT,
    KmmckatC16KetoacylCoAMAT, KmmckatC14KetoacylCoAMAT, KmmckatC12KetoacylCoAMAT,
    KmmckatC10KetoacylCoAMAT, KmmckatC8KetoacylCoAMAT, KmmckatC6KetoacylCoAMAT,
    KmmckatCoAMAT, KmmckatC4AcylCoAMAT, KmmckatC16AcylCoAMAT, KmmckatC14AcylCoAMAT,
    KmmckatC12AcylCoAMAT, KmmckatC10AcylCoAMAT, KmmckatC8AcylCoAMAT,
    KmmckatC6AcylCoAMAT, KmmckatAcetylCoAMAT, Keqmckat, C4AcetoacylCoAMAT[t],
    C16KetoacylCoAMAT[t], C14KetoacylCoAMAT[t], C12KetoacylCoAMAT[t],
    C10KetoacylCoAMAT[t], C8KetoacylCoAMAT[t], C6KetoacylCoAMAT[t], CoAMAT,
    C4AcylCoAMAT[t], C16AcylCoAMAT[t], C14AcylCoAMAT[t], C12AcylCoAMAT[t],
    C10AcylCoAMAT[t], C8AcylCoAMAT[t], C6AcylCoAMAT[t], AcetylCoAMAT],
  vmtpC16 → MTP[sfmtpC16, Vmtp, KmmtpC16EnoylCoAMAT, KmmtpC14EnoylCoAMAT,
    KmmtpC12EnoylCoAMAT, KmmtpC10EnoylCoAMAT, KmmtpC8EnoylCoAMAT,
    KmmtpNADMAT, KmmtpCoAMAT, KmmtpC14AcylCoAMAT, KmmtpC16AcylCoAMAT,
    KmmtpC12AcylCoAMAT, KmmtpC10AcylCoAMAT, KmmtpC8AcylCoAMAT,
    KmmtpC6AcylCoAMAT, KmmtpNADHMAT, KmmtpAcetylCoAMAT, KicrotC4AcetoacylCoA,
    Keqmtp, C16EnoylCoAMAT[t], C14EnoylCoAMAT[t], C12EnoylCoAMAT[t],
    C10EnoylCoAMAT[t], C8EnoylCoAMAT[t], NADtMAT, CoAMAT, C14AcylCoAMAT[t],
    C16AcylCoAMAT[t], C12AcylCoAMAT[t], C10AcylCoAMAT[t], C8AcylCoAMAT[t],
    C6AcylCoAMAT[t], NADHMAT, AcetylCoAMAT, C4AcetoacylCoAMAT[t]],
  vmtpC14 → MTP[sfmtpC14, Vmtp, KmmtpC14EnoylCoAMAT, KmmtpC16EnoylCoAMAT,
    KmmtpC12EnoylCoAMAT, KmmtpC10EnoylCoAMAT, KmmtpC8EnoylCoAMAT,
    KmmtpNADMAT, KmmtpCoAMAT, KmmtpC12AcylCoAMAT, KmmtpC16AcylCoAMAT,
    KmmtpC14AcylCoAMAT, KmmtpC10AcylCoAMAT, KmmtpC8AcylCoAMAT,
    KmmtpC6AcylCoAMAT, KmmtpNADHMAT, KmmtpAcetylCoAMAT, KicrotC4AcetoacylCoA,
    Keqmtp, C14EnoylCoAMAT[t], C16EnoylCoAMAT[t], C12EnoylCoAMAT[t],
    C10EnoylCoAMAT[t], C8EnoylCoAMAT[t], NADtMAT, CoAMAT, C12AcylCoAMAT[t],
    C16AcylCoAMAT[t], C14AcylCoAMAT[t], C10AcylCoAMAT[t], C8AcylCoAMAT[t],
    C6AcylCoAMAT[t], NADHMAT, AcetylCoAMAT, C4AcetoacylCoAMAT[t]],
  vmtpC12 → MTP[sfmtpC12, Vmtp, KmmtpC12EnoylCoAMAT, KmmtpC16EnoylCoAMAT,
    KmmtpC14EnoylCoAMAT, KmmtpC10EnoylCoAMAT, KmmtpC8EnoylCoAMAT,
    KmmtpNADMAT, KmmtpCoAMAT, KmmtpC10AcylCoAMAT, KmmtpC16AcylCoAMAT,
    KmmtpC14AcylCoAMAT, KmmtpC12AcylCoAMAT, KmmtpC8AcylCoAMAT,
    KmmtpC6AcylCoAMAT, KmmtpNADHMAT, KmmtpAcetylCoAMAT, KicrotC4AcetoacylCoA,
    Keqmtp, C12EnoylCoAMAT[t], C16EnoylCoAMAT[t], C14EnoylCoAMAT[t],
    C10EnoylCoAMAT[t], C8EnoylCoAMAT[t], NADtMAT, CoAMAT, C10AcylCoAMAT[t],
    C16AcylCoAMAT[t], C14AcylCoAMAT[t], C12AcylCoAMAT[t], C8AcylCoAMAT[t],
    C6AcylCoAMAT[t], NADHMAT, AcetylCoAMAT, C4AcetoacylCoAMAT[t]],
  vmtpC10 → MTP[sfmtpC10, Vmtp, KmmtpC10EnoylCoAMAT, KmmtpC16EnoylCoAMAT,
    KmmtpC14EnoylCoAMAT, KmmtpC12EnoylCoAMAT, KmmtpC8EnoylCoAMAT,
    KmmtpNADMAT, KmmtpCoAMAT, KmmtpC8AcylCoAMAT, KmmtpC16AcylCoAMAT,
```

```
      KmmtpC14AcylCoAMAT, KmmtpC12AcylCoAMAT, KmmtpC10AcylCoAMAT,
      KmmtpC6AcylCoAMAT, KmmtpNADHMAT, KmmtpAcetylCoAMAT, KicrotC4AcetoacylCoA,
      Keqmtp, C10EnoylCoAMAT[t], C16EnoylCoAMAT[t], C14EnoylCoAMAT[t],
      C12EnoylCoAMAT[t], C8EnoylCoAMAT[t], NADtMAT, CoAMAT, C8AcylCoAMAT[t],
      C16AcylCoAMAT[t], C14AcylCoAMAT[t], C12AcylCoAMAT[t], C10AcylCoAMAT[t],
      C6AcylCoAMAT[t], NADHMAT, AcetylCoAMAT, C4AcetoacylCoAMAT[t]],
    vmtpC8 → MTP[sfmtpC8, Vmtp, KmmtpC8EnoylCoAMAT, KmmtpC16EnoylCoAMAT,
      KmmtpC14EnoylCoAMAT, KmmtpC12EnoylCoAMAT, KmmtpC10EnoylCoAMAT,
      KmmtpNADMAT, KmmtpCoAMAT, KmmtpC6AcylCoAMAT, KmmtpC16AcylCoAMAT,
      KmmtpC14AcylCoAMAT, KmmtpC12AcylCoAMAT, KmmtpC10AcylCoAMAT,
      KmmtpC8AcylCoAMAT, KmmtpNADHMAT, KmmtpAcetylCoAMAT, KicrotC4AcetoacylCoA,
      Keqmtp, C8EnoylCoAMAT[t], C16EnoylCoAMAT[t], C14EnoylCoAMAT[t],
      C12EnoylCoAMAT[t], C10EnoylCoAMAT[t], NADtMAT, CoAMAT, C6AcylCoAMAT[t],
      C16AcylCoAMAT[t], C14AcylCoAMAT[t], C12AcylCoAMAT[t], C10AcylCoAMAT[t],
      C8AcylCoAMAT[t], NADHMAT, AcetylCoAMAT, C4AcetoacylCoAMAT[t]],
    vacesink → RES[Ksacesink, AcetylCoAMAT, K1acesink],
    vfadhsink → RES[Ksfadhsink, FADHMAT, K1fadhsink],
    vnadhsink → RES[Ksnadhsink, NADHMAT, K1nadhsink]};

CoAMATX =
   {CoAMAT → CoAMATt - C16AcylCoAMAT[t] - C16EnoylCoAMAT[t] - C16HydroxyacylCoAMAT[t] -
      C16KetoacylCoAMAT[t] - C14AcylCoAMAT[t] - C14EnoylCoAMAT[t] -
      C14HydroxyacylCoAMAT[t] - C14KetoacylCoAMAT[t] - C12AcylCoAMAT[t] -
      C12EnoylCoAMAT[t] - C12HydroxyacylCoAMAT[t] - C12KetoacylCoAMAT[t] -
      C10AcylCoAMAT[t] - C10EnoylCoAMAT[t] - C10HydroxyacylCoAMAT[t] -
      C10KetoacylCoAMAT[t] - C8AcylCoAMAT[t] - C8EnoylCoAMAT[t] -
      C8HydroxyacylCoAMAT[t] - C8KetoacylCoAMAT[t] - C6AcylCoAMAT[t] - C6EnoylCoAMAT[t] -
      C6HydroxyacylCoAMAT[t] - C6KetoacylCoAMAT[t] - C4AcylCoAMAT[t] -
      C4EnoylCoAMAT[t] - C4HydroxyacylCoAMAT[t] - C4AcetoacylCoAMAT[t] - AcetylCoAMAT};

Parm = {
    sfcpt1C16 → 1, Vcpt1 → 0.012, Kmcpt1C16AcylCoACYT → 13.8,
    Kmcpt1CarCYT → 250, Kmcpt1C16AcylCarCYT → 136, Kmcpt1CoACYT → 40.7,
    Kicpt1MalCoACYT → 9.1, Keqcpt1 → 0.45, ncpt1 → 2.4799,
    Vfcact → 0.42, Vrcact → 0.42, KmcactC16AcylCarCYT → 15,
    KmcactC14AcylCarCYT → 15, KmcactC12AcylCarCYT → 15, KmcactC10AcylCarCYT → 15,
    KmcactC8AcylCarCYT → 15, KmcactC6AcylCarCYT → 15, KmcactC4AcylCarCYT → 15,
    KmcactCarMAT → 130, KmcactC16AcylCarMAT → 15, KmcactC14AcylCarMAT → 15,
    KmcactC12AcylCarMAT → 15, KmcactC10AcylCarMAT → 15, KmcactC8AcylCarMAT → 15,
    KmcactC6AcylCarMAT → 15, KmcactC4AcylCarMAT → 15, KmcactCarCYT → 130,
    KicactC16AcylCarCYT → 56, KicactC14AcylCarCYT → 56, KicactC12AcylCarCYT → 56,
    KicactC10AcylCarCYT → 56, KicactC8AcylCarCYT → 56, KicactC6AcylCarCYT → 56,
    KicactC4AcylCarCYT → 56, KicactCarCYT → 200, Keqcact → 1,
    sfcpt2C16 → 0.85, sfcpt2C14 → 1, sfcpt2C12 → 0.95, sfcpt2C10 → 0.95,
    sfcpt2C8 → 0.35, sfcpt2C6 → 0.15, sfcpt2C4 → 0.01, Vcpt2 → 0.391,
    Kmcpt2C16AcylCarMAT → 51, Kmcpt2C14AcylCarMAT → 51, Kmcpt2C12AcylCarMAT → 51,
    Kmcpt2C10AcylCarMAT → 51, Kmcpt2C8AcylCarMAT → 51, Kmcpt2C6AcylCarMAT → 51,
    Kmcpt2C4AcylCarMAT → 51, Kmcpt2CoAMAT → 30, Kmcpt2C16AcylCoAMAT → 38,
    Kmcpt2C14AcylCoAMAT → 38, Kmcpt2C12AcylCoAMAT → 38,
    Kmcpt2C10AcylCoAMAT → 38, Kmcpt2C8AcylCoAMAT → 38, Kmcpt2C6AcylCoAMAT → 1000,
    Kmcpt2C4AcylCoAMAT → 1000000, Kmcpt2CarMAT → 350, Keqcpt2 → 2.22,
    sfvlcadC16 → 1, sfvlcadC14 → 0.42, sfvlcadC12 → 0.11, Vvlcad → 0.008,
    KmvlcadC16AcylCoAMAT → 6.5, KmvlcadC14AcylCoAMAT → 4, KmvlcadC12AcylCoAMAT → 2.7,
    KmvlcadFAD → 0.12, KmvlcadC16EnoylCoAMAT → 1.08, KmvlcadC14EnoylCoAMAT → 1.08,
    KmvlcadC12EnoylCoAMAT → 1.08, KmvlcadFADH → 24.2, Keqvlcad → 6,
```

```
sflcadC16 → 0.9, sflcadC14 → 1, sflcadC12 → 0.9, sflcadC10 → 0.75, sflcadC8 → 0.4,
Vlcad → 0.01, KmlcadC16AcylCoAMAT → 2.5, KmlcadC14AcylCoAMAT → 7.4,
KmlcadC12AcylCoAMAT → 9, KmlcadC10AcylCoAMAT → 24.3, KmlcadC8AcylCoAMAT → 123,
KmlcadFAD → 0.12, KmlcadC16EnoylCoAMAT → 1.08, KmlcadC14EnoylCoAMAT → 1.08,
KmlcadC12EnoylCoAMAT → 1.08, KmlcadC10EnoylCoAMAT → 1.08,
KmlcadC8EnoylCoAMAT → 1.08, KmlcadFADH → 24.2, Keqlcad → 6,
sfmcadC12 → 0.38, sfmcadC10 → 0.8, sfmcadC8 → 0.87, sfmcadC6 → 1, sfmcadC4 → 0.12,
Vmcad → 0.081, KmmcadC12AcylCoAMAT → 5.7, KmmcadC10AcylCoAMAT → 5.4,
KmmcadC8AcylCoAMAT → 4, KmmcadC6AcylCoAMAT → 9.4, KmmcadC4AcylCoAMAT → 135,
KmmcadFAD → 0.12, KmmcadC12EnoylCoAMAT → 1.08, KmmcadC10EnoylCoAMAT → 1.08,
KmmcadC8EnoylCoAMAT → 1.08, KmmcadC6EnoylCoAMAT → 1.08,
KmmcadC4EnoylCoAMAT → 1.08, KmmcadFADH → 24.2, Keqmcad → 6,
sfscadC6 → 0.3, sfscadC4 → 1, Vscad → 0.081, KmscadC6AcylCoAMAT → 285,
KmscadC4AcylCoAMAT → 10.7, KmscadFAD → 0.12, KmscadC6EnoylCoAMAT → 1.08,
KmscadC4EnoylCoAMAT → 1.08, KmscadFADH → 24.2, Keqscad -> 6,
sfcrotC16 → 0.13, sfcrotC14 → 0.2, sfcrotC12 → 0.25, sfcrotC10 → 0.33, sfcrotC8 → 0.58,
sfcrotC6 → 0.83, sfcrotC4 → 1, Vcrot → 3.6, KmcrotC16EnoylCoAMAT → 150,
KmcrotC14EnoylCoAMAT → 100, KmcrotC12EnoylCoAMAT → 25, KmcrotC10EnoylCoAMAT → 25,
KmcrotC8EnoylCoAMAT → 25, KmcrotC6EnoylCoAMAT → 25, KmcrotC4EnoylCoAMAT → 40,
KmcrotC16HydroxyacylCoAMAT → 45, KmcrotC14HydroxyacylCoAMAT → 45,
KmcrotC12HydroxyacylCoAMAT → 45, KmcrotC10HydroxyacylCoAMAT → 45,
KmcrotC8HydroxyacylCoAMAT → 45, KmcrotC6HydroxyacylCoAMAT → 45,
KmcrotC4HydroxyacylCoAMAT → 45, KicrotC4AcetoacylCoA → 1.6, Keqcrot → 3.13,
sfmschadC16 → 0.6, sfmschadC14 → 0.5, sfmschadC12 → 0.43, sfmschadC10 → 0.64,
sfmschadC8 → 0.89, sfmschadC6 → 1, sfmschadC4 → 0.67, Vmschad → 1,
KmmschadC16HydroxyacylCoAMAT → 1.5, KmmschadC14HydroxyacylCoAMAT → 1.8,
KmmschadC12HydroxyacylCoAMAT → 3.7, KmmschadC10HydroxyacylCoAMAT → 8.8,
KmmschadC8HydroxyacylCoAMAT → 16.3, KmmschadC6HydroxyacylCoAMAT → 28.6,
KmmschadC4HydroxyacylCoAMAT → 69.9, KmmschadNADMAT → 58.5,
KmmschadC16KetoacylCoAMAT → 1.4, KmmschadC14KetoacylCoAMAT → 1.4,
KmmschadC12KetoacylCoAMAT → 1.6, KmmschadC10KetoacylCoAMAT → 2.3,
KmmschadC8KetoacylCoAMAT → 4.1, KmmschadC6KetoacylCoAMAT → 5.8,
KmmschadC4AcetoacylCoAMAT → 16.9, KmmschadNADHMAT → 5.4, Keqmschad → 2.17 * 10^-4,
sfmckatC16 → 0, sfmckatC14 → 0.2, sfmckatC12 → 0.38, sfmckatC10 → 0.65,
sfmckatC8 → 0.81, sfmckatC6 → 1, sfmckatC4 → 0.49, Vmckat → 0.377,
KmmckatC16KetoacylCoAMAT → 1.1, KmmckatC14KetoacylCoAMAT → 1.2,
KmmckatC12KetoacylCoAMAT → 1.3, KmmckatC10KetoacylCoAMAT → 2.1,
KmmckatC8KetoacylCoAMAT → 3.2, KmmckatC6KetoacylCoAMAT → 6.7,
KmmckatC4AcetoacylCoAMAT → 12.4, KmmckatCoAMAT → 26.6,
KmmckatC14AcylCoAMAT → 13.83, KmmckatC16AcylCoAMAT → 13.83,
KmmckatC12AcylCoAMAT → 13.83, KmmckatC10AcylCoAMAT → 13.83,
KmmckatC8AcylCoAMAT → 13.83, KmmckatC6AcylCoAMAT → 13.83,
KmmckatC4AcylCoAMAT → 13.83, KmmckatAcetylCoAMAT → 30, Keqmckat → 1051,
sfmtpC16 → 1, sfmtpC14 → 0.9, sfmtpC12 → 0.81, sfmtpC10 → 0.73, sfmtpC8 → 0.34,
Vmtp → 2.84, KmmtpC16EnoylCoAMAT → 25, KmmtpC14EnoylCoAMAT → 25,
KmmtpC12EnoylCoAMAT → 25, KmmtpC10EnoylCoAMAT → 25, KmmtpC8EnoylCoAMAT → 25,
KmmtpNADMAT → 60, KmmtpCoAMAT → 30, KmmtpC14AcylCoAMAT → 13.83,
KmmtpC16AcylCoAMAT → 13.83, KmmtpC12AcylCoAMAT → 13.83,
KmmtpC10AcylCoAMAT → 13.83, KmmtpC8AcylCoAMAT → 13.83, KmmtpC6AcylCoAMAT → 13.83,
KmmtpNADHMAT → 50, KmmtpAcetylCoAMAT → 30, Keqmtp → 0.71,
Ksacesink → 6000000, K1acesink → 70, Ksfadhsink → 6000000,
K1fadhsink → 0.46, Ksnadhsink → 6000000, K1nadhsink → 12,
C16AcylCoACYT → 25, CarCYT → 200, CoACYT → 140, MalCoACYT → 0,
CarMAT -> 950, FADtMAT → 0.77, NADtMAT → 250, CoAMATt → 5000,
VCYT → 2.2 * 10^-6, VMAT → 1.8 * 10^-6, AcetylCoAMAT → 70, FADHMAT → 0.46, NADHMAT → 12};
```

```
InitialConditions = {
   C16AcylCarCYT[0] == 0, C16AcylCarMAT[0] == 0, C16AcylCoAMAT[0] == 0,
   C16EnoylCoAMAT[0] == 0, C16HydroxyacylCoAMAT[0] == 0, C16KetoacylCoAMAT[0] == 0,
   C14AcylCarCYT[0] == 0, C14AcylCarMAT[0] == 0, C14AcylCoAMAT[0] == 0,
   C14EnoylCoAMAT[0] == 0, C14HydroxyacylCoAMAT[0] == 0, C14KetoacylCoAMAT[0] == 0,
   C12AcylCarCYT[0] == 0, C12AcylCarMAT[0] == 0, C12AcylCoAMAT[0] == 0,
   C12EnoylCoAMAT[0] == 0, C12HydroxyacylCoAMAT[0] == 0, C12KetoacylCoAMAT[0] == 0,
   C10AcylCarCYT[0] == 0, C10AcylCarMAT[0] == 0, C10AcylCoAMAT[0] == 0,
   C10EnoylCoAMAT[0] == 0, C10HydroxyacylCoAMAT[0] == 0, C10KetoacylCoAMAT[0] == 0,
   C8AcylCarCYT[0] == 0, C8AcylCarMAT[0] == 0, C8AcylCoAMAT[0] == 0,
   C8EnoylCoAMAT[0] == 0, C8HydroxyacylCoAMAT[0] == 0, C8KetoacylCoAMAT[0] == 0,
   C6AcylCarCYT[0] == 0, C6AcylCarMAT[0] == 0, C6AcylCoAMAT[0] == 0,
   C6EnoylCoAMAT[0] == 0, C6HydroxyacylCoAMAT[0] == 0, C6KetoacylCoAMAT[0] == 0,
   C4AcylCarCYT[0] == 0, C4AcylCarMAT[0] == 0, C4AcylCoAMAT[0] == 0,
   C4EnoylCoAMAT[0] == 0, C4HydroxyacylCoAMAT[0] == 0, C4AcetoacylCoAMAT[0] == 0};

Vars = {
   C16AcylCarCYT, C16AcylCarMAT, C16AcylCoAMAT,
   C16EnoylCoAMAT, C16HydroxyacylCoAMAT, C16KetoacylCoAMAT,
   C14AcylCarCYT, C14AcylCarMAT, C14AcylCoAMAT, C14EnoylCoAMAT,
   C14HydroxyacylCoAMAT, C14KetoacylCoAMAT,
   C12AcylCarCYT, C12AcylCarMAT, C12AcylCoAMAT, C12EnoylCoAMAT,
   C12HydroxyacylCoAMAT, C12KetoacylCoAMAT,
   C10AcylCarCYT, C10AcylCarMAT, C10AcylCoAMAT, C10EnoylCoAMAT,
   C10HydroxyacylCoAMAT, C10KetoacylCoAMAT,
   C8AcylCarCYT, C8AcylCarMAT, C8AcylCoAMAT, C8EnoylCoAMAT,
   C8HydroxyacylCoAMAT, C8KetoacylCoAMAT,
   C6AcylCarCYT, C6AcylCarMAT, C6AcylCoAMAT, C6EnoylCoAMAT,
   C6HydroxyacylCoAMAT, C6KetoacylCoAMAT,
   C4AcylCarCYT, C4AcylCarMAT, C4AcylCoAMAT, C4EnoylCoAMAT,
   C4HydroxyacylCoAMAT, C4AcetoacylCoAMAT};
```

In[*]:=
```
TableForm[Odes];
TableForm[RateEqs];
TableForm[Odes /. RateEqs /. CoAMATX /. Parm ];
TableForm[RateEqs /. Parm ];
TableForm[InitialConditions];
```

In[*]:=
```
tsol = NDSolve[Join[Odes /. RateEqs /. CoAMATX /. Parm , InitialConditions],
   Vars, {t, 0, 1000000000}];
```

In[ ]:=
```
Table[{Vars[[i]][t], (Vars[[i]][900000000] /. tsol)[[1]]}, {i, 1, Length[Vars]}]
```

Out[ ]=
```
{{C16AcylCarCYT[t], 0.167997}, {C16AcylCarMAT[t], 0.355963},
 {C16AcylCoAMAT[t], 0.872403}, {C16EnoylCoAMAT[t], 0.0487436},
 {C16HydroxyacylCoAMAT[t], 0.152568}, {C16KetoacylCoAMAT[t], 0.000656626},
 {C14AcylCarCYT[t], 0.0373818}, {C14AcylCarMAT[t], 0.177564},
 {C14AcylCoAMAT[t], 1.93364}, {C14EnoylCoAMAT[t], 0.0544366},
 {C14HydroxyacylCoAMAT[t], 0.154607}, {C14KetoacylCoAMAT[t], 0.000664719},
 {C12AcylCarCYT[t], 0.0510162}, {C12AcylCarMAT[t], 0.242327},
 {C12AcylCoAMAT[t], 2.63889}, {C12EnoylCoAMAT[t], 0.0621263},
 {C12HydroxyacylCoAMAT[t], 0.187943}, {C12KetoacylCoAMAT[t], 0.000805513},
 {C10AcylCarCYT[t], 0.0916998}, {C10AcylCarMAT[t], 0.435574},
 {C10AcylCoAMAT[t], 4.74332}, {C10EnoylCoAMAT[t], 0.0684024},
 {C10HydroxyacylCoAMAT[t], 0.208289}, {C10KetoacylCoAMAT[t], 0.000890113},
 {C8AcylCarCYT[t], 0.0941203}, {C8AcylCarMAT[t], 0.447072},
 {C8AcylCoAMAT[t], 4.86853}, {C8EnoylCoAMAT[t], 0.148284},
 {C8HydroxyacylCoAMAT[t], 0.458288}, {C8KetoacylCoAMAT[t], 0.0019575},
 {C6AcylCarCYT[t], 0.249827}, {C6AcylCarMAT[t], 1.18668}, {C6AcylCoAMAT[t], 12.9227},
 {C6EnoylCoAMAT[t], 11.1239}, {C6HydroxyacylCoAMAT[t], 34.6175},
 {C6KetoacylCoAMAT[t], 0.147847}, {C4AcylCarCYT[t], 0.414653},
 {C4AcylCarMAT[t], 1.9696}, {C4AcylCoAMAT[t], 21.4486}, {C4EnoylCoAMAT[t], 41.836},
 {C4HydroxyacylCoAMAT[t], 130.681}, {C4AcetoacylCoAMAT[t], 0.558265}}
```

A. Steady state computation with varying palmitoyl-CoA (X) and NAD:-NADH ratio (Z)

In[ ]:=
```
ParmScan[X_, Z_] := {
   sfcpt1C16 → 1, Vcpt1 → 0.012, Kmcpt1C16AcylCoACYT → 13.8,
   Kmcpt1CarCYT → 250, Kmcpt1C16AcylCarCYT → 136, Kmcpt1CoACYT → 40.7,
   Kicpt1MalCoACYT → 9.1, Keqcpt1 → 0.45,  ncpt1 → 2.4799,
   Vfcact → 0.42, Vrcact → 0.42, KmcactC16AcylCarCYT → 15,
   KmcactC14AcylCarCYT → 15, KmcactC12AcylCarCYT → 15, KmcactC10AcylCarCYT → 15,
   KmcactC8AcylCarCYT → 15, KmcactC6AcylCarCYT → 15, KmcactC4AcylCarCYT → 15,
   KmcactCarMAT → 130, KmcactC16AcylCarMAT → 15, KmcactC14AcylCarMAT → 15,
   KmcactC12AcylCarMAT → 15, KmcactC10AcylCarMAT → 15, KmcactC8AcylCarMAT → 15,
   KmcactC6AcylCarMAT → 15, KmcactC4AcylCarMAT → 15, KmcactCarCYT → 130,
   KicactC16AcylCarCYT → 56, KicactC14AcylCarCYT → 56, KicactC12AcylCarCYT → 56,
   KicactC10AcylCarCYT → 56, KicactC8AcylCarCYT → 56, KicactC6AcylCarCYT → 56,
   KicactC4AcylCarCYT → 56, KicactCarCYT → 200, Keqcact → 1,
   sfcpt2C16 → 0.85, sfcpt2C14 → 1, sfcpt2C12 → 0.95, sfcpt2C10 → 0.95,
   sfcpt2C8 → 0.35, sfcpt2C6 → 0.15, sfcpt2C4 → 0.01, Vcpt2 → 0.391,
   Kmcpt2C16AcylCarMAT → 51, Kmcpt2C14AcylCarMAT → 51, Kmcpt2C12AcylCarMAT → 51,
   Kmcpt2C10AcylCarMAT → 51, Kmcpt2C8AcylCarMAT → 51, Kmcpt2C6AcylCarMAT → 51,
   Kmcpt2C4AcylCarMAT → 51, Kmcpt2CoAMAT → 30, Kmcpt2C16AcylCoAMAT → 38,
   Kmcpt2C14AcylCoAMAT → 38, Kmcpt2C12AcylCoAMAT → 38,
   Kmcpt2C10AcylCoAMAT → 38, Kmcpt2C8AcylCoAMAT → 38, Kmcpt2C6AcylCoAMAT → 1000,
   Kmcpt2C4AcylCoAMAT → 1000000, Kmcpt2CarMAT → 350, Keqcpt2 → 2.22,
   sfvlcadC16 → 1, sfvlcadC14 → 0.42, sfvlcadC12 → 0.11, Vvlcad → 0.008,
   KmvlcadC16AcylCoAMAT → 6.5, KmvlcadC14AcylCoAMAT → 4, KmvlcadC12AcylCoAMAT → 2.7,
   KmvlcadFAD → 0.12, KmvlcadC16EnoylCoAMAT → 1.08, KmvlcadC14EnoylCoAMAT → 1.08,
   KmvlcadC12EnoylCoAMAT → 1.08, KmvlcadFADH → 24.2, Keqvlcad → 6,
```

```
sflcadC16 → 0.9, sflcadC14 → 1, sflcadC12 → 0.9, sflcadC10 → 0.75, sflcadC8 → 0.4,
Vlcad → 0.01, KmlcadC16AcylCoAMAT → 2.5, KmlcadC14AcylCoAMAT → 7.4,
KmlcadC12AcylCoAMAT → 9, KmlcadC10AcylCoAMAT → 24.3, KmlcadC8AcylCoAMAT → 123,
KmlcadFAD → 0.12, KmlcadC16EnoylCoAMAT → 1.08, KmlcadC14EnoylCoAMAT → 1.08,
KmlcadC12EnoylCoAMAT → 1.08, KmlcadC10EnoylCoAMAT → 1.08,
KmlcadC8EnoylCoAMAT → 1.08, KmlcadFADH → 24.2, Keqlcad → 6,
sfmcadC12 → 0.38, sfmcadC10 → 0.8, sfmcadC8 → 0.87, sfmcadC6 → 1, sfmcadC4 → 0.12,
Vmcad → 0.081, KmmcadC12AcylCoAMAT → 5.7, KmmcadC10AcylCoAMAT → 5.4,
KmmcadC8AcylCoAMAT → 4, KmmcadC6AcylCoAMAT → 9.4, KmmcadC4AcylCoAMAT → 135,
KmmcadFAD → 0.12, KmmcadC12EnoylCoAMAT → 1.08, KmmcadC10EnoylCoAMAT → 1.08,
KmmcadC8EnoylCoAMAT → 1.08, KmmcadC6EnoylCoAMAT → 1.08,
KmmcadC4EnoylCoAMAT → 1.08, KmmcadFADH → 24.2, Keqmcad → 6,
sfscadC6 → 0.3, sfscadC4 → 1, Vscad → 0.081, KmscadC6AcylCoAMAT → 285,
KmscadC4AcylCoAMAT → 10.7, KmscadFAD → 0.12, KmscadC6EnoylCoAMAT → 1.08,
KmscadC4EnoylCoAMAT → 1.08, KmscadFADH → 24.2, Keqscad -> 6,
sfcrotC16 → 0.13, sfcrotC14 → 0.2, sfcrotC12 → 0.25, sfcrotC10 → 0.33, sfcrotC8 → 0.58,
sfcrotC6 → 0.83, sfcrotC4 → 1, Vcrot → 3.6, KmcrotC16EnoylCoAMAT → 150,
KmcrotC14EnoylCoAMAT → 100, KmcrotC12EnoylCoAMAT → 25, KmcrotC10EnoylCoAMAT → 25,
KmcrotC8EnoylCoAMAT → 25, KmcrotC6EnoylCoAMAT → 25, KmcrotC4EnoylCoAMAT → 40,
KmcrotC16HydroxyacylCoAMAT → 45, KmcrotC14HydroxyacylCoAMAT → 45,
KmcrotC12HydroxyacylCoAMAT → 45, KmcrotC10HydroxyacylCoAMAT → 45,
KmcrotC8HydroxyacylCoAMAT → 45, KmcrotC6HydroxyacylCoAMAT → 45,
KmcrotC4HydroxyacylCoAMAT → 45, KicrotC4AcetoacylCoA → 1.6, Keqcrot → 3.13,
sfmschadC16 → 0.6, sfmschadC14 → 0.5, sfmschadC12 → 0.43, sfmschadC10 → 0.64,
sfmschadC8 → 0.89, sfmschadC6 → 1, sfmschadC4 → 0.67, Vmschad → 1,
KmmschadC16HydroxyacylCoAMAT → 1.5, KmmschadC14HydroxyacylCoAMAT → 1.8,
KmmschadC12HydroxyacylCoAMAT → 3.7, KmmschadC10HydroxyacylCoAMAT → 8.8,
KmmschadC8HydroxyacylCoAMAT → 16.3, KmmschadC6HydroxyacylCoAMAT → 28.6,
KmmschadC4HydroxyacylCoAMAT → 69.9, KmmschadNADMAT → 58.5,
KmmschadC16KetoacylCoAMAT → 1.4, KmmschadC14KetoacylCoAMAT → 1.4,
KmmschadC12KetoacylCoAMAT → 1.6, KmmschadC10KetoacylCoAMAT → 2.3,
KmmschadC8KetoacylCoAMAT → 4.1, KmmschadC6KetoacylCoAMAT → 5.8,
KmmschadC4AcetoacylCoAMAT → 16.9, KmmschadNADHMAT → 5.4, Keqmschad → 2.17 * 10^-4,
sfmckatC16 → 0, sfmckatC14 → 0.2, sfmckatC12 → 0.38, sfmckatC10 → 0.65,
sfmckatC8 → 0.81, sfmckatC6 → 1, sfmckatC4 → 0.49, Vmckat → 0.377,
KmmckatC16KetoacylCoAMAT → 1.1, KmmckatC14KetoacylCoAMAT → 1.2,
KmmckatC12KetoacylCoAMAT → 1.3, KmmckatC10KetoacylCoAMAT → 2.1,
KmmckatC8KetoacylCoAMAT → 3.2, KmmckatC6KetoacylCoAMAT → 6.7,
KmmckatC4AcetoacylCoAMAT → 12.4, KmmckatCoAMAT → 26.6,
KmmckatC14AcylCoAMAT → 13.83, KmmckatC16AcylCoAMAT → 13.83,
KmmckatC12AcylCoAMAT → 13.83, KmmckatC10AcylCoAMAT → 13.83,
KmmckatC8AcylCoAMAT → 13.83, KmmckatC6AcylCoAMAT → 13.83,
KmmckatC4AcylCoAMAT → 13.83, KmmckatAcetylCoAMAT → 30, Keqmckat → 1051,
sfmtpC16 → 1, sfmtpC14 → 0.9, sfmtpC12 → 0.81, sfmtpC10 → 0.73, sfmtpC8 → 0.34,
Vmtp → 2.84, KmmtpC16EnoylCoAMAT → 25, KmmtpC14EnoylCoAMAT → 25,
KmmtpC12EnoylCoAMAT → 25, KmmtpC10EnoylCoAMAT → 25, KmmtpC8EnoylCoAMAT → 25,
KmmtpNADMAT → 60, KmmtpCoAMAT → 30, KmmtpC14AcylCoAMAT → 13.83,
KmmtpC16AcylCoAMAT → 13.83, KmmtpC12AcylCoAMAT → 13.83,
KmmtpC10AcylCoAMAT → 13.83, KmmtpC8AcylCoAMAT → 13.83, KmmtpC6AcylCoAMAT → 13.83,
KmmtpNADHMAT → 50, KmmtpAcetylCoAMAT → 30, Keqmtp → 0.71,
Ksacesink → 6 000 000, K1acesink → 70, Ksfadhsink → 6 000 000,
K1fadhsink → 0.46, Ksnadhsink → 6 000 000, K1nadhsink → Z,
C16AcylCoACYT → X, CarCYT → 200, CoACYT → 140, MalCoACYT → 0,
CarMAT -> 950, FADtMAT → 0.77, NADtMAT → 250, CoAMATt → 5000,
VCYT → 2.2 * 10^-6, VMAT → 1.8 * 10^-6, AcetylCoAMAT → 70, FADHMAT → 0.46, NADHMAT → Z};
```

```
tsolScan[X_, Z_] :=
  NDSolve[Join[Odes /. RateEqs /. CoAMATX /. ParmScan[X, Z], InitialConditions],
   Vars, {t, 0, 1000000000}];

SsScan[X_, Z_] := Module[{SSGuess},
  SSGuess := Table[{Vars[[i]][t],
     (Vars[[i]][900000000] /. tsolScan[X, Z])[[1]]}, {i, 1, Length[Vars]}];
  FindRoot[Table[Odes[[i, 2]] == 0, {i, 1, Length[Odes]}] /. RateEqs /. CoAMATX /.
    ParmScan[X, Z], SSGuess]]

In[•]:= ScanDownNDS[Ystart_, dY_, Yend_] := Monitor[Module[{SS, SSGuess},
   DataDownNDSflux = {};
   Xstart = 250;
   Xend = 0;
   YY = {6., 11., 12., 13., 16., 19., 23.};
   For[Y = Ystart, Y ≤ Yend,
    Z = YY[[Y]];
    tsolStart = tsolScan[Xend, Z];
    SSGuess = Table[{Vars[[i]][t],
       (Vars[[i]][900000000] /. tsolStart)[[1]]}, {i, 1, Length[Vars]}];
    SSGuess1 = SSGuess[[All, 1]];
    SSGuess2 = SSGuess[[All, 2]];
    SSGuess1int = SSGuess1 /. t → 0;
    InitialConditionsUD = Thread[SSGuess1int == SSGuess2];
    dX = 1;
    For[X = 250, X ≥ 0,

     tsolScanNDS = NDSolve[Join[Odes /. RateEqs /. CoAMATX /. ParmScan[X, Z],
        InitialConditionsUD], Vars, {t, 0, 1000000000}];
     SSGuess = Table[{Vars[[i]][t], (Vars[[i]][900000000] /. tsolScanNDS)[[1]]},
       {i, 1, Length[Vars]}];
     SSGuess1 = SSGuess[[All, 1]];
     SSGuess2 = SSGuess[[All, 2]];
     SSGuess1int = SSGuess1 /. t → 0;
     InitialConditionsUD = Thread[SSGuess1int == SSGuess2];
     SS = Thread[SSGuess1 → SSGuess2];

     AppendTo[DataDownNDSflux,
      {X, Z, 10^3 vcpt1C16 /. RateEqs /. CoAMATX /. ParmScan[X, Z] /. SS}];
     X = X - dX;];
    Y = Y + dY;]
  ], ProgressIndicator[X, {Xstart, Xend}]]

In[•]:= ScanDownNDS[1, 1, 7]
```

```
In[•]:= ScanUpNDS[Ystart_, dY_, Yend_] := Monitor[Module[{SS, SSGuess},
    DataUpNDSflux = {};
    Xstart = 0;
    Xend = 250;
    YY = {6., 11., 12., 13., 16., 19., 23.};
    For[Y = Ystart, Y ≤ Yend,
     Z = YY[[Y]];
     tsolStart = tsolScan[Xstart, Z ];
     SSGuess = Table[{Vars[[i]][t],
        (Vars[[i]][900000000] /. tsolStart)[[1]]}, {i, 1, Length[Vars]}];
     SSGuess1 = SSGuess[[All, 1]];
     SSGuess2 = SSGuess[[All, 2]];
     SSGuess1int = SSGuess1 /. t → 0;
     InitialConditionsUD = Thread[SSGuess1int == SSGuess2];

     dX = 1;
     For[X = 0, X ≤ 250,

      tsolScanNDS = NDSolve[Join[Odes /. RateEqs /. CoAMATX /. ParmScan[X, Z],
         InitialConditionsUD], Vars, {t, 0, 1000000000}];
      SSGuess = Table[{Vars[[i]][t], (Vars[[i]][900000000] /. tsolScanNDS)[[1]]},
        {i, 1, Length[Vars]}];
      SSGuess1 = SSGuess[[All, 1]];
      SSGuess2 = SSGuess[[All, 2]];
      SSGuess1int = SSGuess1 /. t → 0;
      InitialConditionsUD = Thread[SSGuess1int == SSGuess2];
      SS = Thread[SSGuess1 → SSGuess2];

      AppendTo[DataUpNDSflux,
       {X, Z, 10^3 vcpt1C16 /. RateEqs /. CoAMATX /. ParmScan[X, Z] /. SS}];

      X = X + dX;];
     Y = Y + dY;]
   ], ProgressIndicator[X, {Xstart, Xend}]]
```

```
In[•]:= ScanUpNDS[1, 1, 7]
```

```
In[•]:= ReversedData = {DataDownNDSflux[[1 ;; 251, {1, 3}]],
    DataDownNDSflux[[252 ;; 502, {1, 3}]], DataDownNDSflux[[503 ;; 753, {1, 3}]],
    DataDownNDSflux[[754 ;; 1004, {1, 3}]], DataDownNDSflux[[1005 ;; 1255, {1, 3}]]};
```

```
In[•]:= ForwardData = {DataUpNDSflux[[1 ;; 251, {1, 3}]],
    DataUpNDSflux[[252 ;; 502, {1, 3}]], DataUpNDSflux[[503 ;; 753, {1, 3}]],
    DataUpNDSflux[[754 ;; 1004, {1, 3}]], DataUpNDSflux[[1005 ;; 1255, {1, 3}]]};
```

```
In[•]:=
```

```
In[•]:= (*Export[
   "C:\\Users\\Kindergeneeskunde\\Documents\\FentawNewlaptopPediatrics\\Code_mathematica
     \\mFAOvaryingNADNADHRatio.xls",
   {"forwardFlux" -> DataUpNDSfluxm, "reverseFlux" -> DataDownNDSfluxm}]*)
```

In[•]:=
```
p1 =
 ListLinePlot[{DataUpNDSflux[[1 ;; 251, {1, 3}]], DataUpNDSflux[[252 ;; 502, {1, 3}]],
   DataUpNDSflux[[503 ;; 753, {1, 3}]], DataUpNDSflux[[754 ;; 1004, {1, 3}]],
   DataUpNDSflux[[1005 ;; 1255, {1, 3}]], DataUpNDSflux[[1256 ;; 1506, {1, 3}]],
   DataUpNDSflux[[1507 ;; 1757, {1, 3}]], DataDownNDSflux[[1 ;; 251, {1, 3}]],
   DataDownNDSflux[[252 ;; 502, {1, 3}]], DataDownNDSflux[[503 ;; 753, {1, 3}]],
   DataDownNDSflux[[754 ;; 1004, {1, 3}]], DataDownNDSflux[[1005 ;; 1255, {1, 3}]] ,
   DataDownNDSflux[[1256 ;; 1506, {1, 3}]], DataDownNDSflux[[1507 ;; 1757, {1, 3}]]},
  PlotRange → All, PlotStyle → {Magenta, Blue, Cyan, Green, Darker[Yellow],
    Orange, Red, Magenta, Blue, Cyan, Green, Darker[Yellow], Orange, Red},
  AxesStyle → Directive[Black, 18], LabelStyle → Directive[Black, 18],
  PlotLegends → {"40", "23", "20", "18", "15", "12", "10"},
  PlotLabel → "NAD+:NADH Ratio",
  Frame → {{True, False}, {True, False}},
  FrameLabel → {{"Flux(μmol.min^-1.gProtein^-1)", None}, {"Palmitoyl-CoA(μM)", None}},
  BaseStyle → {FontSize → 18, FontWeight → ""},
  FrameStyle → Thickness[0.00005], ImageSize → Scaled[0.25], AspectRatio → 1]
```

Out[•]=

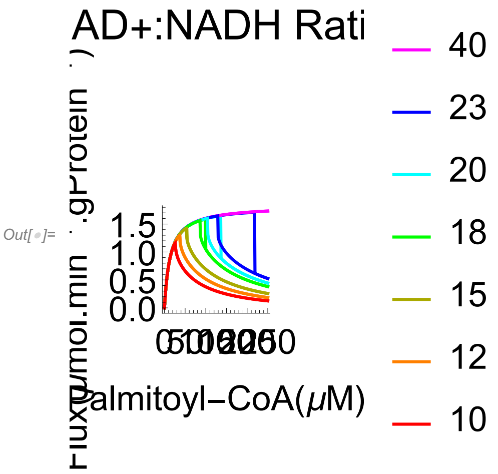

In[•]:=
```
sub1 = ListLinePlot[{DataUpNDSflux[[1 ;; 251, {1, 3}]],
    DataUpNDSflux[[252 ;; 502, {1, 3}]], DataUpNDSflux[[503 ;; 753, {1, 3}]],
    DataUpNDSflux[[754 ;; 1004, {1, 3}]], DataUpNDSflux[[1005 ;; 1255, {1, 3}]],
    DataUpNDSflux[[1256 ;; 1506, {1, 3}]], DataUpNDSflux[[1507 ;; 1757, {1, 3}]],
    DataDownNDSflux[[1 ;; 251, {1, 3}]], DataDownNDSflux[[252 ;; 502, {1, 3}]],
    DataDownNDSflux[[503 ;; 753, {1, 3}]], DataDownNDSflux[[754 ;; 1004, {1, 3}]],
    DataDownNDSflux[[1005 ;; 1255, {1, 3}]] , DataDownNDSflux[[1256 ;; 1506, {1, 3}]],
    DataDownNDSflux[[1507 ;; 1757, {1, 3}]]}, PlotRange → {{20, 60}, {0.5, 1.5}},
  PlotStyle → {Magenta, Blue, Cyan, Green, Darker[Yellow], Orange, Red, Magenta,
    Blue, Cyan, Green, Darker[Yellow], Orange, Red}, AxesStyle → Directive[Black, 18],
  LabelStyle → Directive[Black, 18], PlotLegends → {}, PlotLabel → " ",
  Frame → {{True, False}, {True, False}}, FrameLabel → {},
  BaseStyle → {FontSize → 18, FontWeight → ""},
  FrameStyle → Thickness[0.00005], ImageSize → Scaled[0.15], AspectRatio → 1]
NadnadhRatioVaryingMagnified = ListLinePlot[{DataUpNDSflux[[1 ;; 251, {1, 3}]],
    DataUpNDSflux[[252 ;; 502, {1, 3}]], DataUpNDSflux[[503 ;; 753, {1, 3}]],
    DataUpNDSflux[[754 ;; 1004, {1, 3}]], DataUpNDSflux[[1005 ;; 1255, {1, 3}]],
    DataUpNDSflux[[1256 ;; 1506, {1, 3}]], DataUpNDSflux[[1507 ;; 1757, {1, 3}]],
    DataDownNDSflux[[1 ;; 251, {1, 3}]], DataDownNDSflux[[252 ;; 502, {1, 3}]],
    DataDownNDSflux[[503 ;; 753, {1, 3}]], DataDownNDSflux[[754 ;; 1004, {1, 3}]],
    DataDownNDSflux[[1005 ;; 1255, {1, 3}]] , DataDownNDSflux[[1256 ;; 1506, {1, 3}]],
    DataDownNDSflux[[1507 ;; 1757, {1, 3}]]}, PlotRange → {{0, 250}, {0, 1.8}},
  PlotStyle → {Magenta, Blue, Cyan, Green, Darker[Yellow], Orange,
    Red, Magenta, Blue, Cyan, Green, Darker[Yellow], Orange, Red},
  AxesStyle → Directive[Black, 18], LabelStyle → Directive[Black, 18],
  PlotLegends → {"40", "23", "20", "19", "15", "13", "10"},
  PlotLabel → "NAD:NADH Ratio",
  Frame → {{True, False}, {True, False}},
  FrameLabel → {{"Flux(μmol.min^-1.gProtein^-1)", None}, {"Palmitoyl-CoA(μM)", None}},
  BaseStyle → {FontSize → 18, FontWeight → ""},
  FrameStyle → Thickness[0.00005], ImageSize → Scaled[0.25],
  AspectRatio → 1, Epilog → Inset[sub1], PlotRangeClipping → False]
```

Out[•]=

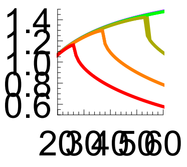

Out[•]=

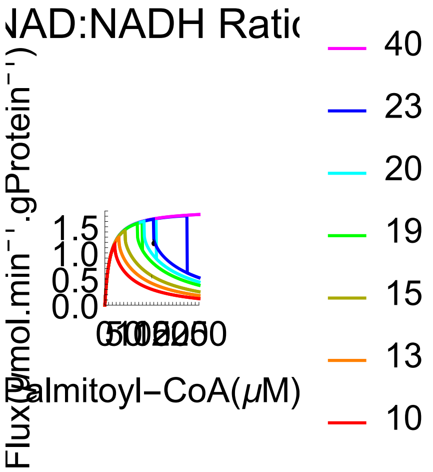

B. Steady state computation with varying palmitoyl-CoA (X) and Malonyl-CoA (Z)

In[ ]:=
```
ParmScan[X_, Z_] := {
    sfcpt1C16 → 1, Vcpt1 → 0.012, Kmcpt1C16AcylCoACYT → 13.8,
    Kmcpt1CarCYT → 250, Kmcpt1C16AcylCarCYT → 136, Kmcpt1CoACYT → 40.7,
    Kicpt1MalCoACYT → 9.1, Keqcpt1 → 0.45, ncpt1 → 2.4799,
    Vfcact → 0.42, Vrcact → 0.42, KmcactC16AcylCarCYT → 15,
    KmcactC14AcylCarCYT → 15, KmcactC12AcylCarCYT → 15, KmcactC10AcylCarCYT → 15,
    KmcactC8AcylCarCYT → 15, KmcactC6AcylCarCYT → 15, KmcactC4AcylCarCYT → 15,
    KmcactCarMAT → 130, KmcactC16AcylCarMAT → 15, KmcactC14AcylCarMAT → 15,
    KmcactC12AcylCarMAT → 15, KmcactC10AcylCarMAT → 15, KmcactC8AcylCarMAT → 15,
    KmcactC6AcylCarMAT → 15, KmcactC4AcylCarMAT → 15, KmcactCarCYT → 130,
    KicactC16AcylCarCYT → 56, KicactC14AcylCarCYT → 56, KicactC12AcylCarCYT → 56,
    KicactC10AcylCarCYT → 56, KicactC8AcylCarCYT → 56, KicactC6AcylCarCYT → 56,
    KicactC4AcylCarCYT → 56, KicactCarCYT → 200, Keqcact → 1,
    sfcpt2C16 → 0.85, sfcpt2C14 → 1, sfcpt2C12 → 0.95, sfcpt2C10 → 0.95,
    sfcpt2C8 → 0.35, sfcpt2C6 → 0.15, sfcpt2C4 → 0.01, Vcpt2 → 0.391,
    Kmcpt2C16AcylCarMAT → 51, Kmcpt2C14AcylCarMAT → 51, Kmcpt2C12AcylCarMAT → 51,
    Kmcpt2C10AcylCarMAT → 51, Kmcpt2C8AcylCarMAT → 51, Kmcpt2C6AcylCarMAT → 51,
    Kmcpt2C4AcylCarMAT → 51, Kmcpt2CoAMAT → 30, Kmcpt2C16AcylCoAMAT → 38,
    Kmcpt2C14AcylCoAMAT → 38, Kmcpt2C12AcylCoAMAT → 38,
    Kmcpt2C10AcylCoAMAT → 38, Kmcpt2C8AcylCoAMAT → 38, Kmcpt2C6AcylCoAMAT → 1000,
    Kmcpt2C4AcylCoAMAT → 1000000, Kmcpt2CarMAT → 350, Keqcpt2 → 2.22,
    sfvlcadC16 → 1, sfvlcadC14 → 0.42, sfvlcadC12 → 0.11, Vvlcad → 0.008,
    KmvlcadC16AcylCoAMAT → 6.5, KmvlcadC14AcylCoAMAT → 4, KmvlcadC12AcylCoAMAT → 2.7,
    KmvlcadFAD → 0.12, KmvlcadC16EnoylCoAMAT → 1.08, KmvlcadC14EnoylCoAMAT → 1.08,
    KmvlcadC12EnoylCoAMAT → 1.08, KmvlcadFADH → 24.2, Keqvlcad → 6,
    sflcadC16 → 0.9, sflcadC14 → 1, sflcadC12 → 0.9, sflcadC10 → 0.75, sflcadC8 → 0.4,
    Vlcad → 0.01, KmlcadC16AcylCoAMAT → 2.5, KmlcadC14AcylCoAMAT → 7.4,
    KmlcadC12AcylCoAMAT → 9, KmlcadC10AcylCoAMAT → 24.3, KmlcadC8AcylCoAMAT → 123,
    KmlcadFAD → 0.12, KmlcadC16EnoylCoAMAT → 1.08, KmlcadC14EnoylCoAMAT → 1.08,
    KmlcadC12EnoylCoAMAT → 1.08, KmlcadC10EnoylCoAMAT → 1.08,
    KmlcadC8EnoylCoAMAT → 1.08, KmlcadFADH → 24.2, Keqlcad → 6,
    sfmcadC12 → 0.38, sfmcadC10 → 0.8, sfmcadC8 → 0.87, sfmcadC6 → 1, sfmcadC4 → 0.12,
    Vmcad → 0.081, KmmcadC12AcylCoAMAT → 5.7, KmmcadC10AcylCoAMAT → 5.4,
    KmmcadC8AcylCoAMAT → 4, KmmcadC6AcylCoAMAT → 9.4, KmmcadC4AcylCoAMAT → 135,
    KmmcadFAD → 0.12, KmmcadC12EnoylCoAMAT → 1.08, KmmcadC10EnoylCoAMAT → 1.08,
    KmmcadC8EnoylCoAMAT → 1.08, KmmcadC6EnoylCoAMAT → 1.08,
    KmmcadC4EnoylCoAMAT → 1.08, KmmcadFADH → 24.2, Keqmcad → 6,
    sfscadC6 → 0.3, sfscadC4 → 1, Vscad → 0.081, KmscadC6AcylCoAMAT → 285,
    KmscadC4AcylCoAMAT → 10.7, KmscadFAD → 0.12, KmscadC6EnoylCoAMAT → 1.08,
    KmscadC4EnoylCoAMAT → 1.08, KmscadFADH → 24.2, Keqscad -> 6,
    sfcrotC16 → 0.13, sfcrotC14 → 0.2, sfcrotC12 → 0.25, sfcrotC10 → 0.33, sfcrotC8 → 0.58,
    sfcrotC6 → 0.83, sfcrotC4 → 1, Vcrot → 3.6, KmcrotC16EnoylCoAMAT → 150,
    KmcrotC14EnoylCoAMAT → 100, KmcrotC12EnoylCoAMAT → 25, KmcrotC10EnoylCoAMAT → 25,
    KmcrotC8EnoylCoAMAT → 25, KmcrotC6EnoylCoAMAT → 25, KmcrotC4EnoylCoAMAT → 40,
    KmcrotC16HydroxyacylCoAMAT → 45, KmcrotC14HydroxyacylCoAMAT → 45,
    KmcrotC12HydroxyacylCoAMAT → 45, KmcrotC10HydroxyacylCoAMAT → 45,
    KmcrotC8HydroxyacylCoAMAT → 45, KmcrotC6HydroxyacylCoAMAT → 45,
```

```
KmcrotC4HydroxyacylCoAMAT → 45, KicrotC4AcetoacylCoA → 1.6, Keqcrot → 3.13,
sfmschadC16 → 0.6, sfmschadC14 → 0.5, sfmschadC12 → 0.43, sfmschadC10 → 0.64,
sfmschadC8 → 0.89, sfmschadC6 → 1, sfmschadC4 → 0.67, Vmschad → 1,
KmmschadC16HydroxyacylCoAMAT → 1.5, KmmschadC14HydroxyacylCoAMAT → 1.8,
KmmschadC12HydroxyacylCoAMAT → 3.7, KmmschadC10HydroxyacylCoAMAT → 8.8,
KmmschadC8HydroxyacylCoAMAT → 16.3, KmmschadC6HydroxyacylCoAMAT → 28.6,
KmmschadC4HydroxyacylCoAMAT → 69.9, KmmschadNADMAT → 58.5,
KmmschadC16KetoacylCoAMAT → 1.4, KmmschadC14KetoacylCoAMAT → 1.4,
KmmschadC12KetoacylCoAMAT → 1.6, KmmschadC10KetoacylCoAMAT → 2.3,
KmmschadC8KetoacylCoAMAT → 4.1, KmmschadC6KetoacylCoAMAT → 5.8,
KmmschadC4AcetoacylCoAMAT → 16.9, KmmschadNADHMAT → 5.4, Keqmschad → 2.17 * 10^-4,
sfmckatC16 → 0, sfmckatC14 → 0.2, sfmckatC12 → 0.38, sfmckatC10 → 0.65,
sfmckatC8 → 0.81, sfmckatC6 → 1, sfmckatC4 → 0.49, Vmckat → 0.377,
KmmckatC16KetoacylCoAMAT → 1.1, KmmckatC14KetoacylCoAMAT → 1.2,
KmmckatC12KetoacylCoAMAT → 1.3, KmmckatC10KetoacylCoAMAT → 2.1,
KmmckatC8KetoacylCoAMAT → 3.2, KmmckatC6KetoacylCoAMAT → 6.7,
KmmckatC4AcetoacylCoAMAT → 12.4, KmmckatCoAMAT → 26.6,
KmmckatC14AcylCoAMAT → 13.83, KmmckatC16AcylCoAMAT → 13.83,
KmmckatC12AcylCoAMAT → 13.83, KmmckatC10AcylCoAMAT → 13.83,
KmmckatC8AcylCoAMAT → 13.83, KmmckatC6AcylCoAMAT → 13.83,
KmmckatC4AcylCoAMAT → 13.83, KmmckatAcetylCoAMAT → 30, Keqmckat → 1051,
sfmtpC16 → 1, sfmtpC14 → 0.9, sfmtpC12 → 0.81, sfmtpC10 → 0.73, sfmtpC8 → 0.34,
Vmtp → 2.84, KmmtpC16EnoylCoAMAT → 25, KmmtpC14EnoylCoAMAT → 25,
KmmtpC12EnoylCoAMAT → 25, KmmtpC10EnoylCoAMAT → 25, KmmtpC8EnoylCoAMAT → 25,
KmmtpNADMAT → 60, KmmtpCoAMAT → 30, KmmtpC14AcylCoAMAT → 13.83,
KmmtpC16AcylCoAMAT → 13.83, KmmtpC12AcylCoAMAT → 13.83,
KmmtpC10AcylCoAMAT → 13.83, KmmtpC8AcylCoAMAT → 13.83, KmmtpC6AcylCoAMAT → 13.83,
KmmtpNADHMAT → 50, KmmtpAcetylCoAMAT → 30, Keqmtp → 0.71,
Ksacesink → 6000000, K1acesink → 70, Ksfadhsink → 6000000,
K1fadhsink → 0.46, Ksnadhsink → 6000000, K1nadhsink → 12,
C16AcylCoACYT → X, CarCYT → 200, CoACYT → 140, MalCoACYT → Z,
CarMAT -> 950, FADtMAT → 0.77, NADtMAT → 250, CoAMATt → 5000,
VCYT → 2.2 * 10^-6, VMAT → 1.8 * 10^-6, AcetylCoAMAT → 70, FADHMAT → 0.46, NADHMAT → 12};

tsolScan[X_, Z_] :=
  NDSolve[Join[Odes /. RateEqs /. CoAMATX /. ParmScan[X, Z], InitialConditions],
   Vars, {t, 0, 1000000000}];

SsScan[X_, Z_] := Module[{SSGuess},
  SSGuess := Table[{Vars[[i]][t],
     (Vars[[i]][900000000] /. tsolScan[X, Z])[[1]]}, {i, 1, Length[Vars]}];
  FindRoot[Table[Odes[[i, 2]] == 0, {i, 1, Length[Odes]}] /. RateEqs /. CoAMATX /.
    ParmScan[X, Z], SSGuess]]
```

In[•]:=
```
ScanDownNDSm[Ystart_, dY_, Yend_] := Monitor[Module[{SS, SSGuess},
   DataDownNDSfluxm = {};
   Xstart = 250;
   Xend = 0;
   YY = {0., 3., 6., 8., 9.};
   For[Y = Ystart, Y ≤ Yend,
    Z = YY[[Y]];
    tsolStart = tsolScan[Xend, Z];
    SSGuess = Table[{Vars[[i]][t],
       (Vars[[i]][900 000 000] /. tsolStart)[[1]]}, {i, 1, Length[Vars]}];
    SSGuess1 = SSGuess[[All, 1]];
    SSGuess2 = SSGuess[[All, 2]];
    SSGuess1int = SSGuess1 /. t → 0;
    InitialConditionsUD = Thread[SSGuess1int == SSGuess2];
    dX = 1;
    For[X = 250, X ≥ 0,
     (*Print[X];*)
     (*SS=FindRoot[Table[Odes[[i,2]]==0,{i,1,Length[Odes]}]/.RateEqs/.CoAMATX/.
        ParmScan[X],SSGuess,MaxIterations→Infinity];
     SSGuess={#[[1]],#[[2]]}&/@SS;*)


     tsolScanNDS = NDSolve[Join[Odes /. RateEqs /. CoAMATX /. ParmScan[X, Z],
        InitialConditionsUD], Vars, {t, 0, 1 000 000 000}];
     SSGuess = Table[{Vars[[i]][t], (Vars[[i]][900 000 000] /. tsolScanNDS)[[1]]},
       {i, 1, Length[Vars]}];
     SSGuess1 = SSGuess[[All, 1]];
     SSGuess2 = SSGuess[[All, 2]];
     SSGuess1int = SSGuess1 /. t → 0;
     InitialConditionsUD = Thread[SSGuess1int == SSGuess2];
     SS = Thread[SSGuess1 → SSGuess2];

     AppendTo[DataDownNDSfluxm,
      {X, Z, 10^3 vcpt1C16 /. RateEqs /. CoAMATX /. ParmScan[X, Z] /. SS}];
     X = X - dX;];
    Y = Y + dY;]
  ], ProgressIndicator[X, {Xstart, Xend}]]
```

In[•]:=
```
ScanDownNDSm[1, 1, 5]
```

```
In[•]:= ScanUpNDSm[Ystart_, dY_, Yend_] := Monitor[Module[{SS, SSGuess},
    DataUpNDSfluxm = {};
    Xstart = 0;
    Xend = 250;
    YY = {0., 3., 6., 8., 9.};
    For[Y = Ystart, Y ≤ Yend,
     Z = YY[[Y]];
     tsolStart = tsolScan[Xstart, Z ];
     SSGuess = Table[{Vars[[i]][t],
        (Vars[[i]][900000000] /. tsolStart)[[1]]}, {i, 1, Length[Vars]}];
     SSGuess1 = SSGuess[[All, 1]];
     SSGuess2 = SSGuess[[All, 2]];
     SSGuess1int = SSGuess1 /. t → 0;
     InitialConditionsUD = Thread[SSGuess1int == SSGuess2];

     dX = 1;
     For[X = 0, X ≤ 250,
      (*Print[X];*)
      (*SS=FindRoot[Table[Odes[[i,2]]==0,{i,1,Length[Odes]}]/.RateEqs/.CoAMATX/.
         ParmScan[X],SSGuess,MaxIterations→Infinity];
      SSGuess={#[[1]],#[[2]]}&/@SS;*)


      tsolScanNDS = NDSolve[Join[Odes /. RateEqs /. CoAMATX /. ParmScan[X, Z],
         InitialConditionsUD], Vars, {t, 0, 1000000000}];
      SSGuess = Table[{Vars[[i]][t], (Vars[[i]][900000000] /. tsolScanNDS)[[1]]},
        {i, 1, Length[Vars]}];
      SSGuess1 = SSGuess[[All, 1]];
      SSGuess2 = SSGuess[[All, 2]];
      SSGuess1int = SSGuess1 /. t → 0;
      InitialConditionsUD = Thread[SSGuess1int == SSGuess2];
      SS = Thread[SSGuess1 → SSGuess2];

      AppendTo[DataUpNDSfluxm,
       {X, Z, 10^3 vcpt1C16 /. RateEqs /. CoAMATX /. ParmScan[X, Z] /. SS}];

      X = X + dX;];
     Y = Y + dY;]
   ], ProgressIndicator[X, {Xstart, Xend}]]


In[•]:= ScanUpNDSm[1, 1, 5]

In[•]:=

In[•]:=

In[•]:= (*Export[
  "C:\\Users\\Kindergeneeskunde\\Documents\\FentawNewlaptopPediatrics\\Code_mathematica
    \\mFAOvaryingMalonylCoA.xls",
  {"forwardFlux" -> DataUpNDSfluxm, "reverseFlux" -> DataDownNDSfluxm}]*)
```

In[•]:=
```
p2 =
 ListLinePlot[{DataUpNDSfluxm[[1 ;; 251, {1, 3}]], DataUpNDSfluxm[[252 ;; 502, {1, 3}]],
   DataUpNDSfluxm[[503 ;; 753, {1, 3}]], DataUpNDSfluxm[[754 ;; 1004, {1, 3}]],
   DataUpNDSfluxm[[1005 ;; 1255, {1, 3}]], DataDownNDSfluxm[[1 ;; 251, {1, 3}]],
   DataDownNDSfluxm[[252 ;; 502, {1, 3}]], DataDownNDSfluxm[[503 ;; 753, {1, 3}]],
   DataDownNDSfluxm[[754 ;; 1004, {1, 3}]], DataDownNDSfluxm[[1005 ;; 1255, {1, 3}]] },
  PlotRange → All, PlotStyle → {Magenta, Blue, Cyan, Green,
    Darker[Yellow], Magenta, Blue, Cyan, Green, Darker[Yellow]},
  AxesStyle → Directive[Black, 18], LabelStyle → Directive[Black, 18],
  PlotLegends → {"0", "3", "6", "8", "9"}, PlotLabel → "Malonyl-CoA",
  Frame → {{True, False}, {True, False}},
  FrameLabel → {{"Flux(μmol.min^-1.gProtein^-1)", None}, {"Palmitoyl-CoA(μM)", None}},
  BaseStyle → {FontSize → 18, FontWeight → ""},
  FrameStyle → Thickness[0.00005], ImageSize → Scaled[0.25], AspectRatio → 1]
```

Out[•]=
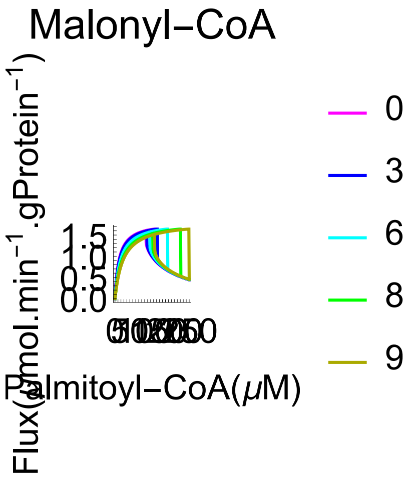

C. Steady state computation with varying palmitoyl-CoA (X) and total CoA pool (Z)

In[•]:=
```
ParmScan[X_, Z_] := {
   sfcpt1C16 → 1, Vcpt1 → 0.012, Kmcpt1C16AcylCoACYT → 13.8,
   Kmcpt1CarCYT → 250, Kmcpt1C16AcylCarCYT → 136, Kmcpt1CoACYT → 40.7,
   Kicpt1MalCoACYT → 9.1, Keqcpt1 → 0.45, ncpt1 → 2.4799,
   Vfcact → 0.42, Vrcact → 0.42, KmcactC16AcylCarCYT → 15,
   KmcactC14AcylCarCYT → 15, KmcactC12AcylCarCYT → 15, KmcactC10AcylCarCYT → 15,
   KmcactC8AcylCarCYT → 15, KmcactC6AcylCarCYT → 15, KmcactC4AcylCarCYT → 15,
   KmcactCarMAT → 130, KmcactC16AcylCarMAT → 15, KmcactC14AcylCarMAT → 15,
   KmcactC12AcylCarMAT → 15, KmcactC10AcylCarMAT → 15, KmcactC8AcylCarMAT → 15,
   KmcactC6AcylCarMAT → 15, KmcactC4AcylCarMAT → 15, KmcactCarCYT → 130,
   KicactC16AcylCarCYT → 56, KicactC14AcylCarCYT → 56, KicactC12AcylCarCYT → 56,
   KicactC10AcylCarCYT → 56, KicactC8AcylCarCYT → 56, KicactC6AcylCarCYT → 56,
   KicactC4AcylCarCYT → 56, KicactCarCYT → 200, Keqcact → 1,
   sfcpt2C16 → 0.85, sfcpt2C14 → 1, sfcpt2C12 → 0.95, sfcpt2C10 → 0.95,
   sfcpt2C8 → 0.35, sfcpt2C6 → 0.15, sfcpt2C4 → 0.01, Vcpt2 → 0.391,
   Kmcpt2C16AcylCarMAT → 51, Kmcpt2C14AcylCarMAT → 51, Kmcpt2C12AcylCarMAT → 51,
   Kmcpt2C10AcylCarMAT → 51, Kmcpt2C8AcylCarMAT → 51, Kmcpt2C6AcylCarMAT → 51,
   Kmcpt2C4AcylCarMAT → 51, Kmcpt2CoAMAT → 30, Kmcpt2C16AcylCoAMAT → 38,
   Kmcpt2C14AcylCoAMAT → 38, Kmcpt2C12AcylCoAMAT → 38,
   Kmcpt2C10AcylCoAMAT → 38, Kmcpt2C8AcylCoAMAT → 38, Kmcpt2C6AcylCoAMAT → 1000,
```

```
Kmcpt2C4AcylCoAMAT → 1000000, Kmcpt2CarMAT → 350, Keqcpt2 → 2.22,
sfvlcadC16 → 1, sfvlcadC14 → 0.42, sfvlcadC12 → 0.11, Vvlcad → 0.008,
KmvlcadC16AcylCoAMAT → 6.5, KmvlcadC14AcylCoAMAT → 4, KmvlcadC12AcylCoAMAT → 2.7,
KmvlcadFAD → 0.12, KmvlcadC16EnoylCoAMAT → 1.08, KmvlcadC14EnoylCoAMAT → 1.08,
KmvlcadC12EnoylCoAMAT → 1.08, KmvlcadFADH → 24.2, Keqvlcad → 6,
sflcadC16 → 0.9, sflcadC14 → 1, sflcadC12 → 0.9, sflcadC10 → 0.75, sflcadC8 → 0.4,
Vlcad → 0.01, KmlcadC16AcylCoAMAT → 2.5, KmlcadC14AcylCoAMAT → 7.4,
KmlcadC12AcylCoAMAT → 9, KmlcadC10AcylCoAMAT → 24.3, KmlcadC8AcylCoAMAT → 123,
KmlcadFAD → 0.12, KmlcadC16EnoylCoAMAT → 1.08, KmlcadC14EnoylCoAMAT → 1.08,
KmlcadC12EnoylCoAMAT → 1.08, KmlcadC10EnoylCoAMAT → 1.08,
KmlcadC8EnoylCoAMAT → 1.08, KmlcadFADH → 24.2, Keqlcad → 6,
sfmcadC12 → 0.38, sfmcadC10 → 0.8, sfmcadC8 → 0.87, sfmcadC6 → 1, sfmcadC4 → 0.12,
Vmcad → 0.081, KmmcadC12AcylCoAMAT → 5.7, KmmcadC10AcylCoAMAT → 5.4,
KmmcadC8AcylCoAMAT → 4, KmmcadC6AcylCoAMAT → 9.4, KmmcadC4AcylCoAMAT → 135,
KmmcadFAD → 0.12, KmmcadC12EnoylCoAMAT → 1.08, KmmcadC10EnoylCoAMAT → 1.08,
KmmcadC8EnoylCoAMAT → 1.08, KmmcadC6EnoylCoAMAT → 1.08,
KmmcadC4EnoylCoAMAT → 1.08, KmmcadFADH → 24.2, Keqmcad → 6,
sfscadC6 → 0.3, sfscadC4 → 1, Vscad → 0.081, KmscadC6AcylCoAMAT → 285,
KmscadC4AcylCoAMAT → 10.7, KmscadFAD → 0.12, KmscadC6EnoylCoAMAT → 1.08,
KmscadC4EnoylCoAMAT → 1.08, KmscadFADH → 24.2, Keqscad -> 6,
sfcrotC16 → 0.13, sfcrotC14 → 0.2, sfcrotC12 → 0.25, sfcrotC10 → 0.33, sfcrotC8 → 0.58,
sfcrotC6 → 0.83, sfcrotC4 → 1, Vcrot → 3.6, KmcrotC16EnoylCoAMAT → 150,
KmcrotC14EnoylCoAMAT → 100, KmcrotC12EnoylCoAMAT → 25, KmcrotC10EnoylCoAMAT → 25,
KmcrotC8EnoylCoAMAT → 25, KmcrotC6EnoylCoAMAT → 25, KmcrotC4EnoylCoAMAT → 40,
KmcrotC16HydroxyacylCoAMAT → 45, KmcrotC14HydroxyacylCoAMAT → 45,
KmcrotC12HydroxyacylCoAMAT → 45, KmcrotC10HydroxyacylCoAMAT → 45,
KmcrotC8HydroxyacylCoAMAT → 45, KmcrotC6HydroxyacylCoAMAT → 45,
KmcrotC4HydroxyacylCoAMAT → 45, KicrotC4AcetoacylCoA → 1.6, Keqcrot → 3.13,
sfmschadC16 → 0.6, sfmschadC14 → 0.5, sfmschadC12 → 0.43, sfmschadC10 → 0.64,
sfmschadC8 → 0.89, sfmschadC6 → 1, sfmschadC4 → 0.67, Vmschad → 1,
KmmschadC16HydroxyacylCoAMAT → 1.5, KmmschadC14HydroxyacylCoAMAT → 1.8,
KmmschadC12HydroxyacylCoAMAT → 3.7, KmmschadC10HydroxyacylCoAMAT → 8.8,
KmmschadC8HydroxyacylCoAMAT → 16.3, KmmschadC6HydroxyacylCoAMAT → 28.6,
KmmschadC4HydroxyacylCoAMAT → 69.9, KmmschadNADMAT → 58.5,
KmmschadC16KetoacylCoAMAT → 1.4, KmmschadC14KetoacylCoAMAT → 1.4,
KmmschadC12KetoacylCoAMAT → 1.6, KmmschadC10KetoacylCoAMAT → 2.3,
KmmschadC8KetoacylCoAMAT → 4.1, KmmschadC6KetoacylCoAMAT → 5.8,
KmmschadC4AcetoacylCoAMAT → 16.9, KmmschadNADHMAT → 5.4, Keqmschad → 2.17 * 10^-4,
sfmckatC16 → 0, sfmckatC14 → 0.2, sfmckatC12 → 0.38, sfmckatC10 → 0.65,
sfmckatC8 → 0.81, sfmckatC6 → 1, sfmckatC4 → 0.49, Vmckat → 0.377,
KmmckatC16KetoacylCoAMAT → 1.1, KmmckatC14KetoacylCoAMAT → 1.2,
KmmckatC12KetoacylCoAMAT → 1.3, KmmckatC10KetoacylCoAMAT → 2.1,
KmmckatC8KetoacylCoAMAT → 3.2, KmmckatC6KetoacylCoAMAT → 6.7,
KmmckatC4AcetoacylCoAMAT → 12.4, KmmckatCoAMAT → 26.6,
KmmckatC14AcylCoAMAT → 13.83, KmmckatC16AcylCoAMAT → 13.83,
KmmckatC12AcylCoAMAT → 13.83, KmmckatC10AcylCoAMAT → 13.83,
KmmckatC8AcylCoAMAT → 13.83, KmmckatC6AcylCoAMAT → 13.83,
KmmckatC4AcylCoAMAT → 13.83, KmmckatAcetylCoAMAT → 30, Keqmckat → 1051,
sfmtpC16 → 1, sfmtpC14 → 0.9, sfmtpC12 → 0.81, sfmtpC10 → 0.73, sfmtpC8 → 0.34,
Vmtp → 2.84, KmmtpC16EnoylCoAMAT → 25, KmmtpC14EnoylCoAMAT → 25,
KmmtpC12EnoylCoAMAT → 25, KmmtpC10EnoylCoAMAT → 25, KmmtpC8EnoylCoAMAT → 25,
KmmtpNADMAT → 60, KmmtpCoAMAT → 30, KmmtpC14AcylCoAMAT → 13.83,
KmmtpC16AcylCoAMAT → 13.83, KmmtpC12AcylCoAMAT → 13.83,
KmmtpC10AcylCoAMAT → 13.83, KmmtpC8AcylCoAMAT → 13.83, KmmtpC6AcylCoAMAT → 13.83,
KmmtpNADHMAT → 50, KmmtpAcetylCoAMAT → 30, Keqmtp → 0.71,
```

```
    Ksacesink → 6 000 000, K1acesink → 70, Ksfadhsink → 6 000 000,
    K1fadhsink → 0.46, Ksnadhsink → 6 000 000, K1nadhsink → 12,
    C16AcylCoACYT → X, CarCYT → 200, CoACYT → 140, MalCoACYT → 0,
    CarMAT -> 950, FADtMAT → 0.77, NADtMAT → 250, CoAMATt → Z,
    VCYT → 2.2 * 10^-6, VMAT → 1.8 * 10^-6, AcetylCoAMAT → 70, FADHMAT → 0.46, NADHMAT → 12};

tsolScan[X_, Z_] :=
  NDSolve[Join[Odes /. RateEqs /. CoAMATX /. ParmScan[X, Z], InitialConditions],
   Vars, {t, 0, 1 000 000 000}];

SsScan[X_, Z_] := Module[{SSGuess},
  SSGuess := Table[{Vars[[i]][t],
     (Vars[[i]][900 000 000] /. tsolScan[X, Z])[[1]]}, {i, 1, Length[Vars]}];
  FindRoot[Table[Odes[[i, 2]] == 0, {i, 1, Length[Odes]}] /. RateEqs /. CoAMATX /.
    ParmScan[X, Z], SSGuess]]

In[•]:= ScanDownNDSc[Ystart_, dY_, Yend_] := Monitor[Module[{SS, SSGuess},
    DataDownNDSfluxc = {};

    DataDownNDSc4coa = {};
    DataDownNDSc6coa = {};
    DataDownNDSc4c6coa = {};
    DataDownNDScintermedcoa = {};
    DataDownNDSfreecoa = {};
    Xstart = 250;
    Xend = 0;
    YY = {2500., 3500., 5000., 7500., 10 000.};
    For[Y = Ystart, Y ≤ Yend,
     Z = YY[[Y]];
     tsolStart = tsolScan[Xend, Z];
     SSGuess = Table[{Vars[[i]][t],
        (Vars[[i]][900 000 000] /. tsolStart)[[1]]}, {i, 1, Length[Vars]}];
     SSGuess1 = SSGuess[[All, 1]];
     SSGuess2 = SSGuess[[All, 2]];
     SSGuess1int = SSGuess1 /. t → 0;
     InitialConditionsUD = Thread[SSGuess1int == SSGuess2];
     dX = 1;
     For[X = 250, X ≥ 0,

      tsolScanNDS = NDSolve[Join[Odes /. RateEqs /. CoAMATX /. ParmScan[X, Z],
         InitialConditionsUD], Vars, {t, 0, 1 000 000 000}];
      SSGuess = Table[{Vars[[i]][t], (Vars[[i]][900 000 000] /. tsolScanNDS)[[1]]},
        {i, 1, Length[Vars]}];
      SSGuess1 = SSGuess[[All, 1]];
      SSGuess2 = SSGuess[[All, 2]];
      SSGuess1int = SSGuess1 /. t → 0;
      InitialConditionsUD = Thread[SSGuess1int == SSGuess2];
      SS = Thread[SSGuess1 → SSGuess2];

      C4CoA = C4AcylCoAMAT[t] + C4EnoylCoAMAT[t] +
         C4HydroxyacylCoAMAT[t] + C4AcetoacylCoAMAT[t] /. SS;
      C6CoA = C6AcylCoAMAT[t] + C6EnoylCoAMAT[t] + C6HydroxyacylCoAMAT[t] +
         C6KetoacylCoAMAT[t] /. SS;
      C4C6CoAs = C4CoA + C6CoA;
      IntermediateCoAs = C4AcylCoAMAT[t] + C4EnoylCoAMAT[t] +
```

```
        C4HydroxyacylCoAMAT[t] + C4AcetoacylCoAMAT[t] + C6AcylCoAMAT[t] +
        C6EnoylCoAMAT[t] + C6HydroxyacylCoAMAT[t] + C6KetoacylCoAMAT[t] +
        C8AcylCoAMAT[t] + C8EnoylCoAMAT[t] + C8HydroxyacylCoAMAT[t] +
        C8KetoacylCoAMAT[t] + C10AcylCoAMAT[t] + C10EnoylCoAMAT[t] +
        C10HydroxyacylCoAMAT[t] + C10KetoacylCoAMAT[t] + C12AcylCoAMAT[t] +
        C12EnoylCoAMAT[t] + C12HydroxyacylCoAMAT[t] + C12KetoacylCoAMAT[t] +
        C14AcylCoAMAT[t] + C14EnoylCoAMAT[t] + C14HydroxyacylCoAMAT[t] +
        C14KetoacylCoAMAT[t] + C16AcylCoAMAT[t] + C16EnoylCoAMAT[t] +
        C16HydroxyacylCoAMAT[t] + C16KetoacylCoAMAT[t]  /. SS;
     FreeCoA = CoAMATt - IntermediateCoAs - 70  /. CoAMATX /. ParmScan[X, Z] /. SS;

     AppendTo[DataDownNDSfluxc,
       {X, Z, 10^3 vcpt1C16 /.  RateEqs /. CoAMATX /. ParmScan[X, Z] /. SS}];
     AppendTo[DataDownNDSc4coa, {X, Z, C4CoA}];
     AppendTo[DataDownNDSc6coa, {X, Z, C6CoA}];
     AppendTo[DataDownNDSc4c6coa, {X, Z, C4C6CoAs}];
     AppendTo[DataDownNDScintermedcoa, {X, Z, IntermediateCoAs}];
     AppendTo[DataDownNDSfreecoa, {X, Z, FreeCoA}];

     X = X - dX;];
    Y = Y + dY;]
  ], ProgressIndicator[X, {Xstart, Xend}]]
```

In[∘]:=
```
ScanDownNDSc[1, 1, 5]
```

In[∘]:=
```
ScanUpNDSc[Ystart_, dY_, Yend_] := Monitor[Module[{SS, SSGuess},
   DataUpNDSfluxc = {};
   DataUpNDSc4coa = {};
   DataUpNDSc6coa = {};
   DataUpNDSc4c6coa = {};
   DataUpNDScintermedcoa = {};
   DataUpNDSfreecoa = {};

   Xstart = 0;
   Xend = 250;
   YY = {2500., 3500., 5000., 7500., 10000.};
   For[Y = Ystart, Y ≤  Yend,
    Z = YY[[Y]];
    tsolStart = tsolScan[Xstart, Z ];
    SSGuess = Table[{Vars[[i]][t],
       (Vars[[i]][900000000] /. tsolStart)[[1]]}, {i, 1, Length[Vars]}];
    SSGuess1 = SSGuess[[All, 1]];
    SSGuess2 = SSGuess[[All, 2]];
    SSGuess1int = SSGuess1 /. t → 0;
    InitialConditionsUD = Thread[SSGuess1int == SSGuess2];

    dX = 1;
    For[X = 0, X ≤  250,
```

```
      tsolScanNDS = NDSolve[Join[Odes /. RateEqs /. CoAMATX /. ParmScan[X, Z],
          InitialConditionsUD], Vars, {t, 0, 1000000000}];
      SSGuess = Table[{Vars[[i]][t], (Vars[[i]][900000000] /. tsolScanNDS)[[1]]},
         {i, 1, Length[Vars]}];
      SSGuess1 = SSGuess[[All, 1]];
      SSGuess2 = SSGuess[[All, 2]];
      SSGuess1int = SSGuess1 /. t → 0;
      InitialConditionsUD = Thread[SSGuess1int == SSGuess2];
      SS = Thread[SSGuess1 → SSGuess2];

      (*AppendTo[DataUpNDSfluxc,
         {X,Z,10^3vcpt1C16/. RateEqs/.CoAMATX/.ParmScan[X,Z]/.SS}];*)
      C4CoA = C4AcylCoAMAT[t] + C4EnoylCoAMAT[t] + C4HydroxyacylCoAMAT[t] +
          C4AcetoacylCoAMAT[t] /. SS;
      C6CoA = C6AcylCoAMAT[t] + C6EnoylCoAMAT[t] + C6HydroxyacylCoAMAT[t] +
          C6KetoacylCoAMAT[t] /. SS;
      C4C6CoAs = C4CoA + C6CoA;
      IntermediateCoAs = C4AcylCoAMAT[t] + C4EnoylCoAMAT[t] +
          C4HydroxyacylCoAMAT[t] + C4AcetoacylCoAMAT[t] + C6AcylCoAMAT[t] +
          C6EnoylCoAMAT[t] + C6HydroxyacylCoAMAT[t] + C6KetoacylCoAMAT[t] +
          C8AcylCoAMAT[t] + C8EnoylCoAMAT[t] + C8HydroxyacylCoAMAT[t] +
          C8KetoacylCoAMAT[t] + C10AcylCoAMAT[t] + C10EnoylCoAMAT[t] +
          C10HydroxyacylCoAMAT[t] + C10KetoacylCoAMAT[t] + C12AcylCoAMAT[t] +
          C12EnoylCoAMAT[t] + C12HydroxyacylCoAMAT[t] + C12KetoacylCoAMAT[t] +
          C14AcylCoAMAT[t] + C14EnoylCoAMAT[t] + C14HydroxyacylCoAMAT[t] +
          C14KetoacylCoAMAT[t] + C16AcylCoAMAT[t] + C16EnoylCoAMAT[t] +
          C16HydroxyacylCoAMAT[t] + C16KetoacylCoAMAT[t] /. SS;
      FreeCoA = CoAMATt - IntermediateCoAs - 70 /. CoAMATX /. ParmScan[X, Z] /. SS;

      AppendTo[DataUpNDSfluxc,
        {X, Z, 10^3 vcpt1C16 /. RateEqs /. CoAMATX /. ParmScan[X, Z] /. SS}];
      AppendTo[DataUpNDSc4coa, {X, Z, C4CoA}];
      AppendTo[DataUpNDSc6coa, {X, Z, C6CoA}];
      AppendTo[DataUpNDSc4c6coa, {X, Z, C4C6CoAs}];
      AppendTo[DataUpNDScintermedcoa, {X, Z, IntermediateCoAs}];
      AppendTo[DataUpNDSfreecoa, {X, Z, FreeCoA}];

      X = X + dX;];
    Y = Y + dY;]
  ], ProgressIndicator[X, {Xstart, Xend}]]
```

In[•]:=
```
ScanUpNDSc[1, 1, 5]
```

In[•]:=
```
(*Export[
  "C:\\Users\\Kindergeneeskunde\\Documents\\FentawNewlaptopPediatrics\\Code_mathematica
    \\mFAOvaryingCoAPool.xls",
  {"forwardFlux" -> DataUpNDSfluxc, "reverseFlux" -> DataDownNDSfluxc}]*)
```

In[ ]:=
```
p3 =
 ListLinePlot[{DataUpNDSfluxc[[1 ;; 251, {1, 3}]], DataUpNDSfluxc[[252 ;; 502, {1, 3}]],
   DataUpNDSfluxc[[503 ;; 753, {1, 3}]], DataUpNDSfluxc[[754 ;; 1004, {1, 3}]],
   DataUpNDSfluxc[[1005 ;; 1255, {1, 3}]], DataDownNDSfluxc[[1 ;; 251, {1, 3}]],
   DataDownNDSfluxc[[252 ;; 502, {1, 3}]], DataDownNDSfluxc[[503 ;; 753, {1, 3}]],
   DataDownNDSfluxc[[754 ;; 1004, {1, 3}]], DataDownNDSfluxc[[1005 ;; 1255, {1, 3}]] },
  PlotRange → All, PlotStyle → {Magenta, Blue, Cyan, Green,
    Darker[Yellow], Magenta, Blue, Cyan, Green, Darker[Yellow]},
  AxesStyle → Directive[Black, 18], LabelStyle → Directive[Black, 18],
  PlotLegends → {"2500", "3500", "5000", "7500", "10000"} ,
  PlotLabel → "Total CoA Pool",
  Frame → {{True, False}, {True, False}},
  FrameLabel → {{"Flux(μmol.min^-1.gProtein^-1)", None}, {"Palmitoyl-CoA(μM)", None}},
  BaseStyle → {FontSize → 18, FontWeight → ""},
  FrameStyle → Thickness[0.00005], ImageSize → Scaled[0.25], AspectRatio → 1]
```

Out[ ]=
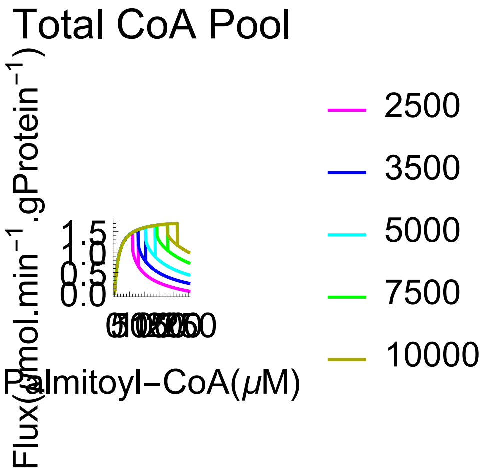

In[ ]:=

In[∘]:=
```
p3coa1 =
 ListLinePlot[{DataUpNDSc4coa[[1 ;; 251, {1, 3}]], DataUpNDSc6coa[[1 ;; 251, {1, 3}]],
   DataUpNDSc4c6coa[[1 ;; 251, {1, 3}]], DataUpNDScintermedcoa[[1 ;; 251, {1, 3}]],
   DataUpNDSfreecoa[[1 ;; 251, {1, 3}]]}, PlotRange → {{0, 250}, {0, 10000}},
  PlotStyle → {Magenta, Blue, Cyan, Green, Darker[Yellow], Magenta, Blue,
    Cyan, Green, Darker[Yellow]}, AxesStyle → Directive[Black, 18],
  LabelStyle → Directive[Black, 18], Frame → {{True, False}, {True, False}},
  FrameLabel → {{"CoA Esters(μM) )", None}, {"Palmitoyl-CoA(μM)", None}},
  BaseStyle → {FontSize → 18, FontWeight → ""},
  FrameStyle → Thickness[0.00005], ImageSize → Scaled[0.25]]
```

Out[∘]=
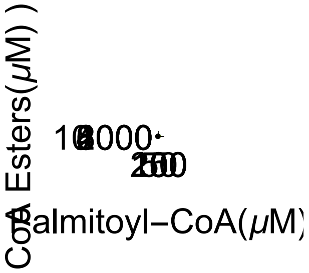

In[∘]:=
```
p3coa2 = ListLinePlot[
  {DataUpNDSc4coa[[252 ;; 502, {1, 3}]], DataUpNDSc6coa[[252 ;; 502 , {1, 3}]],
   DataUpNDSc4c6coa[[252 ;; 502, {1, 3}]], DataUpNDScintermedcoa[[252 ;; 502, {1, 3}]],
   DataUpNDSfreecoa[[252 ;; 502, {1, 3}]]}, PlotRange → {{0, 250}, {0, 10000}},
  PlotStyle → {Magenta, Blue, Cyan, Green, Darker[Yellow], Magenta, Blue,
    Cyan, Green, Darker[Yellow]}, AxesStyle → Directive[Black, 18],
  LabelStyle → Directive[Black, 18], Frame → {{True, False}, {True, False}},
  FrameLabel → {{"CoA Esters(μM) )", None}, {"Palmitoyl-CoA(μM)", None}},
  BaseStyle → {FontSize → 18, FontWeight → ""},
  FrameStyle → Thickness[0.00005], ImageSize → Scaled[0.25]]
```

Out[∘]=
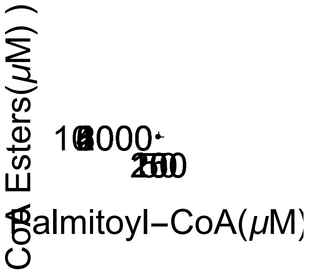

In[•]:=
```
p3coa3 = ListLinePlot[
  {DataUpNDSc4coa[[503 ;; 753, {1, 3}]], DataUpNDSc6coa[[503 ;; 753, {1, 3}]],
   DataUpNDSc4c6coa[[503 ;; 753, {1, 3}]], DataUpNDScintermedcoa[[503 ;; 753, {1, 3}]],
   DataUpNDSfreecoa[[503 ;; 753, {1, 3}]]}, PlotRange → {{0, 250}, {0, 10 000}},
  PlotStyle → {Magenta, Blue, Cyan, Green, Darker[Yellow], Magenta, Blue,
    Cyan, Green, Darker[Yellow]}, AxesStyle → Directive[Black, 18],
  LabelStyle → Directive[Black, 18], Frame → {{True, False}, {True, False}},
  FrameLabel → {{"CoA Esters(μM) )", None}, {"Palmitoyl-CoA(μM)", None}},
  BaseStyle → {FontSize → 18, FontWeight → ""},
  FrameStyle → Thickness[0.00005], ImageSize → Scaled[0.25]]
```

Out[•]=
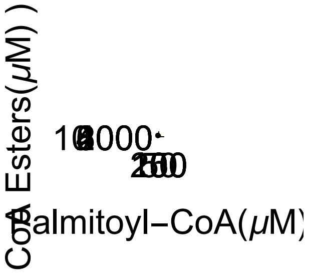

In[•]:=
```
p3coa4 = ListLinePlot[{DataUpNDSc4coa[[754 ;; 1004, {1, 3}]],
   DataUpNDSc6coa[[754 ;; 1004, {1, 3}]], DataUpNDSc4c6coa[[754 ;; 1004, {1, 3}]],
   DataUpNDScintermedcoa[[754 ;; 1004, {1, 3}]],
   DataUpNDSfreecoa[[754 ;; 1004, {1, 3}]]}, PlotRange → {{0, 250}, {0, 10 000}},
  PlotStyle → {Magenta, Blue, Cyan, Green, Darker[Yellow], Magenta, Blue,
    Cyan, Green, Darker[Yellow]}, AxesStyle → Directive[Black, 18],
  LabelStyle → Directive[Black, 18], Frame → {{True, False}, {True, False}},
  FrameLabel → {{"CoA Esters(μM) )", None}, {"Palmitoyl-CoA(μM)", None}},
  BaseStyle → {FontSize → 18, FontWeight → ""},
  FrameStyle → Thickness[0.00005], ImageSize → Scaled[0.25]]
```

Out[•]=
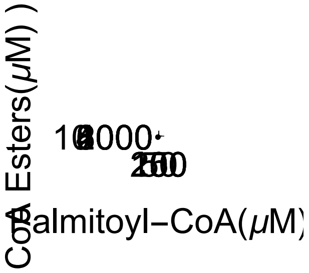

In[•]:=
```
p3coa5 = ListLinePlot[{DataUpNDSc4coa[[1005 ;; 1255, {1, 3}]],
   DataUpNDSc6coa[[1005 ;; 1255, {1, 3}]], DataUpNDSc4c6coa[[1005 ;; 1255, {1, 3}]],
   DataUpNDScintermedcoa[[1005 ;; 1255, {1, 3}]],
   DataUpNDSfreecoa[[1005 ;; 1255, {1, 3}]]}, PlotRange → All,
  PlotStyle → {Magenta, Blue, Cyan, Green, Darker[Yellow], Magenta, Blue,
    Cyan, Green, Darker[Yellow]}, AxesStyle → Directive[Black, 18],
  LabelStyle → Directive[Black, 18], Frame → {{True, False}, {True, False}},
  FrameLabel → {{"CoA Esters(μM) )", None}, {"Palmitoyl-CoA(μM)", None}},
  BaseStyle → {FontSize → 18, FontWeight → ""},
  FrameStyle → Thickness[0.00005], ImageSize → Scaled[0.25]]
```

Out[•]=

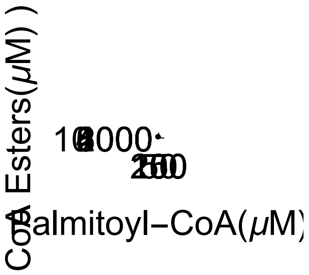

In[•]:=
```
p3coa5 = ListLinePlot[{DataUpNDSc4coa[[1005 ;; 1255, {1, 3}]],
   DataUpNDSc6coa[[1005 ;; 1255, {1, 3}]], DataUpNDSc4c6coa[[1005 ;; 1255, {1, 3}]],
   DataUpNDScintermedcoa[[1005 ;; 1255, {1, 3}]],
   DataUpNDSfreecoa[[1005 ;; 1255, {1, 3}]]}, PlotRange → All,
  PlotStyle → {Magenta, Blue, Cyan, Green, Darker[Yellow],
    Magenta, Blue, Cyan, Green, Darker[Yellow]},
  AxesStyle → Directive[Black, 18], LabelStyle → Directive[Black, 18],
  PlotLegends → Frame → {{True, False}, {True, False}},
  FrameLabel → {{"CoA Esters(μM) )", None}, {"Palmitoyl-CoA(μM)", None}},
  BaseStyle → {FontSize → 18, FontWeight → ""},
  FrameStyle → Thickness[0.00005], ImageSize → Scaled[0.25]]
```

Out[•]=

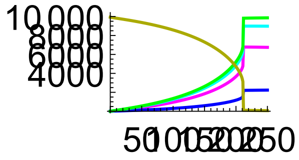

Frame → (True False / True False)

In[∘]:=
```
p3coa51 = ListLinePlot[
  {DataUpNDScintermedcoa[[1 ;; 251, {1, 3}]], DataUpNDSfreecoa[[1 ;; 251, {1, 3}]],
   DataUpNDScintermedcoa[[252 ;; 502, {1, 3}]], DataUpNDSfreecoa[[252 ;; 502, {1, 3}]],
   DataUpNDScintermedcoa[[503 ;; 753, {1, 3}]], DataUpNDSfreecoa[[503 ;; 753, {1, 3}]],
   DataUpNDScintermedcoa[[754 ;; 1004, {1, 3}]],
   DataUpNDSfreecoa[[754 ;; 1004, {1, 3}]], DataUpNDScintermedcoa[[
     1005 ;; 1255, {1, 3}]], DataUpNDSfreecoa[[1005 ;; 1255, {1, 3}]]},
  PlotRange → All, PlotStyle → {Magenta, Blue, Cyan, Green, Darker[Yellow],
    Magenta, Blue, Cyan, Green, Darker[Yellow]},
  AxesStyle → Directive[Black, 18], LabelStyle → Directive[Black, 18],
  PlotLegends → {"Intermediate CoA esters", "Free CoA"},
  Frame → {{True, False}, {True, False}},
  FrameLabel → {{"CoA Esters(μM) )", None}, {"Palmitoyl-CoA(μM)", None}},
  BaseStyle → {FontSize → 18, FontWeight → ""},
  FrameStyle → Thickness[0.00005], ImageSize → Scaled[0.25]]
```

Out[∘]=

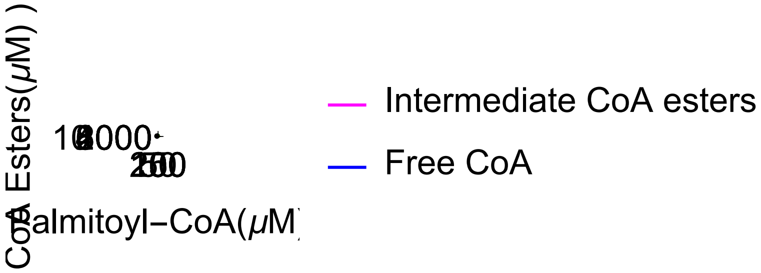

In[∘]:=
```
Grid[{{p3coa1, p3coa2, p3coa3}, {p3coa4, p3coa5}},
 Frame → {True, True, True, True}, Spacings → {2, 2}]
```

Out[∘]=

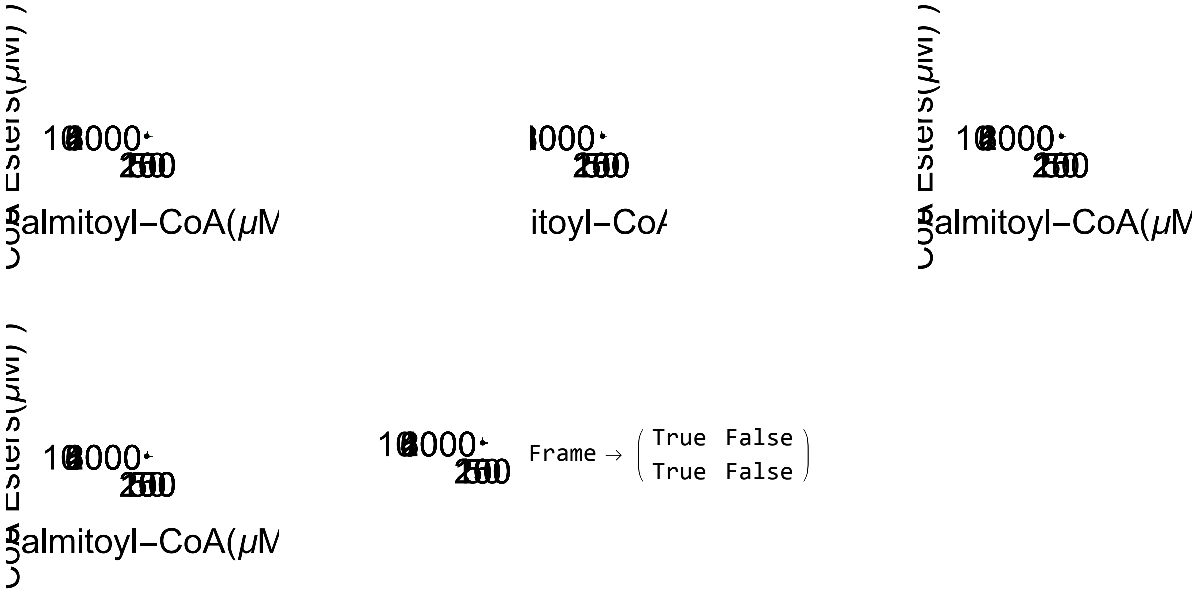

In[∘]:=

D. Steady state computation with varying palmitoyl-CoA (X) and FAD:-FADH Ratio (Z)

In[∘]:=
```
ParmScan[X_, Z_] := {
   sfcpt1C16 → 1, Vcpt1 → 0.012, Kmcpt1C16AcylCoACYT → 13.8,
   Kmcpt1CarCYT → 250, Kmcpt1C16AcylCarCYT → 136, Kmcpt1CoACYT → 40.7,
```

```
Kicpt1MalCoACYT → 9.1, Keqcpt1 → 0.45, ncpt1 → 2.4799,
Vfcact → 0.42, Vrcact → 0.42, KmcactC16AcylCarCYT → 15,
KmcactC14AcylCarCYT → 15, KmcactC12AcylCarCYT → 15, KmcactC10AcylCarCYT → 15,
KmcactC8AcylCarCYT → 15, KmcactC6AcylCarCYT → 15, KmcactC4AcylCarCYT → 15,
KmcactCarMAT → 130, KmcactC16AcylCarMAT → 15, KmcactC14AcylCarMAT → 15,
KmcactC12AcylCarMAT → 15, KmcactC10AcylCarMAT → 15, KmcactC8AcylCarMAT → 15,
KmcactC6AcylCarMAT → 15, KmcactC4AcylCarMAT → 15, KmcactCarCYT → 130,
KicactC16AcylCarCYT → 56, KicactC14AcylCarCYT → 56, KicactC12AcylCarCYT → 56,
KicactC10AcylCarCYT → 56, KicactC8AcylCarCYT → 56, KicactC6AcylCarCYT → 56,
KicactC4AcylCarCYT → 56, KicactCarCYT → 200, Keqcact → 1,
sfcpt2C16 → 0.85, sfcpt2C14 → 1, sfcpt2C12 → 0.95, sfcpt2C10 → 0.95,
sfcpt2C8 → 0.35, sfcpt2C6 → 0.15, sfcpt2C4 → 0.01, Vcpt2 → 0.391,
Kmcpt2C16AcylCarMAT → 51, Kmcpt2C14AcylCarMAT → 51, Kmcpt2C12AcylCarMAT → 51,
Kmcpt2C10AcylCarMAT → 51, Kmcpt2C8AcylCarMAT → 51, Kmcpt2C6AcylCarMAT → 51,
Kmcpt2C4AcylCarMAT → 51, Kmcpt2CoAMAT → 30, Kmcpt2C16AcylCoAMAT → 38,
Kmcpt2C14AcylCoAMAT → 38, Kmcpt2C12AcylCoAMAT → 38,
Kmcpt2C10AcylCoAMAT → 38, Kmcpt2C8AcylCoAMAT → 38, Kmcpt2C6AcylCoAMAT → 1000,
Kmcpt2C4AcylCoAMAT → 1000000, Kmcpt2CarMAT → 350, Keqcpt2 → 2.22,
sfvlcadC16 → 1, sfvlcadC14 → 0.42, sfvlcadC12 → 0.11, Vvlcad → 0.008,
KmvlcadC16AcylCoAMAT → 6.5, KmvlcadC14AcylCoAMAT → 4, KmvlcadC12AcylCoAMAT → 2.7,
KmvlcadFAD → 0.12, KmvlcadC16EnoylCoAMAT → 1.08, KmvlcadC14EnoylCoAMAT → 1.08,
KmvlcadC12EnoylCoAMAT → 1.08, KmvlcadFADH → 24.2, Keqvlcad → 6,
sflcadC16 → 0.9, sflcadC14 → 1, sflcadC12 → 0.9, sflcadC10 → 0.75, sflcadC8 → 0.4,
Vlcad → 0.01, KmlcadC16AcylCoAMAT → 2.5, KmlcadC14AcylCoAMAT → 7.4,
KmlcadC12AcylCoAMAT → 9, KmlcadC10AcylCoAMAT → 24.3, KmlcadC8AcylCoAMAT → 123,
KmlcadFAD → 0.12, KmlcadC16EnoylCoAMAT → 1.08, KmlcadC14EnoylCoAMAT → 1.08,
KmlcadC12EnoylCoAMAT → 1.08, KmlcadC10EnoylCoAMAT → 1.08,
KmlcadC8EnoylCoAMAT → 1.08, KmlcadFADH → 24.2, Keqlcad → 6,
sfmcadC12 → 0.38, sfmcadC10 → 0.8, sfmcadC8 → 0.87, sfmcadC6 → 1, sfmcadC4 → 0.12,
Vmcad → 0.081, KmmcadC12AcylCoAMAT → 5.7, KmmcadC10AcylCoAMAT → 5.4,
KmmcadC8AcylCoAMAT → 4, KmmcadC6AcylCoAMAT → 9.4, KmmcadC4AcylCoAMAT → 135,
KmmcadFAD → 0.12, KmmcadC12EnoylCoAMAT → 1.08, KmmcadC10EnoylCoAMAT → 1.08,
KmmcadC8EnoylCoAMAT → 1.08, KmmcadC6EnoylCoAMAT → 1.08,
KmmcadC4EnoylCoAMAT → 1.08, KmmcadFADH → 24.2, Keqmcad → 6,
sfscadC6 → 0.3, sfscadC4 → 1, Vscad → 0.081, KmscadC6AcylCoAMAT → 285,
KmscadC4AcylCoAMAT → 10.7, KmscadFAD → 0.12, KmscadC6EnoylCoAMAT → 1.08,
KmscadC4EnoylCoAMAT → 1.08, KmscadFADH → 24.2, Keqscad -> 6,
sfcrotC16 → 0.13, sfcrotC14 → 0.2, sfcrotC12 → 0.25, sfcrotC10 → 0.33, sfcrotC8 → 0.58,
sfcrotC6 → 0.83, sfcrotC4 → 1, Vcrot → 3.6, KmcrotC16EnoylCoAMAT → 150,
KmcrotC14EnoylCoAMAT → 100, KmcrotC12EnoylCoAMAT → 25, KmcrotC10EnoylCoAMAT → 25,
KmcrotC8EnoylCoAMAT → 25, KmcrotC6EnoylCoAMAT → 25, KmcrotC4EnoylCoAMAT → 40,
KmcrotC16HydroxyacylCoAMAT → 45, KmcrotC14HydroxyacylCoAMAT → 45,
KmcrotC12HydroxyacylCoAMAT → 45, KmcrotC10HydroxyacylCoAMAT → 45,
KmcrotC8HydroxyacylCoAMAT → 45, KmcrotC6HydroxyacylCoAMAT → 45,
KmcrotC4HydroxyacylCoAMAT → 45, KicrotC4AcetoacylCoA → 1.6, Keqcrot → 3.13,
sfmschadC16 → 0.6, sfmschadC14 → 0.5, sfmschadC12 → 0.43, sfmschadC10 → 0.64,
sfmschadC8 → 0.89, sfmschadC6 → 1, sfmschadC4 → 0.67, Vmschad → 1,
KmmschadC16HydroxyacylCoAMAT → 1.5, KmmschadC14HydroxyacylCoAMAT → 1.8,
KmmschadC12HydroxyacylCoAMAT → 3.7, KmmschadC10HydroxyacylCoAMAT → 8.8,
KmmschadC8HydroxyacylCoAMAT → 16.3, KmmschadC6HydroxyacylCoAMAT → 28.6,
KmmschadC4HydroxyacylCoAMAT → 69.9, KmmschadNADMAT → 58.5,
KmmschadC16KetoacylCoAMAT → 1.4, KmmschadC14KetoacylCoAMAT → 1.4,
KmmschadC12KetoacylCoAMAT → 1.6, KmmschadC10KetoacylCoAMAT → 2.3,
KmmschadC8KetoacylCoAMAT → 4.1, KmmschadC6KetoacylCoAMAT → 5.8,
KmmschadC4AcetoacylCoAMAT → 16.9, KmmschadNADHMAT → 5.4, Keqmschad → 2.17 * 10^-4,
```

```
  sfmckatC16 → 0, sfmckatC14 → 0.2, sfmckatC12 → 0.38, sfmckatC10 → 0.65,
  sfmckatC8 → 0.81, sfmckatC6 → 1, sfmckatC4 → 0.49, Vmckat → 0.377,
  KmmckatC16KetoacylCoAMAT → 1.1, KmmckatC14KetoacylCoAMAT → 1.2,
  KmmckatC12KetoacylCoAMAT → 1.3, KmmckatC10KetoacylCoAMAT → 2.1,
  KmmckatC8KetoacylCoAMAT → 3.2, KmmckatC6KetoacylCoAMAT → 6.7,
  KmmckatC4AcetoacylCoAMAT → 12.4, KmmckatCoAMAT → 26.6,
  KmmckatC14AcylCoAMAT → 13.83, KmmckatC16AcylCoAMAT → 13.83,
  KmmckatC12AcylCoAMAT → 13.83, KmmckatC10AcylCoAMAT → 13.83,
  KmmckatC8AcylCoAMAT → 13.83, KmmckatC6AcylCoAMAT → 13.83,
  KmmckatC4AcylCoAMAT → 13.83, KmmckatAcetylCoAMAT → 30, Keqmckat → 1051,
  sfmtpC16 → 1, sfmtpC14 → 0.9, sfmtpC12 → 0.81, sfmtpC10 → 0.73, sfmtpC8 → 0.34,
  Vmtp → 2.84, KmmtpC16EnoylCoAMAT → 25, KmmtpC14EnoylCoAMAT → 25,
  KmmtpC12EnoylCoAMAT → 25, KmmtpC10EnoylCoAMAT → 25, KmmtpC8EnoylCoAMAT → 25,
  KmmtpNADMAT → 60, KmmtpCoAMAT → 30, KmmtpC14AcylCoAMAT → 13.83,
  KmmtpC16AcylCoAMAT → 13.83, KmmtpC12AcylCoAMAT → 13.83,
  KmmtpC10AcylCoAMAT → 13.83, KmmtpC8AcylCoAMAT → 13.83, KmmtpC6AcylCoAMAT → 13.83,
  KmmtpNADHMAT → 50, KmmtpAcetylCoAMAT → 30, Keqmtp → 0.71,
  Ksacesink → 6 000 000, K1acesink → 70, Ksfadhsink → 6 000 000,
  K1fadhsink → 0.46, Ksnadhsink → 6 000 000, K1nadhsink → 12,
  C16AcylCoACYT → X, CarCYT → 200, CoACYT → 140, MalCoACYT → 0,
  CarMAT -> 950, FADtMAT → 0.77, NADtMAT → 250, CoAMATt → 5000,
  VCYT → 2.2 * 10^-6, VMAT → 1.8 * 10^-6, AcetylCoAMAT → 70, FADHMAT → Z, NADHMAT → 12};

tsolScan[X_, Z_] :=
  NDSolve[Join[Odes /. RateEqs /. CoAMATX /. ParmScan[X, Z], InitialConditions],
   Vars, {t, 0, 1 000 000 000}];

SsScan[X_, Z_] := Module[{SSGuess},
  SSGuess := Table[{Vars[[i]][t],
     (Vars[[i]][900 000 000] /. tsolScan[X, Z])[[1]]}, {i, 1, Length[Vars]}];
  FindRoot[Table[Odes[[i, 2]] == 0, {i, 1, Length[Odes]}] /. RateEqs /. CoAMATX /.
    ParmScan[X, Z], SSGuess]]
```

In[ ]:=
```
ScanDownNDSf[Ystart_, dY_, Yend_] := Monitor[Module[{SS, SSGuess},
   DataDownNDSfluxf = {};
   Xstart = 250;
   Xend = 0;
   YY = {0.40, 0.43, 0.46, 0.50, 0.52, 0.55, 0.60, 0.70};
   (*YY={0.93, 0.79, 0.67, 0.54, 0.48, 0.40, 0.28, 0.10};*)
   For[Y = Ystart, Y ≤ Yend,
    Z = YY[[Y]];
    tsolStart = tsolScan[Xend, Z];
    SSGuess = Table[{Vars[[i]][t],
       (Vars[[i]][900000000] /. tsolStart)[[1]]}, {i, 1, Length[Vars]}];
    SSGuess1 = SSGuess[[All, 1]];
    SSGuess2 = SSGuess[[All, 2]];
    SSGuess1int = SSGuess1 /. t → 0;
    InitialConditionsUD = Thread[SSGuess1int == SSGuess2];
    dX = 1;
    For[X = 250, X ≥ 0,


     tsolScanNDS = NDSolve[Join[Odes /. RateEqs /. CoAMATX /. ParmScan[X, Z],
        InitialConditionsUD], Vars, {t, 0, 1000000000}];
     SSGuess = Table[{Vars[[i]][t], (Vars[[i]][900000000] /. tsolScanNDS)[[1]]},
       {i, 1, Length[Vars]}];
     SSGuess1 = SSGuess[[All, 1]];
     SSGuess2 = SSGuess[[All, 2]];
     SSGuess1int = SSGuess1 /. t → 0;
     InitialConditionsUD = Thread[SSGuess1int == SSGuess2];
     SS = Thread[SSGuess1 → SSGuess2];

     AppendTo[DataDownNDSfluxf,
      {X, Z, 10^3 vcpt1C16 /. RateEqs /. CoAMATX /. ParmScan[X, Z] /. SS}];
     X = X - dX;];
    Y = Y + dY;]
   ], ProgressIndicator[X, {Xstart, Xend}]]
```

In[ ]:=
```
ScanDownNDSf[1, 1, 8]
```

In[•]:=
```
ScanUpNDSf[Ystart_, dY_, Yend_] := Monitor[Module[{SS, SSGuess},
   DataUpNDSfluxf = {};
   Xstart = 0;
   Xend = 250;
   YY = {0.40, 0.43, 0.46, 0.50, 0.52, 0.55, 0.60, 0.70};
   (*YY={0.93, 0.79, 0.67, 0.54, 0.48, 0.40, 0.28, 0.10};*)
   For[Y = Ystart, Y ≤ Yend,
    Z = YY[[Y]];
    tsolStart = tsolScan[Xstart, Z ];
    SSGuess = Table[{Vars[[i]][t],
       (Vars[[i]][900000000] /. tsolStart)[[1]]}, {i, 1, Length[Vars]}];
    SSGuess1 = SSGuess[[All, 1]];
    SSGuess2 = SSGuess[[All, 2]];
    SSGuess1int = SSGuess1 /. t → 0;
    InitialConditionsUD = Thread[SSGuess1int == SSGuess2];

    dX = 1;
    For[X = 0, X ≤ 250,


     tsolScanNDS = NDSolve[Join[Odes /. RateEqs /. CoAMATX /. ParmScan[X, Z],
        InitialConditionsUD], Vars, {t, 0, 1000000000}];
     SSGuess = Table[{Vars[[i]][t], (Vars[[i]][900000000] /. tsolScanNDS)[[1]]},
       {i, 1, Length[Vars]}];
     SSGuess1 = SSGuess[[All, 1]];
     SSGuess2 = SSGuess[[All, 2]];
     SSGuess1int = SSGuess1 /. t → 0;
     InitialConditionsUD = Thread[SSGuess1int == SSGuess2];
     SS = Thread[SSGuess1 → SSGuess2];

     AppendTo[DataUpNDSfluxf,
      {X, Z, 10^3 vcpt1C16 /. RateEqs /. CoAMATX /. ParmScan[X, Z] /. SS}];

     X = X + dX;];
    Y = Y + dY;]
  ], ProgressIndicator[X, {Xstart, Xend}]]
```

In[•]:=
```
ScanUpNDSf[1, 1, 8]
```

In[•]:=
```
(*Export[
 "C:\\Users\\Kindergeneeskunde\\Documents\\FentawNewlaptopPediatrics\\Code_mathematica
   \\mFAOvaryingFDAFADH2Rationew.xls",
 {"forwardFlux" -> DataUpNDSfluxf, "reverseFlux" -> DataDownNDSfluxf}]*)
```

In[∘]:=
```
p4 =
 ListLinePlot[{DataUpNDSfluxf[[1 ;; 251, {1, 3}]], DataUpNDSfluxf[[252 ;; 502, {1, 3}]],
   DataUpNDSfluxf[[503 ;; 753, {1, 3}]], DataUpNDSfluxf[[754 ;; 1004, {1, 3}]],
   DataUpNDSfluxf[[1005 ;; 1255, {1, 3}]], DataUpNDSfluxf[[1256 ;; 1506, {1, 3}]],
   DataUpNDSfluxf[[1507 ;; 1757, {1, 3}]], DataUpNDSfluxf[[1758 ;; 2008, {1, 3}]],
   DataDownNDSfluxf[[1 ;; 251, {1, 3}]], DataDownNDSfluxf[[252 ;; 502, {1, 3}]],
   DataDownNDSfluxf[[503 ;; 753, {1, 3}]], DataDownNDSfluxf[[754 ;; 1004, {1, 3}]],
   DataDownNDSfluxf[[1005 ;; 1255, {1, 3}]], DataDownNDSfluxf[[1256 ;; 1506, {1, 3}]],
   DataDownNDSfluxf[[1507 ;; 1757, {1, 3}]] , DataDownNDSfluxf[[1758 ;; 2008, {1, 3}]]},
  PlotRange → All, PlotStyle → {Magenta, Blue, Cyan, Green, Darker[Yellow], Orange, Red,
    Darker[Red], Magenta, Blue, Cyan, Green, Darker[Yellow], Orange, Red, Darker[Red]},
  AxesStyle → Directive[Black, 18], LabelStyle → Directive[Black, 18],
  PlotLegends → {"0.93", "0.79", "0.67", "0.54", "0.48", "0.40", "0.28", "0.10"},
  PlotLabel → "FAD:FADH2 Ratio",
  Frame → {{True, False}, {True, False}},
  FrameLabel → {{"Flux(μmol.min^-1.gProtein^-1)", None}, {"Palmitoyl-CoA(μM)", None}},
  BaseStyle → {FontSize → 18, FontWeight → ""},
  FrameStyle → Thickness[0.00005], ImageSize → Scaled[0.25], AspectRatio → 1]
```

Out[∘]=

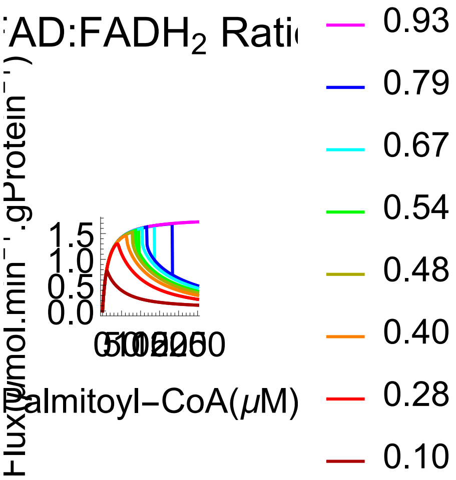

In[ ]:=
```
sub1 = ListLinePlot[{DataUpNDSfluxf[[1 ;; 251, {1, 3}]],
    DataUpNDSfluxf[[252 ;; 502, {1, 3}]], DataUpNDSfluxf[[503 ;; 753, {1, 3}]],
    DataUpNDSfluxf[[754 ;; 1004, {1, 3}]], DataUpNDSfluxf[[1005 ;; 1255, {1, 3}]],
    DataUpNDSfluxf[[1256 ;; 1506, {1, 3}]], DataUpNDSfluxf[[1507 ;; 1757, {1, 3}]],
    DataUpNDSfluxf[[1758 ;; 2008, {1, 3}]], DataDownNDSfluxf[[1 ;; 251, {1, 3}]],
    DataDownNDSfluxf[[252 ;; 502, {1, 3}]], DataDownNDSfluxf[[503 ;; 753, {1, 3}]],
    DataDownNDSfluxf[[754 ;; 1004, {1, 3}]], DataDownNDSfluxf[[1005 ;; 1255, {1, 3}]],
    DataDownNDSfluxf[[1256 ;; 1506, {1, 3}]], DataDownNDSfluxf[[1507 ;; 1757, {1, 3}]] ,
    DataDownNDSfluxf[[1758 ;; 2008, {1, 3}]]}, PlotRange → {{0, 85}, {0.5, 1.6}},
   PlotStyle → {Magenta, Blue, Cyan, Green, Darker[Yellow], Orange, Red, Darker[Red],
     Magenta, Blue, Cyan, Green, Darker[Yellow], Orange, Red, Darker[Red]},
   AxesStyle → Directive[Black, 18], LabelStyle → Directive[Black, 18],
   PlotLegends → {}, PlotLabel → " ",
   Frame → {{True, False}, {True, False}}, FrameLabel → {},
   BaseStyle → {FontSize → 18, FontWeight → ""},
   FrameStyle → Thickness[0.00005], ImageSize → Scaled[0.15], AspectRatio → 1]
FDAFADH2RatioVaryingMagnified = ListLinePlot[
   {DataUpNDSfluxf[[1 ;; 251, {1, 3}]], DataUpNDSfluxf[[252 ;; 502, {1, 3}]],
    DataUpNDSfluxf[[503 ;; 753, {1, 3}]], DataUpNDSfluxf[[754 ;; 1004, {1, 3}]],
    DataUpNDSfluxf[[1005 ;; 1255, {1, 3}]], DataUpNDSfluxf[[1256 ;; 1506, {1, 3}]],
    DataUpNDSfluxf[[1507 ;; 1757, {1, 3}]], DataUpNDSfluxf[[1758 ;; 2008, {1, 3}]],
    DataDownNDSfluxf[[1 ;; 251, {1, 3}]], DataDownNDSfluxf[[252 ;; 502, {1, 3}]],
    DataDownNDSfluxf[[503 ;; 753, {1, 3}]], DataDownNDSfluxf[[754 ;; 1004, {1, 3}]],
    DataDownNDSfluxf[[1005 ;; 1255, {1, 3}]], DataDownNDSfluxf[[1256 ;; 1506, {1, 3}]],
    DataDownNDSfluxf[[1507 ;; 1757, {1, 3}]] , DataDownNDSfluxf[[1758 ;; 2008, {1, 3}]]},
   PlotRange → All, PlotStyle → {Magenta, Blue, Cyan, Green, Darker[Yellow], Orange, Red,
     Darker[Red], Magenta, Blue, Cyan, Green, Darker[Yellow], Orange, Red, Darker[Red]},
   AxesStyle → Directive[Black, 18], LabelStyle → Directive[Black, 18],
   PlotLegends → {"0.40", "0.43", "0.46", "0.50", "0.52", "0.55", "0.60", "0.70"},
   PlotLabel → "FADH2",
   Frame → {{True, False}, {True, False}},
   FrameLabel → {{"Flux(μmol.min^-1.gProtein^-1)", None}, {"Palmitoyl-CoA(μM)", None}},
   BaseStyle → {FontSize → 18, FontWeight → ""},
   FrameStyle → Thickness[0.00005], ImageSize → Scaled[0.25],
   AspectRatio → 1, Epilog → Inset[sub1], PlotRangeClipping → False]
```

Out[ ]=

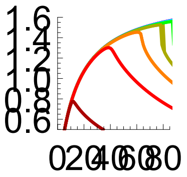

Out[◦]=

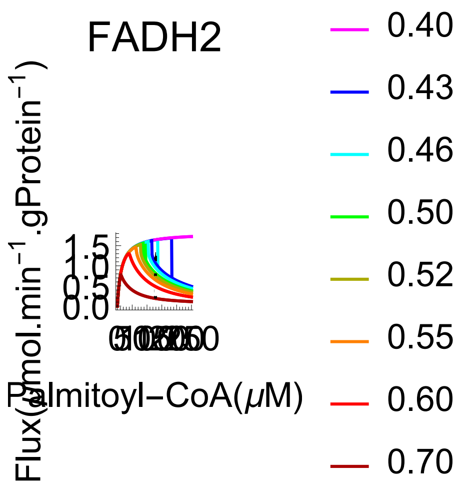

Combine the four plots

In[◦]:=
```
Grid[{{p1, p4}, {p2, p3}}, Frame → {True, True, True, True}, Spacings → {2, 2}]
```

Out[◦]=

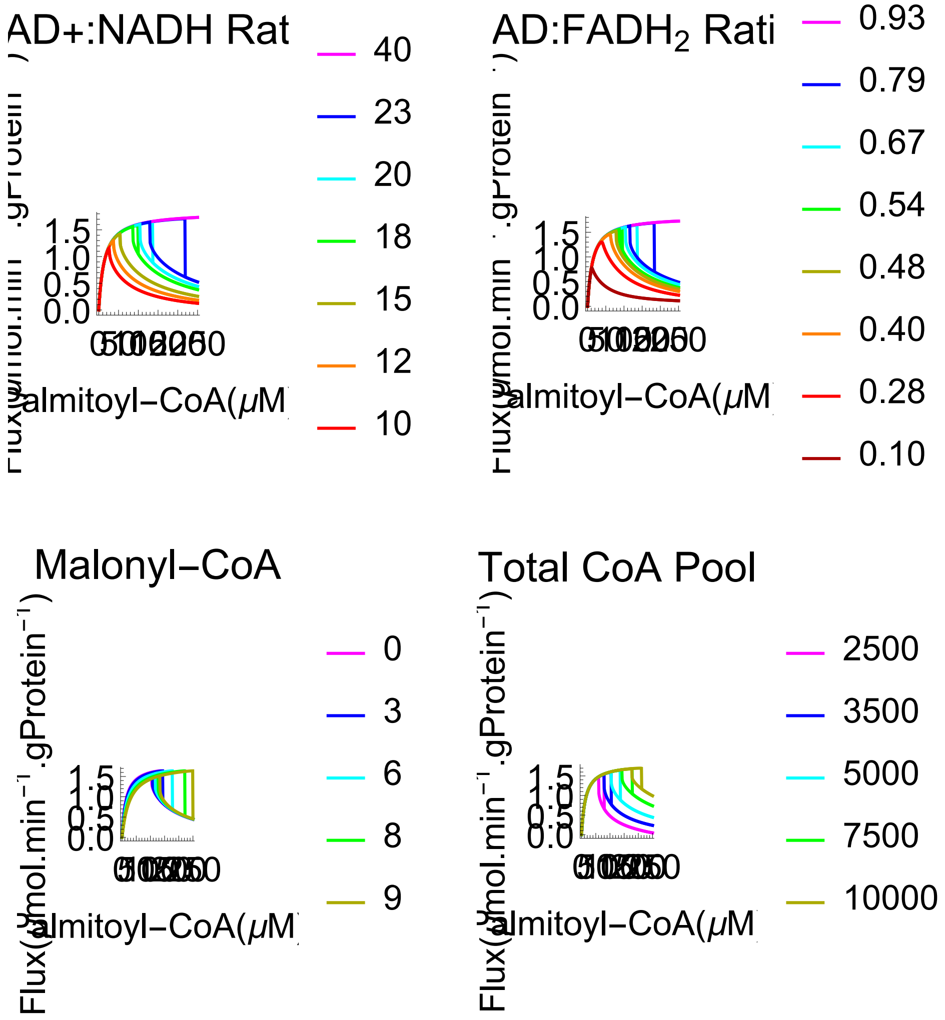

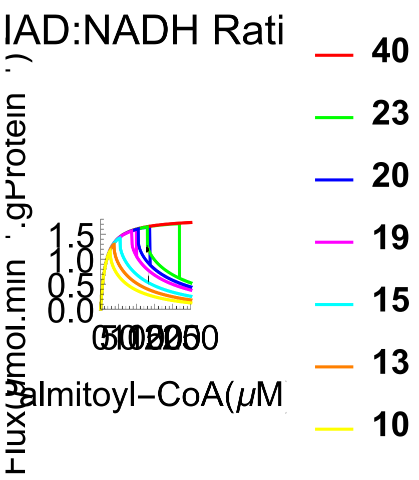
IAD:NADH Rati
40
23
20
19
15
13
10

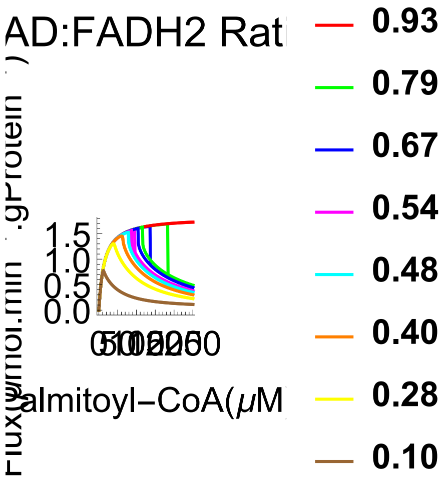
AD:FADH2 Rati
0.93
0.79
0.67
0.54
0.48
0.40
0.28
0.10

In[ ]:=

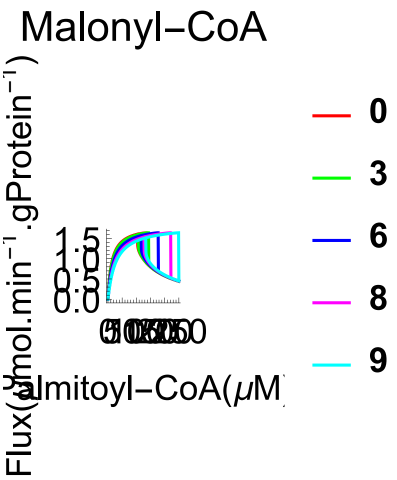
Malonyl–CoA
0
3
6
8
9

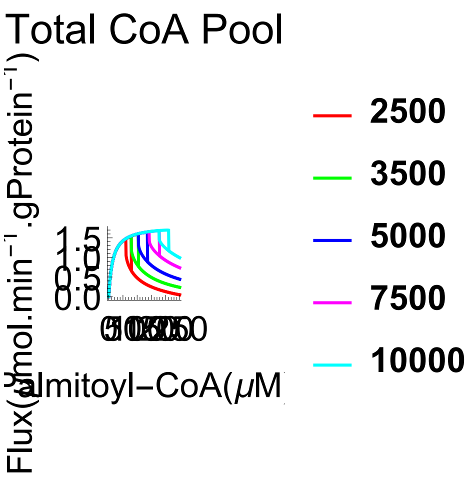
Total CoA Pool
2500
3500
5000
7500
10000

Out[ ]=

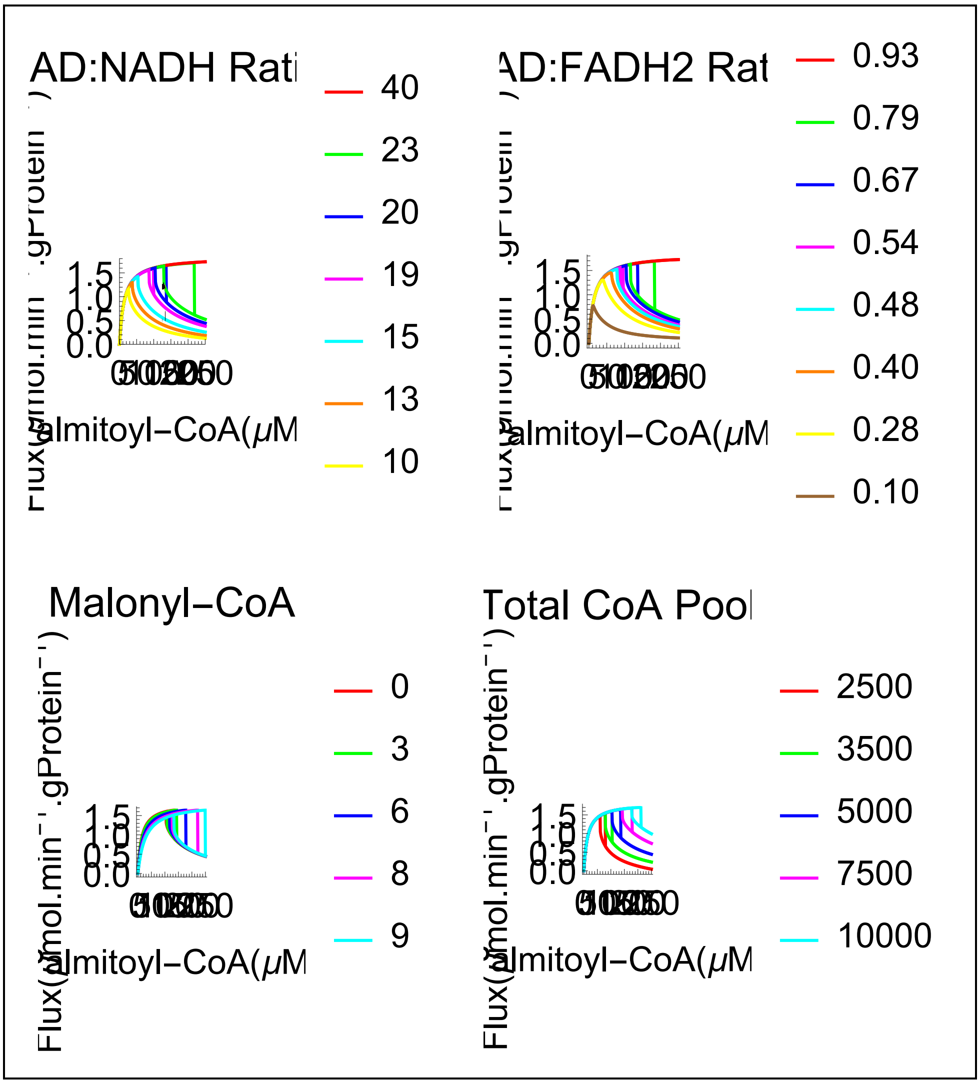

In[ ]:=

```
(*DumpSave[
 "C:\\Users\\Kindergeneeskunde\\Documents\\FentawNewlaptopPediatrics\\Code_mathematica
   \\BistabilitymFAO\\BistabilitymFAO.mx","Global`"]*)
(*Get["E:\\umcg_laptop\\Kindergeneeskunde\\Documents\\FentawNewlaptopPediatrics\\
   Code_mathematica\\BistabilitymFAO\\BistabilitymFAO.mx"]*)
```

E. Steady state computation with varying palmitoyl-CoA (X) and total NAD and NADH (Z)

In[ ]:=

```
ParmScan[X_, Z_] := {
   sfcpt1C16 → 1, Vcpt1 → 0.012, Kmcpt1C16AcylCoACYT → 13.8,
   Kmcpt1CarCYT → 250, Kmcpt1C16AcylCarCYT → 136, Kmcpt1CoACYT → 40.7,
   Kicpt1MalCoACYT → 9.1, Keqcpt1 → 0.45, ncpt1 → 2.4799,
   Vfcact → 0.42, Vrcact → 0.42, KmcactC16AcylCarCYT → 15,
   KmcactC14AcylCarCYT → 15, KmcactC12AcylCarCYT → 15, KmcactC10AcylCarCYT → 15,
   KmcactC8AcylCarCYT → 15, KmcactC6AcylCarCYT → 15, KmcactC4AcylCarCYT → 15,
```

```
KmcactCarMAT → 130, KmcactC16AcylCarMAT → 15, KmcactC14AcylCarMAT → 15,
KmcactC12AcylCarMAT → 15, KmcactC10AcylCarMAT → 15, KmcactC8AcylCarMAT → 15,
KmcactC6AcylCarMAT → 15, KmcactC4AcylCarMAT → 15, KmcactCarCYT → 130,
KicactC16AcylCarCYT → 56, KicactC14AcylCarCYT → 56, KicactC12AcylCarCYT → 56,
KicactC10AcylCarCYT → 56, KicactC8AcylCarCYT → 56, KicactC6AcylCarCYT → 56,
KicactC4AcylCarCYT → 56, KicactCarCYT → 200, Keqcact → 1,
sfcpt2C16 → 0.85, sfcpt2C14 → 1, sfcpt2C12 → 0.95, sfcpt2C10 → 0.95,
sfcpt2C8 → 0.35, sfcpt2C6 → 0.15, sfcpt2C4 → 0.01, Vcpt2 → 0.391,
Kmcpt2C16AcylCarMAT → 51, Kmcpt2C14AcylCarMAT → 51, Kmcpt2C12AcylCarMAT → 51,
Kmcpt2C10AcylCarMAT → 51, Kmcpt2C8AcylCarMAT → 51, Kmcpt2C6AcylCarMAT → 51,
Kmcpt2C4AcylCarMAT → 51, Kmcpt2CoAMAT → 30, Kmcpt2C16AcylCoAMAT → 38,
Kmcpt2C14AcylCoAMAT → 38, Kmcpt2C12AcylCoAMAT → 38,
Kmcpt2C10AcylCoAMAT → 38, Kmcpt2C8AcylCoAMAT → 38, Kmcpt2C6AcylCoAMAT → 1000,
Kmcpt2C4AcylCoAMAT → 1000000, Kmcpt2CarMAT → 350, Keqcpt2 → 2.22,
sfvlcadC16 → 1, sfvlcadC14 → 0.42, sfvlcadC12 → 0.11, Vvlcad → 0.008,
KmvlcadC16AcylCoAMAT → 6.5, KmvlcadC14AcylCoAMAT → 4, KmvlcadC12AcylCoAMAT → 2.7,
KmvlcadFAD → 0.12, KmvlcadC16EnoylCoAMAT → 1.08, KmvlcadC14EnoylCoAMAT → 1.08,
KmvlcadC12EnoylCoAMAT → 1.08, KmvlcadFADH → 24.2, Keqvlcad → 6,
sflcadC16 → 0.9, sflcadC14 → 1, sflcadC12 → 0.9, sflcadC10 → 0.75, sflcadC8 → 0.4,
Vlcad → 0.01, KmlcadC16AcylCoAMAT → 2.5, KmlcadC14AcylCoAMAT → 7.4,
KmlcadC12AcylCoAMAT → 9, KmlcadC10AcylCoAMAT → 24.3, KmlcadC8AcylCoAMAT → 123,
KmlcadFAD → 0.12, KmlcadC16EnoylCoAMAT → 1.08, KmlcadC14EnoylCoAMAT → 1.08,
KmlcadC12EnoylCoAMAT → 1.08, KmlcadC10EnoylCoAMAT → 1.08,
KmlcadC8EnoylCoAMAT → 1.08, KmlcadFADH → 24.2, Keqlcad → 6,
sfmcadC12 → 0.38, sfmcadC10 → 0.8, sfmcadC8 → 0.87, sfmcadC6 → 1, sfmcadC4 → 0.12,
Vmcad → 0.081, KmmcadC12AcylCoAMAT → 5.7, KmmcadC10AcylCoAMAT → 5.4,
KmmcadC8AcylCoAMAT → 4, KmmcadC6AcylCoAMAT → 9.4, KmmcadC4AcylCoAMAT → 135,
KmmcadFAD → 0.12, KmmcadC12EnoylCoAMAT → 1.08, KmmcadC10EnoylCoAMAT → 1.08,
KmmcadC8EnoylCoAMAT → 1.08, KmmcadC6EnoylCoAMAT → 1.08,
KmmcadC4EnoylCoAMAT → 1.08, KmmcadFADH → 24.2, Keqmcad → 6,
sfscadC6 → 0.3, sfscadC4 → 1, Vscad → 0.081, KmscadC6AcylCoAMAT → 285,
KmscadC4AcylCoAMAT → 10.7, KmscadFAD → 0.12, KmscadC6EnoylCoAMAT → 1.08,
KmscadC4EnoylCoAMAT → 1.08, KmscadFADH → 24.2, Keqscad -> 6,
sfcrotC16 → 0.13, sfcrotC14 → 0.2, sfcrotC12 → 0.25, sfcrotC10 → 0.33, sfcrotC8 → 0.58,
sfcrotC6 → 0.83, sfcrotC4 → 1, Vcrot → 3.6, KmcrotC16EnoylCoAMAT → 150,
KmcrotC14EnoylCoAMAT → 100, KmcrotC12EnoylCoAMAT → 25, KmcrotC10EnoylCoAMAT → 25,
KmcrotC8EnoylCoAMAT → 25, KmcrotC6EnoylCoAMAT → 25, KmcrotC4EnoylCoAMAT → 40,
KmcrotC16HydroxyacylCoAMAT → 45, KmcrotC14HydroxyacylCoAMAT → 45,
KmcrotC12HydroxyacylCoAMAT → 45, KmcrotC10HydroxyacylCoAMAT → 45,
KmcrotC8HydroxyacylCoAMAT → 45, KmcrotC6HydroxyacylCoAMAT → 45,
KmcrotC4HydroxyacylCoAMAT → 45, KicrotC4AcetoacylCoA → 1.6, Keqcrot → 3.13,
sfmschadC16 → 0.6, sfmschadC14 → 0.5, sfmschadC12 → 0.43, sfmschadC10 → 0.64,
sfmschadC8 → 0.89, sfmschadC6 → 1, sfmschadC4 → 0.67, Vmschad → 1,
KmmschadC16HydroxyacylCoAMAT → 1.5, KmmschadC14HydroxyacylCoAMAT → 1.8,
KmmschadC12HydroxyacylCoAMAT → 3.7, KmmschadC10HydroxyacylCoAMAT → 8.8,
KmmschadC8HydroxyacylCoAMAT → 16.3, KmmschadC6HydroxyacylCoAMAT → 28.6,
KmmschadC4HydroxyacylCoAMAT → 69.9, KmmschadNADMAT → 58.5,
KmmschadC16KetoacylCoAMAT → 1.4, KmmschadC14KetoacylCoAMAT → 1.4,
KmmschadC12KetoacylCoAMAT → 1.6, KmmschadC10KetoacylCoAMAT → 2.3,
KmmschadC8KetoacylCoAMAT → 4.1, KmmschadC6KetoacylCoAMAT → 5.8,
KmmschadC4AcetoacylCoAMAT → 16.9, KmmschadNADHMAT → 5.4, Keqmschad → 2.17 * 10^-4,
sfmckatC16 → 0, sfmckatC14 → 0.2, sfmckatC12 → 0.38, sfmckatC10 → 0.65,
sfmckatC8 → 0.81, sfmckatC6 → 1, sfmckatC4 → 0.49, Vmckat → 0.377,
KmmckatC16KetoacylCoAMAT → 1.1, KmmckatC14KetoacylCoAMAT → 1.2,
KmmckatC12KetoacylCoAMAT → 1.3, KmmckatC10KetoacylCoAMAT → 2.1,
```

```
    KmmckatC8KetoacylCoAMAT → 3.2, KmmckatC6KetoacylCoAMAT → 6.7,
    KmmckatC4AcetoacylCoAMAT → 12.4, KmmckatCoAMAT → 26.6,
    KmmckatC14AcylCoAMAT → 13.83, KmmckatC16AcylCoAMAT → 13.83,
    KmmckatC12AcylCoAMAT → 13.83, KmmckatC10AcylCoAMAT → 13.83,
    KmmckatC8AcylCoAMAT → 13.83, KmmckatC6AcylCoAMAT → 13.83,
    KmmckatC4AcylCoAMAT → 13.83, KmmckatAcetylCoAMAT → 30, Keqmckat → 1051,
    sfmtpC16 → 1, sfmtpC14 → 0.9, sfmtpC12 → 0.81, sfmtpC10 → 0.73, sfmtpC8 → 0.34,
    Vmtp → 2.84, KmmtpC16EnoylCoAMAT → 25, KmmtpC14EnoylCoAMAT → 25,
    KmmtpC12EnoylCoAMAT → 25, KmmtpC10EnoylCoAMAT → 25, KmmtpC8EnoylCoAMAT → 25,
    KmmtpNADMAT → 60, KmmtpCoAMAT → 30, KmmtpC14AcylCoAMAT → 13.83,
    KmmtpC16AcylCoAMAT → 13.83, KmmtpC12AcylCoAMAT → 13.83,
    KmmtpC10AcylCoAMAT → 13.83, KmmtpC8AcylCoAMAT → 13.83, KmmtpC6AcylCoAMAT → 13.83,
    KmmtpNADHMAT → 50, KmmtpAcetylCoAMAT → 30, Keqmtp → 0.71,
    Ksacesink → 6000000, K1acesink → 70, Ksfadhsink → 6000000,
    K1fadhsink → 0.46, Ksnadhsink → 6000000, K1nadhsink → 12,
    C16AcylCoACYT → X, CarCYT → 200, CoACYT → 140, MalCoACYT → 0,
    CarMAT -> 950, FADtMAT → 0.77, NADtMAT → Z, CoAMATt → 5000,
    VCYT → 2.2 * 10^-6, VMAT → 1.8 * 10^-6, AcetylCoAMAT → 70, FADHMAT → 0.46, NADHMAT → 12};

tsolScan[X_, Z_] :=
  NDSolve[Join[Odes /. RateEqs /. CoAMATX /. ParmScan[X, Z], InitialConditions],
   Vars, {t, 0, 1000000000}];

SsScan[X_, Z_] := Module[{SSGuess},
  SSGuess := Table[{Vars[[i]][t],
      (Vars[[i]][900000000] /. tsolScan[X, Z])[[1]]}, {i, 1, Length[Vars]}];
  FindRoot[Table[Odes[[i, 2]] == 0, {i, 1, Length[Odes]}] /. RateEqs /. CoAMATX /.
    ParmScan[X, Z], SSGuess]]
ScanDownNDSf[Ystart_, dY_, Yend_] := Monitor[Module[{SS, SSGuess},
   DataDownNDSfluxf = {};
   Xstart = 250;
   Xend = 0;
   YY = {100, 150, 175, 200, 225, 250, 275, 300};
   (*YY={0.93, 0.79, 0.67, 0.54, 0.48, 0.40, 0.28, 0.10};*)
   For[Y = Ystart, Y ≤ Yend,
    Z = YY[[Y]];
    tsolStart = tsolScan[Xend, Z];
    SSGuess = Table[{Vars[[i]][t],
       (Vars[[i]][900000000] /. tsolStart)[[1]]}, {i, 1, Length[Vars]}];
    SSGuess1 = SSGuess[[All, 1]];
    SSGuess2 = SSGuess[[All, 2]];
    SSGuess1int = SSGuess1 /. t → 0;
    InitialConditionsUD = Thread[SSGuess1int == SSGuess2];
    dX = 1;
    For[X = 250, X ≥ 0,


     tsolScanNDS = NDSolve[Join[Odes /. RateEqs /. CoAMATX /. ParmScan[X, Z],
        InitialConditionsUD], Vars, {t, 0, 1000000000}];
     SSGuess = Table[{Vars[[i]][t], (Vars[[i]][900000000] /. tsolScanNDS)[[1]]},
       {i, 1, Length[Vars]}];
     SSGuess1 = SSGuess[[All, 1]];
     SSGuess2 = SSGuess[[All, 2]];
     SSGuess1int = SSGuess1 /. t → 0;
```

```
      InitialConditionsUD = Thread[SSGuess1int == SSGuess2];
      SS = Thread[SSGuess1 → SSGuess2];

      AppendTo[DataDownNDSfluxf,
       {X, Z, 10^3 vcpt1C16 /. RateEqs /. CoAMATX /. ParmScan[X, Z] /. SS}];
      X = X - dX;];
     Y = Y + dY;]
   ], ProgressIndicator[X, {Xstart, Xend}]]
```

```
ScanDownNDSf[1, 1, 8]
```

```
ScanUpNDSf[Ystart_, dY_, Yend_] := Monitor[Module[{SS, SSGuess},
    DataUpNDSfluxf = {};
    Xstart = 0;
    Xend = 250;
    YY = {100, 150, 175, 200, 225, 250, 275, 300};
    (*YY={0.93, 0.79, 0.67, 0.54, 0.48, 0.40, 0.28, 0.10};*)
    For[Y = Ystart, Y ≤ Yend,
     Z = YY[[Y]];
     tsolStart = tsolScan[Xstart, Z ];
     SSGuess = Table[{Vars[[i]][t],
        (Vars[[i]][900000000] /. tsolStart)[[1]]}, {i, 1, Length[Vars]}];
     SSGuess1 = SSGuess[[All, 1]];
     SSGuess2 = SSGuess[[All, 2]];
     SSGuess1int = SSGuess1 /. t → 0;
     InitialConditionsUD = Thread[SSGuess1int == SSGuess2];

     dX = 1;
     For[X = 0, X ≤ 250,


      tsolScanNDS = NDSolve[Join[Odes /. RateEqs /. CoAMATX /. ParmScan[X, Z],
         InitialConditionsUD], Vars, {t, 0, 1000000000}];
      SSGuess = Table[{Vars[[i]][t], (Vars[[i]][900000000] /. tsolScanNDS)[[1]]},
        {i, 1, Length[Vars]}];
      SSGuess1 = SSGuess[[All, 1]];
      SSGuess2 = SSGuess[[All, 2]];
      SSGuess1int = SSGuess1 /. t → 0;
      InitialConditionsUD = Thread[SSGuess1int == SSGuess2];
      SS = Thread[SSGuess1 → SSGuess2];

      AppendTo[DataUpNDSfluxf,
       {X, Z, 10^3 vcpt1C16 /. RateEqs /. CoAMATX /. ParmScan[X, Z] /. SS}];

      X = X + dX;];
     Y = Y + dY;]
   ], ProgressIndicator[X, {Xstart, Xend}]]
```

```
ScanUpNDSf[1, 1, 8]
```

```
(*Export[
 "C:\\Users\\Kindergeneeskunde\\Documents\\FentawNewlaptopPediatrics\\Code_mathematica
   \\mFAOvaryingFDAFADH2Rationew.xls",
 {"forwardFlux" -> DataUpNDSfluxf, "reverseFlux" -> DataDownNDSfluxf}]*)

pn =
 ListLinePlot[{DataUpNDSfluxf[[1 ;; 251, {1, 3}]], DataUpNDSfluxf[[252 ;; 502, {1, 3}]],
   DataUpNDSfluxf[[503 ;; 753, {1, 3}]], DataUpNDSfluxf[[754 ;; 1004, {1, 3}]],
   DataUpNDSfluxf[[1005 ;; 1255, {1, 3}]], DataUpNDSfluxf[[1256 ;; 1506, {1, 3}]],
   DataUpNDSfluxf[[1507 ;; 1757, {1, 3}]], DataUpNDSfluxf[[1758 ;; 2008, {1, 3}]],
   DataDownNDSfluxf[[1 ;; 251, {1, 3}]], DataDownNDSfluxf[[252 ;; 502, {1, 3}]],
   DataDownNDSfluxf[[503 ;; 753, {1, 3}]], DataDownNDSfluxf[[754 ;; 1004, {1, 3}]],
   DataDownNDSfluxf[[1005 ;; 1255, {1, 3}]], DataDownNDSfluxf[[1256 ;; 1506, {1, 3}]],
   DataDownNDSfluxf[[1507 ;; 1757, {1, 3}]] , DataDownNDSfluxf[[1758 ;; 2008, {1, 3}]]},
  PlotRange → All, PlotStyle → {Magenta, Blue, Cyan, Green, Darker[Yellow], Orange, Red,
    Darker[Red], Magenta, Blue, Cyan, Green, Darker[Yellow], Orange, Red, Darker[Red]},
  AxesStyle → Directive[Black, 18], LabelStyle → Directive[Black, 18],
  PlotLegends → {"100", "150", "175", "200", "225", "250", "275", "300"},
  PlotLabel → "Total NAD and NADH",
  Frame → {{True, False}, {True, False}},
  FrameLabel → {{"Flux(μmol.min^-1.gProtein^-1)", None}, {"Palmitoyl-CoA(μM)", None}},
  BaseStyle → {FontSize → 18, FontWeight → ""},
  FrameStyle → Thickness[0.00005], ImageSize → Scaled[0.25], AspectRatio → 1]
```

Out[ ]=

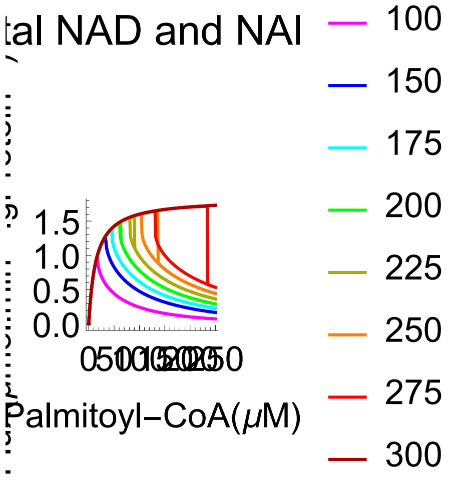

```
pn =
 ListLinePlot[{DataUpNDSfluxf[[1 ;; 251, {1, 3}]], DataUpNDSfluxf[[252 ;; 502, {1, 3}]],
   DataUpNDSfluxf[[503 ;; 753, {1, 3}]], DataUpNDSfluxf[[754 ;; 1004, {1, 3}]],
   DataUpNDSfluxf[[1005 ;; 1255, {1, 3}]], DataUpNDSfluxf[[1256 ;; 1506, {1, 3}]],
   DataUpNDSfluxf[[1507 ;; 1757, {1, 3}]], DataUpNDSfluxf[[1758 ;; 2008, {1, 3}]],
   DataDownNDSfluxf[[1 ;; 251, {1, 3}]], DataDownNDSfluxf[[252 ;; 502, {1, 3}]],
   DataDownNDSfluxf[[503 ;; 753, {1, 3}]], DataDownNDSfluxf[[754 ;; 1004, {1, 3}]],
   DataDownNDSfluxf[[1005 ;; 1255, {1, 3}]], DataDownNDSfluxf[[1256 ;; 1506, {1, 3}]],
   DataDownNDSfluxf[[1507 ;; 1757, {1, 3}]] , DataDownNDSfluxf[[1758 ;; 2008, {1, 3}]]},
  PlotRange → All, PlotStyle → {Magenta, Blue, Cyan, Green, Darker[Yellow], Orange, Red,
    Darker[Red], Magenta, Blue, Cyan, Green, Darker[Yellow], Orange, Red, Darker[Red]},
  AxesStyle → Directive[Black, 18], LabelStyle → Directive[Black, 18],
  PlotLegends → {"100", "150", "175", "200", "225", "250", "275", "300"},
  PlotLabel → "Total NAD and NADH",
  Frame → {{True, False}, {True, False}},
  FrameLabel → {{"Flux(μmol.min^-1.gProtein^-1)", None}, {"Palmitoyl-CoA(μM)", None}},
  BaseStyle → {FontSize → 18, FontWeight → ""},
  FrameStyle → Thickness[0.00005], ImageSize → Scaled[0.25], AspectRatio → 1]
```

Out[•]=

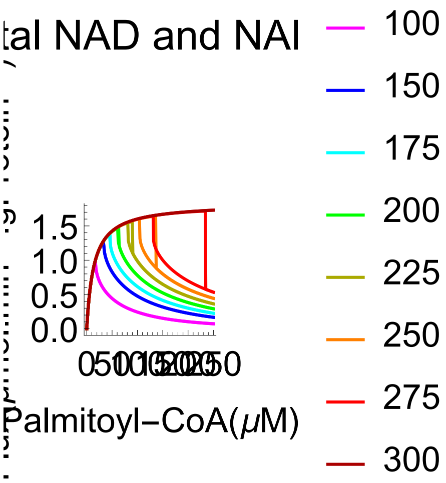

In[•]:=

```
DumpSave["E:\\umcg_laptop\\Kindergeneeskunde\\Documents\\FentawNewlaptopPediatrics\\
   Code_mathematica\\BistabilitymFAO\\BistabilitymFAO.mx", "Global`"]
```

Out[•]= {Global`}

In[•]:=

```
(*Get["E:\\umcg_laptop\\Kindergeneeskunde\\Documents\\FentawNewlaptopPediatrics\\
   Code_mathematica\\BistabilitymFAO\\BistabilitymFAO.mx"]*)
```

F. Steady state computation with varying palmitoyl-CoA (X) and CPT1 Vmax (Z)

In[•]:=

```
ParmScan[X_, Z_] := {
   sfcpt1C16 → 1, Vcpt1 → Z, Kmcpt1C16AcylCoACYT → 13.8,
   Kmcpt1CarCYT → 250, Kmcpt1C16AcylCarCYT → 136, Kmcpt1CoACYT → 40.7,
   Kicpt1MalCoACYT → 9.1, Keqcpt1 → 0.45, ncpt1 → 2.4799,
   Vfcact → 0.42, Vrcact → 0.42, KmcactC16AcylCarCYT → 15,
   KmcactC14AcylCarCYT → 15, KmcactC12AcylCarCYT → 15, KmcactC10AcylCarCYT → 15,
```

KmcactC8AcylCarCYT → 15, KmcactC6AcylCarCYT → 15, KmcactC4AcylCarCYT → 15,
KmcactCarMAT → 130, KmcactC16AcylCarMAT → 15, KmcactC14AcylCarMAT → 15,
KmcactC12AcylCarMAT → 15, KmcactC10AcylCarMAT → 15, KmcactC8AcylCarMAT → 15,
KmcactC6AcylCarMAT → 15, KmcactC4AcylCarMAT → 15, KmcactCarCYT → 130,
KicactC16AcylCarCYT → 56, KicactC14AcylCarCYT → 56, KicactC12AcylCarCYT → 56,
KicactC10AcylCarCYT → 56, KicactC8AcylCarCYT → 56, KicactC6AcylCarCYT → 56,
KicactC4AcylCarCYT → 56, KicactCarCYT → 200, Keqcact → 1,
sfcpt2C16 → 0.85, sfcpt2C14 → 1, sfcpt2C12 → 0.95, sfcpt2C10 → 0.95,
sfcpt2C8 → 0.35, sfcpt2C6 → 0.15, sfcpt2C4 → 0.01, Vcpt2 → 0.391,
Kmcpt2C16AcylCarMAT → 51, Kmcpt2C14AcylCarMAT → 51, Kmcpt2C12AcylCarMAT → 51,
Kmcpt2C10AcylCarMAT → 51, Kmcpt2C8AcylCarMAT → 51, Kmcpt2C6AcylCarMAT → 51,
Kmcpt2C4AcylCarMAT → 51, Kmcpt2CoAMAT → 30, Kmcpt2C16AcylCoAMAT → 38,
Kmcpt2C14AcylCoAMAT → 38, Kmcpt2C12AcylCoAMAT → 38,
Kmcpt2C10AcylCoAMAT → 38, Kmcpt2C8AcylCoAMAT → 38, Kmcpt2C6AcylCoAMAT → 1000,
Kmcpt2C4AcylCoAMAT → 1000000, Kmcpt2CarMAT → 350, Keqcpt2 → 2.22,
sfvlcadC16 → 1, sfvlcadC14 → 0.42, sfvlcadC12 → 0.11, Vvlcad → 0.008,
KmvlcadC16AcylCoAMAT → 6.5, KmvlcadC14AcylCoAMAT → 4, KmvlcadC12AcylCoAMAT → 2.7,
KmvlcadFAD → 0.12, KmvlcadC16EnoylCoAMAT → 1.08, KmvlcadC14EnoylCoAMAT → 1.08,
KmvlcadC12EnoylCoAMAT → 1.08, KmvlcadFADH → 24.2, Keqvlcad → 6,
sflcadC16 → 0.9, sflcadC14 → 1, sflcadC12 → 0.9, sflcadC10 → 0.75, sflcadC8 → 0.4,
Vlcad → 0.01, KmlcadC16AcylCoAMAT → 2.5, KmlcadC14AcylCoAMAT → 7.4,
KmlcadC12AcylCoAMAT → 9, KmlcadC10AcylCoAMAT → 24.3, KmlcadC8AcylCoAMAT → 123,
KmlcadFAD → 0.12, KmlcadC16EnoylCoAMAT → 1.08, KmlcadC14EnoylCoAMAT → 1.08,
KmlcadC12EnoylCoAMAT → 1.08, KmlcadC10EnoylCoAMAT → 1.08,
KmlcadC8EnoylCoAMAT → 1.08, KmlcadFADH → 24.2, Keqlcad → 6,
sfmcadC12 → 0.38, sfmcadC10 → 0.8, sfmcadC8 → 0.87, sfmcadC6 → 1, sfmcadC4 → 0.12,
Vmcad → 0.081, KmmcadC12AcylCoAMAT → 5.7, KmmcadC10AcylCoAMAT → 5.4,
KmmcadC8AcylCoAMAT → 4, KmmcadC6AcylCoAMAT → 9.4, KmmcadC4AcylCoAMAT → 135,
KmmcadFAD → 0.12, KmmcadC12EnoylCoAMAT → 1.08, KmmcadC10EnoylCoAMAT → 1.08,
KmmcadC8EnoylCoAMAT → 1.08, KmmcadC6EnoylCoAMAT → 1.08,
KmmcadC4EnoylCoAMAT → 1.08, KmmcadFADH → 24.2, Keqmcad → 6,
sfscadC6 → 0.3, sfscadC4 → 1, Vscad → 0.081, KmscadC6AcylCoAMAT → 285,
KmscadC4AcylCoAMAT → 10.7, KmscadFAD → 0.12, KmscadC6EnoylCoAMAT → 1.08,
KmscadC4EnoylCoAMAT → 1.08, KmscadFADH → 24.2, Keqscad -> 6,
sfcrotC16 → 0.13, sfcrotC14 → 0.2, sfcrotC12 → 0.25, sfcrotC10 → 0.33, sfcrotC8 → 0.58,
sfcrotC6 → 0.83, sfcrotC4 → 1, Vcrot → 3.6, KmcrotC16EnoylCoAMAT → 150,
KmcrotC14EnoylCoAMAT → 100, KmcrotC12EnoylCoAMAT → 25, KmcrotC10EnoylCoAMAT → 25,
KmcrotC8EnoylCoAMAT → 25, KmcrotC6EnoylCoAMAT → 25, KmcrotC4EnoylCoAMAT → 40,
KmcrotC16HydroxyacylCoAMAT → 45, KmcrotC14HydroxyacylCoAMAT → 45,
KmcrotC12HydroxyacylCoAMAT → 45, KmcrotC10HydroxyacylCoAMAT → 45,
KmcrotC8HydroxyacylCoAMAT → 45, KmcrotC6HydroxyacylCoAMAT → 45,
KmcrotC4HydroxyacylCoAMAT → 45, KicrotC4AcetoacylCoA → 1.6, Keqcrot → 3.13,
sfmschadC16 → 0.6, sfmschadC14 → 0.5, sfmschadC12 → 0.43, sfmschadC10 → 0.64,
sfmschadC8 → 0.89, sfmschadC6 → 1, sfmschadC4 → 0.67, Vmschad → 1,
KmmschadC16HydroxyacylCoAMAT → 1.5, KmmschadC14HydroxyacylCoAMAT → 1.8,
KmmschadC12HydroxyacylCoAMAT → 3.7, KmmschadC10HydroxyacylCoAMAT → 8.8,
KmmschadC8HydroxyacylCoAMAT → 16.3, KmmschadC6HydroxyacylCoAMAT → 28.6,
KmmschadC4HydroxyacylCoAMAT → 69.9, KmmschadNADMAT → 58.5,
KmmschadC16KetoacylCoAMAT → 1.4, KmmschadC14KetoacylCoAMAT → 1.4,
KmmschadC12KetoacylCoAMAT → 1.6, KmmschadC10KetoacylCoAMAT → 2.3,
KmmschadC8KetoacylCoAMAT → 4.1, KmmschadC6KetoacylCoAMAT → 5.8,
KmmschadC4AcetoacylCoAMAT → 16.9, KmmschadNADHMAT → 5.4, Keqmschad → $2.17 * 10^{-4}$,
sfmckatC16 → 0, sfmckatC14 → 0.2, sfmckatC12 → 0.38, sfmckatC10 → 0.65,
sfmckatC8 → 0.81, sfmckatC6 → 1, sfmckatC4 → 0.49, Vmckat → 0.377,
KmmckatC16KetoacylCoAMAT → 1.1, KmmckatC14KetoacylCoAMAT → 1.2,

```
    KmmckatC12KetoacylCoAMAT → 1.3, KmmckatC10KetoacylCoAMAT → 2.1,
    KmmckatC8KetoacylCoAMAT → 3.2, KmmckatC6KetoacylCoAMAT → 6.7,
    KmmckatC4AcetoacylCoAMAT → 12.4, KmmckatCoAMAT → 26.6,
    KmmckatC14AcylCoAMAT → 13.83, KmmckatC16AcylCoAMAT → 13.83,
    KmmckatC12AcylCoAMAT → 13.83, KmmckatC10AcylCoAMAT → 13.83,
    KmmckatC8AcylCoAMAT → 13.83, KmmckatC6AcylCoAMAT → 13.83,
    KmmckatC4AcylCoAMAT → 13.83, KmmckatAcetylCoAMAT → 30, Keqmckat → 1051,
    sfmtpC16 → 1, sfmtpC14 → 0.9, sfmtpC12 → 0.81, sfmtpC10 → 0.73, sfmtpC8 → 0.34,
    Vmtp → 2.84, KmmtpC16EnoylCoAMAT → 25, KmmtpC14EnoylCoAMAT → 25,
    KmmtpC12EnoylCoAMAT → 25, KmmtpC10EnoylCoAMAT → 25, KmmtpC8EnoylCoAMAT → 25,
    KmmtpNADMAT → 60, KmmtpCoAMAT → 30, KmmtpC14AcylCoAMAT → 13.83,
    KmmtpC16AcylCoAMAT → 13.83, KmmtpC12AcylCoAMAT → 13.83,
    KmmtpC10AcylCoAMAT → 13.83, KmmtpC8AcylCoAMAT → 13.83, KmmtpC6AcylCoAMAT → 13.83,
    KmmtpNADHMAT → 50, KmmtpAcetylCoAMAT → 30, Keqmtp → 0.71,
    Ksacesink → 6 000 000, K1acesink → 70, Ksfadhsink → 6 000 000,
    K1fadhsink → 0.46, Ksnadhsink → 6 000 000, K1nadhsink → 12,
    C16AcylCoACYT → X, CarCYT → 200, CoACYT → 140, MalCoACYT → 0,
    CarMAT -> 950, FADtMAT → 0.77, NADtMAT → 250, CoAMATt → 5000,
    VCYT → 2.2 * 10^-6, VMAT → 1.8 * 10^-6, AcetylCoAMAT → 70, FADHMAT → 0.46, NADHMAT → 12};

tsolScan[X_, Z_] :=
  NDSolve[Join[Odes /. RateEqs /. CoAMATX /. ParmScan[X, Z], InitialConditions],
   Vars, {t, 0, 1 000 000 000}];

SsScan[X_, Z_] := Module[{SSGuess},
  SSGuess := Table[{Vars[[i]][t],
     (Vars[[i]][900 000 000] /. tsolScan[X, Z])[[1]]}, {i, 1, Length[Vars]}];
  FindRoot[Table[Odes[[i, 2]] == 0, {i, 1, Length[Odes]}] /. RateEqs /. CoAMATX /.
    ParmScan[X, Z], SSGuess]]
```

```
In[•]:= ScanDownNDSm[Ystart_, dY_, Yend_] := Monitor[Module[{SS, SSGuess},
    DataDownNDSfluxm = {};
    Xstart = 250;
    Xend = 0;
    YY = {0.014, 0.012, 0.0118, 0.0115, 0.0112};
    For[Y = Ystart, Y ≤ Yend,
     Z = YY[[Y]];
     tsolStart = tsolScan[Xend, Z];
     SSGuess = Table[{Vars[[i]][t],
        (Vars[[i]][900000000] /. tsolStart)[[1]]}, {i, 1, Length[Vars]}];
     SSGuess1 = SSGuess[[All, 1]];
     SSGuess2 = SSGuess[[All, 2]];
     SSGuess1int = SSGuess1 /. t → 0;
     InitialConditionsUD = Thread[SSGuess1int == SSGuess2];
     dX = 1;
     For[X = 250, X ≥ 0,
      (*Print[X];*)
      (*SS=FindRoot[Table[Odes[[i,2]]==0,{i,1,Length[Odes]}]/.RateEqs/.CoAMATX/.
         ParmScan[X],SSGuess,MaxIterations→Infinity];
      SSGuess={#[[1]],#[[2]]}&/@SS;*)


      tsolScanNDS = NDSolve[Join[Odes /. RateEqs /. CoAMATX /. ParmScan[X, Z],
         InitialConditionsUD], Vars, {t, 0, 1000000000}];
      SSGuess = Table[{Vars[[i]][t], (Vars[[i]][900000000] /. tsolScanNDS)[[1]]},
        {i, 1, Length[Vars]}];
      SSGuess1 = SSGuess[[All, 1]];
      SSGuess2 = SSGuess[[All, 2]];
      SSGuess1int = SSGuess1 /. t → 0;
      InitialConditionsUD = Thread[SSGuess1int == SSGuess2];
      SS = Thread[SSGuess1 → SSGuess2];

      AppendTo[DataDownNDSfluxm,
       {X, Z, 10^3 vcpt1C16 /. RateEqs /. CoAMATX /. ParmScan[X, Z] /. SS}];
      X = X - dX;];
     Y = Y + dY;]
   ], ProgressIndicator[X, {Xstart, Xend}]]



In[•]:= ScanDownNDSm[1, 1, 5]
```

```
In[•]:= ScanUpNDSm[Ystart_, dY_, Yend_] := Monitor[Module[{SS, SSGuess},
   DataUpNDSfluxm = {};
   Xstart = 0;
   Xend = 250;
   YY = {0.014, 0.012, 0.0118, 0.0115, 0.0112};
   For[Y = Ystart, Y ≤ Yend,
    Z = YY[[Y]];
    tsolStart = tsolScan[Xstart, Z ];
    SSGuess = Table[{Vars[[i]][t],
       (Vars[[i]][900000000] /. tsolStart)[[1]]}, {i, 1, Length[Vars]}];
    SSGuess1 = SSGuess[[All, 1]];
    SSGuess2 = SSGuess[[All, 2]];
    SSGuess1int = SSGuess1 /. t → 0;
    InitialConditionsUD = Thread[SSGuess1int == SSGuess2];

    dX = 1;
    For[X = 0, X ≤ 250,
     (*Print[X];*)
     (*SS=FindRoot[Table[Odes[[i,2]]==0,{i,1,Length[Odes]}]/.RateEqs/.CoAMATX/.
        ParmScan[X],SSGuess,MaxIterations→Infinity];
     SSGuess={#[[1]],#[[2]]}&/@SS;*)


     tsolScanNDS = NDSolve[Join[Odes /. RateEqs /. CoAMATX /. ParmScan[X, Z],
        InitialConditionsUD], Vars, {t, 0, 1000000000}];
     SSGuess = Table[{Vars[[i]][t], (Vars[[i]][900000000] /. tsolScanNDS)[[1]]},
       {i, 1, Length[Vars]}];
     SSGuess1 = SSGuess[[All, 1]];
     SSGuess2 = SSGuess[[All, 2]];
     SSGuess1int = SSGuess1 /. t → 0;
     InitialConditionsUD = Thread[SSGuess1int == SSGuess2];
     SS = Thread[SSGuess1 → SSGuess2];

     AppendTo[DataUpNDSfluxm,
      {X, Z, 10^3 vcpt1C16 /. RateEqs /. CoAMATX /. ParmScan[X, Z] /. SS}];

     X = X + dX;];
    Y = Y + dY;]
   ], ProgressIndicator[X, {Xstart, Xend}]]


In[•]:= ScanUpNDSm[1, 1, 5]

In[•]:=

In[•]:=

In[•]:= (*Export[
  "C:\\Users\\Kindergeneeskunde\\Documents\\FentawNewlaptopPediatrics\\Code_mathematica
    \\mFAOvaryingMalonylCoA.xls",
  {"forwardFlux" -> DataUpNDSfluxm, "reverseFlux" -> DataDownNDSfluxm}]*)
```

In[ ]:=
```
p71 =
 ListLinePlot[{DataUpNDSfluxm[[1 ;; 251, {1, 3}]], DataUpNDSfluxm[[252 ;; 502, {1, 3}]],
   DataUpNDSfluxm[[503 ;; 753, {1, 3}]], DataUpNDSfluxm[[754 ;; 1004, {1, 3}]],
   DataUpNDSfluxm[[1005 ;; 1255, {1, 3}]], DataDownNDSfluxm[[1 ;; 251, {1, 3}]],
   DataDownNDSfluxm[[252 ;; 502, {1, 3}]], DataDownNDSfluxm[[503 ;; 753, {1, 3}]],
   DataDownNDSfluxm[[754 ;; 1004, {1, 3}]], DataDownNDSfluxm[[1005 ;; 1255, {1, 3}]] },
  PlotRange → All, PlotStyle → {Magenta, Blue, Cyan, Green,
    Darker[Yellow], Magenta, Blue, Cyan, Green, Darker[Yellow]},
  AxesStyle → Directive[Black, 18], LabelStyle → Directive[Black, 14],
  PlotLegends → {"0.0140", "0.0120", "0.0118", "0.0115" , "0.0112"},
  PlotLabel → "Vmax of CPT1(μmol.min^-1.gProtein^-1)",
  Frame → {{True, False}, {True, False}},
  FrameLabel → {{"Flux(μmol.min^-1.gProtein^-1)", None}, {"Palmitoyl-CoA(μM)", None}},
  BaseStyle → {FontSize → 16, FontWeight → ""},
  FrameStyle → Thickness[0.00005], ImageSize → Scaled[0.25], AspectRatio → 1]
```

Out[ ]=

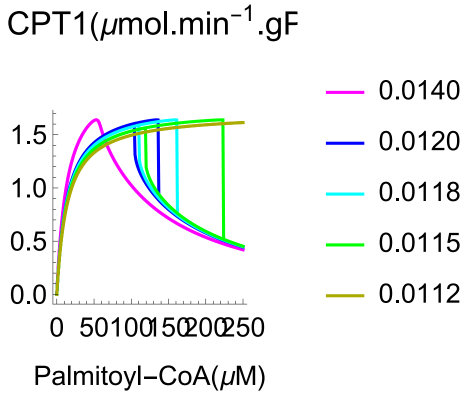

G. Steady state computation with varying palmitoyl-CoA (X) and MCKAT Vmax (Z)

In[ ]:=
```
ParmScan[X_, Z_] := {
   sfcpt1C16 → 1, Vcpt1 → 0.012, Kmcpt1C16AcylCoACYT → 13.8,
   Kmcpt1CarCYT → 250, Kmcpt1C16AcylCarCYT → 136, Kmcpt1CoACYT → 40.7,
   Kicpt1MalCoACYT → 9.1, Keqcpt1 → 0.45, ncpt1 → 2.4799,
   Vfcact → 0.42, Vrcact → 0.42, KmcactC16AcylCarCYT → 15,
   KmcactC14AcylCarCYT → 15, KmcactC12AcylCarCYT → 15, KmcactC10AcylCarCYT → 15,
   KmcactC8AcylCarCYT → 15, KmcactC6AcylCarCYT → 15, KmcactC4AcylCarCYT → 15,
   KmcactCarMAT → 130, KmcactC16AcylCarMAT → 15, KmcactC14AcylCarMAT → 15,
   KmcactC12AcylCarMAT → 15, KmcactC10AcylCarMAT → 15, KmcactC8AcylCarMAT → 15,
   KmcactC6AcylCarMAT → 15, KmcactC4AcylCarMAT → 15, KmcactCarCYT → 130,
   KicactC16AcylCarCYT → 56, KicactC14AcylCarCYT → 56, KicactC12AcylCarCYT → 56,
   KicactC10AcylCarCYT → 56, KicactC8AcylCarCYT → 56, KicactC6AcylCarCYT → 56,
   KicactC4AcylCarCYT → 56, KicactCarCYT → 200, Keqcact → 1,
   sfcpt2C16 → 0.85, sfcpt2C14 → 1, sfcpt2C12 → 0.95, sfcpt2C10 → 0.95,
   sfcpt2C8 → 0.35, sfcpt2C6 → 0.15, sfcpt2C4 → 0.01, Vcpt2 → 0.391,
   Kmcpt2C16AcylCarMAT → 51, Kmcpt2C14AcylCarMAT → 51, Kmcpt2C12AcylCarMAT → 51,
   Kmcpt2C10AcylCarMAT → 51, Kmcpt2C8AcylCarMAT → 51, Kmcpt2C6AcylCarMAT → 51,
   Kmcpt2C4AcylCarMAT → 51, Kmcpt2CoAMAT → 30, Kmcpt2C16AcylCoAMAT → 38,
```

```
Kmcpt2C14AcylCoAMAT → 38, Kmcpt2C12AcylCoAMAT → 38,
Kmcpt2C10AcylCoAMAT → 38, Kmcpt2C8AcylCoAMAT → 38, Kmcpt2C6AcylCoAMAT → 1000,
Kmcpt2C4AcylCoAMAT → 1000000, Kmcpt2CarMAT → 350, Keqcpt2 → 2.22,
sfvlcadC16 → 1, sfvlcadC14 → 0.42, sfvlcadC12 → 0.11, Vvlcad → 0.008,
KmvlcadC16AcylCoAMAT → 6.5, KmvlcadC14AcylCoAMAT → 4, KmvlcadC12AcylCoAMAT → 2.7,
KmvlcadFAD → 0.12, KmvlcadC16EnoylCoAMAT → 1.08, KmvlcadC14EnoylCoAMAT → 1.08,
KmvlcadC12EnoylCoAMAT → 1.08, KmvlcadFADH → 24.2, Keqvlcad → 6,
sflcadC16 → 0.9, sflcadC14 → 1, sflcadC12 → 0.9, sflcadC10 → 0.75, sflcadC8 → 0.4,
Vlcad → 0.01, KmlcadC16AcylCoAMAT → 2.5, KmlcadC14AcylCoAMAT → 7.4,
KmlcadC12AcylCoAMAT → 9, KmlcadC10AcylCoAMAT → 24.3, KmlcadC8AcylCoAMAT → 123,
KmlcadFAD → 0.12, KmlcadC16EnoylCoAMAT → 1.08, KmlcadC14EnoylCoAMAT → 1.08,
KmlcadC12EnoylCoAMAT → 1.08, KmlcadC10EnoylCoAMAT → 1.08,
KmlcadC8EnoylCoAMAT → 1.08, KmlcadFADH → 24.2, Keqlcad → 6,
sfmcadC12 → 0.38, sfmcadC10 → 0.8, sfmcadC8 → 0.87, sfmcadC6 → 1, sfmcadC4 → 0.12,
Vmcad → 0.081, KmmcadC12AcylCoAMAT → 5.7, KmmcadC10AcylCoAMAT → 5.4,
KmmcadC8AcylCoAMAT → 4, KmmcadC6AcylCoAMAT → 9.4, KmmcadC4AcylCoAMAT → 135,
KmmcadFAD → 0.12, KmmcadC12EnoylCoAMAT → 1.08, KmmcadC10EnoylCoAMAT → 1.08,
KmmcadC8EnoylCoAMAT → 1.08, KmmcadC6EnoylCoAMAT → 1.08,
KmmcadC4EnoylCoAMAT → 1.08, KmmcadFADH → 24.2, Keqmcad → 6,
sfscadC6 → 0.3, sfscadC4 → 1, Vscad → 0.081, KmscadC6AcylCoAMAT → 285,
KmscadC4AcylCoAMAT → 10.7, KmscadFAD → 0.12, KmscadC6EnoylCoAMAT → 1.08,
KmscadC4EnoylCoAMAT → 1.08, KmscadFADH → 24.2, Keqscad -> 6,
sfcrotC16 → 0.13, sfcrotC14 → 0.2, sfcrotC12 → 0.25, sfcrotC10 → 0.33, sfcrotC8 → 0.58,
sfcrotC6 → 0.83, sfcrotC4 → 1, Vcrot → 3.6, KmcrotC16EnoylCoAMAT → 150,
KmcrotC14EnoylCoAMAT → 100, KmcrotC12EnoylCoAMAT → 25, KmcrotC10EnoylCoAMAT → 25,
KmcrotC8EnoylCoAMAT → 25, KmcrotC6EnoylCoAMAT → 25, KmcrotC4EnoylCoAMAT → 40,
KmcrotC16HydroxyacylCoAMAT → 45, KmcrotC14HydroxyacylCoAMAT → 45,
KmcrotC12HydroxyacylCoAMAT → 45, KmcrotC10HydroxyacylCoAMAT → 45,
KmcrotC8HydroxyacylCoAMAT → 45, KmcrotC6HydroxyacylCoAMAT → 45,
KmcrotC4HydroxyacylCoAMAT → 45, KicrotC4AcetoacylCoA → 1.6, Keqcrot → 3.13,
sfmschadC16 → 0.6, sfmschadC14 → 0.5, sfmschadC12 → 0.43, sfmschadC10 → 0.64,
sfmschadC8 → 0.89, sfmschadC6 → 1, sfmschadC4 → 0.67, Vmschad → 1,
KmmschadC16HydroxyacylCoAMAT → 1.5, KmmschadC14HydroxyacylCoAMAT → 1.8,
KmmschadC12HydroxyacylCoAMAT → 3.7, KmmschadC10HydroxyacylCoAMAT → 8.8,
KmmschadC8HydroxyacylCoAMAT → 16.3, KmmschadC6HydroxyacylCoAMAT → 28.6,
KmmschadC4HydroxyacylCoAMAT → 69.9, KmmschadNADMAT → 58.5,
KmmschadC16KetoacylCoAMAT → 1.4, KmmschadC14KetoacylCoAMAT → 1.4,
KmmschadC12KetoacylCoAMAT → 1.6, KmmschadC10KetoacylCoAMAT → 2.3,
KmmschadC8KetoacylCoAMAT → 4.1, KmmschadC6KetoacylCoAMAT → 5.8,
KmmschadC4AcetoacylCoAMAT → 16.9, KmmschadNADHMAT → 5.4, Keqmschad → 2.17 * 10^-4,
sfmckatC16 → 0, sfmckatC14 → 0.2, sfmckatC12 → 0.38, sfmckatC10 → 0.65,
sfmckatC8 → 0.81, sfmckatC6 → 1, sfmckatC4 → 0.49, Vmckat →  Z(*0.377*),
KmmckatC16KetoacylCoAMAT → 1.1, KmmckatC14KetoacylCoAMAT → 1.2,
KmmckatC12KetoacylCoAMAT → 1.3, KmmckatC10KetoacylCoAMAT → 2.1,
KmmckatC8KetoacylCoAMAT → 3.2, KmmckatC6KetoacylCoAMAT → 6.7,
KmmckatC4AcetoacylCoAMAT → 12.4, KmmckatCoAMAT → 26.6,
KmmckatC14AcylCoAMAT → 13.83, KmmckatC16AcylCoAMAT → 13.83,
KmmckatC12AcylCoAMAT → 13.83, KmmckatC10AcylCoAMAT → 13.83,
KmmckatC8AcylCoAMAT → 13.83, KmmckatC6AcylCoAMAT → 13.83,
KmmckatC4AcylCoAMAT → 13.83, KmmckatAcetylCoAMAT → 30, Keqmckat → 1051,
sfmtpC16 → 1, sfmtpC14 → 0.9, sfmtpC12 → 0.81, sfmtpC10 → 0.73, sfmtpC8 → 0.34,
Vmtp → 2.84, KmmtpC16EnoylCoAMAT → 25, KmmtpC14EnoylCoAMAT → 25,
KmmtpC12EnoylCoAMAT → 25, KmmtpC10EnoylCoAMAT → 25, KmmtpC8EnoylCoAMAT → 25,
KmmtpNADMAT → 60, KmmtpCoAMAT → 30, KmmtpC14AcylCoAMAT → 13.83,
KmmtpC16AcylCoAMAT → 13.83, KmmtpC12AcylCoAMAT → 13.83,
```

```
    KmmtpC10AcylCoAMAT → 13.83, KmmtpC8AcylCoAMAT → 13.83, KmmtpC6AcylCoAMAT → 13.83,
    KmmtpNADHMAT → 50, KmmtpAcetylCoAMAT → 30, Keqmtp → 0.71,
    Ksacesink → 6 000 000, K1acesink → 70, Ksfadhsink → 6 000 000,
    K1fadhsink → 0.46, Ksnadhsink → 6 000 000, K1nadhsink → 12,
    C16AcylCoACYT → X, CarCYT → 200, CoACYT → 140, MalCoACYT → 0,
    CarMAT -> 950, FADtMAT → 0.77, NADtMAT → 250, CoAMATt → 5000,
    VCYT → 2.2 * 10^-6, VMAT → 1.8 * 10^-6, AcetylCoAMAT → 70, FADHMAT → 0.46, NADHMAT → 12};

tsolScan[X_, Z_] :=
  NDSolve[Join[Odes /. RateEqs /. CoAMATX /. ParmScan[X, Z], InitialConditions],
   Vars, {t, 0, 1 000 000 000}];

SsScan[X_, Z_] := Module[{SSGuess},
  SSGuess := Table[{Vars[[i]][t],
     (Vars[[i]][900 000 000] /. tsolScan[X, Z])[[1]]}, {i, 1, Length[Vars]}];
  FindRoot[Table[Odes[[i, 2]] == 0, {i, 1, Length[Odes]}] /. RateEqs /. CoAMATX /.
    ParmScan[X, Z], SSGuess]]
```

In[•]:=
```
ScanDownNDSm[Ystart_, dY_, Yend_] := Monitor[Module[{SS, SSGuess},
   DataDownNDSfluxmc = {};
   DataDownNDSfluxmck = {};
   Xstart = 250;
   Xend = 0;
   YY = {0.320, 0.350, 0.377, 0.400, 0.500};
   For[Y = Ystart, Y ≤ Yend,
    Z = YY[[Y]];
    tsolStart = tsolScan[Xend, Z];
    SSGuess = Table[{Vars[[i]][t],
       (Vars[[i]][900 000 000] /. tsolStart)[[1]]}, {i, 1, Length[Vars]}];
    SSGuess1 = SSGuess[[All, 1]];
    SSGuess2 = SSGuess[[All, 2]];
    SSGuess1int = SSGuess1 /. t → 0;
    InitialConditionsUD = Thread[SSGuess1int == SSGuess2];
    dX = 1;
    For[X = 250, X ≥ 0,
     (*Print[X];*)
     (*SS=FindRoot[Table[Odes[[i,2]]==0,{i,1,Length[Odes]}]/.RateEqs/.CoAMATX/.
        ParmScan[X],SSGuess,MaxIterations→Infinity];
     SSGuess={#[[1]],#[[2]]}&/@SS;*)


     tsolScanNDS = NDSolve[Join[Odes /. RateEqs /. CoAMATX /. ParmScan[X, Z],
        InitialConditionsUD], Vars, {t, 0, 1 000 000 000}];
     SSGuess = Table[{Vars[[i]][t], (Vars[[i]][900 000 000] /. tsolScanNDS)[[1]]},
       {i, 1, Length[Vars]}];
     SSGuess1 = SSGuess[[All, 1]];
     SSGuess2 = SSGuess[[All, 2]];
     SSGuess1int = SSGuess1 /. t → 0;
     InitialConditionsUD = Thread[SSGuess1int == SSGuess2];
     SS = Thread[SSGuess1 → SSGuess2];

     AppendTo[DataDownNDSfluxmc,
      {X, Z, 10^3 vcpt1C16 /. RateEqs /. CoAMATX /. ParmScan[X, Z] /. SS}];
     AppendTo[DataDownNDSfluxmck, {X, Z,
       10^3 vmckatC4 /. RateEqs /. CoAMATX /. ParmScan[X, Z] /. SS}];
     X = X - dX;];
    Y = Y + dY;]
   ], ProgressIndicator[X, {Xstart, Xend}]]
```

In[•]:=
```
ScanDownNDSm[1, 1, 5]
```

In[•]:=
```
ScanUpNDSm[Ystart_, dY_, Yend_] := Monitor[Module[{SS, SSGuess},
    DataUpNDSfluxmc = {};
    DataUpNDSfluxmck = {};
    Xstart = 0;
    Xend = 250;
    YY = {0.320, 0.350, 0.377, 0.400, 0.500};
    For[Y = Ystart, Y ≤ Yend,
     Z = YY[[Y]];
     tsolStart = tsolScan[Xstart, Z ];
     SSGuess = Table[{Vars[[i]][t],
        (Vars[[i]][900000000] /. tsolStart)[[1]]}, {i, 1, Length[Vars]}];
     SSGuess1 = SSGuess[[All, 1]];
     SSGuess2 = SSGuess[[All, 2]];
     SSGuess1int = SSGuess1 /. t → 0;
     InitialConditionsUD = Thread[SSGuess1int == SSGuess2];

     dX = 1;
     For[X = 0, X ≤ 250,
      (*Print[X];*)
      (*SS=FindRoot[Table[Odes[[i,2]]==0,{i,1,Length[Odes]}]/.RateEqs/.CoAMATX/.
         ParmScan[X],SSGuess,MaxIterations→Infinity];
      SSGuess={#[[1]],#[[2]]}&/@SS;*)


      tsolScanNDS = NDSolve[Join[Odes /. RateEqs /. CoAMATX /. ParmScan[X, Z],
         InitialConditionsUD], Vars, {t, 0, 1000000000}];
      SSGuess = Table[{Vars[[i]][t], (Vars[[i]][900000000] /. tsolScanNDS)[[1]]},
        {i, 1, Length[Vars]}];
      SSGuess1 = SSGuess[[All, 1]];
      SSGuess2 = SSGuess[[All, 2]];
      SSGuess1int = SSGuess1 /. t → 0;
      InitialConditionsUD = Thread[SSGuess1int == SSGuess2];
      SS = Thread[SSGuess1 → SSGuess2];

      AppendTo[DataUpNDSfluxmc,
       {X, Z, 10^3 vcpt1C16 /. RateEqs /. CoAMATX /. ParmScan[X, Z] /. SS}];
      AppendTo[DataUpNDSfluxmck, {X, Z,
        10^3 vmckatC4 /. RateEqs /. CoAMATX /. ParmScan[X, Z] /. SS}];

      X = X + dX;];
     Y = Y + dY;]
   ], ProgressIndicator[X, {Xstart, Xend}]]
```

In[•]:=
```
ScanUpNDSm[1, 1, 5]
```

In[•]:=

In[•]:=

In[ ]:=
```
(*Export[
 "C:\\Users\\Kindergeneeskunde\\Documents\\FentawNewlaptopPediatrics\\Code_mathematica
   \\mFAOvaryingMalonylCoA.xls",
 {"forwardFlux" -> DataUpNDSfluxm, "reverseFlux" -> DataDownNDSfluxm}]*)
```

In[ ]:=

```
p8cpt1c16 = ListLinePlot[{DataUpNDSfluxmc[[1 ;; 251, {1, 3}]],
    DataUpNDSfluxmc[[252 ;; 502, {1, 3}]], DataUpNDSfluxmc[[503 ;; 753, {1, 3}]],
    DataUpNDSfluxmc[[754 ;; 1004, {1, 3}]], DataUpNDSfluxmc[[1005 ;; 1255, {1, 3}]],
    DataDownNDSfluxmc[[1 ;; 251, {1, 3}]], DataDownNDSfluxmc[[252 ;; 502, {1, 3}]],
    DataDownNDSfluxmc[[503 ;; 753, {1, 3}]], DataDownNDSfluxmc[[754 ;; 1004, {1, 3}]],
    DataDownNDSfluxmc[[1005 ;; 1255, {1, 3}]] },
  PlotRange → All, PlotStyle → {Magenta, Blue, Cyan, Green,
    Darker[Yellow], Magenta, Blue, Cyan, Green, Darker[Yellow]},
  AxesStyle → Directive[Black, 18], LabelStyle → Directive[Black, 14],
  PlotLegends → {"0.320", "0.350", "0.377", "0.400", "0.500"},
  PlotLabel → "Vmax of MCKAT(μmol.min^-1.gProtein^-1)",
  Frame → {{True, False}, {True, False}},
  FrameLabel → {{"Flux(μmol.min^-1.gProtein^-1)", None}, {"Palmitoyl-CoA(μM)", None}},
  BaseStyle → {FontSize → 18, FontWeight → ""},
  FrameStyle → Thickness[0.00005], ImageSize → Scaled[0.25], AspectRatio → 1]
```
