# Supplementary material for: Bistability in fatty-acid oxidation resulting from substrate inhibition
Source: PLoS Comput Biol. 2021 Aug 12;17(8):e1009259. doi: 10.1371/journal.pcbi.1009259 (PMC8396765; doi:10.1371/journal.pcbi.1009259)

Mitochondrial Fatty Acid Oxidation Kinetic Model Extension with SCFAs Regulation

Definitions of the various functions(CPT1 and NAD production equations are modified to be a function of Acetate concentration in the liver: AcetateMAT=0 with out acetate treatment and AcetateMAT=1 with 3 mM acetate treatment)

In[∘]:= CPT1[sf_, V_, Kms1_, Kms2_, Kmp1_, Kmp2_, Ki1_, Keq_, S1_, S2_, P1_, P2_, I1_, n_, vsh_, Kmsh_, SH1_, propacetcpt1_, propacetmal_, nc_] :=

$$\frac{sf * ((1 + SH1) * V) * \left(\frac{S1*S2}{Kms1*Kms2} - \frac{P1*P2}{Kms1*Kms2*Keq}\right)}{\left(1 + \frac{S1}{Kms1} + \frac{P1}{Kmp1} + \left(\frac{I1}{Ki1}\right)^{n}\right) * \left(1 + \frac{S2}{Kms2} + \frac{P2}{Kmp2}\right)}$$

In[∘]:=

In[∘]:= CACT[Vf_, Vr_, Kms1_, Kms2_, Kmp1_, Kmp2_, Kis1_, Kip2_, Keq_, S1_, S2_, P1_, P2_, vsh_, Kmsh_, SH1_, propacetcact_] :=

$$\frac{Vf * \left(S1 * S2 - \frac{P1*P2}{Keq}\right)}{S1 * S2 + Kms2 * S1 + Kms1 * S2 * \left(1 + \frac{P2}{Kip2}\right) + \frac{Vf}{Vr*Keq} * \left(Kmp2 * P1 * \left(1 + \frac{S1}{Kis1}\right) + P2 * (Kmp1 + P1)\right)}$$

In[∘]:= CPT2[sf_, V_, Kms1_, Kms2_, Kms3_, Kms4_, Kms5_, Kms6_, Kms7_, Kms8_, Kmp1_, Kmp2_, Kmp3_, Kmp4_, Kmp5_, Kmp6_, Kmp7_, Kmp8_, Keq_, S1_, S2_, S3_, S4_, S5_, S6_, S7_, S8_, P1_, P2_, P3_, P4_, P5_, P6_, P7_, P8_, vsh_, Kmsh_, SH1_, propacetcpt2_] :=

$$\left(sf * \left(V * \left(\frac{S1 * S8}{Kms1 * Kms8} - \frac{P1 * P8}{Kms1 * Kms8 * Keq}\right)\right)\right) \Big/ \left(\left(1 + \frac{S1}{Kms1} + \frac{P1}{Kmp1} + \frac{S2}{Kms2} + \frac{P2}{Kmp2} + \frac{S3}{Kms3} + \frac{P3}{Kmp3} + \frac{S4}{Kms4} + \frac{P4}{Kmp4} + \frac{S5}{Kms5} + \frac{P5}{Kmp5} + \frac{S6}{Kms6} + \frac{P6}{Kmp6} + \frac{S7}{Kms7} + \frac{P7}{Kmp7}\right) * \left(1 + \frac{S8}{Kms8} + \frac{P8}{Kmp8}\right)\right)$$

In[∘]:= VLCAD[sf_, V_, Kms1_, Kms2_, Kms3_, Kms4_, Kmp1_, Kmp2_, Kmp3_, Kmp4_, Keq_, S1_, S2_, S3_, S4_, P1_, P2_, P3_, P4_] :=

$$\frac{sf * V * \left(\frac{S1*(S4-P4)}{Kms1*Kms4} - \frac{P1*P4}{Kms1*Kms4*Keq}\right)}{\left(1 + \frac{S1}{Kms1} + \frac{P1}{Kmp1} + \frac{S2}{Kms2} + \frac{P2}{Kmp2} + \frac{S3}{Kms3} + \frac{P3}{Kmp3}\right) * \left(1 + \frac{(S4-P4)}{Kms4} + \frac{P4}{Kmp4}\right)}$$

In[∘]:= LCAD[sf_, V_, Kms1_, Kms2_, Kms3_, Kms4_, Kms5_, Kms6_, Kmp1_, Kmp2_, Kmp3_, Kmp4_, Kmp5_, Kmp6_, Keq_, S1_, S2_, S3_, S4_, S5_, S6_, P1_, P2_, P3_, P4_, P5_, P6_] :=

$$\frac{sf * V * \left(\frac{S1*(S6-P6)}{Kms1*Kms6} - \frac{P1*P6}{Kms1*Kms6*Keq}\right)}{\left(1 + \frac{S1}{Kms1} + \frac{P1}{Kmp1} + \frac{S2}{Kms2} + \frac{P2}{Kmp2} + \frac{S3}{Kms3} + \frac{P3}{Kmp3} + \frac{S4}{Kms4} + \frac{P4}{Kmp4} + \frac{S5}{Kms5} + \frac{P5}{Kmp5}\right) * \left(1 + \frac{(S6-P6)}{Kms6} + \frac{P6}{Kmp6}\right)}$$

In[ ]:=
$$\text{MCAD[sf\_, V\_, Kms1\_, Kms2\_, Kms3\_, Kms4\_, Kms5\_, Kms6\_, Kmp1\_, Kmp2\_, Kmp3\_, Kmp4\_, Kmp5\_, Kmp6\_, Keq\_, S1\_, S2\_, S3\_, S4\_, S5\_, S6\_, P1\_, P2\_, P3\_, P4\_, P5\_, P6\_]} := \frac{sf*V*\left(\frac{S1*(S6-P6)}{Kms1*Kms6}-\frac{P1*P6}{Kms1*Kms6*Keq}\right)}{\left(1+\frac{S1}{Kms1}+\frac{P1}{Kmp1}+\frac{S2}{Kms2}+\frac{P2}{Kmp2}+\frac{S3}{Kms3}+\frac{P3}{Kmp3}+\frac{S4}{Kms4}+\frac{P4}{Kmp4}+\frac{S5}{Kms5}+\frac{P5}{Kmp5}\right)*\left(1+\frac{(S6-P6)}{Kms6}+\frac{P6}{Kmp6}\right)}$$

In[ ]:=
$$\text{SCAD[sf\_, V\_, Kms1\_, Kms2\_, Kms3\_, Kmp1\_, Kmp2\_, Kmp3\_, Keq\_, S1\_, S2\_, S3\_, P1\_, P2\_, P3\_]} := \frac{sf*V*\left(\frac{S1*(S3-P3)}{Kms1*Kms3}-\frac{P1*P3}{Kms1*Kms3*Keq}\right)}{\left(1+\frac{S1}{Kms1}+\frac{P1}{Kmp1}+\frac{S2}{Kms2}+\frac{P2}{Kmp2}\right)*\left(1+\frac{(S3-P3)}{Kms3}+\frac{P3}{Kmp3}\right)}$$

In[ ]:=
$$\text{CROT[sf\_, V\_, Kms1\_, Kms2\_, Kms3\_, Kms4\_, Kms5\_, Kms6\_, Kms7\_, Kmp1\_, Kmp2\_, Kmp3\_, Kmp4\_, Kmp5\_, Kmp6\_, Kmp7\_, Ki1\_, Keq\_, S1\_, S2\_, S3\_, S4\_, S5\_, S6\_, S7\_, P1\_, P2\_, P3\_, P4\_, P5\_, P6\_, P7\_, I1\_]} := \frac{sf*V*\left(\frac{S1}{Kms1}-\frac{P1}{Kms1*Keq}\right)}{1+\frac{S1}{Kms1}+\frac{P1}{Kmp1}+\frac{S2}{Kms2}+\frac{P2}{Kmp2}+\frac{S3}{Kms3}+\frac{P3}{Kmp3}+\frac{S4}{Kms4}+\frac{P4}{Kmp4}+\frac{S5}{Kms5}+\frac{P5}{Kmp5}+\frac{S6}{Kms6}+\frac{P6}{Kmp6}+\frac{S7}{Kms7}+\frac{P7}{Kmp7}+\frac{I1}{Ki1}}$$

In[ ]:=
$$\text{MSCHAD[sf\_, V\_, Kms1\_, Kms2\_, Kms3\_, Kms4\_, Kms5\_, Kms6\_, Kms7\_, Kms8\_, Kmp1\_, Kmp2\_, Kmp3\_, Kmp4\_, Kmp5\_, Kmp6\_, Kmp7\_, Kmp8\_, Keq\_, S1\_, S2\_, S3\_, S4\_, S5\_, S6\_, S7\_, S8\_, P1\_, P2\_, P3\_, P4\_, P5\_, P6\_, P7\_, P8\_]} := \left(sf*V*\left(\frac{S1*(S8-P8)}{Kms1*Kms8}-\frac{P1*P8}{Kms1*Kms8*Keq}\right)\right) \Big/ \left(\left(1+\frac{S1}{Kms1}+\frac{P1}{Kmp1}+\frac{S2}{Kms2}+\frac{P2}{Kmp2}+\frac{S3}{Kms3}+\frac{P3}{Kmp3}+\frac{S4}{Kms4}+\frac{P4}{Kmp4}+\frac{S5}{Kms5}+\frac{P5}{Kmp5}+\frac{S6}{Kms6}+\frac{P6}{Kmp6}+\frac{S7}{Kms7}+\frac{P7}{Kmp7}\right)*\left(1+\frac{(S8-P8)}{Kms8}+\frac{P8}{Kmp8}\right)\right)$$

In[ ]:=
$$\text{MCKATA[sf\_, V\_, Kms1\_, Kms2\_, Kms3\_, Kms4\_, Kms5\_, Kms6\_, Kms7\_, Kms8\_, Kmp1\_, Kmp2\_, Kmp3\_, Kmp4\_, Kmp5\_, Kmp6\_, Kmp7\_, Kmp8\_, Keq\_, S1\_, S2\_, S3\_, S4\_, S5\_, S6\_, S7\_, S8\_, P1\_, P2\_, P3\_, P4\_, P5\_, P6\_, P7\_, P8\_, E1\_, KmE1\_, nm\_, vsh\_, Kmsh\_, SH1\_, propacetmckat\_]} := \left(sf*\left(V*\left(\frac{S1*S8}{Kms1*Kms8}-\frac{P1*P8}{Kms1*Kms8*Keq}\right)\right)\right) \Big/ \left(\left(1+\frac{S1}{Kms1}+\frac{P1}{Kmp1}+\frac{S2}{Kms2}+\frac{P2}{Kmp2}+\frac{S3}{Kms3}+\frac{P3}{Kmp3}+\frac{S4}{Kms4}+\frac{P4}{Kmp4}+\frac{S5}{Kms5}+\frac{P5}{Kmp5}+\frac{S6}{Kms6}+\frac{P6}{Kmp6}+\frac{S7}{Kms7}+\frac{P7}{Kmp7}+\frac{P8}{Kmp8}\right)*\left(1+\frac{S8}{Kms8}+\frac{P8}{Kmp8}\right)\right)$$

In[ ]:=
$$\text{MCKATB[sf\_, V\_, Kms1\_, Kms2\_, Kms3\_, Kms4\_, Kms5\_, Kms6\_, Kms7\_, Kms8\_, Kmp1\_, Kmp2\_, Kmp3\_, Kmp4\_, Kmp5\_, Kmp6\_, Kmp7\_, Kmp8\_, Keq\_, S1\_, S2\_, S3\_, S4\_, S5\_, S6\_, S7\_, S8\_, P1\_, P2\_, P3\_, P4\_, P5\_, P6\_, P7\_, P8\_, E1\_, KmE1\_, nm\_, vsh\_, Kmsh\_, SH1\_, propacetmckat\_]} := \left(sf*\left(V*\left(\frac{S1*S8}{Kms1*Kms8}-\frac{P8*P8}{Kms1*Kms8*Keq}\right)\right)\right) \Big/ \left(\left(1+\frac{S1}{Kms1}+\frac{P1}{Kmp1}+\frac{S2}{Kms2}+\frac{P2}{Kmp2}+\frac{S3}{Kms3}+\frac{P3}{Kmp3}+\frac{S4}{Kms4}+\frac{P4}{Kmp4}+\frac{S5}{Kms5}+\frac{P5}{Kmp5}+\frac{S6}{Kms6}+\frac{P6}{Kmp6}+\frac{S7}{Kms7}+\frac{P7}{Kmp7}+\frac{P8}{Kmp8}\right)*\left(1+\frac{S8}{Kms8}+\frac{P8}{Kmp8}\right)\right) \text{ (* }*\left(1+nm*\left(\frac{E1}{KmE1}\right)^{1}\right)\text{*)}$$

In[◦]:=
```
MTP[sf_, V_, Kms1_, Kms2_, Kms3_, Kms4_, Kms5_, Kms7_, Kms8_, Kmp1_,
  Kmp2_, Kmp3_, Kmp4_, Kmp5_, Kmp6_, Kmp7_, Kmp8_, Ki1_, Keq_, S1_, S2_,
  S3_, S4_, S5_, S7_, S8_, P1_, P2_, P3_, P4_, P5_, P6_, P7_, P8_, I1_] :=
 (sf * V * (S1 * (S7 - P7) * S8 / (Kms1 * Kms7 * Kms8) - P1 * P7 * P8 / (Kms1 * Kms7 * Kms8 * Keq))) /
 ((1 + S1/Kms1 + P1/Kmp1 + S2/Kms2 + P2/Kmp2 + S3/Kms3 + P3/Kmp3 + S4/Kms4 + P4/Kmp4 + S5/Kms5 + P5/Kmp5 + P6/Kmp6 + I1/Ki1) *
   (1 + (S7 - P7)/Kms7 + P7/Kmp7) * (1 + S8/Kms8 + P8/Kmp8))
```

In[◦]:=
```
RES[Ks_, S_, K1_] := Ks * (S - K1)
```

```
OXPH[V_, Kms1_, Kmp1_, S1_, P1_, vsh_, Kmsh_, SH1_] := ((1 + SH1) * V) * (S1/Kms1) / (1 + S1/Kms1 + P1/Kmp1)
```

In[◦]:=
```
PDH[V_, Kms1_, Kmp1_, S1_, P1_] := V * (S1/Kms1) / (1 + S1/Kms1 + P1/Kmp1)
```

In[◦]:=

Define the differential equations

```
Odes = {
  C16AcylCarCYT'[t] == (vcpt1C16 - vcactC16)/VCYT,
  C16AcylCarMAT'[t] == (vcactC16 - vcpt2C16)/VMAT,
  C16AcylCoAMAT'[t] == (vcpt2C16 - vvlcadC16 - vlcadC16)/VMAT,
  C16EnoylCoAMAT'[t] == (vvlcadC16 + vlcadC16 - vcrotC16 - vmtpC16)/VMAT,
  C16HydroxyacylCoAMAT'[t] == (vcrotC16 - vmschadC16)/VMAT,
  C16KetoacylCoAMAT'[t] == (vmschadC16 - vmckatC16)/VMAT,
  C14AcylCarCYT'[t] == (-vcactC14)/VCYT,
  C14AcylCarMAT'[t] == (vcactC14 - vcpt2C14)/VMAT,
  C14AcylCoAMAT'[t] == (vcpt2C14 + vmtpC16 + vmckatC16 - vvlcadC14 - vlcadC14)/VMAT,
  C14EnoylCoAMAT'[t] == (vvlcadC14 + vlcadC14 - vcrotC14 - vmtpC14)/VMAT,
  C14HydroxyacylCoAMAT'[t] == (vcrotC14 - vmschadC14)/VMAT,
  C14KetoacylCoAMAT'[t] == (vmschadC14 - vmckatC14)/VMAT,
```

$$C12AcylCarCYT'[t] == \frac{-vcactC12}{VCYT},$$
$$C12AcylCarMAT'[t] == \frac{vcactC12 - vcpt2C12}{VMAT},$$
$$C12AcylCoAMAT'[t] == \frac{vcpt2C12 + vmtpC14 + vmckatC14 - vvlcadC12 - vlcadC12 - vmcadC12}{VMAT},$$
$$C12EnoylCoAMAT'[t] == \frac{vvlcadC12 + vlcadC12 + vmcadC12 - vcrotC12 - vmtpC12}{VMAT},$$
$$C12HydroxyacylCoAMAT'[t] == \frac{vcrotC12 - vmschadC12}{VMAT},$$
$$C12KetoacylCoAMAT'[t] == \frac{vmschadC12 - vmckatC12}{VMAT},$$
$$C10AcylCarCYT'[t] == \frac{-vcactC10}{VCYT},$$
$$C10AcylCarMAT'[t] == \frac{vcactC10 - vcpt2C10}{VMAT},$$
$$C10AcylCoAMAT'[t] == \frac{vcpt2C10 + vmtpC12 + vmckatC12 - vlcadC10 - vmcadC10}{VMAT},$$
$$C10EnoylCoAMAT'[t] == \frac{vlcadC10 + vmcadC10 - vcrotC10 - vmtpC10}{VMAT},$$
$$C10HydroxyacylCoAMAT'[t] == \frac{vcrotC10 - vmschadC10}{VMAT},$$
$$C10KetoacylCoAMAT'[t] == \frac{vmschadC10 - vmckatC10}{VMAT},$$
$$C8AcylCarCYT'[t] == \frac{-vcactC8}{VCYT},$$
$$C8AcylCarMAT'[t] == \frac{vcactC8 - vcpt2C8}{VMAT},$$
$$C8AcylCoAMAT'[t] == \frac{vcpt2C8 + vmtpC10 + vmckatC10 - vlcadC8 - vmcadC8}{VMAT},$$
$$C8EnoylCoAMAT'[t] == \frac{vlcadC8 + vmcadC8 - vcrotC8 - vmtpC8}{VMAT},$$
$$C8HydroxyacylCoAMAT'[t] == \frac{vcrotC8 - vmschadC8}{VMAT},$$
$$C8KetoacylCoAMAT'[t] == \frac{vmschadC8 - vmckatC8}{VMAT},$$
$$C6AcylCarCYT'[t] == \frac{-vcactC6}{VCYT},$$
$$C6AcylCarMAT'[t] == \frac{vcactC6 - vcpt2C6}{VMAT},$$
$$C6AcylCoAMAT'[t] == \frac{vcpt2C6 + vmtpC8 + vmckatC8 - vmcadC6 - vscadC6}{VMAT},$$
$$C6EnoylCoAMAT'[t] == \frac{vmcadC6 + vscadC6 - vcrotC6}{VMAT},$$
$$C6HydroxyacylCoAMAT'[t] == \frac{vcrotC6 - vmschadC6}{VMAT},$$

```
C6KetoacylCoAMAT'[t] == (vmschadC6 - vmckatC6) / VMAT,
C4AcylCarCYT'[t] == (-vcactC4) / VCYT,
C4AcylCarMAT'[t] == (vcactC4 - vcpt2C4) / VMAT,
C4AcylCoAMAT'[t] == (vcpt2C4 + vmckatC6 - vmcadC4 - vscadC4) / VMAT,
C4EnoylCoAMAT'[t] == (vmcadC4 + vscadC4 - vcrotC4) / VMAT,
C4HydroxyacylCoAMAT'[t] == (vcrotC4 - vmschadC4) / VMAT,
C4AcetoacylCoAMAT'[t] == (vmschadC4 - vmckatC4) / VMAT,
AcetylCoAMAT'[t] ==
 1/VMAT (vmtpC16 + vmckatC16 + vmtpC14 + vmckatC14 + vmtpC12 + vmckatC12 + vmtpC10 +
    vmckatC10 + vmtpC8 + vmckatC8 + vmckatC6 + 2 * vmckatC4  - vacesink), (*-vacesink*)
FADHMAT'[t] == 1/VMAT (vvlcadC16 + vvlcadC14 + vvlcadC12 + vlcadC16 +
    vlcadC14 + vlcadC12 + vlcadC10 + vlcadC8 + vmcadC12 + vmcadC10 +
    vmcadC8 + vmcadC6 + vmcadC4 + vscadC6 + vscadC4 - vfadhsink),
NADHMAT'[t] == 1/VMAT (vmtpC16 + vmtpC14 + vmtpC12 + vmtpC10 + vmtpC8 +
    vmschadC16 + vmschadC14 + vmschadC12 + vmschadC10 +
    vmschadC8 + vmschadC6 + vmschadC4 + vpdhnad - voxphnadh)
};

RateEqs = {vcpt1C16 → CPT1[sfcpt1C16, Vcpt1, Kmcpt1C16AcylCoACYT,
     Kmcpt1CarCYT, Kmcpt1C16AcylCarCYT, Kmcpt1CoACYT, Kicpt1MalCoACYT,
     Keqcpt1, C16AcylCoACYT, CarCYT, C16AcylCarCYT[t], CoACYT, MalCoACYT,
     ncpt1, vmct1acet, kmct1acet, AcetateMAT, propacetcpt1, propacetmal, nc],
   vcactC16 → CACT[Vfcact, Vrcact, KmcactC16AcylCarCYT, KmcactCarMAT,
     KmcactC16AcylCarMAT, KmcactCarCYT, KicactC16AcylCarCYT,
     KicactCarCYT, Keqcact, C16AcylCarCYT[t], CarMAT, C16AcylCarMAT[t],
     CarCYT, vmct1acet, kmct1acet, AcetateMAT, propacetcact],
   vcactC14 → CACT[Vfcact, Vrcact, KmcactC14AcylCarCYT, KmcactCarMAT,
     KmcactC14AcylCarMAT, KmcactCarCYT, KicactC14AcylCarCYT, KicactCarCYT,
     Keqcact, C14AcylCarCYT[t], CarMAT, C14AcylCarMAT[t], CarCYT,
     vmct1acet, kmct1acet, AcetateMAT, propacetcact], vcactC12 →
    CACT[Vfcact, Vrcact, KmcactC12AcylCarCYT, KmcactCarMAT, KmcactC12AcylCarMAT,
     KmcactCarCYT, KicactC12AcylCarCYT, KicactCarCYT, Keqcact, C12AcylCarCYT[t],
     CarMAT, C12AcylCarMAT[t], CarCYT, vmct1acet, kmct1acet, AcetateMAT, propacetcact],
   vcactC10 → CACT[Vfcact, Vrcact, KmcactC10AcylCarCYT, KmcactCarMAT,
     KmcactC10AcylCarMAT, KmcactCarCYT, KicactC10AcylCarCYT,
     KicactCarCYT, Keqcact, C10AcylCarCYT[t], CarMAT, C10AcylCarMAT[t],
     CarCYT, vmct1acet, kmct1acet, AcetateMAT, propacetcact],
   vcactC8 → CACT[Vfcact, Vrcact, KmcactC8AcylCarCYT, KmcactCarMAT, KmcactC8AcylCarMAT,
     KmcactCarCYT, KicactC8AcylCarCYT, KicactCarCYT, Keqcact, C8AcylCarCYT[t],
     CarMAT, C8AcylCarMAT[t], CarCYT, vmct1acet, kmct1acet, AcetateMAT, propacetcact],
   vcactC6 → CACT[Vfcact, Vrcact, KmcactC6AcylCarCYT, KmcactCarMAT, KmcactC6AcylCarMAT,
```

```
    KmcactCarCYT, KicactC6AcylCarCYT, KicactCarCYT, Keqcact, C6AcylCarCYT[t],
    CarMAT, C6AcylCarMAT[t], CarCYT, vmct1acet, kmct1acet, AcetateMAT, propacetcact],
  vcactC4 → CACT[Vfcact, Vrcact, KmcactC4AcylCarCYT, KmcactCarMAT, KmcactC4AcylCarMAT,
    KmcactCarCYT, KicactC4AcylCarCYT, KicactCarCYT, Keqcact, C4AcylCarCYT[t],
    CarMAT, C4AcylCarMAT[t], CarCYT, vmct1acet, kmct1acet, AcetateMAT, propacetcact],
  vcpt2C16 → CPT2[sfcpt2C16, Vcpt2, Kmcpt2C16AcylCarMAT, Kmcpt2C14AcylCarMAT,
    Kmcpt2C12AcylCarMAT, Kmcpt2C10AcylCarMAT, Kmcpt2C8AcylCarMAT, Kmcpt2C6AcylCarMAT,
    Kmcpt2C4AcylCarMAT, Kmcpt2CoAMAT, Kmcpt2C16AcylCoAMAT, Kmcpt2C14AcylCoAMAT,
    Kmcpt2C12AcylCoAMAT, Kmcpt2C10AcylCoAMAT, Kmcpt2C8AcylCoAMAT,
    Kmcpt2C6AcylCoAMAT, Kmcpt2C4AcylCoAMAT, Kmcpt2CarMAT, Keqcpt2, C16AcylCarMAT[t],
    C14AcylCarMAT[t], C12AcylCarMAT[t], C10AcylCarMAT[t], C8AcylCarMAT[t],
    C6AcylCarMAT[t], C4AcylCarMAT[t], CoAMAT, C16AcylCoAMAT[t], C14AcylCoAMAT[t],
    C12AcylCoAMAT[t], C10AcylCoAMAT[t], C8AcylCoAMAT[t], C6AcylCoAMAT[t],
    C4AcylCoAMAT[t], CarMAT, vmct1acet, kmct1acet, AcetateMAT, propacetcpt2],
  vcpt2C14 → CPT2[sfcpt2C14, Vcpt2, Kmcpt2C14AcylCarMAT, Kmcpt2C16AcylCarMAT,
    Kmcpt2C12AcylCarMAT, Kmcpt2C10AcylCarMAT, Kmcpt2C8AcylCarMAT, Kmcpt2C6AcylCarMAT,
    Kmcpt2C4AcylCoAMAT, Kmcpt2CoAMAT, Kmcpt2C14AcylCoAMAT, Kmcpt2C16AcylCoAMAT,
    Kmcpt2C12AcylCoAMAT, Kmcpt2C10AcylCoAMAT, Kmcpt2C8AcylCoAMAT,
    Kmcpt2C6AcylCoAMAT, Kmcpt2C4AcylCoAMAT, Kmcpt2CarMAT, Keqcpt2, C14AcylCarMAT[t],
    C16AcylCarMAT[t], C12AcylCarMAT[t], C10AcylCarMAT[t], C8AcylCarMAT[t],
    C6AcylCarMAT[t], C4AcylCarMAT[t], CoAMAT, C14AcylCoAMAT[t], C16AcylCoAMAT[t],
    C12AcylCoAMAT[t], C10AcylCoAMAT[t], C8AcylCoAMAT[t], C6AcylCoAMAT[t],
    C4AcylCoAMAT[t], CarMAT, vmct1acet, kmct1acet, AcetateMAT, propacetcpt2],
  vcpt2C12 → CPT2[sfcpt2C12, Vcpt2, Kmcpt2C12AcylCarMAT, Kmcpt2C16AcylCarMAT,
    Kmcpt2C14AcylCarMAT, Kmcpt2C10AcylCarMAT, Kmcpt2C8AcylCarMAT, Kmcpt2C6AcylCarMAT,
    Kmcpt2C4AcylCarMAT, Kmcpt2CoAMAT, Kmcpt2C12AcylCoAMAT, Kmcpt2C16AcylCoAMAT,
    Kmcpt2C14AcylCoAMAT, Kmcpt2C10AcylCoAMAT, Kmcpt2C8AcylCoAMAT,
    Kmcpt2C6AcylCoAMAT, Kmcpt2C4AcylCoAMAT, Kmcpt2CarMAT, Keqcpt2, C12AcylCarMAT[t],
    C16AcylCarMAT[t], C14AcylCarMAT[t], C10AcylCarMAT[t], C8AcylCarMAT[t],
    C6AcylCarMAT[t], C4AcylCarMAT[t], CoAMAT, C12AcylCoAMAT[t], C16AcylCoAMAT[t],
    C14AcylCoAMAT[t], C10AcylCoAMAT[t], C8AcylCoAMAT[t], C6AcylCoAMAT[t],
    C4AcylCoAMAT[t], CarMAT, vmct1acet, kmct1acet, AcetateMAT, propacetcpt2],
  vcpt2C10 → CPT2[sfcpt2C10, Vcpt2, Kmcpt2C10AcylCarMAT, Kmcpt2C16AcylCarMAT,
    Kmcpt2C14AcylCarMAT, Kmcpt2C12AcylCarMAT, Kmcpt2C8AcylCarMAT, Kmcpt2C6AcylCarMAT,
    Kmcpt2C4AcylCarMAT, Kmcpt2CoAMAT, Kmcpt2C10AcylCoAMAT, Kmcpt2C16AcylCoAMAT,
    Kmcpt2C14AcylCoAMAT, Kmcpt2C12AcylCoAMAT, Kmcpt2C8AcylCoAMAT,
    Kmcpt2C6AcylCoAMAT, Kmcpt2C4AcylCoAMAT, Kmcpt2CarMAT, Keqcpt2, C10AcylCarMAT[t],
    C16AcylCarMAT[t], C14AcylCarMAT[t], C12AcylCarMAT[t], C8AcylCarMAT[t],
    C6AcylCarMAT[t], C4AcylCarMAT[t], CoAMAT, C10AcylCoAMAT[t], C16AcylCoAMAT[t],
    C14AcylCoAMAT[t], C12AcylCoAMAT[t], C8AcylCoAMAT[t], C6AcylCoAMAT[t],
    C4AcylCoAMAT[t], CarMAT, vmct1acet, kmct1acet, AcetateMAT, propacetcpt2],
  vcpt2C8 → CPT2[sfcpt2C8, Vcpt2, Kmcpt2C8AcylCarMAT, Kmcpt2C16AcylCarMAT,
    Kmcpt2C14AcylCarMAT, Kmcpt2C12AcylCarMAT, Kmcpt2C10AcylCarMAT, Kmcpt2C6AcylCarMAT,
    Kmcpt2C4AcylCarMAT, Kmcpt2CoAMAT, Kmcpt2C8AcylCoAMAT, Kmcpt2C16AcylCoAMAT,
    Kmcpt2C14AcylCoAMAT, Kmcpt2C12AcylCoAMAT, Kmcpt2C10AcylCoAMAT,
    Kmcpt2C6AcylCoAMAT, Kmcpt2C4AcylCoAMAT, Kmcpt2CarMAT, Keqcpt2, C8AcylCarMAT[t],
    C16AcylCarMAT[t], C14AcylCarMAT[t], C12AcylCarMAT[t], C10AcylCarMAT[t],
    C6AcylCarMAT[t], C4AcylCarMAT[t], CoAMAT, C8AcylCoAMAT[t], C16AcylCoAMAT[t],
    C14AcylCoAMAT[t], C12AcylCoAMAT[t], C10AcylCoAMAT[t], C6AcylCoAMAT[t],
    C4AcylCoAMAT[t], CarMAT, vmct1acet, kmct1acet, AcetateMAT, propacetcpt2],
  vcpt2C6 → CPT2[sfcpt2C6, Vcpt2, Kmcpt2C6AcylCarMAT, Kmcpt2C16AcylCarMAT,
    Kmcpt2C14AcylCarMAT, Kmcpt2C12AcylCarMAT, Kmcpt2C10AcylCarMAT, Kmcpt2C8AcylCarMAT,
    Kmcpt2C4AcylCarMAT, Kmcpt2CoAMAT, Kmcpt2C6AcylCoAMAT, Kmcpt2C16AcylCoAMAT,
    Kmcpt2C14AcylCoAMAT, Kmcpt2C12AcylCoAMAT, Kmcpt2C10AcylCoAMAT,
    Kmcpt2C8AcylCoAMAT, Kmcpt2C4AcylCoAMAT, Kmcpt2CarMAT, Keqcpt2, C6AcylCarMAT[t],
```

```
   C16AcylCarMAT[t], C14AcylCarMAT[t], C12AcylCarMAT[t], C10AcylCarMAT[t],
   C8AcylCarMAT[t], C4AcylCarMAT[t], CoAMAT, C6AcylCoAMAT[t], C16AcylCoAMAT[t],
   C14AcylCoAMAT[t], C12AcylCoAMAT[t], C10AcylCoAMAT[t], C8AcylCoAMAT[t],
   C4AcylCoAMAT[t], CarMAT, vmct1acet, kmct1acet, AcetateMAT, propacetcpt2],
vcpt2C4 → CPT2[sfcpt2C4, Vcpt2, Kmcpt2C4AcylCarMAT, Kmcpt2C16AcylCarMAT,
   Kmcpt2C14AcylCarMAT, Kmcpt2C12AcylCarMAT, Kmcpt2C10AcylCarMAT, Kmcpt2C8AcylCarMAT,
   Kmcpt2C6AcylCarMAT, Kmcpt2CoAMAT, Kmcpt2C4AcylCoAMAT, Kmcpt2C16AcylCoAMAT,
   Kmcpt2C14AcylCoAMAT, Kmcpt2C12AcylCoAMAT, Kmcpt2C10AcylCoAMAT,
   Kmcpt2C8AcylCoAMAT, Kmcpt2C6AcylCoAMAT, Kmcpt2CarMAT, Keqcpt2, C4AcylCarMAT[t],
   C16AcylCarMAT[t], C14AcylCarMAT[t], C12AcylCarMAT[t], C10AcylCarMAT[t],
   C8AcylCarMAT[t], C6AcylCarMAT[t], CoAMAT, C4AcylCoAMAT[t], C16AcylCoAMAT[t],
   C14AcylCoAMAT[t], C12AcylCoAMAT[t], C10AcylCoAMAT[t], C8AcylCoAMAT[t],
   C6AcylCoAMAT[t], CarMAT, vmct1acet, kmct1acet, AcetateMAT, propacetcpt2],
vvlcadC16 → VLCAD[sfvlcadC16, Vvlcad, KmvlcadC16AcylCoAMAT, KmvlcadC14AcylCoAMAT,
   KmvlcadC12AcylCoAMAT, KmvlcadFAD, KmvlcadC16EnoylCoAMAT,
   KmvlcadC14EnoylCoAMAT, KmvlcadC12EnoylCoAMAT, KmvlcadFADH, Keqvlcad,
   C16AcylCoAMAT[t], C14AcylCoAMAT[t], C12AcylCoAMAT[t], FADtMAT,
   C16EnoylCoAMAT[t], C14EnoylCoAMAT[t], C12EnoylCoAMAT[t], FADHMAT[t]],
vvlcadC14 → VLCAD[sfvlcadC14, Vvlcad, KmvlcadC14AcylCoAMAT, KmvlcadC16AcylCoAMAT,
   KmvlcadC12AcylCoAMAT, KmvlcadFAD, KmvlcadC14EnoylCoAMAT,
   KmvlcadC16EnoylCoAMAT, KmvlcadC12EnoylCoAMAT, KmvlcadFADH, Keqvlcad,
   C14AcylCoAMAT[t], C16AcylCoAMAT[t], C12AcylCoAMAT[t], FADtMAT,
   C14EnoylCoAMAT[t], C16EnoylCoAMAT[t], C12EnoylCoAMAT[t], FADHMAT[t]],
vvlcadC12 → VLCAD[sfvlcadC12, Vvlcad, KmvlcadC12AcylCoAMAT, KmvlcadC16AcylCoAMAT,
   KmvlcadC14AcylCoAMAT, KmvlcadFAD, KmvlcadC12EnoylCoAMAT,
   KmvlcadC16EnoylCoAMAT, KmvlcadC14EnoylCoAMAT, KmvlcadFADH, Keqvlcad,
   C12AcylCoAMAT[t], C16AcylCoAMAT[t], C14AcylCoAMAT[t], FADtMAT,
   C12EnoylCoAMAT[t], C16EnoylCoAMAT[t], C14EnoylCoAMAT[t], FADHMAT[t]],
vlcadC16 → LCAD[sflcadC16, Vlcad, KmlcadC16AcylCoAMAT, KmlcadC14AcylCoAMAT,
   KmlcadC12AcylCoAMAT, KmlcadC10AcylCoAMAT, KmlcadC8AcylCoAMAT, KmlcadFAD,
   KmlcadC16EnoylCoAMAT, KmlcadC14EnoylCoAMAT, KmlcadC12EnoylCoAMAT,
   KmlcadC10EnoylCoAMAT, KmlcadC8EnoylCoAMAT, KmlcadFADH, Keqlcad,
   C16AcylCoAMAT[t], C14AcylCoAMAT[t], C12AcylCoAMAT[t], C10AcylCoAMAT[t],
   C8AcylCoAMAT[t], FADtMAT, C16EnoylCoAMAT[t], C14EnoylCoAMAT[t],
   C12EnoylCoAMAT[t], C10EnoylCoAMAT[t], C8EnoylCoAMAT[t], FADHMAT[t]],
vlcadC14 → LCAD[sflcadC14, Vlcad, KmlcadC14AcylCoAMAT, KmlcadC16AcylCoAMAT,
   KmlcadC12AcylCoAMAT, KmlcadC10AcylCoAMAT, KmlcadC8AcylCoAMAT, KmlcadFAD,
   KmlcadC14EnoylCoAMAT, KmlcadC16EnoylCoAMAT, KmlcadC12EnoylCoAMAT,
   KmlcadC10EnoylCoAMAT, KmlcadC8EnoylCoAMAT, KmlcadFADH, Keqlcad,
   C14AcylCoAMAT[t], C16AcylCoAMAT[t], C12AcylCoAMAT[t], C10AcylCoAMAT[t],
   C8AcylCoAMAT[t], FADtMAT, C14EnoylCoAMAT[t], C16EnoylCoAMAT[t],
   C12EnoylCoAMAT[t], C10EnoylCoAMAT[t], C8EnoylCoAMAT[t], FADHMAT[t]],
vlcadC12 → LCAD[sflcadC12, Vlcad, KmlcadC12AcylCoAMAT, KmlcadC16AcylCoAMAT,
   KmlcadC14AcylCoAMAT, KmlcadC10AcylCoAMAT, KmlcadC8AcylCoAMAT, KmlcadFAD,
   KmlcadC12EnoylCoAMAT, KmlcadC16EnoylCoAMAT, KmlcadC14EnoylCoAMAT,
   KmlcadC10EnoylCoAMAT, KmlcadC8EnoylCoAMAT, KmlcadFADH, Keqlcad,
   C12AcylCoAMAT[t], C16AcylCoAMAT[t], C14AcylCoAMAT[t], C10AcylCoAMAT[t],
   C8AcylCoAMAT[t], FADtMAT, C14EnoylCoAMAT[t], C16EnoylCoAMAT[t],
   C14EnoylCoAMAT[t], C10EnoylCoAMAT[t], C8EnoylCoAMAT[t], FADHMAT[t]],
vlcadC10 → LCAD[sflcadC10, Vlcad, KmlcadC10AcylCoAMAT, KmlcadC16AcylCoAMAT,
   KmlcadC14AcylCoAMAT, KmlcadC12AcylCoAMAT, KmlcadC8AcylCoAMAT, KmlcadFAD,
   KmlcadC10EnoylCoAMAT, KmlcadC16EnoylCoAMAT, KmlcadC14EnoylCoAMAT,
   KmlcadC12EnoylCoAMAT, KmlcadC8EnoylCoAMAT, KmlcadFADH, Keqlcad,
   C10AcylCoAMAT[t], C16AcylCoAMAT[t], C14AcylCoAMAT[t], C12AcylCoAMAT[t],
   C8AcylCoAMAT[t], FADtMAT, C10EnoylCoAMAT[t], C16EnoylCoAMAT[t],
```

```
    C14EnoylCoAMAT[t], C12EnoylCoAMAT[t], C8EnoylCoAMAT[t], FADHMAT[t]],
  vlcadC8 → LCAD[sflcadC8, Vlcad, KmlcadC8AcylCoAMAT, KmlcadC16AcylCoAMAT,
    KmlcadC14AcylCoAMAT, KmlcadC12AcylCoAMAT, KmlcadC10AcylCoAMAT, KmlcadFAD,
    KmlcadC8EnoylCoAMAT, KmlcadC16EnoylCoAMAT, KmlcadC14EnoylCoAMAT,
    KmlcadC12EnoylCoAMAT, KmlcadC10EnoylCoAMAT, KmlcadFADH, Keqlcad,
    C8AcylCoAMAT[t], C16AcylCoAMAT[t], C14AcylCoAMAT[t], C12AcylCoAMAT[t],
    C10AcylCoAMAT[t], FADtMAT, C8EnoylCoAMAT[t], C16EnoylCoAMAT[t],
    C14EnoylCoAMAT[t], C12EnoylCoAMAT[t], C10EnoylCoAMAT[t], FADHMAT[t]],
  vmcadC12 → MCAD[sfmcadC12, Vmcad, KmmcadC12AcylCoAMAT, KmmcadC10AcylCoAMAT,
    KmmcadC8AcylCoAMAT, KmmcadC6AcylCoAMAT, KmmcadC4AcylCoAMAT, KmmcadFAD,
    KmmcadC12EnoylCoAMAT, KmmcadC10EnoylCoAMAT, KmmcadC8EnoylCoAMAT,
    KmmcadC6EnoylCoAMAT, KmmcadC4EnoylCoAMAT, KmmcadFADH, Keqmcad,
    C12AcylCoAMAT[t], C10AcylCoAMAT[t], C8AcylCoAMAT[t], C6AcylCoAMAT[t],
    C4AcylCoAMAT[t], FADtMAT, C12EnoylCoAMAT[t], C10EnoylCoAMAT[t],
    C8EnoylCoAMAT[t], C6EnoylCoAMAT[t], C4EnoylCoAMAT[t], FADHMAT[t]],
  vmcadC10 → MCAD[sfmcadC10, Vmcad, KmmcadC10AcylCoAMAT, KmmcadC12AcylCoAMAT,
    KmmcadC8AcylCoAMAT, KmmcadC6AcylCoAMAT, KmmcadC4AcylCoAMAT, KmmcadFAD,
    KmmcadC10EnoylCoAMAT, KmmcadC12EnoylCoAMAT, KmmcadC8EnoylCoAMAT,
    KmmcadC6EnoylCoAMAT, KmmcadC4EnoylCoAMAT, KmmcadFADH, Keqmcad,
    C10AcylCoAMAT[t], C12AcylCoAMAT[t], C8AcylCoAMAT[t], C6AcylCoAMAT[t],
    C4AcylCoAMAT[t], FADtMAT, C10EnoylCoAMAT[t], C12EnoylCoAMAT[t],
    C8EnoylCoAMAT[t], C6EnoylCoAMAT[t], C4EnoylCoAMAT[t], FADHMAT[t]],
  vmcadC8 → MCAD[sfmcadC8, Vmcad, KmmcadC8AcylCoAMAT, KmmcadC12AcylCoAMAT,
    KmmcadC10AcylCoAMAT, KmmcadC6AcylCoAMAT, KmmcadC4AcylCoAMAT, KmmcadFAD,
    KmmcadC8EnoylCoAMAT, KmmcadC12EnoylCoAMAT, KmmcadC10EnoylCoAMAT,
    KmmcadC6EnoylCoAMAT, KmmcadC4EnoylCoAMAT, KmmcadFADH, Keqmcad,
    C8AcylCoAMAT[t], C12AcylCoAMAT[t], C10AcylCoAMAT[t], C6AcylCoAMAT[t],
    C4AcylCoAMAT[t], FADtMAT, C8EnoylCoAMAT[t], C12EnoylCoAMAT[t],
    C10EnoylCoAMAT[t], C6EnoylCoAMAT[t], C4EnoylCoAMAT[t], FADHMAT[t]],
  vmcadC6 → MCAD[sfmcadC6, Vmcad, KmmcadC6AcylCoAMAT, KmmcadC12AcylCoAMAT,
    KmmcadC10AcylCoAMAT, KmmcadC8AcylCoAMAT, KmmcadC4AcylCoAMAT, KmmcadFAD,
    KmmcadC6EnoylCoAMAT, KmmcadC12EnoylCoAMAT, KmmcadC10EnoylCoAMAT,
    KmmcadC8EnoylCoAMAT, KmmcadC4EnoylCoAMAT, KmmcadFADH, Keqmcad,
    C6AcylCoAMAT[t], C12AcylCoAMAT[t], C10AcylCoAMAT[t], C8AcylCoAMAT[t],
    C4AcylCoAMAT[t], FADtMAT, C6EnoylCoAMAT[t], C12EnoylCoAMAT[t],
    C10EnoylCoAMAT[t], C8EnoylCoAMAT[t], C4EnoylCoAMAT[t], FADHMAT[t]],
  vmcadC4 → MCAD[sfmcadC4, Vmcad, KmmcadC4AcylCoAMAT, KmmcadC12AcylCoAMAT,
    KmmcadC10AcylCoAMAT, KmmcadC8AcylCoAMAT, KmmcadC6AcylCoAMAT, KmmcadFAD,
    KmmcadC4EnoylCoAMAT, KmmcadC12EnoylCoAMAT, KmmcadC10EnoylCoAMAT,
    KmmcadC8EnoylCoAMAT, KmmcadC6EnoylCoAMAT, KmmcadFADH, Keqmcad,
    C4AcylCoAMAT[t], C12AcylCoAMAT[t], C10AcylCoAMAT[t], C8AcylCoAMAT[t],
    C6AcylCoAMAT[t], FADtMAT, C4EnoylCoAMAT[t], C12EnoylCoAMAT[t],
    C10EnoylCoAMAT[t], C8EnoylCoAMAT[t], C6EnoylCoAMAT[t], FADHMAT[t]],
  vscadC6 → SCAD[sfscadC6, Vscad, KmscadC6AcylCoAMAT, KmscadC4AcylCoAMAT, KmscadFAD,
    KmscadC6EnoylCoAMAT, KmscadC4EnoylCoAMAT, KmscadFADH, Keqscad, C6AcylCoAMAT[t],
    C4AcylCoAMAT[t], FADtMAT, C6EnoylCoAMAT[t], C4EnoylCoAMAT[t], FADHMAT[t]],
  vscadC4 → SCAD[sfscadC4, Vscad, KmscadC4AcylCoAMAT, KmscadC6AcylCoAMAT, KmscadFAD,
    KmscadC4EnoylCoAMAT, KmscadC6EnoylCoAMAT, KmscadFADH, Keqscad, C4AcylCoAMAT[t],
    C6AcylCoAMAT[t], FADtMAT, C4EnoylCoAMAT[t], C6EnoylCoAMAT[t], FADHMAT[t]],
  vcrotC16 → CROT[sfcrotC16, Vcrot, KmcrotC16EnoylCoAMAT, KmcrotC14EnoylCoAMAT,
    KmcrotC12EnoylCoAMAT, KmcrotC10EnoylCoAMAT, KmcrotC8EnoylCoAMAT,
    KmcrotC6EnoylCoAMAT, KmcrotC4EnoylCoAMAT, KmcrotC16HydroxyacylCoAMAT,
    KmcrotC14HydroxyacylCoAMAT, KmcrotC12HydroxyacylCoAMAT,
    KmcrotC10HydroxyacylCoAMAT, KmcrotC8HydroxyacylCoAMAT,
    KmcrotC6HydroxyacylCoAMAT, KmcrotC4HydroxyacylCoAMAT, KicrotC4AcetoacylCoA,
```

```
    Keqcrot, C16EnoylCoAMAT[t], C14EnoylCoAMAT[t], C12EnoylCoAMAT[t],
    C10EnoylCoAMAT[t], C8EnoylCoAMAT[t], C6EnoylCoAMAT[t],
    C4EnoylCoAMAT[t], C16HydroxyacylCoAMAT[t], C14HydroxyacylCoAMAT[t],
    C12HydroxyacylCoAMAT[t], C10HydroxyacylCoAMAT[t], C8HydroxyacylCoAMAT[t],
    C6HydroxyacylCoAMAT[t], C4HydroxyacylCoAMAT[t], C4AcetoacylCoAMAT[t]],
  vcrotC14 → CROT[sfcrotC14, Vcrot, KmcrotC14EnoylCoAMAT, KmcrotC16EnoylCoAMAT,
    KmcrotC12EnoylCoAMAT, KmcrotC10EnoylCoAMAT, KmcrotC8EnoylCoAMAT,
    KmcrotC6EnoylCoAMAT, KmcrotC4EnoylCoAMAT, KmcrotC14HydroxyacylCoAMAT,
    KmcrotC16HydroxyacylCoAMAT, KmcrotC12HydroxyacylCoAMAT,
    KmcrotC10HydroxyacylCoAMAT, KmcrotC8HydroxyacylCoAMAT,
    KmcrotC6HydroxyacylCoAMAT, KmcrotC4HydroxyacylCoAMAT, KicrotC4AcetoacylCoA,
    Keqcrot, C14EnoylCoAMAT[t], C16EnoylCoAMAT[t], C12EnoylCoAMAT[t],
    C10EnoylCoAMAT[t], C8EnoylCoAMAT[t], C6EnoylCoAMAT[t],
    C4EnoylCoAMAT[t], C14HydroxyacylCoAMAT[t], C16HydroxyacylCoAMAT[t],
    C12HydroxyacylCoAMAT[t], C10HydroxyacylCoAMAT[t], C8HydroxyacylCoAMAT[t],
    C6HydroxyacylCoAMAT[t], C4HydroxyacylCoAMAT[t], C4AcetoacylCoAMAT[t]],
  vcrotC12 → CROT[sfcrotC12, Vcrot, KmcrotC12EnoylCoAMAT, KmcrotC16EnoylCoAMAT,
    KmcrotC14EnoylCoAMAT, KmcrotC10EnoylCoAMAT, KmcrotC8EnoylCoAMAT,
    KmcrotC6EnoylCoAMAT, KmcrotC4EnoylCoAMAT, KmcrotC12HydroxyacylCoAMAT,
    KmcrotC16HydroxyacylCoAMAT, KmcrotC14HydroxyacylCoAMAT,
    KmcrotC10HydroxyacylCoAMAT, KmcrotC8HydroxyacylCoAMAT,
    KmcrotC6HydroxyacylCoAMAT, KmcrotC4HydroxyacylCoAMAT, KicrotC4AcetoacylCoA,
    Keqcrot, C12EnoylCoAMAT[t], C16EnoylCoAMAT[t], C14EnoylCoAMAT[t],
    C10EnoylCoAMAT[t], C8EnoylCoAMAT[t], C6EnoylCoAMAT[t],
    C4EnoylCoAMAT[t], C12HydroxyacylCoAMAT[t], C16HydroxyacylCoAMAT[t],
    C14HydroxyacylCoAMAT[t], C10HydroxyacylCoAMAT[t], C8HydroxyacylCoAMAT[t],
    C6HydroxyacylCoAMAT[t], C4HydroxyacylCoAMAT[t], C4AcetoacylCoAMAT[t]],
  vcrotC10 → CROT[sfcrotC10, Vcrot, KmcrotC10EnoylCoAMAT, KmcrotC16EnoylCoAMAT,
    KmcrotC14EnoylCoAMAT, KmcrotC12EnoylCoAMAT, KmcrotC8EnoylCoAMAT,
    KmcrotC6EnoylCoAMAT, KmcrotC4EnoylCoAMAT, KmcrotC10HydroxyacylCoAMAT,
    KmcrotC16HydroxyacylCoAMAT, KmcrotC14HydroxyacylCoAMAT,
    KmcrotC12HydroxyacylCoAMAT, KmcrotC8HydroxyacylCoAMAT,
    KmcrotC6HydroxyacylCoAMAT, KmcrotC4HydroxyacylCoAMAT, KicrotC4AcetoacylCoA,
    Keqcrot, C10EnoylCoAMAT[t], C16EnoylCoAMAT[t], C14EnoylCoAMAT[t],
    C12EnoylCoAMAT[t], C8EnoylCoAMAT[t], C6EnoylCoAMAT[t],
    C4EnoylCoAMAT[t], C10HydroxyacylCoAMAT[t], C16HydroxyacylCoAMAT[t],
    C14HydroxyacylCoAMAT[t], C12HydroxyacylCoAMAT[t], C8HydroxyacylCoAMAT[t],
    C6HydroxyacylCoAMAT[t], C4HydroxyacylCoAMAT[t], C4AcetoacylCoAMAT[t]],
  vcrotC8 → CROT[sfcrotC8, Vcrot, KmcrotC8EnoylCoAMAT, KmcrotC16EnoylCoAMAT,
    KmcrotC14EnoylCoAMAT, KmcrotC12EnoylCoAMAT, KmcrotC10EnoylCoAMAT,
    KmcrotC6EnoylCoAMAT, KmcrotC4EnoylCoAMAT, KmcrotC8HydroxyacylCoAMAT,
    KmcrotC16HydroxyacylCoAMAT, KmcrotC14HydroxyacylCoAMAT,
    KmcrotC12HydroxyacylCoAMAT, KmcrotC10HydroxyacylCoAMAT,
    KmcrotC6HydroxyacylCoAMAT, KmcrotC4HydroxyacylCoAMAT, KicrotC4AcetoacylCoA,
    Keqcrot, C8EnoylCoAMAT[t], C16EnoylCoAMAT[t], C14EnoylCoAMAT[t],
    C12EnoylCoAMAT[t], C10EnoylCoAMAT[t], C6EnoylCoAMAT[t],
    C4EnoylCoAMAT[t], C8HydroxyacylCoAMAT[t], C16HydroxyacylCoAMAT[t],
    C14HydroxyacylCoAMAT[t], C12HydroxyacylCoAMAT[t], C10HydroxyacylCoAMAT[t],
    C6HydroxyacylCoAMAT[t], C4HydroxyacylCoAMAT[t], C4AcetoacylCoAMAT[t]],
  vcrotC6 → CROT[sfcrotC6, Vcrot, KmcrotC6EnoylCoAMAT, KmcrotC16EnoylCoAMAT,
    KmcrotC14EnoylCoAMAT, KmcrotC12EnoylCoAMAT, KmcrotC10EnoylCoAMAT,
    KmcrotC8EnoylCoAMAT, KmcrotC4EnoylCoAMAT, KmcrotC6HydroxyacylCoAMAT,
    KmcrotC16HydroxyacylCoAMAT, KmcrotC14HydroxyacylCoAMAT,
    KmcrotC12HydroxyacylCoAMAT, KmcrotC10HydroxyacylCoAMAT,
    KmcrotC8HydroxyacylCoAMAT, KmcrotC4HydroxyacylCoAMAT, KicrotC4AcetoacylCoA,
```

```
    Keqcrot, C6EnoylCoAMAT[t], C16EnoylCoAMAT[t], C14EnoylCoAMAT[t],
    C12EnoylCoAMAT[t], C10EnoylCoAMAT[t], C8EnoylCoAMAT[t],
    C4EnoylCoAMAT[t], C6HydroxyacylCoAMAT[t], C16HydroxyacylCoAMAT[t],
    C14HydroxyacylCoAMAT[t], C12HydroxyacylCoAMAT[t], C10HydroxyacylCoAMAT[t],
    C8HydroxyacylCoAMAT[t], C4HydroxyacylCoAMAT[t], C4AcetoacylCoAMAT[t]],
  vcrotC4 → CROT[sfcrotC4, Vcrot, KmcrotC4EnoylCoAMAT, KmcrotC16EnoylCoAMAT,
    KmcrotC14EnoylCoAMAT, KmcrotC12EnoylCoAMAT, KmcrotC10EnoylCoAMAT,
    KmcrotC8EnoylCoAMAT, KmcrotC6EnoylCoAMAT, KmcrotC4HydroxyacylCoAMAT,
    KmcrotC16HydroxyacylCoAMAT, KmcrotC14HydroxyacylCoAMAT,
    KmcrotC12HydroxyacylCoAMAT, KmcrotC10HydroxyacylCoAMAT,
    KmcrotC8HydroxyacylCoAMAT, KmcrotC6HydroxyacylCoAMAT, KicrotC4AcetoacylCoA,
    Keqcrot, C4EnoylCoAMAT[t], C16EnoylCoAMAT[t], C14EnoylCoAMAT[t],
    C12EnoylCoAMAT[t], C10EnoylCoAMAT[t], C8EnoylCoAMAT[t],
    C6EnoylCoAMAT[t], C4HydroxyacylCoAMAT[t], C16HydroxyacylCoAMAT[t],
    C14HydroxyacylCoAMAT[t], C12HydroxyacylCoAMAT[t], C10HydroxyacylCoAMAT[t],
    C8HydroxyacylCoAMAT[t], C6HydroxyacylCoAMAT[t], C4AcetoacylCoAMAT[t]],
  vmschadC16 → MSCHAD[sfmschadC16, Vmschad, KmmschadC16HydroxyacylCoAMAT,
    KmmschadC14HydroxyacylCoAMAT, KmmschadC12HydroxyacylCoAMAT,
    KmmschadC10HydroxyacylCoAMAT, KmmschadC8HydroxyacylCoAMAT,
    KmmschadC6HydroxyacylCoAMAT, KmmschadC4HydroxyacylCoAMAT,
    KmmschadNADMAT, KmmschadC16KetoacylCoAMAT, KmmschadC14KetoacylCoAMAT,
    KmmschadC12KetoacylCoAMAT, KmmschadC10KetoacylCoAMAT, KmmschadC8KetoacylCoAMAT,
    KmmschadC6KetoacylCoAMAT, KmmschadC4AcetoacylCoAMAT, KmmschadNADHMAT,
    Keqmschad, C16HydroxyacylCoAMAT[t], C14HydroxyacylCoAMAT[t],
    C12HydroxyacylCoAMAT[t], C10HydroxyacylCoAMAT[t], C8HydroxyacylCoAMAT[t],
    C6HydroxyacylCoAMAT[t], C4HydroxyacylCoAMAT[t], NADtMAT, C16KetoacylCoAMAT[t],
    C14KetoacylCoAMAT[t], C12KetoacylCoAMAT[t], C10KetoacylCoAMAT[t],
    C8KetoacylCoAMAT[t], C6KetoacylCoAMAT[t], C4AcetoacylCoAMAT[t], NADHMAT[t]],
  vmschadC14 → MSCHAD[sfmschadC14, Vmschad, KmmschadC14HydroxyacylCoAMAT,
    KmmschadC16HydroxyacylCoAMAT, KmmschadC12HydroxyacylCoAMAT,
    KmmschadC10HydroxyacylCoAMAT, KmmschadC8HydroxyacylCoAMAT,
    KmmschadC6HydroxyacylCoAMAT, KmmschadC4HydroxyacylCoAMAT,
    KmmschadNADMAT, KmmschadC14KetoacylCoAMAT, KmmschadC16KetoacylCoAMAT,
    KmmschadC12KetoacylCoAMAT, KmmschadC10KetoacylCoAMAT, KmmschadC8KetoacylCoAMAT,
    KmmschadC6KetoacylCoAMAT, KmmschadC4AcetoacylCoAMAT, KmmschadNADHMAT,
    Keqmschad, C14HydroxyacylCoAMAT[t], C16HydroxyacylCoAMAT[t],
    C12HydroxyacylCoAMAT[t], C10HydroxyacylCoAMAT[t], C8HydroxyacylCoAMAT[t],
    C6HydroxyacylCoAMAT[t], C4HydroxyacylCoAMAT[t], NADtMAT, C14KetoacylCoAMAT[t],
    C16KetoacylCoAMAT[t], C12KetoacylCoAMAT[t], C10KetoacylCoAMAT[t],
    C8KetoacylCoAMAT[t], C6KetoacylCoAMAT[t], C4AcetoacylCoAMAT[t], NADHMAT[t]],
  vmschadC12 → MSCHAD[sfmschadC12, Vmschad, KmmschadC12HydroxyacylCoAMAT,
    KmmschadC16HydroxyacylCoAMAT, KmmschadC14HydroxyacylCoAMAT,
    KmmschadC10HydroxyacylCoAMAT, KmmschadC8HydroxyacylCoAMAT,
    KmmschadC6HydroxyacylCoAMAT, KmmschadC4HydroxyacylCoAMAT,
    KmmschadNADMAT, KmmschadC12KetoacylCoAMAT, KmmschadC16KetoacylCoAMAT,
    KmmschadC14KetoacylCoAMAT, KmmschadC10KetoacylCoAMAT, KmmschadC8KetoacylCoAMAT,
    KmmschadC6KetoacylCoAMAT, KmmschadC4AcetoacylCoAMAT, KmmschadNADHMAT,
    Keqmschad, C12HydroxyacylCoAMAT[t], C16HydroxyacylCoAMAT[t],
    C14HydroxyacylCoAMAT[t], C10HydroxyacylCoAMAT[t], C8HydroxyacylCoAMAT[t],
    C6HydroxyacylCoAMAT[t], C4HydroxyacylCoAMAT[t], NADtMAT, C12KetoacylCoAMAT[t],
    C16KetoacylCoAMAT[t], C14KetoacylCoAMAT[t], C10KetoacylCoAMAT[t],
    C8KetoacylCoAMAT[t], C6KetoacylCoAMAT[t], C4AcetoacylCoAMAT[t], NADHMAT[t]],
  vmschadC10 → MSCHAD[sfmschadC10, Vmschad, KmmschadC10HydroxyacylCoAMAT,
    KmmschadC16HydroxyacylCoAMAT, KmmschadC14HydroxyacylCoAMAT,
    KmmschadC12HydroxyacylCoAMAT, KmmschadC8HydroxyacylCoAMAT,
```

```
    KmmschadC6HydroxyacylCoAMAT, KmmschadC4HydroxyacylCoAMAT,
    KmmschadNADMAT, KmmschadC10KetoacylCoAMAT, KmmschadC16KetoacylCoAMAT,
    KmmschadC14KetoacylCoAMAT, KmmschadC12KetoacylCoAMAT, KmmschadC8KetoacylCoAMAT,
    KmmschadC6KetoacylCoAMAT, KmmschadC4AcetoacylCoAMAT, KmmschadNADHMAT,
    Keqmschad, C10HydroxyacylCoAMAT[t], C16HydroxyacylCoAMAT[t],
    C14HydroxyacylCoAMAT[t], C12HydroxyacylCoAMAT[t], C8HydroxyacylCoAMAT[t],
    C6HydroxyacylCoAMAT[t], C4HydroxyacylCoAMAT[t], NADtMAT, C10KetoacylCoAMAT[t],
    C16KetoacylCoAMAT[t], C14KetoacylCoAMAT[t], C12KetoacylCoAMAT[t],
    C8KetoacylCoAMAT[t], C6KetoacylCoAMAT[t], C4AcetoacylCoAMAT[t], NADHMAT[t]],
vmschadC8 → MSCHAD[sfmschadC8, Vmschad, KmmschadC8HydroxyacylCoAMAT,
    KmmschadC16HydroxyacylCoAMAT, KmmschadC14HydroxyacylCoAMAT,
    KmmschadC12HydroxyacylCoAMAT, KmmschadC10HydroxyacylCoAMAT,
    KmmschadC6HydroxyacylCoAMAT, KmmschadC4HydroxyacylCoAMAT,
    KmmschadNADMAT, KmmschadC8KetoacylCoAMAT, KmmschadC16KetoacylCoAMAT,
    KmmschadC14KetoacylCoAMAT, KmmschadC12KetoacylCoAMAT, KmmschadC10KetoacylCoAMAT,
    KmmschadC6KetoacylCoAMAT, KmmschadC4AcetoacylCoAMAT, KmmschadNADHMAT,
    Keqmschad, C8HydroxyacylCoAMAT[t], C16HydroxyacylCoAMAT[t],
    C14HydroxyacylCoAMAT[t], C12HydroxyacylCoAMAT[t], C10HydroxyacylCoAMAT[t],
    C6HydroxyacylCoAMAT[t], C4HydroxyacylCoAMAT[t], NADtMAT, C8KetoacylCoAMAT[t],
    C16KetoacylCoAMAT[t], C14KetoacylCoAMAT[t], C12KetoacylCoAMAT[t],
    C10KetoacylCoAMAT[t], C6KetoacylCoAMAT[t], C4AcetoacylCoAMAT[t], NADHMAT[t]],
vmschadC6 → MSCHAD[sfmschadC6, Vmschad, KmmschadC6HydroxyacylCoAMAT,
    KmmschadC16HydroxyacylCoAMAT, KmmschadC14HydroxyacylCoAMAT,
    KmmschadC12HydroxyacylCoAMAT, KmmschadC10HydroxyacylCoAMAT,
    KmmschadC8HydroxyacylCoAMAT, KmmschadC4HydroxyacylCoAMAT,
    KmmschadNADMAT, KmmschadC6KetoacylCoAMAT, KmmschadC16KetoacylCoAMAT,
    KmmschadC14KetoacylCoAMAT, KmmschadC12KetoacylCoAMAT, KmmschadC10KetoacylCoAMAT,
    KmmschadC8KetoacylCoAMAT, KmmschadC4AcetoacylCoAMAT, KmmschadNADHMAT,
    Keqmschad, C6HydroxyacylCoAMAT[t], C16HydroxyacylCoAMAT[t],
    C14HydroxyacylCoAMAT[t], C12HydroxyacylCoAMAT[t], C10HydroxyacylCoAMAT[t],
    C8HydroxyacylCoAMAT[t], C4HydroxyacylCoAMAT[t], NADtMAT, C6KetoacylCoAMAT[t],
    C16KetoacylCoAMAT[t], C14KetoacylCoAMAT[t], C12KetoacylCoAMAT[t],
    C10KetoacylCoAMAT[t], C8KetoacylCoAMAT[t], C4AcetoacylCoAMAT[t], NADHMAT[t]],
vmschadC4 → MSCHAD[sfmschadC4, Vmschad, KmmschadC4HydroxyacylCoAMAT,
    KmmschadC16HydroxyacylCoAMAT, KmmschadC14HydroxyacylCoAMAT,
    KmmschadC12HydroxyacylCoAMAT, KmmschadC10HydroxyacylCoAMAT,
    KmmschadC8HydroxyacylCoAMAT, KmmschadC6HydroxyacylCoAMAT,
    KmmschadNADMAT, KmmschadC4AcetoacylCoAMAT, KmmschadC16KetoacylCoAMAT,
    KmmschadC14KetoacylCoAMAT, KmmschadC12KetoacylCoAMAT, KmmschadC10KetoacylCoAMAT,
    KmmschadC8KetoacylCoAMAT, KmmschadC6KetoacylCoAMAT, KmmschadNADHMAT,
    Keqmschad, C4HydroxyacylCoAMAT[t], C16HydroxyacylCoAMAT[t],
    C14HydroxyacylCoAMAT[t], C12HydroxyacylCoAMAT[t], C10HydroxyacylCoAMAT[t],
    C8HydroxyacylCoAMAT[t], C6HydroxyacylCoAMAT[t], NADtMAT, C4AcetoacylCoAMAT[t],
    C16KetoacylCoAMAT[t], C14KetoacylCoAMAT[t], C12KetoacylCoAMAT[t],
    C10KetoacylCoAMAT[t], C8KetoacylCoAMAT[t], C6KetoacylCoAMAT[t], NADHMAT[t]],
vmckatC16 → MCKATA[sfmckatC16, Vmckat, KmmckatC16KetoacylCoAMAT,
    KmmckatC14KetoacylCoAMAT, KmmckatC12KetoacylCoAMAT, KmmckatC10KetoacylCoAMAT,
    KmmckatC8KetoacylCoAMAT, KmmckatC6KetoacylCoAMAT, KmmckatC4AcetoacylCoAMAT,
    KmmckatCoAMAT, KmmckatC14AcylCoAMAT, KmmckatC16AcylCoAMAT, KmmckatC12AcylCoAMAT,
    KmmckatC10AcylCoAMAT, KmmckatC8AcylCoAMAT, KmmckatC6AcylCoAMAT,
    KmmckatC4AcylCoAMAT, KmmckatAcetylCoAMAT, Keqmckat, C16KetoacylCoAMAT[t],
    C14KetoacylCoAMAT[t], C12KetoacylCoAMAT[t], C10KetoacylCoAMAT[t],
    C8KetoacylCoAMAT[t], C6KetoacylCoAMAT[t], C4AcetoacylCoAMAT[t], CoAMAT,
    C14AcylCoAMAT[t], C16AcylCoAMAT[t], C12AcylCoAMAT[t], C10AcylCoAMAT[t],
    C8AcylCoAMAT[t], C6AcylCoAMAT[t], C4AcylCoAMAT[t], AcetylCoAMAT[t],
```

```
    CE1, KmCE1, nE1, vmct1acet, kmct1acet, AcetateMAT, propacetmckat],
  vmckatC14 → MCKATA[sfmckatC14, Vmckat, KmmckatC14KetoacylCoAMAT,
    KmmckatC16KetoacylCoAMAT, KmmckatC12KetoacylCoAMAT, KmmckatC10KetoacylCoAMAT,
    KmmckatC8KetoacylCoAMAT, KmmckatC6KetoacylCoAMAT, KmmckatC4AcetoacylCoAMAT,
    KmmckatCoAMAT, KmmckatC12AcylCoAMAT, KmmckatC16AcylCoAMAT, KmmckatC14AcylCoAMAT,
    KmmckatC10AcylCoAMAT, KmmckatC8AcylCoAMAT, KmmckatC6AcylCoAMAT,
    KmmckatC4AcylCoAMAT, KmmckatAcetylCoAMAT, Keqmckat, C14KetoacylCoAMAT[t],
    C16KetoacylCoAMAT[t], C12KetoacylCoAMAT[t], C10KetoacylCoAMAT[t],
    C8KetoacylCoAMAT[t], C6KetoacylCoAMAT[t], C4AcetoacylCoAMAT[t], CoAMAT,
    C12AcylCoAMAT[t], C16AcylCoAMAT[t], C14AcylCoAMAT[t], C10AcylCoAMAT[t],
    C8AcylCoAMAT[t], C6AcylCoAMAT[t], C4AcylCoAMAT[t], AcetylCoAMAT[t],
    CE1, KmCE1, nE1, vmct1acet, kmct1acet, AcetateMAT, propacetmckat],
  vmckatC12 → MCKATA[sfmckatC12, Vmckat, KmmckatC12KetoacylCoAMAT,
    KmmckatC16KetoacylCoAMAT, KmmckatC14KetoacylCoAMAT, KmmckatC10KetoacylCoAMAT,
    KmmckatC8KetoacylCoAMAT, KmmckatC6KetoacylCoAMAT, KmmckatC4AcetoacylCoAMAT,
    KmmckatCoAMAT, KmmckatC10AcylCoAMAT, KmmckatC16AcylCoAMAT, KmmckatC14AcylCoAMAT,
    KmmckatC12AcylCoAMAT, KmmckatC8AcylCoAMAT, KmmckatC6AcylCoAMAT,
    KmmckatC4AcylCoAMAT, KmmckatAcetylCoAMAT, Keqmckat, C12KetoacylCoAMAT[t],
    C16KetoacylCoAMAT[t], C14KetoacylCoAMAT[t], C10KetoacylCoAMAT[t],
    C8KetoacylCoAMAT[t], C6KetoacylCoAMAT[t], C4AcetoacylCoAMAT[t], CoAMAT,
    C10AcylCoAMAT[t], C16AcylCoAMAT[t], C14AcylCoAMAT[t], C12AcylCoAMAT[t],
    C8AcylCoAMAT[t], C6AcylCoAMAT[t], C4AcylCoAMAT[t], AcetylCoAMAT[t],
    CE1, KmCE1, nE1, vmct1acet, kmct1acet, AcetateMAT, propacetmckat],
  vmckatC10 → MCKATA[sfmckatC10, Vmckat, KmmckatC10KetoacylCoAMAT,
    KmmckatC16KetoacylCoAMAT, KmmckatC14KetoacylCoAMAT, KmmckatC12KetoacylCoAMAT,
    KmmckatC8KetoacylCoAMAT, KmmckatC6KetoacylCoAMAT, KmmckatC4AcetoacylCoAMAT,
    KmmckatCoAMAT, KmmckatC8AcylCoAMAT, KmmckatC16AcylCoAMAT, KmmckatC14AcylCoAMAT,
    KmmckatC12AcylCoAMAT, KmmckatC10AcylCoAMAT, KmmckatC6AcylCoAMAT,
    KmmckatC4AcylCoAMAT, KmmckatAcetylCoAMAT, Keqmckat, C10KetoacylCoAMAT[t],
    C16KetoacylCoAMAT[t], C14KetoacylCoAMAT[t], C12KetoacylCoAMAT[t],
    C8KetoacylCoAMAT[t], C6KetoacylCoAMAT[t], C4AcetoacylCoAMAT[t], CoAMAT,
    C8AcylCoAMAT[t], C16AcylCoAMAT[t], C14AcylCoAMAT[t], C12AcylCoAMAT[t],
    C10AcylCoAMAT[t], C6AcylCoAMAT[t], C4AcylCoAMAT[t], AcetylCoAMAT[t],
    CE1, KmCE1, nE1, vmct1acet, kmct1acet, AcetateMAT, propacetmckat],
  vmckatC8 → MCKATA[sfmckatC8, Vmckat, KmmckatC8KetoacylCoAMAT,
    KmmckatC16KetoacylCoAMAT, KmmckatC14KetoacylCoAMAT, KmmckatC12KetoacylCoAMAT,
    KmmckatC10KetoacylCoAMAT, KmmckatC6KetoacylCoAMAT, KmmckatC4AcetoacylCoAMAT,
    KmmckatCoAMAT, KmmckatC6AcylCoAMAT, KmmckatC16AcylCoAMAT, KmmckatC14AcylCoAMAT,
    KmmckatC12AcylCoAMAT, KmmckatC10AcylCoAMAT, KmmckatC8AcylCoAMAT,
    KmmckatC4AcylCoAMAT, KmmckatAcetylCoAMAT, Keqmckat, C8KetoacylCoAMAT[t],
    C16KetoacylCoAMAT[t], C14KetoacylCoAMAT[t], C12KetoacylCoAMAT[t],
    C10KetoacylCoAMAT[t], C6KetoacylCoAMAT[t], C4AcetoacylCoAMAT[t], CoAMAT,
    C6AcylCoAMAT[t], C16AcylCoAMAT[t], C14AcylCoAMAT[t], C12AcylCoAMAT[t],
    C10AcylCoAMAT[t], C8AcylCoAMAT[t], C4AcylCoAMAT[t], AcetylCoAMAT[t],
    CE1, KmCE1, nE1, vmct1acet, kmct1acet, AcetateMAT, propacetmckat],
  vmckatC6 → MCKATA[sfmckatC6, Vmckat, KmmckatC6KetoacylCoAMAT,
    KmmckatC16KetoacylCoAMAT, KmmckatC14KetoacylCoAMAT, KmmckatC12KetoacylCoAMAT,
    KmmckatC10KetoacylCoAMAT, KmmckatC8KetoacylCoAMAT, KmmckatC4AcetoacylCoAMAT,
    KmmckatCoAMAT, KmmckatC4AcylCoAMAT, KmmckatC16AcylCoAMAT, KmmckatC14AcylCoAMAT,
    KmmckatC12AcylCoAMAT, KmmckatC10AcylCoAMAT, KmmckatC8AcylCoAMAT,
    KmmckatC6AcylCoAMAT, KmmckatAcetylCoAMAT, Keqmckat, C6KetoacylCoAMAT[t],
    C16KetoacylCoAMAT[t], C14KetoacylCoAMAT[t], C12KetoacylCoAMAT[t],
    C10KetoacylCoAMAT[t], C8KetoacylCoAMAT[t], C4AcetoacylCoAMAT[t], CoAMAT,
    C4AcylCoAMAT[t], C16AcylCoAMAT[t], C14AcylCoAMAT[t], C12AcylCoAMAT[t],
    C10AcylCoAMAT[t], C8AcylCoAMAT[t], C6AcylCoAMAT[t], AcetylCoAMAT[t],
```

```
  CE1, KmCE1, nE1, vmct1acet, kmct1acet, AcetateMAT, propacetmckat],
vmckatC4 → MCKATB[sfmckatC4, Vmckat, KmmckatC4AcetoacylCoAMAT,
  KmmckatC16KetoacylCoAMAT, KmmckatC14KetoacylCoAMAT, KmmckatC12KetoacylCoAMAT,
  KmmckatC10KetoacylCoAMAT, KmmckatC8KetoacylCoAMAT, KmmckatC6KetoacylCoAMAT,
  KmmckatCoAMAT, KmmckatC4AcylCoAMAT, KmmckatC16AcylCoAMAT, KmmckatC14AcylCoAMAT,
  KmmckatC12AcylCoAMAT, KmmckatC10AcylCoAMAT, KmmckatC8AcylCoAMAT,
  KmmckatC6AcylCoAMAT, KmmckatAcetylCoAMAT, Keqmckat, C4AcetoacylCoAMAT[t],
  C16KetoacylCoAMAT[t], C14KetoacylCoAMAT[t], C12KetoacylCoAMAT[t],
  C10KetoacylCoAMAT[t], C8KetoacylCoAMAT[t], C6KetoacylCoAMAT[t], CoAMAT,
  C4AcylCoAMAT[t], C16AcylCoAMAT[t], C14AcylCoAMAT[t], C12AcylCoAMAT[t],
  C10AcylCoAMAT[t], C8AcylCoAMAT[t], C6AcylCoAMAT[t], AcetylCoAMAT[t],
  CE1, KmCE1, nE1, vmct1acet, kmct1acet, AcetateMAT, propacetmckat],
vmtpC16 → MTP[sfmtpC16, Vmtp, KmmtpC16EnoylCoAMAT, KmmtpC14EnoylCoAMAT,
  KmmtpC12EnoylCoAMAT, KmmtpC10EnoylCoAMAT, KmmtpC8EnoylCoAMAT,
  KmmtpNADMAT, KmmtpCoAMAT, KmmtpC14AcylCoAMAT, KmmtpC16AcylCoAMAT,
  KmmtpC12AcylCoAMAT, KmmtpC10AcylCoAMAT, KmmtpC8AcylCoAMAT,
  KmmtpC6AcylCoAMAT, KmmtpNADHMAT, KmmtpAcetylCoAMAT, KicrotC4AcetoacylCoA,
  Keqmtp, C16EnoylCoAMAT[t], C14EnoylCoAMAT[t], C12EnoylCoAMAT[t],
  C10EnoylCoAMAT[t], C8EnoylCoAMAT[t], NADtMAT, CoAMAT, C14AcylCoAMAT[t],
  C16AcylCoAMAT[t], C12AcylCoAMAT[t], C10AcylCoAMAT[t], C8AcylCoAMAT[t],
  C6AcylCoAMAT[t], NADHMAT[t], AcetylCoAMAT[t], C4AcetoacylCoAMAT[t]],
vmtpC14 → MTP[sfmtpC14, Vmtp, KmmtpC14EnoylCoAMAT, KmmtpC16EnoylCoAMAT,
  KmmtpC12EnoylCoAMAT, KmmtpC10EnoylCoAMAT, KmmtpC8EnoylCoAMAT,
  KmmtpNADMAT, KmmtpCoAMAT, KmmtpC12AcylCoAMAT, KmmtpC16AcylCoAMAT,
  KmmtpC14AcylCoAMAT, KmmtpC10AcylCoAMAT, KmmtpC8AcylCoAMAT,
  KmmtpC6AcylCoAMAT, KmmtpNADHMAT, KmmtpAcetylCoAMAT, KicrotC4AcetoacylCoA,
  Keqmtp, C14EnoylCoAMAT[t], C16EnoylCoAMAT[t], C12EnoylCoAMAT[t],
  C10EnoylCoAMAT[t], C8EnoylCoAMAT[t], NADtMAT, CoAMAT, C12AcylCoAMAT[t],
  C16AcylCoAMAT[t], C14AcylCoAMAT[t], C10AcylCoAMAT[t], C8AcylCoAMAT[t],
  C6AcylCoAMAT[t], NADHMAT[t], AcetylCoAMAT[t], C4AcetoacylCoAMAT[t]],
vmtpC12 → MTP[sfmtpC12, Vmtp, KmmtpC12EnoylCoAMAT, KmmtpC16EnoylCoAMAT,
  KmmtpC14EnoylCoAMAT, KmmtpC10EnoylCoAMAT, KmmtpC8EnoylCoAMAT,
  KmmtpNADMAT, KmmtpCoAMAT, KmmtpC10AcylCoAMAT, KmmtpC16AcylCoAMAT,
  KmmtpC14AcylCoAMAT, KmmtpC12AcylCoAMAT, KmmtpC8AcylCoAMAT,
  KmmtpC6AcylCoAMAT, KmmtpNADHMAT, KmmtpAcetylCoAMAT, KicrotC4AcetoacylCoA,
  Keqmtp, C12EnoylCoAMAT[t], C16EnoylCoAMAT[t], C14EnoylCoAMAT[t],
  C10EnoylCoAMAT[t], C8EnoylCoAMAT[t], NADtMAT, CoAMAT, C10AcylCoAMAT[t],
  C16AcylCoAMAT[t], C14AcylCoAMAT[t], C12AcylCoAMAT[t], C8AcylCoAMAT[t],
  C6AcylCoAMAT[t], NADHMAT[t], AcetylCoAMAT[t], C4AcetoacylCoAMAT[t]],
vmtpC10 → MTP[sfmtpC10, Vmtp, KmmtpC10EnoylCoAMAT, KmmtpC16EnoylCoAMAT,
  KmmtpC14EnoylCoAMAT, KmmtpC12EnoylCoAMAT, KmmtpC8EnoylCoAMAT,
  KmmtpNADMAT, KmmtpCoAMAT, KmmtpC8AcylCoAMAT, KmmtpC16AcylCoAMAT,
  KmmtpC14AcylCoAMAT, KmmtpC12AcylCoAMAT, KmmtpC10AcylCoAMAT,
  KmmtpC6AcylCoAMAT, KmmtpNADHMAT, KmmtpAcetylCoAMAT, KicrotC4AcetoacylCoA,
  Keqmtp, C10EnoylCoAMAT[t], C16EnoylCoAMAT[t], C14EnoylCoAMAT[t],
  C12EnoylCoAMAT[t], C8EnoylCoAMAT[t], NADtMAT, CoAMAT, C8AcylCoAMAT[t],
  C16AcylCoAMAT[t], C14AcylCoAMAT[t], C12AcylCoAMAT[t], C10AcylCoAMAT[t],
  C6AcylCoAMAT[t], NADHMAT[t], AcetylCoAMAT[t], C4AcetoacylCoAMAT[t]],
vmtpC8 → MTP[sfmtpC8, Vmtp, KmmtpC8EnoylCoAMAT, KmmtpC16EnoylCoAMAT,
  KmmtpC14EnoylCoAMAT, KmmtpC12EnoylCoAMAT, KmmtpC10EnoylCoAMAT,
  KmmtpNADMAT, KmmtpCoAMAT, KmmtpC6AcylCoAMAT, KmmtpC16AcylCoAMAT,
  KmmtpC14AcylCoAMAT, KmmtpC12AcylCoAMAT, KmmtpC10AcylCoAMAT,
  KmmtpC8AcylCoAMAT, KmmtpNADHMAT, KmmtpAcetylCoAMAT, KicrotC4AcetoacylCoA,
  Keqmtp, C8EnoylCoAMAT[t], C16EnoylCoAMAT[t], C14EnoylCoAMAT[t],
  C12EnoylCoAMAT[t], C10EnoylCoAMAT[t], NADtMAT, CoAMAT, C6AcylCoAMAT[t],
```

```
      C16AcylCoAMAT[t], C14AcylCoAMAT[t], C12AcylCoAMAT[t], C10AcylCoAMAT[t],
      C8AcylCoAMAT[t], NADHMAT[t], AcetylCoAMAT[t], C4AcetoacylCoAMAT[t]],
    vacesink → RES[Ksacesink, AcetylCoAMAT[t], K1acesink],
    vfadhsink → RES[Ksfadhsink, FADHMAT[t], K1fadhsink],
    voxphnadh → OXPH[VmaxOxPh, KmnadhOxPh, KmnadOxPh,
      NADHMAT[t], NADtMAT - NADHMAT[t], vmct1acet, kmct1acet, AcetateMAT],
    vpdhnad → PDH[Vmaxpdh, KmnadPDH, KmnadhPDH, NADtMAT - NADHMAT[t], NADHMAT[t]]
   };


CoAMATX =
  {CoAMAT → CoAMATt - C16AcylCoAMAT[t] - C16EnoylCoAMAT[t] - C16HydroxyacylCoAMAT[t] -
      C16KetoacylCoAMAT[t] - C14AcylCoAMAT[t] - C14EnoylCoAMAT[t] -
      C14HydroxyacylCoAMAT[t] - C14KetoacylCoAMAT[t] - C12AcylCoAMAT[t] -
      C12EnoylCoAMAT[t] - C12HydroxyacylCoAMAT[t] - C12KetoacylCoAMAT[t] -
      C10AcylCoAMAT[t] - C10EnoylCoAMAT[t] - C10HydroxyacylCoAMAT[t] -
      C10KetoacylCoAMAT[t] - C8AcylCoAMAT[t] - C8EnoylCoAMAT[t] - C8HydroxyacylCoAMAT[t] -
      C8KetoacylCoAMAT[t] - C6AcylCoAMAT[t] - C6EnoylCoAMAT[t] - C6HydroxyacylCoAMAT[t] -
      C6KetoacylCoAMAT[t] - C4AcylCoAMAT[t] - C4EnoylCoAMAT[t] - C4HydroxyacylCoAMAT[t] -
      C4AcetoacylCoAMAT[t] - AcetylCoAMAT[t]};  (*+COATCA[t]};*)


Parm = {
    sfcpt1C16 → 1, Vcpt1 → 0.012, Kmcpt1C16AcylCoACYT → 13.8,
    Kmcpt1CarCYT → 250, Kmcpt1C16AcylCarCYT → 136, Kmcpt1CoACYT → 40.7,
    Kicpt1MalCoACYT → 9.1, Keqcpt1 → 0.45, ncpt1 → 2.4799,
    Vfcact → 0.42, Vrcact → 0.42, KmcactC16AcylCarCYT → 15,
    KmcactC14AcylCarCYT → 15, KmcactC12AcylCarCYT → 15, KmcactC10AcylCarCYT → 15,
    KmcactC8AcylCarCYT → 15, KmcactC6AcylCarCYT → 15, KmcactC4AcylCarCYT → 15,
    KmcactCarMAT → 130, KmcactC16AcylCarMAT → 15, KmcactC14AcylCarMAT → 15,
    KmcactC12AcylCarMAT → 15, KmcactC10AcylCarMAT → 15, KmcactC8AcylCarMAT → 15,
    KmcactC6AcylCarMAT → 15, KmcactC4AcylCarMAT → 15, KmcactCarCYT → 130,
    KicactC16AcylCarCYT → 56, KicactC14AcylCarCYT → 56, KicactC12AcylCarCYT → 56,
    KicactC10AcylCarCYT → 56, KicactC8AcylCarCYT → 56, KicactC6AcylCarCYT → 56,
    KicactC4AcylCarCYT → 56, KicactCarCYT → 200, Keqcact → 1,
    sfcpt2C16 → 0.85, sfcpt2C14 → 1, sfcpt2C12 → 0.95, sfcpt2C10 → 0.95,
    sfcpt2C8 → 0.35, sfcpt2C6 → 0.15, sfcpt2C4 → 0.01, Vcpt2 → 0.391,
    Kmcpt2C16AcylCarMAT → 51, Kmcpt2C14AcylCarMAT → 51, Kmcpt2C12AcylCarMAT → 51,
    Kmcpt2C10AcylCarMAT → 51, Kmcpt2C8AcylCarMAT → 51, Kmcpt2C6AcylCarMAT → 51,
    Kmcpt2C4AcylCarMAT → 51, Kmcpt2CoAMAT → 30, Kmcpt2C16AcylCoAMAT → 38,
    Kmcpt2C14AcylCoAMAT → 38, Kmcpt2C12AcylCoAMAT → 38,
    Kmcpt2C10AcylCoAMAT → 38, Kmcpt2C8AcylCoAMAT → 38, Kmcpt2C6AcylCoAMAT → 1000,
    Kmcpt2C4AcylCoAMAT → 1000000, Kmcpt2CarMAT → 350, Keqcpt2 → 2.22,
    sfvlcadC16 → 1, sfvlcadC14 → 0.42, sfvlcadC12 → 0.11, Vvlcad → 0.008,
    KmvlcadC16AcylCoAMAT → 6.5, KmvlcadC14AcylCoAMAT → 4, KmvlcadC12AcylCoAMAT → 2.7,
    KmvlcadFAD → 0.12, KmvlcadC16EnoylCoAMAT → 1.08, KmvlcadC14EnoylCoAMAT → 1.08,
    KmvlcadC12EnoylCoAMAT → 1.08, KmvlcadFADH → 24.2, Keqvlcad → 6,
    sflcadC16 → 0.9, sflcadC14 → 1, sflcadC12 → 0.9, sflcadC10 → 0.75, sflcadC8 → 0.4,
    Vlcad → 0.01, KmlcadC16AcylCoAMAT → 2.5, KmlcadC14AcylCoAMAT → 7.4,
    KmlcadC12AcylCoAMAT → 9, KmlcadC10AcylCoAMAT → 24.3, KmlcadC8AcylCoAMAT → 123,
    KmlcadFAD → 0.12, KmlcadC16EnoylCoAMAT → 1.08, KmlcadC14EnoylCoAMAT → 1.08,
    KmlcadC12EnoylCoAMAT → 1.08, KmlcadC10EnoylCoAMAT → 1.08,
    KmlcadC8EnoylCoAMAT → 1.08, KmlcadFADH → 24.2, Keqlcad → 6,
    sfmcadC12 → 0.38, sfmcadC10 → 0.8, sfmcadC8 → 0.87, sfmcadC6 → 1, sfmcadC4 → 0.12,
```

```
Vmcad → 0.081, KmmcadC12AcylCoAMAT → 5.7, KmmcadC10AcylCoAMAT → 5.4,
KmmcadC8AcylCoAMAT → 4, KmmcadC6AcylCoAMAT → 9.4, KmmcadC4AcylCoAMAT → 135,
KmmcadFAD → 0.12, KmmcadC12EnoylCoAMAT → 1.08, KmmcadC10EnoylCoAMAT → 1.08,
KmmcadC8EnoylCoAMAT → 1.08, KmmcadC6EnoylCoAMAT → 1.08,
KmmcadC4EnoylCoAMAT → 1.08, KmmcadFADH → 24.2, Keqmcad → 6,
sfscadC6 → 0.3, sfscadC4 → 1, Vscad → 0.081, KmscadC6AcylCoAMAT → 285,
KmscadC4AcylCoAMAT → 10.7, KmscadFAD → 0.12, KmscadC6EnoylCoAMAT → 1.08,
KmscadC4EnoylCoAMAT → 1.08, KmscadFADH → 24.2, Keqscad -> 6,
sfcrotC16 → 0.13, sfcrotC14 → 0.2, sfcrotC12 → 0.25, sfcrotC10 → 0.33, sfcrotC8 → 0.58,
sfcrotC6 → 0.83, sfcrotC4 → 1, Vcrot → 3.6, KmcrotC16EnoylCoAMAT → 150,
KmcrotC14EnoylCoAMAT → 100, KmcrotC12EnoylCoAMAT → 25, KmcrotC10EnoylCoAMAT → 25,
KmcrotC8EnoylCoAMAT → 25, KmcrotC6EnoylCoAMAT → 25, KmcrotC4EnoylCoAMAT → 40,
KmcrotC16HydroxyacylCoAMAT → 45, KmcrotC14HydroxyacylCoAMAT → 45,
KmcrotC12HydroxyacylCoAMAT → 45, KmcrotC10HydroxyacylCoAMAT → 45,
KmcrotC8HydroxyacylCoAMAT → 45, KmcrotC6HydroxyacylCoAMAT → 45,
KmcrotC4HydroxyacylCoAMAT → 45, KicrotC4AcetoacylCoA → 1.6, Keqcrot → 3.13,
sfmschadC16 → 0.6, sfmschadC14 → 0.5, sfmschadC12 → 0.43, sfmschadC10 → 0.64,
sfmschadC8 → 0.89, sfmschadC6 → 1, sfmschadC4 → 0.67, Vmschad → 1,
KmmschadC16HydroxyacylCoAMAT → 1.5, KmmschadC14HydroxyacylCoAMAT → 1.8,
KmmschadC12HydroxyacylCoAMAT → 3.7, KmmschadC10HydroxyacylCoAMAT → 8.8,
KmmschadC8HydroxyacylCoAMAT → 16.3, KmmschadC6HydroxyacylCoAMAT → 28.6,
KmmschadC4HydroxyacylCoAMAT → 69.9, KmmschadNADMAT → 58.5,
KmmschadC16KetoacylCoAMAT → 1.4, KmmschadC14KetoacylCoAMAT → 1.4,
KmmschadC12KetoacylCoAMAT → 1.6, KmmschadC10KetoacylCoAMAT → 2.3,
KmmschadC8KetoacylCoAMAT → 4.1, KmmschadC6KetoacylCoAMAT → 5.8,
KmmschadC4AcetoacylCoAMAT → 16.9, KmmschadNADHMAT → 5.4, Keqmschad → 2.17 * 10^-4,
sfmckatC16 → 0, sfmckatC14 → 0.2, sfmckatC12 → 0.38, sfmckatC10 → 0.65,
sfmckatC8 → 0.81, sfmckatC6 → 1, sfmckatC4 → 0.49, Vmckat → 0.377,
KmmckatC16KetoacylCoAMAT → 1.1, KmmckatC14KetoacylCoAMAT → 1.2,
KmmckatC12KetoacylCoAMAT → 1.3, KmmckatC10KetoacylCoAMAT → 2.1,
KmmckatC8KetoacylCoAMAT → 3.2, KmmckatC6KetoacylCoAMAT → 6.7,
KmmckatC4AcetoacylCoAMAT → 12.4, KmmckatCoAMAT → 26.6,
KmmckatC14AcylCoAMAT → 13.83, KmmckatC16AcylCoAMAT → 13.83,
KmmckatC12AcylCoAMAT → 13.83, KmmckatC10AcylCoAMAT → 13.83,
KmmckatC8AcylCoAMAT → 13.83, KmmckatC6AcylCoAMAT → 13.83,
KmmckatC4AcylCoAMAT → 13.83, KmmckatAcetylCoAMAT → 30, Keqmckat → 1051,
sfmtpC16 → 1, sfmtpC14 → 0.9, sfmtpC12 → 0.81, sfmtpC10 → 0.73, sfmtpC8 → 0.34,
Vmtp → 2.84, KmmtpC16EnoylCoAMAT → 25, KmmtpC14EnoylCoAMAT → 25,
KmmtpC12EnoylCoAMAT → 25, KmmtpC10EnoylCoAMAT → 25, KmmtpC8EnoylCoAMAT → 25,
KmmtpNADMAT → 60, KmmtpCoAMAT → 30, KmmtpC14AcylCoAMAT → 13.83,
KmmtpC16AcylCoAMAT → 13.83, KmmtpC12AcylCoAMAT → 13.83,
KmmtpC10AcylCoAMAT → 13.83, KmmtpC8AcylCoAMAT → 13.83, KmmtpC6AcylCoAMAT → 13.83,
KmmtpNADHMAT → 50, KmmtpAcetylCoAMAT → 30, Keqmtp → 0.71,
Ksacesink → 6000000, K1acesink → 70, Ksfadhsink → 6000000,
K1fadhsink → 0.46, Ksnadhsink → 6000000, K1nadhsink → 12,
C16AcylCoACYT → 25, CarCYT → 200, CoACYT → 140, MalCoACYT → 0,
CarMAT -> 950, FADtMAT → 0.77, NADtMAT → 250, CoAMATt → 5000,
VCYT → 2.2 * 10^-6, VMAT → 1.8 * 10^-6,
CE1 → 50, KmCE1 → 50, nE1 → 1,
Vmaxoxph → 0.155(*0.137,0.152 , 1.791*), KmnadhOxPh → 4.3,
KmnadOxPh → 780, VmaxPDH → 0.127, KmnadPDH → 60.7, KmnadhPDH → 40,
vmct1acet → 8.3, kmct1acet → 1.5, AcetateMAT → 0, propacetcpt1 → 0.12 ,
propacetsink → 0.13 , propacetcact → 0, propacetcpt2 → 0,
propacetmal → 0, propacetmckat → 0 (*0.1*), nc → 2};
```

```
InitialConditions = {
   C16AcylCarCYT[0] == 0, C16AcylCarMAT[0] == 0, C16AcylCoAMAT[0] == 0,
   C16EnoylCoAMAT[0] == 0, C16HydroxyacylCoAMAT[0] == 0, C16KetoacylCoAMAT[0] == 0,
   C14AcylCarCYT[0] == 0, C14AcylCarMAT[0] == 0, C14AcylCoAMAT[0] == 0,
   C14EnoylCoAMAT[0] == 0, C14HydroxyacylCoAMAT[0] == 0, C14KetoacylCoAMAT[0] == 0,
   C12AcylCarCYT[0] == 0, C12AcylCarMAT[0] == 0, C12AcylCoAMAT[0] == 0,
   C12EnoylCoAMAT[0] == 0, C12HydroxyacylCoAMAT[0] == 0, C12KetoacylCoAMAT[0] == 0,
   C10AcylCarCYT[0] == 0, C10AcylCarMAT[0] == 0, C10AcylCoAMAT[0] == 0,
   C10EnoylCoAMAT[0] == 0, C10HydroxyacylCoAMAT[0] == 0, C10KetoacylCoAMAT[0] == 0,
   C8AcylCarCYT[0] == 0, C8AcylCarMAT[0] == 0, C8AcylCoAMAT[0] == 0,
   C8EnoylCoAMAT[0] == 0, C8HydroxyacylCoAMAT[0] == 0, C8KetoacylCoAMAT[0] == 0,
   C6AcylCarCYT[0] == 0, C6AcylCarMAT[0] == 0, C6AcylCoAMAT[0] == 0,
   C6EnoylCoAMAT[0] == 0, C6HydroxyacylCoAMAT[0] == 0, C6KetoacylCoAMAT[0] == 0,
   C4AcylCarCYT[0] == 0, C4AcylCarMAT[0] == 0, C4AcylCoAMAT[0] == 0,
   C4EnoylCoAMAT[0] == 0, C4HydroxyacylCoAMAT[0] == 0, C4AcetoacylCoAMAT[0] == 0,
   AcetylCoAMAT[0] == 70, FADHMAT[0] == 0.46, NADHMAT[0] == 12};

Vars = {
   C16AcylCarCYT, C16AcylCarMAT, C16AcylCoAMAT,
   C16EnoylCoAMAT, C16HydroxyacylCoAMAT, C16KetoacylCoAMAT,
   C14AcylCarCYT, C14AcylCarMAT, C14AcylCoAMAT, C14EnoylCoAMAT,
   C14HydroxyacylCoAMAT, C14KetoacylCoAMAT,
   C12AcylCarCYT, C12AcylCarMAT, C12AcylCoAMAT, C12EnoylCoAMAT,
   C12HydroxyacylCoAMAT, C12KetoacylCoAMAT,
   C10AcylCarCYT, C10AcylCarMAT, C10AcylCoAMAT, C10EnoylCoAMAT,
   C10HydroxyacylCoAMAT, C10KetoacylCoAMAT,
   C8AcylCarCYT, C8AcylCarMAT, C8AcylCoAMAT, C8EnoylCoAMAT,
   C8HydroxyacylCoAMAT, C8KetoacylCoAMAT,
   C6AcylCarCYT, C6AcylCarMAT, C6AcylCoAMAT, C6EnoylCoAMAT,
   C6HydroxyacylCoAMAT, C6KetoacylCoAMAT,
   C4AcylCarCYT, C4AcylCarMAT, C4AcylCoAMAT, C4EnoylCoAMAT,
   C4HydroxyacylCoAMAT, C4AcetoacylCoAMAT,
   AcetylCoAMAT, FADHMAT, NADHMAT};
```

In[•]:=

```
TableForm[Odes];
TableForm[RateEqs];
TableForm[Odes /. RateEqs /. CoAMATX /. Parm ];
TableForm[RateEqs /. Parm ];
TableForm[InitialConditions];
```

In[•]:=

```
tsol = NDSolve[Join[Odes /. RateEqs /. CoAMATX /. Parm , InitialConditions],
   Vars, {t, 0, 1000000000}];
```

In[•]:=
```
Table[{Vars[[i]][t], (Vars[[i]][900000000] /. tsol)[[1]]}, {i, 1, Length[Vars]}]
```

Out[•]=
```
{{C16AcylCarCYT[t], 0.166282}, {C16AcylCarMAT[t], 0.34783},
 {C16AcylCoAMAT[t], 0.851861}, {C16EnoylCoAMAT[t], 0.0462384},
 {C16HydroxyacylCoAMAT[t], 0.144726}, {C16KetoacylCoAMAT[t], 0.000656667},
 {C14AcylCarCYT[t], 0.0363412}, {C14AcylCarMAT[t], 0.172621},
 {C14AcylCoAMAT[t], 1.8894}, {C14EnoylCoAMAT[t], 0.0515851},
 {C14HydroxyacylCoAMAT[t], 0.146228}, {C14KetoacylCoAMAT[t], 0.000662792},
 {C12AcylCarCYT[t], 0.0490286}, {C12AcylCarMAT[t], 0.232886},
 {C12AcylCoAMAT[t], 2.54902}, {C12EnoylCoAMAT[t], 0.0586393},
 {C12HydroxyacylCoAMAT[t], 0.177249}, {C12KetoacylCoAMAT[t], 0.000800836},
 {C10AcylCarCYT[t], 0.0852545}, {C10AcylCarMAT[t], 0.404959},
 {C10AcylCoAMAT[t], 4.43243}, {C10EnoylCoAMAT[t], 0.0644893},
 {C10HydroxyacylCoAMAT[t], 0.196237}, {C10KetoacylCoAMAT[t], 0.000883998},
 {C8AcylCarCYT[t], 0.0856958}, {C8AcylCarMAT[t], 0.407055},
 {C8AcylCoAMAT[t], 4.45537}, {C8EnoylCoAMAT[t], 0.139494},
 {C8HydroxyacylCoAMAT[t], 0.430973}, {C8KetoacylCoAMAT[t], 0.00194042},
 {C6AcylCarCYT[t], 0.226682}, {C6AcylCarMAT[t], 1.07674}, {C6AcylCoAMAT[t], 11.7853},
 {C6EnoylCoAMAT[t], 10.1182}, {C6HydroxyacylCoAMAT[t], 31.4843},
 {C6KetoacylCoAMAT[t], 0.141749}, {C4AcylCarCYT[t], 0.375688},
 {C4AcylCarMAT[t], 1.78452}, {C4AcylCoAMAT[t], 19.5322}, {C4EnoylCoAMAT[t], 38.0576},
 {C4HydroxyacylCoAMAT[t], 118.874}, {C4AcetoacylCoAMAT[t], 0.535337},
 {AcetylCoAMAT[t], 70.}, {FADHMAT[t], 0.46}, {NADHMAT[t], 11.4107}}
```

Steady state computation with varying palmitoyl-CoA (X) and acetate levels (Z)

In[•]:=
```
ParmScan[X_, Z_] := {
   sfcpt1C16 → 1, Vcpt1 → 0.012, Kmcpt1C16AcylCoACYT → 13.8,
   Kmcpt1CarCYT → 250, Kmcpt1C16AcylCarCYT → 136, Kmcpt1CoACYT → 40.7,
   Kicpt1MalCoACYT → 9.1, Keqcpt1 → 0.45, ncpt1 → 2.4799,
   Vfcact → 0.42, Vrcact → 0.42, KmcactC16AcylCarCYT → 15,
   KmcactC14AcylCarCYT → 15, KmcactC12AcylCarCYT → 15, KmcactC10AcylCarCYT → 15,
   KmcactC8AcylCarCYT → 15, KmcactC6AcylCarCYT → 15, KmcactC4AcylCarCYT → 15,
   KmcactCarMAT → 130, KmcactC16AcylCarMAT → 15, KmcactC14AcylCarMAT → 15,
   KmcactC12AcylCarMAT → 15, KmcactC10AcylCarMAT → 15, KmcactC8AcylCarMAT → 15,
   KmcactC6AcylCarMAT → 15, KmcactC4AcylCarMAT → 15, KmcactCarCYT → 130,
   KicactC16AcylCarCYT → 56, KicactC14AcylCarCYT → 56, KicactC12AcylCarCYT → 56,
   KicactC10AcylCarCYT → 56, KicactC8AcylCarCYT → 56, KicactC6AcylCarCYT → 56,
   KicactC4AcylCarCYT → 56, KicactCarCYT → 200, Keqcact → 1,
   sfcpt2C16 → 0.85, sfcpt2C14 → 1, sfcpt2C12 → 0.95, sfcpt2C10 → 0.95,
   sfcpt2C8 → 0.35, sfcpt2C6 → 0.15, sfcpt2C4 → 0.01, Vcpt2 → 0.391,
   Kmcpt2C16AcylCarMAT → 51, Kmcpt2C14AcylCarMAT → 51, Kmcpt2C12AcylCarMAT → 51,
   Kmcpt2C10AcylCarMAT → 51, Kmcpt2C8AcylCarMAT → 51, Kmcpt2C6AcylCarMAT → 51,
   Kmcpt2C4AcylCarMAT → 51, Kmcpt2CoAMAT → 30, Kmcpt2C16AcylCoAMAT → 38,
   Kmcpt2C14AcylCoAMAT → 38, Kmcpt2C12AcylCoAMAT → 38,
   Kmcpt2C10AcylCoAMAT → 38, Kmcpt2C8AcylCoAMAT → 38, Kmcpt2C6AcylCoAMAT → 1000,
   Kmcpt2C4AcylCoAMAT → 1000000, Kmcpt2CarMAT → 350, Keqcpt2 → 2.22,
   sfvlcadC16 → 1, sfvlcadC14 → 0.42, sfvlcadC12 → 0.11, Vvlcad → 0.008,
   KmvlcadC16AcylCoAMAT → 6.5, KmvlcadC14AcylCoAMAT → 4, KmvlcadC12AcylCoAMAT → 2.7,
   KmvlcadFAD → 0.12, KmvlcadC16EnoylCoAMAT → 1.08, KmvlcadC14EnoylCoAMAT → 1.08,
   KmvlcadC12EnoylCoAMAT → 1.08, KmvlcadFADH → 24.2, Keqvlcad → 6,
   sflcadC16 → 0.9, sflcadC14 → 1, sflcadC12 → 0.9, sflcadC10 → 0.75, sflcadC8 → 0.4,
   Vlcad → 0.01, KmlcadC16AcylCoAMAT → 2.5, KmlcadC14AcylCoAMAT → 7.4,
   KmlcadC12AcylCoAMAT → 9, KmlcadC10AcylCoAMAT → 24.3, KmlcadC8AcylCoAMAT → 123,
```

```
KmlcadFAD → 0.12, KmlcadC16EnoylCoAMAT → 1.08, KmlcadC14EnoylCoAMAT → 1.08,
KmlcadC12EnoylCoAMAT → 1.08, KmlcadC10EnoylCoAMAT → 1.08,
KmlcadC8EnoylCoAMAT → 1.08, KmlcadFADH → 24.2, Keqlcad → 6,
sfmcadC12 → 0.38, sfmcadC10 → 0.8, sfmcadC8 → 0.87, sfmcadC6 → 1, sfmcadC4 → 0.12,
Vmcad →  0.081, KmmcadC12AcylCoAMAT → 5.7, KmmcadC10AcylCoAMAT → 5.4,
KmmcadC8AcylCoAMAT → 4, KmmcadC6AcylCoAMAT → 9.4, KmmcadC4AcylCoAMAT → 135,
KmmcadFAD → 0.12, KmmcadC12EnoylCoAMAT → 1.08, KmmcadC10EnoylCoAMAT → 1.08,
KmmcadC8EnoylCoAMAT → 1.08, KmmcadC6EnoylCoAMAT → 1.08,
KmmcadC4EnoylCoAMAT → 1.08, KmmcadFADH → 24.2, Keqmcad → 6,
sfscadC6 → 0.3, sfscadC4 → 1, Vscad → 0.081, KmscadC6AcylCoAMAT → 285,
KmscadC4AcylCoAMAT → 10.7, KmscadFAD → 0.12, KmscadC6EnoylCoAMAT → 1.08,
KmscadC4EnoylCoAMAT → 1.08, KmscadFADH → 24.2, Keqscad -> 6,
sfcrotC16 → 0.13, sfcrotC14 → 0.2, sfcrotC12 → 0.25, sfcrotC10 → 0.33, sfcrotC8 → 0.58,
sfcrotC6 → 0.83, sfcrotC4 → 1, Vcrot → 3.6, KmcrotC16EnoylCoAMAT → 150,
KmcrotC14EnoylCoAMAT → 100, KmcrotC12EnoylCoAMAT → 25, KmcrotC10EnoylCoAMAT → 25,
KmcrotC8EnoylCoAMAT → 25, KmcrotC6EnoylCoAMAT → 25, KmcrotC4EnoylCoAMAT → 40,
KmcrotC16HydroxyacylCoAMAT → 45, KmcrotC14HydroxyacylCoAMAT → 45,
KmcrotC12HydroxyacylCoAMAT → 45, KmcrotC10HydroxyacylCoAMAT → 45,
KmcrotC8HydroxyacylCoAMAT → 45, KmcrotC6HydroxyacylCoAMAT → 45,
KmcrotC4HydroxyacylCoAMAT → 45, KicrotC4AcetoacylCoA → 1.6, Keqcrot → 3.13,
sfmschadC16 → 0.6, sfmschadC14 → 0.5, sfmschadC12 → 0.43, sfmschadC10 → 0.64,
sfmschadC8 → 0.89, sfmschadC6 → 1, sfmschadC4 → 0.67, Vmschad → 1,
KmmschadC16HydroxyacylCoAMAT → 1.5, KmmschadC14HydroxyacylCoAMAT → 1.8,
KmmschadC12HydroxyacylCoAMAT → 3.7, KmmschadC10HydroxyacylCoAMAT → 8.8,
KmmschadC8HydroxyacylCoAMAT → 16.3, KmmschadC6HydroxyacylCoAMAT → 28.6,
KmmschadC4HydroxyacylCoAMAT → 69.9, KmmschadNADMAT → 58.5,
KmmschadC16KetoacylCoAMAT → 1.4, KmmschadC14KetoacylCoAMAT → 1.4,
KmmschadC12KetoacylCoAMAT → 1.6, KmmschadC10KetoacylCoAMAT → 2.3,
KmmschadC8KetoacylCoAMAT → 4.1, KmmschadC6KetoacylCoAMAT → 5.8,
KmmschadC4AcetoacylCoAMAT → 16.9, KmmschadNADHMAT → 5.4, Keqmschad → 2.17 * 10^-4,
sfmckatC16 → 0, sfmckatC14 → 0.2, sfmckatC12 → 0.38, sfmckatC10 → 0.65,
sfmckatC8 → 0.81, sfmckatC6 → 1, sfmckatC4 → 0.49, Vmckat → 0.377,
KmmckatC16KetoacylCoAMAT → 1.1, KmmckatC14KetoacylCoAMAT → 1.2,
KmmckatC12KetoacylCoAMAT → 1.3, KmmckatC10KetoacylCoAMAT → 2.1,
KmmckatC8KetoacylCoAMAT → 3.2, KmmckatC6KetoacylCoAMAT → 6.7,
KmmckatC4AcetoacylCoAMAT → 12.4, KmmckatCoAMAT → 26.6,
KmmckatC14AcylCoAMAT → 13.83, KmmckatC16AcylCoAMAT → 13.83,
KmmckatC12AcylCoAMAT → 13.83, KmmckatC10AcylCoAMAT → 13.83,
KmmckatC8AcylCoAMAT → 13.83, KmmckatC6AcylCoAMAT → 13.83,
KmmckatC4AcylCoAMAT → 13.83, KmmckatAcetylCoAMAT → 30, Keqmckat → 1051,
sfmtpC16 → 1, sfmtpC14 → 0.9, sfmtpC12 → 0.81, sfmtpC10 → 0.73, sfmtpC8 → 0.34,
Vmtp → 2.84, KmmtpC16EnoylCoAMAT → 25, KmmtpC14EnoylCoAMAT → 25,
KmmtpC12EnoylCoAMAT → 25, KmmtpC10EnoylCoAMAT → 25, KmmtpC8EnoylCoAMAT → 25,
KmmtpNADMAT → 60, KmmtpCoAMAT → 30, KmmtpC14AcylCoAMAT → 13.83,
KmmtpC16AcylCoAMAT → 13.83, KmmtpC12AcylCoAMAT → 13.83,
KmmtpC10AcylCoAMAT → 13.83, KmmtpC8AcylCoAMAT → 13.83, KmmtpC6AcylCoAMAT → 13.83,
KmmtpNADHMAT → 50, KmmtpAcetylCoAMAT → 30, Keqmtp → 0.71,
Ksacesink → 6000000, K1acesink → 70, Ksfadhsink → 6000000,
K1fadhsink → 0.46, Ksnadhsink → 6000000, K1nadhsink → 12,
C16AcylCoACYT → X, CarCYT → 200, CoACYT → 140, MalCoACYT → 0,
CarMAT -> 950, FADtMAT → 0.77, NADtMAT → 250, CoAMATt → 5000,
VCYT → 2.2 * 10^-6, VMAT → 1.8 * 10^-6,
CE1 → 50, KmCE1 → 50, nE1 → 1,
Vnadqo → 0.155(*0.137,0.152 , 1.791*), KmnadhETC → 4.3,
KmnadETC → 780, VnadPDHo → 0.127, KmnadPDH → 60.7, KmnadhPDH → 40,
```

```
    vmct1acet → 8.3, kmct1acet → 1.5, AcetateMAT → Z, propacetcpt1 → 0.12 ,
    propacetsink → 0.13 , propacetcact → 0, propacetcpt2 → 0,
    propacetmal → 0, propacetmckat → 0 (*0.1*), nc → 2};
tsolScan[X_, Z_] := NDSolve[Join[Odes /. RateEqs /. CoAMATX /. ParmScan[X, Z],
    InitialConditions], Vars, {t, 0, 1000000000}];

SsScan[X_, Z_] := Module[{SSGuess},
  SSGuess := Table[{Vars[[i]][t],
     (Vars[[i]][900000000] /. tsolScan[X, Z])[[1]]}, {i, 1, Length[Vars]}];
  FindRoot[Table[Odes[[i, 2]] == 0, {i, 1, Length[Odes]}] /. RateEqs /. CoAMATX /.
    ParmScan[X, Z], SSGuess]]
```

In[•]:=
```
ScanDownNDSm[Ystart_, dY_, Yend_] := Monitor[Module[{SS, SSGuess},
   DataDownNDSfluxm = {};
   Xstart = 250;
   Xend = 0;
   YY = {0.0, 0.9};
   For[Y = Ystart, Y ≤ Yend,
    Z = YY[[Y]];
    tsolStart = tsolScan[Xend, Z];
    SSGuess = Table[{Vars[[i]][t],
       (Vars[[i]][900000000] /. tsolStart)[[1]]}, {i, 1, Length[Vars]}];
    SSGuess1 = SSGuess[[All, 1]];
    SSGuess2 = SSGuess[[All, 2]];
    SSGuess1int = SSGuess1 /. t → 0;
    InitialConditionsUD = Thread[SSGuess1int == SSGuess2];
    dX = 1;
    For[X = 250, X ≥ 0,
     tsolScanNDS = NDSolve[Join[Odes /. RateEqs /. CoAMATX /. ParmScan[X, Z],
        InitialConditionsUD], Vars, {t, 0, 1000000000}];
     SSGuess = Table[{Vars[[i]][t], (Vars[[i]][900000000] /. tsolScanNDS)[[1]]},
       {i, 1, Length[Vars]}];
     SSGuess1 = SSGuess[[All, 1]];
     SSGuess2 = SSGuess[[All, 2]];
     SSGuess1int = SSGuess1 /. t → 0;
     InitialConditionsUD = Thread[SSGuess1int == SSGuess2];
     SS = Thread[SSGuess1 → SSGuess2];

     AppendTo[DataDownNDSfluxm,
      {X, Z, 10^3 vcpt1C16 /. RateEqs /. CoAMATX /. ParmScan[X, Z] /. SS}];
     X = X - dX;];
    Y = Y + dY;]
  ], ProgressIndicator[X, {Xstart, Xend}]]
```

In[•]:=
```
ScanDownNDSm[1, 1, 2]
```

```
In[•]:= ScanUpNDSm[Ystart_, dY_, Yend_] := Monitor[Module[{SS, SSGuess},
   DataUpNDSfluxm = {};
   Xstart = 0;
   Xend = 250;
   YY = {0.0, 0.9};
   For[Y = Ystart, Y ≤ Yend,
    Z = YY[[Y]];
    tsolStart = tsolScan[Xstart, Z ];
    SSGuess = Table[{Vars[[i]][t],
       (Vars[[i]][900000000] /. tsolStart)[[1]]}, {i, 1, Length[Vars]}];
    SSGuess1 = SSGuess[[All, 1]];
    SSGuess2 = SSGuess[[All, 2]];
    SSGuess1int = SSGuess1 /. t → 0;
    InitialConditionsUD = Thread[SSGuess1int == SSGuess2];

    dX = 1;
    For[X = 0, X ≤ 250,

     tsolScanNDS = NDSolve[Join[Odes /. RateEqs /. CoAMATX /. ParmScan[X, Z],
        InitialConditionsUD], Vars, {t, 0, 1000000000}];
     SSGuess = Table[{Vars[[i]][t], (Vars[[i]][900000000] /. tsolScanNDS)[[1]]},
       {i, 1, Length[Vars]}];
     SSGuess1 = SSGuess[[All, 1]];
     SSGuess2 = SSGuess[[All, 2]];
     SSGuess1int = SSGuess1 /. t → 0;
     InitialConditionsUD = Thread[SSGuess1int == SSGuess2];
     SS = Thread[SSGuess1 → SSGuess2];

     AppendTo[DataUpNDSfluxm,
      {X, Z, 10^3 vcpt1C16 /. RateEqs /. CoAMATX /. ParmScan[X, Z] /. SS}];

     X = X + dX;];
    Y = Y + dY;]
   ], ProgressIndicator[X, {Xstart, Xend}]]

In[•]:= ScanUpNDSm[1, 1, 2]
```

In[ ]:=
```
p5new =
 ListLinePlot[{DataUpNDSfluxm[[1 ;; 251, {1, 3}]], DataUpNDSfluxm[[252 ;; 502, {1, 3}]],
   DataDownNDSfluxm[[1 ;; 251, {1, 3}]], DataDownNDSfluxm[[252 ;; 502, {1, 3}]]},
  PlotRange → All, PlotStyle → {Red, Blue, Red, Blue},
  AxesStyle → Directive[Black, 16], LabelStyle → Directive[Black],
  PlotLegends → Placed[LineLegend[{"0 mM", "3 mM"}, LabelStyle → {FontSize → 16}],
    {Center, Bottom}], PlotLabel → "Acetate levels",
  Frame → {{True, False}, {True, False}}, FrameLabel →
   {{"Uptake Flux(μmol.min^-1.gProtein^-1)", None}, {"Palmitoyl-CoA(μM)", None}},
  BaseStyle → {FontSize → 18, FontWeight → "3", AbsoluteThickness[2]},
  FrameStyle → Thickness[0.00005], ImageSize → Scaled[0.30], AspectRatio → 0.75]
```

Out[ ]=

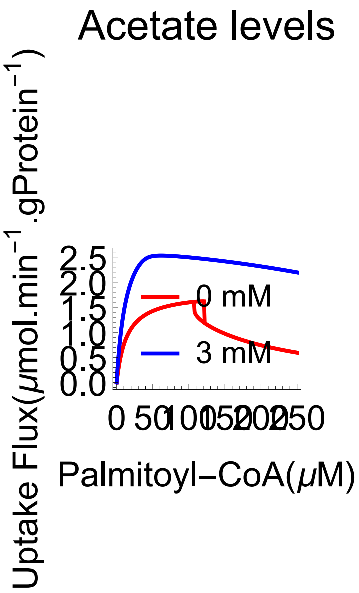

Supplement: S3 Appendix — (ZIP) [file pcbi.1009259.s014.zip › MFAOExtensionwithSCFAsRegulationRev.pdf]
