# Supplementary material for: Bistability in fatty-acid oxidation resulting from substrate inhibition
Source: PLoS Comput Biol. 2021 Aug 12;17(8):e1009259. doi: 10.1371/journal.pcbi.1009259 (PMC8396765; doi:10.1371/journal.pcbi.1009259)

Mitochondrial Fatty Acid Oxidation Kinetic Model Extension with p46Shc Regulation of MCKAT

Definitions of the various functions

In[•]:= $$\mathrm{CPT1}[\mathrm{sf\_},\mathrm{V\_},\mathrm{Kms1\_},\mathrm{Kms2\_},\mathrm{Kmp1\_},\mathrm{Kmp2\_},\mathrm{Ki1\_},\mathrm{Keq\_},\mathrm{S1\_},\mathrm{S2\_},\mathrm{P1\_},\mathrm{P2\_},\mathrm{I1\_},\mathrm{n\_}] := \frac{\mathrm{sf}*\mathrm{V}*\left(\frac{\mathrm{S1}*\mathrm{S2}}{\mathrm{Kms1}*\mathrm{Kms2}}-\frac{\mathrm{P1}*\mathrm{P2}}{\mathrm{Kms1}*\mathrm{Kms2}*\mathrm{Keq}}\right)}{\left(1+\frac{\mathrm{S1}}{\mathrm{Kms1}}+\frac{\mathrm{P1}}{\mathrm{Kmp1}}+\left(\frac{\mathrm{I1}}{\mathrm{Ki1}}\right)^{n}\right)*\left(1+\frac{\mathrm{S2}}{\mathrm{Kms2}}+\frac{\mathrm{P2}}{\mathrm{Kmp2}}\right)}$$

In[•]:= $$\mathrm{CACT}[\mathrm{Vf\_},\mathrm{Vr\_},\mathrm{Kms1\_},\mathrm{Kms2\_},\mathrm{Kmp1\_},\mathrm{Kmp2\_},\mathrm{Kis1\_},\mathrm{Kip2\_},\mathrm{Keq\_},\mathrm{S1\_},\mathrm{S2\_},\mathrm{P1\_},\mathrm{P2\_}] := \frac{\mathrm{Vf}*\left(\mathrm{S1}*\mathrm{S2}-\frac{\mathrm{P1}*\mathrm{P2}}{\mathrm{Keq}}\right)}{\mathrm{S1}*\mathrm{S2}+\mathrm{Kms2}*\mathrm{S1}+\mathrm{Kms1}*\mathrm{S2}*\left(1+\frac{\mathrm{P2}}{\mathrm{Kip2}}\right)+\frac{\mathrm{Vf}}{\mathrm{Vr}*\mathrm{Keq}}*\left(\mathrm{Kmp2}*\mathrm{P1}*\left(1+\frac{\mathrm{S1}}{\mathrm{Kis1}}\right)+\mathrm{P2}*(\mathrm{Kmp1}+\mathrm{P1})\right)}$$

In[•]:= $$\mathrm{CPT2}[\mathrm{sf\_},\mathrm{V\_},\mathrm{Kms1\_},\mathrm{Kms2\_},\mathrm{Kms3\_},\mathrm{Kms4\_},\mathrm{Kms5\_},\mathrm{Kms6\_},\mathrm{Kms7\_},\mathrm{Kms8\_},\mathrm{Kmp1\_},\mathrm{Kmp2\_},\mathrm{Kmp3\_},\mathrm{Kmp4\_},\mathrm{Kmp5\_},\mathrm{Kmp6\_},\mathrm{Kmp7\_},\mathrm{Kmp8\_},\mathrm{Keq\_},\mathrm{S1\_},\mathrm{S2\_},\mathrm{S3\_},\mathrm{S4\_},\mathrm{S5\_},\mathrm{S6\_},\mathrm{S7\_},\mathrm{S8\_},\mathrm{P1\_},\mathrm{P2\_},\mathrm{P3\_},\mathrm{P4\_},\mathrm{P5\_},\mathrm{P6\_},\mathrm{P7\_},\mathrm{P8\_}] := \left(\mathrm{sf}*\mathrm{V}*\left(\frac{\mathrm{S1}*\mathrm{S8}}{\mathrm{Kms1}*\mathrm{Kms8}}-\frac{\mathrm{P1}*\mathrm{P8}}{\mathrm{Kms1}*\mathrm{Kms8}*\mathrm{Keq}}\right)\right)\Big/\left(\left(1+\frac{\mathrm{S1}}{\mathrm{Kms1}}+\frac{\mathrm{P1}}{\mathrm{Kmp1}}+\frac{\mathrm{S2}}{\mathrm{Kms2}}+\frac{\mathrm{P2}}{\mathrm{Kmp2}}+\frac{\mathrm{S3}}{\mathrm{Kms3}}+\frac{\mathrm{P3}}{\mathrm{Kmp3}}+\frac{\mathrm{S4}}{\mathrm{Kms4}}+\frac{\mathrm{P4}}{\mathrm{Kmp4}}+\frac{\mathrm{S5}}{\mathrm{Kms5}}+\frac{\mathrm{P5}}{\mathrm{Kmp5}}+\frac{\mathrm{S6}}{\mathrm{Kms6}}+\frac{\mathrm{P6}}{\mathrm{Kmp6}}+\frac{\mathrm{S7}}{\mathrm{Kms7}}+\frac{\mathrm{P7}}{\mathrm{Kmp7}}\right)*\left(1+\frac{\mathrm{S8}}{\mathrm{Kms8}}+\frac{\mathrm{P8}}{\mathrm{Kmp8}}\right)\right)$$

In[•]:= $$\mathrm{VLCAD}[\mathrm{sf\_},\mathrm{V\_},\mathrm{Kms1\_},\mathrm{Kms2\_},\mathrm{Kms3\_},\mathrm{Kms4\_},\mathrm{Kmp1\_},\mathrm{Kmp2\_},\mathrm{Kmp3\_},\mathrm{Kmp4\_},\mathrm{Keq\_},\mathrm{S1\_},\mathrm{S2\_},\mathrm{S3\_},\mathrm{S4\_},\mathrm{P1\_},\mathrm{P2\_},\mathrm{P3\_},\mathrm{P4\_}] := \frac{\mathrm{sf}*\mathrm{V}*\left(\frac{\mathrm{S1}*(\mathrm{S4}-\mathrm{P4})}{\mathrm{Kms1}*\mathrm{Kms4}}-\frac{\mathrm{P1}*\mathrm{P4}}{\mathrm{Kms1}*\mathrm{Kms4}*\mathrm{Keq}}\right)}{\left(1+\frac{\mathrm{S1}}{\mathrm{Kms1}}+\frac{\mathrm{P1}}{\mathrm{Kmp1}}+\frac{\mathrm{S2}}{\mathrm{Kms2}}+\frac{\mathrm{P2}}{\mathrm{Kmp2}}+\frac{\mathrm{S3}}{\mathrm{Kms3}}+\frac{\mathrm{P3}}{\mathrm{Kmp3}}\right)*\left(1+\frac{(\mathrm{S4}-\mathrm{P4})}{\mathrm{Kms4}}+\frac{\mathrm{P4}}{\mathrm{Kmp4}}\right)}$$

In[•]:= $$\mathrm{LCAD}[\mathrm{sf\_},\mathrm{V\_},\mathrm{Kms1\_},\mathrm{Kms2\_},\mathrm{Kms3\_},\mathrm{Kms4\_},\mathrm{Kms5\_},\mathrm{Kms6\_},\mathrm{Kmp1\_},\mathrm{Kmp2\_},\mathrm{Kmp3\_},\mathrm{Kmp4\_},\mathrm{Kmp5\_},\mathrm{Kmp6\_},\mathrm{Keq\_},\mathrm{S1\_},\mathrm{S2\_},\mathrm{S3\_},\mathrm{S4\_},\mathrm{S5\_},\mathrm{S6\_},\mathrm{P1\_},\mathrm{P2\_},\mathrm{P3\_},\mathrm{P4\_},\mathrm{P5\_},\mathrm{P6\_}] := \frac{\mathrm{sf}*\mathrm{V}*\left(\frac{\mathrm{S1}*(\mathrm{S6}-\mathrm{P6})}{\mathrm{Kms1}*\mathrm{Kms6}}-\frac{\mathrm{P1}*\mathrm{P6}}{\mathrm{Kms1}*\mathrm{Kms6}*\mathrm{Keq}}\right)}{\left(1+\frac{\mathrm{S1}}{\mathrm{Kms1}}+\frac{\mathrm{P1}}{\mathrm{Kmp1}}+\frac{\mathrm{S2}}{\mathrm{Kms2}}+\frac{\mathrm{P2}}{\mathrm{Kmp2}}+\frac{\mathrm{S3}}{\mathrm{Kms3}}+\frac{\mathrm{P3}}{\mathrm{Kmp3}}+\frac{\mathrm{S4}}{\mathrm{Kms4}}+\frac{\mathrm{P4}}{\mathrm{Kmp4}}+\frac{\mathrm{S5}}{\mathrm{Kms5}}+\frac{\mathrm{P5}}{\mathrm{Kmp5}}\right)*\left(1+\frac{(\mathrm{S6}-\mathrm{P6})}{\mathrm{Kms6}}+\frac{\mathrm{P6}}{\mathrm{Kmp6}}\right)}$$

In[•]:= $$\mathrm{MCAD}[\mathrm{sf\_},\mathrm{V\_},\mathrm{Kms1\_},\mathrm{Kms2\_},\mathrm{Kms3\_},\mathrm{Kms4\_},\mathrm{Kms5\_},\mathrm{Kms6\_},\mathrm{Kmp1\_},\mathrm{Kmp2\_},\mathrm{Kmp3\_},\mathrm{Kmp4\_},\mathrm{Kmp5\_},\mathrm{Kmp6\_},\mathrm{Keq\_},\mathrm{S1\_},\mathrm{S2\_},\mathrm{S3\_},\mathrm{S4\_},\mathrm{S5\_},\mathrm{S6\_},\mathrm{P1\_},\mathrm{P2\_},\mathrm{P3\_},\mathrm{P4\_},\mathrm{P5\_},\mathrm{P6\_}] := \frac{\mathrm{sf}*\mathrm{V}*\left(\frac{\mathrm{S1}*(\mathrm{S6}-\mathrm{P6})}{\mathrm{Kms1}*\mathrm{Kms6}}-\frac{\mathrm{P1}*\mathrm{P6}}{\mathrm{Kms1}*\mathrm{Kms6}*\mathrm{Keq}}\right)}{\left(1+\frac{\mathrm{S1}}{\mathrm{Kms1}}+\frac{\mathrm{P1}}{\mathrm{Kmp1}}+\frac{\mathrm{S2}}{\mathrm{Kms2}}+\frac{\mathrm{P2}}{\mathrm{Kmp2}}+\frac{\mathrm{S3}}{\mathrm{Kms3}}+\frac{\mathrm{P3}}{\mathrm{Kmp3}}+\frac{\mathrm{S4}}{\mathrm{Kms4}}+\frac{\mathrm{P4}}{\mathrm{Kmp4}}+\frac{\mathrm{S5}}{\mathrm{Kms5}}+\frac{\mathrm{P5}}{\mathrm{Kmp5}}\right)*\left(1+\frac{(\mathrm{S6}-\mathrm{P6})}{\mathrm{Kms6}}+\frac{\mathrm{P6}}{\mathrm{Kmp6}}\right)}$$

In[•]:= $$\mathrm{SCAD}[\mathrm{sf\_},\mathrm{V\_},\mathrm{Kms1\_},\mathrm{Kms2\_},\mathrm{Kms3\_},\mathrm{Kmp1\_},\mathrm{Kmp2\_},\mathrm{Kmp3\_},\mathrm{Keq\_},\mathrm{S1\_},\mathrm{S2\_},\mathrm{S3\_},\mathrm{P1\_},\mathrm{P2\_},\mathrm{P3\_}] := \frac{\mathrm{sf}*\mathrm{V}*\left(\frac{\mathrm{S1}*(\mathrm{S3}-\mathrm{P3})}{\mathrm{Kms1}*\mathrm{Kms3}}-\frac{\mathrm{P1}*\mathrm{P3}}{\mathrm{Kms1}*\mathrm{Kms3}*\mathrm{Keq}}\right)}{\left(1+\frac{\mathrm{S1}}{\mathrm{Kms1}}+\frac{\mathrm{P1}}{\mathrm{Kmp1}}+\frac{\mathrm{S2}}{\mathrm{Kms2}}+\frac{\mathrm{P2}}{\mathrm{Kmp2}}\right)*\left(1+\frac{(\mathrm{S3}-\mathrm{P3})}{\mathrm{Kms3}}+\frac{\mathrm{P3}}{\mathrm{Kmp3}}\right)}$$

```
In[•]:= CROT[sf_, V_, Kms1_, Kms2_, Kms3_, Kms4_, Kms5_, Kms6_, Kms7_,
  Kmp1_, Kmp2_, Kmp3_, Kmp4_, Kmp5_, Kmp6_, Kmp7_, Ki1_, Keq_, S1_, S2_,
  S3_, S4_, S5_, S6_, S7_, P1_, P2_, P3_, P4_, P5_, P6_, P7_, I1_] :=
  (sf * V * (S1/Kms1 - P1/(Kms1*Keq))) /
  (1 + S1/Kms1 + P1/Kmp1 + S2/Kms2 + P2/Kmp2 + S3/Kms3 + P3/Kmp3 + S4/Kms4 + P4/Kmp4 + S5/Kms5 + P5/Kmp5 + S6/Kms6 + P6/Kmp6 + S7/Kms7 + P7/Kmp7 + I1/Ki1)

In[•]:= MSCHAD[sf_, V_, Kms1_, Kms2_, Kms3_, Kms4_, Kms5_, Kms6_, Kms7_, Kms8_, Kmp1_, Kmp2_,
  Kmp3_, Kmp4_, Kmp5_, Kmp6_, Kmp7_, Kmp8_, Keq_, S1_, S2_, S3_, S4_, S5_, S6_, S7_, S8_,
  P1_, P2_, P3_, P4_, P5_, P6_, P7_, P8_] := (sf * V * ((S1 * (S8 - P8))/(Kms1 * Kms8) - (P1 * P8)/(Kms1 * Kms8 * Keq))) /
  ((1 + S1/Kms1 + P1/Kmp1 + S2/Kms2 + P2/Kmp2 + S3/Kms3 + P3/Kmp3 + S4/Kms4 + P4/Kmp4 +
     S5/Kms5 + P5/Kmp5 + S6/Kms6 + P6/Kmp6 + S7/Kms7 + P7/Kmp7) * (1 + (S8 - P8)/Kms8 + P8/Kmp8))

In[•]:= MCKATA[sf_, V_, Kms1_, Kms2_, Kms3_, Kms4_, Kms5_, Kms6_, Kms7_, Kms8_, Kmp1_,
  Kmp2_, Kmp3_, Kmp4_, Kmp5_, Kmp6_, Kmp7_, Kmp8_, Keq_, S1_, S2_, S3_, S4_,
  S5_, S6_, S7_, S8_, P1_, P2_, P3_, P4_, P5_, P6_, P7_, P8_, E1_, KmE1_, nm_] :=
  (sf * V * (1 + 0.30 * E1) * ((S1 * S8)/(Kms1 * Kms8) - (P1 * P8)/(Kms1 * Kms8 * Keq))) /
  ((1 + S1/Kms1 + P1/Kmp1 + S2/Kms2 + P2/Kmp2 + S3/Kms3 + P3/Kmp3 + S4/Kms4 + P4/Kmp4 + S5/Kms5 + P5/Kmp5 +
     S6/Kms6 + P6/Kmp6 + S7/Kms7 + P7/Kmp7 + P8/Kmp8) * (1 + S8/Kms8 + P8/Kmp8))   (*Exp[(1- E1/KmE1)]*)

In[•]:= MCKATB[sf_, V_, Kms1_, Kms2_, Kms3_, Kms4_, Kms5_, Kms6_, Kms7_, Kms8_, Kmp1_,
  Kmp2_, Kmp3_, Kmp4_, Kmp5_, Kmp6_, Kmp7_, Kmp8_, Keq_, S1_, S2_, S3_, S4_,
  S5_, S6_, S7_, S8_, P1_, P2_, P3_, P4_, P5_, P6_, P7_, P8_, E1_, KmE1_, nm_] :=
  (sf * V * (1 + 0.30 * E1) * ((S1 * S8)/(Kms1 * Kms8) - (P8 * P8)/(Kms1 * Kms8 * Keq))) /
  ((1 + S1/Kms1 + P1/Kmp1 + S2/Kms2 + P2/Kmp2 + S3/Kms3 + P3/Kmp3 + S4/Kms4 + P4/Kmp4 + S5/Kms5 +
     P5/Kmp5 + S6/Kms6 + P6/Kmp6 + S7/Kms7 + P7/Kmp7 + P8/Kmp8) * (1 + S8/Kms8 + P8/Kmp8))

In[•]:= MTP[sf_, V_, Kms1_, Kms2_, Kms3_, Kms4_, Kms5_, Kms7_, Kms8_, Kmp1_,
  Kmp2_, Kmp3_, Kmp4_, Kmp5_, Kmp6_, Kmp7_, Kmp8_, Ki1_, Keq_, S1_, S2_,
  S3_, S4_, S5_, S7_, S8_, P1_, P2_, P3_, P4_, P5_, P6_, P7_, P8_, I1_] :=
  (sf * V * ((S1 * (S7 - P7) * S8)/(Kms1 * Kms7 * Kms8) - (P1 * P7 * P8)/(Kms1 * Kms7 * Kms8 * Keq))) /
  ((1 + S1/Kms1 + P1/Kmp1 + S2/Kms2 + P2/Kmp2 + S3/Kms3 + P3/Kmp3 + S4/Kms4 + P4/Kmp4 + S5/Kms5 +
     P5/Kmp5 + P6/Kmp6 + I1/Ki1) * (1 + (S7 - P7)/Kms7 + P7/Kmp7) * (1 + S8/Kms8 + P8/Kmp8))

In[•]:= RES[Ks_, S_, K1_] := Ks * (S - K1)

In[•]:=
```

Define the differential equations

In[]:= Odes = {

$$C16AcylCarCYT'[t] == \frac{vcpt1C16 - vcactC16}{VCYT},$$

$$C16AcylCarMAT'[t] == \frac{vcactC16 - vcpt2C16}{VMAT},$$

$$C16AcylCoAMAT'[t] == \frac{vcpt2C16 - vvlcadC16 - vlcadC16}{VMAT},$$

$$C16EnoylCoAMAT'[t] == \frac{vvlcadC16 + vlcadC16 - vcrotC16 - vmtpC16}{VMAT},$$

$$C16HydroxyacylCoAMAT'[t] == \frac{vcrotC16 - vmschadC16}{VMAT},$$

$$C16KetoacylCoAMAT'[t] == \frac{vmschadC16 - vmckatC16}{VMAT},$$

$$C14AcylCarCYT'[t] == \frac{-vcactC14}{VCYT},$$

$$C14AcylCarMAT'[t] == \frac{vcactC14 - vcpt2C14}{VMAT},$$

$$C14AcylCoAMAT'[t] == \frac{vcpt2C14 + vmtpC16 + vmckatC16 - vvlcadC14 - vlcadC14}{VMAT},$$

$$C14EnoylCoAMAT'[t] == \frac{vvlcadC14 + vlcadC14 - vcrotC14 - vmtpC14}{VMAT},$$

$$C14HydroxyacylCoAMAT'[t] == \frac{vcrotC14 - vmschadC14}{VMAT},$$

$$C14KetoacylCoAMAT'[t] == \frac{vmschadC14 - vmckatC14}{VMAT},$$

$$C12AcylCarCYT'[t] == \frac{-vcactC12}{VCYT},$$

$$C12AcylCarMAT'[t] == \frac{vcactC12 - vcpt2C12}{VMAT},$$

$$C12AcylCoAMAT'[t] == \frac{vcpt2C12 + vmtpC14 + vmckatC14 - vvlcadC12 - vlcadC12 - vmcadC12}{VMAT},$$

$$C12EnoylCoAMAT'[t] == \frac{vvlcadC12 + vlcadC12 + vmcadC12 - vcrotC12 - vmtpC12}{VMAT},$$

$$C12HydroxyacylCoAMAT'[t] == \frac{vcrotC12 - vmschadC12}{VMAT},$$

$$C12KetoacylCoAMAT'[t] == \frac{vmschadC12 - vmckatC12}{VMAT},$$

$$C10AcylCarCYT'[t] == \frac{-vcactC10}{VCYT},$$

$$C10AcylCarMAT'[t] == \frac{vcactC10 - vcpt2C10}{VMAT},$$

$$C10AcylCoAMAT'[t] == \frac{vcpt2C10 + vmtpC12 + vmckatC12 - vlcadC10 - vmcadC10}{VMAT},$$

```
C10EnoylCoAMAT'[t] == (vlcadC10 + vmcadC10 - vcrotC10 - vmtpC10)/VMAT,
C10HydroxyacylCoAMAT'[t] == (vcrotC10 - vmschadC10)/VMAT,
C10KetoacylCoAMAT'[t] == (vmschadC10 - vmckatC10)/VMAT,
C8AcylCarCYT'[t] == (-vcactC8)/VCYT,
C8AcylCarMAT'[t] == (vcactC8 - vcpt2C8)/VMAT,
C8AcylCoAMAT'[t] == (vcpt2C8 + vmtpC10 + vmckatC10 - vlcadC8 - vmcadC8)/VMAT,
C8EnoylCoAMAT'[t] == (vlcadC8 + vmcadC8 - vcrotC8 - vmtpC8)/VMAT,
C8HydroxyacylCoAMAT'[t] == (vcrotC8 - vmschadC8)/VMAT,
C8KetoacylCoAMAT'[t] == (vmschadC8 - vmckatC8)/VMAT,
C6AcylCarCYT'[t] == (-vcactC6)/VCYT,
C6AcylCarMAT'[t] == (vcactC6 - vcpt2C6)/VMAT,
C6AcylCoAMAT'[t] == (vcpt2C6 + vmtpC8 + vmckatC8 - vmcadC6 - vscadC6)/VMAT,
C6EnoylCoAMAT'[t] == (vmcadC6 + vscadC6 - vcrotC6)/VMAT,
C6HydroxyacylCoAMAT'[t] == (vcrotC6 - vmschadC6)/VMAT,
C6KetoacylCoAMAT'[t] == (vmschadC6 - vmckatC6)/VMAT,
C4AcylCarCYT'[t] == (-vcactC4)/VCYT,
C4AcylCarMAT'[t] == (vcactC4 - vcpt2C4)/VMAT,
C4AcylCoAMAT'[t] == (vcpt2C4 + vmckatC6 - vmcadC4 - vscadC4)/VMAT,
C4EnoylCoAMAT'[t] == (vmcadC4 + vscadC4 - vcrotC4)/VMAT,
C4HydroxyacylCoAMAT'[t] == (vcrotC4 - vmschadC4)/VMAT,
C4AcetoacylCoAMAT'[t] == (vmschadC4 - vmckatC4)/VMAT};

RateEqs = {vcpt1C16 → CPT1[sfcpt1C16, Vcpt1, Kmcpt1C16AcylCoACYT,
    Kmcpt1CarCYT, Kmcpt1C16AcylCarCYT, Kmcpt1CoACYT, Kicpt1MalCoACYT, Keqcpt1,
    C16AcylCoACYT, CarCYT, C16AcylCarCYT[t], CoACYT, MalCoACYT, ncpt1],
  vcactC16 → CACT[Vfcact, Vrcact, KmcactC16AcylCarCYT, KmcactCarMAT,
```

```
    KmcactC16AcylCarMAT, KmcactCarCYT, KicactC16AcylCarCYT, KicactCarCYT,
    Keqcact, C16AcylCarCYT[t], CarMAT, C16AcylCarMAT[t], CarCYT],
  vcactC14 → CACT[Vfcact, Vrcact, KmcactC14AcylCarCYT, KmcactCarMAT,
    KmcactC14AcylCarMAT, KmcactCarCYT, KicactC14AcylCarCYT, KicactCarCYT,
    Keqcact, C14AcylCarCYT[t], CarMAT, C14AcylCarMAT[t], CarCYT],
  vcactC12 → CACT[Vfcact, Vrcact, KmcactC12AcylCarCYT, KmcactCarMAT,
    KmcactC12AcylCarMAT, KmcactCarCYT, KicactC12AcylCarCYT, KicactCarCYT,
    Keqcact, C12AcylCarCYT[t], CarMAT, C12AcylCarMAT[t], CarCYT],
  vcactC10 → CACT[Vfcact, Vrcact, KmcactC10AcylCarCYT, KmcactCarMAT,
    KmcactC10AcylCarMAT, KmcactCarCYT, KicactC10AcylCarCYT, KicactCarCYT,
    Keqcact, C10AcylCarCYT[t], CarMAT, C10AcylCarMAT[t], CarCYT],
  vcactC8 → CACT[Vfcact, Vrcact, KmcactC8AcylCarCYT, KmcactCarMAT,
    KmcactC8AcylCarMAT, KmcactCarCYT, KicactC8AcylCarCYT, KicactCarCYT,
    Keqcact, C8AcylCarCYT[t], CarMAT, C8AcylCarMAT[t], CarCYT],
  vcactC6 → CACT[Vfcact, Vrcact, KmcactC6AcylCarCYT, KmcactCarMAT,
    KmcactC6AcylCarMAT, KmcactCarCYT, KicactC6AcylCarCYT, KicactCarCYT,
    Keqcact, C6AcylCarCYT[t], CarMAT, C6AcylCarMAT[t], CarCYT],
  vcactC4 → CACT[Vfcact, Vrcact, KmcactC4AcylCarCYT, KmcactCarMAT,
    KmcactC4AcylCarMAT, KmcactCarCYT, KicactC4AcylCarCYT, KicactCarCYT,
    Keqcact, C4AcylCarCYT[t], CarMAT, C4AcylCarMAT[t], CarCYT],
  vcpt2C16 → CPT2[sfcpt2C16, Vcpt2, Kmcpt2C16AcylCarMAT, Kmcpt2C14AcylCarMAT,
    Kmcpt2C12AcylCarMAT, Kmcpt2C10AcylCarMAT, Kmcpt2C8AcylCarMAT, Kmcpt2C6AcylCarMAT,
    Kmcpt2C4AcylCarMAT, Kmcpt2CoAMAT, Kmcpt2C16AcylCoAMAT, Kmcpt2C14AcylCoAMAT,
    Kmcpt2C12AcylCoAMAT, Kmcpt2C10AcylCoAMAT, Kmcpt2C8AcylCoAMAT, Kmcpt2C6AcylCoAMAT,
    Kmcpt2C4AcylCoAMAT, Kmcpt2CarMAT, Keqcpt2, C16AcylCarMAT[t], C14AcylCarMAT[t],
    C12AcylCarMAT[t], C10AcylCarMAT[t], C8AcylCarMAT[t], C6AcylCarMAT[t],
    C4AcylCarMAT[t], CoAMAT, C16AcylCoAMAT[t], C14AcylCoAMAT[t], C12AcylCoAMAT[t],
    C10AcylCoAMAT[t], C8AcylCoAMAT[t], C6AcylCoAMAT[t], C4AcylCoAMAT[t], CarMAT],
  vcpt2C14 → CPT2[sfcpt2C14, Vcpt2, Kmcpt2C14AcylCarMAT, Kmcpt2C16AcylCarMAT,
    Kmcpt2C12AcylCarMAT, Kmcpt2C10AcylCarMAT, Kmcpt2C8AcylCarMAT, Kmcpt2C6AcylCarMAT,
    Kmcpt2C4AcylCoAMAT, Kmcpt2CoAMAT, Kmcpt2C14AcylCoAMAT, Kmcpt2C16AcylCoAMAT,
    Kmcpt2C12AcylCoAMAT, Kmcpt2C10AcylCoAMAT, Kmcpt2C8AcylCoAMAT, Kmcpt2C6AcylCoAMAT,
    Kmcpt2C4AcylCoAMAT, Kmcpt2CarMAT, Keqcpt2, C14AcylCarMAT[t], C16AcylCarMAT[t],
    C12AcylCarMAT[t], C10AcylCarMAT[t], C8AcylCarMAT[t], C6AcylCarMAT[t],
    C4AcylCarMAT[t], CoAMAT, C14AcylCoAMAT[t], C16AcylCoAMAT[t], C12AcylCoAMAT[t],
    C10AcylCoAMAT[t], C8AcylCoAMAT[t], C6AcylCoAMAT[t], C4AcylCoAMAT[t], CarMAT],
  vcpt2C12 → CPT2[sfcpt2C12, Vcpt2, Kmcpt2C12AcylCarMAT, Kmcpt2C16AcylCarMAT,
    Kmcpt2C14AcylCarMAT, Kmcpt2C10AcylCarMAT, Kmcpt2C8AcylCarMAT, Kmcpt2C6AcylCarMAT,
    Kmcpt2C4AcylCarMAT, Kmcpt2CoAMAT, Kmcpt2C12AcylCoAMAT, Kmcpt2C16AcylCoAMAT,
    Kmcpt2C14AcylCoAMAT, Kmcpt2C10AcylCoAMAT, Kmcpt2C8AcylCoAMAT, Kmcpt2C6AcylCoAMAT,
    Kmcpt2C4AcylCoAMAT, Kmcpt2CarMAT, Keqcpt2, C12AcylCarMAT[t], C16AcylCarMAT[t],
    C14AcylCarMAT[t], C10AcylCarMAT[t], C8AcylCarMAT[t], C6AcylCarMAT[t],
    C4AcylCarMAT[t], CoAMAT, C12AcylCoAMAT[t], C16AcylCoAMAT[t], C14AcylCoAMAT[t],
    C10AcylCoAMAT[t], C8AcylCoAMAT[t], C6AcylCoAMAT[t], C4AcylCoAMAT[t], CarMAT],
  vcpt2C10 → CPT2[sfcpt2C10, Vcpt2, Kmcpt2C10AcylCarMAT, Kmcpt2C16AcylCarMAT,
    Kmcpt2C14AcylCarMAT, Kmcpt2C12AcylCarMAT, Kmcpt2C8AcylCarMAT, Kmcpt2C6AcylCarMAT,
    Kmcpt2C4AcylCarMAT, Kmcpt2CoAMAT, Kmcpt2C10AcylCoAMAT, Kmcpt2C16AcylCoAMAT,
    Kmcpt2C14AcylCoAMAT, Kmcpt2C12AcylCoAMAT, Kmcpt2C8AcylCoAMAT, Kmcpt2C6AcylCoAMAT,
    Kmcpt2C4AcylCoAMAT, Kmcpt2CarMAT, Keqcpt2, C10AcylCarMAT[t], C16AcylCarMAT[t],
    C14AcylCarMAT[t], C12AcylCarMAT[t], C8AcylCarMAT[t], C6AcylCarMAT[t],
    C4AcylCarMAT[t], CoAMAT, C10AcylCoAMAT[t], C16AcylCoAMAT[t], C14AcylCoAMAT[t],
    C12AcylCoAMAT[t], C8AcylCoAMAT[t], C6AcylCoAMAT[t], C4AcylCoAMAT[t], CarMAT],
  vcpt2C8 → CPT2[sfcpt2C8, Vcpt2, Kmcpt2C8AcylCarMAT, Kmcpt2C16AcylCarMAT,
    Kmcpt2C14AcylCarMAT, Kmcpt2C12AcylCarMAT, Kmcpt2C10AcylCarMAT, Kmcpt2C6AcylCarMAT,
    Kmcpt2C4AcylCarMAT, Kmcpt2CoAMAT, Kmcpt2C8AcylCoAMAT, Kmcpt2C16AcylCoAMAT,
```

```
    Kmcpt2C14AcylCoAMAT, Kmcpt2C12AcylCoAMAT, Kmcpt2C10AcylCoAMAT, Kmcpt2C6AcylCoAMAT,
    Kmcpt2C4AcylCoAMAT, Kmcpt2CarMAT, Keqcpt2, C8AcylCarMAT[t], C16AcylCarMAT[t],
    C14AcylCarMAT[t], C12AcylCarMAT[t], C10AcylCarMAT[t], C6AcylCarMAT[t],
    C4AcylCarMAT[t], CoAMAT, C8AcylCoAMAT[t], C16AcylCoAMAT[t], C14AcylCoAMAT[t],
    C12AcylCoAMAT[t], C10AcylCoAMAT[t], C6AcylCoAMAT[t], C4AcylCoAMAT[t], CarMAT],
  vcpt2C6 → CPT2[sfcpt2C6, Vcpt2, Kmcpt2C6AcylCarMAT, Kmcpt2C16AcylCarMAT,
    Kmcpt2C14AcylCarMAT, Kmcpt2C12AcylCarMAT, Kmcpt2C10AcylCarMAT, Kmcpt2C8AcylCarMAT,
    Kmcpt2C4AcylCarMAT, Kmcpt2CoAMAT, Kmcpt2C6AcylCoAMAT, Kmcpt2C16AcylCoAMAT,
    Kmcpt2C14AcylCoAMAT, Kmcpt2C12AcylCoAMAT, Kmcpt2C10AcylCoAMAT, Kmcpt2C8AcylCoAMAT,
    Kmcpt2C4AcylCoAMAT, Kmcpt2CarMAT, Keqcpt2, C6AcylCarMAT[t], C16AcylCarMAT[t],
    C14AcylCarMAT[t], C12AcylCarMAT[t], C10AcylCarMAT[t], C8AcylCarMAT[t],
    C4AcylCarMAT[t], CoAMAT, C6AcylCoAMAT[t], C16AcylCoAMAT[t], C14AcylCoAMAT[t],
    C12AcylCoAMAT[t], C10AcylCoAMAT[t], C8AcylCoAMAT[t], C4AcylCoAMAT[t], CarMAT],
  vcpt2C4 → CPT2[sfcpt2C4, Vcpt2, Kmcpt2C4AcylCarMAT, Kmcpt2C16AcylCarMAT,
    Kmcpt2C14AcylCarMAT, Kmcpt2C12AcylCarMAT, Kmcpt2C10AcylCarMAT, Kmcpt2C8AcylCarMAT,
    Kmcpt2C6AcylCarMAT, Kmcpt2CoAMAT, Kmcpt2C4AcylCoAMAT, Kmcpt2C16AcylCoAMAT,
    Kmcpt2C14AcylCoAMAT, Kmcpt2C12AcylCoAMAT, Kmcpt2C10AcylCoAMAT, Kmcpt2C8AcylCoAMAT,
    Kmcpt2C6AcylCoAMAT, Kmcpt2CarMAT, Keqcpt2, C4AcylCarMAT[t], C16AcylCarMAT[t],
    C14AcylCarMAT[t], C12AcylCarMAT[t], C10AcylCarMAT[t], C8AcylCarMAT[t],
    C6AcylCarMAT[t], CoAMAT, C4AcylCoAMAT[t], C16AcylCoAMAT[t], C14AcylCoAMAT[t],
    C12AcylCoAMAT[t], C10AcylCoAMAT[t], C8AcylCoAMAT[t], C6AcylCoAMAT[t], CarMAT],
  vvlcadC16 → VLCAD[sfvlcadC16, Vvlcad, KmvlcadC16AcylCoAMAT, KmvlcadC14AcylCoAMAT,
    KmvlcadC12AcylCoAMAT, KmvlcadFAD, KmvlcadC16EnoylCoAMAT,
    KmvlcadC14EnoylCoAMAT, KmvlcadC12EnoylCoAMAT, KmvlcadFADH, Keqvlcad,
    C16AcylCoAMAT[t], C14AcylCoAMAT[t], C12AcylCoAMAT[t], FADtMAT,
    C16EnoylCoAMAT[t], C14EnoylCoAMAT[t], C12EnoylCoAMAT[t], FADHMAT],
  vvlcadC14 → VLCAD[sfvlcadC14, Vvlcad, KmvlcadC14AcylCoAMAT, KmvlcadC16AcylCoAMAT,
    KmvlcadC12AcylCoAMAT, KmvlcadFAD, KmvlcadC14EnoylCoAMAT,
    KmvlcadC16EnoylCoAMAT, KmvlcadC12EnoylCoAMAT, KmvlcadFADH, Keqvlcad,
    C14AcylCoAMAT[t], C16AcylCoAMAT[t], C12AcylCoAMAT[t], FADtMAT,
    C14EnoylCoAMAT[t], C16EnoylCoAMAT[t], C12EnoylCoAMAT[t], FADHMAT],
  vvlcadC12 → VLCAD[sfvlcadC12, Vvlcad, KmvlcadC12AcylCoAMAT, KmvlcadC16AcylCoAMAT,
    KmvlcadC14AcylCoAMAT, KmvlcadFAD, KmvlcadC12EnoylCoAMAT,
    KmvlcadC16EnoylCoAMAT, KmvlcadC14EnoylCoAMAT, KmvlcadFADH, Keqvlcad,
    C12AcylCoAMAT[t], C16AcylCoAMAT[t], C14AcylCoAMAT[t], FADtMAT,
    C12EnoylCoAMAT[t], C16EnoylCoAMAT[t], C14EnoylCoAMAT[t], FADHMAT],
  vlcadC16 → LCAD[sflcadC16, Vlcad, KmlcadC16AcylCoAMAT, KmlcadC14AcylCoAMAT,
    KmlcadC12AcylCoAMAT, KmlcadC10AcylCoAMAT, KmlcadC8AcylCoAMAT, KmlcadFAD,
    KmlcadC16EnoylCoAMAT, KmlcadC14EnoylCoAMAT, KmlcadC12EnoylCoAMAT,
    KmlcadC10EnoylCoAMAT, KmlcadC8EnoylCoAMAT, KmlcadFADH, Keqlcad,
    C16AcylCoAMAT[t], C14AcylCoAMAT[t], C12AcylCoAMAT[t], C10AcylCoAMAT[t],
    C8AcylCoAMAT[t], FADtMAT, C16EnoylCoAMAT[t], C14EnoylCoAMAT[t],
    C12EnoylCoAMAT[t], C10EnoylCoAMAT[t], C8EnoylCoAMAT[t], FADHMAT],
  vlcadC14 → LCAD[sflcadC14, Vlcad, KmlcadC14AcylCoAMAT, KmlcadC16AcylCoAMAT,
    KmlcadC12AcylCoAMAT, KmlcadC10AcylCoAMAT, KmlcadC8AcylCoAMAT, KmlcadFAD,
    KmlcadC14EnoylCoAMAT, KmlcadC16EnoylCoAMAT, KmlcadC12EnoylCoAMAT,
    KmlcadC10EnoylCoAMAT, KmlcadC8EnoylCoAMAT, KmlcadFADH, Keqlcad,
    C14AcylCoAMAT[t], C16AcylCoAMAT[t], C12AcylCoAMAT[t], C10AcylCoAMAT[t],
    C8AcylCoAMAT[t], FADtMAT, C14EnoylCoAMAT[t], C16EnoylCoAMAT[t],
    C12EnoylCoAMAT[t], C10EnoylCoAMAT[t], C8EnoylCoAMAT[t], FADHMAT],
  vlcadC12 → LCAD[sflcadC12, Vlcad, KmlcadC12AcylCoAMAT, KmlcadC16AcylCoAMAT,
    KmlcadC14AcylCoAMAT, KmlcadC10AcylCoAMAT, KmlcadC8AcylCoAMAT, KmlcadFAD,
    KmlcadC12EnoylCoAMAT, KmlcadC16EnoylCoAMAT, KmlcadC14EnoylCoAMAT,
    KmlcadC10EnoylCoAMAT, KmlcadC8EnoylCoAMAT, KmlcadFADH, Keqlcad,
    C12AcylCoAMAT[t], C16AcylCoAMAT[t], C14AcylCoAMAT[t], C10AcylCoAMAT[t],
```

```
    C8AcylCoAMAT[t], FADtMAT, C14EnoylCoAMAT[t], C16EnoylCoAMAT[t],
    C14EnoylCoAMAT[t], C10EnoylCoAMAT[t], C8EnoylCoAMAT[t], FADHMAT],
  vlcadC10 → LCAD[sflcadC10, Vlcad, KmlcadC10AcylCoAMAT, KmlcadC16AcylCoAMAT,
    KmlcadC14AcylCoAMAT, KmlcadC12AcylCoAMAT, KmlcadC8AcylCoAMAT, KmlcadFAD,
    KmlcadC10EnoylCoAMAT, KmlcadC16EnoylCoAMAT, KmlcadC14EnoylCoAMAT,
    KmlcadC12EnoylCoAMAT, KmlcadC8EnoylCoAMAT, KmlcadFADH, Keqlcad,
    C10AcylCoAMAT[t], C16AcylCoAMAT[t], C14AcylCoAMAT[t], C12AcylCoAMAT[t],
    C8AcylCoAMAT[t], FADtMAT, C10EnoylCoAMAT[t], C16EnoylCoAMAT[t],
    C14EnoylCoAMAT[t], C12EnoylCoAMAT[t], C8EnoylCoAMAT[t], FADHMAT],
  vlcadC8 → LCAD[sflcadC8, Vlcad, KmlcadC8AcylCoAMAT, KmlcadC16AcylCoAMAT,
    KmlcadC14AcylCoAMAT, KmlcadC12AcylCoAMAT, KmlcadC10AcylCoAMAT, KmlcadFAD,
    KmlcadC8EnoylCoAMAT, KmlcadC16EnoylCoAMAT, KmlcadC14EnoylCoAMAT,
    KmlcadC12EnoylCoAMAT, KmlcadC10EnoylCoAMAT, KmlcadFADH, Keqlcad,
    C8AcylCoAMAT[t], C16AcylCoAMAT[t], C14AcylCoAMAT[t], C12AcylCoAMAT[t],
    C10AcylCoAMAT[t], FADtMAT, C8EnoylCoAMAT[t], C16EnoylCoAMAT[t],
    C14EnoylCoAMAT[t], C12EnoylCoAMAT[t], C10EnoylCoAMAT[t], FADHMAT],
  vmcadC12 → MCAD[sfmcadC12, Vmcad, KmmcadC12AcylCoAMAT, KmmcadC10AcylCoAMAT,
    KmmcadC8AcylCoAMAT, KmmcadC6AcylCoAMAT, KmmcadC4AcylCoAMAT, KmmcadFAD,
    KmmcadC12EnoylCoAMAT, KmmcadC10EnoylCoAMAT, KmmcadC8EnoylCoAMAT,
    KmmcadC6EnoylCoAMAT, KmmcadC4EnoylCoAMAT, KmmcadFADH, Keqmcad,
    C12AcylCoAMAT[t], C10AcylCoAMAT[t], C8AcylCoAMAT[t], C6AcylCoAMAT[t],
    C4AcylCoAMAT[t], FADtMAT, C12EnoylCoAMAT[t], C10EnoylCoAMAT[t],
    C8EnoylCoAMAT[t], C6EnoylCoAMAT[t], C4EnoylCoAMAT[t], FADHMAT],
  vmcadC10 → MCAD[sfmcadC10, Vmcad, KmmcadC10AcylCoAMAT, KmmcadC12AcylCoAMAT,
    KmmcadC8AcylCoAMAT, KmmcadC6AcylCoAMAT, KmmcadC4AcylCoAMAT, KmmcadFAD,
    KmmcadC10EnoylCoAMAT, KmmcadC12EnoylCoAMAT, KmmcadC8EnoylCoAMAT,
    KmmcadC6EnoylCoAMAT, KmmcadC4EnoylCoAMAT, KmmcadFADH, Keqmcad,
    C10AcylCoAMAT[t], C12AcylCoAMAT[t], C8AcylCoAMAT[t], C6AcylCoAMAT[t],
    C4AcylCoAMAT[t], FADtMAT, C10EnoylCoAMAT[t], C12EnoylCoAMAT[t],
    C8EnoylCoAMAT[t], C6EnoylCoAMAT[t], C4EnoylCoAMAT[t], FADHMAT],
  vmcadC8 → MCAD[sfmcadC8, Vmcad, KmmcadC8AcylCoAMAT, KmmcadC12AcylCoAMAT,
    KmmcadC10AcylCoAMAT, KmmcadC6AcylCoAMAT, KmmcadC4AcylCoAMAT, KmmcadFAD,
    KmmcadC8EnoylCoAMAT, KmmcadC12EnoylCoAMAT, KmmcadC10EnoylCoAMAT,
    KmmcadC6EnoylCoAMAT, KmmcadC4EnoylCoAMAT, KmmcadFADH, Keqmcad,
    C8AcylCoAMAT[t], C12AcylCoAMAT[t], C10AcylCoAMAT[t], C6AcylCoAMAT[t],
    C4AcylCoAMAT[t], FADtMAT, C8EnoylCoAMAT[t], C12EnoylCoAMAT[t],
    C10EnoylCoAMAT[t], C6EnoylCoAMAT[t], C4EnoylCoAMAT[t], FADHMAT],
  vmcadC6 → MCAD[sfmcadC6, Vmcad, KmmcadC6AcylCoAMAT, KmmcadC12AcylCoAMAT,
    KmmcadC10AcylCoAMAT, KmmcadC8AcylCoAMAT, KmmcadC4AcylCoAMAT, KmmcadFAD,
    KmmcadC6EnoylCoAMAT, KmmcadC12EnoylCoAMAT, KmmcadC10EnoylCoAMAT,
    KmmcadC8EnoylCoAMAT, KmmcadC4EnoylCoAMAT, KmmcadFADH, Keqmcad,
    C6AcylCoAMAT[t], C12AcylCoAMAT[t], C10AcylCoAMAT[t], C8AcylCoAMAT[t],
    C4AcylCoAMAT[t], FADtMAT, C6EnoylCoAMAT[t], C12EnoylCoAMAT[t],
    C10EnoylCoAMAT[t], C8EnoylCoAMAT[t], C4EnoylCoAMAT[t], FADHMAT],
  vmcadC4 → MCAD[sfmcadC4, Vmcad, KmmcadC4AcylCoAMAT, KmmcadC12AcylCoAMAT,
    KmmcadC10AcylCoAMAT, KmmcadC8AcylCoAMAT, KmmcadC6AcylCoAMAT, KmmcadFAD,
    KmmcadC4EnoylCoAMAT, KmmcadC12EnoylCoAMAT, KmmcadC10EnoylCoAMAT,
    KmmcadC8EnoylCoAMAT, KmmcadC6EnoylCoAMAT, KmmcadFADH, Keqmcad,
    C4AcylCoAMAT[t], C12AcylCoAMAT[t], C10AcylCoAMAT[t], C8AcylCoAMAT[t],
    C6AcylCoAMAT[t], FADtMAT, C4EnoylCoAMAT[t], C12EnoylCoAMAT[t],
    C10EnoylCoAMAT[t], C8EnoylCoAMAT[t], C6EnoylCoAMAT[t], FADHMAT],
  vscadC6 → SCAD[sfscadC6, Vscad, KmscadC6AcylCoAMAT, KmscadC4AcylCoAMAT, KmscadFAD,
    KmscadC6EnoylCoAMAT, KmscadC4EnoylCoAMAT, KmscadFADH, Keqscad, C6AcylCoAMAT[t],
    C4AcylCoAMAT[t], FADtMAT, C6EnoylCoAMAT[t], C4EnoylCoAMAT[t], FADHMAT],
  vscadC4 → SCAD[sfscadC4, Vscad, KmscadC4AcylCoAMAT, KmscadC6AcylCoAMAT, KmscadFAD,
```

```
    KmscadC4EnoylCoAMAT, KmscadC6EnoylCoAMAT, KmscadFADH, Keqscad, C4AcylCoAMAT[t],
    C6AcylCoAMAT[t], FADtMAT, C4EnoylCoAMAT[t], C6EnoylCoAMAT[t], FADHMAT],
  vcrotC16 → CROT[sfcrotC16, Vcrot, KmcrotC16EnoylCoAMAT, KmcrotC14EnoylCoAMAT,
    KmcrotC12EnoylCoAMAT, KmcrotC10EnoylCoAMAT, KmcrotC8EnoylCoAMAT,
    KmcrotC6EnoylCoAMAT, KmcrotC4EnoylCoAMAT, KmcrotC16HydroxyacylCoAMAT,
    KmcrotC14HydroxyacylCoAMAT, KmcrotC12HydroxyacylCoAMAT,
    KmcrotC10HydroxyacylCoAMAT, KmcrotC8HydroxyacylCoAMAT,
    KmcrotC6HydroxyacylCoAMAT, KmcrotC4HydroxyacylCoAMAT, KicrotC4AcetoacylCoA,
    Keqcrot, C16EnoylCoAMAT[t], C14EnoylCoAMAT[t], C12EnoylCoAMAT[t],
    C10EnoylCoAMAT[t], C8EnoylCoAMAT[t], C6EnoylCoAMAT[t],
    C4EnoylCoAMAT[t], C16HydroxyacylCoAMAT[t], C14HydroxyacylCoAMAT[t],
    C12HydroxyacylCoAMAT[t], C10HydroxyacylCoAMAT[t], C8HydroxyacylCoAMAT[t],
    C6HydroxyacylCoAMAT[t], C4HydroxyacylCoAMAT[t], C4AcetoacylCoAMAT[t]],
  vcrotC14 → CROT[sfcrotC14, Vcrot, KmcrotC14EnoylCoAMAT, KmcrotC16EnoylCoAMAT,
    KmcrotC12EnoylCoAMAT, KmcrotC10EnoylCoAMAT, KmcrotC8EnoylCoAMAT,
    KmcrotC6EnoylCoAMAT, KmcrotC4EnoylCoAMAT, KmcrotC14HydroxyacylCoAMAT,
    KmcrotC16HydroxyacylCoAMAT, KmcrotC12HydroxyacylCoAMAT,
    KmcrotC10HydroxyacylCoAMAT, KmcrotC8HydroxyacylCoAMAT,
    KmcrotC6HydroxyacylCoAMAT, KmcrotC4HydroxyacylCoAMAT, KicrotC4AcetoacylCoA,
    Keqcrot, C14EnoylCoAMAT[t], C16EnoylCoAMAT[t], C12EnoylCoAMAT[t],
    C10EnoylCoAMAT[t], C8EnoylCoAMAT[t], C6EnoylCoAMAT[t],
    C4EnoylCoAMAT[t], C14HydroxyacylCoAMAT[t], C16HydroxyacylCoAMAT[t],
    C12HydroxyacylCoAMAT[t], C10HydroxyacylCoAMAT[t], C8HydroxyacylCoAMAT[t],
    C6HydroxyacylCoAMAT[t], C4HydroxyacylCoAMAT[t], C4AcetoacylCoAMAT[t]],
  vcrotC12 → CROT[sfcrotC12, Vcrot, KmcrotC12EnoylCoAMAT, KmcrotC16EnoylCoAMAT,
    KmcrotC14EnoylCoAMAT, KmcrotC10EnoylCoAMAT, KmcrotC8EnoylCoAMAT,
    KmcrotC6EnoylCoAMAT, KmcrotC4EnoylCoAMAT, KmcrotC12HydroxyacylCoAMAT,
    KmcrotC16HydroxyacylCoAMAT, KmcrotC14HydroxyacylCoAMAT,
    KmcrotC10HydroxyacylCoAMAT, KmcrotC8HydroxyacylCoAMAT,
    KmcrotC6HydroxyacylCoAMAT, KmcrotC4HydroxyacylCoAMAT, KicrotC4AcetoacylCoA,
    Keqcrot, C12EnoylCoAMAT[t], C16EnoylCoAMAT[t], C14EnoylCoAMAT[t],
    C10EnoylCoAMAT[t], C8EnoylCoAMAT[t], C6EnoylCoAMAT[t],
    C4EnoylCoAMAT[t], C12HydroxyacylCoAMAT[t], C16HydroxyacylCoAMAT[t],
    C14HydroxyacylCoAMAT[t], C10HydroxyacylCoAMAT[t], C8HydroxyacylCoAMAT[t],
    C6HydroxyacylCoAMAT[t], C4HydroxyacylCoAMAT[t], C4AcetoacylCoAMAT[t]],
  vcrotC10 → CROT[sfcrotC10, Vcrot, KmcrotC10EnoylCoAMAT, KmcrotC16EnoylCoAMAT,
    KmcrotC14EnoylCoAMAT, KmcrotC12EnoylCoAMAT, KmcrotC8EnoylCoAMAT,
    KmcrotC6EnoylCoAMAT, KmcrotC4EnoylCoAMAT, KmcrotC10HydroxyacylCoAMAT,
    KmcrotC16HydroxyacylCoAMAT, KmcrotC14HydroxyacylCoAMAT,
    KmcrotC12HydroxyacylCoAMAT, KmcrotC8HydroxyacylCoAMAT,
    KmcrotC6HydroxyacylCoAMAT, KmcrotC4HydroxyacylCoAMAT, KicrotC4AcetoacylCoA,
    Keqcrot, C10EnoylCoAMAT[t], C16EnoylCoAMAT[t], C14EnoylCoAMAT[t],
    C12EnoylCoAMAT[t], C8EnoylCoAMAT[t], C6EnoylCoAMAT[t],
    C4EnoylCoAMAT[t], C10HydroxyacylCoAMAT[t], C16HydroxyacylCoAMAT[t],
    C14HydroxyacylCoAMAT[t], C12HydroxyacylCoAMAT[t], C8HydroxyacylCoAMAT[t],
    C6HydroxyacylCoAMAT[t], C4HydroxyacylCoAMAT[t], C4AcetoacylCoAMAT[t]],
  vcrotC8 → CROT[sfcrotC8, Vcrot, KmcrotC8EnoylCoAMAT, KmcrotC16EnoylCoAMAT,
    KmcrotC14EnoylCoAMAT, KmcrotC12EnoylCoAMAT, KmcrotC10EnoylCoAMAT,
    KmcrotC6EnoylCoAMAT, KmcrotC4EnoylCoAMAT, KmcrotC8HydroxyacylCoAMAT,
    KmcrotC16HydroxyacylCoAMAT, KmcrotC14HydroxyacylCoAMAT,
    KmcrotC12HydroxyacylCoAMAT, KmcrotC10HydroxyacylCoAMAT,
    KmcrotC6HydroxyacylCoAMAT, KmcrotC4HydroxyacylCoAMAT, KicrotC4AcetoacylCoA,
    Keqcrot, C8EnoylCoAMAT[t], C16EnoylCoAMAT[t], C14EnoylCoAMAT[t],
    C12EnoylCoAMAT[t], C10EnoylCoAMAT[t], C6EnoylCoAMAT[t],
    C4EnoylCoAMAT[t], C8HydroxyacylCoAMAT[t], C16HydroxyacylCoAMAT[t],
```

```
    C14HydroxyacylCoAMAT[t], C12HydroxyacylCoAMAT[t], C10HydroxyacylCoAMAT[t],
    C6HydroxyacylCoAMAT[t], C4HydroxyacylCoAMAT[t], C4AcetoacylCoAMAT[t]],
  vcrotC6 → CROT[sfcrotC6, Vcrot, KmcrotC6EnoylCoAMAT, KmcrotC16EnoylCoAMAT,
    KmcrotC14EnoylCoAMAT, KmcrotC12EnoylCoAMAT, KmcrotC10EnoylCoAMAT,
    KmcrotC8EnoylCoAMAT, KmcrotC4EnoylCoAMAT, KmcrotC6HydroxyacylCoAMAT,
    KmcrotC16HydroxyacylCoAMAT, KmcrotC14HydroxyacylCoAMAT,
    KmcrotC12HydroxyacylCoAMAT, KmcrotC10HydroxyacylCoAMAT,
    KmcrotC8HydroxyacylCoAMAT, KmcrotC4HydroxyacylCoAMAT, KicrotC4AcetoacylCoA,
    Keqcrot, C6EnoylCoAMAT[t], C16EnoylCoAMAT[t], C14EnoylCoAMAT[t],
    C12EnoylCoAMAT[t], C10EnoylCoAMAT[t], C8EnoylCoAMAT[t],
    C4EnoylCoAMAT[t], C6HydroxyacylCoAMAT[t], C16HydroxyacylCoAMAT[t],
    C14HydroxyacylCoAMAT[t], C12HydroxyacylCoAMAT[t], C10HydroxyacylCoAMAT[t],
    C8HydroxyacylCoAMAT[t], C4HydroxyacylCoAMAT[t], C4AcetoacylCoAMAT[t]],
  vcrotC4 → CROT[sfcrotC4, Vcrot, KmcrotC4EnoylCoAMAT, KmcrotC16EnoylCoAMAT,
    KmcrotC14EnoylCoAMAT, KmcrotC12EnoylCoAMAT, KmcrotC10EnoylCoAMAT,
    KmcrotC8EnoylCoAMAT, KmcrotC6EnoylCoAMAT, KmcrotC4HydroxyacylCoAMAT,
    KmcrotC16HydroxyacylCoAMAT, KmcrotC14HydroxyacylCoAMAT,
    KmcrotC12HydroxyacylCoAMAT, KmcrotC10HydroxyacylCoAMAT,
    KmcrotC8HydroxyacylCoAMAT, KmcrotC6HydroxyacylCoAMAT, KicrotC4AcetoacylCoA,
    Keqcrot, C4EnoylCoAMAT[t], C16EnoylCoAMAT[t], C14EnoylCoAMAT[t],
    C12EnoylCoAMAT[t], C10EnoylCoAMAT[t], C8EnoylCoAMAT[t],
    C6EnoylCoAMAT[t], C4HydroxyacylCoAMAT[t], C16HydroxyacylCoAMAT[t],
    C14HydroxyacylCoAMAT[t], C12HydroxyacylCoAMAT[t], C10HydroxyacylCoAMAT[t],
    C8HydroxyacylCoAMAT[t], C6HydroxyacylCoAMAT[t], C4AcetoacylCoAMAT[t]],
  vmschadC16 → MSCHAD[sfmschadC16, Vmschad, KmmschadC16HydroxyacylCoAMAT,
    KmmschadC14HydroxyacylCoAMAT, KmmschadC12HydroxyacylCoAMAT,
    KmmschadC10HydroxyacylCoAMAT, KmmschadC8HydroxyacylCoAMAT,
    KmmschadC6HydroxyacylCoAMAT, KmmschadC4HydroxyacylCoAMAT,
    KmmschadNADMAT, KmmschadC16KetoacylCoAMAT, KmmschadC14KetoacylCoAMAT,
    KmmschadC12KetoacylCoAMAT, KmmschadC10KetoacylCoAMAT, KmmschadC8KetoacylCoAMAT,
    KmmschadC6KetoacylCoAMAT, KmmschadC4AcetoacylCoAMAT, KmmschadNADHMAT,
    Keqmschad, C16HydroxyacylCoAMAT[t], C14HydroxyacylCoAMAT[t],
    C12HydroxyacylCoAMAT[t], C10HydroxyacylCoAMAT[t], C8HydroxyacylCoAMAT[t],
    C6HydroxyacylCoAMAT[t], C4HydroxyacylCoAMAT[t], NADtMAT, C16KetoacylCoAMAT[t],
    C14KetoacylCoAMAT[t], C12KetoacylCoAMAT[t], C10KetoacylCoAMAT[t],
    C8KetoacylCoAMAT[t], C6KetoacylCoAMAT[t], C4AcetoacylCoAMAT[t], NADHMAT],
  vmschadC14 → MSCHAD[sfmschadC14, Vmschad, KmmschadC14HydroxyacylCoAMAT,
    KmmschadC16HydroxyacylCoAMAT, KmmschadC12HydroxyacylCoAMAT,
    KmmschadC10HydroxyacylCoAMAT, KmmschadC8HydroxyacylCoAMAT,
    KmmschadC6HydroxyacylCoAMAT, KmmschadC4HydroxyacylCoAMAT,
    KmmschadNADMAT, KmmschadC14KetoacylCoAMAT, KmmschadC16KetoacylCoAMAT,
    KmmschadC12KetoacylCoAMAT, KmmschadC10KetoacylCoAMAT, KmmschadC8KetoacylCoAMAT,
    KmmschadC6KetoacylCoAMAT, KmmschadC4AcetoacylCoAMAT, KmmschadNADHMAT,
    Keqmschad, C14HydroxyacylCoAMAT[t], C16HydroxyacylCoAMAT[t],
    C12HydroxyacylCoAMAT[t], C10HydroxyacylCoAMAT[t], C8HydroxyacylCoAMAT[t],
    C6HydroxyacylCoAMAT[t], C4HydroxyacylCoAMAT[t], NADtMAT, C14KetoacylCoAMAT[t],
    C16KetoacylCoAMAT[t], C12KetoacylCoAMAT[t], C10KetoacylCoAMAT[t],
    C8KetoacylCoAMAT[t], C6KetoacylCoAMAT[t], C4AcetoacylCoAMAT[t], NADHMAT],
  vmschadC12 → MSCHAD[sfmschadC12, Vmschad, KmmschadC12HydroxyacylCoAMAT,
    KmmschadC16HydroxyacylCoAMAT, KmmschadC14HydroxyacylCoAMAT,
    KmmschadC10HydroxyacylCoAMAT, KmmschadC8HydroxyacylCoAMAT,
    KmmschadC6HydroxyacylCoAMAT, KmmschadC4HydroxyacylCoAMAT,
    KmmschadNADMAT, KmmschadC12KetoacylCoAMAT, KmmschadC16KetoacylCoAMAT,
    KmmschadC14KetoacylCoAMAT, KmmschadC10KetoacylCoAMAT, KmmschadC8KetoacylCoAMAT,
    KmmschadC6KetoacylCoAMAT, KmmschadC4AcetoacylCoAMAT, KmmschadNADHMAT,
```

```
    Keqmschad, C12HydroxyacylCoAMAT[t], C16HydroxyacylCoAMAT[t],
    C14HydroxyacylCoAMAT[t], C10HydroxyacylCoAMAT[t], C8HydroxyacylCoAMAT[t],
    C6HydroxyacylCoAMAT[t], C4HydroxyacylCoAMAT[t], NADtMAT, C12KetoacylCoAMAT[t],
    C16KetoacylCoAMAT[t], C14KetoacylCoAMAT[t], C10KetoacylCoAMAT[t],
    C8KetoacylCoAMAT[t], C6KetoacylCoAMAT[t], C4AcetoacylCoAMAT[t], NADHMAT],
  vmschadC10 → MSCHAD[sfmschadC10, Vmschad, KmmschadC10HydroxyacylCoAMAT,
    KmmschadC16HydroxyacylCoAMAT, KmmschadC14HydroxyacylCoAMAT,
    KmmschadC12HydroxyacylCoAMAT, KmmschadC8HydroxyacylCoAMAT,
    KmmschadC6HydroxyacylCoAMAT, KmmschadC4HydroxyacylCoAMAT,
    KmmschadNADMAT, KmmschadC10KetoacylCoAMAT, KmmschadC16KetoacylCoAMAT,
    KmmschadC14KetoacylCoAMAT, KmmschadC12KetoacylCoAMAT, KmmschadC8KetoacylCoAMAT,
    KmmschadC6KetoacylCoAMAT, KmmschadC4AcetoacylCoAMAT, KmmschadNADHMAT,
    Keqmschad, C10HydroxyacylCoAMAT[t], C16HydroxyacylCoAMAT[t],
    C14HydroxyacylCoAMAT[t], C12HydroxyacylCoAMAT[t], C8HydroxyacylCoAMAT[t],
    C6HydroxyacylCoAMAT[t], C4HydroxyacylCoAMAT[t], NADtMAT, C10KetoacylCoAMAT[t],
    C16KetoacylCoAMAT[t], C14KetoacylCoAMAT[t], C12KetoacylCoAMAT[t],
    C8KetoacylCoAMAT[t], C6KetoacylCoAMAT[t], C4AcetoacylCoAMAT[t], NADHMAT],
  vmschadC8 → MSCHAD[sfmschadC8, Vmschad, KmmschadC8HydroxyacylCoAMAT,
    KmmschadC16HydroxyacylCoAMAT, KmmschadC14HydroxyacylCoAMAT,
    KmmschadC12HydroxyacylCoAMAT, KmmschadC10HydroxyacylCoAMAT,
    KmmschadC6HydroxyacylCoAMAT, KmmschadC4HydroxyacylCoAMAT,
    KmmschadNADMAT, KmmschadC8KetoacylCoAMAT, KmmschadC16KetoacylCoAMAT,
    KmmschadC14KetoacylCoAMAT, KmmschadC12KetoacylCoAMAT, KmmschadC10KetoacylCoAMAT,
    KmmschadC6KetoacylCoAMAT, KmmschadC4AcetoacylCoAMAT, KmmschadNADHMAT,
    Keqmschad, C8HydroxyacylCoAMAT[t], C16HydroxyacylCoAMAT[t],
    C14HydroxyacylCoAMAT[t], C12HydroxyacylCoAMAT[t], C10HydroxyacylCoAMAT[t],
    C6HydroxyacylCoAMAT[t], C4HydroxyacylCoAMAT[t], NADtMAT, C8KetoacylCoAMAT[t],
    C16KetoacylCoAMAT[t], C14KetoacylCoAMAT[t], C12KetoacylCoAMAT[t],
    C10KetoacylCoAMAT[t], C6KetoacylCoAMAT[t], C4AcetoacylCoAMAT[t], NADHMAT],
  vmschadC6 → MSCHAD[sfmschadC6, Vmschad, KmmschadC6HydroxyacylCoAMAT,
    KmmschadC16HydroxyacylCoAMAT, KmmschadC14HydroxyacylCoAMAT,
    KmmschadC12HydroxyacylCoAMAT, KmmschadC10HydroxyacylCoAMAT,
    KmmschadC8HydroxyacylCoAMAT, KmmschadC4HydroxyacylCoAMAT,
    KmmschadNADMAT, KmmschadC6KetoacylCoAMAT, KmmschadC16KetoacylCoAMAT,
    KmmschadC14KetoacylCoAMAT, KmmschadC12KetoacylCoAMAT, KmmschadC10KetoacylCoAMAT,
    KmmschadC8KetoacylCoAMAT, KmmschadC4AcetoacylCoAMAT, KmmschadNADHMAT,
    Keqmschad, C6HydroxyacylCoAMAT[t], C16HydroxyacylCoAMAT[t],
    C14HydroxyacylCoAMAT[t], C12HydroxyacylCoAMAT[t], C10HydroxyacylCoAMAT[t],
    C8HydroxyacylCoAMAT[t], C4HydroxyacylCoAMAT[t], NADtMAT, C6KetoacylCoAMAT[t],
    C16KetoacylCoAMAT[t], C14KetoacylCoAMAT[t], C12KetoacylCoAMAT[t],
    C10KetoacylCoAMAT[t], C8KetoacylCoAMAT[t], C4AcetoacylCoAMAT[t], NADHMAT],
  vmschadC4 → MSCHAD[sfmschadC4, Vmschad, KmmschadC4HydroxyacylCoAMAT,
    KmmschadC16HydroxyacylCoAMAT, KmmschadC14HydroxyacylCoAMAT,
    KmmschadC12HydroxyacylCoAMAT, KmmschadC10HydroxyacylCoAMAT,
    KmmschadC8HydroxyacylCoAMAT, KmmschadC6HydroxyacylCoAMAT,
    KmmschadNADMAT, KmmschadC4AcetoacylCoAMAT, KmmschadC16KetoacylCoAMAT,
    KmmschadC14KetoacylCoAMAT, KmmschadC12KetoacylCoAMAT, KmmschadC10KetoacylCoAMAT,
    KmmschadC8KetoacylCoAMAT, KmmschadC6KetoacylCoAMAT, KmmschadNADHMAT,
    Keqmschad, C4HydroxyacylCoAMAT[t], C16HydroxyacylCoAMAT[t],
    C14HydroxyacylCoAMAT[t], C12HydroxyacylCoAMAT[t], C10HydroxyacylCoAMAT[t],
    C8HydroxyacylCoAMAT[t], C6HydroxyacylCoAMAT[t], NADtMAT, C4AcetoacylCoAMAT[t],
    C16KetoacylCoAMAT[t], C14KetoacylCoAMAT[t], C12KetoacylCoAMAT[t],
    C10KetoacylCoAMAT[t], C8KetoacylCoAMAT[t], C6KetoacylCoAMAT[t], NADHMAT],
  vmckatC16 → MCKATA[sfmckatC16, Vmckat, KmmckatC16KetoacylCoAMAT,
    KmmckatC14KetoacylCoAMAT, KmmckatC12KetoacylCoAMAT,
```

```
    KmmckatC10KetoacylCoAMAT, KmmckatC8KetoacylCoAMAT, KmmckatC6KetoacylCoAMAT,
    KmmckatC4AcetoacylCoAMAT, KmmckatCoAMAT, KmmckatC14AcylCoAMAT,
    KmmckatC16AcylCoAMAT, KmmckatC12AcylCoAMAT, KmmckatC10AcylCoAMAT,
    KmmckatC8AcylCoAMAT, KmmckatC6AcylCoAMAT, KmmckatC4AcylCoAMAT,
    KmmckatAcetylCoAMAT, Keqmckat, C16KetoacylCoAMAT[t], C14KetoacylCoAMAT[t],
    C12KetoacylCoAMAT[t], C10KetoacylCoAMAT[t], C8KetoacylCoAMAT[t],
    C6KetoacylCoAMAT[t], C4AcetoacylCoAMAT[t], CoAMAT, C14AcylCoAMAT[t],
    C16AcylCoAMAT[t], C12AcylCoAMAT[t], C10AcylCoAMAT[t], C8AcylCoAMAT[t],
    C6AcylCoAMAT[t], C4AcylCoAMAT[t], AcetylCoAMAT, CE1, KmCE1, nE1],
  vmckatC14 → MCKATA[sfmckatC14, Vmckat, KmmckatC14KetoacylCoAMAT,
    KmmckatC16KetoacylCoAMAT, KmmckatC12KetoacylCoAMAT,
    KmmckatC10KetoacylCoAMAT, KmmckatC8KetoacylCoAMAT, KmmckatC6KetoacylCoAMAT,
    KmmckatC4AcetoacylCoAMAT, KmmckatCoAMAT, KmmckatC12AcylCoAMAT,
    KmmckatC16AcylCoAMAT, KmmckatC14AcylCoAMAT, KmmckatC10AcylCoAMAT,
    KmmckatC8AcylCoAMAT, KmmckatC6AcylCoAMAT, KmmckatC4AcylCoAMAT,
    KmmckatAcetylCoAMAT, Keqmckat, C14KetoacylCoAMAT[t], C16KetoacylCoAMAT[t],
    C12KetoacylCoAMAT[t], C10KetoacylCoAMAT[t], C8KetoacylCoAMAT[t],
    C6KetoacylCoAMAT[t], C4AcetoacylCoAMAT[t], CoAMAT, C12AcylCoAMAT[t],
    C16AcylCoAMAT[t], C14AcylCoAMAT[t], C10AcylCoAMAT[t], C8AcylCoAMAT[t],
    C6AcylCoAMAT[t], C4AcylCoAMAT[t], AcetylCoAMAT, CE1, KmCE1, nE1],
  vmckatC12 → MCKATA[sfmckatC12, Vmckat, KmmckatC12KetoacylCoAMAT,
    KmmckatC16KetoacylCoAMAT, KmmckatC14KetoacylCoAMAT,
    KmmckatC10KetoacylCoAMAT, KmmckatC8KetoacylCoAMAT, KmmckatC6KetoacylCoAMAT,
    KmmckatC4AcetoacylCoAMAT, KmmckatCoAMAT, KmmckatC10AcylCoAMAT,
    KmmckatC16AcylCoAMAT, KmmckatC14AcylCoAMAT, KmmckatC12AcylCoAMAT,
    KmmckatC8AcylCoAMAT, KmmckatC6AcylCoAMAT, KmmckatC4AcylCoAMAT,
    KmmckatAcetylCoAMAT, Keqmckat, C12KetoacylCoAMAT[t], C16KetoacylCoAMAT[t],
    C14KetoacylCoAMAT[t], C10KetoacylCoAMAT[t], C8KetoacylCoAMAT[t],
    C6KetoacylCoAMAT[t], C4AcetoacylCoAMAT[t], CoAMAT, C10AcylCoAMAT[t],
    C16AcylCoAMAT[t], C14AcylCoAMAT[t], C12AcylCoAMAT[t], C8AcylCoAMAT[t],
    C6AcylCoAMAT[t], C4AcylCoAMAT[t], AcetylCoAMAT, CE1, KmCE1, nE1],
  vmckatC10 → MCKATA[sfmckatC10, Vmckat, KmmckatC10KetoacylCoAMAT,
    KmmckatC16KetoacylCoAMAT, KmmckatC14KetoacylCoAMAT,
    KmmckatC12KetoacylCoAMAT, KmmckatC8KetoacylCoAMAT, KmmckatC6KetoacylCoAMAT,
    KmmckatC4AcetoacylCoAMAT, KmmckatCoAMAT, KmmckatC8AcylCoAMAT,
    KmmckatC16AcylCoAMAT, KmmckatC14AcylCoAMAT, KmmckatC12AcylCoAMAT,
    KmmckatC10AcylCoAMAT, KmmckatC6AcylCoAMAT, KmmckatC4AcylCoAMAT,
    KmmckatAcetylCoAMAT, Keqmckat, C10KetoacylCoAMAT[t], C16KetoacylCoAMAT[t],
    C14KetoacylCoAMAT[t], C12KetoacylCoAMAT[t], C8KetoacylCoAMAT[t],
    C6KetoacylCoAMAT[t], C4AcetoacylCoAMAT[t], CoAMAT, C8AcylCoAMAT[t],
    C16AcylCoAMAT[t], C14AcylCoAMAT[t], C12AcylCoAMAT[t], C10AcylCoAMAT[t],
    C6AcylCoAMAT[t], C4AcylCoAMAT[t], AcetylCoAMAT, CE1, KmCE1, nE1],
  vmckatC8 → MCKATA[sfmckatC8, Vmckat, KmmckatC8KetoacylCoAMAT,
    KmmckatC16KetoacylCoAMAT, KmmckatC14KetoacylCoAMAT,
    KmmckatC12KetoacylCoAMAT, KmmckatC10KetoacylCoAMAT, KmmckatC6KetoacylCoAMAT,
    KmmckatC4AcetoacylCoAMAT, KmmckatCoAMAT, KmmckatC6AcylCoAMAT,
    KmmckatC16AcylCoAMAT, KmmckatC14AcylCoAMAT, KmmckatC12AcylCoAMAT,
    KmmckatC10AcylCoAMAT, KmmckatC8AcylCoAMAT, KmmckatC4AcylCoAMAT,
    KmmckatAcetylCoAMAT, Keqmckat, C8KetoacylCoAMAT[t], C16KetoacylCoAMAT[t],
    C14KetoacylCoAMAT[t], C12KetoacylCoAMAT[t], C10KetoacylCoAMAT[t],
    C6KetoacylCoAMAT[t], C4AcetoacylCoAMAT[t], CoAMAT, C6AcylCoAMAT[t],
    C16AcylCoAMAT[t], C14AcylCoAMAT[t], C12AcylCoAMAT[t], C10AcylCoAMAT[t],
    C8AcylCoAMAT[t], C4AcylCoAMAT[t], AcetylCoAMAT, CE1, KmCE1, nE1],
  vmckatC6 → MCKATA[sfmckatC6, Vmckat, KmmckatC6KetoacylCoAMAT,
    KmmckatC16KetoacylCoAMAT, KmmckatC14KetoacylCoAMAT,
```

```
    KmmckatC12KetoacylCoAMAT, KmmckatC10KetoacylCoAMAT, KmmckatC8KetoacylCoAMAT,
    KmmckatC4AcetoacylCoAMAT, KmmckatCoAMAT, KmmckatC4AcylCoAMAT,
    KmmckatC16AcylCoAMAT, KmmckatC14AcylCoAMAT, KmmckatC12AcylCoAMAT,
    KmmckatC10AcylCoAMAT, KmmckatC8AcylCoAMAT, KmmckatC6AcylCoAMAT,
    KmmckatAcetylCoAMAT, Keqmckat, C6KetoacylCoAMAT[t], C16KetoacylCoAMAT[t],
    C14KetoacylCoAMAT[t], C12KetoacylCoAMAT[t], C10KetoacylCoAMAT[t],
    C8KetoacylCoAMAT[t], C4AcetoacylCoAMAT[t], CoAMAT, C4AcylCoAMAT[t],
    C16AcylCoAMAT[t], C14AcylCoAMAT[t], C12AcylCoAMAT[t], C10AcylCoAMAT[t],
    C8AcylCoAMAT[t], C6AcylCoAMAT[t], AcetylCoAMAT, CE1, KmCE1, nE1],
  vmckatC4 → MCKATB[sfmckatC4, Vmckat, KmmckatC4AcetoacylCoAMAT,
    KmmckatC16KetoacylCoAMAT, KmmckatC14KetoacylCoAMAT,
    KmmckatC12KetoacylCoAMAT, KmmckatC10KetoacylCoAMAT, KmmckatC8KetoacylCoAMAT,
    KmmckatC6KetoacylCoAMAT, KmmckatCoAMAT, KmmckatC4AcylCoAMAT,
    KmmckatC16AcylCoAMAT, KmmckatC14AcylCoAMAT, KmmckatC12AcylCoAMAT,
    KmmckatC10AcylCoAMAT, KmmckatC8AcylCoAMAT, KmmckatC6AcylCoAMAT,
    KmmckatAcetylCoAMAT, Keqmckat, C4AcetoacylCoAMAT[t], C16KetoacylCoAMAT[t],
    C14KetoacylCoAMAT[t], C12KetoacylCoAMAT[t], C10KetoacylCoAMAT[t],
    C8KetoacylCoAMAT[t], C6KetoacylCoAMAT[t], CoAMAT, C4AcylCoAMAT[t],
    C16AcylCoAMAT[t], C14AcylCoAMAT[t], C12AcylCoAMAT[t], C10AcylCoAMAT[t],
    C8AcylCoAMAT[t], C6AcylCoAMAT[t], AcetylCoAMAT, CE1, KmCE1, nE1],
  vmtpC16 → MTP[sfmtpC16, Vmtp, KmmtpC16EnoylCoAMAT, KmmtpC14EnoylCoAMAT,
    KmmtpC12EnoylCoAMAT, KmmtpC10EnoylCoAMAT, KmmtpC8EnoylCoAMAT,
    KmmtpNADMAT, KmmtpCoAMAT, KmmtpC14AcylCoAMAT, KmmtpC16AcylCoAMAT,
    KmmtpC12AcylCoAMAT, KmmtpC10AcylCoAMAT, KmmtpC8AcylCoAMAT,
    KmmtpC6AcylCoAMAT, KmmtpNADHMAT, KmmtpAcetylCoAMAT, KicrotC4AcetoacylCoA,
    Keqmtp, C16EnoylCoAMAT[t], C14EnoylCoAMAT[t], C12EnoylCoAMAT[t],
    C10EnoylCoAMAT[t], C8EnoylCoAMAT[t], NADtMAT, CoAMAT, C14AcylCoAMAT[t],
    C16AcylCoAMAT[t], C12AcylCoAMAT[t], C10AcylCoAMAT[t], C8AcylCoAMAT[t],
    C6AcylCoAMAT[t], NADHMAT, AcetylCoAMAT, C4AcetoacylCoAMAT[t]],
  vmtpC14 → MTP[sfmtpC14, Vmtp, KmmtpC14EnoylCoAMAT, KmmtpC16EnoylCoAMAT,
    KmmtpC12EnoylCoAMAT, KmmtpC10EnoylCoAMAT, KmmtpC8EnoylCoAMAT,
    KmmtpNADMAT, KmmtpCoAMAT, KmmtpC12AcylCoAMAT, KmmtpC16AcylCoAMAT,
    KmmtpC14AcylCoAMAT, KmmtpC10AcylCoAMAT, KmmtpC8AcylCoAMAT,
    KmmtpC6AcylCoAMAT, KmmtpNADHMAT, KmmtpAcetylCoAMAT, KicrotC4AcetoacylCoA,
    Keqmtp, C14EnoylCoAMAT[t], C16EnoylCoAMAT[t], C12EnoylCoAMAT[t],
    C10EnoylCoAMAT[t], C8EnoylCoAMAT[t], NADtMAT, CoAMAT, C12AcylCoAMAT[t],
    C16AcylCoAMAT[t], C14AcylCoAMAT[t], C10AcylCoAMAT[t], C8AcylCoAMAT[t],
    C6AcylCoAMAT[t], NADHMAT, AcetylCoAMAT, C4AcetoacylCoAMAT[t]],
  vmtpC12 → MTP[sfmtpC12, Vmtp, KmmtpC12EnoylCoAMAT, KmmtpC16EnoylCoAMAT,
    KmmtpC14EnoylCoAMAT, KmmtpC10EnoylCoAMAT, KmmtpC8EnoylCoAMAT,
    KmmtpNADMAT, KmmtpCoAMAT, KmmtpC10AcylCoAMAT, KmmtpC16AcylCoAMAT,
    KmmtpC14AcylCoAMAT, KmmtpC12AcylCoAMAT, KmmtpC8AcylCoAMAT,
    KmmtpC6AcylCoAMAT, KmmtpNADHMAT, KmmtpAcetylCoAMAT, KicrotC4AcetoacylCoA,
    Keqmtp, C12EnoylCoAMAT[t], C16EnoylCoAMAT[t], C14EnoylCoAMAT[t],
    C10EnoylCoAMAT[t], C8EnoylCoAMAT[t], NADtMAT, CoAMAT, C10AcylCoAMAT[t],
    C16AcylCoAMAT[t], C14AcylCoAMAT[t], C12AcylCoAMAT[t], C8AcylCoAMAT[t],
    C6AcylCoAMAT[t], NADHMAT, AcetylCoAMAT, C4AcetoacylCoAMAT[t]],
  vmtpC10 → MTP[sfmtpC10, Vmtp, KmmtpC10EnoylCoAMAT, KmmtpC16EnoylCoAMAT,
    KmmtpC14EnoylCoAMAT, KmmtpC12EnoylCoAMAT, KmmtpC8EnoylCoAMAT,
    KmmtpNADMAT, KmmtpCoAMAT, KmmtpC8AcylCoAMAT, KmmtpC16AcylCoAMAT,
    KmmtpC14AcylCoAMAT, KmmtpC12AcylCoAMAT, KmmtpC10AcylCoAMAT,
    KmmtpC6AcylCoAMAT, KmmtpNADHMAT, KmmtpAcetylCoAMAT, KicrotC4AcetoacylCoA,
    Keqmtp, C10EnoylCoAMAT[t], C16EnoylCoAMAT[t], C14EnoylCoAMAT[t],
    C12EnoylCoAMAT[t], C8EnoylCoAMAT[t], NADtMAT, CoAMAT, C8AcylCoAMAT[t],
    C16AcylCoAMAT[t], C14AcylCoAMAT[t], C12AcylCoAMAT[t], C10AcylCoAMAT[t],
```

```
      C6AcylCoAMAT[t], NADHMAT, AcetylCoAMAT, C4AcetoacylCoAMAT[t]],
    vmtpC8 → MTP[sfmtpC8, Vmtp, KmmtpC8EnoylCoAMAT, KmmtpC16EnoylCoAMAT,
      KmmtpC14EnoylCoAMAT, KmmtpC12EnoylCoAMAT, KmmtpC10EnoylCoAMAT,
      KmmtpNADMAT, KmmtpCoAMAT, KmmtpC6AcylCoAMAT, KmmtpC16AcylCoAMAT,
      KmmtpC14AcylCoAMAT, KmmtpC12AcylCoAMAT, KmmtpC10AcylCoAMAT,
      KmmtpC8AcylCoAMAT, KmmtpNADHMAT, KmmtpAcetylCoAMAT, KicrotC4AcetoacylCoA,
      Keqmtp, C8EnoylCoAMAT[t], C16EnoylCoAMAT[t], C14EnoylCoAMAT[t],
      C12EnoylCoAMAT[t], C10EnoylCoAMAT[t], NADtMAT, CoAMAT, C6AcylCoAMAT[t],
      C16AcylCoAMAT[t], C14AcylCoAMAT[t], C12AcylCoAMAT[t], C10AcylCoAMAT[t],
      C8AcylCoAMAT[t], NADHMAT, AcetylCoAMAT, C4AcetoacylCoAMAT[t]],
    vacesink → RES[Ksacesink, AcetylCoAMAT, K1acesink],
    vfadhsink → RES[Ksfadhsink, FADHMAT, K1fadhsink],
    vnadhsink → RES[Ksnadhsink, NADHMAT, K1nadhsink]};

CoAMATX =
   {CoAMAT → CoAMATt - C16AcylCoAMAT[t] - C16EnoylCoAMAT[t] - C16HydroxyacylCoAMAT[t] -
      C16KetoacylCoAMAT[t] - C14AcylCoAMAT[t] - C14EnoylCoAMAT[t] -
      C14HydroxyacylCoAMAT[t] - C14KetoacylCoAMAT[t] - C12AcylCoAMAT[t] -
      C12EnoylCoAMAT[t] - C12HydroxyacylCoAMAT[t] - C12KetoacylCoAMAT[t] -
      C10AcylCoAMAT[t] - C10EnoylCoAMAT[t] - C10HydroxyacylCoAMAT[t] -
      C10KetoacylCoAMAT[t] - C8AcylCoAMAT[t] - C8EnoylCoAMAT[t] -
      C8HydroxyacylCoAMAT[t] - C8KetoacylCoAMAT[t] - C6AcylCoAMAT[t] - C6EnoylCoAMAT[t] -
      C6HydroxyacylCoAMAT[t] - C6KetoacylCoAMAT[t] - C4AcylCoAMAT[t] -
      C4EnoylCoAMAT[t] - C4HydroxyacylCoAMAT[t] - C4AcetoacylCoAMAT[t] - AcetylCoAMAT};

Parm = {
    sfcpt1C16 → 1, Vcpt1 → 0.012, Kmcpt1C16AcylCoACYT → 13.8,
    Kmcpt1CarCYT → 250, Kmcpt1C16AcylCarCYT → 136, Kmcpt1CoACYT → 40.7,
    Kicpt1MalCoACYT → 9.1, Keqcpt1 → 0.45, ncpt1 → 2.4799,
    Vfcact → 0.42, Vrcact → 0.42, KmcactC16AcylCarCYT → 15,
    KmcactC14AcylCarCYT → 15, KmcactC12AcylCarCYT → 15, KmcactC10AcylCarCYT → 15,
    KmcactC8AcylCarCYT → 15, KmcactC6AcylCarCYT → 15, KmcactC4AcylCarCYT → 15,
    KmcactCarMAT → 130, KmcactC16AcylCarMAT → 15, KmcactC14AcylCarMAT → 15,
    KmcactC12AcylCarMAT → 15, KmcactC10AcylCarMAT → 15, KmcactC8AcylCarMAT → 15,
    KmcactC6AcylCarMAT → 15, KmcactC4AcylCarMAT → 15, KmcactCarCYT → 130,
    KicactC16AcylCarCYT → 56, KicactC14AcylCarCYT → 56, KicactC12AcylCarCYT → 56,
    KicactC10AcylCarCYT → 56, KicactC8AcylCarCYT → 56, KicactC6AcylCarCYT → 56,
    KicactC4AcylCarCYT → 56, KicactCarCYT → 200, Keqcact → 1,
    sfcpt2C16 → 0.85, sfcpt2C14 → 1, sfcpt2C12 → 0.95, sfcpt2C10 → 0.95,
    sfcpt2C8 → 0.35, sfcpt2C6 → 0.15, sfcpt2C4 → 0.01, Vcpt2 → 0.391,
    Kmcpt2C16AcylCarMAT → 51, Kmcpt2C14AcylCarMAT → 51, Kmcpt2C12AcylCarMAT → 51,
    Kmcpt2C10AcylCarMAT → 51, Kmcpt2C8AcylCarMAT → 51, Kmcpt2C6AcylCarMAT → 51,
    Kmcpt2C4AcylCarMAT → 51, Kmcpt2CoAMAT → 30, Kmcpt2C16AcylCoAMAT → 38,
    Kmcpt2C14AcylCoAMAT → 38, Kmcpt2C12AcylCoAMAT → 38,
    Kmcpt2C10AcylCoAMAT → 38, Kmcpt2C8AcylCoAMAT → 38, Kmcpt2C6AcylCoAMAT → 1000,
    Kmcpt2C4AcylCoAMAT → 1 000 000, Kmcpt2CarMAT → 350, Keqcpt2 → 2.22,
    sfvlcadC16 → 1, sfvlcadC14 → 0.42, sfvlcadC12 → 0.11, Vvlcad → 0.008,
    KmvlcadC16AcylCoAMAT → 6.5, KmvlcadC14AcylCoAMAT → 4, KmvlcadC12AcylCoAMAT → 2.7,
    KmvlcadFAD → 0.12, KmvlcadC16EnoylCoAMAT → 1.08, KmvlcadC14EnoylCoAMAT → 1.08,
    KmvlcadC12EnoylCoAMAT → 1.08, KmvlcadFADH → 24.2, Keqvlcad → 6,
    sflcadC16 → 0.9, sflcadC14 → 1, sflcadC12 → 0.9, sflcadC10 → 0.75, sflcadC8 → 0.4,
    Vlcad → 0.01, KmlcadC16AcylCoAMAT → 2.5, KmlcadC14AcylCoAMAT → 7.4,
    KmlcadC12AcylCoAMAT → 9, KmlcadC10AcylCoAMAT → 24.3, KmlcadC8AcylCoAMAT → 123,
    KmlcadFAD → 0.12, KmlcadC16EnoylCoAMAT → 1.08, KmlcadC14EnoylCoAMAT → 1.08,
    KmlcadC12EnoylCoAMAT → 1.08, KmlcadC10EnoylCoAMAT → 1.08,
```

```
KmlcadC8EnoylCoAMAT → 1.08, KmlcadFADH → 24.2, Keqlcad → 6,
sfmcadC12 → 0.38, sfmcadC10 → 0.8, sfmcadC8 → 0.87, sfmcadC6 → 1, sfmcadC4 → 0.12,
Vmcad → 0.081, KmmcadC12AcylCoAMAT → 5.7, KmmcadC10AcylCoAMAT → 5.4,
KmmcadC8AcylCoAMAT → 4, KmmcadC6AcylCoAMAT → 9.4, KmmcadC4AcylCoAMAT → 135,
KmmcadFAD → 0.12, KmmcadC12EnoylCoAMAT → 1.08, KmmcadC10EnoylCoAMAT → 1.08,
KmmcadC8EnoylCoAMAT → 1.08, KmmcadC6EnoylCoAMAT → 1.08,
KmmcadC4EnoylCoAMAT → 1.08, KmmcadFADH → 24.2, Keqmcad → 6,
sfscadC6 → 0.3, sfscadC4 → 1, Vscad → 0.081, KmscadC6AcylCoAMAT → 285,
KmscadC4AcylCoAMAT → 10.7, KmscadFAD → 0.12, KmscadC6EnoylCoAMAT → 1.08,
KmscadC4EnoylCoAMAT → 1.08, KmscadFADH → 24.2, Keqscad -> 6,
sfcrotC16 → 0.13, sfcrotC14 → 0.2, sfcrotC12 → 0.25, sfcrotC10 → 0.33, sfcrotC8 → 0.58,
sfcrotC6 → 0.83, sfcrotC4 → 1, Vcrot → 3.6, KmcrotC16EnoylCoAMAT → 150,
KmcrotC14EnoylCoAMAT → 100, KmcrotC12EnoylCoAMAT → 25, KmcrotC10EnoylCoAMAT → 25,
KmcrotC8EnoylCoAMAT → 25, KmcrotC6EnoylCoAMAT → 25, KmcrotC4EnoylCoAMAT → 40,
KmcrotC16HydroxyacylCoAMAT → 45, KmcrotC14HydroxyacylCoAMAT → 45,
KmcrotC12HydroxyacylCoAMAT → 45, KmcrotC10HydroxyacylCoAMAT → 45,
KmcrotC8HydroxyacylCoAMAT → 45, KmcrotC6HydroxyacylCoAMAT → 45,
KmcrotC4HydroxyacylCoAMAT → 45, KicrotC4AcetoacylCoA → 1.6, Keqcrot → 3.13,
sfmschadC16 → 0.6, sfmschadC14 → 0.5, sfmschadC12 → 0.43, sfmschadC10 → 0.64,
sfmschadC8 → 0.89, sfmschadC6 → 1, sfmschadC4 → 0.67, Vmschad → 1,
KmmschadC16HydroxyacylCoAMAT → 1.5, KmmschadC14HydroxyacylCoAMAT → 1.8,
KmmschadC12HydroxyacylCoAMAT → 3.7, KmmschadC10HydroxyacylCoAMAT → 8.8,
KmmschadC8HydroxyacylCoAMAT → 16.3, KmmschadC6HydroxyacylCoAMAT → 28.6,
KmmschadC4HydroxyacylCoAMAT → 69.9, KmmschadNADMAT → 58.5,
KmmschadC16KetoacylCoAMAT → 1.4, KmmschadC14KetoacylCoAMAT → 1.4,
KmmschadC12KetoacylCoAMAT → 1.6, KmmschadC10KetoacylCoAMAT → 2.3,
KmmschadC8KetoacylCoAMAT → 4.1, KmmschadC6KetoacylCoAMAT → 5.8,
KmmschadC4AcetoacylCoAMAT → 16.9, KmmschadNADHMAT → 5.4, Keqmschad → 2.17 * 10^-4,
sfmckatC16 → 0, sfmckatC14 → 0.2, sfmckatC12 → 0.38, sfmckatC10 → 0.65,
sfmckatC8 → 0.81, sfmckatC6 → 1, sfmckatC4 → 0.49, Vmckat → 0.377,
KmmckatC16KetoacylCoAMAT → 1.1, KmmckatC14KetoacylCoAMAT → 1.2,
KmmckatC12KetoacylCoAMAT → 1.3, KmmckatC10KetoacylCoAMAT → 2.1,
KmmckatC8KetoacylCoAMAT → 3.2, KmmckatC6KetoacylCoAMAT → 6.7,
KmmckatC4AcetoacylCoAMAT → 12.4, KmmckatCoAMAT → 26.6,
KmmckatC14AcylCoAMAT → 13.83, KmmckatC16AcylCoAMAT → 13.83,
KmmckatC12AcylCoAMAT → 13.83, KmmckatC10AcylCoAMAT → 13.83,
KmmckatC8AcylCoAMAT → 13.83, KmmckatC6AcylCoAMAT → 13.83,
KmmckatC4AcylCoAMAT → 13.83, KmmckatAcetylCoAMAT → 30, Keqmckat → 1051,
sfmtpC16 → 1, sfmtpC14 → 0.9, sfmtpC12 → 0.81, sfmtpC10 → 0.73, sfmtpC8 → 0.34,
Vmtp → 2.84, KmmtpC16EnoylCoAMAT → 25, KmmtpC14EnoylCoAMAT → 25,
KmmtpC12EnoylCoAMAT → 25, KmmtpC10EnoylCoAMAT → 25, KmmtpC8EnoylCoAMAT → 25,
KmmtpNADMAT → 60, KmmtpCoAMAT → 30, KmmtpC14AcylCoAMAT → 13.83,
KmmtpC16AcylCoAMAT → 13.83, KmmtpC12AcylCoAMAT → 13.83,
KmmtpC10AcylCoAMAT → 13.83, KmmtpC8AcylCoAMAT → 13.83, KmmtpC6AcylCoAMAT → 13.83,
KmmtpNADHMAT → 50, KmmtpAcetylCoAMAT → 30, Keqmtp → 0.71,
Ksacesink → 6 000 000, K1acesink → 70, Ksfadhsink → 6 000 000,
K1fadhsink → 0.46, Ksnadhsink → 6 000 000, K1nadhsink → 12,
C16AcylCoACYT → 25, CarCYT → 200, CoACYT → 140, MalCoACYT → 0,
CarMAT -> 950, FADtMAT → 0.77, NADtMAT → 250, CoAMATt → 5000,
VCYT → 2.2 * 10^-6, VMAT → 1.8 * 10^-6, AcetylCoAMAT → 70, FADHMAT → 0.46,
NADHMAT → 12, CE1 → 0.2 , KmCE1 → 0.449 , nE1 → 1};


InitialConditions = {
  C16AcylCarCYT[0] == 0, C16AcylCarMAT[0] == 0, C16AcylCoAMAT[0] == 0,
```

```
    C16EnoylCoAMAT[0] == 0, C16HydroxyacylCoAMAT[0] == 0, C16KetoacylCoAMAT[0] == 0,
    C14AcylCarCYT[0] == 0, C14AcylCarMAT[0] == 0, C14AcylCoAMAT[0] == 0,
    C14EnoylCoAMAT[0] == 0, C14HydroxyacylCoAMAT[0] == 0, C14KetoacylCoAMAT[0] == 0,
    C12AcylCarCYT[0] == 0, C12AcylCarMAT[0] == 0, C12AcylCoAMAT[0] == 0,
    C12EnoylCoAMAT[0] == 0, C12HydroxyacylCoAMAT[0] == 0, C12KetoacylCoAMAT[0] == 0,
    C10AcylCarCYT[0] == 0, C10AcylCarMAT[0] == 0, C10AcylCoAMAT[0] == 0,
    C10EnoylCoAMAT[0] == 0, C10HydroxyacylCoAMAT[0] == 0, C10KetoacylCoAMAT[0] == 0,
    C8AcylCarCYT[0] == 0, C8AcylCarMAT[0] == 0, C8AcylCoAMAT[0] == 0,
    C8EnoylCoAMAT[0] == 0, C8HydroxyacylCoAMAT[0] == 0, C8KetoacylCoAMAT[0] == 0,
    C6AcylCarCYT[0] == 0, C6AcylCarMAT[0] == 0, C6AcylCoAMAT[0] == 0,
    C6EnoylCoAMAT[0] == 0, C6HydroxyacylCoAMAT[0] == 0, C6KetoacylCoAMAT[0] == 0,
    C4AcylCarCYT[0] == 0, C4AcylCarMAT[0] == 0, C4AcylCoAMAT[0] == 0,
    C4EnoylCoAMAT[0] == 0, C4HydroxyacylCoAMAT[0] == 0, C4AcetoacylCoAMAT[0] == 0};

Vars = {
    C16AcylCarCYT, C16AcylCarMAT, C16AcylCoAMAT,
    C16EnoylCoAMAT, C16HydroxyacylCoAMAT, C16KetoacylCoAMAT,
    C14AcylCarCYT, C14AcylCarMAT, C14AcylCoAMAT, C14EnoylCoAMAT,
    C14HydroxyacylCoAMAT, C14KetoacylCoAMAT,
    C12AcylCarCYT, C12AcylCarMAT, C12AcylCoAMAT, C12EnoylCoAMAT,
    C12HydroxyacylCoAMAT, C12KetoacylCoAMAT,
    C10AcylCarCYT, C10AcylCarMAT, C10AcylCoAMAT, C10EnoylCoAMAT,
    C10HydroxyacylCoAMAT, C10KetoacylCoAMAT,
    C8AcylCarCYT, C8AcylCarMAT, C8AcylCoAMAT, C8EnoylCoAMAT,
    C8HydroxyacylCoAMAT, C8KetoacylCoAMAT,
    C6AcylCarCYT, C6AcylCarMAT, C6AcylCoAMAT, C6EnoylCoAMAT,
    C6HydroxyacylCoAMAT, C6KetoacylCoAMAT,
    C4AcylCarCYT, C4AcylCarMAT, C4AcylCoAMAT, C4EnoylCoAMAT,
    C4HydroxyacylCoAMAT, C4AcetoacylCoAMAT};
```

```
In[•]:= TableForm[Odes];
TableForm[RateEqs];
TableForm[Odes /. RateEqs /. CoAMATX /. Parm ];
TableForm[RateEqs /. Parm ];
TableForm[InitialConditions];
```

```
In[•]:=
tsol = NDSolve[Join[Odes /. RateEqs /. CoAMATX /. Parm , InitialConditions],
    Vars, {t, 0, 1000000000}];
```

In[•]:=
```
Table[{Vars[[i]][t], (Vars[[i]][900000000] /. tsol)[[1]]}, {i, 1, Length[Vars]}]
```

Out[•]=
```
{{C16AcylCarCYT[t], 0.166114}, {C16AcylCarMAT[t], 0.347035},
 {C16AcylCoAMAT[t], 0.85001}, {C16EnoylCoAMAT[t], 0.0459782},
 {C16HydroxyacylCoAMAT[t], 0.143912}, {C16KetoacylCoAMAT[t], 0.000619373},
 {C14AcylCarCYT[t], 0.0362455}, {C14AcylCarMAT[t], 0.172166},
 {C14AcylCoAMAT[t], 1.8854}, {C14EnoylCoAMAT[t], 0.0513172},
 {C14HydroxyacylCoAMAT[t], 0.145524}, {C14KetoacylCoAMAT[t], 0.000625653},
 {C12AcylCarCYT[t], 0.0488349}, {C12AcylCarMAT[t], 0.231966},
 {C12AcylCoAMAT[t], 2.54027}, {C12EnoylCoAMAT[t], 0.0584068},
 {C12HydroxyacylCoAMAT[t], 0.176577}, {C12KetoacylCoAMAT[t], 0.000756706},
 {C10AcylCarCYT[t], 0.0846054}, {C10AcylCarMAT[t], 0.401876},
 {C10AcylCoAMAT[t], 4.40097}, {C10EnoylCoAMAT[t], 0.0641998},
 {C10HydroxyacylCoAMAT[t], 0.195382}, {C10KetoacylCoAMAT[t], 0.000834768},
 {C8AcylCarCYT[t], 0.0848497}, {C8AcylCarMAT[t], 0.403036},
 {C8AcylCoAMAT[t], 4.41368}, {C8EnoylCoAMAT[t], 0.138973},
 {C8HydroxyacylCoAMAT[t], 0.429392}, {C8KetoacylCoAMAT[t], 0.00183363},
 {C6AcylCarCYT[t], 0.224354}, {C6AcylCarMAT[t], 1.06568}, {C6AcylCoAMAT[t], 11.6704},
 {C6EnoylCoAMAT[t], 10.0164}, {C6HydroxyacylCoAMAT[t], 31.1679},
 {C6KetoacylCoAMAT[t], 0.133085}, {C4AcylCarCYT[t], 0.371765},
 {C4AcylCarMAT[t], 1.76588}, {C4AcylCoAMAT[t], 19.3383}, {C4EnoylCoAMAT[t], 37.6754},
 {C4HydroxyacylCoAMAT[t], 117.68}, {C4AcetoacylCoAMAT[t], 0.502623}}
```

Steady state computation with varying palmitoyl-CoA (X) and p46Shc KO and control (Z)

In[•]:=
```
ParmScan[X_, Z_] := {
   sfcpt1C16 → 1, Vcpt1 → 0.012, Kmcpt1C16AcylCoACYT → 13.8,
   Kmcpt1CarCYT → 250, Kmcpt1C16AcylCarCYT → 136, Kmcpt1CoACYT → 40.7,
   Kicpt1MalCoACYT → 9.1, Keqcpt1 → 0.45, ncpt1 → 2.4799,
   Vfcact → 0.42, Vrcact → 0.42, KmcactC16AcylCarCYT → 15,
   KmcactC14AcylCarCYT → 15, KmcactC12AcylCarCYT → 15, KmcactC10AcylCarCYT → 15,
   KmcactC8AcylCarCYT → 15, KmcactC6AcylCarCYT → 15, KmcactC4AcylCarCYT → 15,
   KmcactCarMAT → 130, KmcactC16AcylCarMAT → 15, KmcactC14AcylCarMAT → 15,
   KmcactC12AcylCarMAT → 15, KmcactC10AcylCarMAT → 15, KmcactC8AcylCarMAT → 15,
   KmcactC6AcylCarMAT → 15, KmcactC4AcylCarMAT → 15, KmcactCarCYT → 130,
   KicactC16AcylCarCYT → 56, KicactC14AcylCarCYT → 56, KicactC12AcylCarCYT → 56,
   KicactC10AcylCarCYT → 56, KicactC8AcylCarCYT → 56, KicactC6AcylCarCYT → 56,
   KicactC4AcylCarCYT → 56, KicactCarCYT → 200, Keqcact → 1,
   sfcpt2C16 → 0.85, sfcpt2C14 → 1, sfcpt2C12 → 0.95, sfcpt2C10 → 0.95,
   sfcpt2C8 → 0.35, sfcpt2C6 → 0.15, sfcpt2C4 → 0.01, Vcpt2 → 0.391,
   Kmcpt2C16AcylCarMAT → 51, Kmcpt2C14AcylCarMAT → 51, Kmcpt2C12AcylCarMAT → 51,
   Kmcpt2C10AcylCarMAT → 51, Kmcpt2C8AcylCarMAT → 51, Kmcpt2C6AcylCarMAT → 51,
   Kmcpt2C4AcylCarMAT → 51, Kmcpt2CoAMAT → 30, Kmcpt2C16AcylCoAMAT → 38,
   Kmcpt2C14AcylCoAMAT → 38, Kmcpt2C12AcylCoAMAT → 38,
   Kmcpt2C10AcylCoAMAT → 38, Kmcpt2C8AcylCoAMAT → 38, Kmcpt2C6AcylCoAMAT → 1000,
   Kmcpt2C4AcylCoAMAT → 1000000, Kmcpt2CarMAT → 350, Keqcpt2 → 2.22,
   sfvlcadC16 → 1, sfvlcadC14 → 0.42, sfvlcadC12 → 0.11, Vvlcad → 0.008,
   KmvlcadC16AcylCoAMAT → 6.5, KmvlcadC14AcylCoAMAT → 4, KmvlcadC12AcylCoAMAT → 2.7,
   KmvlcadFAD → 0.12, KmvlcadC16EnoylCoAMAT → 1.08, KmvlcadC14EnoylCoAMAT → 1.08,
   KmvlcadC12EnoylCoAMAT → 1.08, KmvlcadFADH → 24.2, Keqvlcad → 6,
   sflcadC16 → 0.9, sflcadC14 → 1, sflcadC12 → 0.9, sflcadC10 → 0.75, sflcadC8 → 0.4,
   Vlcad → 0.01, KmlcadC16AcylCoAMAT → 2.5, KmlcadC14AcylCoAMAT → 7.4,
   KmlcadC12AcylCoAMAT → 9, KmlcadC10AcylCoAMAT → 24.3, KmlcadC8AcylCoAMAT → 123,
```

```
KmlcadFAD → 0.12, KmlcadC16EnoylCoAMAT → 1.08, KmlcadC14EnoylCoAMAT → 1.08,
KmlcadC12EnoylCoAMAT → 1.08, KmlcadC10EnoylCoAMAT → 1.08,
KmlcadC8EnoylCoAMAT → 1.08, KmlcadFADH → 24.2, Keqlcad → 6,
sfmcadC12 → 0.38, sfmcadC10 → 0.8, sfmcadC8 → 0.87, sfmcadC6 → 1, sfmcadC4 → 0.12,
Vmcad → 0.081, KmmcadC12AcylCoAMAT → 5.7, KmmcadC10AcylCoAMAT → 5.4,
KmmcadC8AcylCoAMAT → 4, KmmcadC6AcylCoAMAT → 9.4, KmmcadC4AcylCoAMAT → 135,
KmmcadFAD → 0.12, KmmcadC12EnoylCoAMAT → 1.08, KmmcadC10EnoylCoAMAT → 1.08,
KmmcadC8EnoylCoAMAT → 1.08, KmmcadC6EnoylCoAMAT → 1.08,
KmmcadC4EnoylCoAMAT → 1.08, KmmcadFADH → 24.2, Keqmcad → 6,
sfscadC6 → 0.3, sfscadC4 → 1, Vscad → 0.081, KmscadC6AcylCoAMAT → 285,
KmscadC4AcylCoAMAT → 10.7, KmscadFAD → 0.12, KmscadC6EnoylCoAMAT → 1.08,
KmscadC4EnoylCoAMAT → 1.08, KmscadFADH → 24.2, Keqscad -> 6,
sfcrotC16 → 0.13, sfcrotC14 → 0.2, sfcrotC12 → 0.25, sfcrotC10 → 0.33, sfcrotC8 → 0.58,
sfcrotC6 → 0.83, sfcrotC4 → 1, Vcrot → 3.6, KmcrotC16EnoylCoAMAT → 150,
KmcrotC14EnoylCoAMAT → 100, KmcrotC12EnoylCoAMAT → 25, KmcrotC10EnoylCoAMAT → 25,
KmcrotC8EnoylCoAMAT → 25, KmcrotC6EnoylCoAMAT → 25, KmcrotC4EnoylCoAMAT → 40,
KmcrotC16HydroxyacylCoAMAT → 45, KmcrotC14HydroxyacylCoAMAT → 45,
KmcrotC12HydroxyacylCoAMAT → 45, KmcrotC10HydroxyacylCoAMAT → 45,
KmcrotC8HydroxyacylCoAMAT → 45, KmcrotC6HydroxyacylCoAMAT → 45,
KmcrotC4HydroxyacylCoAMAT → 45, KicrotC4AcetoacylCoA → 1.6, Keqcrot → 3.13,
sfmschadC16 → 0.6, sfmschadC14 → 0.5, sfmschadC12 → 0.43, sfmschadC10 → 0.64,
sfmschadC8 → 0.89, sfmschadC6 → 1, sfmschadC4 → 0.67, Vmschad → 1,
KmmschadC16HydroxyacylCoAMAT → 1.5, KmmschadC14HydroxyacylCoAMAT → 1.8,
KmmschadC12HydroxyacylCoAMAT → 3.7, KmmschadC10HydroxyacylCoAMAT → 8.8,
KmmschadC8HydroxyacylCoAMAT → 16.3, KmmschadC6HydroxyacylCoAMAT → 28.6,
KmmschadC4HydroxyacylCoAMAT → 69.9, KmmschadNADMAT → 58.5,
KmmschadC16KetoacylCoAMAT → 1.4, KmmschadC14KetoacylCoAMAT → 1.4,
KmmschadC12KetoacylCoAMAT → 1.6, KmmschadC10KetoacylCoAMAT → 2.3,
KmmschadC8KetoacylCoAMAT → 4.1, KmmschadC6KetoacylCoAMAT → 5.8,
KmmschadC4AcetoacylCoAMAT → 16.9, KmmschadNADHMAT → 5.4, Keqmschad → 2.17 * 10^-4,
sfmckatC16 → 0, sfmckatC14 → 0.2, sfmckatC12 → 0.38, sfmckatC10 → 0.65,
sfmckatC8 → 0.81, sfmckatC6 → 1, sfmckatC4 → 0.49, Vmckat → 0.377,
KmmckatC16KetoacylCoAMAT → 1.1, KmmckatC14KetoacylCoAMAT → 1.2,
KmmckatC12KetoacylCoAMAT → 1.3, KmmckatC10KetoacylCoAMAT → 2.1,
KmmckatC8KetoacylCoAMAT → 3.2, KmmckatC6KetoacylCoAMAT → 6.7,
KmmckatC4AcetoacylCoAMAT → 12.4, KmmckatCoAMAT → 26.6,
KmmckatC14AcylCoAMAT → 13.83, KmmckatC16AcylCoAMAT → 13.83,
KmmckatC12AcylCoAMAT → 13.83, KmmckatC10AcylCoAMAT → 13.83,
KmmckatC8AcylCoAMAT → 13.83, KmmckatC6AcylCoAMAT → 13.83,
KmmckatC4AcylCoAMAT → 13.83, KmmckatAcetylCoAMAT → 30, Keqmckat → 1051,
sfmtpC16 → 1, sfmtpC14 → 0.9, sfmtpC12 → 0.81, sfmtpC10 → 0.73, sfmtpC8 → 0.34,
Vmtp → 2.84, KmmtpC16EnoylCoAMAT → 25, KmmtpC14EnoylCoAMAT → 25,
KmmtpC12EnoylCoAMAT → 25, KmmtpC10EnoylCoAMAT → 25, KmmtpC8EnoylCoAMAT → 25,
KmmtpNADMAT → 60, KmmtpCoAMAT → 30, KmmtpC14AcylCoAMAT → 13.83,
KmmtpC16AcylCoAMAT → 13.83, KmmtpC12AcylCoAMAT → 13.83,
KmmtpC10AcylCoAMAT → 13.83, KmmtpC8AcylCoAMAT → 13.83, KmmtpC6AcylCoAMAT → 13.83,
KmmtpNADHMAT → 50, KmmtpAcetylCoAMAT → 30, Keqmtp → 0.71,
Ksacesink → 6 000 000, K1acesink → 70, Ksfadhsink → 6 000 000,
K1fadhsink → 0.46, Ksnadhsink → 6 000 000, K1nadhsink → 12,
C16AcylCoACYT → X(*25*), CarCYT → 200, CoACYT → 140, MalCoACYT → 0,
CarMAT -> 950, FADtMAT → 0.77, NADtMAT → 250, CoAMATt → 5000,
VCYT → 2.2 * 10^-6, VMAT → 1.8 * 10^-6, AcetylCoAMAT → 70, FADHMAT → 0.46,
NADHMAT → 12, CE1 → Z (*54.6*) , KmCE1 → 0.74 (*449,
54.6*) (*5.46*10^-2*), nE1 → 1(*0.3, nE1→2.041*)};
tsolScan[X_, Z_] := NDSolve[Join[Odes /. RateEqs /. CoAMATX /. ParmScan[X, Z],
```

```
    InitialConditions], Vars, {t, 0, 1000000000}];

SsScan[X_, Z_] := Module[{SSGuess},
  SSGuess := Table[{Vars[[i]][t],
     (Vars[[i]][900000000] /. tsolScan[X, Z])[[1]]}, {i, 1, Length[Vars]}];
  FindRoot[Table[Odes[[i, 2]] == 0, {i, 1, Length[Odes]}] /. RateEqs /. CoAMATX /.
    ParmScan[X, Z], SSGuess]]
```

In[•]:=
```
ScanDownNDSm[Ystart_, dY_, Yend_] := Monitor[Module[{SS, SSGuess},
   DataDownNDSfluxm = {};
   Xstart = 250;
   Xend = 0;
   YY = {0.0, 1.0};
   For[Y = Ystart, Y ≤ Yend,
    Z = YY[[Y]];
    tsolStart = tsolScan[Xend, Z];
    SSGuess = Table[{Vars[[i]][t],
       (Vars[[i]][900000000] /. tsolStart)[[1]]}, {i, 1, Length[Vars]}];
    SSGuess1 = SSGuess[[All, 1]];
    SSGuess2 = SSGuess[[All, 2]];
    SSGuess1int = SSGuess1 /. t → 0;
    InitialConditionsUD = Thread[SSGuess1int == SSGuess2];
    dX = 1;
    For[X = 250, X ≥ 0,

     tsolScanNDS = NDSolve[Join[Odes /. RateEqs /. CoAMATX /. ParmScan[X, Z],
        InitialConditionsUD], Vars, {t, 0, 1000000000}];
     SSGuess = Table[{Vars[[i]][t], (Vars[[i]][900000000] /. tsolScanNDS)[[1]]},
       {i, 1, Length[Vars]}];
     SSGuess1 = SSGuess[[All, 1]];
     SSGuess2 = SSGuess[[All, 2]];
     SSGuess1int = SSGuess1 /. t → 0;
     InitialConditionsUD = Thread[SSGuess1int == SSGuess2];
     SS = Thread[SSGuess1 → SSGuess2];

     AppendTo[DataDownNDSfluxm,
      {X, Z, 10^3 vcpt1C16 /. RateEqs /. CoAMATX /. ParmScan[X, Z] /. SS}];
     X = X - dX;];
    Y = Y + dY;]
  ], ProgressIndicator[X, {Xstart, Xend}]]
```

In[•]:=
```
ScanDownNDSm[1, 1, 2]
```

In[•]:=
```
ScanUpNDSm[Ystart_, dY_, Yend_] := Monitor[Module[{SS, SSGuess},
   DataUpNDSfluxm = {};
   Xstart = 0;
   Xend = 250;
   YY = {0.0, 1.0};
   For[Y = Ystart, Y ≤ Yend,
    Z = YY[[Y]];
    tsolStart = tsolScan[Xstart, Z ];
    SSGuess = Table[{Vars[[i]][t],
        (Vars[[i]][900000000] /. tsolStart)[[1]]}, {i, 1, Length[Vars]}];
    SSGuess1 = SSGuess[[All, 1]];
    SSGuess2 = SSGuess[[All, 2]];
    SSGuess1int = SSGuess1 /. t → 0;
    InitialConditionsUD = Thread[SSGuess1int == SSGuess2];

    dX = 1;
    For[X = 0, X ≤ 250,

     tsolScanNDS = NDSolve[Join[Odes /. RateEqs /. CoAMATX /. ParmScan[X, Z],
         InitialConditionsUD], Vars, {t, 0, 1000000000}];
     SSGuess = Table[{Vars[[i]][t], (Vars[[i]][900000000] /. tsolScanNDS)[[1]]},
        {i, 1, Length[Vars]}];
     SSGuess1 = SSGuess[[All, 1]];
     SSGuess2 = SSGuess[[All, 2]];
     SSGuess1int = SSGuess1 /. t → 0;
     InitialConditionsUD = Thread[SSGuess1int == SSGuess2];
     SS = Thread[SSGuess1 → SSGuess2];

     AppendTo[DataUpNDSfluxm,
      {X, Z, 10^3 vcpt1C16 /. RateEqs /. CoAMATX /. ParmScan[X, Z] /. SS}];

     X = X + dX;];
    Y = Y + dY;]
  ], ProgressIndicator[X, {Xstart, Xend}]]
```

In[•]:=

In[•]:=
```
ScanUpNDSm[1, 1, 2]
```

```
p3n1 =
 ListLinePlot[{DataUpNDSfluxm[[1 ;; 251, {1, 3}]], DataUpNDSfluxm[[252 ;; 502, {1, 3}]],
   DataDownNDSfluxm[[1 ;; 251, {1, 3}]], DataDownNDSfluxm[[252 ;; 502, {1, 3}]]},
  PlotRange → All, PlotStyle → {Red, Blue, Red, Blue},
  AxesStyle → Directive[Black, 16], LabelStyle → Directive[Black],
  PlotLegends → Placed[LineLegend[{"WT", "ShcKO"}, LabelStyle → {FontSize → 16}],
    {Center, Bottom}], PlotLabel → "p46Shc protein",
  Frame → {{True, False}, {True, False}}, FrameLabel →
   {{"Uptake Flux(μmol.min^-1.gProtein^-1)", None}, {"Palmitoyl-CoA(μM)", None}},
  BaseStyle → {FontSize → 18, FontWeight → "3", AbsoluteThickness[2]},
  FrameStyle → Thickness[0.00005], ImageSize → Scaled[0.3], AspectRatio → 0.75]
```

Out[ ]=

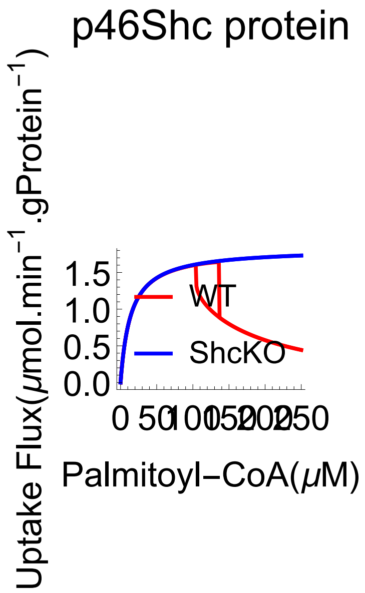

```
p3n1 =
 ListLinePlot[{DataUpNDSfluxm[[1 ;; 251, {1, 3}]], DataUpNDSfluxm[[252 ;; 502, {1, 3}]],
   DataDownNDSfluxm[[1 ;; 251, {1, 3}]], DataDownNDSfluxm[[252 ;; 502, {1, 3}]]},
  PlotRange → All, PlotStyle → {Red, Blue, Red, Blue}, AxesStyle → Directive[Black, 16],
  LabelStyle → Directive[Black], PlotLegends → {"WT", "KO", "WT", "KO"},
  PlotLabel → "p46Shc protein relative expressions",
  Frame → {{True, False}, {True, False}}, FrameLabel →
   {{"Uptake Flux(μmol.min^-1.gProtein^-1)", None}, {"Palmitoyl-CoA(μM)", None}},
  BaseStyle → {FontSize → 18, FontWeight → "2"}, FrameStyle → Thickness[0.00005],
  ImageSize → Scaled[0.25], AspectRatio → 0.75]
```

rotein relative ex

Out[•]=

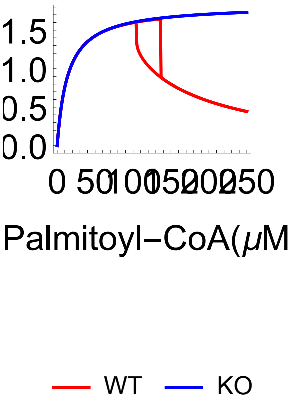

Supplement: S4 Appendix — (ZIP) [file pcbi.1009259.s015.zip › MFAOExtensionwithp46ShcRegulationofMCKAT.pdf]
