# Supplementary material for: Bistability in fatty-acid oxidation resulting from substrate inhibition
Source: PLoS Comput Biol. 2021 Aug 12;17(8):e1009259. doi: 10.1371/journal.pcbi.1009259 (PMC8396765; doi:10.1371/journal.pcbi.1009259)

Mitochondrial Fatty Acid Oxidation Kinetic model with ACOT Extension

Definitions of the various functions

In[∘]:= CPT1[sf_, V_, Kms1_, Kms2_, Kmp1_, Kmp2_, Ki1_, Keq_, S1_, S2_, P1_, P2_, I1_, n_] :=

$$\frac{sf*V*\left(\frac{S1*S2}{Kms1*Kms2}-\frac{P1*P2}{Kms1*Kms2*Keq}\right)}{\left(1+\frac{S1}{Kms1}+\frac{P1}{Kmp1}+\left(\frac{I1}{Ki1}\right)^{n}\right)*\left(1+\frac{S2}{Kms2}+\frac{P2}{Kmp2}\right)}$$

In[∘]:= CACT[Vf_, Vr_, Kms1_, Kms2_, Kmp1_, Kmp2_, Kis1_, Kip2_, Keq_, S1_, S2_, P1_, P2_] :=

$$\frac{Vf*\left(S1*S2-\frac{P1*P2}{Keq}\right)}{S1*S2+Kms2*S1+Kms1*S2*\left(1+\frac{P2}{Kip2}\right)+\frac{Vf}{Vr*Keq}*\left(Kmp2*P1*\left(1+\frac{S1}{Kis1}\right)+P2*(Kmp1+P1)\right)}$$

In[∘]:= CPT2[sf_, V_, Kms1_, Kms2_, Kms3_, Kms4_, Kms5_, Kms6_, Kms7_, Kms8_, Kmp1_, Kmp2_, Kmp3_, Kmp4_, Kmp5_, Kmp6_, Kmp7_, Kmp8_, Keq_, S1_, S2_, S3_, S4_, S5_, S6_, S7_, S8_, P1_, P2_, P3_, P4_, P5_, P6_, P7_, P8_] :=

$$\left(sf*V*\left(\frac{S1*S8}{Kms1*Kms8}-\frac{P1*P8}{Kms1*Kms8*Keq}\right)\right)\Big/\left(\left(1+\frac{S1}{Kms1}+\frac{P1}{Kmp1}+\frac{S2}{Kms2}+\frac{P2}{Kmp2}+\frac{S3}{Kms3}+\frac{P3}{Kmp3}+\frac{S4}{Kms4}+\frac{P4}{Kmp4}+\frac{S5}{Kms5}+\frac{P5}{Kmp5}+\frac{S6}{Kms6}+\frac{P6}{Kmp6}+\frac{S7}{Kms7}+\frac{P7}{Kmp7}\right)*\left(1+\frac{S8}{Kms8}+\frac{P8}{Kmp8}\right)\right)$$

In[∘]:= VLCAD[sf_, V_, Kms1_, Kms2_, Kms3_, Kms4_, Kmp1_, Kmp2_, Kmp3_, Kmp4_, Keq_, S1_, S2_, S3_, S4_, P1_, P2_, P3_, P4_] :=

$$\frac{sf*V*\left(\frac{S1*(S4-P4)}{Kms1*Kms4}-\frac{P1*P4}{Kms1*Kms4*Keq}\right)}{\left(1+\frac{S1}{Kms1}+\frac{P1}{Kmp1}+\frac{S2}{Kms2}+\frac{P2}{Kmp2}+\frac{S3}{Kms3}+\frac{P3}{Kmp3}\right)*\left(1+\frac{(S4-P4)}{Kms4}+\frac{P4}{Kmp4}\right)}$$

In[∘]:= LCAD[sf_, V_, Kms1_, Kms2_, Kms3_, Kms4_, Kms5_, Kms6_, Kmp1_, Kmp2_, Kmp3_, Kmp4_, Kmp5_, Kmp6_, Keq_, S1_, S2_, S3_, S4_, S5_, S6_, P1_, P2_, P3_, P4_, P5_, P6_] :=

$$\frac{sf*V*\left(\frac{S1*(S6-P6)}{Kms1*Kms6}-\frac{P1*P6}{Kms1*Kms6*Keq}\right)}{\left(1+\frac{S1}{Kms1}+\frac{P1}{Kmp1}+\frac{S2}{Kms2}+\frac{P2}{Kmp2}+\frac{S3}{Kms3}+\frac{P3}{Kmp3}+\frac{S4}{Kms4}+\frac{P4}{Kmp4}+\frac{S5}{Kms5}+\frac{P5}{Kmp5}\right)*\left(1+\frac{(S6-P6)}{Kms6}+\frac{P6}{Kmp6}\right)}$$

In[∘]:= MCAD[sf_, V_, Kms1_, Kms2_, Kms3_, Kms4_, Kms5_, Kms6_, Kmp1_, Kmp2_, Kmp3_, Kmp4_, Kmp5_, Kmp6_, Keq_, S1_, S2_, S3_, S4_, S5_, S6_, P1_, P2_, P3_, P4_, P5_, P6_] :=

$$\frac{sf*V*\left(\frac{S1*(S6-P6)}{Kms1*Kms6}-\frac{P1*P6}{Kms1*Kms6*Keq}\right)}{\left(1+\frac{S1}{Kms1}+\frac{P1}{Kmp1}+\frac{S2}{Kms2}+\frac{P2}{Kmp2}+\frac{S3}{Kms3}+\frac{P3}{Kmp3}+\frac{S4}{Kms4}+\frac{P4}{Kmp4}+\frac{S5}{Kms5}+\frac{P5}{Kmp5}\right)*\left(1+\frac{(S6-P6)}{Kms6}+\frac{P6}{Kmp6}\right)}$$

In[∘]:= SCAD[sf_, V_, Kms1_, Kms2_, Kms3_, Kmp1_, Kmp2_, Kmp3_, Keq_, S1_, S2_, S3_, P1_, P2_, P3_] :=

$$\frac{sf*V*\left(\frac{S1*(S3-P3)}{Kms1*Kms3}-\frac{P1*P3}{Kms1*Kms3*Keq}\right)}{\left(1+\frac{S1}{Kms1}+\frac{P1}{Kmp1}+\frac{S2}{Kms2}+\frac{P2}{Kmp2}\right)*\left(1+\frac{(S3-P3)}{Kms3}+\frac{P3}{Kmp3}\right)}$$

In[•]:=

$$\mathrm{CROT}[\mathrm{sf\_}, \mathrm{V\_}, \mathrm{Kms1\_}, \mathrm{Kms2\_}, \mathrm{Kms3\_}, \mathrm{Kms4\_}, \mathrm{Kms5\_}, \mathrm{Kms6\_}, \mathrm{Kms7\_}, \mathrm{Kmp1\_}, \mathrm{Kmp2\_}, \mathrm{Kmp3\_}, \mathrm{Kmp4\_}, \mathrm{Kmp5\_}, \mathrm{Kmp6\_}, \mathrm{Kmp7\_}, \mathrm{Ki1\_}, \mathrm{Keq\_}, \mathrm{S1\_}, \mathrm{S2\_}, \mathrm{S3\_}, \mathrm{S4\_}, \mathrm{S5\_}, \mathrm{S6\_}, \mathrm{S7\_}, \mathrm{P1\_}, \mathrm{P2\_}, \mathrm{P3\_}, \mathrm{P4\_}, \mathrm{P5\_}, \mathrm{P6\_}, \mathrm{P7\_}, \mathrm{I1\_}] := \frac{\mathrm{sf} * \mathrm{V} * \left(\frac{\mathrm{S1}}{\mathrm{Kms1}} - \frac{\mathrm{P1}}{\mathrm{Kms1} * \mathrm{Keq}}\right)}{1 + \frac{\mathrm{S1}}{\mathrm{Kms1}} + \frac{\mathrm{P1}}{\mathrm{Kmp1}} + \frac{\mathrm{S2}}{\mathrm{Kms2}} + \frac{\mathrm{P2}}{\mathrm{Kmp2}} + \frac{\mathrm{S3}}{\mathrm{Kms3}} + \frac{\mathrm{P3}}{\mathrm{Kmp3}} + \frac{\mathrm{S4}}{\mathrm{Kms4}} + \frac{\mathrm{P4}}{\mathrm{Kmp4}} + \frac{\mathrm{S5}}{\mathrm{Kms5}} + \frac{\mathrm{P5}}{\mathrm{Kmp5}} + \frac{\mathrm{S6}}{\mathrm{Kms6}} + \frac{\mathrm{P6}}{\mathrm{Kmp6}} + \frac{\mathrm{S7}}{\mathrm{Kms7}} + \frac{\mathrm{P7}}{\mathrm{Kmp7}} + \frac{\mathrm{I1}}{\mathrm{Ki1}}}$$

In[•]:=

$$\mathrm{MSCHAD}[\mathrm{sf\_}, \mathrm{V\_}, \mathrm{Kms1\_}, \mathrm{Kms2\_}, \mathrm{Kms3\_}, \mathrm{Kms4\_}, \mathrm{Kms5\_}, \mathrm{Kms6\_}, \mathrm{Kms7\_}, \mathrm{Kms8\_}, \mathrm{Kmp1\_}, \mathrm{Kmp2\_}, \mathrm{Kmp3\_}, \mathrm{Kmp4\_}, \mathrm{Kmp5\_}, \mathrm{Kmp6\_}, \mathrm{Kmp7\_}, \mathrm{Kmp8\_}, \mathrm{Keq\_}, \mathrm{S1\_}, \mathrm{S2\_}, \mathrm{S3\_}, \mathrm{S4\_}, \mathrm{S5\_}, \mathrm{S6\_}, \mathrm{S7\_}, \mathrm{S8\_}, \mathrm{P1\_}, \mathrm{P2\_}, \mathrm{P3\_}, \mathrm{P4\_}, \mathrm{P5\_}, \mathrm{P6\_}, \mathrm{P7\_}, \mathrm{P8\_}] := \left(\mathrm{sf} * \mathrm{V} * \left(\frac{\mathrm{S1} * (\mathrm{S8} - \mathrm{P8})}{\mathrm{Kms1} * \mathrm{Kms8}} - \frac{\mathrm{P1} * \mathrm{P8}}{\mathrm{Kms1} * \mathrm{Kms8} * \mathrm{Keq}}\right)\right) \Big/ \left(\left(1 + \frac{\mathrm{S1}}{\mathrm{Kms1}} + \frac{\mathrm{P1}}{\mathrm{Kmp1}} + \frac{\mathrm{S2}}{\mathrm{Kms2}} + \frac{\mathrm{P2}}{\mathrm{Kmp2}} + \frac{\mathrm{S3}}{\mathrm{Kms3}} + \frac{\mathrm{P3}}{\mathrm{Kmp3}} + \frac{\mathrm{S4}}{\mathrm{Kms4}} + \frac{\mathrm{P4}}{\mathrm{Kmp4}} + \frac{\mathrm{S5}}{\mathrm{Kms5}} + \frac{\mathrm{P5}}{\mathrm{Kmp5}} + \frac{\mathrm{S6}}{\mathrm{Kms6}} + \frac{\mathrm{P6}}{\mathrm{Kmp6}} + \frac{\mathrm{S7}}{\mathrm{Kms7}} + \frac{\mathrm{P7}}{\mathrm{Kmp7}}\right) * \left(1 + \frac{(\mathrm{S8} - \mathrm{P8})}{\mathrm{Kms8}} + \frac{\mathrm{P8}}{\mathrm{Kmp8}}\right)\right)$$

In[•]:=

$$\mathrm{MCKATA}[\mathrm{sf\_}, \mathrm{V\_}, \mathrm{Kms1\_}, \mathrm{Kms2\_}, \mathrm{Kms3\_}, \mathrm{Kms4\_}, \mathrm{Kms5\_}, \mathrm{Kms6\_}, \mathrm{Kms7\_}, \mathrm{Kms8\_}, \mathrm{Kmp1\_}, \mathrm{Kmp2\_}, \mathrm{Kmp3\_}, \mathrm{Kmp4\_}, \mathrm{Kmp5\_}, \mathrm{Kmp6\_}, \mathrm{Kmp7\_}, \mathrm{Kmp8\_}, \mathrm{Keq\_}, \mathrm{S1\_}, \mathrm{S2\_}, \mathrm{S3\_}, \mathrm{S4\_}, \mathrm{S5\_}, \mathrm{S6\_}, \mathrm{S7\_}, \mathrm{S8\_}, \mathrm{P1\_}, \mathrm{P2\_}, \mathrm{P3\_}, \mathrm{P4\_}, \mathrm{P5\_}, \mathrm{P6\_}, \mathrm{P7\_}, \mathrm{P8\_}, \mathrm{E1\_}, \mathrm{KmE1\_}, \mathrm{nm\_}] := \left(\mathrm{sf} * \mathrm{V} * \left(\frac{\mathrm{S1} * \mathrm{S8}}{\mathrm{Kms1} * \mathrm{Kms8}} - \frac{\mathrm{P1} * \mathrm{P8}}{\mathrm{Kms1} * \mathrm{Kms8} * \mathrm{Keq}}\right)\right) \Big/ \left(\left(1 + \frac{\mathrm{S1}}{\mathrm{Kms1}} + \frac{\mathrm{P1}}{\mathrm{Kmp1}} + \frac{\mathrm{S2}}{\mathrm{Kms2}} + \frac{\mathrm{P2}}{\mathrm{Kmp2}} + \frac{\mathrm{S3}}{\mathrm{Kms3}} + \frac{\mathrm{P3}}{\mathrm{Kmp3}} + \frac{\mathrm{S4}}{\mathrm{Kms4}} + \frac{\mathrm{P4}}{\mathrm{Kmp4}} + \frac{\mathrm{S5}}{\mathrm{Kms5}} + \frac{\mathrm{P5}}{\mathrm{Kmp5}} + \frac{\mathrm{S6}}{\mathrm{Kms6}} + \frac{\mathrm{P6}}{\mathrm{Kmp6}} + \frac{\mathrm{S7}}{\mathrm{Kms7}} + \frac{\mathrm{P7}}{\mathrm{Kmp7}} + \frac{\mathrm{P8}}{\mathrm{Kmp8}}\right) * \left(1 + \frac{\mathrm{S8}}{\mathrm{Kms8}} + \frac{\mathrm{P8}}{\mathrm{Kmp8}}\right)\right)$$

In[•]:=

$$\mathrm{MCKATB}[\mathrm{sf\_}, \mathrm{V\_}, \mathrm{Kms1\_}, \mathrm{Kms2\_}, \mathrm{Kms3\_}, \mathrm{Kms4\_}, \mathrm{Kms5\_}, \mathrm{Kms6\_}, \mathrm{Kms7\_}, \mathrm{Kms8\_}, \mathrm{Kmp1\_}, \mathrm{Kmp2\_}, \mathrm{Kmp3\_}, \mathrm{Kmp4\_}, \mathrm{Kmp5\_}, \mathrm{Kmp6\_}, \mathrm{Kmp7\_}, \mathrm{Kmp8\_}, \mathrm{Keq\_}, \mathrm{S1\_}, \mathrm{S2\_}, \mathrm{S3\_}, \mathrm{S4\_}, \mathrm{S5\_}, \mathrm{S6\_}, \mathrm{S7\_}, \mathrm{S8\_}, \mathrm{P1\_}, \mathrm{P2\_}, \mathrm{P3\_}, \mathrm{P4\_}, \mathrm{P5\_}, \mathrm{P6\_}, \mathrm{P7\_}, \mathrm{P8\_}, \mathrm{E1\_}, \mathrm{KmE1\_}, \mathrm{nm\_}] := \left(\mathrm{sf} * \mathrm{V} * \left(\frac{\mathrm{S1} * \mathrm{S8}}{\mathrm{Kms1} * \mathrm{Kms8}} - \frac{\mathrm{P8} * \mathrm{P8}}{\mathrm{Kms1} * \mathrm{Kms8} * \mathrm{Keq}}\right)\right) \Big/ \left(\left(1 + \frac{\mathrm{S1}}{\mathrm{Kms1}} + \frac{\mathrm{P1}}{\mathrm{Kmp1}} + \frac{\mathrm{S2}}{\mathrm{Kms2}} + \frac{\mathrm{P2}}{\mathrm{Kmp2}} + \frac{\mathrm{S3}}{\mathrm{Kms3}} + \frac{\mathrm{P3}}{\mathrm{Kmp3}} + \frac{\mathrm{S4}}{\mathrm{Kms4}} + \frac{\mathrm{P4}}{\mathrm{Kmp4}} + \frac{\mathrm{S5}}{\mathrm{Kms5}} + \frac{\mathrm{P5}}{\mathrm{Kmp5}} + \frac{\mathrm{S6}}{\mathrm{Kms6}} + \frac{\mathrm{P6}}{\mathrm{Kmp6}} + \frac{\mathrm{S7}}{\mathrm{Kms7}} + \frac{\mathrm{P7}}{\mathrm{Kmp7}} + \frac{\mathrm{P8}}{\mathrm{Kmp8}}\right) * \left(1 + \frac{\mathrm{S8}}{\mathrm{Kms8}} + \frac{\mathrm{P8}}{\mathrm{Kmp8}}\right)\right)$$

In[•]:=

$$\mathrm{MTP}[\mathrm{sf\_}, \mathrm{V\_}, \mathrm{Kms1\_}, \mathrm{Kms2\_}, \mathrm{Kms3\_}, \mathrm{Kms4\_}, \mathrm{Kms5\_}, \mathrm{Kms7\_}, \mathrm{Kms8\_}, \mathrm{Kmp1\_}, \mathrm{Kmp2\_}, \mathrm{Kmp3\_}, \mathrm{Kmp4\_}, \mathrm{Kmp5\_}, \mathrm{Kmp6\_}, \mathrm{Kmp7\_}, \mathrm{Kmp8\_}, \mathrm{Ki1\_}, \mathrm{Keq\_}, \mathrm{S1\_}, \mathrm{S2\_}, \mathrm{S3\_}, \mathrm{S4\_}, \mathrm{S5\_}, \mathrm{S7\_}, \mathrm{S8\_}, \mathrm{P1\_}, \mathrm{P2\_}, \mathrm{P3\_}, \mathrm{P4\_}, \mathrm{P5\_}, \mathrm{P6\_}, \mathrm{P7\_}, \mathrm{P8\_}, \mathrm{I1\_}] := \left(\mathrm{sf} * \mathrm{V} * \left(\frac{\mathrm{S1} * (\mathrm{S7} - \mathrm{P7}) * \mathrm{S8}}{\mathrm{Kms1} * \mathrm{Kms7} * \mathrm{Kms8}} - \frac{\mathrm{P1} * \mathrm{P7} * \mathrm{P8}}{\mathrm{Kms1} * \mathrm{Kms7} * \mathrm{Kms8} * \mathrm{Keq}}\right)\right) \Big/ \left(\left(1 + \frac{\mathrm{S1}}{\mathrm{Kms1}} + \frac{\mathrm{P1}}{\mathrm{Kmp1}} + \frac{\mathrm{S2}}{\mathrm{Kms2}} + \frac{\mathrm{P2}}{\mathrm{Kmp2}} + \frac{\mathrm{S3}}{\mathrm{Kms3}} + \frac{\mathrm{P3}}{\mathrm{Kmp3}} + \frac{\mathrm{S4}}{\mathrm{Kms4}} + \frac{\mathrm{P4}}{\mathrm{Kmp4}} + \frac{\mathrm{S5}}{\mathrm{Kms5}} + \frac{\mathrm{P5}}{\mathrm{Kmp5}} + \frac{\mathrm{P6}}{\mathrm{Kmp6}} + \frac{\mathrm{I1}}{\mathrm{Ki1}}\right) * \left(1 + \frac{(\mathrm{S7} - \mathrm{P7})}{\mathrm{Kms7}} + \frac{\mathrm{P7}}{\mathrm{Kmp7}}\right) * \left(1 + \frac{\mathrm{S8}}{\mathrm{Kms8}} + \frac{\mathrm{P8}}{\mathrm{Kmp8}}\right)\right)$$

In[•]:=

$$\mathrm{RES}[\mathrm{Ks\_}, \mathrm{S\_}, \mathrm{K1\_}] := \mathrm{Ks} * (\mathrm{S} - \mathrm{K1})$$

In[•]:=

$$\mathrm{ACOT}[\mathrm{sf\_}, \mathrm{V\_}, \mathrm{Kms1\_}, \mathrm{Kms2\_}, \mathrm{Kms3\_}, \mathrm{Kms4\_}, \mathrm{Kms5\_}, \mathrm{Kms6\_}, \mathrm{Kms7\_}, \mathrm{Kmcoa\_}, \mathrm{Kmp1\_}, \mathrm{Kmp2\_}, \mathrm{Kmp3\_}, \mathrm{Kmp4\_}, \mathrm{Kmp5\_}, \mathrm{Kmp6\_}, \mathrm{Kmp7\_}, \mathrm{Keq\_}, \mathrm{S1\_}, \mathrm{S2\_}, \mathrm{S3\_}, \mathrm{S4\_}, \mathrm{S5\_}, \mathrm{S6\_}, \mathrm{S7\_}, \mathrm{coa\_}, \mathrm{P1\_}, \mathrm{P2\_}, \mathrm{P3\_}, \mathrm{P4\_}, \mathrm{P5\_}, \mathrm{P6\_}, \mathrm{P7\_}] := \left(\mathrm{sf} * \mathrm{V} * \left(\frac{\mathrm{S1}}{\mathrm{Kms1}} - \frac{\mathrm{coa} * \mathrm{P1}}{\mathrm{Kms1} * \mathrm{Keq}}\right)\right) \Big/ \left(\left(1 + \frac{\mathrm{S1}}{\mathrm{Kms1}} + \frac{\mathrm{P1}}{\mathrm{Kmp1}} + \frac{\mathrm{S2}}{\mathrm{Kms2}} + \frac{\mathrm{P2}}{\mathrm{Kmp2}} + \frac{\mathrm{S3}}{\mathrm{Kms3}} + \frac{\mathrm{P3}}{\mathrm{Kmp3}} + \frac{\mathrm{S4}}{\mathrm{Kms4}} + \frac{\mathrm{P4}}{\mathrm{Kmp4}} + \frac{\mathrm{S5}}{\mathrm{Kms5}} + \frac{\mathrm{P5}}{\mathrm{Kmp5}} + \frac{\mathrm{S6}}{\mathrm{Kms6}} + \frac{\mathrm{P6}}{\mathrm{Kmp6}} + \frac{\mathrm{S7}}{\mathrm{Kms7}} + \frac{\mathrm{P7}}{\mathrm{Kmp7}}\right) * \left(1 + \frac{\mathrm{coa}}{\mathrm{Kmcoa}}\right)\right)$$

In[•]:=
```
ExportRate[ke_, S_] := ke * S
```

In[•]:=

Define the differential equations

In[•]:=
```
Odes = {
   C16AcylCarCYT'[t] == (vcpt1C16 - vcactC16) / VCYT,
   C16AcylCarMAT'[t] == (vcactC16 - vcpt2C16) / VMAT,
   C16AcylCoAMAT'[t] == (vcpt2C16 - vvlcadC16 - vlcadC16 - vacotC16) / VMAT,
   C16EnoylCoAMAT'[t] == (vvlcadC16 + vlcadC16 - vcrotC16 - vmtpC16) / VMAT,
   C16HydroxyacylCoAMAT'[t] == (vcrotC16 - vmschadC16) / VMAT,
   C16KetoacylCoAMAT'[t] == (vmschadC16 - vmckatC16) / VMAT,
   C14AcylCarCYT'[t] == (-vcactC14) / VCYT,
   C14AcylCarMAT'[t] == (vcactC14 - vcpt2C14) / VMAT,
   C14AcylCoAMAT'[t] ==
    (vcpt2C14 + vmtpC16 + vmckatC16 - vvlcadC14 - vlcadC14 - vacotC14) / VMAT,
   C14EnoylCoAMAT'[t] == (vvlcadC14 + vlcadC14 - vcrotC14 - vmtpC14) / VMAT,
   C14HydroxyacylCoAMAT'[t] == (vcrotC14 - vmschadC14) / VMAT,
   C14KetoacylCoAMAT'[t] == (vmschadC14 - vmckatC14) / VMAT,
   C12AcylCarCYT'[t] == (-vcactC12) / VCYT,
   C12AcylCarMAT'[t] == (vcactC12 - vcpt2C12) / VMAT,
   C12AcylCoAMAT'[t] ==
    (vcpt2C12 + vmtpC14 + vmckatC14 - vvlcadC12 - vlcadC12 - vmcadC12 - vacotC12) / VMAT,
   C12EnoylCoAMAT'[t] == (vvlcadC12 + vlcadC12 + vmcadC12 - vcrotC12 - vmtpC12) / VMAT,
   C12HydroxyacylCoAMAT'[t] == (vcrotC12 - vmschadC12) / VMAT,
   C12KetoacylCoAMAT'[t] == (vmschadC12 - vmckatC12) / VMAT,
   C10AcylCarCYT'[t] == (-vcactC10) / VCYT,
   C10AcylCarMAT'[t] == (vcactC10 - vcpt2C10) / VMAT,
   C10AcylCoAMAT'[t] ==
    (vcpt2C10 + vmtpC12 + vmckatC12 - vlcadC10 - vmcadC10 - vacotC10) / VMAT,
   C10EnoylCoAMAT'[t] == (vlcadC10 + vmcadC10 - vcrotC10 - vmtpC10) / VMAT,
   C10HydroxyacylCoAMAT'[t] == (vcrotC10 - vmschadC10) / VMAT,
   C10KetoacylCoAMAT'[t] == (vmschadC10 - vmckatC10) / VMAT,
   C8AcylCarCYT'[t] == (-vcactC8) / VCYT,
   C8AcylCarMAT'[t] == (vcactC8 - vcpt2C8) / VMAT,
   C8AcylCoAMAT'[t] ==
    (vcpt2C8 + vmtpC10 + vmckatC10 - vlcadC8 - vmcadC8 - vacotC8) / VMAT,
   C8EnoylCoAMAT'[t] == (vlcadC8 + vmcadC8 - vcrotC8 - vmtpC8) / VMAT,
   C8HydroxyacylCoAMAT'[t] == (vcrotC8 - vmschadC8) / VMAT,
   C8KetoacylCoAMAT'[t] == (vmschadC8 - vmckatC8) / VMAT,
   C6AcylCarCYT'[t] == (-vcactC6) / VCYT,
   C6AcylCarMAT'[t] == (vcactC6 - vcpt2C6) / VMAT,
   C6AcylCoAMAT'[t] == (vcpt2C6 + vmtpC8 + vmckatC8 - vmcadC6 - vscadC6 - vacotC6) / VMAT,
   C6EnoylCoAMAT'[t] == (vmcadC6 + vscadC6 - vcrotC6) / VMAT,
   C6HydroxyacylCoAMAT'[t] == (vcrotC6 - vmschadC6) / VMAT,
   C6KetoacylCoAMAT'[t] == (vmschadC6 - vmckatC6) / VMAT,
   C4AcylCarCYT'[t] == (-vcactC4) / VCYT,
   C4AcylCarMAT'[t] == (vcactC4 - vcpt2C4) / VMAT,
   C4AcylCoAMAT'[t] == (vcpt2C4 + vmckatC6 - vmcadC4 - vscadC4 - vacotC4) / VMAT,
   C4EnoylCoAMAT'[t] == (vmcadC4 + vscadC4 - vcrotC4) / VMAT,
   C4HydroxyacylCoAMAT'[t] == (vcrotC4 - vmschadC4) / VMAT,
   C4AcetoacylCoAMAT'[t] == (vmschadC4 - vmckatC4) / VMAT,
   AcetylCoAMAT'[t] ==
    (1 / VMAT) (vmtpC16 + vmckatC16 + vmtpC14 + vmckatC14 + vmtpC12 + vmckatC12 +
```

```
        vmtpC10 + vmckatC10 + vmtpC8 + vmckatC8 + vmckatC6 + 2 * vmckatC4 - vacesink),
    FADHMAT'[t] == (1 / VMAT) (vvlcadC16 + vvlcadC14 + vvlcadC12 + vlcadC16 +
        vlcadC14 + vlcadC12 + vlcadC10 + vlcadC8 + vmcadC12 + vmcadC10 +
        vmcadC8 + vmcadC6 + vmcadC4 + vscadC6 + vscadC4 - vfadhsink),
    NADHMAT'[t] == (1 / VMAT) (vmtpC16 + vmtpC14 + vmtpC12 + vmtpC10 +
        vmtpC8 + vmschadC16 + vmschadC14 + vmschadC12 + vmschadC10 +
        vmschadC8 + vmschadC6 + vmschadC4 - vnadhsink),
    C16FFA'[t] == (vacotC16 - vffaC16) / VMAT,
    C14FFA'[t] == (vacotC14 - vffaC14) / VMAT,
    C12FFA'[t] == (vacotC12 - vffaC12) / VMAT,
    C10FFA'[t] == (vacotC10 - vffaC10) / VMAT,
    C8FFA'[t] == (vacotC8 - vffaC8) / VMAT,
    C6FFA'[t] == (vacotC6 - vffaC6) / VMAT,
    C4FFA'[t] == (vacotC4 - vffaC4) / VMAT};

RateEqs = {vcpt1C16 → CPT1[sfcpt1C16, Vcpt1, Kmcpt1C16AcylCoACYT,
      Kmcpt1CarCYT, Kmcpt1C16AcylCarCYT, Kmcpt1CoACYT, Kicpt1MalCoACYT, Keqcpt1,
      C16AcylCoACYT, CarCYT, C16AcylCarCYT[t], CoACYT, MalCoACYT, ncpt1],
    vcactC16 → CACT[Vfcact, Vrcact, KmcactC16AcylCarCYT, KmcactCarMAT,
      KmcactC16AcylCarMAT, KmcactCarCYT, KicactC16AcylCarCYT, KicactCarCYT,
      Keqcact, C16AcylCarCYT[t], CarMAT, C16AcylCarMAT[t], CarCYT],
    vcactC14 → CACT[Vfcact, Vrcact, KmcactC14AcylCarCYT, KmcactCarMAT,
      KmcactC14AcylCarMAT, KmcactCarCYT, KicactC14AcylCarCYT, KicactCarCYT,
      Keqcact, C14AcylCarCYT[t], CarMAT, C14AcylCarMAT[t], CarCYT],
    vcactC12 → CACT[Vfcact, Vrcact, KmcactC12AcylCarCYT, KmcactCarMAT,
      KmcactC12AcylCarMAT, KmcactCarCYT, KicactC12AcylCarCYT, KicactCarCYT,
      Keqcact, C12AcylCarCYT[t], CarMAT, C12AcylCarMAT[t], CarCYT],
    vcactC10 → CACT[Vfcact, Vrcact, KmcactC10AcylCarCYT, KmcactCarMAT,
      KmcactC10AcylCarMAT, KmcactCarCYT, KicactC10AcylCarCYT, KicactCarCYT,
      Keqcact, C10AcylCarCYT[t], CarMAT, C10AcylCarMAT[t], CarCYT],
    vcactC8 → CACT[Vfcact, Vrcact, KmcactC8AcylCarCYT, KmcactCarMAT,
      KmcactC8AcylCarMAT, KmcactCarCYT, KicactC8AcylCarCYT, KicactCarCYT,
      Keqcact, C8AcylCarCYT[t], CarMAT, C8AcylCarMAT[t], CarCYT],
    vcactC6 → CACT[Vfcact, Vrcact, KmcactC6AcylCarCYT, KmcactCarMAT,
      KmcactC6AcylCarMAT, KmcactCarCYT, KicactC6AcylCarCYT, KicactCarCYT,
      Keqcact, C6AcylCarCYT[t], CarMAT, C6AcylCarMAT[t], CarCYT],
    vcactC4 → CACT[Vfcact, Vrcact, KmcactC4AcylCarCYT, KmcactCarMAT,
      KmcactC4AcylCarMAT, KmcactCarCYT, KicactC4AcylCarCYT, KicactCarCYT,
      Keqcact, C4AcylCarCYT[t], CarMAT, C4AcylCarMAT[t], CarCYT],
    vcpt2C16 → CPT2[sfcpt2C16, Vcpt2, Kmcpt2C16AcylCarMAT, Kmcpt2C14AcylCarMAT,
      Kmcpt2C12AcylCarMAT, Kmcpt2C10AcylCarMAT, Kmcpt2C8AcylCarMAT, Kmcpt2C6AcylCarMAT,
      Kmcpt2C4AcylCarMAT, Kmcpt2CoAMAT, Kmcpt2C16AcylCoAMAT, Kmcpt2C14AcylCoAMAT,
      Kmcpt2C12AcylCoAMAT, Kmcpt2C10AcylCoAMAT, Kmcpt2C8AcylCoAMAT, Kmcpt2C6AcylCoAMAT,
      Kmcpt2C4AcylCoAMAT, Kmcpt2CarMAT, Keqcpt2, C16AcylCarMAT[t], C14AcylCarMAT[t],
      C12AcylCarMAT[t], C10AcylCarMAT[t], C8AcylCarMAT[t], C6AcylCarMAT[t],
      C4AcylCarMAT[t], CoAMAT, C16AcylCoAMAT[t], C14AcylCoAMAT[t], C12AcylCoAMAT[t],
      C10AcylCoAMAT[t], C8AcylCoAMAT[t], C6AcylCoAMAT[t], C4AcylCoAMAT[t], CarMAT],
    vcpt2C14 → CPT2[sfcpt2C14, Vcpt2, Kmcpt2C14AcylCarMAT, Kmcpt2C16AcylCarMAT,
      Kmcpt2C12AcylCarMAT, Kmcpt2C10AcylCarMAT, Kmcpt2C8AcylCarMAT, Kmcpt2C6AcylCarMAT,
      Kmcpt2C4AcylCoAMAT, Kmcpt2CoAMAT, Kmcpt2C14AcylCoAMAT, Kmcpt2C16AcylCoAMAT,
      Kmcpt2C12AcylCoAMAT, Kmcpt2C10AcylCoAMAT, Kmcpt2C8AcylCoAMAT, Kmcpt2C6AcylCoAMAT,
      Kmcpt2C4AcylCoAMAT, Kmcpt2CarMAT, Keqcpt2, C14AcylCarMAT[t], C16AcylCarMAT[t],
      C12AcylCarMAT[t], C10AcylCarMAT[t], C8AcylCarMAT[t], C6AcylCarMAT[t],
      C4AcylCarMAT[t], CoAMAT, C14AcylCoAMAT[t], C16AcylCoAMAT[t], C12AcylCoAMAT[t],
      C10AcylCoAMAT[t], C8AcylCoAMAT[t], C6AcylCoAMAT[t], C4AcylCoAMAT[t], CarMAT],
```

```
vcpt2C12 → CPT2[sfcpt2C12, Vcpt2, Kmcpt2C12AcylCarMAT, Kmcpt2C16AcylCarMAT,
   Kmcpt2C14AcylCarMAT, Kmcpt2C10AcylCarMAT, Kmcpt2C8AcylCarMAT, Kmcpt2C6AcylCarMAT,
   Kmcpt2C4AcylCarMAT, Kmcpt2CoAMAT, Kmcpt2C12AcylCoAMAT, Kmcpt2C16AcylCoAMAT,
   Kmcpt2C14AcylCoAMAT, Kmcpt2C10AcylCoAMAT, Kmcpt2C8AcylCoAMAT, Kmcpt2C6AcylCoAMAT,
   Kmcpt2C4AcylCoAMAT, Kmcpt2CarMAT, Keqcpt2, C12AcylCarMAT[t], C16AcylCarMAT[t],
   C14AcylCarMAT[t], C10AcylCarMAT[t], C8AcylCarMAT[t], C6AcylCarMAT[t],
   C4AcylCarMAT[t], CoAMAT, C12AcylCoAMAT[t], C16AcylCoAMAT[t], C14AcylCoAMAT[t],
   C10AcylCoAMAT[t], C8AcylCoAMAT[t], C6AcylCoAMAT[t], C4AcylCoAMAT[t], CarMAT],
vcpt2C10 → CPT2[sfcpt2C10, Vcpt2, Kmcpt2C10AcylCarMAT, Kmcpt2C16AcylCarMAT,
   Kmcpt2C14AcylCarMAT, Kmcpt2C12AcylCarMAT, Kmcpt2C8AcylCarMAT, Kmcpt2C6AcylCarMAT,
   Kmcpt2C4AcylCarMAT, Kmcpt2CoAMAT, Kmcpt2C10AcylCoAMAT, Kmcpt2C16AcylCoAMAT,
   Kmcpt2C14AcylCoAMAT, Kmcpt2C12AcylCoAMAT, Kmcpt2C8AcylCoAMAT, Kmcpt2C6AcylCoAMAT,
   Kmcpt2C4AcylCoAMAT, Kmcpt2CarMAT, Keqcpt2, C10AcylCarMAT[t], C16AcylCarMAT[t],
   C14AcylCarMAT[t], C12AcylCarMAT[t], C8AcylCarMAT[t], C6AcylCarMAT[t],
   C4AcylCarMAT[t], CoAMAT, C10AcylCoAMAT[t], C16AcylCoAMAT[t], C14AcylCoAMAT[t],
   C12AcylCoAMAT[t], C8AcylCoAMAT[t], C6AcylCoAMAT[t], C4AcylCoAMAT[t], CarMAT],
vcpt2C8 → CPT2[sfcpt2C8, Vcpt2, Kmcpt2C8AcylCarMAT, Kmcpt2C16AcylCarMAT,
   Kmcpt2C14AcylCarMAT, Kmcpt2C12AcylCarMAT, Kmcpt2C10AcylCarMAT, Kmcpt2C6AcylCarMAT,
   Kmcpt2C4AcylCarMAT, Kmcpt2CoAMAT, Kmcpt2C8AcylCoAMAT, Kmcpt2C16AcylCoAMAT,
   Kmcpt2C14AcylCoAMAT, Kmcpt2C12AcylCoAMAT, Kmcpt2C10AcylCoAMAT, Kmcpt2C6AcylCoAMAT,
   Kmcpt2C4AcylCoAMAT, Kmcpt2CarMAT, Keqcpt2, C8AcylCarMAT[t], C16AcylCarMAT[t],
   C14AcylCarMAT[t], C12AcylCarMAT[t], C10AcylCarMAT[t], C6AcylCarMAT[t],
   C4AcylCarMAT[t], CoAMAT, C8AcylCoAMAT[t], C16AcylCoAMAT[t], C14AcylCoAMAT[t],
   C12AcylCoAMAT[t], C10AcylCoAMAT[t], C6AcylCoAMAT[t], C4AcylCoAMAT[t], CarMAT],
vcpt2C6 → CPT2[sfcpt2C6, Vcpt2, Kmcpt2C6AcylCarMAT, Kmcpt2C16AcylCarMAT,
   Kmcpt2C14AcylCarMAT, Kmcpt2C12AcylCarMAT, Kmcpt2C10AcylCarMAT, Kmcpt2C8AcylCarMAT,
   Kmcpt2C4AcylCarMAT, Kmcpt2CoAMAT, Kmcpt2C6AcylCoAMAT, Kmcpt2C16AcylCoAMAT,
   Kmcpt2C14AcylCoAMAT, Kmcpt2C12AcylCoAMAT, Kmcpt2C10AcylCoAMAT, Kmcpt2C8AcylCoAMAT,
   Kmcpt2C4AcylCoAMAT, Kmcpt2CarMAT, Keqcpt2, C6AcylCarMAT[t], C16AcylCarMAT[t],
   C14AcylCarMAT[t], C12AcylCarMAT[t], C10AcylCarMAT[t], C8AcylCarMAT[t],
   C4AcylCarMAT[t], CoAMAT, C6AcylCoAMAT[t], C16AcylCoAMAT[t], C14AcylCoAMAT[t],
   C12AcylCoAMAT[t], C10AcylCoAMAT[t], C8AcylCoAMAT[t], C4AcylCoAMAT[t], CarMAT],
vcpt2C4 → CPT2[sfcpt2C4, Vcpt2, Kmcpt2C4AcylCarMAT, Kmcpt2C16AcylCarMAT,
   Kmcpt2C14AcylCarMAT, Kmcpt2C12AcylCarMAT, Kmcpt2C10AcylCarMAT, Kmcpt2C8AcylCarMAT,
   Kmcpt2C6AcylCarMAT, Kmcpt2CoAMAT, Kmcpt2C4AcylCoAMAT, Kmcpt2C16AcylCoAMAT,
   Kmcpt2C14AcylCoAMAT, Kmcpt2C12AcylCoAMAT, Kmcpt2C10AcylCoAMAT, Kmcpt2C8AcylCoAMAT,
   Kmcpt2C6AcylCoAMAT, Kmcpt2CarMAT, Keqcpt2, C4AcylCarMAT[t], C16AcylCarMAT[t],
   C14AcylCarMAT[t], C12AcylCarMAT[t], C10AcylCarMAT[t], C8AcylCarMAT[t],
   C6AcylCarMAT[t], CoAMAT, C4AcylCoAMAT[t], C16AcylCoAMAT[t], C14AcylCoAMAT[t],
   C12AcylCoAMAT[t], C10AcylCoAMAT[t], C8AcylCoAMAT[t], C6AcylCoAMAT[t], CarMAT],
vvlcadC16 → VLCAD[sfvlcadC16, Vvlcad, KmvlcadC16AcylCoAMAT, KmvlcadC14AcylCoAMAT,
   KmvlcadC12AcylCoAMAT, KmvlcadFAD, KmvlcadC16EnoylCoAMAT,
   KmvlcadC14EnoylCoAMAT, KmvlcadC12EnoylCoAMAT, KmvlcadFADH, Keqvlcad,
   C16AcylCoAMAT[t], C14AcylCoAMAT[t], C12AcylCoAMAT[t], FADtMAT,
   C16EnoylCoAMAT[t], C14EnoylCoAMAT[t], C12EnoylCoAMAT[t], FADHMAT[t]],
vvlcadC14 → VLCAD[sfvlcadC14, Vvlcad, KmvlcadC14AcylCoAMAT, KmvlcadC16AcylCoAMAT,
   KmvlcadC12AcylCoAMAT, KmvlcadFAD, KmvlcadC14EnoylCoAMAT,
   KmvlcadC16EnoylCoAMAT, KmvlcadC12EnoylCoAMAT, KmvlcadFADH, Keqvlcad,
   C14AcylCoAMAT[t], C16AcylCoAMAT[t], C12AcylCoAMAT[t], FADtMAT,
   C14EnoylCoAMAT[t], C16EnoylCoAMAT[t], C12EnoylCoAMAT[t], FADHMAT[t]],
vvlcadC12 → VLCAD[sfvlcadC12, Vvlcad, KmvlcadC12AcylCoAMAT, KmvlcadC16AcylCoAMAT,
   KmvlcadC14AcylCoAMAT, KmvlcadFAD, KmvlcadC12EnoylCoAMAT,
   KmvlcadC16EnoylCoAMAT, KmvlcadC14EnoylCoAMAT, KmvlcadFADH, Keqvlcad,
   C12AcylCoAMAT[t], C16AcylCoAMAT[t], C14AcylCoAMAT[t], FADtMAT,
   C12EnoylCoAMAT[t], C16EnoylCoAMAT[t], C14EnoylCoAMAT[t], FADHMAT[t]],
```

```
vlcadC16 → LCAD[sflcadC16, Vlcad, KmlcadC16AcylCoAMAT, KmlcadC14AcylCoAMAT,
  KmlcadC12AcylCoAMAT, KmlcadC10AcylCoAMAT, KmlcadC8AcylCoAMAT, KmlcadFAD,
  KmlcadC16EnoylCoAMAT, KmlcadC14EnoylCoAMAT, KmlcadC12EnoylCoAMAT,
  KmlcadC10EnoylCoAMAT, KmlcadC8EnoylCoAMAT, KmlcadFADH, Keqlcad,
  C16AcylCoAMAT[t], C14AcylCoAMAT[t], C12AcylCoAMAT[t], C10AcylCoAMAT[t],
  C8AcylCoAMAT[t], FADtMAT, C16EnoylCoAMAT[t], C14EnoylCoAMAT[t],
  C12EnoylCoAMAT[t], C10EnoylCoAMAT[t], C8EnoylCoAMAT[t], FADHMAT[t]],
vlcadC14 → LCAD[sflcadC14, Vlcad, KmlcadC14AcylCoAMAT, KmlcadC16AcylCoAMAT,
  KmlcadC12AcylCoAMAT, KmlcadC10AcylCoAMAT, KmlcadC8AcylCoAMAT, KmlcadFAD,
  KmlcadC14EnoylCoAMAT, KmlcadC16EnoylCoAMAT, KmlcadC12EnoylCoAMAT,
  KmlcadC10EnoylCoAMAT, KmlcadC8EnoylCoAMAT, KmlcadFADH, Keqlcad,
  C14AcylCoAMAT[t], C16AcylCoAMAT[t], C12AcylCoAMAT[t], C10AcylCoAMAT[t],
  C8AcylCoAMAT[t], FADtMAT, C14EnoylCoAMAT[t], C16EnoylCoAMAT[t],
  C12EnoylCoAMAT[t], C10EnoylCoAMAT[t], C8EnoylCoAMAT[t], FADHMAT[t]],
vlcadC12 → LCAD[sflcadC12, Vlcad, KmlcadC12AcylCoAMAT, KmlcadC16AcylCoAMAT,
  KmlcadC14AcylCoAMAT, KmlcadC10AcylCoAMAT, KmlcadC8AcylCoAMAT, KmlcadFAD,
  KmlcadC12EnoylCoAMAT, KmlcadC16EnoylCoAMAT, KmlcadC14EnoylCoAMAT,
  KmlcadC10EnoylCoAMAT, KmlcadC8EnoylCoAMAT, KmlcadFADH, Keqlcad,
  C12AcylCoAMAT[t], C16AcylCoAMAT[t], C14AcylCoAMAT[t], C10AcylCoAMAT[t],
  C8AcylCoAMAT[t], FADtMAT, C14EnoylCoAMAT[t], C16EnoylCoAMAT[t],
  C14EnoylCoAMAT[t], C10EnoylCoAMAT[t], C8EnoylCoAMAT[t], FADHMAT[t]],
vlcadC10 → LCAD[sflcadC10, Vlcad, KmlcadC10AcylCoAMAT, KmlcadC16AcylCoAMAT,
  KmlcadC14AcylCoAMAT, KmlcadC12AcylCoAMAT, KmlcadC8AcylCoAMAT, KmlcadFAD,
  KmlcadC10EnoylCoAMAT, KmlcadC16EnoylCoAMAT, KmlcadC14EnoylCoAMAT,
  KmlcadC12EnoylCoAMAT, KmlcadC8EnoylCoAMAT, KmlcadFADH, Keqlcad,
  C10AcylCoAMAT[t], C16AcylCoAMAT[t], C14AcylCoAMAT[t], C12AcylCoAMAT[t],
  C8AcylCoAMAT[t], FADtMAT, C10EnoylCoAMAT[t], C16EnoylCoAMAT[t],
  C14EnoylCoAMAT[t], C12EnoylCoAMAT[t], C8EnoylCoAMAT[t], FADHMAT[t]],
vlcadC8 → LCAD[sflcadC8, Vlcad, KmlcadC8AcylCoAMAT, KmlcadC16AcylCoAMAT,
  KmlcadC14AcylCoAMAT, KmlcadC12AcylCoAMAT, KmlcadC10AcylCoAMAT, KmlcadFAD,
  KmlcadC8EnoylCoAMAT, KmlcadC16EnoylCoAMAT, KmlcadC14EnoylCoAMAT,
  KmlcadC12EnoylCoAMAT, KmlcadC10EnoylCoAMAT, KmlcadFADH, Keqlcad,
  C8AcylCoAMAT[t], C16AcylCoAMAT[t], C14AcylCoAMAT[t], C12AcylCoAMAT[t],
  C10AcylCoAMAT[t], FADtMAT, C8EnoylCoAMAT[t], C16EnoylCoAMAT[t],
  C14EnoylCoAMAT[t], C12EnoylCoAMAT[t], C10EnoylCoAMAT[t], FADHMAT[t]],
vmcadC12 → MCAD[sfmcadC12, Vmcad, KmmcadC12AcylCoAMAT, KmmcadC10AcylCoAMAT,
  KmmcadC8AcylCoAMAT, KmmcadC6AcylCoAMAT, KmmcadC4AcylCoAMAT, KmmcadFAD,
  KmmcadC12EnoylCoAMAT, KmmcadC10EnoylCoAMAT, KmmcadC8EnoylCoAMAT,
  KmmcadC6EnoylCoAMAT, KmmcadC4EnoylCoAMAT, KmmcadFADH, Keqmcad,
  C12AcylCoAMAT[t], C10AcylCoAMAT[t], C8AcylCoAMAT[t], C6AcylCoAMAT[t],
  C4AcylCoAMAT[t], FADtMAT, C12EnoylCoAMAT[t], C10EnoylCoAMAT[t],
  C8EnoylCoAMAT[t], C6EnoylCoAMAT[t], C4EnoylCoAMAT[t], FADHMAT[t]],
vmcadC10 → MCAD[sfmcadC10, Vmcad, KmmcadC10AcylCoAMAT, KmmcadC12AcylCoAMAT,
  KmmcadC8AcylCoAMAT, KmmcadC6AcylCoAMAT, KmmcadC4AcylCoAMAT, KmmcadFAD,
  KmmcadC10EnoylCoAMAT, KmmcadC12EnoylCoAMAT, KmmcadC8EnoylCoAMAT,
  KmmcadC6EnoylCoAMAT, KmmcadC4EnoylCoAMAT, KmmcadFADH, Keqmcad,
  C10AcylCoAMAT[t], C12AcylCoAMAT[t], C8AcylCoAMAT[t], C6AcylCoAMAT[t],
  C4AcylCoAMAT[t], FADtMAT, C10EnoylCoAMAT[t], C12EnoylCoAMAT[t],
  C8EnoylCoAMAT[t], C6EnoylCoAMAT[t], C4EnoylCoAMAT[t], FADHMAT[t]],
vmcadC8 → MCAD[sfmcadC8, Vmcad, KmmcadC8AcylCoAMAT, KmmcadC12AcylCoAMAT,
  KmmcadC10AcylCoAMAT, KmmcadC6AcylCoAMAT, KmmcadC4AcylCoAMAT, KmmcadFAD,
  KmmcadC8EnoylCoAMAT, KmmcadC12EnoylCoAMAT, KmmcadC10EnoylCoAMAT,
  KmmcadC6EnoylCoAMAT, KmmcadC4EnoylCoAMAT, KmmcadFADH, Keqmcad,
  C8AcylCoAMAT[t], C12AcylCoAMAT[t], C10AcylCoAMAT[t], C6AcylCoAMAT[t],
  C4AcylCoAMAT[t], FADtMAT, C8EnoylCoAMAT[t], C12EnoylCoAMAT[t],
```

```
    C10EnoylCoAMAT[t], C6EnoylCoAMAT[t], C4EnoylCoAMAT[t], FADHMAT[t]],
  vmcadC6 → MCAD[sfmcadC6, Vmcad, KmmcadC6AcylCoAMAT, KmmcadC12AcylCoAMAT,
    KmmcadC10AcylCoAMAT, KmmcadC8AcylCoAMAT, KmmcadC4AcylCoAMAT, KmmcadFAD,
    KmmcadC6EnoylCoAMAT, KmmcadC12EnoylCoAMAT, KmmcadC10EnoylCoAMAT,
    KmmcadC8EnoylCoAMAT, KmmcadC4EnoylCoAMAT, KmmcadFADH, Keqmcad,
    C6AcylCoAMAT[t], C12AcylCoAMAT[t], C10AcylCoAMAT[t], C8AcylCoAMAT[t],
    C4AcylCoAMAT[t], FADtMAT, C6EnoylCoAMAT[t], C12EnoylCoAMAT[t],
    C10EnoylCoAMAT[t], C8EnoylCoAMAT[t], C4EnoylCoAMAT[t], FADHMAT[t]],
  vmcadC4 → MCAD[sfmcadC4, Vmcad, KmmcadC4AcylCoAMAT, KmmcadC12AcylCoAMAT,
    KmmcadC10AcylCoAMAT, KmmcadC8AcylCoAMAT, KmmcadC6AcylCoAMAT, KmmcadFAD,
    KmmcadC4EnoylCoAMAT, KmmcadC12EnoylCoAMAT, KmmcadC10EnoylCoAMAT,
    KmmcadC8EnoylCoAMAT, KmmcadC6EnoylCoAMAT, KmmcadFADH, Keqmcad,
    C4AcylCoAMAT[t], C12AcylCoAMAT[t], C10AcylCoAMAT[t], C8AcylCoAMAT[t],
    C6AcylCoAMAT[t], FADtMAT, C4EnoylCoAMAT[t], C12EnoylCoAMAT[t],
    C10EnoylCoAMAT[t], C8EnoylCoAMAT[t], C6EnoylCoAMAT[t], FADHMAT[t]],
  vscadC6 → SCAD[sfscadC6, Vscad, KmscadC6AcylCoAMAT, KmscadC4AcylCoAMAT, KmscadFAD,
    KmscadC6EnoylCoAMAT, KmscadC4EnoylCoAMAT, KmscadFADH, Keqscad, C6AcylCoAMAT[t],
    C4AcylCoAMAT[t], FADtMAT, C6EnoylCoAMAT[t], C4EnoylCoAMAT[t], FADHMAT[t]],
  vscadC4 → SCAD[sfscadC4, Vscad, KmscadC4AcylCoAMAT, KmscadC6AcylCoAMAT, KmscadFAD,
    KmscadC4EnoylCoAMAT, KmscadC6EnoylCoAMAT, KmscadFADH, Keqscad, C4AcylCoAMAT[t],
    C6AcylCoAMAT[t], FADtMAT, C4EnoylCoAMAT[t], C6EnoylCoAMAT[t], FADHMAT[t]],
  vcrotC16 → CROT[sfcrotC16, Vcrot, KmcrotC16EnoylCoAMAT, KmcrotC14EnoylCoAMAT,
    KmcrotC12EnoylCoAMAT, KmcrotC10EnoylCoAMAT, KmcrotC8EnoylCoAMAT,
    KmcrotC6EnoylCoAMAT, KmcrotC4EnoylCoAMAT, KmcrotC16HydroxyacylCoAMAT,
    KmcrotC14HydroxyacylCoAMAT, KmcrotC12HydroxyacylCoAMAT,
    KmcrotC10HydroxyacylCoAMAT, KmcrotC8HydroxyacylCoAMAT,
    KmcrotC6HydroxyacylCoAMAT, KmcrotC4HydroxyacylCoAMAT, KicrotC4AcetoacylCoA,
    Keqcrot, C16EnoylCoAMAT[t], C14EnoylCoAMAT[t], C12EnoylCoAMAT[t],
    C10EnoylCoAMAT[t], C8EnoylCoAMAT[t], C6EnoylCoAMAT[t],
    C4EnoylCoAMAT[t], C16HydroxyacylCoAMAT[t], C14HydroxyacylCoAMAT[t],
    C12HydroxyacylCoAMAT[t], C10HydroxyacylCoAMAT[t], C8HydroxyacylCoAMAT[t],
    C6HydroxyacylCoAMAT[t], C4HydroxyacylCoAMAT[t], C4AcetoacylCoAMAT[t]],
  vcrotC14 → CROT[sfcrotC14, Vcrot, KmcrotC14EnoylCoAMAT, KmcrotC16EnoylCoAMAT,
    KmcrotC12EnoylCoAMAT, KmcrotC10EnoylCoAMAT, KmcrotC8EnoylCoAMAT,
    KmcrotC6EnoylCoAMAT, KmcrotC4EnoylCoAMAT, KmcrotC14HydroxyacylCoAMAT,
    KmcrotC16HydroxyacylCoAMAT, KmcrotC12HydroxyacylCoAMAT,
    KmcrotC10HydroxyacylCoAMAT, KmcrotC8HydroxyacylCoAMAT,
    KmcrotC6HydroxyacylCoAMAT, KmcrotC4HydroxyacylCoAMAT, KicrotC4AcetoacylCoA,
    Keqcrot, C14EnoylCoAMAT[t], C16EnoylCoAMAT[t], C12EnoylCoAMAT[t],
    C10EnoylCoAMAT[t], C8EnoylCoAMAT[t], C6EnoylCoAMAT[t],
    C4EnoylCoAMAT[t], C14HydroxyacylCoAMAT[t], C16HydroxyacylCoAMAT[t],
    C12HydroxyacylCoAMAT[t], C10HydroxyacylCoAMAT[t], C8HydroxyacylCoAMAT[t],
    C6HydroxyacylCoAMAT[t], C4HydroxyacylCoAMAT[t], C4AcetoacylCoAMAT[t]],
  vcrotC12 → CROT[sfcrotC12, Vcrot, KmcrotC12EnoylCoAMAT, KmcrotC16EnoylCoAMAT,
    KmcrotC14EnoylCoAMAT, KmcrotC10EnoylCoAMAT, KmcrotC8EnoylCoAMAT,
    KmcrotC6EnoylCoAMAT, KmcrotC4EnoylCoAMAT, KmcrotC12HydroxyacylCoAMAT,
    KmcrotC16HydroxyacylCoAMAT, KmcrotC14HydroxyacylCoAMAT,
    KmcrotC10HydroxyacylCoAMAT, KmcrotC8HydroxyacylCoAMAT,
    KmcrotC6HydroxyacylCoAMAT, KmcrotC4HydroxyacylCoAMAT, KicrotC4AcetoacylCoA,
    Keqcrot, C12EnoylCoAMAT[t], C16EnoylCoAMAT[t], C14EnoylCoAMAT[t],
    C10EnoylCoAMAT[t], C8EnoylCoAMAT[t], C6EnoylCoAMAT[t],
    C4EnoylCoAMAT[t], C12HydroxyacylCoAMAT[t], C16HydroxyacylCoAMAT[t],
    C14HydroxyacylCoAMAT[t], C10HydroxyacylCoAMAT[t], C8HydroxyacylCoAMAT[t],
    C6HydroxyacylCoAMAT[t], C4HydroxyacylCoAMAT[t], C4AcetoacylCoAMAT[t]],
  vcrotC10 → CROT[sfcrotC10, Vcrot, KmcrotC10EnoylCoAMAT, KmcrotC16EnoylCoAMAT,
```

```
    KmcrotC14EnoylCoAMAT, KmcrotC12EnoylCoAMAT, KmcrotC8EnoylCoAMAT,
    KmcrotC6EnoylCoAMAT, KmcrotC4EnoylCoAMAT, KmcrotC10HydroxyacylCoAMAT,
    KmcrotC16HydroxyacylCoAMAT, KmcrotC14HydroxyacylCoAMAT,
    KmcrotC12HydroxyacylCoAMAT, KmcrotC8HydroxyacylCoAMAT,
    KmcrotC6HydroxyacylCoAMAT, KmcrotC4HydroxyacylCoAMAT, KicrotC4AcetoacylCoA,
    Keqcrot, C10EnoylCoAMAT[t], C16EnoylCoAMAT[t], C14EnoylCoAMAT[t],
    C12EnoylCoAMAT[t], C8EnoylCoAMAT[t], C6EnoylCoAMAT[t],
    C4EnoylCoAMAT[t], C10HydroxyacylCoAMAT[t], C16HydroxyacylCoAMAT[t],
    C14HydroxyacylCoAMAT[t], C12HydroxyacylCoAMAT[t], C8HydroxyacylCoAMAT[t],
    C6HydroxyacylCoAMAT[t], C4HydroxyacylCoAMAT[t], C4AcetoacylCoAMAT[t]],
  vcrotC8 → CROT[sfcrotC8, Vcrot, KmcrotC8EnoylCoAMAT, KmcrotC16EnoylCoAMAT,
    KmcrotC14EnoylCoAMAT, KmcrotC12EnoylCoAMAT, KmcrotC10EnoylCoAMAT,
    KmcrotC6EnoylCoAMAT, KmcrotC4EnoylCoAMAT, KmcrotC8HydroxyacylCoAMAT,
    KmcrotC16HydroxyacylCoAMAT, KmcrotC14HydroxyacylCoAMAT,
    KmcrotC12HydroxyacylCoAMAT, KmcrotC10HydroxyacylCoAMAT,
    KmcrotC6HydroxyacylCoAMAT, KmcrotC4HydroxyacylCoAMAT, KicrotC4AcetoacylCoA,
    Keqcrot, C8EnoylCoAMAT[t], C16EnoylCoAMAT[t], C14EnoylCoAMAT[t],
    C12EnoylCoAMAT[t], C10EnoylCoAMAT[t], C6EnoylCoAMAT[t],
    C4EnoylCoAMAT[t], C8HydroxyacylCoAMAT[t], C16HydroxyacylCoAMAT[t],
    C14HydroxyacylCoAMAT[t], C12HydroxyacylCoAMAT[t], C10HydroxyacylCoAMAT[t],
    C6HydroxyacylCoAMAT[t], C4HydroxyacylCoAMAT[t], C4AcetoacylCoAMAT[t]],
  vcrotC6 → CROT[sfcrotC6, Vcrot, KmcrotC6EnoylCoAMAT, KmcrotC16EnoylCoAMAT,
    KmcrotC14EnoylCoAMAT, KmcrotC12EnoylCoAMAT, KmcrotC10EnoylCoAMAT,
    KmcrotC8EnoylCoAMAT, KmcrotC4EnoylCoAMAT, KmcrotC6HydroxyacylCoAMAT,
    KmcrotC16HydroxyacylCoAMAT, KmcrotC14HydroxyacylCoAMAT,
    KmcrotC12HydroxyacylCoAMAT, KmcrotC10HydroxyacylCoAMAT,
    KmcrotC8HydroxyacylCoAMAT, KmcrotC4HydroxyacylCoAMAT, KicrotC4AcetoacylCoA,
    Keqcrot, C6EnoylCoAMAT[t], C16EnoylCoAMAT[t], C14EnoylCoAMAT[t],
    C12EnoylCoAMAT[t], C10EnoylCoAMAT[t], C8EnoylCoAMAT[t],
    C4EnoylCoAMAT[t], C6HydroxyacylCoAMAT[t], C16HydroxyacylCoAMAT[t],
    C14HydroxyacylCoAMAT[t], C12HydroxyacylCoAMAT[t], C10HydroxyacylCoAMAT[t],
    C8HydroxyacylCoAMAT[t], C4HydroxyacylCoAMAT[t], C4AcetoacylCoAMAT[t]],
  vcrotC4 → CROT[sfcrotC4, Vcrot, KmcrotC4EnoylCoAMAT, KmcrotC16EnoylCoAMAT,
    KmcrotC14EnoylCoAMAT, KmcrotC12EnoylCoAMAT, KmcrotC10EnoylCoAMAT,
    KmcrotC8EnoylCoAMAT, KmcrotC6EnoylCoAMAT, KmcrotC4HydroxyacylCoAMAT,
    KmcrotC16HydroxyacylCoAMAT, KmcrotC14HydroxyacylCoAMAT,
    KmcrotC12HydroxyacylCoAMAT, KmcrotC10HydroxyacylCoAMAT,
    KmcrotC8HydroxyacylCoAMAT, KmcrotC6HydroxyacylCoAMAT, KicrotC4AcetoacylCoA,
    Keqcrot, C4EnoylCoAMAT[t], C16EnoylCoAMAT[t], C14EnoylCoAMAT[t],
    C12EnoylCoAMAT[t], C10EnoylCoAMAT[t], C8EnoylCoAMAT[t],
    C6EnoylCoAMAT[t], C4HydroxyacylCoAMAT[t], C16HydroxyacylCoAMAT[t],
    C14HydroxyacylCoAMAT[t], C12HydroxyacylCoAMAT[t], C10HydroxyacylCoAMAT[t],
    C8HydroxyacylCoAMAT[t], C6HydroxyacylCoAMAT[t], C4AcetoacylCoAMAT[t]],
  vmschadC16 → MSCHAD[sfmschadC16, Vmschad, KmmschadC16HydroxyacylCoAMAT,
    KmmschadC14HydroxyacylCoAMAT, KmmschadC12HydroxyacylCoAMAT,
    KmmschadC10HydroxyacylCoAMAT, KmmschadC8HydroxyacylCoAMAT,
    KmmschadC6HydroxyacylCoAMAT, KmmschadC4HydroxyacylCoAMAT,
    KmmschadNADMAT, KmmschadC16KetoacylCoAMAT, KmmschadC14KetoacylCoAMAT,
    KmmschadC12KetoacylCoAMAT, KmmschadC10KetoacylCoAMAT, KmmschadC8KetoacylCoAMAT,
    KmmschadC6KetoacylCoAMAT, KmmschadC4AcetoacylCoAMAT, KmmschadNADHMAT,
    Keqmschad, C16HydroxyacylCoAMAT[t], C14HydroxyacylCoAMAT[t],
    C12HydroxyacylCoAMAT[t], C10HydroxyacylCoAMAT[t], C8HydroxyacylCoAMAT[t],
    C6HydroxyacylCoAMAT[t], C4HydroxyacylCoAMAT[t], NADtMAT, C16KetoacylCoAMAT[t],
    C14KetoacylCoAMAT[t], C12KetoacylCoAMAT[t], C10KetoacylCoAMAT[t],
    C8KetoacylCoAMAT[t], C6KetoacylCoAMAT[t], C4AcetoacylCoAMAT[t], NADHMAT[t]],
```

```
vmschadC14 → MSCHAD[sfmschadC14, Vmschad, KmmschadC14HydroxyacylCoAMAT,
   KmmschadC16HydroxyacylCoAMAT, KmmschadC12HydroxyacylCoAMAT,
   KmmschadC10HydroxyacylCoAMAT, KmmschadC8HydroxyacylCoAMAT,
   KmmschadC6HydroxyacylCoAMAT, KmmschadC4HydroxyacylCoAMAT,
   KmmschadNADMAT, KmmschadC14KetoacylCoAMAT, KmmschadC16KetoacylCoAMAT,
   KmmschadC12KetoacylCoAMAT, KmmschadC10KetoacylCoAMAT, KmmschadC8KetoacylCoAMAT,
   KmmschadC6KetoacylCoAMAT, KmmschadC4AcetoacylCoAMAT, KmmschadNADHMAT,
   Keqmschad, C14HydroxyacylCoAMAT[t], C16HydroxyacylCoAMAT[t],
   C12HydroxyacylCoAMAT[t], C10HydroxyacylCoAMAT[t], C8HydroxyacylCoAMAT[t],
   C6HydroxyacylCoAMAT[t], C4HydroxyacylCoAMAT[t], NADtMAT, C14KetoacylCoAMAT[t],
   C16KetoacylCoAMAT[t], C12KetoacylCoAMAT[t], C10KetoacylCoAMAT[t],
   C8KetoacylCoAMAT[t], C6KetoacylCoAMAT[t], C4AcetoacylCoAMAT[t], NADHMAT[t]],
vmschadC12 → MSCHAD[sfmschadC12, Vmschad, KmmschadC12HydroxyacylCoAMAT,
   KmmschadC16HydroxyacylCoAMAT, KmmschadC14HydroxyacylCoAMAT,
   KmmschadC10HydroxyacylCoAMAT, KmmschadC8HydroxyacylCoAMAT,
   KmmschadC6HydroxyacylCoAMAT, KmmschadC4HydroxyacylCoAMAT,
   KmmschadNADMAT, KmmschadC12KetoacylCoAMAT, KmmschadC16KetoacylCoAMAT,
   KmmschadC14KetoacylCoAMAT, KmmschadC10KetoacylCoAMAT, KmmschadC8KetoacylCoAMAT,
   KmmschadC6KetoacylCoAMAT, KmmschadC4AcetoacylCoAMAT, KmmschadNADHMAT,
   Keqmschad, C12HydroxyacylCoAMAT[t], C16HydroxyacylCoAMAT[t],
   C14HydroxyacylCoAMAT[t], C10HydroxyacylCoAMAT[t], C8HydroxyacylCoAMAT[t],
   C6HydroxyacylCoAMAT[t], C4HydroxyacylCoAMAT[t], NADtMAT, C12KetoacylCoAMAT[t],
   C16KetoacylCoAMAT[t], C14KetoacylCoAMAT[t], C10KetoacylCoAMAT[t],
   C8KetoacylCoAMAT[t], C6KetoacylCoAMAT[t], C4AcetoacylCoAMAT[t], NADHMAT[t]],
vmschadC10 → MSCHAD[sfmschadC10, Vmschad, KmmschadC10HydroxyacylCoAMAT,
   KmmschadC16HydroxyacylCoAMAT, KmmschadC14HydroxyacylCoAMAT,
   KmmschadC12HydroxyacylCoAMAT, KmmschadC8HydroxyacylCoAMAT,
   KmmschadC6HydroxyacylCoAMAT, KmmschadC4HydroxyacylCoAMAT,
   KmmschadNADMAT, KmmschadC10KetoacylCoAMAT, KmmschadC16KetoacylCoAMAT,
   KmmschadC14KetoacylCoAMAT, KmmschadC12KetoacylCoAMAT, KmmschadC8KetoacylCoAMAT,
   KmmschadC6KetoacylCoAMAT, KmmschadC4AcetoacylCoAMAT, KmmschadNADHMAT,
   Keqmschad, C10HydroxyacylCoAMAT[t], C16HydroxyacylCoAMAT[t],
   C14HydroxyacylCoAMAT[t], C12HydroxyacylCoAMAT[t], C8HydroxyacylCoAMAT[t],
   C6HydroxyacylCoAMAT[t], C4HydroxyacylCoAMAT[t], NADtMAT, C10KetoacylCoAMAT[t],
   C16KetoacylCoAMAT[t], C14KetoacylCoAMAT[t], C12KetoacylCoAMAT[t],
   C8KetoacylCoAMAT[t], C6KetoacylCoAMAT[t], C4AcetoacylCoAMAT[t], NADHMAT[t]],
vmschadC8 → MSCHAD[sfmschadC8, Vmschad, KmmschadC8HydroxyacylCoAMAT,
   KmmschadC16HydroxyacylCoAMAT, KmmschadC14HydroxyacylCoAMAT,
   KmmschadC12HydroxyacylCoAMAT, KmmschadC10HydroxyacylCoAMAT,
   KmmschadC6HydroxyacylCoAMAT, KmmschadC4HydroxyacylCoAMAT,
   KmmschadNADMAT, KmmschadC8KetoacylCoAMAT, KmmschadC16KetoacylCoAMAT,
   KmmschadC14KetoacylCoAMAT, KmmschadC12KetoacylCoAMAT, KmmschadC10KetoacylCoAMAT,
   KmmschadC6KetoacylCoAMAT, KmmschadC4AcetoacylCoAMAT, KmmschadNADHMAT,
   Keqmschad, C8HydroxyacylCoAMAT[t], C16HydroxyacylCoAMAT[t],
   C14HydroxyacylCoAMAT[t], C12HydroxyacylCoAMAT[t], C10HydroxyacylCoAMAT[t],
   C6HydroxyacylCoAMAT[t], C4HydroxyacylCoAMAT[t], NADtMAT, C8KetoacylCoAMAT[t],
   C16KetoacylCoAMAT[t], C14KetoacylCoAMAT[t], C12KetoacylCoAMAT[t],
   C10KetoacylCoAMAT[t], C6KetoacylCoAMAT[t], C4AcetoacylCoAMAT[t], NADHMAT[t]],
vmschadC6 → MSCHAD[sfmschadC6, Vmschad, KmmschadC6HydroxyacylCoAMAT,
   KmmschadC16HydroxyacylCoAMAT, KmmschadC14HydroxyacylCoAMAT,
   KmmschadC12HydroxyacylCoAMAT, KmmschadC10HydroxyacylCoAMAT,
   KmmschadC8HydroxyacylCoAMAT, KmmschadC4HydroxyacylCoAMAT,
   KmmschadNADMAT, KmmschadC6KetoacylCoAMAT, KmmschadC16KetoacylCoAMAT,
   KmmschadC14KetoacylCoAMAT, KmmschadC12KetoacylCoAMAT, KmmschadC10KetoacylCoAMAT,
   KmmschadC8KetoacylCoAMAT, KmmschadC4AcetoacylCoAMAT, KmmschadNADHMAT,
```

```
    Keqmschad, C6HydroxyacylCoAMAT[t], C16HydroxyacylCoAMAT[t],
    C14HydroxyacylCoAMAT[t], C12HydroxyacylCoAMAT[t], C10HydroxyacylCoAMAT[t],
    C8HydroxyacylCoAMAT[t], C4HydroxyacylCoAMAT[t], NADtMAT, C6KetoacylCoAMAT[t],
    C16KetoacylCoAMAT[t], C14KetoacylCoAMAT[t], C12KetoacylCoAMAT[t],
    C10KetoacylCoAMAT[t], C8KetoacylCoAMAT[t], C4AcetoacylCoAMAT[t], NADHMAT[t]],
  vmschadC4 → MSCHAD[sfmschadC4, Vmschad, KmmschadC4HydroxyacylCoAMAT,
    KmmschadC16HydroxyacylCoAMAT, KmmschadC14HydroxyacylCoAMAT,
    KmmschadC12HydroxyacylCoAMAT, KmmschadC10HydroxyacylCoAMAT,
    KmmschadC8HydroxyacylCoAMAT, KmmschadC6HydroxyacylCoAMAT,
    KmmschadNADMAT, KmmschadC4AcetoacylCoAMAT, KmmschadC16KetoacylCoAMAT,
    KmmschadC14KetoacylCoAMAT, KmmschadC12KetoacylCoAMAT, KmmschadC10KetoacylCoAMAT,
    KmmschadC8KetoacylCoAMAT, KmmschadC6KetoacylCoAMAT, KmmschadNADHMAT,
    Keqmschad, C4HydroxyacylCoAMAT[t], C16HydroxyacylCoAMAT[t],
    C14HydroxyacylCoAMAT[t], C12HydroxyacylCoAMAT[t], C10HydroxyacylCoAMAT[t],
    C8HydroxyacylCoAMAT[t], C6HydroxyacylCoAMAT[t], NADtMAT, C4AcetoacylCoAMAT[t],
    C16KetoacylCoAMAT[t], C14KetoacylCoAMAT[t], C12KetoacylCoAMAT[t],
    C10KetoacylCoAMAT[t], C8KetoacylCoAMAT[t], C6KetoacylCoAMAT[t], NADHMAT[t]],
  vmckatC16 → MCKATA[sfmckatC16, Vmckat, KmmckatC16KetoacylCoAMAT,
    KmmckatC14KetoacylCoAMAT, KmmckatC12KetoacylCoAMAT,
    KmmckatC10KetoacylCoAMAT, KmmckatC8KetoacylCoAMAT, KmmckatC6KetoacylCoAMAT,
    KmmckatC4AcetoacylCoAMAT, KmmckatCoAMAT, KmmckatC14AcylCoAMAT,
    KmmckatC16AcylCoAMAT, KmmckatC12AcylCoAMAT, KmmckatC10AcylCoAMAT,
    KmmckatC8AcylCoAMAT, KmmckatC6AcylCoAMAT, KmmckatC4AcylCoAMAT,
    KmmckatAcetylCoAMAT, Keqmckat, C16KetoacylCoAMAT[t], C14KetoacylCoAMAT[t],
    C12KetoacylCoAMAT[t], C10KetoacylCoAMAT[t], C8KetoacylCoAMAT[t],
    C6KetoacylCoAMAT[t], C4AcetoacylCoAMAT[t], CoAMAT, C14AcylCoAMAT[t],
    C16AcylCoAMAT[t], C12AcylCoAMAT[t], C10AcylCoAMAT[t], C8AcylCoAMAT[t],
    C6AcylCoAMAT[t], C4AcylCoAMAT[t], AcetylCoAMAT[t], CE1, KmCE1, nE1],
  vmckatC14 → MCKATA[sfmckatC14, Vmckat, KmmckatC14KetoacylCoAMAT,
    KmmckatC16KetoacylCoAMAT, KmmckatC12KetoacylCoAMAT,
    KmmckatC10KetoacylCoAMAT, KmmckatC8KetoacylCoAMAT, KmmckatC6KetoacylCoAMAT,
    KmmckatC4AcetoacylCoAMAT, KmmckatCoAMAT, KmmckatC12AcylCoAMAT,
    KmmckatC16AcylCoAMAT, KmmckatC14AcylCoAMAT, KmmckatC10AcylCoAMAT,
    KmmckatC8AcylCoAMAT, KmmckatC6AcylCoAMAT, KmmckatC4AcylCoAMAT,
    KmmckatAcetylCoAMAT, Keqmckat, C14KetoacylCoAMAT[t], C16KetoacylCoAMAT[t],
    C12KetoacylCoAMAT[t], C10KetoacylCoAMAT[t], C8KetoacylCoAMAT[t],
    C6KetoacylCoAMAT[t], C4AcetoacylCoAMAT[t], CoAMAT, C12AcylCoAMAT[t],
    C16AcylCoAMAT[t], C14AcylCoAMAT[t], C10AcylCoAMAT[t], C8AcylCoAMAT[t],
    C6AcylCoAMAT[t], C4AcylCoAMAT[t], AcetylCoAMAT[t], CE1, KmCE1, nE1],
  vmckatC12 → MCKATA[sfmckatC12, Vmckat, KmmckatC12KetoacylCoAMAT,
    KmmckatC16KetoacylCoAMAT, KmmckatC14KetoacylCoAMAT,
    KmmckatC10KetoacylCoAMAT, KmmckatC8KetoacylCoAMAT, KmmckatC6KetoacylCoAMAT,
    KmmckatC4AcetoacylCoAMAT, KmmckatCoAMAT, KmmckatC10AcylCoAMAT,
    KmmckatC16AcylCoAMAT, KmmckatC14AcylCoAMAT, KmmckatC12AcylCoAMAT,
    KmmckatC8AcylCoAMAT, KmmckatC6AcylCoAMAT, KmmckatC4AcylCoAMAT,
    KmmckatAcetylCoAMAT, Keqmckat, C12KetoacylCoAMAT[t], C16KetoacylCoAMAT[t],
    C14KetoacylCoAMAT[t], C10KetoacylCoAMAT[t], C8KetoacylCoAMAT[t],
    C6KetoacylCoAMAT[t], C4AcetoacylCoAMAT[t], CoAMAT, C10AcylCoAMAT[t],
    C16AcylCoAMAT[t], C14AcylCoAMAT[t], C12AcylCoAMAT[t], C8AcylCoAMAT[t],
    C6AcylCoAMAT[t], C4AcylCoAMAT[t], AcetylCoAMAT[t], CE1, KmCE1, nE1],
  vmckatC10 → MCKATA[sfmckatC10, Vmckat, KmmckatC10KetoacylCoAMAT,
    KmmckatC16KetoacylCoAMAT, KmmckatC14KetoacylCoAMAT,
    KmmckatC12KetoacylCoAMAT, KmmckatC8KetoacylCoAMAT, KmmckatC6KetoacylCoAMAT,
    KmmckatC4AcetoacylCoAMAT, KmmckatCoAMAT, KmmckatC8AcylCoAMAT,
    KmmckatC16AcylCoAMAT, KmmckatC14AcylCoAMAT, KmmckatC12AcylCoAMAT,
```

```
    KmmckatC10AcylCoAMAT, KmmckatC6AcylCoAMAT, KmmckatC4AcylCoAMAT,
    KmmckatAcetylCoAMAT, Keqmckat, C10KetoacylCoAMAT[t], C16KetoacylCoAMAT[t],
    C14KetoacylCoAMAT[t], C12KetoacylCoAMAT[t], C8KetoacylCoAMAT[t],
    C6KetoacylCoAMAT[t], C4AcetoacylCoAMAT[t], CoAMAT, C8AcylCoAMAT[t],
    C16AcylCoAMAT[t], C14AcylCoAMAT[t], C12AcylCoAMAT[t], C10AcylCoAMAT[t],
    C6AcylCoAMAT[t], C4AcylCoAMAT[t], AcetylCoAMAT[t], CE1, KmCE1, nE1],
  vmckatC8 → MCKATA[sfmckatC8, Vmckat, KmmckatC8KetoacylCoAMAT,
    KmmckatC16KetoacylCoAMAT, KmmckatC14KetoacylCoAMAT,
    KmmckatC12KetoacylCoAMAT, KmmckatC10KetoacylCoAMAT, KmmckatC6KetoacylCoAMAT,
    KmmckatC4AcetoacylCoAMAT, KmmckatCoAMAT, KmmckatC6AcylCoAMAT,
    KmmckatC16AcylCoAMAT, KmmckatC14AcylCoAMAT, KmmckatC12AcylCoAMAT,
    KmmckatC10AcylCoAMAT, KmmckatC8AcylCoAMAT, KmmckatC4AcylCoAMAT,
    KmmckatAcetylCoAMAT, Keqmckat, C8KetoacylCoAMAT[t], C16KetoacylCoAMAT[t],
    C14KetoacylCoAMAT[t], C12KetoacylCoAMAT[t], C10KetoacylCoAMAT[t],
    C6KetoacylCoAMAT[t], C4AcetoacylCoAMAT[t], CoAMAT, C6AcylCoAMAT[t],
    C16AcylCoAMAT[t], C14AcylCoAMAT[t], C12AcylCoAMAT[t], C10AcylCoAMAT[t],
    C8AcylCoAMAT[t], C4AcylCoAMAT[t], AcetylCoAMAT[t], CE1, KmCE1, nE1],
  vmckatC6 → MCKATA[sfmckatC6, Vmckat, KmmckatC6KetoacylCoAMAT,
    KmmckatC16KetoacylCoAMAT, KmmckatC14KetoacylCoAMAT,
    KmmckatC12KetoacylCoAMAT, KmmckatC10KetoacylCoAMAT, KmmckatC8KetoacylCoAMAT,
    KmmckatC4AcetoacylCoAMAT, KmmckatCoAMAT, KmmckatC4AcylCoAMAT,
    KmmckatC16AcylCoAMAT, KmmckatC14AcylCoAMAT, KmmckatC12AcylCoAMAT,
    KmmckatC10AcylCoAMAT, KmmckatC8AcylCoAMAT, KmmckatC6AcylCoAMAT,
    KmmckatAcetylCoAMAT, Keqmckat, C6KetoacylCoAMAT[t], C16KetoacylCoAMAT[t],
    C14KetoacylCoAMAT[t], C12KetoacylCoAMAT[t], C10KetoacylCoAMAT[t],
    C8KetoacylCoAMAT[t], C4AcetoacylCoAMAT[t], CoAMAT, C4AcylCoAMAT[t],
    C16AcylCoAMAT[t], C14AcylCoAMAT[t], C12AcylCoAMAT[t], C10AcylCoAMAT[t],
    C8AcylCoAMAT[t], C6AcylCoAMAT[t], AcetylCoAMAT[t], CE1, KmCE1, nE1],
  vmckatC4 → MCKATB[sfmckatC4, Vmckat, KmmckatC4AcetoacylCoAMAT,
    KmmckatC16KetoacylCoAMAT, KmmckatC14KetoacylCoAMAT,
    KmmckatC12KetoacylCoAMAT, KmmckatC10KetoacylCoAMAT, KmmckatC8KetoacylCoAMAT,
    KmmckatC6KetoacylCoAMAT, KmmckatCoAMAT, KmmckatC4AcylCoAMAT,
    KmmckatC16AcylCoAMAT, KmmckatC14AcylCoAMAT, KmmckatC12AcylCoAMAT,
    KmmckatC10AcylCoAMAT, KmmckatC8AcylCoAMAT, KmmckatC6AcylCoAMAT,
    KmmckatAcetylCoAMAT, Keqmckat, C4AcetoacylCoAMAT[t], C16KetoacylCoAMAT[t],
    C14KetoacylCoAMAT[t], C12KetoacylCoAMAT[t], C10KetoacylCoAMAT[t],
    C8KetoacylCoAMAT[t], C6KetoacylCoAMAT[t], CoAMAT, C4AcylCoAMAT[t],
    C16AcylCoAMAT[t], C14AcylCoAMAT[t], C12AcylCoAMAT[t], C10AcylCoAMAT[t],
    C8AcylCoAMAT[t], C6AcylCoAMAT[t], AcetylCoAMAT[t], CE1, KmCE1, nE1],
  vmtpC16 → MTP[sfmtpC16, Vmtp, KmmtpC16EnoylCoAMAT, KmmtpC14EnoylCoAMAT,
    KmmtpC12EnoylCoAMAT, KmmtpC10EnoylCoAMAT, KmmtpC8EnoylCoAMAT,
    KmmtpNADMAT, KmmtpCoAMAT, KmmtpC14AcylCoAMAT, KmmtpC16AcylCoAMAT,
    KmmtpC12AcylCoAMAT, KmmtpC10AcylCoAMAT, KmmtpC8AcylCoAMAT,
    KmmtpC6AcylCoAMAT, KmmtpNADHMAT, KmmtpAcetylCoAMAT, KicrotC4AcetoacylCoA,
    Keqmtp, C16EnoylCoAMAT[t], C14EnoylCoAMAT[t], C12EnoylCoAMAT[t],
    C10EnoylCoAMAT[t], C8EnoylCoAMAT[t], NADtMAT, CoAMAT, C14AcylCoAMAT[t],
    C16AcylCoAMAT[t], C12AcylCoAMAT[t], C10AcylCoAMAT[t], C8AcylCoAMAT[t],
    C6AcylCoAMAT[t], NADHMAT[t], AcetylCoAMAT[t], C4AcetoacylCoAMAT[t]],
  vmtpC14 → MTP[sfmtpC14, Vmtp, KmmtpC14EnoylCoAMAT, KmmtpC16EnoylCoAMAT,
    KmmtpC12EnoylCoAMAT, KmmtpC10EnoylCoAMAT, KmmtpC8EnoylCoAMAT,
    KmmtpNADMAT, KmmtpCoAMAT, KmmtpC12AcylCoAMAT, KmmtpC16AcylCoAMAT,
    KmmtpC14AcylCoAMAT, KmmtpC10AcylCoAMAT, KmmtpC8AcylCoAMAT,
    KmmtpC6AcylCoAMAT, KmmtpNADHMAT, KmmtpAcetylCoAMAT, KicrotC4AcetoacylCoA,
    Keqmtp, C14EnoylCoAMAT[t], C16EnoylCoAMAT[t], C12EnoylCoAMAT[t],
    C10EnoylCoAMAT[t], C8EnoylCoAMAT[t], NADtMAT, CoAMAT, C12AcylCoAMAT[t],
```

```
    C16AcylCoAMAT[t], C14AcylCoAMAT[t], C10AcylCoAMAT[t], C8AcylCoAMAT[t],
    C6AcylCoAMAT[t], NADHMAT[t], AcetylCoAMAT[t], C4AcetoacylCoAMAT[t]],
  vmtpC12 → MTP[sfmtpC12, Vmtp, KmmtpC12EnoylCoAMAT, KmmtpC16EnoylCoAMAT,
    KmmtpC14EnoylCoAMAT, KmmtpC10EnoylCoAMAT, KmmtpC8EnoylCoAMAT,
    KmmtpNADMAT, KmmtpCoAMAT, KmmtpC10AcylCoAMAT, KmmtpC16AcylCoAMAT,
    KmmtpC14AcylCoAMAT, KmmtpC12AcylCoAMAT, KmmtpC8AcylCoAMAT,
    KmmtpC6AcylCoAMAT, KmmtpNADHMAT, KmmtpAcetylCoAMAT, KicrotC4AcetoacylCoA,
    Keqmtp, C12EnoylCoAMAT[t], C16EnoylCoAMAT[t], C14EnoylCoAMAT[t],
    C10EnoylCoAMAT[t], C8EnoylCoAMAT[t], NADtMAT, CoAMAT, C10AcylCoAMAT[t],
    C16AcylCoAMAT[t], C14AcylCoAMAT[t], C12AcylCoAMAT[t], C8AcylCoAMAT[t],
    C6AcylCoAMAT[t], NADHMAT[t], AcetylCoAMAT[t], C4AcetoacylCoAMAT[t]],
  vmtpC10 → MTP[sfmtpC10, Vmtp, KmmtpC10EnoylCoAMAT, KmmtpC16EnoylCoAMAT,
    KmmtpC14EnoylCoAMAT, KmmtpC12EnoylCoAMAT, KmmtpC8EnoylCoAMAT,
    KmmtpNADMAT, KmmtpCoAMAT, KmmtpC8AcylCoAMAT, KmmtpC16AcylCoAMAT,
    KmmtpC14AcylCoAMAT, KmmtpC12AcylCoAMAT, KmmtpC10AcylCoAMAT,
    KmmtpC6AcylCoAMAT, KmmtpNADHMAT, KmmtpAcetylCoAMAT, KicrotC4AcetoacylCoA,
    Keqmtp, C10EnoylCoAMAT[t], C16EnoylCoAMAT[t], C14EnoylCoAMAT[t],
    C12EnoylCoAMAT[t], C8EnoylCoAMAT[t], NADtMAT, CoAMAT, C8AcylCoAMAT[t],
    C16AcylCoAMAT[t], C14AcylCoAMAT[t], C12AcylCoAMAT[t], C10AcylCoAMAT[t],
    C6AcylCoAMAT[t], NADHMAT[t], AcetylCoAMAT[t], C4AcetoacylCoAMAT[t]],
  vmtpC8 → MTP[sfmtpC8, Vmtp, KmmtpC8EnoylCoAMAT, KmmtpC16EnoylCoAMAT,
    KmmtpC14EnoylCoAMAT, KmmtpC12EnoylCoAMAT, KmmtpC10EnoylCoAMAT,
    KmmtpNADMAT, KmmtpCoAMAT, KmmtpC6AcylCoAMAT, KmmtpC16AcylCoAMAT,
    KmmtpC14AcylCoAMAT, KmmtpC12AcylCoAMAT, KmmtpC10AcylCoAMAT,
    KmmtpC8AcylCoAMAT, KmmtpNADHMAT, KmmtpAcetylCoAMAT, KicrotC4AcetoacylCoA,
    Keqmtp, C8EnoylCoAMAT[t], C16EnoylCoAMAT[t], C14EnoylCoAMAT[t],
    C12EnoylCoAMAT[t], C10EnoylCoAMAT[t], NADtMAT, CoAMAT, C6AcylCoAMAT[t],
    C16AcylCoAMAT[t], C14AcylCoAMAT[t], C12AcylCoAMAT[t], C10AcylCoAMAT[t],
    C8AcylCoAMAT[t], NADHMAT[t], AcetylCoAMAT[t], C4AcetoacylCoAMAT[t]],
  vacotC16 → ACOT[sfacotC16, Vmacot, KmacotC16CoA, KmacotC14CoA, KmacotC12CoA,
    KmacotC10CoA, KmacotC8CoA, KmacotC6CoA, KmacotC4CoA, KmCoA, KmacotC16FFA,
    KmacotC14FFA, KmacotC12FFA, KmacotC10FFA, KmacotC8FFA, KmacotC6FFA,
    KmacotC4FFA, KeqacotC16, C16AcylCoAMAT[t], C14AcylCoAMAT[t], C12AcylCoAMAT[t],
    C10AcylCoAMAT[t], C8AcylCoAMAT[t], C6AcylCoAMAT[t], C4AcylCoAMAT[t], CoAMAT,
    C16FFA[t], C14FFA[t], C12FFA[t], C10FFA[t], C8FFA[t], C6FFA[t], C4FFA[t]],
  vacotC14 → ACOT[sfacotC14, Vmacot, KmacotC14CoA, KmacotC16CoA, KmacotC12CoA,
    KmacotC10CoA, KmacotC8CoA, KmacotC6CoA, KmacotC4CoA, KmCoA, KmacotC14FFA,
    KmacotC16FFA, KmacotC12FFA, KmacotC10FFA, KmacotC8FFA, KmacotC6FFA,
    KmacotC4FFA, KeqacotC14, C14AcylCoAMAT[t], C16AcylCoAMAT[t], C12AcylCoAMAT[t],
    C10AcylCoAMAT[t], C8AcylCoAMAT[t], C6AcylCoAMAT[t], C4AcylCoAMAT[t], CoAMAT,
    C14FFA[t], C16FFA[t], C12FFA[t], C10FFA[t], C8FFA[t], C6FFA[t], C4FFA[t]],
  vacotC12 → ACOT[sfacotC12, Vmacot, KmacotC12CoA, KmacotC16CoA, KmacotC14CoA,
    KmacotC10CoA, KmacotC8CoA, KmacotC6CoA, KmacotC4CoA, KmCoA, KmacotC12FFA,
    KmacotC16FFA, KmacotC14FFA, KmacotC10FFA, KmacotC8FFA, KmacotC6FFA,
    KmacotC4FFA, KeqacotC12, C12AcylCoAMAT[t], C16AcylCoAMAT[t], C14AcylCoAMAT[t],
    C10AcylCoAMAT[t], C8AcylCoAMAT[t], C6AcylCoAMAT[t], C4AcylCoAMAT[t], CoAMAT,
    C12FFA[t], C16FFA[t], C14FFA[t], C10FFA[t], C8FFA[t], C6FFA[t], C4FFA[t]],
  vacotC10 → ACOT[sfacotC10, Vmacot, KmacotC10CoA, KmacotC16CoA, KmacotC14CoA,
    KmacotC12CoA, KmacotC8CoA, KmacotC6CoA, KmacotC4CoA, KmCoA, KmacotC10FFA,
    KmacotC16FFA, KmacotC14FFA, KmacotC12FFA, KmacotC8FFA, KmacotC6FFA,
    KmacotC4FFA, KeqacotC10, C10AcylCoAMAT[t], C16AcylCoAMAT[t], C14AcylCoAMAT[t],
    C12AcylCoAMAT[t], C8AcylCoAMAT[t], C6AcylCoAMAT[t], C4AcylCoAMAT[t], CoAMAT,
    C10FFA[t], C16FFA[t], C14FFA[t], C12FFA[t], C8FFA[t], C6FFA[t], C4FFA[t]],
  vacotC8 → ACOT[sfacotC8, Vmacot, KmacotC8CoA, KmacotC16CoA, KmacotC14CoA,
    KmacotC12CoA, KmacotC10CoA, KmacotC6CoA, KmacotC4CoA, KmCoA, KmacotC8FFA,
```

```
      KmacotC16FFA, KmacotC14FFA, KmacotC12FFA, KmacotC10FFA, KmacotC6FFA,
      KmacotC4FFA, KeqacotC8, C8AcylCoAMAT[t], C16AcylCoAMAT[t], C14AcylCoAMAT[t],
      C12AcylCoAMAT[t], C10AcylCoAMAT[t], C6AcylCoAMAT[t], C4AcylCoAMAT[t], CoAMAT,
      C8FFA[t], C16FFA[t], C14FFA[t], C12FFA[t], C10FFA[t], C6FFA[t], C4FFA[t]],
    vacotC6 → ACOT[sfacotC6, Vmacot, KmacotC6CoA, KmacotC16CoA, KmacotC14CoA,
      KmacotC12CoA, KmacotC10CoA, KmacotC8CoA, KmacotC4CoA, KmCoA, KmacotC6FFA,
      KmacotC16FFA, KmacotC14FFA, KmacotC12FFA, KmacotC10FFA, KmacotC8FFA,
      KmacotC4FFA, KeqacotC6, C6AcylCoAMAT[t], C16AcylCoAMAT[t], C14AcylCoAMAT[t],
      C12AcylCoAMAT[t], C10AcylCoAMAT[t], C8AcylCoAMAT[t], C4AcylCoAMAT[t], CoAMAT,
      C6FFA[t], C16FFA[t], C14FFA[t], C12FFA[t], C10FFA[t], C8FFA[t], C4FFA[t]],
    vacotC4 → ACOT[sfacotC4, Vmacot, KmacotC4CoA, KmacotC16CoA, KmacotC14CoA,
      KmacotC12CoA, KmacotC10CoA, KmacotC8CoA, KmacotC6CoA, KmCoA, KmacotC4FFA,
      KmacotC16FFA, KmacotC14FFA, KmacotC12FFA, KmacotC10FFA, KmacotC8FFA,
      KmacotC6FFA, KeqacotC4, C4AcylCoAMAT[t], C16AcylCoAMAT[t], C14AcylCoAMAT[t],
      C12AcylCoAMAT[t], C10AcylCoAMAT[t], C8AcylCoAMAT[t], C6AcylCoAMAT[t], CoAMAT,
      C4FFA[t], C16FFA[t], C14FFA[t], C12FFA[t], C10FFA[t], C8FFA[t], C6FFA[t]],
    vffaC16 → ExportRate[keFFA, C16FFA[t]]
    vffaC14 → ExportRate[keFFA, C14FFA[t]]
    vffaC12 → ExportRate[keFFA, C12FFA[t]]
    vffaC10 → ExportRate[keFFA, C10FFA[t]]
    vffaC8 → ExportRate[keFFA, C8FFA[t]]
    vffaC6 → ExportRate[keFFA, C6FFA[t]],
    vffaC4 → ExportRate[keFFA, C4FFA[t]]
    vacesink → RES[Ksacesink, AcetylCoAMAT[t], K1acesink],
    vfadhsink → RES[Ksfadhsink, FADHMAT[t], K1fadhsink],
    vnadhsink → RES[Ksnadhsink, NADHMAT[t], K1nadhsink]};

CoAMATX =
  {CoAMAT → CoAMATt - C16AcylCoAMAT[t] - C16EnoylCoAMAT[t] - C16HydroxyacylCoAMAT[t] -
      C16KetoacylCoAMAT[t] - C14AcylCoAMAT[t] - C14EnoylCoAMAT[t] -
      C14HydroxyacylCoAMAT[t] - C14KetoacylCoAMAT[t] - C12AcylCoAMAT[t] -
      C12EnoylCoAMAT[t] - C12HydroxyacylCoAMAT[t] - C12KetoacylCoAMAT[t] -
      C10AcylCoAMAT[t] - C10EnoylCoAMAT[t] - C10HydroxyacylCoAMAT[t] -
      C10KetoacylCoAMAT[t] - C8AcylCoAMAT[t] - C8EnoylCoAMAT[t] -
      C8HydroxyacylCoAMAT[t] - C8KetoacylCoAMAT[t] - C6AcylCoAMAT[t] - C6EnoylCoAMAT[t] -
      C6HydroxyacylCoAMAT[t] - C6KetoacylCoAMAT[t] - C4AcylCoAMAT[t] - C4EnoylCoAMAT[t] -
      C4HydroxyacylCoAMAT[t] - C4AcetoacylCoAMAT[t] - AcetylCoAMAT[t]};


Parm = {
    sfcpt1C16 → 1, Vcpt1 → 0.012, Kmcpt1C16AcylCoACYT → 13.8,
    Kmcpt1CarCYT → 250, Kmcpt1C16AcylCarCYT → 136, Kmcpt1CoACYT → 40.7,
    Kicpt1MalCoACYT → 9.1, Keqcpt1 → 0.45, ncpt1 → 2.4799,
    Vfcact → 0.42, Vrcact → 0.42, KmcactC16AcylCarCYT → 15,
    KmcactC14AcylCarCYT → 15, KmcactC12AcylCarCYT → 15, KmcactC10AcylCarCYT → 15,
    KmcactC8AcylCarCYT → 15, KmcactC6AcylCarCYT → 15, KmcactC4AcylCarCYT → 15,
    KmcactCarMAT → 130, KmcactC16AcylCarMAT → 15, KmcactC14AcylCarMAT → 15,
    KmcactC12AcylCarMAT → 15, KmcactC10AcylCarMAT → 15, KmcactC8AcylCarMAT → 15,
    KmcactC6AcylCarMAT → 15, KmcactC4AcylCarMAT → 15, KmcactCarCYT → 130,
    KicactC16AcylCarCYT → 56, KicactC14AcylCarCYT → 56, KicactC12AcylCarCYT → 56,
    KicactC10AcylCarCYT → 56, KicactC8AcylCarCYT → 56, KicactC6AcylCarCYT → 56,
    KicactC4AcylCarCYT → 56, KicactCarCYT → 200, Keqcact → 1,
    sfcpt2C16 → 0.85, sfcpt2C14 → 1, sfcpt2C12 → 0.95, sfcpt2C10 → 0.95,
    sfcpt2C8 → 0.35, sfcpt2C6 → 0.15, sfcpt2C4 → 0.01, Vcpt2 → 0.391,
    Kmcpt2C16AcylCarMAT → 51, Kmcpt2C14AcylCarMAT → 51, Kmcpt2C12AcylCarMAT → 51,
```

```
Kmcpt2C10AcylCarMAT → 51, Kmcpt2C8AcylCarMAT → 51, Kmcpt2C6AcylCarMAT → 51,
Kmcpt2C4AcylCarMAT → 51, Kmcpt2CoAMAT → 30, Kmcpt2C16AcylCoAMAT → 38,
Kmcpt2C14AcylCoAMAT → 38, Kmcpt2C12AcylCoAMAT → 38,
Kmcpt2C10AcylCoAMAT → 38, Kmcpt2C8AcylCoAMAT → 38, Kmcpt2C6AcylCoAMAT → 1000,
Kmcpt2C4AcylCoAMAT → 1000000, Kmcpt2CarMAT → 350, Keqcpt2 → 2.22,
sfvlcadC16 → 1, sfvlcadC14 → 0.42, sfvlcadC12 → 0.11, Vvlcad → 0.008,
KmvlcadC16AcylCoAMAT → 6.5, KmvlcadC14AcylCoAMAT → 4, KmvlcadC12AcylCoAMAT → 2.7,
KmvlcadFAD → 0.12, KmvlcadC16EnoylCoAMAT → 1.08, KmvlcadC14EnoylCoAMAT → 1.08,
KmvlcadC12EnoylCoAMAT → 1.08, KmvlcadFADH → 24.2, Keqvlcad → 6,
sflcadC16 → 0.9, sflcadC14 → 1, sflcadC12 → 0.9, sflcadC10 → 0.75, sflcadC8 → 0.4,
Vlcad → 0.01, KmlcadC16AcylCoAMAT → 2.5, KmlcadC14AcylCoAMAT → 7.4,
KmlcadC12AcylCoAMAT → 9, KmlcadC10AcylCoAMAT → 24.3, KmlcadC8AcylCoAMAT → 123,
KmlcadFAD → 0.12, KmlcadC16EnoylCoAMAT → 1.08, KmlcadC14EnoylCoAMAT → 1.08,
KmlcadC12EnoylCoAMAT → 1.08, KmlcadC10EnoylCoAMAT → 1.08,
KmlcadC8EnoylCoAMAT → 1.08, KmlcadFADH → 24.2, Keqlcad → 6,
sfmcadC12 → 0.38, sfmcadC10 → 0.8, sfmcadC8 → 0.87, sfmcadC6 → 1, sfmcadC4 → 0.12,
Vmcad → 0.081, KmmcadC12AcylCoAMAT → 5.7, KmmcadC10AcylCoAMAT → 5.4,
KmmcadC8AcylCoAMAT → 4, KmmcadC6AcylCoAMAT → 9.4, KmmcadC4AcylCoAMAT → 135,
KmmcadFAD → 0.12, KmmcadC12EnoylCoAMAT → 1.08, KmmcadC10EnoylCoAMAT → 1.08,
KmmcadC8EnoylCoAMAT → 1.08, KmmcadC6EnoylCoAMAT → 1.08,
KmmcadC4EnoylCoAMAT → 1.08, KmmcadFADH → 24.2, Keqmcad → 6,
sfscadC6 → 0.3, sfscadC4 → 1, Vscad → 0.081, KmscadC6AcylCoAMAT → 285,
KmscadC4AcylCoAMAT → 10.7, KmscadFAD → 0.12, KmscadC6EnoylCoAMAT → 1.08,
KmscadC4EnoylCoAMAT → 1.08, KmscadFADH → 24.2, Keqscad -> 6,
sfcrotC16 → 0.13, sfcrotC14 → 0.2, sfcrotC12 → 0.25, sfcrotC10 → 0.33, sfcrotC8 → 0.58,
sfcrotC6 → 0.83, sfcrotC4 → 1, Vcrot → 3.6, KmcrotC16EnoylCoAMAT → 150,
KmcrotC14EnoylCoAMAT → 100, KmcrotC12EnoylCoAMAT → 25, KmcrotC10EnoylCoAMAT → 25,
KmcrotC8EnoylCoAMAT → 25, KmcrotC6EnoylCoAMAT → 25, KmcrotC4EnoylCoAMAT → 40,
KmcrotC16HydroxyacylCoAMAT → 45, KmcrotC14HydroxyacylCoAMAT → 45,
KmcrotC12HydroxyacylCoAMAT → 45, KmcrotC10HydroxyacylCoAMAT → 45,
KmcrotC8HydroxyacylCoAMAT → 45, KmcrotC6HydroxyacylCoAMAT → 45,
KmcrotC4HydroxyacylCoAMAT → 45, KicrotC4AcetoacylCoA → 1.6, Keqcrot → 3.13,
sfmschadC16 → 0.6, sfmschadC14 → 0.5, sfmschadC12 → 0.43, sfmschadC10 → 0.64,
sfmschadC8 → 0.89, sfmschadC6 → 1, sfmschadC4 → 0.67, Vmschad → 1,
KmmschadC16HydroxyacylCoAMAT → 1.5, KmmschadC14HydroxyacylCoAMAT → 1.8,
KmmschadC12HydroxyacylCoAMAT → 3.7, KmmschadC10HydroxyacylCoAMAT → 8.8,
KmmschadC8HydroxyacylCoAMAT → 16.3, KmmschadC6HydroxyacylCoAMAT → 28.6,
KmmschadC4HydroxyacylCoAMAT → 69.9, KmmschadNADMAT → 58.5,
KmmschadC16KetoacylCoAMAT → 1.4, KmmschadC14KetoacylCoAMAT → 1.4,
KmmschadC12KetoacylCoAMAT → 1.6, KmmschadC10KetoacylCoAMAT → 2.3,
KmmschadC8KetoacylCoAMAT → 4.1, KmmschadC6KetoacylCoAMAT → 5.8,
KmmschadC4AcetoacylCoAMAT → 16.9, KmmschadNADHMAT → 5.4, Keqmschad → 2.17 * 10^-4,
sfmckatC16 → 0, sfmckatC14 → 0.2, sfmckatC12 → 0.38, sfmckatC10 → 0.65,
sfmckatC8 → 0.81, sfmckatC6 → 1, sfmckatC4 → 0.49, Vmckat → 0.377,
KmmckatC16KetoacylCoAMAT → 1.1, KmmckatC14KetoacylCoAMAT → 1.2,
KmmckatC12KetoacylCoAMAT → 1.3, KmmckatC10KetoacylCoAMAT → 2.1,
KmmckatC8KetoacylCoAMAT → 3.2, KmmckatC6KetoacylCoAMAT → 6.7,
KmmckatC4AcetoacylCoAMAT → 12.4, KmmckatCoAMAT → 26.6,
KmmckatC14AcylCoAMAT → 13.83, KmmckatC16AcylCoAMAT → 13.83,
KmmckatC12AcylCoAMAT → 13.83, KmmckatC10AcylCoAMAT → 13.83,
KmmckatC8AcylCoAMAT → 13.83, KmmckatC6AcylCoAMAT → 13.83,
KmmckatC4AcylCoAMAT → 13.83, KmmckatAcetylCoAMAT → 30, Keqmckat → 1051,
sfmtpC16 → 1, sfmtpC14 → 0.9, sfmtpC12 → 0.81, sfmtpC10 → 0.73, sfmtpC8 → 0.34,
Vmtp → 2.84, KmmtpC16EnoylCoAMAT → 25, KmmtpC14EnoylCoAMAT → 25,
KmmtpC12EnoylCoAMAT → 25, KmmtpC10EnoylCoAMAT → 25, KmmtpC8EnoylCoAMAT → 25,
```

```
    KmmtpNADMAT → 60, KmmtpCoAMAT → 30, KmmtpC14AcylCoAMAT → 13.83,
    KmmtpC16AcylCoAMAT → 13.83, KmmtpC12AcylCoAMAT → 13.83,
    KmmtpC10AcylCoAMAT → 13.83, KmmtpC8AcylCoAMAT → 13.83, KmmtpC6AcylCoAMAT → 13.83,
    KmmtpNADHMAT → 50, KmmtpAcetylCoAMAT → 30, Keqmtp → 0.71,
    Vmacot → 0.00371, sfacotC16 → 0.70, sfacotC14 → 0.86, sfacotC12 → 0.68,
    sfacotC10 → 1.0, sfacotC8 → 0.21, sfacotC6 → 0.55, sfacotC4 → 0.70,
    KmacotC16CoA → 10, KmacotC14CoA → 15.2, KmacotC12CoA → 27.5, KmacotC10CoA → 47.2,
    KmacotC8CoA → 150.89, KmacotC6CoA → 138.2, KmacotC4CoA → 1036.29, KmCoA → 9,
    KmacotC16FFA → 27 000, KmacotC14FFA → 130 000, KmacotC12FFA → 230 000,
    KmacotC10FFA → 400 000, KmacotC8FFA → 1 300 000, KmacotC6FFA → 350 000 000,
    KmacotC4FFA → 3 000 000, KeqacotC16 → 24 000, KeqacotC14 → 76 000, KeqacotC12 → 76 000,
    KeqacotC10 → 76 000, KeqacotC8 → 76 000, KeqacotC6 → 76 000, KeqacotC4 → 3 000 000,
    keFFA → 2 * 10^-6, keC14 → 4 * 10^-6,
    Ksacesink → 6 000 000, K1acesink → 70, Ksfadhsink → 6 000 000,
    K1fadhsink → 0.46, Ksnadhsink → 6 000 000, K1nadhsink → 12,
    C16AcylCoACYT → 25, CarCYT → 200, CoACYT → 140, MalCoACYT → 0,
    CarMAT -> 950, FADtMAT → 0.77, NADtMAT → 250, CoAMATt → 5000,
    VCYT → 2.2 * 10^-6, VMAT → 1.8 * 10^-6};

InitialConditions = {
    C16AcylCarCYT[0] == 0, C16AcylCarMAT[0] == 0, C16AcylCoAMAT[0] == 0,
    C16EnoylCoAMAT[0] == 0, C16HydroxyacylCoAMAT[0] == 0, C16KetoacylCoAMAT[0] == 0,
    C14AcylCarCYT[0] == 0, C14AcylCarMAT[0] == 0, C14AcylCoAMAT[0] == 0,
    C14EnoylCoAMAT[0] == 0, C14HydroxyacylCoAMAT[0] == 0, C14KetoacylCoAMAT[0] == 0,
    C12AcylCarCYT[0] == 0, C12AcylCarMAT[0] == 0, C12AcylCoAMAT[0] == 0,
    C12EnoylCoAMAT[0] == 0, C12HydroxyacylCoAMAT[0] == 0, C12KetoacylCoAMAT[0] == 0,
    C10AcylCarCYT[0] == 0, C10AcylCarMAT[0] == 0, C10AcylCoAMAT[0] == 0,
    C10EnoylCoAMAT[0] == 0, C10HydroxyacylCoAMAT[0] == 0, C10KetoacylCoAMAT[0] == 0,
    C8AcylCarCYT[0] == 0, C8AcylCarMAT[0] == 0, C8AcylCoAMAT[0] == 0,
    C8EnoylCoAMAT[0] == 0, C8HydroxyacylCoAMAT[0] == 0, C8KetoacylCoAMAT[0] == 0,
    C6AcylCarCYT[0] == 0, C6AcylCarMAT[0] == 0, C6AcylCoAMAT[0] == 0,
    C6EnoylCoAMAT[0] == 0, C6HydroxyacylCoAMAT[0] == 0, C6KetoacylCoAMAT[0] == 0,
    C4AcylCarCYT[0] == 0, C4AcylCarMAT[0] == 0, C4AcylCoAMAT[0] == 0,
    C4EnoylCoAMAT[0] == 0, C4HydroxyacylCoAMAT[0] == 0, C4AcetoacylCoAMAT[0] == 0,
    AcetylCoAMAT[0] == 70, FADHMAT[0] == 0.46, NADHMAT[0] == 12,
    C16FFA[0] == 0, C14FFA[0] == 0, C12FFA[0] == 0,
    C10FFA[0] == 0, C8FFA[0] == 0, C6FFA[0] == 0, C4FFA[0] == 0};

Vars = {
    C16AcylCarCYT, C16AcylCarMAT, C16AcylCoAMAT,
    C16EnoylCoAMAT, C16HydroxyacylCoAMAT, C16KetoacylCoAMAT,
    C14AcylCarCYT, C14AcylCarMAT, C14AcylCoAMAT, C14EnoylCoAMAT,
    C14HydroxyacylCoAMAT, C14KetoacylCoAMAT,
    C12AcylCarCYT, C12AcylCarMAT, C12AcylCoAMAT, C12EnoylCoAMAT,
    C12HydroxyacylCoAMAT, C12KetoacylCoAMAT,
    C10AcylCarCYT, C10AcylCarMAT, C10AcylCoAMAT, C10EnoylCoAMAT,
    C10HydroxyacylCoAMAT, C10KetoacylCoAMAT,
    C8AcylCarCYT, C8AcylCarMAT, C8AcylCoAMAT, C8EnoylCoAMAT,
    C8HydroxyacylCoAMAT, C8KetoacylCoAMAT,
    C6AcylCarCYT, C6AcylCarMAT, C6AcylCoAMAT, C6EnoylCoAMAT,
    C6HydroxyacylCoAMAT, C6KetoacylCoAMAT,
    C4AcylCarCYT, C4AcylCarMAT, C4AcylCoAMAT, C4EnoylCoAMAT,
    C4HydroxyacylCoAMAT, C4AcetoacylCoAMAT,
    AcetylCoAMAT, FADHMAT, NADHMAT,
     C16FFA, C14FFA, C12FFA, C10FFA, C8FFA, C6FFA, C4FFA};
```

```
In[•]:= TableForm[Odes];
TableForm[RateEqs];
TableForm[Odes /. RateEqs /. CoAMATX /. Parm ];
TableForm[RateEqs /. Parm ];
TableForm[InitialConditions];

In[•]:=
tsol = NDSolve[Join[Odes /. RateEqs /. CoAMATX /. Parm , InitialConditions],
   Vars, {t, 0, 1000000000}];

In[•]:= Table[{Vars[[i]][t], (Vars[[i]][900000000] /. tsol)[[1]]}, {i, 1, Length[Vars]}]

Out[•]= {{C16AcylCarCYT[t], 0.167871}, {C16AcylCarMAT[t], 0.355364},
 {C16AcylCoAMAT[t], 0.870444}, {C16EnoylCoAMAT[t], 0.0485765},
 {C16HydroxyacylCoAMAT[t], 0.152044}, {C16KetoacylCoAMAT[t], 0.000654373},
 {C14AcylCarCYT[t], 0.0372737}, {C14AcylCarMAT[t], 0.17705},
 {C14AcylCoAMAT[t], 1.92851}, {C14EnoylCoAMAT[t], 0.0542268},
 {C14HydroxyacylCoAMAT[t], 0.154025}, {C14KetoacylCoAMAT[t], 0.000662217},
 {C12AcylCarCYT[t], 0.0508391}, {C12AcylCarMAT[t], 0.241486},
 {C12AcylCoAMAT[t], 2.63037}, {C12EnoylCoAMAT[t], 0.0618663},
 {C12HydroxyacylCoAMAT[t], 0.187162}, {C12KetoacylCoAMAT[t], 0.000802167},
 {C10AcylCarCYT[t], 0.0912553}, {C10AcylCarMAT[t], 0.433463},
 {C10AcylCoAMAT[t], 4.72147}, {C10EnoylCoAMAT[t], 0.0680884},
 {C10HydroxyacylCoAMAT[t], 0.207337}, {C10KetoacylCoAMAT[t], 0.00088605},
 {C8AcylCarCYT[t], 0.0936016}, {C8AcylCarMAT[t], 0.444608}, {C8AcylCoAMAT[t], 4.84287},
 {C8EnoylCoAMAT[t], 0.147599}, {C8HydroxyacylCoAMAT[t], 0.456175},
 {C8KetoacylCoAMAT[t], 0.00194848}, {C6AcylCarCYT[t], 0.248461},
 {C6AcylCarMAT[t], 1.18019}, {C6AcylCoAMAT[t], 12.8552}, {C6EnoylCoAMAT[t], 11.0793},
 {C6HydroxyacylCoAMAT[t], 34.4789}, {C6KetoacylCoAMAT[t], 0.147256},
 {C4AcylCarCYT[t], 0.412546}, {C4AcylCarMAT[t], 1.95959}, {C4AcylCoAMAT[t], 21.3448},
 {C4EnoylCoAMAT[t], 41.6664}, {C4HydroxyacylCoAMAT[t], 130.151},
 {C4AcetoacylCoAMAT[t], 0.556006}, {AcetylCoAMAT[t], 70.},
 {FADHMAT[t], 0.46}, {NADHMAT[t], 12.}, {C16FFA[t], 0.135803},
 {C14FFA[t], 0.248805}, {C12FFA[t], 0.148975}, {C10FFA[t], 0.229231},
 {C8FFA[t], 0.0154885}, {C6FFA[t], 0.117523}, {C4FFA[t], 0.033139}}
```

Steady state computation by varying palmitoyl-CoA with ACOT extension

```
In[•]:= ParmScan[X_] := {
   sfcpt1C16 → 1, Vcpt1 → 0.012, Kmcpt1C16AcylCoACYT → 13.8,
   Kmcpt1CarCYT → 250, Kmcpt1C16AcylCarCYT → 136, Kmcpt1CoACYT → 40.7,
   Kicpt1MalCoACYT → 9.1, Keqcpt1 → 0.45,  ncpt1 → 2.4799,
   Vfcact → 0.42, Vrcact → 0.42, KmcactC16AcylCarCYT → 15,
   KmcactC14AcylCarCYT → 15, KmcactC12AcylCarCYT → 15, KmcactC10AcylCarCYT → 15,
   KmcactC8AcylCarCYT → 15, KmcactC6AcylCarCYT → 15, KmcactC4AcylCarCYT → 15,
   KmcactCarMAT → 130, KmcactC16AcylCarMAT → 15, KmcactC14AcylCarMAT → 15,
   KmcactC12AcylCarMAT → 15, KmcactC10AcylCarMAT → 15, KmcactC8AcylCarMAT → 15,
   KmcactC6AcylCarMAT → 15, KmcactC4AcylCarMAT → 15, KmcactCarCYT → 130,
   KicactC16AcylCarCYT → 56, KicactC14AcylCarCYT → 56, KicactC12AcylCarCYT → 56,
   KicactC10AcylCarCYT → 56, KicactC8AcylCarCYT → 56, KicactC6AcylCarCYT → 56,
   KicactC4AcylCarCYT → 56, KicactCarCYT → 200, Keqcact → 1,
   sfcpt2C16 → 0.85, sfcpt2C14 → 1, sfcpt2C12 → 0.95, sfcpt2C10 → 0.95,
   sfcpt2C8 → 0.35, sfcpt2C6 → 0.15, sfcpt2C4 → 0.01, Vcpt2 → 0.391,
   Kmcpt2C16AcylCarMAT → 51, Kmcpt2C14AcylCarMAT → 51, Kmcpt2C12AcylCarMAT → 51,
   Kmcpt2C10AcylCarMAT → 51, Kmcpt2C8AcylCarMAT → 51, Kmcpt2C6AcylCarMAT → 51,
   Kmcpt2C4AcylCarMAT → 51, Kmcpt2CoAMAT → 30, Kmcpt2C16AcylCoAMAT → 38,
```

```
Kmcpt2C14AcylCoAMAT → 38, Kmcpt2C12AcylCoAMAT → 38,
Kmcpt2C10AcylCoAMAT → 38, Kmcpt2C8AcylCoAMAT → 38, Kmcpt2C6AcylCoAMAT → 1000,
Kmcpt2C4AcylCoAMAT → 1000000, Kmcpt2CarMAT → 350, Keqcpt2 → 2.22,
sfvlcadC16 → 1, sfvlcadC14 → 0.42, sfvlcadC12 → 0.11, Vvlcad → 0.008,
KmvlcadC16AcylCoAMAT → 6.5, KmvlcadC14AcylCoAMAT → 4, KmvlcadC12AcylCoAMAT → 2.7,
KmvlcadFAD → 0.12, KmvlcadC16EnoylCoAMAT → 1.08, KmvlcadC14EnoylCoAMAT → 1.08,
KmvlcadC12EnoylCoAMAT → 1.08, KmvlcadFADH → 24.2, Keqvlcad → 6,
sflcadC16 → 0.9, sflcadC14 → 1, sflcadC12 → 0.9, sflcadC10 → 0.75, sflcadC8 → 0.4,
Vlcad → 0.01, KmlcadC16AcylCoAMAT → 2.5, KmlcadC14AcylCoAMAT → 7.4,
KmlcadC12AcylCoAMAT → 9, KmlcadC10AcylCoAMAT → 24.3, KmlcadC8AcylCoAMAT → 123,
KmlcadFAD → 0.12, KmlcadC16EnoylCoAMAT → 1.08, KmlcadC14EnoylCoAMAT → 1.08,
KmlcadC12EnoylCoAMAT → 1.08, KmlcadC10EnoylCoAMAT → 1.08,
KmlcadC8EnoylCoAMAT → 1.08, KmlcadFADH → 24.2, Keqlcad → 6,
sfmcadC12 → 0.38, sfmcadC10 → 0.8, sfmcadC8 → 0.87, sfmcadC6 → 1, sfmcadC4 → 0.12,
Vmcad → 0.081, KmmcadC12AcylCoAMAT → 5.7, KmmcadC10AcylCoAMAT → 5.4,
KmmcadC8AcylCoAMAT → 4, KmmcadC6AcylCoAMAT → 9.4, KmmcadC4AcylCoAMAT → 135,
KmmcadFAD → 0.12, KmmcadC12EnoylCoAMAT → 1.08, KmmcadC10EnoylCoAMAT → 1.08,
KmmcadC8EnoylCoAMAT → 1.08, KmmcadC6EnoylCoAMAT → 1.08,
KmmcadC4EnoylCoAMAT → 1.08, KmmcadFADH → 24.2, Keqmcad → 6,
sfscadC6 → 0.3, sfscadC4 → 1, Vscad → 0.081, KmscadC6AcylCoAMAT → 285,
KmscadC4AcylCoAMAT → 10.7, KmscadFAD → 0.12, KmscadC6EnoylCoAMAT → 1.08,
KmscadC4EnoylCoAMAT → 1.08, KmscadFADH → 24.2, Keqscad -> 6,
sfcrotC16 → 0.13, sfcrotC14 → 0.2, sfcrotC12 → 0.25, sfcrotC10 → 0.33, sfcrotC8 → 0.58,
sfcrotC6 → 0.83, sfcrotC4 → 1, Vcrot → 3.6, KmcrotC16EnoylCoAMAT → 150,
KmcrotC14EnoylCoAMAT → 100, KmcrotC12EnoylCoAMAT → 25, KmcrotC10EnoylCoAMAT → 25,
KmcrotC8EnoylCoAMAT → 25, KmcrotC6EnoylCoAMAT → 25, KmcrotC4EnoylCoAMAT → 40,
KmcrotC16HydroxyacylCoAMAT → 45, KmcrotC14HydroxyacylCoAMAT → 45,
KmcrotC12HydroxyacylCoAMAT → 45, KmcrotC10HydroxyacylCoAMAT → 45,
KmcrotC8HydroxyacylCoAMAT → 45, KmcrotC6HydroxyacylCoAMAT → 45,
KmcrotC4HydroxyacylCoAMAT → 45, KicrotC4AcetoacylCoA → 1.6, Keqcrot → 3.13,
sfmschadC16 → 0.6, sfmschadC14 → 0.5, sfmschadC12 → 0.43, sfmschadC10 → 0.64,
sfmschadC8 → 0.89, sfmschadC6 → 1, sfmschadC4 → 0.67, Vmschad → 1,
KmmschadC16HydroxyacylCoAMAT → 1.5, KmmschadC14HydroxyacylCoAMAT → 1.8,
KmmschadC12HydroxyacylCoAMAT → 3.7, KmmschadC10HydroxyacylCoAMAT → 8.8,
KmmschadC8HydroxyacylCoAMAT → 16.3, KmmschadC6HydroxyacylCoAMAT → 28.6,
KmmschadC4HydroxyacylCoAMAT → 69.9, KmmschadNADMAT → 58.5,
KmmschadC16KetoacylCoAMAT → 1.4, KmmschadC14KetoacylCoAMAT → 1.4,
KmmschadC12KetoacylCoAMAT → 1.6, KmmschadC10KetoacylCoAMAT → 2.3,
KmmschadC8KetoacylCoAMAT → 4.1, KmmschadC6KetoacylCoAMAT → 5.8,
KmmschadC4AcetoacylCoAMAT → 16.9, KmmschadNADHMAT → 5.4, Keqmschad → 2.17 * 10^-4,
sfmckatC16 → 0, sfmckatC14 → 0.2, sfmckatC12 → 0.38, sfmckatC10 → 0.65,
sfmckatC8 → 0.81, sfmckatC6 → 1, sfmckatC4 → 0.49, Vmckat → 0.377,
KmmckatC16KetoacylCoAMAT → 1.1, KmmckatC14KetoacylCoAMAT → 1.2,
KmmckatC12KetoacylCoAMAT → 1.3, KmmckatC10KetoacylCoAMAT → 2.1,
KmmckatC8KetoacylCoAMAT → 3.2, KmmckatC6KetoacylCoAMAT → 6.7,
KmmckatC4AcetoacylCoAMAT → 12.4, KmmckatCoAMAT → 26.6,
KmmckatC14AcylCoAMAT → 13.83, KmmckatC16AcylCoAMAT → 13.83,
KmmckatC12AcylCoAMAT → 13.83, KmmckatC10AcylCoAMAT → 13.83,
KmmckatC8AcylCoAMAT → 13.83, KmmckatC6AcylCoAMAT → 13.83,
KmmckatC4AcylCoAMAT → 13.83, KmmckatAcetylCoAMAT → 30, Keqmckat → 1051,
sfmtpC16 → 1, sfmtpC14 → 0.9, sfmtpC12 → 0.81, sfmtpC10 → 0.73, sfmtpC8 → 0.34,
Vmtp → 2.84, KmmtpC16EnoylCoAMAT → 25, KmmtpC14EnoylCoAMAT → 25,
KmmtpC12EnoylCoAMAT → 25, KmmtpC10EnoylCoAMAT → 25, KmmtpC8EnoylCoAMAT → 25,
KmmtpNADMAT → 60, KmmtpCoAMAT → 30, KmmtpC14AcylCoAMAT → 13.83,
KmmtpC16AcylCoAMAT → 13.83, KmmtpC12AcylCoAMAT → 13.83,
```

```
    KmmtpC10AcylCoAMAT → 13.83, KmmtpC8AcylCoAMAT → 13.83, KmmtpC6AcylCoAMAT → 13.83,
    KmmtpNADHMAT → 50, KmmtpAcetylCoAMAT → 30, Keqmtp → 0.71,
    Vmacot → 0.00371, sfacotC16 → 0.70, sfacotC14 → 0.86, sfacotC12 → 0.68,
    sfacotC10 → 1.0, sfacotC8 → 0.21, sfacotC6 → 0.55, sfacotC4 → 0.70,
    KmacotC16CoA → 10, KmacotC14CoA → 15.2, KmacotC12CoA → 27.5, KmacotC10CoA → 47.2,
    KmacotC8CoA → 150.89, KmacotC6CoA → 138.2, KmacotC4CoA → 1036.29, KmCoA → 9,
    KmacotC16FFA → 27000, KmacotC14FFA → 130000, KmacotC12FFA → 230000,
    KmacotC10FFA → 400000, KmacotC8FFA → 1300000, KmacotC6FFA → 350000000,
    KmacotC4FFA → 3000000, KeqacotC16 → 24000, KeqacotC14 → 76000, KeqacotC12 → 76000,
    KeqacotC10 → 76000, KeqacotC8 → 76000, KeqacotC6 → 76000, KeqacotC4 → 3000000,
    keFFA → 2 * 10^-6, keC14 → 4 * 10^-6,
    Ksacesink → 6000000, K1acesink → 70, Ksfadhsink → 6000000,
    K1fadhsink → 0.46, Ksnadhsink → 6000000, K1nadhsink → 12,
    C16AcylCoACYT → X, CarCYT → 200, CoACYT → 140, MalCoACYT → 0,
    CarMAT -> 950, FADtMAT → 0.77, NADtMAT → 250, CoAMATt → 5000,
    VCYT → 2.2 * 10^-6, VMAT → 1.8 * 10^-6};

tsolScan[X_] :=
  NDSolve[Join[Odes /. RateEqs /. CoAMATX /. ParmScan[X], InitialConditions],
   Vars, {t, 0, 1000000000}];

SsScan[X_] := Module[{SSGuess},
  SSGuess := Table[{Vars[[i]][t],
     (Vars[[i]][900000000] /. tsolScan[X])[[1]]}, {i, 1, Length[Vars]}];
  FindRoot[Table[Odes[[i, 2]] == 0, {i, 1, Length[Odes]}] /. RateEqs /. CoAMATX /.
    ParmScan[X], SSGuess]]
```

In[∘]:=

```
In[∘]:= ScanDownNDS[Xstart_, dX_, Xend_] := Monitor[Module[{SS, SSGuess},
    DataDownNDSfluxacot = {};

    DataDownNDSconc = {};
    DataDownNDSc4coa = {};
    DataDownNDSc6coa = {};
    DataDownNDSc4c6coa = {};
    DataDownNDScintermedcoa = {};
    DataDownNDSfreecoa = {};

    DataDownNDSvacesink = {};
    DataDownNDSvfadhsink = {};
    DataDownNDSvnadhsinkACOT = {};
    DataDownNDSvacotC4 = {};
    DataDownNDSvacotC6 = {};
    DataDownNDSvacotC8 = {};
    DataDownNDSvacotC10 = {};
    DataDownNDSvacotC12 = {};
    DataDownNDSvacotC14 = {};
    DataDownNDSvacotC16 = {};
    tsolStart = tsolScan[Xend];
    SSGuess = Table[{Vars[[i]][t],
       (Vars[[i]][900000000] /. tsolStart)[[1]]}, {i, 1, Length[Vars]}];
    SSGuess1 = SSGuess[[All, 1]];
    SSGuess2 = SSGuess[[All, 2]];
    SSGuess1int = SSGuess1 /. t → 0;
```

```
InitialConditionsUD = Thread[SSGuess1int == SSGuess2];

For[X = Xend, X ≥ Xstart,
 tsolScanNDS = NDSolve[Join[Odes /. RateEqs /. CoAMATX /. ParmScan[X],
    InitialConditionsUD], Vars, {t, 0, 1000000000}];
 SSGuess = Table[{Vars[[i]][t], (Vars[[i]][900000000] /. tsolScanNDS)[[1]]},
   {i, 1, Length[Vars]}];
 SSGuess1 = SSGuess[[All, 1]];
 SSGuess2 = SSGuess[[All, 2]];
 SSGuess1int = SSGuess1 /. t → 0;
 InitialConditionsUD = Thread[SSGuess1int == SSGuess2];
 SS = Thread[SSGuess1 → SSGuess2];

 c4coa = C4AcylCoAMAT[t] + C4EnoylCoAMAT[t] +
    C4HydroxyacylCoAMAT[t] + C4AcetoacylCoAMAT[t] /. SS;
 c6coa = C6AcylCoAMAT[t] + C6EnoylCoAMAT[t] + C6HydroxyacylCoAMAT[t] +
    C6KetoacylCoAMAT[t] /. SS;
 c4c6coa = c4coa + c6coa;
 intermediatecoa =
  C4AcylCoAMAT[t] + C4EnoylCoAMAT[t] + C4HydroxyacylCoAMAT[t] + C4AcetoacylCoAMAT[t] +
    C6AcylCoAMAT[t] + C6EnoylCoAMAT[t] + C6HydroxyacylCoAMAT[t] +
    C6KetoacylCoAMAT[t] + C8AcylCoAMAT[t] + C8EnoylCoAMAT[t] +
    C8HydroxyacylCoAMAT[t] + C8KetoacylCoAMAT[t] + C10AcylCoAMAT[t] +
    C10EnoylCoAMAT[t] + C10HydroxyacylCoAMAT[t] + C10KetoacylCoAMAT[t] +
    C12AcylCoAMAT[t] + C12EnoylCoAMAT[t] + C12HydroxyacylCoAMAT[t] +
    C12KetoacylCoAMAT[t] + C14AcylCoAMAT[t] + C14EnoylCoAMAT[t] +
    C14HydroxyacylCoAMAT[t] + C14KetoacylCoAMAT[t] + C16AcylCoAMAT[t] +
    C16EnoylCoAMAT[t] + C16HydroxyacylCoAMAT[t] + C16KetoacylCoAMAT[t]  /. SS;
 freeCoa = CoAMATt - intermediatecoa - 70 /. CoAMATX /. ParmScan[X] /. SS;


 C16AcylCarnitineCYT = C16AcylCarCYT[t] /. SS;
 C14AcylCarnitineCYT = C14AcylCarCYT[t] /. SS;
 C12AcylCarnitineCYT = C12AcylCarCYT[t] /. SS;
 C10AcylCarnitineCYT = C10AcylCarCYT[t] /. SS;
 C8AcylCarnitineCYT = C8AcylCarCYT[t] /. SS;
 C6AcylCarnitineCYT = C6AcylCarCYT[t] /. SS;
 C4AcylCarnitineCYT = C4AcylCarCYT[t] /. SS;

 AppendTo[DataDownNDSfluxacot,
  {X, 10^3 vcactC16 /. RateEqs /. CoAMATX /. ParmScan[X] /. SS}];
 AppendTo[DataDownNDSc4coa, {X, c4coa}];
 AppendTo[DataDownNDSc6coa, {X, c6coa}];
 AppendTo[DataDownNDSc4c6coa, {X, c4c6coa}];
 AppendTo[DataDownNDScintermedcoa, {X, intermediatecoa}];
 AppendTo[DataDownNDSfreecoa, {X, freeCoa}];

 AppendTo[DataDownNDSvacesink,
  {X, 10^3 vacesink /. RateEqs /. CoAMATX /. ParmScan[X] /. SS}];
 AppendTo[DataDownNDSvfadhsink,
  {X, 10^3 vfadhsink /. RateEqs /. CoAMATX /. ParmScan[X] /. SS}];
 AppendTo[DataDownNDSvnadhsinkACOT,
  {X, 10^3 vnadhsink /. RateEqs /. CoAMATX /. ParmScan[X] /. SS}];
```

```
      AppendTo[DataDownNDSvacotC16,
        {X, 10^3 vacotC16 /. RateEqs /. CoAMATX /. ParmScan[X] /. SS}];
      AppendTo[DataDownNDSvacotC14,
        {X, 10^3 vacotC14 /. RateEqs /. CoAMATX /. ParmScan[X] /. SS}];
      AppendTo[DataDownNDSvacotC12,
        {X, 10^3 vacotC12 /. RateEqs /. CoAMATX /. ParmScan[X] /. SS}];
      AppendTo[DataDownNDSvacotC10,
        {X, 10^3 vacotC10 /. RateEqs /. CoAMATX /. ParmScan[X] /. SS}];
      AppendTo[DataDownNDSvacotC8,
        {X, 10^3 vacotC8 /. RateEqs /. CoAMATX /. ParmScan[X] /. SS}];
      AppendTo[DataDownNDSvacotC6,
        {X, 10^3 vacotC6 /. RateEqs /. CoAMATX /. ParmScan[X] /. SS}];
      AppendTo[DataDownNDSvacotC4,
        {X, 10^3 vacotC4 /. RateEqs /. CoAMATX /. ParmScan[X] /. SS}];
      X = X - dX;]
    ], ProgressIndicator[X, {Xstart, Xend}]]
```

```
In[•]:= ScanDownNDS[0, 1, 250]
```

```
In[•]:= ScanUpNDS[Xstart_, dX_, Xend_] := Monitor[Module[{SS, SSGuess},
    DataUpNDSfluxacot = {};

    DataUpNDSconc = {};
    DataUpNDSc4coa = {};
    DataUpNDSc6coa = {};
    DataUpNDSc4c6coa = {};
    DataUpNDScintermedcoa = {};
    DataUpNDSfreecoa = {};

    DataUpNDSvacesink = {};
    DataUpNDSvfadhsink = {};
    DataUpNDSvnadhsinkACOT = {};
    DataUpNDSvacotC4 = {};
    DataUpNDSvacotC6 = {};
    DataUpNDSvacotC8 = {};
    DataUpNDSvacotC10 = {};
    DataUpNDSvacotC12 = {};
    DataUpNDSvacotC14 = {};
    DataUpNDSvacotC16 = {};
    tsolStart = tsolScan[Xstart];
    SSGuess = Table[{Vars[[i]][t],
        (Vars[[i]][900 000 000] /. tsolStart)[[1]]}, {i, 1, Length[Vars]}];
    SSGuess1 = SSGuess[[All, 1]];
    SSGuess2 = SSGuess[[All, 2]];
    SSGuess1int = SSGuess1 /. t → 0;
    InitialConditionsUD = Thread[SSGuess1int == SSGuess2];

    For[X = Xstart, X ≤ Xend,
```

```
tsolScanNDS = NDSolve[Join[Odes /. RateEqs /. CoAMATX /. ParmScan[X],
    InitialConditionsUD], Vars, {t, 0, 1000000000}];
SSGuess = Table[{Vars[[i]][t], (Vars[[i]][900000000] /. tsolScanNDS)[[1]]},
   {i, 1, Length[Vars]}];
SSGuess1 = SSGuess[[All, 1]];
SSGuess2 = SSGuess[[All, 2]];
SSGuess1int = SSGuess1 /. t → 0;
InitialConditionsUD = Thread[SSGuess1int == SSGuess2];
SS = Thread[SSGuess1 → SSGuess2];

c4coa = C4AcylCoAMAT[t] + C4EnoylCoAMAT[t] +
    C4HydroxyacylCoAMAT[t] + C4AcetoacylCoAMAT[t] /. SS;
c6coa = C6AcylCoAMAT[t] + C6EnoylCoAMAT[t] + C6HydroxyacylCoAMAT[t] +
    C6KetoacylCoAMAT[t] /. SS;
c4c6coa = c4coa + c6coa;
intermediatecoa =
  C4AcylCoAMAT[t] + C4EnoylCoAMAT[t] + C4HydroxyacylCoAMAT[t] + C4AcetoacylCoAMAT[t] +
    C6AcylCoAMAT[t] + C6EnoylCoAMAT[t] + C6HydroxyacylCoAMAT[t] +
    C6KetoacylCoAMAT[t] + C8AcylCoAMAT[t] + C8EnoylCoAMAT[t] +
    C8HydroxyacylCoAMAT[t] + C8KetoacylCoAMAT[t] + C10AcylCoAMAT[t] +
    C10EnoylCoAMAT[t] + C10HydroxyacylCoAMAT[t] + C10KetoacylCoAMAT[t] +
    C12AcylCoAMAT[t] + C12EnoylCoAMAT[t] + C12HydroxyacylCoAMAT[t] +
    C12KetoacylCoAMAT[t] + C14AcylCoAMAT[t] + C14EnoylCoAMAT[t] +
    C14HydroxyacylCoAMAT[t] + C14KetoacylCoAMAT[t] + C16AcylCoAMAT[t] +
    C16EnoylCoAMAT[t] + C16HydroxyacylCoAMAT[t] + C16KetoacylCoAMAT[t]  /. SS;
freeCoa = CoAMATt - intermediatecoa - 70 /. CoAMATX /. ParmScan[X] /. SS;


C16AcylCarnitineCYT = C16AcylCarCYT[t] /. SS;
C14AcylCarnitineCYT = C14AcylCarCYT[t] /. SS;
C12AcylCarnitineCYT = C12AcylCarCYT[t] /. SS;
C10AcylCarnitineCYT = C10AcylCarCYT[t] /. SS;
C8AcylCarnitineCYT = C8AcylCarCYT[t] /. SS;
C6AcylCarnitineCYT = C6AcylCarCYT[t] /. SS;
C4AcylCarnitineCYT = C4AcylCarCYT[t] /. SS;

AppendTo[DataUpNDSfluxacot,
  {X, 10^3 vcactC16 /. RateEqs /. CoAMATX /. ParmScan[X] /. SS}];
AppendTo[DataUpNDSc4coa, {X, c4coa}];
AppendTo[DataUpNDSc6coa, {X, c6coa}];
AppendTo[DataUpNDSc4c6coa, {X, c4c6coa}];
AppendTo[DataUpNDScintermedcoa, {X, intermediatecoa}];
AppendTo[DataUpNDSfreecoa, {X, freeCoa}];

AppendTo[DataUpNDSvacesink,
  {X, 10^3 vacesink /. RateEqs /. CoAMATX /. ParmScan[X] /. SS}];
AppendTo[DataUpNDSvfadhsink,
  {X, 10^3 vfadhsink /. RateEqs /. CoAMATX /. ParmScan[X] /. SS}];
AppendTo[DataUpNDSvnadhsinkACOT,
  {X, 10^3 vnadhsink /. RateEqs /. CoAMATX /. ParmScan[X] /. SS}];
AppendTo[DataUpNDSvacotC16, {X,
   10^3 vacotC16 /. RateEqs /. CoAMATX /. ParmScan[X] /. SS}];
```

```
    AppendTo[DataUpNDSvacotC14, {X, 10^3 vacotC14 /. RateEqs /. CoAMATX /.
        ParmScan[X] /. SS}];
    AppendTo[DataUpNDSvacotC12, {X, 10^3 vacotC12 /. RateEqs /. CoAMATX /.
        ParmScan[X] /. SS}];
    AppendTo[DataUpNDSvacotC10, {X, 10^3 vacotC10 /. RateEqs /. CoAMATX /.
        ParmScan[X] /. SS}];
    AppendTo[DataUpNDSvacotC8, {X, 10^3 vacotC8 /. RateEqs /. CoAMATX /.
        ParmScan[X] /. SS}];
    AppendTo[DataUpNDSvacotC6, {X, 10^3 vacotC6 /. RateEqs /. CoAMATX /.
        ParmScan[X] /. SS}];
    AppendTo[DataUpNDSvacotC4, {X, 10^3 vacotC4 /. RateEqs /. CoAMATX /.
        ParmScan[X] /. SS}];
    X = X + dX;]
  ], ProgressIndicator[X, {Xstart, Xend}]]
```

In[∘]:=
```
ScanUpNDS[0, 1, 250]
```

In[∘]:=

In[∘]:=
```
PA1 = ListLinePlot[{DataUpNDSvacotC16, DataUpNDSvacotC14, DataUpNDSvacotC12,
    DataUpNDSvacotC10, DataUpNDSvacotC8, DataUpNDSvacotC6, DataUpNDSvacotC4},
  PlotRange → All, PlotStyle → {Red, Green, Cyan, Pink, Blue, Magenta, Orange},
  AxesStyle → Directive[Black, 14],
  LabelStyle → Directive[Thick, Black, Bold], PlotLegends →
   {"ACOTC16", "ACOTC14", "ACOTC12", "ACOTC10", "ACOTC8", "ACOTC6", "ACOTC4"}]
```

Out[∘]=

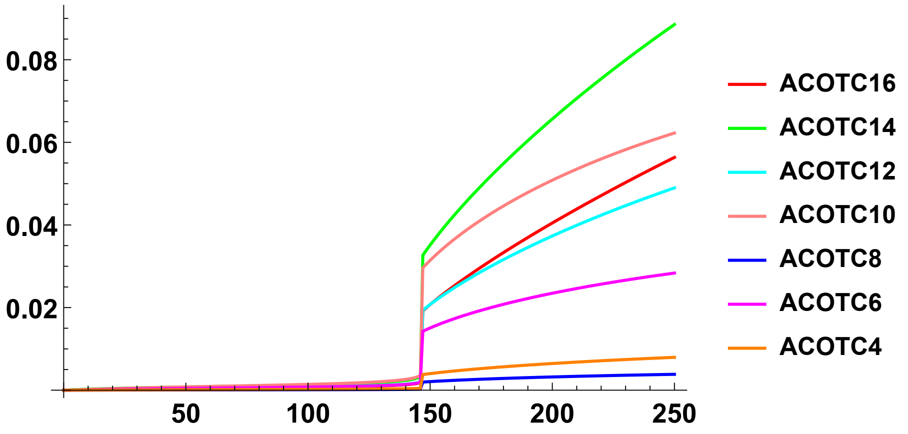

In[•]:=
```
PA2 = ListLinePlot[{DataDownNDSvacotC16, DataDownNDSvacotC14, DataDownNDSvacotC12,
    DataDownNDSvacotC10, DataDownNDSvacotC8, DataDownNDSvacotC6, DataDownNDSvacotC4},
  PlotRange → All, PlotStyle → {Red, Green, Cyan, Pink, Blue, Magenta, Orange},
  AxesStyle → Directive[Black, 14],
  LabelStyle → Directive[Thick, Black, Bold], PlotLegends →
   {"ACOTC16", "ACOTC14", "ACOTC12", "ACOTC10", "ACOTC8", "ACOTC6", "ACOTC4"}]
```

Out[•]=

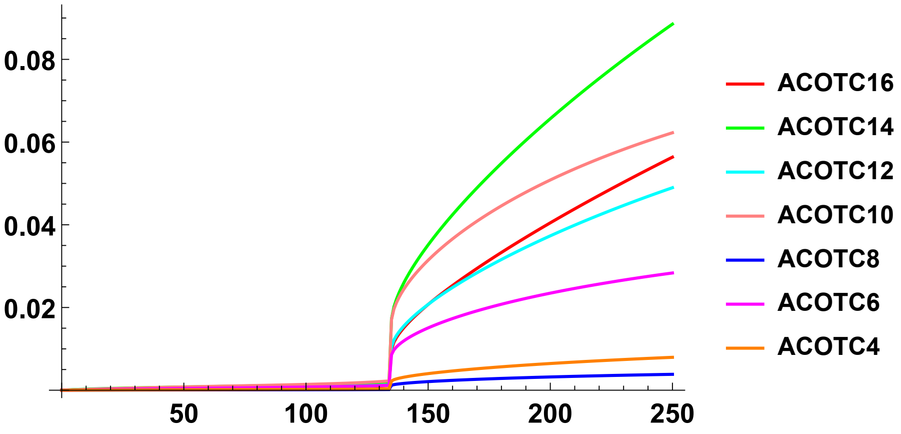

In[•]:=

Mitochondrial Fatty Acid Oxidation Kinetic model

Definitions of the various functions

In[•]:=
```
CPT1[sf_, V_, Kms1_, Kms2_, Kmp1_, Kmp2_, Ki1_, Keq_, S1_, S2_, P1_, P2_, I1_, n_] :=
 (sf * V * ((S1 * S2) / (Kms1 * Kms2) - (P1 * P2) / (Kms1 * Kms2 * Keq))) /
  ((1 + S1 / Kms1 + P1 / Kmp1 + (I1 / Ki1)^n) * (1 + S2 / Kms2 + P2 / Kmp2))
```

In[•]:=
```
CACT[Vf_, Vr_, Kms1_, Kms2_, Kmp1_, Kmp2_, Kis1_, Kip2_, Keq_, S1_, S2_, P1_, P2_] :=
 (Vf * (S1 * S2 - (P1 * P2) / Keq)) / (S1 * S2 + Kms2 * S1 + Kms1 * S2 * (1 + P2 / Kip2) +
    (Vf / (Vr * Keq)) * (Kmp2 * P1 * (1 + S1 / Kis1) + P2 * (Kmp1 + P1)))
```

In[•]:=
```
CPT2[sf_, V_, Kms1_, Kms2_, Kms3_, Kms4_, Kms5_, Kms6_, Kms7_, Kms8_,
  Kmp1_, Kmp2_, Kmp3_, Kmp4_, Kmp5_, Kmp6_, Kmp7_, Kmp8_, Keq_, S1_, S2_,
  S3_, S4_, S5_, S6_, S7_, S8_, P1_, P2_, P3_, P4_, P5_, P6_, P7_, P8_] :=
 (sf * V * ((S1 * S8) / (Kms1 * Kms8) - (P1 * P8) / (Kms1 * Kms8 * Keq))) /
  ((1 + S1 / Kms1 + P1 / Kmp1 + S2 / Kms2 + P2 / Kmp2 + S3 / Kms3 +
      P3 / Kmp3 + S4 / Kms4 + P4 / Kmp4 + S5 / Kms5 + P5 / Kmp5 + S6 / Kms6 +
      P6 / Kmp6 + S7 / Kms7 + P7 / Kmp7) * (1 + S8 / Kms8 + P8 / Kmp8))
```

In[•]:=
```
VLCAD[sf_, V_, Kms1_, Kms2_, Kms3_, Kms4_, Kmp1_, Kmp2_,
  Kmp3_, Kmp4_, Keq_, S1_, S2_, S3_, S4_, P1_, P2_, P3_, P4_] :=
 (sf * V * ((S1 * (S4 - P4)) / (Kms1 * Kms4) - (P1 * P4) / (Kms1 * Kms4 * Keq))) /
  ((1 + S1 / Kms1 + P1 / Kmp1 + S2 / Kms2 + P2 / Kmp2 + S3 / Kms3 + P3 / Kmp3) *
    (1 + (S4 - P4) / Kms4 + P4 / Kmp4))
```

```
In[•]:= LCAD[sf_, V_, Kms1_, Kms2_, Kms3_, Kms4_, Kms5_, Kms6_, Kmp1_, Kmp2_, Kmp3_, Kmp4_,
    Kmp5_, Kmp6_, Keq_, S1_, S2_, S3_, S4_, S5_, S6_, P1_, P2_, P3_, P4_, P5_, P6_] :=
   (sf * V * ((S1 * (S6 - P6)) / (Kms1 * Kms6) - (P1 * P6) / (Kms1 * Kms6 * Keq))) /
    ((1 + S1 / Kms1 + P1 / Kmp1 + S2 / Kms2 + P2 / Kmp2 + S3 / Kms3 + P3 / Kmp3 +
        S4 / Kms4 + P4 / Kmp4 + S5 / Kms5 + P5 / Kmp5) * (1 + (S6 - P6) / Kms6 + P6 / Kmp6))

In[•]:= MCAD[sf_, V_, Kms1_, Kms2_, Kms3_, Kms4_, Kms5_, Kms6_, Kmp1_, Kmp2_, Kmp3_, Kmp4_,
    Kmp5_, Kmp6_, Keq_, S1_, S2_, S3_, S4_, S5_, S6_, P1_, P2_, P3_, P4_, P5_, P6_] :=
   (sf * V * ((S1 * (S6 - P6)) / (Kms1 * Kms6) - (P1 * P6) / (Kms1 * Kms6 * Keq))) /
    ((1 + S1 / Kms1 + P1 / Kmp1 + S2 / Kms2 + P2 / Kmp2 + S3 / Kms3 + P3 / Kmp3 +
        S4 / Kms4 + P4 / Kmp4 + S5 / Kms5 + P5 / Kmp5) * (1 + (S6 - P6) / Kms6 + P6 / Kmp6))

In[•]:= SCAD[sf_, V_, Kms1_, Kms2_, Kms3_, Kmp1_, Kmp2_, Kmp3_, Keq_, S1_, S2_, S3_, P1_, P2_,
    P3_] := (sf * V * ((S1 * (S3 - P3)) / (Kms1 * Kms3) - (P1 * P3) / (Kms1 * Kms3 * Keq))) /
    ((1 + S1 / Kms1 + P1 / Kmp1 + S2 / Kms2 + P2 / Kmp2) * (1 + (S3 - P3) / Kms3 + P3 / Kmp3))

In[•]:= CROT[sf_, V_, Kms1_, Kms2_, Kms3_, Kms4_, Kms5_, Kms6_, Kms7_, Kmp1_, Kmp2_, Kmp3_,
    Kmp4_, Kmp5_, Kmp6_, Kmp7_, Ki1_, Keq_, S1_, S2_, S3_, S4_, S5_, S6_, S7_, P1_,
    P2_, P3_, P4_, P5_, P6_, P7_, I1_] := (sf * V * (S1 / Kms1 - P1 / (Kms1 * Keq))) /
    (1 + S1 / Kms1 + P1 / Kmp1 + S2 / Kms2 + P2 / Kmp2 + S3 / Kms3 + P3 / Kmp3 + S4 / Kms4 +
      P4 / Kmp4 + S5 / Kms5 + P5 / Kmp5 + S6 / Kms6 + P6 / Kmp6 + S7 / Kms7 + P7 / Kmp7 + I1 / Ki1)

In[•]:= MSCHAD[sf_, V_, Kms1_, Kms2_, Kms3_, Kms4_, Kms5_, Kms6_, Kms7_, Kms8_,
    Kmp1_, Kmp2_, Kmp3_, Kmp4_, Kmp5_, Kmp6_, Kmp7_, Kmp8_, Keq_, S1_, S2_,
    S3_, S4_, S5_, S6_, S7_, S8_, P1_, P2_, P3_, P4_, P5_, P6_, P7_, P8_] :=
   (sf * V * ((S1 * (S8 - P8)) / (Kms1 * Kms8) - (P1 * P8) / (Kms1 * Kms8 * Keq))) /
    ((1 + S1 / Kms1 + P1 / Kmp1 + S2 / Kms2 + P2 / Kmp2 + S3 / Kms3 +
        P3 / Kmp3 + S4 / Kms4 + P4 / Kmp4 + S5 / Kms5 + P5 / Kmp5 + S6 / Kms6 +
        P6 / Kmp6 + S7 / Kms7 + P7 / Kmp7) * (1 + (S8 - P8) / Kms8 + P8 / Kmp8))

In[•]:= MCKATA[sf_, V_, Kms1_, Kms2_, Kms3_, Kms4_, Kms5_, Kms6_, Kms7_, Kms8_,
    Kmp1_, Kmp2_, Kmp3_, Kmp4_, Kmp5_, Kmp6_, Kmp7_, Kmp8_, Keq_, S1_, S2_,
    S3_, S4_, S5_, S6_, S7_, S8_, P1_, P2_, P3_, P4_, P5_, P6_, P7_, P8_] :=
   (sf * V * ((S1 * S8) / (Kms1 * Kms8) - (P1 * P8) / (Kms1 * Kms8 * Keq))) /
    ((1 + S1 / Kms1 + P1 / Kmp1 + S2 / Kms2 + P2 / Kmp2 + S3 / Kms3 +
        P3 / Kmp3 + S4 / Kms4 + P4 / Kmp4 + S5 / Kms5 + P5 / Kmp5 + S6 / Kms6 +
        P6 / Kmp6 + S7 / Kms7 + P7 / Kmp7 + P8 / Kmp8) * (1 + S8 / Kms8 + P8 / Kmp8))

In[•]:= MCKATB[sf_, V_, Kms1_, Kms2_, Kms3_, Kms4_, Kms5_, Kms6_, Kms7_, Kms8_,
    Kmp1_, Kmp2_, Kmp3_, Kmp4_, Kmp5_, Kmp6_, Kmp7_, Kmp8_, Keq_, S1_, S2_,
    S3_, S4_, S5_, S6_, S7_, S8_, P1_, P2_, P3_, P4_, P5_, P6_, P7_, P8_] :=
   (sf * V * ((S1 * S8) / (Kms1 * Kms8) - (P8 * P8) / (Kms1 * Kms8 * Keq))) /
    ((1 + S1 / Kms1 + P1 / Kmp1 + S2 / Kms2 + P2 / Kmp2 + S3 / Kms3 +
        P3 / Kmp3 + S4 / Kms4 + P4 / Kmp4 + S5 / Kms5 + P5 / Kmp5 + S6 / Kms6 +
        P6 / Kmp6 + S7 / Kms7 + P7 / Kmp7 + P8 / Kmp8) * (1 + S8 / Kms8 + P8 / Kmp8))

In[•]:= MTP[sf_, V_, Kms1_, Kms2_, Kms3_, Kms4_, Kms5_, Kms7_, Kms8_, Kmp1_,
    Kmp2_, Kmp3_, Kmp4_, Kmp5_, Kmp6_, Kmp7_, Kmp8_, Ki1_, Keq_, S1_, S2_, S3_,
    S4_, S5_, S7_, S8_, P1_, P2_, P3_, P4_, P5_, P6_, P7_, P8_, I1_] := (sf * V *
      ((S1 * (S7 - P7) * S8) / (Kms1 * Kms7 * Kms8) - (P1 * P7 * P8) / (Kms1 * Kms7 * Kms8 * Keq))) /
    ((1 + S1 / Kms1 + P1 / Kmp1 + S2 / Kms2 + P2 / Kmp2 + S3 / Kms3 + P3 / Kmp3 +
        S4 / Kms4 + P4 / Kmp4 + S5 / Kms5 + P5 / Kmp5 + P6 / Kmp6 + I1 / Ki1) *
      (1 + (S7 - P7) / Kms7 + P7 / Kmp7) * (1 + S8 / Kms8 + P8 / Kmp8))

In[•]:= RES[Ks_, S_, K1_] := Ks * (S - K1)
```

Define the differential equations

```
In[•]:= Odes = {
     C16AcylCarCYT'[t] == (vcpt1C16 - vcactC16) / VCYT,
```

```
   C16AcylCarMAT'[t] == (vcactC16 - vcpt2C16) / VMAT,
   C16AcylCoAMAT'[t] == (vcpt2C16 - vvlcadC16 - vlcadC16) / VMAT,
   C16EnoylCoAMAT'[t] == (vvlcadC16 + vlcadC16 - vcrotC16 - vmtpC16) / VMAT,
   C16HydroxyacylCoAMAT'[t] == (vcrotC16 - vmschadC16) / VMAT,
   C16KetoacylCoAMAT'[t] == (vmschadC16 - vmckatC16) / VMAT,
   C14AcylCarCYT'[t] == (-vcactC14) / VCYT,
   C14AcylCarMAT'[t] == (vcactC14 - vcpt2C14) / VMAT,
   C14AcylCoAMAT'[t] == (vcpt2C14 + vmtpC16 + vmckatC16 - vvlcadC14 - vlcadC14) / VMAT,
   C14EnoylCoAMAT'[t] == (vvlcadC14 + vlcadC14 - vcrotC14 - vmtpC14) / VMAT,
   C14HydroxyacylCoAMAT'[t] == (vcrotC14 - vmschadC14) / VMAT,
   C14KetoacylCoAMAT'[t] == (vmschadC14 - vmckatC14) / VMAT,
   C12AcylCarCYT'[t] == (-vcactC12) / VCYT,
   C12AcylCarMAT'[t] == (vcactC12 - vcpt2C12) / VMAT,
   C12AcylCoAMAT'[t] ==
    (vcpt2C12 + vmtpC14 + vmckatC14 - vvlcadC12 - vlcadC12 - vmcadC12) / VMAT,
   C12EnoylCoAMAT'[t] == (vvlcadC12 + vlcadC12 + vmcadC12 - vcrotC12 - vmtpC12) / VMAT,
   C12HydroxyacylCoAMAT'[t] == (vcrotC12 - vmschadC12) / VMAT,
   C12KetoacylCoAMAT'[t] == (vmschadC12 - vmckatC12) / VMAT,
   C10AcylCarCYT'[t] == (-vcactC10) / VCYT,
   C10AcylCarMAT'[t] == (vcactC10 - vcpt2C10) / VMAT,
   C10AcylCoAMAT'[t] == (vcpt2C10 + vmtpC12 + vmckatC12 - vlcadC10 - vmcadC10) / VMAT,
   C10EnoylCoAMAT'[t] == (vlcadC10 + vmcadC10 - vcrotC10 - vmtpC10) / VMAT,
   C10HydroxyacylCoAMAT'[t] == (vcrotC10 - vmschadC10) / VMAT,
   C10KetoacylCoAMAT'[t] == (vmschadC10 - vmckatC10) / VMAT,
   C8AcylCarCYT'[t] == (-vcactC8) / VCYT,
   C8AcylCarMAT'[t] == (vcactC8 - vcpt2C8) / VMAT,
   C8AcylCoAMAT'[t] == (vcpt2C8 + vmtpC10 + vmckatC10 - vlcadC8 - vmcadC8) / VMAT,
   C8EnoylCoAMAT'[t] == (vlcadC8 + vmcadC8 - vcrotC8 - vmtpC8) / VMAT,
   C8HydroxyacylCoAMAT'[t] == (vcrotC8 - vmschadC8) / VMAT,
   C8KetoacylCoAMAT'[t] == (vmschadC8 - vmckatC8) / VMAT,
   C6AcylCarCYT'[t] == (-vcactC6) / VCYT,
   C6AcylCarMAT'[t] == (vcactC6 - vcpt2C6) / VMAT,
   C6AcylCoAMAT'[t] == (vcpt2C6 + vmtpC8 + vmckatC8 - vmcadC6 - vscadC6) / VMAT,
   C6EnoylCoAMAT'[t] == (vmcadC6 + vscadC6 - vcrotC6) / VMAT,
   C6HydroxyacylCoAMAT'[t] == (vcrotC6 - vmschadC6) / VMAT,
   C6KetoacylCoAMAT'[t] == (vmschadC6 - vmckatC6) / VMAT,
   C4AcylCarCYT'[t] == (-vcactC4) / VCYT,
   C4AcylCarMAT'[t] == (vcactC4 - vcpt2C4) / VMAT,
   C4AcylCoAMAT'[t] == (vcpt2C4 + vmckatC6 - vmcadC4 - vscadC4) / VMAT,
   C4EnoylCoAMAT'[t] == (vmcadC4 + vscadC4 - vcrotC4) / VMAT,
   C4HydroxyacylCoAMAT'[t] == (vcrotC4 - vmschadC4) / VMAT,
   C4AcetoacylCoAMAT'[t] == (vmschadC4 - vmckatC4) / VMAT,
   AcetylCoAMAT'[t] ==
    (1 / VMAT) (vmtpC16 + vmckatC16 + vmtpC14 + vmckatC14 + vmtpC12 + vmckatC12 +
       vmtpC10 + vmckatC10 + vmtpC8 + vmckatC8 + vmckatC6 + 2 * vmckatC4 - vacesink),
   FADHMAT'[t] == (1 / VMAT) (vvlcadC16 + vvlcadC14 + vvlcadC12 + vlcadC16 +
       vlcadC14 + vlcadC12 + vlcadC10 + vlcadC8 + vmcadC12 + vmcadC10 +
       vmcadC8 + vmcadC6 + vmcadC4 + vscadC6 + vscadC4 - vfadhsink),
   NADHMAT'[t] == (1 / VMAT) (vmtpC16 + vmtpC14 + vmtpC12 + vmtpC10 +
       vmtpC8 + vmschadC16 + vmschadC14 + vmschadC12 + vmschadC10 +
       vmschadC8 + vmschadC6 + vmschadC4 - vnadhsink)};

RateEqs = {vcpt1C16 → CPT1[sfcpt1C16, Vcpt1, Kmcpt1C16AcylCoACYT,
      Kmcpt1CarCYT, Kmcpt1C16AcylCarCYT, Kmcpt1CoACYT, Kicpt1MalCoACYT, Keqcpt1,
      C16AcylCoACYT, CarCYT, C16AcylCarCYT[t], CoACYT, MalCoACYT, ncpt1],
```

```
vcactC16 → CACT[Vfcact, Vrcact, KmcactC16AcylCarCYT, KmcactCarMAT,
   KmcactC16AcylCarMAT, KmcactCarCYT, KicactC16AcylCarCYT, KicactCarCYT,
   Keqcact, C16AcylCarCYT[t], CarMAT, C16AcylCarMAT[t], CarCYT],
vcactC14 → CACT[Vfcact, Vrcact, KmcactC14AcylCarCYT, KmcactCarMAT,
   KmcactC14AcylCarMAT, KmcactCarCYT, KicactC14AcylCarCYT, KicactCarCYT,
   Keqcact, C14AcylCarCYT[t], CarMAT, C14AcylCarMAT[t], CarCYT],
vcactC12 → CACT[Vfcact, Vrcact, KmcactC12AcylCarCYT, KmcactCarMAT,
   KmcactC12AcylCarMAT, KmcactCarCYT, KicactC12AcylCarCYT, KicactCarCYT,
   Keqcact, C12AcylCarCYT[t], CarMAT, C12AcylCarMAT[t], CarCYT],
vcactC10 → CACT[Vfcact, Vrcact, KmcactC10AcylCarCYT, KmcactCarMAT,
   KmcactC10AcylCarMAT, KmcactCarCYT, KicactC10AcylCarCYT, KicactCarCYT,
   Keqcact, C10AcylCarCYT[t], CarMAT, C10AcylCarMAT[t], CarCYT],
vcactC8 → CACT[Vfcact, Vrcact, KmcactC8AcylCarCYT, KmcactCarMAT,
   KmcactC8AcylCarMAT, KmcactCarCYT, KicactC8AcylCarCYT, KicactCarCYT,
   Keqcact, C8AcylCarCYT[t], CarMAT, C8AcylCarMAT[t], CarCYT],
vcactC6 → CACT[Vfcact, Vrcact, KmcactC6AcylCarCYT, KmcactCarMAT,
   KmcactC6AcylCarMAT, KmcactCarCYT, KicactC6AcylCarCYT, KicactCarCYT,
   Keqcact, C6AcylCarCYT[t], CarMAT, C6AcylCarMAT[t], CarCYT],
vcactC4 → CACT[Vfcact, Vrcact, KmcactC4AcylCarCYT, KmcactCarMAT,
   KmcactC4AcylCarMAT, KmcactCarCYT, KicactC4AcylCarCYT, KicactCarCYT,
   Keqcact, C4AcylCarCYT[t], CarMAT, C4AcylCarMAT[t], CarCYT],
vcpt2C16 → CPT2[sfcpt2C16, Vcpt2, Kmcpt2C16AcylCarMAT, Kmcpt2C14AcylCarMAT,
   Kmcpt2C12AcylCarMAT, Kmcpt2C10AcylCarMAT, Kmcpt2C8AcylCarMAT, Kmcpt2C6AcylCarMAT,
   Kmcpt2C4AcylCarMAT, Kmcpt2CoAMAT, Kmcpt2C16AcylCoAMAT, Kmcpt2C14AcylCoAMAT,
   Kmcpt2C12AcylCoAMAT, Kmcpt2C10AcylCoAMAT, Kmcpt2C8AcylCoAMAT, Kmcpt2C6AcylCoAMAT,
   Kmcpt2C4AcylCoAMAT, Kmcpt2CarMAT, Keqcpt2, C16AcylCarMAT[t], C14AcylCarMAT[t],
   C12AcylCarMAT[t], C10AcylCarMAT[t], C8AcylCarMAT[t], C6AcylCarMAT[t],
   C4AcylCarMAT[t], CoAMAT, C16AcylCoAMAT[t], C14AcylCoAMAT[t], C12AcylCoAMAT[t],
   C10AcylCoAMAT[t], C8AcylCoAMAT[t], C6AcylCoAMAT[t], C4AcylCoAMAT[t], CarMAT],
vcpt2C14 → CPT2[sfcpt2C14, Vcpt2, Kmcpt2C14AcylCarMAT, Kmcpt2C16AcylCarMAT,
   Kmcpt2C12AcylCarMAT, Kmcpt2C10AcylCarMAT, Kmcpt2C8AcylCarMAT, Kmcpt2C6AcylCarMAT,
   Kmcpt2C4AcylCoAMAT, Kmcpt2CoAMAT, Kmcpt2C14AcylCoAMAT, Kmcpt2C16AcylCoAMAT,
   Kmcpt2C12AcylCoAMAT, Kmcpt2C10AcylCoAMAT, Kmcpt2C8AcylCoAMAT, Kmcpt2C6AcylCoAMAT,
   Kmcpt2C4AcylCoAMAT, Kmcpt2CarMAT, Keqcpt2, C14AcylCarMAT[t], C16AcylCarMAT[t],
   C12AcylCarMAT[t], C10AcylCarMAT[t], C8AcylCarMAT[t], C6AcylCarMAT[t],
   C4AcylCarMAT[t], CoAMAT, C14AcylCoAMAT[t], C16AcylCoAMAT[t], C12AcylCoAMAT[t],
   C10AcylCoAMAT[t], C8AcylCoAMAT[t], C6AcylCoAMAT[t], C4AcylCoAMAT[t], CarMAT],
vcpt2C12 → CPT2[sfcpt2C12, Vcpt2, Kmcpt2C12AcylCarMAT, Kmcpt2C16AcylCarMAT,
   Kmcpt2C14AcylCarMAT, Kmcpt2C10AcylCarMAT, Kmcpt2C8AcylCarMAT, Kmcpt2C6AcylCarMAT,
   Kmcpt2C4AcylCarMAT, Kmcpt2CoAMAT, Kmcpt2C12AcylCoAMAT, Kmcpt2C16AcylCoAMAT,
   Kmcpt2C14AcylCoAMAT, Kmcpt2C10AcylCoAMAT, Kmcpt2C8AcylCoAMAT, Kmcpt2C6AcylCoAMAT,
   Kmcpt2C4AcylCoAMAT, Kmcpt2CarMAT, Keqcpt2, C12AcylCarMAT[t], C16AcylCarMAT[t],
   C14AcylCarMAT[t], C10AcylCarMAT[t], C8AcylCarMAT[t], C6AcylCarMAT[t],
   C4AcylCarMAT[t], CoAMAT, C12AcylCoAMAT[t], C16AcylCoAMAT[t], C14AcylCoAMAT[t],
   C10AcylCoAMAT[t], C8AcylCoAMAT[t], C6AcylCoAMAT[t], C4AcylCoAMAT[t], CarMAT],
vcpt2C10 → CPT2[sfcpt2C10, Vcpt2, Kmcpt2C10AcylCarMAT, Kmcpt2C16AcylCarMAT,
   Kmcpt2C14AcylCarMAT, Kmcpt2C12AcylCarMAT, Kmcpt2C8AcylCarMAT, Kmcpt2C6AcylCarMAT,
   Kmcpt2C4AcylCarMAT, Kmcpt2CoAMAT, Kmcpt2C10AcylCoAMAT, Kmcpt2C16AcylCoAMAT,
   Kmcpt2C14AcylCoAMAT, Kmcpt2C12AcylCoAMAT, Kmcpt2C8AcylCoAMAT, Kmcpt2C6AcylCoAMAT,
   Kmcpt2C4AcylCoAMAT, Kmcpt2CarMAT, Keqcpt2, C10AcylCarMAT[t], C16AcylCarMAT[t],
   C14AcylCarMAT[t], C12AcylCarMAT[t], C8AcylCarMAT[t], C6AcylCarMAT[t],
   C4AcylCarMAT[t], CoAMAT, C10AcylCoAMAT[t], C16AcylCoAMAT[t], C14AcylCoAMAT[t],
   C12AcylCoAMAT[t], C8AcylCoAMAT[t], C6AcylCoAMAT[t], C4AcylCoAMAT[t], CarMAT],
vcpt2C8 → CPT2[sfcpt2C8, Vcpt2, Kmcpt2C8AcylCarMAT, Kmcpt2C16AcylCarMAT,
   Kmcpt2C14AcylCarMAT, Kmcpt2C12AcylCarMAT, Kmcpt2C10AcylCarMAT, Kmcpt2C6AcylCarMAT,
```

```
   Kmcpt2C4AcylCarMAT, Kmcpt2CoAMAT, Kmcpt2C8AcylCoAMAT, Kmcpt2C16AcylCoAMAT,
   Kmcpt2C14AcylCoAMAT, Kmcpt2C12AcylCoAMAT, Kmcpt2C10AcylCoAMAT, Kmcpt2C6AcylCoAMAT,
   Kmcpt2C4AcylCoAMAT, Kmcpt2CarMAT, Keqcpt2, C8AcylCarMAT[t], C16AcylCarMAT[t],
   C14AcylCarMAT[t], C12AcylCarMAT[t], C10AcylCarMAT[t], C6AcylCarMAT[t],
   C4AcylCarMAT[t], CoAMAT, C8AcylCoAMAT[t], C16AcylCoAMAT[t], C14AcylCoAMAT[t],
   C12AcylCoAMAT[t], C10AcylCoAMAT[t], C6AcylCoAMAT[t], C4AcylCoAMAT[t], CarMAT],
vcpt2C6 → CPT2[sfcpt2C6, Vcpt2, Kmcpt2C6AcylCarMAT, Kmcpt2C16AcylCarMAT,
   Kmcpt2C14AcylCarMAT, Kmcpt2C12AcylCarMAT, Kmcpt2C10AcylCarMAT, Kmcpt2C8AcylCarMAT,
   Kmcpt2C4AcylCarMAT, Kmcpt2CoAMAT, Kmcpt2C6AcylCoAMAT, Kmcpt2C16AcylCoAMAT,
   Kmcpt2C14AcylCoAMAT, Kmcpt2C12AcylCoAMAT, Kmcpt2C10AcylCoAMAT, Kmcpt2C8AcylCoAMAT,
   Kmcpt2C4AcylCoAMAT, Kmcpt2CarMAT, Keqcpt2, C6AcylCarMAT[t], C16AcylCarMAT[t],
   C14AcylCarMAT[t], C12AcylCarMAT[t], C10AcylCarMAT[t], C8AcylCarMAT[t],
   C4AcylCarMAT[t], CoAMAT, C6AcylCoAMAT[t], C16AcylCoAMAT[t], C14AcylCoAMAT[t],
   C12AcylCoAMAT[t], C10AcylCoAMAT[t], C8AcylCoAMAT[t], C4AcylCoAMAT[t], CarMAT],
vcpt2C4 → CPT2[sfcpt2C4, Vcpt2, Kmcpt2C4AcylCarMAT, Kmcpt2C16AcylCarMAT,
   Kmcpt2C14AcylCarMAT, Kmcpt2C12AcylCarMAT, Kmcpt2C10AcylCarMAT, Kmcpt2C8AcylCarMAT,
   Kmcpt2C6AcylCarMAT, Kmcpt2CoAMAT, Kmcpt2C4AcylCoAMAT, Kmcpt2C16AcylCoAMAT,
   Kmcpt2C14AcylCoAMAT, Kmcpt2C12AcylCoAMAT, Kmcpt2C10AcylCoAMAT, Kmcpt2C8AcylCoAMAT,
   Kmcpt2C6AcylCoAMAT, Kmcpt2CarMAT, Keqcpt2, C4AcylCarMAT[t], C16AcylCarMAT[t],
   C14AcylCarMAT[t], C12AcylCarMAT[t], C10AcylCarMAT[t], C8AcylCarMAT[t],
   C6AcylCarMAT[t], CoAMAT, C4AcylCoAMAT[t], C16AcylCoAMAT[t], C14AcylCoAMAT[t],
   C12AcylCoAMAT[t], C10AcylCoAMAT[t], C8AcylCoAMAT[t], C6AcylCoAMAT[t], CarMAT],
vvlcadC16 → VLCAD[sfvlcadC16, Vvlcad, KmvlcadC16AcylCoAMAT, KmvlcadC14AcylCoAMAT,
   KmvlcadC12AcylCoAMAT, KmvlcadFAD, KmvlcadC16EnoylCoAMAT,
   KmvlcadC14EnoylCoAMAT, KmvlcadC12EnoylCoAMAT, KmvlcadFADH, Keqvlcad,
   C16AcylCoAMAT[t], C14AcylCoAMAT[t], C12AcylCoAMAT[t], FADtMAT,
   C16EnoylCoAMAT[t], C14EnoylCoAMAT[t], C12EnoylCoAMAT[t], FADHMAT[t]],
vvlcadC14 → VLCAD[sfvlcadC14, Vvlcad, KmvlcadC14AcylCoAMAT, KmvlcadC16AcylCoAMAT,
   KmvlcadC12AcylCoAMAT, KmvlcadFAD, KmvlcadC14EnoylCoAMAT,
   KmvlcadC16EnoylCoAMAT, KmvlcadC12EnoylCoAMAT, KmvlcadFADH, Keqvlcad,
   C14AcylCoAMAT[t], C16AcylCoAMAT[t], C12AcylCoAMAT[t], FADtMAT,
   C14EnoylCoAMAT[t], C16EnoylCoAMAT[t], C12EnoylCoAMAT[t], FADHMAT[t]],
vvlcadC12 → VLCAD[sfvlcadC12, Vvlcad, KmvlcadC12AcylCoAMAT, KmvlcadC16AcylCoAMAT,
   KmvlcadC14AcylCoAMAT, KmvlcadFAD, KmvlcadC12EnoylCoAMAT,
   KmvlcadC16EnoylCoAMAT, KmvlcadC14EnoylCoAMAT, KmvlcadFADH, Keqvlcad,
   C12AcylCoAMAT[t], C16AcylCoAMAT[t], C14AcylCoAMAT[t], FADtMAT,
   C12EnoylCoAMAT[t], C16EnoylCoAMAT[t], C14EnoylCoAMAT[t], FADHMAT[t]],
vlcadC16 → LCAD[sflcadC16, Vlcad, KmlcadC16AcylCoAMAT, KmlcadC14AcylCoAMAT,
   KmlcadC12AcylCoAMAT, KmlcadC10AcylCoAMAT, KmlcadC8AcylCoAMAT, KmlcadFAD,
   KmlcadC16EnoylCoAMAT, KmlcadC14EnoylCoAMAT, KmlcadC12EnoylCoAMAT,
   KmlcadC10EnoylCoAMAT, KmlcadC8EnoylCoAMAT, KmlcadFADH, Keqlcad,
   C16AcylCoAMAT[t], C14AcylCoAMAT[t], C12AcylCoAMAT[t], C10AcylCoAMAT[t],
   C8AcylCoAMAT[t], FADtMAT, C16EnoylCoAMAT[t], C14EnoylCoAMAT[t],
   C12EnoylCoAMAT[t], C10EnoylCoAMAT[t], C8EnoylCoAMAT[t], FADHMAT[t]],
vlcadC14 → LCAD[sflcadC14, Vlcad, KmlcadC14AcylCoAMAT, KmlcadC16AcylCoAMAT,
   KmlcadC12AcylCoAMAT, KmlcadC10AcylCoAMAT, KmlcadC8AcylCoAMAT, KmlcadFAD,
   KmlcadC14EnoylCoAMAT, KmlcadC16EnoylCoAMAT, KmlcadC12EnoylCoAMAT,
   KmlcadC10EnoylCoAMAT, KmlcadC8EnoylCoAMAT, KmlcadFADH, Keqlcad,
   C14AcylCoAMAT[t], C16AcylCoAMAT[t], C12AcylCoAMAT[t], C10AcylCoAMAT[t],
   C8AcylCoAMAT[t], FADtMAT, C14EnoylCoAMAT[t], C16EnoylCoAMAT[t],
   C12EnoylCoAMAT[t], C10EnoylCoAMAT[t], C8EnoylCoAMAT[t], FADHMAT[t]],
vlcadC12 → LCAD[sflcadC12, Vlcad, KmlcadC12AcylCoAMAT, KmlcadC16AcylCoAMAT,
   KmlcadC14AcylCoAMAT, KmlcadC10AcylCoAMAT, KmlcadC8AcylCoAMAT, KmlcadFAD,
   KmlcadC12EnoylCoAMAT, KmlcadC16EnoylCoAMAT, KmlcadC14EnoylCoAMAT,
   KmlcadC10EnoylCoAMAT, KmlcadC8EnoylCoAMAT, KmlcadFADH, Keqlcad,
```

```
    C12AcylCoAMAT[t], C16AcylCoAMAT[t], C14AcylCoAMAT[t], C10AcylCoAMAT[t],
    C8AcylCoAMAT[t], FADtMAT, C14EnoylCoAMAT[t], C16EnoylCoAMAT[t],
    C14EnoylCoAMAT[t], C10EnoylCoAMAT[t], C8EnoylCoAMAT[t], FADHMAT[t]],
  vlcadC10 → LCAD[sflcadC10, Vlcad, KmlcadC10AcylCoAMAT, KmlcadC16AcylCoAMAT,
    KmlcadC14AcylCoAMAT, KmlcadC12AcylCoAMAT, KmlcadC8AcylCoAMAT, KmlcadFAD,
    KmlcadC10EnoylCoAMAT, KmlcadC16EnoylCoAMAT, KmlcadC14EnoylCoAMAT,
    KmlcadC12EnoylCoAMAT, KmlcadC8EnoylCoAMAT, KmlcadFADH, Keqlcad,
    C10AcylCoAMAT[t], C16AcylCoAMAT[t], C14AcylCoAMAT[t], C12AcylCoAMAT[t],
    C8AcylCoAMAT[t], FADtMAT, C10EnoylCoAMAT[t], C16EnoylCoAMAT[t],
    C14EnoylCoAMAT[t], C12EnoylCoAMAT[t], C8EnoylCoAMAT[t], FADHMAT[t]],
  vlcadC8 → LCAD[sflcadC8, Vlcad, KmlcadC8AcylCoAMAT, KmlcadC16AcylCoAMAT,
    KmlcadC14AcylCoAMAT, KmlcadC12AcylCoAMAT, KmlcadC10AcylCoAMAT, KmlcadFAD,
    KmlcadC8EnoylCoAMAT, KmlcadC16EnoylCoAMAT, KmlcadC14EnoylCoAMAT,
    KmlcadC12EnoylCoAMAT, KmlcadC10EnoylCoAMAT, KmlcadFADH, Keqlcad,
    C8AcylCoAMAT[t], C16AcylCoAMAT[t], C14AcylCoAMAT[t], C12AcylCoAMAT[t],
    C10AcylCoAMAT[t], FADtMAT, C8EnoylCoAMAT[t], C16EnoylCoAMAT[t],
    C14EnoylCoAMAT[t], C12EnoylCoAMAT[t], C10EnoylCoAMAT[t], FADHMAT[t]],
  vmcadC12 → MCAD[sfmcadC12, Vmcad, KmmcadC12AcylCoAMAT, KmmcadC10AcylCoAMAT,
    KmmcadC8AcylCoAMAT, KmmcadC6AcylCoAMAT, KmmcadC4AcylCoAMAT, KmmcadFAD,
    KmmcadC12EnoylCoAMAT, KmmcadC10EnoylCoAMAT, KmmcadC8EnoylCoAMAT,
    KmmcadC6EnoylCoAMAT, KmmcadC4EnoylCoAMAT, KmmcadFADH, Keqmcad,
    C12AcylCoAMAT[t], C10AcylCoAMAT[t], C8AcylCoAMAT[t], C6AcylCoAMAT[t],
    C4AcylCoAMAT[t], FADtMAT, C12EnoylCoAMAT[t], C10EnoylCoAMAT[t],
    C8EnoylCoAMAT[t], C6EnoylCoAMAT[t], C4EnoylCoAMAT[t], FADHMAT[t]],
  vmcadC10 → MCAD[sfmcadC10, Vmcad, KmmcadC10AcylCoAMAT, KmmcadC12AcylCoAMAT,
    KmmcadC8AcylCoAMAT, KmmcadC6AcylCoAMAT, KmmcadC4AcylCoAMAT, KmmcadFAD,
    KmmcadC10EnoylCoAMAT, KmmcadC12EnoylCoAMAT, KmmcadC8EnoylCoAMAT,
    KmmcadC6EnoylCoAMAT, KmmcadC4EnoylCoAMAT, KmmcadFADH, Keqmcad,
    C10AcylCoAMAT[t], C12AcylCoAMAT[t], C8AcylCoAMAT[t], C6AcylCoAMAT[t],
    C4AcylCoAMAT[t], FADtMAT, C10EnoylCoAMAT[t], C12EnoylCoAMAT[t],
    C8EnoylCoAMAT[t], C6EnoylCoAMAT[t], C4EnoylCoAMAT[t], FADHMAT[t]],
  vmcadC8 → MCAD[sfmcadC8, Vmcad, KmmcadC8AcylCoAMAT, KmmcadC12AcylCoAMAT,
    KmmcadC10AcylCoAMAT, KmmcadC6AcylCoAMAT, KmmcadC4AcylCoAMAT, KmmcadFAD,
    KmmcadC8EnoylCoAMAT, KmmcadC12EnoylCoAMAT, KmmcadC10EnoylCoAMAT,
    KmmcadC6EnoylCoAMAT, KmmcadC4EnoylCoAMAT, KmmcadFADH, Keqmcad,
    C8AcylCoAMAT[t], C12AcylCoAMAT[t], C10AcylCoAMAT[t], C6AcylCoAMAT[t],
    C4AcylCoAMAT[t], FADtMAT, C8EnoylCoAMAT[t], C12EnoylCoAMAT[t],
    C10EnoylCoAMAT[t], C6EnoylCoAMAT[t], C4EnoylCoAMAT[t], FADHMAT[t]],
  vmcadC6 → MCAD[sfmcadC6, Vmcad, KmmcadC6AcylCoAMAT, KmmcadC12AcylCoAMAT,
    KmmcadC10AcylCoAMAT, KmmcadC8AcylCoAMAT, KmmcadC4AcylCoAMAT, KmmcadFAD,
    KmmcadC6EnoylCoAMAT, KmmcadC12EnoylCoAMAT, KmmcadC10EnoylCoAMAT,
    KmmcadC8EnoylCoAMAT, KmmcadC4EnoylCoAMAT, KmmcadFADH, Keqmcad,
    C6AcylCoAMAT[t], C12AcylCoAMAT[t], C10AcylCoAMAT[t], C8AcylCoAMAT[t],
    C4AcylCoAMAT[t], FADtMAT, C6EnoylCoAMAT[t], C12EnoylCoAMAT[t],
    C10EnoylCoAMAT[t], C8EnoylCoAMAT[t], C4EnoylCoAMAT[t], FADHMAT[t]],
  vmcadC4 → MCAD[sfmcadC4, Vmcad, KmmcadC4AcylCoAMAT, KmmcadC12AcylCoAMAT,
    KmmcadC10AcylCoAMAT, KmmcadC8AcylCoAMAT, KmmcadC6AcylCoAMAT, KmmcadFAD,
    KmmcadC4EnoylCoAMAT, KmmcadC12EnoylCoAMAT, KmmcadC10EnoylCoAMAT,
    KmmcadC8EnoylCoAMAT, KmmcadC6EnoylCoAMAT, KmmcadFADH, Keqmcad,
    C4AcylCoAMAT[t], C12AcylCoAMAT[t], C10AcylCoAMAT[t], C8AcylCoAMAT[t],
    C6AcylCoAMAT[t], FADtMAT, C4EnoylCoAMAT[t], C12EnoylCoAMAT[t],
    C10EnoylCoAMAT[t], C8EnoylCoAMAT[t], C6EnoylCoAMAT[t], FADHMAT[t]],
  vscadC6 → SCAD[sfscadC6, Vscad, KmscadC6AcylCoAMAT, KmscadC4AcylCoAMAT, KmscadFAD,
    KmscadC6EnoylCoAMAT, KmscadC4EnoylCoAMAT, KmscadFADH, Keqscad, C6AcylCoAMAT[t],
    C4AcylCoAMAT[t], FADtMAT, C6EnoylCoAMAT[t], C4EnoylCoAMAT[t], FADHMAT[t]],
```

```
vscadC4 → SCAD[sfscadC4, Vscad, KmscadC4AcylCoAMAT, KmscadC6AcylCoAMAT, KmscadFAD,
   KmscadC4EnoylCoAMAT, KmscadC6EnoylCoAMAT, KmscadFADH, Keqscad, C4AcylCoAMAT[t],
   C6AcylCoAMAT[t], FADtMAT, C4EnoylCoAMAT[t], C6EnoylCoAMAT[t], FADHMAT[t]],
vcrotC16 → CROT[sfcrotC16, Vcrot, KmcrotC16EnoylCoAMAT, KmcrotC14EnoylCoAMAT,
   KmcrotC12EnoylCoAMAT, KmcrotC10EnoylCoAMAT, KmcrotC8EnoylCoAMAT,
   KmcrotC6EnoylCoAMAT, KmcrotC4EnoylCoAMAT, KmcrotC16HydroxyacylCoAMAT,
   KmcrotC14HydroxyacylCoAMAT, KmcrotC12HydroxyacylCoAMAT,
   KmcrotC10HydroxyacylCoAMAT, KmcrotC8HydroxyacylCoAMAT,
   KmcrotC6HydroxyacylCoAMAT, KmcrotC4HydroxyacylCoAMAT, KicrotC4AcetoacylCoA,
   Keqcrot, C16EnoylCoAMAT[t], C14EnoylCoAMAT[t], C12EnoylCoAMAT[t],
   C10EnoylCoAMAT[t], C8EnoylCoAMAT[t], C6EnoylCoAMAT[t],
   C4EnoylCoAMAT[t], C16HydroxyacylCoAMAT[t], C14HydroxyacylCoAMAT[t],
   C12HydroxyacylCoAMAT[t], C10HydroxyacylCoAMAT[t], C8HydroxyacylCoAMAT[t],
   C6HydroxyacylCoAMAT[t], C4HydroxyacylCoAMAT[t], C4AcetoacylCoAMAT[t]],
vcrotC14 → CROT[sfcrotC14, Vcrot, KmcrotC14EnoylCoAMAT, KmcrotC16EnoylCoAMAT,
   KmcrotC12EnoylCoAMAT, KmcrotC10EnoylCoAMAT, KmcrotC8EnoylCoAMAT,
   KmcrotC6EnoylCoAMAT, KmcrotC4EnoylCoAMAT, KmcrotC14HydroxyacylCoAMAT,
   KmcrotC16HydroxyacylCoAMAT, KmcrotC12HydroxyacylCoAMAT,
   KmcrotC10HydroxyacylCoAMAT, KmcrotC8HydroxyacylCoAMAT,
   KmcrotC6HydroxyacylCoAMAT, KmcrotC4HydroxyacylCoAMAT, KicrotC4AcetoacylCoA,
   Keqcrot, C14EnoylCoAMAT[t], C16EnoylCoAMAT[t], C12EnoylCoAMAT[t],
   C10EnoylCoAMAT[t], C8EnoylCoAMAT[t], C6EnoylCoAMAT[t],
   C4EnoylCoAMAT[t], C14HydroxyacylCoAMAT[t], C16HydroxyacylCoAMAT[t],
   C12HydroxyacylCoAMAT[t], C10HydroxyacylCoAMAT[t], C8HydroxyacylCoAMAT[t],
   C6HydroxyacylCoAMAT[t], C4HydroxyacylCoAMAT[t], C4AcetoacylCoAMAT[t]],
vcrotC12 → CROT[sfcrotC12, Vcrot, KmcrotC12EnoylCoAMAT, KmcrotC16EnoylCoAMAT,
   KmcrotC14EnoylCoAMAT, KmcrotC10EnoylCoAMAT, KmcrotC8EnoylCoAMAT,
   KmcrotC6EnoylCoAMAT, KmcrotC4EnoylCoAMAT, KmcrotC12HydroxyacylCoAMAT,
   KmcrotC16HydroxyacylCoAMAT, KmcrotC14HydroxyacylCoAMAT,
   KmcrotC10HydroxyacylCoAMAT, KmcrotC8HydroxyacylCoAMAT,
   KmcrotC6HydroxyacylCoAMAT, KmcrotC4HydroxyacylCoAMAT, KicrotC4AcetoacylCoA,
   Keqcrot, C12EnoylCoAMAT[t], C16EnoylCoAMAT[t], C14EnoylCoAMAT[t],
   C10EnoylCoAMAT[t], C8EnoylCoAMAT[t], C6EnoylCoAMAT[t],
   C4EnoylCoAMAT[t], C12HydroxyacylCoAMAT[t], C16HydroxyacylCoAMAT[t],
   C14HydroxyacylCoAMAT[t], C10HydroxyacylCoAMAT[t], C8HydroxyacylCoAMAT[t],
   C6HydroxyacylCoAMAT[t], C4HydroxyacylCoAMAT[t], C4AcetoacylCoAMAT[t]],
vcrotC10 → CROT[sfcrotC10, Vcrot, KmcrotC10EnoylCoAMAT, KmcrotC16EnoylCoAMAT,
   KmcrotC14EnoylCoAMAT, KmcrotC12EnoylCoAMAT, KmcrotC8EnoylCoAMAT,
   KmcrotC6EnoylCoAMAT, KmcrotC4EnoylCoAMAT, KmcrotC10HydroxyacylCoAMAT,
   KmcrotC16HydroxyacylCoAMAT, KmcrotC14HydroxyacylCoAMAT,
   KmcrotC12HydroxyacylCoAMAT, KmcrotC8HydroxyacylCoAMAT,
   KmcrotC6HydroxyacylCoAMAT, KmcrotC4HydroxyacylCoAMAT, KicrotC4AcetoacylCoA,
   Keqcrot, C10EnoylCoAMAT[t], C16EnoylCoAMAT[t], C14EnoylCoAMAT[t],
   C12EnoylCoAMAT[t], C8EnoylCoAMAT[t], C6EnoylCoAMAT[t],
   C4EnoylCoAMAT[t], C10HydroxyacylCoAMAT[t], C16HydroxyacylCoAMAT[t],
   C14HydroxyacylCoAMAT[t], C12HydroxyacylCoAMAT[t], C8HydroxyacylCoAMAT[t],
   C6HydroxyacylCoAMAT[t], C4HydroxyacylCoAMAT[t], C4AcetoacylCoAMAT[t]],
vcrotC8 → CROT[sfcrotC8, Vcrot, KmcrotC8EnoylCoAMAT, KmcrotC16EnoylCoAMAT,
   KmcrotC14EnoylCoAMAT, KmcrotC12EnoylCoAMAT, KmcrotC10EnoylCoAMAT,
   KmcrotC6EnoylCoAMAT, KmcrotC4EnoylCoAMAT, KmcrotC8HydroxyacylCoAMAT,
   KmcrotC16HydroxyacylCoAMAT, KmcrotC14HydroxyacylCoAMAT,
   KmcrotC12HydroxyacylCoAMAT, KmcrotC10HydroxyacylCoAMAT,
   KmcrotC6HydroxyacylCoAMAT, KmcrotC4HydroxyacylCoAMAT, KicrotC4AcetoacylCoA,
   Keqcrot, C8EnoylCoAMAT[t], C16EnoylCoAMAT[t], C14EnoylCoAMAT[t],
   C12EnoylCoAMAT[t], C10EnoylCoAMAT[t], C6EnoylCoAMAT[t],
```

```
    C4EnoylCoAMAT[t], C8HydroxyacylCoAMAT[t], C16HydroxyacylCoAMAT[t],
    C14HydroxyacylCoAMAT[t], C12HydroxyacylCoAMAT[t], C10HydroxyacylCoAMAT[t],
    C6HydroxyacylCoAMAT[t], C4HydroxyacylCoAMAT[t], C4AcetoacylCoAMAT[t]],
  vcrotC6 → CROT[sfcrotC6, Vcrot, KmcrotC6EnoylCoAMAT, KmcrotC16EnoylCoAMAT,
    KmcrotC14EnoylCoAMAT, KmcrotC12EnoylCoAMAT, KmcrotC10EnoylCoAMAT,
    KmcrotC8EnoylCoAMAT, KmcrotC4EnoylCoAMAT, KmcrotC6HydroxyacylCoAMAT,
    KmcrotC16HydroxyacylCoAMAT, KmcrotC14HydroxyacylCoAMAT,
    KmcrotC12HydroxyacylCoAMAT, KmcrotC10HydroxyacylCoAMAT,
    KmcrotC8HydroxyacylCoAMAT, KmcrotC4HydroxyacylCoAMAT, KicrotC4AcetoacylCoA,
    Keqcrot, C6EnoylCoAMAT[t], C16EnoylCoAMAT[t], C14EnoylCoAMAT[t],
    C12EnoylCoAMAT[t], C10EnoylCoAMAT[t], C8EnoylCoAMAT[t],
    C4EnoylCoAMAT[t], C6HydroxyacylCoAMAT[t], C16HydroxyacylCoAMAT[t],
    C14HydroxyacylCoAMAT[t], C12HydroxyacylCoAMAT[t], C10HydroxyacylCoAMAT[t],
    C8HydroxyacylCoAMAT[t], C4HydroxyacylCoAMAT[t], C4AcetoacylCoAMAT[t]],
  vcrotC4 → CROT[sfcrotC4, Vcrot, KmcrotC4EnoylCoAMAT, KmcrotC16EnoylCoAMAT,
    KmcrotC14EnoylCoAMAT, KmcrotC12EnoylCoAMAT, KmcrotC10EnoylCoAMAT,
    KmcrotC8EnoylCoAMAT, KmcrotC6EnoylCoAMAT, KmcrotC4HydroxyacylCoAMAT,
    KmcrotC16HydroxyacylCoAMAT, KmcrotC14HydroxyacylCoAMAT,
    KmcrotC12HydroxyacylCoAMAT, KmcrotC10HydroxyacylCoAMAT,
    KmcrotC8HydroxyacylCoAMAT, KmcrotC6HydroxyacylCoAMAT, KicrotC4AcetoacylCoA,
    Keqcrot, C4EnoylCoAMAT[t], C16EnoylCoAMAT[t], C14EnoylCoAMAT[t],
    C12EnoylCoAMAT[t], C10EnoylCoAMAT[t], C8EnoylCoAMAT[t],
    C6EnoylCoAMAT[t], C4HydroxyacylCoAMAT[t], C16HydroxyacylCoAMAT[t],
    C14HydroxyacylCoAMAT[t], C12HydroxyacylCoAMAT[t], C10HydroxyacylCoAMAT[t],
    C8HydroxyacylCoAMAT[t], C6HydroxyacylCoAMAT[t], C4AcetoacylCoAMAT[t]],
  vmschadC16 → MSCHAD[sfmschadC16, Vmschad, KmmschadC16HydroxyacylCoAMAT,
    KmmschadC14HydroxyacylCoAMAT, KmmschadC12HydroxyacylCoAMAT,
    KmmschadC10HydroxyacylCoAMAT, KmmschadC8HydroxyacylCoAMAT,
    KmmschadC6HydroxyacylCoAMAT, KmmschadC4HydroxyacylCoAMAT,
    KmmschadNADMAT, KmmschadC16KetoacylCoAMAT, KmmschadC14KetoacylCoAMAT,
    KmmschadC12KetoacylCoAMAT, KmmschadC10KetoacylCoAMAT, KmmschadC8KetoacylCoAMAT,
    KmmschadC6KetoacylCoAMAT, KmmschadC4AcetoacylCoAMAT, KmmschadNADHMAT,
    Keqmschad, C16HydroxyacylCoAMAT[t], C14HydroxyacylCoAMAT[t],
    C12HydroxyacylCoAMAT[t], C10HydroxyacylCoAMAT[t], C8HydroxyacylCoAMAT[t],
    C6HydroxyacylCoAMAT[t], C4HydroxyacylCoAMAT[t], NADtMAT, C16KetoacylCoAMAT[t],
    C14KetoacylCoAMAT[t], C12KetoacylCoAMAT[t], C10KetoacylCoAMAT[t],
    C8KetoacylCoAMAT[t], C6KetoacylCoAMAT[t], C4AcetoacylCoAMAT[t], NADHMAT[t]],
  vmschadC14 → MSCHAD[sfmschadC14, Vmschad, KmmschadC14HydroxyacylCoAMAT,
    KmmschadC16HydroxyacylCoAMAT, KmmschadC12HydroxyacylCoAMAT,
    KmmschadC10HydroxyacylCoAMAT, KmmschadC8HydroxyacylCoAMAT,
    KmmschadC6HydroxyacylCoAMAT, KmmschadC4HydroxyacylCoAMAT,
    KmmschadNADMAT, KmmschadC14KetoacylCoAMAT, KmmschadC16KetoacylCoAMAT,
    KmmschadC12KetoacylCoAMAT, KmmschadC10KetoacylCoAMAT, KmmschadC8KetoacylCoAMAT,
    KmmschadC6KetoacylCoAMAT, KmmschadC4AcetoacylCoAMAT, KmmschadNADHMAT,
    Keqmschad, C14HydroxyacylCoAMAT[t], C16HydroxyacylCoAMAT[t],
    C12HydroxyacylCoAMAT[t], C10HydroxyacylCoAMAT[t], C8HydroxyacylCoAMAT[t],
    C6HydroxyacylCoAMAT[t], C4HydroxyacylCoAMAT[t], NADtMAT, C14KetoacylCoAMAT[t],
    C16KetoacylCoAMAT[t], C12KetoacylCoAMAT[t], C10KetoacylCoAMAT[t],
    C8KetoacylCoAMAT[t], C6KetoacylCoAMAT[t], C4AcetoacylCoAMAT[t], NADHMAT[t]],
  vmschadC12 → MSCHAD[sfmschadC12, Vmschad, KmmschadC12HydroxyacylCoAMAT,
    KmmschadC16HydroxyacylCoAMAT, KmmschadC14HydroxyacylCoAMAT,
    KmmschadC10HydroxyacylCoAMAT, KmmschadC8HydroxyacylCoAMAT,
    KmmschadC6HydroxyacylCoAMAT, KmmschadC4HydroxyacylCoAMAT,
    KmmschadNADMAT, KmmschadC12KetoacylCoAMAT, KmmschadC16KetoacylCoAMAT,
    KmmschadC14KetoacylCoAMAT, KmmschadC10KetoacylCoAMAT, KmmschadC8KetoacylCoAMAT,
```

```
    KmmschadC6KetoacylCoAMAT, KmmschadC4AcetoacylCoAMAT, KmmschadNADHMAT,
    Keqmschad, C12HydroxyacylCoAMAT[t], C16HydroxyacylCoAMAT[t],
    C14HydroxyacylCoAMAT[t], C10HydroxyacylCoAMAT[t], C8HydroxyacylCoAMAT[t],
    C6HydroxyacylCoAMAT[t], C4HydroxyacylCoAMAT[t], NADtMAT, C12KetoacylCoAMAT[t],
    C16KetoacylCoAMAT[t], C14KetoacylCoAMAT[t], C10KetoacylCoAMAT[t],
    C8KetoacylCoAMAT[t], C6KetoacylCoAMAT[t], C4AcetoacylCoAMAT[t], NADHMAT[t]],
vmschadC10 → MSCHAD[sfmschadC10, Vmschad, KmmschadC10HydroxyacylCoAMAT,
    KmmschadC16HydroxyacylCoAMAT, KmmschadC14HydroxyacylCoAMAT,
    KmmschadC12HydroxyacylCoAMAT, KmmschadC8HydroxyacylCoAMAT,
    KmmschadC6HydroxyacylCoAMAT, KmmschadC4HydroxyacylCoAMAT,
    KmmschadNADMAT, KmmschadC10KetoacylCoAMAT, KmmschadC16KetoacylCoAMAT,
    KmmschadC14KetoacylCoAMAT, KmmschadC12KetoacylCoAMAT, KmmschadC8KetoacylCoAMAT,
    KmmschadC6KetoacylCoAMAT, KmmschadC4AcetoacylCoAMAT, KmmschadNADHMAT,
    Keqmschad, C10HydroxyacylCoAMAT[t], C16HydroxyacylCoAMAT[t],
    C14HydroxyacylCoAMAT[t], C12HydroxyacylCoAMAT[t], C8HydroxyacylCoAMAT[t],
    C6HydroxyacylCoAMAT[t], C4HydroxyacylCoAMAT[t], NADtMAT, C10KetoacylCoAMAT[t],
    C16KetoacylCoAMAT[t], C14KetoacylCoAMAT[t], C12KetoacylCoAMAT[t],
    C8KetoacylCoAMAT[t], C6KetoacylCoAMAT[t], C4AcetoacylCoAMAT[t], NADHMAT[t]],
vmschadC8 → MSCHAD[sfmschadC8, Vmschad, KmmschadC8HydroxyacylCoAMAT,
    KmmschadC16HydroxyacylCoAMAT, KmmschadC14HydroxyacylCoAMAT,
    KmmschadC12HydroxyacylCoAMAT, KmmschadC10HydroxyacylCoAMAT,
    KmmschadC6HydroxyacylCoAMAT, KmmschadC4HydroxyacylCoAMAT,
    KmmschadNADMAT, KmmschadC8KetoacylCoAMAT, KmmschadC16KetoacylCoAMAT,
    KmmschadC14KetoacylCoAMAT, KmmschadC12KetoacylCoAMAT, KmmschadC10KetoacylCoAMAT,
    KmmschadC6KetoacylCoAMAT, KmmschadC4AcetoacylCoAMAT, KmmschadNADHMAT,
    Keqmschad, C8HydroxyacylCoAMAT[t], C16HydroxyacylCoAMAT[t],
    C14HydroxyacylCoAMAT[t], C12HydroxyacylCoAMAT[t], C10HydroxyacylCoAMAT[t],
    C6HydroxyacylCoAMAT[t], C4HydroxyacylCoAMAT[t], NADtMAT, C8KetoacylCoAMAT[t],
    C16KetoacylCoAMAT[t], C14KetoacylCoAMAT[t], C12KetoacylCoAMAT[t],
    C10KetoacylCoAMAT[t], C6KetoacylCoAMAT[t], C4AcetoacylCoAMAT[t], NADHMAT[t]],
vmschadC6 → MSCHAD[sfmschadC6, Vmschad, KmmschadC6HydroxyacylCoAMAT,
    KmmschadC16HydroxyacylCoAMAT, KmmschadC14HydroxyacylCoAMAT,
    KmmschadC12HydroxyacylCoAMAT, KmmschadC10HydroxyacylCoAMAT,
    KmmschadC8HydroxyacylCoAMAT, KmmschadC4HydroxyacylCoAMAT,
    KmmschadNADMAT, KmmschadC6KetoacylCoAMAT, KmmschadC16KetoacylCoAMAT,
    KmmschadC14KetoacylCoAMAT, KmmschadC12KetoacylCoAMAT, KmmschadC10KetoacylCoAMAT,
    KmmschadC8KetoacylCoAMAT, KmmschadC4AcetoacylCoAMAT, KmmschadNADHMAT,
    Keqmschad, C6HydroxyacylCoAMAT[t], C16HydroxyacylCoAMAT[t],
    C14HydroxyacylCoAMAT[t], C12HydroxyacylCoAMAT[t], C10HydroxyacylCoAMAT[t],
    C8HydroxyacylCoAMAT[t], C4HydroxyacylCoAMAT[t], NADtMAT, C6KetoacylCoAMAT[t],
    C16KetoacylCoAMAT[t], C14KetoacylCoAMAT[t], C12KetoacylCoAMAT[t],
    C10KetoacylCoAMAT[t], C8KetoacylCoAMAT[t], C4AcetoacylCoAMAT[t], NADHMAT[t]],
vmschadC4 → MSCHAD[sfmschadC4, Vmschad, KmmschadC4HydroxyacylCoAMAT,
    KmmschadC16HydroxyacylCoAMAT, KmmschadC14HydroxyacylCoAMAT,
    KmmschadC12HydroxyacylCoAMAT, KmmschadC10HydroxyacylCoAMAT,
    KmmschadC8HydroxyacylCoAMAT, KmmschadC6HydroxyacylCoAMAT,
    KmmschadNADMAT, KmmschadC4AcetoacylCoAMAT, KmmschadC16KetoacylCoAMAT,
    KmmschadC14KetoacylCoAMAT, KmmschadC12KetoacylCoAMAT, KmmschadC10KetoacylCoAMAT,
    KmmschadC8KetoacylCoAMAT, KmmschadC6KetoacylCoAMAT, KmmschadNADHMAT,
    Keqmschad, C4HydroxyacylCoAMAT[t], C16HydroxyacylCoAMAT[t],
    C14HydroxyacylCoAMAT[t], C12HydroxyacylCoAMAT[t], C10HydroxyacylCoAMAT[t],
    C8HydroxyacylCoAMAT[t], C6HydroxyacylCoAMAT[t], NADtMAT, C4AcetoacylCoAMAT[t],
    C16KetoacylCoAMAT[t], C14KetoacylCoAMAT[t], C12KetoacylCoAMAT[t],
    C10KetoacylCoAMAT[t], C8KetoacylCoAMAT[t], C6KetoacylCoAMAT[t], NADHMAT[t]],
vmckatC16 → MCKATA[sfmckatC16, Vmckat, KmmckatC16KetoacylCoAMAT,
```

```
    KmmckatC14KetoacylCoAMAT, KmmckatC12KetoacylCoAMAT, KmmckatC10KetoacylCoAMAT,
    KmmckatC8KetoacylCoAMAT, KmmckatC6KetoacylCoAMAT, KmmckatC4AcetoacylCoAMAT,
    KmmckatCoAMAT, KmmckatC14AcylCoAMAT, KmmckatC16AcylCoAMAT, KmmckatC12AcylCoAMAT,
    KmmckatC10AcylCoAMAT, KmmckatC8AcylCoAMAT, KmmckatC6AcylCoAMAT,
    KmmckatC4AcylCoAMAT, KmmckatAcetylCoAMAT, Keqmckat, C16KetoacylCoAMAT[t],
    C14KetoacylCoAMAT[t], C12KetoacylCoAMAT[t], C10KetoacylCoAMAT[t],
    C8KetoacylCoAMAT[t], C6KetoacylCoAMAT[t], C4AcetoacylCoAMAT[t], CoAMAT,
    C14AcylCoAMAT[t], C16AcylCoAMAT[t], C12AcylCoAMAT[t], C10AcylCoAMAT[t],
    C8AcylCoAMAT[t], C6AcylCoAMAT[t], C4AcylCoAMAT[t], AcetylCoAMAT[t]],
  vmckatC14 → MCKATA[sfmckatC14, Vmckat, KmmckatC14KetoacylCoAMAT,
    KmmckatC16KetoacylCoAMAT, KmmckatC12KetoacylCoAMAT, KmmckatC10KetoacylCoAMAT,
    KmmckatC8KetoacylCoAMAT, KmmckatC6KetoacylCoAMAT, KmmckatC4AcetoacylCoAMAT,
    KmmckatCoAMAT, KmmckatC12AcylCoAMAT, KmmckatC16AcylCoAMAT, KmmckatC14AcylCoAMAT,
    KmmckatC10AcylCoAMAT, KmmckatC8AcylCoAMAT, KmmckatC6AcylCoAMAT,
    KmmckatC4AcylCoAMAT, KmmckatAcetylCoAMAT, Keqmckat, C14KetoacylCoAMAT[t],
    C16KetoacylCoAMAT[t], C12KetoacylCoAMAT[t], C10KetoacylCoAMAT[t],
    C8KetoacylCoAMAT[t], C6KetoacylCoAMAT[t], C4AcetoacylCoAMAT[t], CoAMAT,
    C12AcylCoAMAT[t], C16AcylCoAMAT[t], C14AcylCoAMAT[t], C10AcylCoAMAT[t],
    C8AcylCoAMAT[t], C6AcylCoAMAT[t], C4AcylCoAMAT[t], AcetylCoAMAT[t]],
  vmckatC12 → MCKATA[sfmckatC12, Vmckat, KmmckatC12KetoacylCoAMAT,
    KmmckatC16KetoacylCoAMAT, KmmckatC14KetoacylCoAMAT, KmmckatC10KetoacylCoAMAT,
    KmmckatC8KetoacylCoAMAT, KmmckatC6KetoacylCoAMAT, KmmckatC4AcetoacylCoAMAT,
    KmmckatCoAMAT, KmmckatC10AcylCoAMAT, KmmckatC16AcylCoAMAT, KmmckatC14AcylCoAMAT,
    KmmckatC12AcylCoAMAT, KmmckatC8AcylCoAMAT, KmmckatC6AcylCoAMAT,
    KmmckatC4AcylCoAMAT, KmmckatAcetylCoAMAT, Keqmckat, C12KetoacylCoAMAT[t],
    C16KetoacylCoAMAT[t], C14KetoacylCoAMAT[t], C10KetoacylCoAMAT[t],
    C8KetoacylCoAMAT[t], C6KetoacylCoAMAT[t], C4AcetoacylCoAMAT[t], CoAMAT,
    C10AcylCoAMAT[t], C16AcylCoAMAT[t], C14AcylCoAMAT[t], C12AcylCoAMAT[t],
    C8AcylCoAMAT[t], C6AcylCoAMAT[t], C4AcylCoAMAT[t], AcetylCoAMAT[t]],
  vmckatC10 → MCKATA[sfmckatC10, Vmckat, KmmckatC10KetoacylCoAMAT,
    KmmckatC16KetoacylCoAMAT, KmmckatC14KetoacylCoAMAT, KmmckatC12KetoacylCoAMAT,
    KmmckatC8KetoacylCoAMAT, KmmckatC6KetoacylCoAMAT, KmmckatC4AcetoacylCoAMAT,
    KmmckatCoAMAT, KmmckatC8AcylCoAMAT, KmmckatC16AcylCoAMAT, KmmckatC14AcylCoAMAT,
    KmmckatC12AcylCoAMAT, KmmckatC10AcylCoAMAT, KmmckatC6AcylCoAMAT,
    KmmckatC4AcylCoAMAT, KmmckatAcetylCoAMAT, Keqmckat, C10KetoacylCoAMAT[t],
    C16KetoacylCoAMAT[t], C14KetoacylCoAMAT[t], C12KetoacylCoAMAT[t],
    C8KetoacylCoAMAT[t], C6KetoacylCoAMAT[t], C4AcetoacylCoAMAT[t], CoAMAT,
    C8AcylCoAMAT[t], C16AcylCoAMAT[t], C14AcylCoAMAT[t], C12AcylCoAMAT[t],
    C10AcylCoAMAT[t], C6AcylCoAMAT[t], C4AcylCoAMAT[t], AcetylCoAMAT[t]],
  vmckatC8 → MCKATA[sfmckatC8, Vmckat, KmmckatC8KetoacylCoAMAT,
    KmmckatC16KetoacylCoAMAT, KmmckatC14KetoacylCoAMAT, KmmckatC12KetoacylCoAMAT,
    KmmckatC10KetoacylCoAMAT, KmmckatC6KetoacylCoAMAT, KmmckatC4AcetoacylCoAMAT,
    KmmckatCoAMAT, KmmckatC6AcylCoAMAT, KmmckatC16AcylCoAMAT, KmmckatC14AcylCoAMAT,
    KmmckatC12AcylCoAMAT, KmmckatC10AcylCoAMAT, KmmckatC8AcylCoAMAT,
    KmmckatC4AcylCoAMAT, KmmckatAcetylCoAMAT, Keqmckat, C8KetoacylCoAMAT[t],
    C16KetoacylCoAMAT[t], C14KetoacylCoAMAT[t], C12KetoacylCoAMAT[t],
    C10KetoacylCoAMAT[t], C6KetoacylCoAMAT[t], C4AcetoacylCoAMAT[t], CoAMAT,
    C6AcylCoAMAT[t], C16AcylCoAMAT[t], C14AcylCoAMAT[t], C12AcylCoAMAT[t],
    C10AcylCoAMAT[t], C8AcylCoAMAT[t], C4AcylCoAMAT[t], AcetylCoAMAT[t]],
  vmckatC6 → MCKATA[sfmckatC6, Vmckat, KmmckatC6KetoacylCoAMAT,
    KmmckatC16KetoacylCoAMAT, KmmckatC14KetoacylCoAMAT, KmmckatC12KetoacylCoAMAT,
    KmmckatC10KetoacylCoAMAT, KmmckatC8KetoacylCoAMAT, KmmckatC4AcetoacylCoAMAT,
    KmmckatCoAMAT, KmmckatC4AcylCoAMAT, KmmckatC16AcylCoAMAT, KmmckatC14AcylCoAMAT,
    KmmckatC12AcylCoAMAT, KmmckatC10AcylCoAMAT, KmmckatC8AcylCoAMAT,
    KmmckatC6AcylCoAMAT, KmmckatAcetylCoAMAT, Keqmckat, C6KetoacylCoAMAT[t],
```

```
    C16KetoacylCoAMAT[t], C14KetoacylCoAMAT[t], C12KetoacylCoAMAT[t],
    C10KetoacylCoAMAT[t], C8KetoacylCoAMAT[t], C4AcetoacylCoAMAT[t], CoAMAT,
    C4AcylCoAMAT[t], C16AcylCoAMAT[t], C14AcylCoAMAT[t], C12AcylCoAMAT[t],
    C10AcylCoAMAT[t], C8AcylCoAMAT[t], C6AcylCoAMAT[t], AcetylCoAMAT[t]],
  vmckatC4 → MCKATB[sfmckatC4, Vmckat, KmmckatC4AcetoacylCoAMAT,
    KmmckatC16KetoacylCoAMAT, KmmckatC14KetoacylCoAMAT, KmmckatC12KetoacylCoAMAT,
    KmmckatC10KetoacylCoAMAT, KmmckatC8KetoacylCoAMAT, KmmckatC6KetoacylCoAMAT,
    KmmckatCoAMAT, KmmckatC4AcylCoAMAT, KmmckatC16AcylCoAMAT, KmmckatC14AcylCoAMAT,
    KmmckatC12AcylCoAMAT, KmmckatC10AcylCoAMAT, KmmckatC8AcylCoAMAT,
    KmmckatC6AcylCoAMAT, KmmckatAcetylCoAMAT, Keqmckat, C4AcetoacylCoAMAT[t],
    C16KetoacylCoAMAT[t], C14KetoacylCoAMAT[t], C12KetoacylCoAMAT[t],
    C10KetoacylCoAMAT[t], C8KetoacylCoAMAT[t], C6KetoacylCoAMAT[t], CoAMAT,
    C4AcylCoAMAT[t], C16AcylCoAMAT[t], C14AcylCoAMAT[t], C12AcylCoAMAT[t],
    C10AcylCoAMAT[t], C8AcylCoAMAT[t], C6AcylCoAMAT[t], AcetylCoAMAT[t]],
  vmtpC16 → MTP[sfmtpC16, Vmtp, KmmtpC16EnoylCoAMAT, KmmtpC14EnoylCoAMAT,
    KmmtpC12EnoylCoAMAT, KmmtpC10EnoylCoAMAT, KmmtpC8EnoylCoAMAT,
    KmmtpNADMAT, KmmtpCoAMAT, KmmtpC14AcylCoAMAT, KmmtpC16AcylCoAMAT,
    KmmtpC12AcylCoAMAT, KmmtpC10AcylCoAMAT, KmmtpC8AcylCoAMAT,
    KmmtpC6AcylCoAMAT, KmmtpNADHMAT, KmmtpAcetylCoAMAT, KicrotC4AcetoacylCoA,
    Keqmtp, C16EnoylCoAMAT[t], C14EnoylCoAMAT[t], C12EnoylCoAMAT[t],
    C10EnoylCoAMAT[t], C8EnoylCoAMAT[t], NADtMAT, CoAMAT, C14AcylCoAMAT[t],
    C16AcylCoAMAT[t], C12AcylCoAMAT[t], C10AcylCoAMAT[t], C8AcylCoAMAT[t],
    C6AcylCoAMAT[t], NADHMAT[t], AcetylCoAMAT[t], C4AcetoacylCoAMAT[t]],
  vmtpC14 → MTP[sfmtpC14, Vmtp, KmmtpC14EnoylCoAMAT, KmmtpC16EnoylCoAMAT,
    KmmtpC12EnoylCoAMAT, KmmtpC10EnoylCoAMAT, KmmtpC8EnoylCoAMAT,
    KmmtpNADMAT, KmmtpCoAMAT, KmmtpC12AcylCoAMAT, KmmtpC16AcylCoAMAT,
    KmmtpC14AcylCoAMAT, KmmtpC10AcylCoAMAT, KmmtpC8AcylCoAMAT,
    KmmtpC6AcylCoAMAT, KmmtpNADHMAT, KmmtpAcetylCoAMAT, KicrotC4AcetoacylCoA,
    Keqmtp, C14EnoylCoAMAT[t], C16EnoylCoAMAT[t], C12EnoylCoAMAT[t],
    C10EnoylCoAMAT[t], C8EnoylCoAMAT[t], NADtMAT, CoAMAT, C12AcylCoAMAT[t],
    C16AcylCoAMAT[t], C14AcylCoAMAT[t], C10AcylCoAMAT[t], C8AcylCoAMAT[t],
    C6AcylCoAMAT[t], NADHMAT[t], AcetylCoAMAT[t], C4AcetoacylCoAMAT[t]],
  vmtpC12 → MTP[sfmtpC12, Vmtp, KmmtpC12EnoylCoAMAT, KmmtpC16EnoylCoAMAT,
    KmmtpC14EnoylCoAMAT, KmmtpC10EnoylCoAMAT, KmmtpC8EnoylCoAMAT,
    KmmtpNADMAT, KmmtpCoAMAT, KmmtpC10AcylCoAMAT, KmmtpC16AcylCoAMAT,
    KmmtpC14AcylCoAMAT, KmmtpC12AcylCoAMAT, KmmtpC8AcylCoAMAT,
    KmmtpC6AcylCoAMAT, KmmtpNADHMAT, KmmtpAcetylCoAMAT, KicrotC4AcetoacylCoA,
    Keqmtp, C12EnoylCoAMAT[t], C16EnoylCoAMAT[t], C14EnoylCoAMAT[t],
    C10EnoylCoAMAT[t], C8EnoylCoAMAT[t], NADtMAT, CoAMAT, C10AcylCoAMAT[t],
    C16AcylCoAMAT[t], C14AcylCoAMAT[t], C12AcylCoAMAT[t], C8AcylCoAMAT[t],
    C6AcylCoAMAT[t], NADHMAT[t], AcetylCoAMAT[t], C4AcetoacylCoAMAT[t]],
  vmtpC10 → MTP[sfmtpC10, Vmtp, KmmtpC10EnoylCoAMAT, KmmtpC16EnoylCoAMAT,
    KmmtpC14EnoylCoAMAT, KmmtpC12EnoylCoAMAT, KmmtpC8EnoylCoAMAT,
    KmmtpNADMAT, KmmtpCoAMAT, KmmtpC8AcylCoAMAT, KmmtpC16AcylCoAMAT,
    KmmtpC14AcylCoAMAT, KmmtpC12AcylCoAMAT, KmmtpC10AcylCoAMAT,
    KmmtpC6AcylCoAMAT, KmmtpNADHMAT, KmmtpAcetylCoAMAT, KicrotC4AcetoacylCoA,
    Keqmtp, C10EnoylCoAMAT[t], C16EnoylCoAMAT[t], C14EnoylCoAMAT[t],
    C12EnoylCoAMAT[t], C8EnoylCoAMAT[t], NADtMAT, CoAMAT, C8AcylCoAMAT[t],
    C16AcylCoAMAT[t], C14AcylCoAMAT[t], C12AcylCoAMAT[t], C10AcylCoAMAT[t],
    C6AcylCoAMAT[t], NADHMAT[t], AcetylCoAMAT[t], C4AcetoacylCoAMAT[t]],
  vmtpC8 → MTP[sfmtpC8, Vmtp, KmmtpC8EnoylCoAMAT, KmmtpC16EnoylCoAMAT,
    KmmtpC14EnoylCoAMAT, KmmtpC12EnoylCoAMAT, KmmtpC10EnoylCoAMAT,
    KmmtpNADMAT, KmmtpCoAMAT, KmmtpC6AcylCoAMAT, KmmtpC16AcylCoAMAT,
    KmmtpC14AcylCoAMAT, KmmtpC12AcylCoAMAT, KmmtpC10AcylCoAMAT,
    KmmtpC8AcylCoAMAT, KmmtpNADHMAT, KmmtpAcetylCoAMAT, KicrotC4AcetoacylCoA,
```

```
      Keqmtp, C8EnoylCoAMAT[t], C16EnoylCoAMAT[t], C14EnoylCoAMAT[t],
      C12EnoylCoAMAT[t], C10EnoylCoAMAT[t], NADtMAT, CoAMAT, C6AcylCoAMAT[t],
      C16AcylCoAMAT[t], C14AcylCoAMAT[t], C12AcylCoAMAT[t], C10AcylCoAMAT[t],
      C8AcylCoAMAT[t], NADHMAT[t], AcetylCoAMAT[t], C4AcetoacylCoAMAT[t]],
    vacesink → RES[Ksacesink, AcetylCoAMAT[t], K1acesink],
    vfadhsink → RES[Ksfadhsink, FADHMAT[t], K1fadhsink],
    vnadhsink → RES[Ksnadhsink, NADHMAT[t], K1nadhsink]};

CoAMATX =
  {CoAMAT → CoAMATt - C16AcylCoAMAT[t] - C16EnoylCoAMAT[t] - C16HydroxyacylCoAMAT[t] -
      C16KetoacylCoAMAT[t] - C14AcylCoAMAT[t] - C14EnoylCoAMAT[t] -
      C14HydroxyacylCoAMAT[t] - C14KetoacylCoAMAT[t] - C12AcylCoAMAT[t] -
      C12EnoylCoAMAT[t] - C12HydroxyacylCoAMAT[t] - C12KetoacylCoAMAT[t] -
      C10AcylCoAMAT[t] - C10EnoylCoAMAT[t] - C10HydroxyacylCoAMAT[t] -
      C10KetoacylCoAMAT[t] - C8AcylCoAMAT[t] - C8EnoylCoAMAT[t] -
      C8HydroxyacylCoAMAT[t] - C8KetoacylCoAMAT[t] - C6AcylCoAMAT[t] - C6EnoylCoAMAT[t] -
      C6HydroxyacylCoAMAT[t] - C6KetoacylCoAMAT[t] - C4AcylCoAMAT[t] - C4EnoylCoAMAT[t] -
      C4HydroxyacylCoAMAT[t] - C4AcetoacylCoAMAT[t] - AcetylCoAMAT[t]};


Parm = {
    sfcpt1C16 → 1, Vcpt1 → 0.012, Kmcpt1C16AcylCoACYT → 13.8,
    Kmcpt1CarCYT → 250, Kmcpt1C16AcylCarCYT → 136, Kmcpt1CoACYT → 40.7,
    Kicpt1MalCoACYT → 9.1, Keqcpt1 → 0.45, ncpt1 → 2.4799,
    Vfcact → 0.42, Vrcact → 0.42, KmcactC16AcylCarCYT → 15,
    KmcactC14AcylCarCYT → 15, KmcactC12AcylCarCYT → 15, KmcactC10AcylCarCYT → 15,
    KmcactC8AcylCarCYT → 15, KmcactC6AcylCarCYT → 15, KmcactC4AcylCarCYT → 15,
    KmcactCarMAT → 130, KmcactC16AcylCarMAT → 15, KmcactC14AcylCarMAT → 15,
    KmcactC12AcylCarMAT → 15, KmcactC10AcylCarMAT → 15, KmcactC8AcylCarMAT → 15,
    KmcactC6AcylCarMAT → 15, KmcactC4AcylCarMAT → 15, KmcactCarCYT → 130,
    KicactC16AcylCarCYT → 56, KicactC14AcylCarCYT → 56, KicactC12AcylCarCYT → 56,
    KicactC10AcylCarCYT → 56, KicactC8AcylCarCYT → 56, KicactC6AcylCarCYT → 56,
    KicactC4AcylCarCYT → 56, KicactCarCYT → 200, Keqcact → 1,
    sfcpt2C16 → 0.85, sfcpt2C14 → 1, sfcpt2C12 → 0.95, sfcpt2C10 → 0.95,
    sfcpt2C8 → 0.35, sfcpt2C6 → 0.15, sfcpt2C4 → 0.01, Vcpt2 → 0.391,
    Kmcpt2C16AcylCarMAT → 51, Kmcpt2C14AcylCarMAT → 51, Kmcpt2C12AcylCarMAT → 51,
    Kmcpt2C10AcylCarMAT → 51, Kmcpt2C8AcylCarMAT → 51, Kmcpt2C6AcylCarMAT → 51,
    Kmcpt2C4AcylCarMAT → 51, Kmcpt2CoAMAT → 30, Kmcpt2C16AcylCoAMAT → 38,
    Kmcpt2C14AcylCoAMAT → 38, Kmcpt2C12AcylCoAMAT → 38,
    Kmcpt2C10AcylCoAMAT → 38, Kmcpt2C8AcylCoAMAT → 38, Kmcpt2C6AcylCoAMAT → 1000,
    Kmcpt2C4AcylCoAMAT → 1000000, Kmcpt2CarMAT → 350, Keqcpt2 → 2.22,
    sfvlcadC16 → 1, sfvlcadC14 → 0.42, sfvlcadC12 → 0.11, Vvlcad → 0.008,
    KmvlcadC16AcylCoAMAT → 6.5, KmvlcadC14AcylCoAMAT → 4, KmvlcadC12AcylCoAMAT → 2.7,
    KmvlcadFAD → 0.12, KmvlcadC16EnoylCoAMAT → 1.08, KmvlcadC14EnoylCoAMAT → 1.08,
    KmvlcadC12EnoylCoAMAT → 1.08, KmvlcadFADH → 24.2, Keqvlcad → 6,
    sflcadC16 → 0.9, sflcadC14 → 1, sflcadC12 → 0.9, sflcadC10 → 0.75, sflcadC8 → 0.4,
    Vlcad → 0.01, KmlcadC16AcylCoAMAT → 2.5, KmlcadC14AcylCoAMAT → 7.4,
    KmlcadC12AcylCoAMAT → 9, KmlcadC10AcylCoAMAT → 24.3, KmlcadC8AcylCoAMAT → 123,
    KmlcadFAD → 0.12, KmlcadC16EnoylCoAMAT → 1.08, KmlcadC14EnoylCoAMAT → 1.08,
    KmlcadC12EnoylCoAMAT → 1.08, KmlcadC10EnoylCoAMAT → 1.08,
    KmlcadC8EnoylCoAMAT → 1.08, KmlcadFADH → 24.2, Keqlcad → 6,
    sfmcadC12 → 0.38, sfmcadC10 → 0.8, sfmcadC8 → 0.87, sfmcadC6 → 1, sfmcadC4 → 0.12,
    Vmcad → 0.081, KmmcadC12AcylCoAMAT → 5.7, KmmcadC10AcylCoAMAT → 5.4,
    KmmcadC8AcylCoAMAT → 4, KmmcadC6AcylCoAMAT → 9.4, KmmcadC4AcylCoAMAT → 135,
    KmmcadFAD → 0.12, KmmcadC12EnoylCoAMAT → 1.08, KmmcadC10EnoylCoAMAT → 1.08,
```

```
    KmmcadC8EnoylCoAMAT → 1.08, KmmcadC6EnoylCoAMAT → 1.08,
    KmmcadC4EnoylCoAMAT → 1.08, KmmcadFADH → 24.2, Keqmcad → 6,
    sfscadC6 → 0.3, sfscadC4 → 1, Vscad → 0.081, KmscadC6AcylCoAMAT → 285,
    KmscadC4AcylCoAMAT → 10.7, KmscadFAD → 0.12, KmscadC6EnoylCoAMAT → 1.08,
    KmscadC4EnoylCoAMAT → 1.08, KmscadFADH → 24.2, Keqscad -> 6,
    sfcrotC16 → 0.13, sfcrotC14 → 0.2, sfcrotC12 → 0.25, sfcrotC10 → 0.33, sfcrotC8 → 0.58,
    sfcrotC6 → 0.83, sfcrotC4 → 1, Vcrot → 3.6, KmcrotC16EnoylCoAMAT → 150,
    KmcrotC14EnoylCoAMAT → 100, KmcrotC12EnoylCoAMAT → 25, KmcrotC10EnoylCoAMAT → 25,
    KmcrotC8EnoylCoAMAT → 25, KmcrotC6EnoylCoAMAT → 25, KmcrotC4EnoylCoAMAT → 40,
    KmcrotC16HydroxyacylCoAMAT → 45, KmcrotC14HydroxyacylCoAMAT → 45,
    KmcrotC12HydroxyacylCoAMAT → 45, KmcrotC10HydroxyacylCoAMAT → 45,
    KmcrotC8HydroxyacylCoAMAT → 45, KmcrotC6HydroxyacylCoAMAT → 45,
    KmcrotC4HydroxyacylCoAMAT → 45, KicrotC4AcetoacylCoA → 1.6, Keqcrot → 3.13,
    sfmschadC16 → 0.6, sfmschadC14 → 0.5, sfmschadC12 → 0.43, sfmschadC10 → 0.64,
    sfmschadC8 → 0.89, sfmschadC6 → 1, sfmschadC4 → 0.67, Vmschad → 1,
    KmmschadC16HydroxyacylCoAMAT → 1.5, KmmschadC14HydroxyacylCoAMAT → 1.8,
    KmmschadC12HydroxyacylCoAMAT → 3.7, KmmschadC10HydroxyacylCoAMAT → 8.8,
    KmmschadC8HydroxyacylCoAMAT → 16.3, KmmschadC6HydroxyacylCoAMAT → 28.6,
    KmmschadC4HydroxyacylCoAMAT → 69.9, KmmschadNADMAT → 58.5,
    KmmschadC16KetoacylCoAMAT → 1.4, KmmschadC14KetoacylCoAMAT → 1.4,
    KmmschadC12KetoacylCoAMAT → 1.6, KmmschadC10KetoacylCoAMAT → 2.3,
    KmmschadC8KetoacylCoAMAT → 4.1, KmmschadC6KetoacylCoAMAT → 5.8,
    KmmschadC4AcetoacylCoAMAT → 16.9, KmmschadNADHMAT → 5.4, Keqmschad → 2.17 * 10^-4,
    sfmckatC16 → 0, sfmckatC14 → 0.2, sfmckatC12 → 0.38, sfmckatC10 → 0.65,
    sfmckatC8 → 0.81, sfmckatC6 → 1, sfmckatC4 → 0.49, Vmckat → 0.377,
    KmmckatC16KetoacylCoAMAT → 1.1, KmmckatC14KetoacylCoAMAT → 1.2,
    KmmckatC12KetoacylCoAMAT → 1.3, KmmckatC10KetoacylCoAMAT → 2.1,
    KmmckatC8KetoacylCoAMAT → 3.2, KmmckatC6KetoacylCoAMAT → 6.7,
    KmmckatC4AcetoacylCoAMAT → 12.4, KmmckatCoAMAT → 26.6,
    KmmckatC14AcylCoAMAT → 13.83, KmmckatC16AcylCoAMAT → 13.83,
    KmmckatC12AcylCoAMAT → 13.83, KmmckatC10AcylCoAMAT → 13.83,
    KmmckatC8AcylCoAMAT → 13.83, KmmckatC6AcylCoAMAT → 13.83,
    KmmckatC4AcylCoAMAT → 13.83, KmmckatAcetylCoAMAT → 30, Keqmckat → 1051,
    sfmtpC16 → 1, sfmtpC14 → 0.9, sfmtpC12 → 0.81, sfmtpC10 → 0.73, sfmtpC8 → 0.34,
    Vmtp → 2.84, KmmtpC16EnoylCoAMAT → 25, KmmtpC14EnoylCoAMAT → 25,
    KmmtpC12EnoylCoAMAT → 25, KmmtpC10EnoylCoAMAT → 25, KmmtpC8EnoylCoAMAT → 25,
    KmmtpNADMAT → 60, KmmtpCoAMAT → 30, KmmtpC14AcylCoAMAT → 13.83,
    KmmtpC16AcylCoAMAT → 13.83, KmmtpC12AcylCoAMAT → 13.83,
    KmmtpC10AcylCoAMAT → 13.83, KmmtpC8AcylCoAMAT → 13.83, KmmtpC6AcylCoAMAT → 13.83,
    KmmtpNADHMAT → 50, KmmtpAcetylCoAMAT → 30, Keqmtp → 0.71,
    Ksacesink → 6 000 000, K1acesink → 70, Ksfadhsink → 6 000 000,
    K1fadhsink → 0.46, Ksnadhsink → 6 000 000, K1nadhsink → 12,
    C16AcylCoACYT → 25 , CarCYT → 200, CoACYT → 140, MalCoACYT → 0,
    CarMAT -> 950, FADtMAT → 0.77, NADtMAT → 250, CoAMATt → 5000,
    VCYT → 2.2 * 10^-6, VMAT → 1.8 * 10^-6};

InitialConditions = {
    C16AcylCarCYT[0] == 0, C16AcylCarMAT[0] == 0, C16AcylCoAMAT[0] == 0,
    C16EnoylCoAMAT[0] == 0, C16HydroxyacylCoAMAT[0] == 0, C16KetoacylCoAMAT[0] == 0,
    C14AcylCarCYT[0] == 0, C14AcylCarMAT[0] == 0, C14AcylCoAMAT[0] == 0,
    C14EnoylCoAMAT[0] == 0, C14HydroxyacylCoAMAT[0] == 0, C14KetoacylCoAMAT[0] == 0,
    C12AcylCarCYT[0] == 0, C12AcylCarMAT[0] == 0, C12AcylCoAMAT[0] == 0,
    C12EnoylCoAMAT[0] == 0, C12HydroxyacylCoAMAT[0] == 0, C12KetoacylCoAMAT[0] == 0,
    C10AcylCarCYT[0] == 0, C10AcylCarMAT[0] == 0, C10AcylCoAMAT[0] == 0,
    C10EnoylCoAMAT[0] == 0, C10HydroxyacylCoAMAT[0] == 0, C10KetoacylCoAMAT[0] == 0,
```

```
    C8AcylCarCYT[0] == 0, C8AcylCarMAT[0] == 0, C8AcylCoAMAT[0] == 0,
    C8EnoylCoAMAT[0] == 0, C8HydroxyacylCoAMAT[0] == 0, C8KetoacylCoAMAT[0] == 0,
    C6AcylCarCYT[0] == 0, C6AcylCarMAT[0] == 0, C6AcylCoAMAT[0] == 0,
    C6EnoylCoAMAT[0] == 0, C6HydroxyacylCoAMAT[0] == 0, C6KetoacylCoAMAT[0] == 0,
    C4AcylCarCYT[0] == 0, C4AcylCarMAT[0] == 0, C4AcylCoAMAT[0] == 0,
    C4EnoylCoAMAT[0] == 0, C4HydroxyacylCoAMAT[0] == 0, C4AcetoacylCoAMAT[0] == 0,
    AcetylCoAMAT[0] == 70, FADHMAT[0] == 0.46, NADHMAT[0] == 12};

Vars = {
    C16AcylCarCYT, C16AcylCarMAT, C16AcylCoAMAT,
    C16EnoylCoAMAT, C16HydroxyacylCoAMAT, C16KetoacylCoAMAT,
    C14AcylCarCYT, C14AcylCarMAT, C14AcylCoAMAT, C14EnoylCoAMAT,
    C14HydroxyacylCoAMAT, C14KetoacylCoAMAT,
    C12AcylCarCYT, C12AcylCarMAT, C12AcylCoAMAT, C12EnoylCoAMAT,
    C12HydroxyacylCoAMAT, C12KetoacylCoAMAT,
    C10AcylCarCYT, C10AcylCarMAT, C10AcylCoAMAT, C10EnoylCoAMAT,
    C10HydroxyacylCoAMAT, C10KetoacylCoAMAT,
    C8AcylCarCYT, C8AcylCarMAT, C8AcylCoAMAT, C8EnoylCoAMAT,
    C8HydroxyacylCoAMAT, C8KetoacylCoAMAT,
    C6AcylCarCYT, C6AcylCarMAT, C6AcylCoAMAT, C6EnoylCoAMAT,
    C6HydroxyacylCoAMAT, C6KetoacylCoAMAT,
    C4AcylCarCYT, C4AcylCarMAT, C4AcylCoAMAT, C4EnoylCoAMAT,
    C4HydroxyacylCoAMAT, C4AcetoacylCoAMAT,
    AcetylCoAMAT, FADHMAT, NADHMAT};
```

```
In[•]:= TableForm[Odes];
TableForm[RateEqs];
TableForm[Odes /. RateEqs /. CoAMATX /. Parm ];
TableForm[RateEqs /. Parm ];
TableForm[InitialConditions];
```

```
In[•]:=
tsol = NDSolve[Join[Odes /. RateEqs /. CoAMATX /. Parm , InitialConditions],
    Vars, {t, 0, 1000000000}];
```

In[ ]:=
```
Table[{Vars[[i]][t], (Vars[[i]][900000000] /. tsol)[[1]]}, {i, 1, Length[Vars]}]
```

Out[ ]=
```
{{C16AcylCarCYT[t], 0.167997}, {C16AcylCarMAT[t], 0.355963},
 {C16AcylCoAMAT[t], 0.872403}, {C16EnoylCoAMAT[t], 0.0487436},
 {C16HydroxyacylCoAMAT[t], 0.152568}, {C16KetoacylCoAMAT[t], 0.000656626},
 {C14AcylCarCYT[t], 0.0373818}, {C14AcylCarMAT[t], 0.177564},
 {C14AcylCoAMAT[t], 1.93364}, {C14EnoylCoAMAT[t], 0.0544366},
 {C14HydroxyacylCoAMAT[t], 0.154607}, {C14KetoacylCoAMAT[t], 0.000664719},
 {C12AcylCarCYT[t], 0.0510162}, {C12AcylCarMAT[t], 0.242327},
 {C12AcylCoAMAT[t], 2.63889}, {C12EnoylCoAMAT[t], 0.0621263},
 {C12HydroxyacylCoAMAT[t], 0.187943}, {C12KetoacylCoAMAT[t], 0.000805513},
 {C10AcylCarCYT[t], 0.0916998}, {C10AcylCarMAT[t], 0.435574},
 {C10AcylCoAMAT[t], 4.74332}, {C10EnoylCoAMAT[t], 0.0684024},
 {C10HydroxyacylCoAMAT[t], 0.208289}, {C10KetoacylCoAMAT[t], 0.000890113},
 {C8AcylCarCYT[t], 0.0941203}, {C8AcylCarMAT[t], 0.447072},
 {C8AcylCoAMAT[t], 4.86853}, {C8EnoylCoAMAT[t], 0.148284},
 {C8HydroxyacylCoAMAT[t], 0.458288}, {C8KetoacylCoAMAT[t], 0.0019575},
 {C6AcylCarCYT[t], 0.249827}, {C6AcylCarMAT[t], 1.18668}, {C6AcylCoAMAT[t], 12.9227},
 {C6EnoylCoAMAT[t], 11.1239}, {C6HydroxyacylCoAMAT[t], 34.6175},
 {C6KetoacylCoAMAT[t], 0.147847}, {C4AcylCarCYT[t], 0.414653},
 {C4AcylCarMAT[t], 1.9696}, {C4AcylCoAMAT[t], 21.4486}, {C4EnoylCoAMAT[t], 41.836},
 {C4HydroxyacylCoAMAT[t], 130.681}, {C4AcetoacylCoAMAT[t], 0.558265},
 {AcetylCoAMAT[t], 70.}, {FADHMAT[t], 0.46}, {NADHMAT[t], 12.}}
```

Steady state computation by varying palmitoyl-CoA

In[ ]:=
```
ParmScan[X_] := {
    sfcpt1C16 → 1, Vcpt1 → 0.012, Kmcpt1C16AcylCoACYT → 13.8,
    Kmcpt1CarCYT → 250, Kmcpt1C16AcylCarCYT → 136, Kmcpt1CoACYT → 40.7,
    Kicpt1MalCoACYT → 9.1, Keqcpt1 → 0.45, ncpt1 → 2.4799,
    Vfcact → 0.42, Vrcact → 0.42, KmcactC16AcylCarCYT → 15,
    KmcactC14AcylCarCYT → 15, KmcactC12AcylCarCYT → 15, KmcactC10AcylCarCYT → 15,
    KmcactC8AcylCarCYT → 15, KmcactC6AcylCarCYT → 15, KmcactC4AcylCarCYT → 15,
    KmcactCarMAT → 130, KmcactC16AcylCarMAT → 15, KmcactC14AcylCarMAT → 15,
    KmcactC12AcylCarMAT → 15, KmcactC10AcylCarMAT → 15, KmcactC8AcylCarMAT → 15,
    KmcactC6AcylCarMAT → 15, KmcactC4AcylCarMAT → 15, KmcactCarCYT → 130,
    KicactC16AcylCarCYT → 56, KicactC14AcylCarCYT → 56, KicactC12AcylCarCYT → 56,
    KicactC10AcylCarCYT → 56, KicactC8AcylCarCYT → 56, KicactC6AcylCarCYT → 56,
    KicactC4AcylCarCYT → 56, KicactCarCYT → 200, Keqcact → 1,
    sfcpt2C16 → 0.85, sfcpt2C14 → 1, sfcpt2C12 → 0.95, sfcpt2C10 → 0.95,
    sfcpt2C8 → 0.35, sfcpt2C6 → 0.15, sfcpt2C4 → 0.01, Vcpt2 → 0.391,
    Kmcpt2C16AcylCarMAT → 51, Kmcpt2C14AcylCarMAT → 51, Kmcpt2C12AcylCarMAT → 51,
    Kmcpt2C10AcylCarMAT → 51, Kmcpt2C8AcylCarMAT → 51, Kmcpt2C6AcylCarMAT → 51,
    Kmcpt2C4AcylCarMAT → 51, Kmcpt2CoAMAT → 30, Kmcpt2C16AcylCoAMAT → 38,
    Kmcpt2C14AcylCoAMAT → 38, Kmcpt2C12AcylCoAMAT → 38,
    Kmcpt2C10AcylCoAMAT → 38, Kmcpt2C8AcylCoAMAT → 38, Kmcpt2C6AcylCoAMAT → 1000,
    Kmcpt2C4AcylCoAMAT → 1000000, Kmcpt2CarMAT → 350, Keqcpt2 → 2.22,
    sfvlcadC16 → 1, sfvlcadC14 → 0.42, sfvlcadC12 → 0.11, Vvlcad → 0.008,
    KmvlcadC16AcylCoAMAT → 6.5, KmvlcadC14AcylCoAMAT → 4, KmvlcadC12AcylCoAMAT → 2.7,
    KmvlcadFAD → 0.12, KmvlcadC16EnoylCoAMAT → 1.08, KmvlcadC14EnoylCoAMAT → 1.08,
    KmvlcadC12EnoylCoAMAT → 1.08, KmvlcadFADH → 24.2, Keqvlcad → 6,
    sflcadC16 → 0.9, sflcadC14 → 1, sflcadC12 → 0.9, sflcadC10 → 0.75, sflcadC8 → 0.4,
    Vlcad → 0.01, KmlcadC16AcylCoAMAT → 2.5, KmlcadC14AcylCoAMAT → 7.4,
    KmlcadC12AcylCoAMAT → 9, KmlcadC10AcylCoAMAT → 24.3, KmlcadC8AcylCoAMAT → 123,
```

```
KmlcadFAD → 0.12, KmlcadC16EnoylCoAMAT → 1.08, KmlcadC14EnoylCoAMAT → 1.08,
KmlcadC12EnoylCoAMAT → 1.08, KmlcadC10EnoylCoAMAT → 1.08,
KmlcadC8EnoylCoAMAT → 1.08, KmlcadFADH → 24.2, Keqlcad → 6,
sfmcadC12 → 0.38, sfmcadC10 → 0.8, sfmcadC8 → 0.87, sfmcadC6 → 1, sfmcadC4 → 0.12,
Vmcad → 0.081, KmmcadC12AcylCoAMAT → 5.7, KmmcadC10AcylCoAMAT → 5.4,
KmmcadC8AcylCoAMAT → 4, KmmcadC6AcylCoAMAT → 9.4, KmmcadC4AcylCoAMAT → 135,
KmmcadFAD → 0.12, KmmcadC12EnoylCoAMAT → 1.08, KmmcadC10EnoylCoAMAT → 1.08,
KmmcadC8EnoylCoAMAT → 1.08, KmmcadC6EnoylCoAMAT → 1.08,
KmmcadC4EnoylCoAMAT → 1.08, KmmcadFADH → 24.2, Keqmcad → 6,
sfscadC6 → 0.3, sfscadC4 → 1, Vscad → 0.081, KmscadC6AcylCoAMAT → 285,
KmscadC4AcylCoAMAT → 10.7, KmscadFAD → 0.12, KmscadC6EnoylCoAMAT → 1.08,
KmscadC4EnoylCoAMAT → 1.08, KmscadFADH → 24.2, Keqscad -> 6,
sfcrotC16 → 0.13, sfcrotC14 → 0.2, sfcrotC12 → 0.25, sfcrotC10 → 0.33, sfcrotC8 → 0.58,
sfcrotC6 → 0.83, sfcrotC4 → 1, Vcrot → 3.6, KmcrotC16EnoylCoAMAT → 150,
KmcrotC14EnoylCoAMAT → 100, KmcrotC12EnoylCoAMAT → 25, KmcrotC10EnoylCoAMAT → 25,
KmcrotC8EnoylCoAMAT → 25, KmcrotC6EnoylCoAMAT → 25, KmcrotC4EnoylCoAMAT → 40,
KmcrotC16HydroxyacylCoAMAT → 45, KmcrotC14HydroxyacylCoAMAT → 45,
KmcrotC12HydroxyacylCoAMAT → 45, KmcrotC10HydroxyacylCoAMAT → 45,
KmcrotC8HydroxyacylCoAMAT → 45, KmcrotC6HydroxyacylCoAMAT → 45,
KmcrotC4HydroxyacylCoAMAT → 45, KicrotC4AcetoacylCoA → 1.6, Keqcrot → 3.13,
sfmschadC16 → 0.6, sfmschadC14 → 0.5, sfmschadC12 → 0.43, sfmschadC10 → 0.64,
sfmschadC8 → 0.89, sfmschadC6 → 1, sfmschadC4 → 0.67, Vmschad → 1,
KmmschadC16HydroxyacylCoAMAT → 1.5, KmmschadC14HydroxyacylCoAMAT → 1.8,
KmmschadC12HydroxyacylCoAMAT → 3.7, KmmschadC10HydroxyacylCoAMAT → 8.8,
KmmschadC8HydroxyacylCoAMAT → 16.3, KmmschadC6HydroxyacylCoAMAT → 28.6,
KmmschadC4HydroxyacylCoAMAT → 69.9, KmmschadNADMAT → 58.5,
KmmschadC16KetoacylCoAMAT → 1.4, KmmschadC14KetoacylCoAMAT → 1.4,
KmmschadC12KetoacylCoAMAT → 1.6, KmmschadC10KetoacylCoAMAT → 2.3,
KmmschadC8KetoacylCoAMAT → 4.1, KmmschadC6KetoacylCoAMAT → 5.8,
KmmschadC4AcetoacylCoAMAT → 16.9, KmmschadNADHMAT → 5.4, Keqmschad → 2.17 * 10^-4,
sfmckatC16 → 0, sfmckatC14 → 0.2, sfmckatC12 → 0.38, sfmckatC10 → 0.65,
sfmckatC8 → 0.81, sfmckatC6 → 1, sfmckatC4 → 0.49, Vmckat → 0.377,
KmmckatC16KetoacylCoAMAT → 1.1, KmmckatC14KetoacylCoAMAT → 1.2,
KmmckatC12KetoacylCoAMAT → 1.3, KmmckatC10KetoacylCoAMAT → 2.1,
KmmckatC8KetoacylCoAMAT → 3.2, KmmckatC6KetoacylCoAMAT → 6.7,
KmmckatC4AcetoacylCoAMAT → 12.4, KmmckatCoAMAT → 26.6,
KmmckatC14AcylCoAMAT → 13.83, KmmckatC16AcylCoAMAT → 13.83,
KmmckatC12AcylCoAMAT → 13.83, KmmckatC10AcylCoAMAT → 13.83,
KmmckatC8AcylCoAMAT → 13.83, KmmckatC6AcylCoAMAT → 13.83,
KmmckatC4AcylCoAMAT → 13.83, KmmckatAcetylCoAMAT → 30, Keqmckat → 1051,
sfmtpC16 → 1, sfmtpC14 → 0.9, sfmtpC12 → 0.81, sfmtpC10 → 0.73, sfmtpC8 → 0.34,
Vmtp → 2.84, KmmtpC16EnoylCoAMAT → 25, KmmtpC14EnoylCoAMAT → 25,
KmmtpC12EnoylCoAMAT → 25, KmmtpC10EnoylCoAMAT → 25, KmmtpC8EnoylCoAMAT → 25,
KmmtpNADMAT → 60, KmmtpCoAMAT → 30, KmmtpC14AcylCoAMAT → 13.83,
KmmtpC16AcylCoAMAT → 13.83, KmmtpC12AcylCoAMAT → 13.83,
KmmtpC10AcylCoAMAT → 13.83, KmmtpC8AcylCoAMAT → 13.83, KmmtpC6AcylCoAMAT → 13.83,
KmmtpNADHMAT → 50, KmmtpAcetylCoAMAT → 30, Keqmtp → 0.71,
Ksacesink → 6000000, K1acesink → 70, Ksfadhsink → 6000000,
K1fadhsink → 0.46, Ksnadhsink → 6000000, K1nadhsink → 12,
C16AcylCoACYT → X , CarCYT → 200, CoACYT → 140, MalCoACYT → 0,
CarMAT -> 950, FADtMAT → 0.77, NADtMAT → 250, CoAMATt → 5000,
VCYT → 2.2 * 10^-6, VMAT → 1.8 * 10^-6};

tsolScanWO[X_] :=
  NDSolve[Join[Odes /. RateEqs /. CoAMATX /. ParmScan[X], InitialConditions],
```

```
    Vars, {t, 0, 1000000000}];

SsScan[X_] := Module[{SSGuess},
  SSGuess := Table[{Vars[[i]][t],
     (Vars[[i]][900000000] /. tsolScanWO[X])[[1]]}, {i, 1, Length[Vars]}];
  FindRoot[Table[Odes[[i, 2]] == 0, {i, 1, Length[Odes]}] /. RateEqs /. CoAMATX /.
    ParmScan[X], SSGuess]]
```

In[ ]:=

In[ ]:=
```
ScanDownNDSWO[Xstart_, dX_, Xend_] := Monitor[Module[{SS, SSGuess},
    DataDownNDSflux = {};
    DataDownC16AcylCoAMAT = {};
    DataDownNDSconc = {};
    DataDownNDSc4coa = {};
    DataDownNDSc6coa = {};
    DataDownNDSc4c6coa = {};
    DataDownNDScintermedcoa = {};
    DataDownNDSfreecoawa = {};

    DataDownNDSvacesink = {};
    DataDownNDSvfadhsink = {};
    DataDownNDSvnadhsink = {};
    tsolStart = tsolScanWO[Xend];
    SSGuess = Table[{Vars[[i]][t],
       (Vars[[i]][900000000] /. tsolStart)[[1]]}, {i, 1, Length[Vars]}];
    SSGuess1 = SSGuess[[All, 1]];
    SSGuess2 = SSGuess[[All, 2]];
    SSGuess1int = SSGuess1 /. t → 0;
    InitialConditionsUD = Thread[SSGuess1int == SSGuess2];

    For[X = Xend, X ≥ Xstart,

     tsolScanNDS = NDSolve[Join[Odes /. RateEqs /. CoAMATX /. ParmScan[X],
        InitialConditionsUD], Vars, {t, 0, 1000000000}];
     SSGuess = Table[{Vars[[i]][t], (Vars[[i]][900000000] /. tsolScanNDS)[[1]]},
       {i, 1, Length[Vars]}];
     SSGuess1 = SSGuess[[All, 1]];
     SSGuess2 = SSGuess[[All, 2]];
     SSGuess1int = SSGuess1 /. t → 0;
     InitialConditionsUD = Thread[SSGuess1int == SSGuess2];
     SS = Thread[SSGuess1 → SSGuess2];

     c4coa = C4AcylCoAMAT[t] + C4EnoylCoAMAT[t] +
        C4HydroxyacylCoAMAT[t] + C4AcetoacylCoAMAT[t] /. SS;
     c6coa = C6AcylCoAMAT[t] + C6EnoylCoAMAT[t] + C6HydroxyacylCoAMAT[t] +
        C6KetoacylCoAMAT[t] /. SS;
     c4c6coa = c4coa + c6coa;
     intermediatecoa =
      C4AcylCoAMAT[t] + C4EnoylCoAMAT[t] + C4HydroxyacylCoAMAT[t] + C4AcetoacylCoAMAT[t] +
        C6AcylCoAMAT[t] + C6EnoylCoAMAT[t] + C6HydroxyacylCoAMAT[t] +
        C6KetoacylCoAMAT[t] + C8AcylCoAMAT[t] + C8EnoylCoAMAT[t] +
        C8HydroxyacylCoAMAT[t] + C8KetoacylCoAMAT[t] + C10AcylCoAMAT[t] +
        C10EnoylCoAMAT[t] + C10HydroxyacylCoAMAT[t] + C10KetoacylCoAMAT[t] +
        C12AcylCoAMAT[t] + C12EnoylCoAMAT[t] + C12HydroxyacylCoAMAT[t] +
        C12KetoacylCoAMAT[t] + C14AcylCoAMAT[t] + C14EnoylCoAMAT[t] +
```

```
C14HydroxyacylCoAMAT[t] + C14KetoacylCoAMAT[t] + C16AcylCoAMAT[t] +
    C16EnoylCoAMAT[t] + C16HydroxyacylCoAMAT[t] + C16KetoacylCoAMAT[t] /. SS;
freeCoa = CoAMATt - intermediatecoa - 70 /. CoAMATX /. ParmScan[X] /. SS;

DC16AcylCoAMAT = C16AcylCoAMAT[t] /. SS;
C16AcylCarnitineCYT = C16AcylCarCYT[t] /. SS;
C14AcylCarnitineCYT = C14AcylCarCYT[t] /. SS;
C12AcylCarnitineCYT = C12AcylCarCYT[t] /. SS;
C10AcylCarnitineCYT = C10AcylCarCYT[t] /. SS;
C8AcylCarnitineCYT = C8AcylCarCYT[t] /. SS;
C6AcylCarnitineCYT = C6AcylCarCYT[t] /. SS;
C4AcylCarnitineCYT = C4AcylCarCYT[t] /. SS;
AppendTo[DataDownC16AcylCoAMAT, {X, DC16AcylCoAMAT}];
AppendTo[DataDownNDSflux,
  {X, 10^3 vcactC16 /. RateEqs /. CoAMATX /. ParmScan[X] /. SS}];
AppendTo[DataDownNDSc4coa, {X, c4coa}];
AppendTo[DataDownNDSc6coa, {X, c6coa}];
AppendTo[DataDownNDSc4c6coa, {X, c4c6coa}];
AppendTo[DataDownNDScintermedcoa, {X, intermediatecoa}];
AppendTo[DataDownNDSfreecoawa, {X, freeCoa}];

AppendTo[DataDownNDSvacesink,
  {X, 10^3 vacesink /. RateEqs /. CoAMATX /. ParmScan[X] /. SS}];
AppendTo[DataDownNDSvfadhsink,
  {X, 10^3 vfadhsink /. RateEqs /. CoAMATX /. ParmScan[X] /. SS}];
AppendTo[DataDownNDSvnadhsink,
  {X, 10^3 vnadhsink /. RateEqs /. CoAMATX /. ParmScan[X] /. SS}];
X = X - dX;]
], ProgressIndicator[X, {Xstart, Xend}]]
```

In[•]:=
```
ScanDownNDSWO[0, 1, 250]
```

In[•]:=
```
ScanUpNDSWO[Xstart_, dX_, Xend_] := Monitor[Module[{SS, SSGuess},
   DataUpNDSflux = {};
   DataUpC16AcylCoAMAT == {};
   DataUpNDSconc = {};
   DataUpNDSc4coa = {};
   DataUpNDSc6coa = {};
   DataUpNDSc4c6coa = {};
   DataUpNDScintermedcoa = {};
   DataUpNDSfreecoawa = {};

   DataUpNDSvacesink = {};
   DataUpNDSvfadhsink = {};
   DataUpNDSvnadhsink = {};

   tsolStart = tsolScanWO[Xstart];
   SSGuess = Table[{Vars[[i]][t],
      (Vars[[i]][900000000] /. tsolStart)[[1]]}, {i, 1, Length[Vars]}];
   SSGuess1 = SSGuess[[All, 1]];
```

```
SSGuess2 = SSGuess[[All, 2]];
SSGuess1int = SSGuess1 /. t → 0;
InitialConditionsUD = Thread[SSGuess1int == SSGuess2];

For[X = Xstart, X ≤ Xend,

 tsolScanNDS = NDSolve[Join[Odes /. RateEqs /. CoAMATX /. ParmScan[X],
    InitialConditionsUD], Vars, {t, 0, 1000000000}];
 SSGuess = Table[{Vars[[i]][t], (Vars[[i]][900000000] /. tsolScanNDS)[[1]]},
   {i, 1, Length[Vars]}];
 SSGuess1 = SSGuess[[All, 1]];
 SSGuess2 = SSGuess[[All, 2]];
 SSGuess1int = SSGuess1 /. t → 0;
 InitialConditionsUD = Thread[SSGuess1int == SSGuess2];
 SS = Thread[SSGuess1 → SSGuess2];

 c4coa = C4AcylCoAMAT[t] + C4EnoylCoAMAT[t] +
    C4HydroxyacylCoAMAT[t] + C4AcetoacylCoAMAT[t] /. SS;
 c6coa = C6AcylCoAMAT[t] + C6EnoylCoAMAT[t] + C6HydroxyacylCoAMAT[t] +
    C6KetoacylCoAMAT[t] /. SS;
 c4c6coa = c4coa + c6coa;
 intermediatecoa =
  C4AcylCoAMAT[t] + C4EnoylCoAMAT[t] + C4HydroxyacylCoAMAT[t] + C4AcetoacylCoAMAT[t] +
    C6AcylCoAMAT[t] + C6EnoylCoAMAT[t] + C6HydroxyacylCoAMAT[t] +
    C6KetoacylCoAMAT[t] + C8AcylCoAMAT[t] + C8EnoylCoAMAT[t] +
    C8HydroxyacylCoAMAT[t] + C8KetoacylCoAMAT[t] + C10AcylCoAMAT[t] +
    C10EnoylCoAMAT[t] + C10HydroxyacylCoAMAT[t] + C10KetoacylCoAMAT[t] +
    C12AcylCoAMAT[t] + C12EnoylCoAMAT[t] + C12HydroxyacylCoAMAT[t] +
    C12KetoacylCoAMAT[t] + C14AcylCoAMAT[t] + C14EnoylCoAMAT[t] +
    C14HydroxyacylCoAMAT[t] + C14KetoacylCoAMAT[t] + C16AcylCoAMAT[t] +
    C16EnoylCoAMAT[t] + C16HydroxyacylCoAMAT[t] + C16KetoacylCoAMAT[t] /. SS;
 freeCoa = CoAMATt - intermediatecoa - 70 /. CoAMATX /. ParmScan[X] /. SS;


 DC16AcylCoAMAT = C16AcylCoAMAT[t] /. SS;
 C16AcylCarnitineCYT = C16AcylCarCYT[t] /. SS;
 C14AcylCarnitineCYT = C14AcylCarCYT[t] /. SS;
 C12AcylCarnitineCYT = C12AcylCarCYT[t] /. SS;
 C10AcylCarnitineCYT = C10AcylCarCYT[t] /. SS;
 C8AcylCarnitineCYT = C8AcylCarCYT[t] /. SS;
 C6AcylCarnitineCYT = C6AcylCarCYT[t] /. SS;
 C4AcylCarnitineCYT = C4AcylCarCYT[t] /. SS;
 AppendTo[DataUpC16AcylCoAMAT, {X, DC16AcylCoAMAT}];

 AppendTo[DataUpNDSflux,
  {X, 10^3 vcactC16 /. RateEqs /. CoAMATX /. ParmScan[X] /. SS}];
 AppendTo[DataUpNDSc4coa, {X, c4coa}];
 AppendTo[DataUpNDSc6coa, {X, c6coa}];
 AppendTo[DataUpNDSc4c6coa, {X, c4c6coa}];
 AppendTo[DataUpNDScintermedcoa, {X, intermediatecoa}];
 AppendTo[DataUpNDSfreecoawa, {X, freeCoa}];

 AppendTo[DataUpNDSvacesink,
  {X, 10^3 vacesink /. RateEqs /. CoAMATX /. ParmScan[X] /. SS}];
```

```
    AppendTo[DataUpNDSvfadhsink,
     {X, 10^3 vfadhsink /. RateEqs /. CoAMATX /. ParmScan[X] /. SS}];
    AppendTo[DataUpNDSvnadhsink,
     {X, 10^3 vnadhsink /. RateEqs /. CoAMATX /. ParmScan[X] /. SS}];
    X = X + dX;]
  ], ProgressIndicator[X, {Xstart, Xend}]]
```

In[•]:=
```
ScanUpNDSWO[0, 1, 250]
```

AppendTo: DataUpC16AcylCoAMAT is not a variable with a value, so its value cannot be changed.

AppendTo: DataUpC16AcylCoAMAT is not a variable with a value, so its value cannot be changed.

AppendTo: DataUpC16AcylCoAMAT is not a variable with a value, so its value cannot be changed.

General: Further output of AppendTo::rvalue will be suppressed during this calculation.

Steady state plots for varying palmitoyl-CoA with and without ACOT

In[•]:=
```
ListLinePlot[{DataUpNDSfreecoawa, DataUpNDSfreecoa},
 PlotRange → All, PlotStyle → {Red , Blue}, AxesStyle → Directive[Black, 16],
 LabelStyle → Directive[Black], Frame → {{True, False}, {True, False}},
 FrameLabel → {{"Free CoA(μM)", None}, {"Palmitoyl-CoA(μM)", "Palmitoyl-CoA(μM)"}},
 PlotLegends → Placed[LineLegend[{"Without ACOT", "With ACOT"},
    LabelStyle → {FontSize → 16}], {Left, Bottom}], PlotLabel → "Free CoA",
 BaseStyle → {FontSize → 18, FontWeight → "3", AbsoluteThickness[2]},
 FrameStyle → Thickness[0.00005], ImageSize → Scaled[0.3], AspectRatio → 0.75]
```

Out[•]=
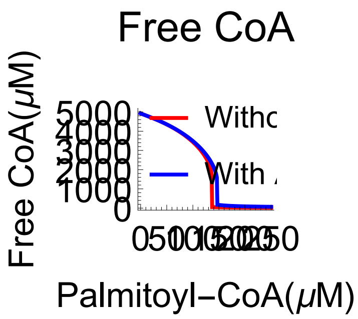

In[•]:=

```
PlotLegends → Placed[LineLegend[{"WT", "ShcKO"},
   LegendMarkerSize → {{30, 10}}, LabelStyle → {FontSize → 18}], {Right, Bottom}]
```

In[•]:=
```
ListLinePlot[{DataUpNDSvnadhsink, DataUpNDSvnadhsinkACOT, DataDownNDSvnadhsink,
  DataDownNDSvnadhsinkACOT}, PlotRange → All, PlotStyle → {Red , Blue, Red, Blue},
 AxesStyle → Directive[Black, 16], LabelStyle → Directive[Black],
 Frame → {{True, False}, {True, False}}, FrameLabel → {{"NADH Production Flux", None},
   {"Palmitoyl-CoA(μM)", "Palmitoyl-CoA(μM)"}}, PlotLegends →
  Placed[LineLegend[{"NADH production without ACOT", "NADH production with ACOT"},
    LabelStyle → {FontSize → 16}], {Right, Bottom}], PlotLabel → "ACOT",
 BaseStyle → {FontSize → 18,  FontWeight → "3", AbsoluteThickness[2]},
 FrameStyle → Thickness[0.00005], ImageSize → Scaled[0.3], AspectRatio → 0.75]
```

Out[•]=

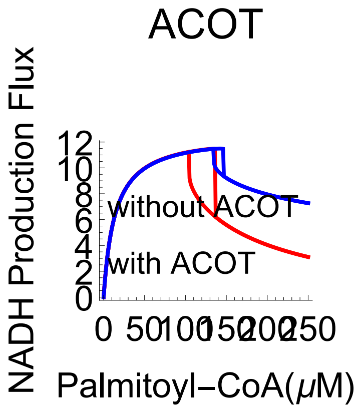

In[•]:=
```
ListLinePlot[{DataUpNDSflux, DataDownNDSflux, DataUpNDSfluxacot, DataDownNDSfluxacot},
 PlotRange → All, PlotStyle → {Red , Red, Blue, Blue}, AxesStyle → Directive[Black, 14],
 LabelStyle → Directive[14, Black, Bold], Frame → {{True, False}, {True, False}},
 FrameLabel → {{"Uptake Flux", None}, {"Palmitoyl-CoA(μM)", "Palmitoyl-CoA(μM)"}},
 PlotLegends → {"Uptake flux without ACOT", "Uptake flux without ACOT",
   "Uptake flux with ACOT", "Uptake flux with ACOT"}, PlotLabel → "ACOT"]
```

Out[•]=

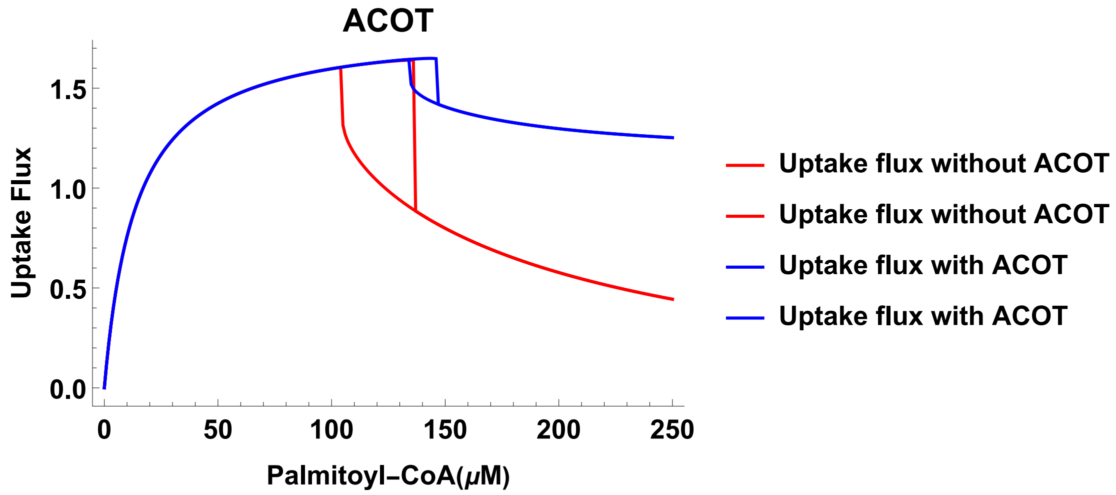

Ratio of NADH production flux to CPT1 or Uptake flux without and with ACOT

In[•]:=
```
NADHtoCPT1upwithoutACOT = DataUpNDSvnadhsink[[All, 2]] / DataUpNDSflux[[All, 2]];
```

In[•]:=
```
NADHtoCPT1downwithoutACOT = DataDownNDSvnadhsink[[All, 2]] / DataDownNDSflux[[All, 2]];
```

In[ ]:=
```
NADHtoCPT1upwithACOT = DataUpNDSvnadhsinkACOT[[All, 2]] / DataUpNDSfluxacot[[All, 2]];

NADHtoCPT1DownwithACOT =
  DataDownNDSvnadhsinkACOT[[All, 2]] / DataDownNDSfluxacot[[All, 2]];
```

In[ ]:=
```
(*ListLinePlot[{Thread[{DataUpNDSflux[[All,1]],NADHtoCPT1upwithoutACOT}],
  Thread[{DataDownNDSflux[[All,1]],NADHtoCPT1downwithoutACOT}],
  Thread[{DataUpNDSflux[[All,1]],NADHtoCPT1upwithACOT}],
  Thread[{DataDownNDSflux[[All,1]],NADHtoCPT1DownwithACOT}]},PlotRange→All,
 PlotStyle→{Red ,Red,Blue,Blue}, AxesStyle→Directive[Black,14],
 LabelStyle→Directive[14,Black,Bold],Frame→{{True,False},{True,False}},
 FrameLabel→{{"NADH production flux/Uptake Flux",None},
   {"Palmitoyl-CoA(μM)","Palmitoyl-CoA(μM)"}},
 PlotLegends→{"NADH production flux/Uptake Flux without ACOT",
   "NADH production flux/Uptake Flux without ACOT",
   "NADH production flux/Uptake Flux with ACOT",
   "NADH production flux/Uptake Flux with ACOT"},PlotLabel→"ACOT"]*)
```

In[ ]:=
```
ListLinePlot[{Thread[{DataUpNDSflux[[All, 1]], NADHtoCPT1upwithoutACOT}],
  Thread[{DataUpNDSflux[[All, 1]], NADHtoCPT1upwithACOT}]},
 PlotRange → All, PlotStyle → {Red , Blue}, AxesStyle → Directive[Black, 14],
 LabelStyle → Directive[14, Black, Bold], Frame → {{True, False}, {True, False}},
 FrameLabel → {{"NADH production flux/Uptake Flux", None},
   {"Palmitoyl-CoA(μM)", "Palmitoyl-CoA(μM)"}},
 PlotLegends → {"Without ACOT", "With ACOT"},
 PlotLabel → "Ratio of NADH production flux to uptake flux"]
```

Out[ ]=

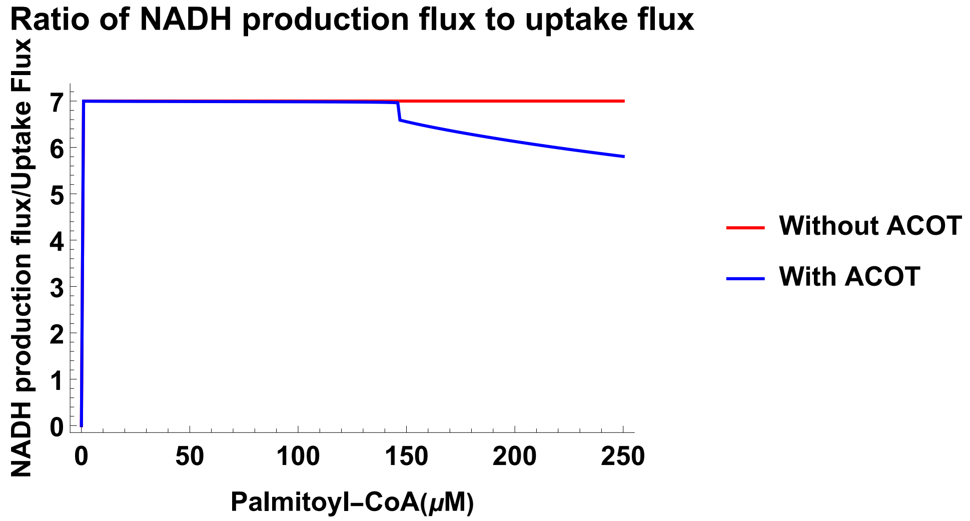

In[∘]:=
```
ListLinePlot[{Thread[{DataDownNDSflux[[All, 1]], NADHtoCPT1downwithoutACOT}],
  Thread[{DataDownNDSflux[[All, 1]], NADHtoCPT1DownwithACOT}]},
 PlotRange → All, PlotStyle → {Red , Blue}, AxesStyle → Directive[Black, 14],
 LabelStyle → Directive[14, Black, Bold], Frame → {{True, False}, {True, False}},
 FrameLabel → {{"NADH production flux/Uptake Flux", None},
   {"Palmitoyl-CoA(μM)", "Palmitoyl-CoA(μM)"}},
 PlotLegends → {"Without ACOT", "With ACOT"},
 PlotLabel → "Ratio of NADH production flux to uptake flux"]
```

Out[∘]=

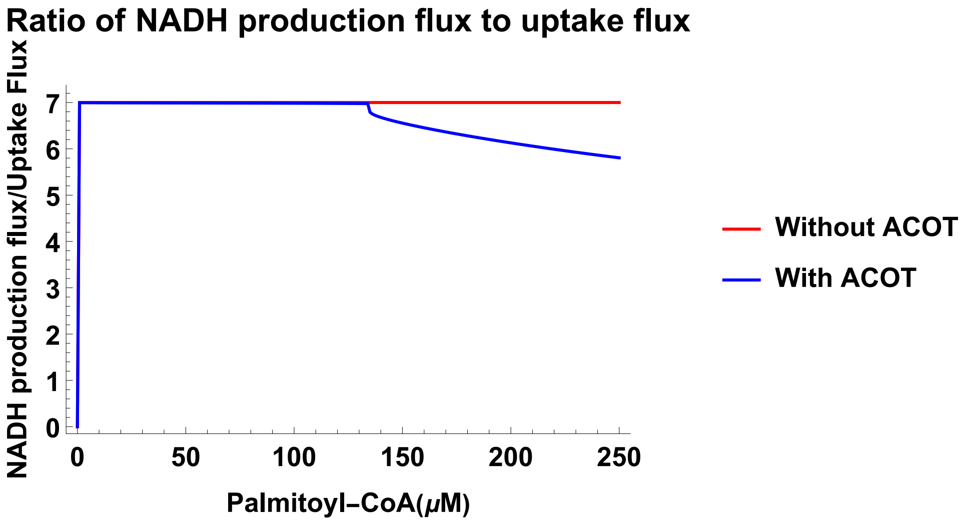

Supplement: S5 Appendix — (ZIP) [file pcbi.1009259.s016.zip › MFAOModelwithACOTExtension.pdf]
