# Supplementary material for: Bistability in fatty-acid oxidation resulting from substrate inhibition
Source: PLoS Comput Biol. 2021 Aug 12;17(8):e1009259. doi: 10.1371/journal.pcbi.1009259 (PMC8396765; doi:10.1371/journal.pcbi.1009259)
Supplement: Attachment — Submitted filename: revision letter Abegaz PLoS Comp Biol-20210616.docx [file pcbi.1009259.s017.docx]

To:	Dr. Anders Wallqvist, Associate Editor of PLOS Computational Biology

From:	Prof. dr. Barbara M. Bakker

Concerning:	Manuscript revision

Groningen, 16/06/2021

Dear Dr. Wallqvist,

Herewith we wish to submit the revised version of our manuscript entitled **‘Bistability in Fatty-Acid Oxidation resulting from Substrate Inhibition’** for publication in *PLOS Computational Biology.*

We were pleased with the positive, constructive and detailed comments of the reviewers. We address then in detail below and revised the manuscript accordingly.

We sincerely hope that you will accept this revised version for publication.

Yours sincerely,

Prof. Barbara M. Bakker

Prof. dr. Barbara M. Bakker, Professor of Medical Systems Biology
Department of Pediatrics, University of Groningen, University Medical Center Groningen
Mail address : Postbus 196, 9700 AD Groningen, The Netherlands (Internal ZIP Code EA12)
+31 50 361 1542, [B.M.Bakker01@umcg.nl](mailto:B.M.Bakker01@umcg.nl)

**Detailed response to the reviewers’ comments**

Below we respond to all the comments (answers in blue). Changes in the manuscript are also highlighted in blue. Only minor textual changes were not highlighted in the manuscript, but we did include them.

**Reviewer #1**

In this article, Abegaz et al. perform bi-stability analysis of a mathematical model describing beta-oxidation of fatty acids in rat-liver mitochondria. The article lays out the rationale for performing the analysis and then investigates the effect of specific parameters, such NAD/NADH ratio, on the observed bi-stable region of the model. The authors have assumed a fixed NAD/NADH ratio to avoid its effects on their model calculations. Specifically, the authors have chosen the NAD/NADH ratio of 20, which is twice the ratio reported by Williamson et al. (PMID: 4291787) and Henley et al. (PMID: 4312780) in rat-liver mitochondria. This is a major drawback of their model observations because the bi-stability phenomenon would not exist if they have chosen the ratio reported in Williamson et al. or Henley et al. Moreover, the authors found that hysteresis was observed with increasing concentrations of Palmitoyl-CoA; full oxidation of Palmitoyl-CoA produces 7 molecules of NADH and acetyl-CoA entering the TCA cycle generates 3 NADH molecules. Therefore, I suggest that the authors discuss the consequences of having a fixed NAD/NADH ratio on their modeling predictions and how their model results may be affected with a dynamic NAD/NADH ratio.

We agree with the reviewer that the bistability depends on the concentrations of boundary metabolites. To adress the first point, the ratio that we used has been found in the literature, albeit in isolated hepatocytes. So indeed, our ratio is higher than what has been found in vivo. We like to clarify, however, that the behaviour depends on the interaction between many parameters, which may all vary between conditions and are measured with a certain error. Therefore, we believe that the strength of the model is rather to point out qualitatively that the pathway is prone to bistability and which parameters have the strongest impact. With respect to the second point, indeed the first part of the paper deals with a fixed [NAD^+^]/[NADH] ratio. In the extended model in which the effect of short chain fatty acids (SCFA) was analysed, [NAD^+^]/[NADH] was modelled as a variable, which depended on reduction by the fatty-acid oxidation pathway and oxidation by the respiratory chain. In the control situation (no SCFA) bistability was still observed. We clarified both points in the relevant sections of the Results and Discussion, with additional references.

In addition, I have a few minor comments:

1. Line 224-226: “This can be seen easily from Figure 4B: outside the stable points the system will move towards a higher C4-ketoacyl-CoA if the production by C4-MSCHAD is faster than its consumption by C4-MCKAT, and vice versa.” Outside of the stable point ‘S3’ C4-ketoacyl-CoA seems to be decreasing in Figure 4B -- it’s not clear what the authors are saying.

We clarified this in the manuscript. In essense: outside the stable points the rate of production of C4-ketoacyl-CoA (green line, vMSCHADC4) differs from its rate of consumption (blue line, vMCKATC4). If the production is faster than the consumption, the concentration of C4-ketoacyl-CoA will increase, and vice versa. Thus, it can be inferred from the figure that the system tends to move towards steady states S1 or S3 and away from S2.

2. Line 231, “S0 in Figure 3C.” I think, the authors mean “Figure 4C.”

3. Line 325, “the rate v of enzyme i.” I think, the authors mean “the rate vi of enzyme i.”

4. Line 344, “[NAD+]:[NAD]” should be replaced by “[NAD+]:[NADH].”

We thank the reviewer for pointing this out and made all the corrections.

**Reviewer #2**

This is a well-written paper about a computational model for the mitochondrial fatty acid oxidation (FAO) pathway. The work extends upon a previously published model (Ref 40) that identified medium chain 3-keto-acyl-CoA thiolase as a key enzyme in the pathway with regards to limiting flux. The current paper examines that effect in further detail and explores some interesting ways to increase flux through the pathway, which would be physiologically beneficial and clinically relevant if possible.

This reviewer is an expert in FAO physiology, and not computational biology. The following criticisms are raised from the perspective of relating the model back to physiology.

Major Criticisms

1. The model does not include a component related to carnitine availability or the role of the reverse carnitine cycle in maintaining intra-mitochondrial levels of CoA. The enzyme CrAT can convert short to medium-length acyl-CoAs (up to 8 carbons) into acylcarnitines and free CoA. The acylcarnitines are released. Similarly, CPT2 can operate in reverse if longer-chain acyl-CoAs accumulate. This should be discussed and incorporated into the model if possible.

We apologize for our lack of clarity. In this model the CPT2 is reversible and accepts acyl-CoAs and acyl-carnitines of chain lengths C4 – C16. Also the CACT transporter reversibly transports C4-C16 acyl-carnitines. Only acetyl-CoA, an important CrAT substrate, is not converted to actyl-carnitine in this model version, since the acetyl-CoA is at this stage a fixed boundary metabolite at low concentration. For simplicity, we have not made a distinction between CPT2 and CrAT. We made this explicit in Figure 1 and in the Methods section.

2. The section on modeling of malonyl-CoA effects (beginning line 295) ends with the statement "Due to the stretching of the high-flux branch, malonyl-CoA can therefore activate the mFAO flux even though it acts through inhibition of one of its enzymes." The physiological relevance of the malonyl-CoA modeling is unclear. Under what circumstance would you have both high malonyl-coa and high levels of long-chain fatty acids? It is doubtful that malonyl-CoA would ever activate mFAO flux in vivo.

Yes, we agree with the reviewer and assessed this more carefully. It is possible that this is merely a theoretical exercise, showing us that any effector that attenuates CPT1 activity in a fatty-acid overloaded situation may prevent the low-flux state. We wonder, however, if an overloaded situation with both fatty acids and fructose, for instance, could not give rise to a high malonyl-CoA concentration in the liver (cf. Softic et al. PMID: 31577934, who found high malonyl-CoA concentration on a high-fat diet with fructose). Moreover, we calculated that the concentration range in which we varied malonyl-CoA (0-10 µM) is in the lower range of what is measured experimentally (2-60 µM, see our response to reviewer 3 for the conversion factors used). We added these considerations to the Discussion.

3. In the section on acyl-CoA thioesterases as a safety valve (beginning line 378), is the presence of ACOT2 already accounted for in the computational model? The model is based on liver mitocondria, which possess the thiolase ACOT2 as described in Ref 64. Is the model adding addtional thiolase activity? This was unclear to me.

Indeed, the original model had zero thioesterase activity. We clarified this in the corresponding Results section (*Acyl-CoA thioesterases as a safety valve*).

Further, it is stated "However, excessive accumulation of intracellular acyl-CoAs can be toxic in itself and also limit the mFAO flux due to CoA depletion." But free fatty acids are also toxic, which should be discussed. Further, liver possesses abundant medium-chain acyl-CoA synthases inside the matrix. In the model, would adding a medium-chain thiolase merely a create a vicious cycle of cleaving medium-chain acyl-CoAs and re-esterifying them to CoA at the expense of matrix ATP?

This is an interesting point. Indeed thioesterase activity is energetically costly, since ATP was invested in the synthesis of the CoA ester. Interestingly, however, the increase in beta-oxidation flux would generate much more ATP than would be lost by the moderate flux through ACOT. We highlighted this more clearly in the corresponding section in the Results. With respect to the toxicity of the fatty acids, we fully agree. However, not to disrupt the storyline, we removed the statement about toxicity entirely, and focused on the CoA availability.

**Reviewer #3**

General

The presented manuscript “Bistability in Fatty-Acid Oxidation resulting from Substrate Inhibition” provides by kinetic modeling and mathematical analysis the prediction of bistability in fatty-acid oxidation, which is characterized by the coexistence of a stable low and high-flux state of fatty-acid oxidation. The mathematical analysis is original, the manuscript well written and is of substantial interest for theoretical as well as experimental research devoted to fatty-acid metabolism. Despite that, I advise to strengthen the prediction of bistability by a comparison to experimental data and observations as well as suggestions how to perform an experimental validation. In addition, I suggest below some major and minor remarks and suggestions to improve this manuscript.

Major

• Link or relevance of bistability under physiological conditions. I am missing throughout the text the effort to convince the reader that the bistability is present under physiological conditions. Perhaps, gene expression data or any other experimental observations are only explainable by the coexistence of a high and low flux state? Further, how do you suggest to experimentally verify your predictions and which systems could be used (human studies, animal models, tissue cultures, cell cultures)?

We agree with the reviewer that the manuscript is purely theoretical and a clear relation to physiology is important. We elaborated on this in the Discussion. As we already wrote in the original version of the manuscript, in studies with ‘responders’ and ‘non-responders’ to a diet, the non-responders are often discarded. To get more evidence for bistability, it is important that heterogeneity in animal studies is embraced. In the current version we added another example of a threshold phenomen related to fatty acid oxidation, namely the sudden and severe metabolic decompensation in patients with an inherited fatty acid oxidation disorder. Also here, the dynamics have been insufficiently studied to claim bistability, but it might play a role.

• Enzyme concentration is fixed during simulation (Vmax = kcat*E). Cell could alter “E” by transcription etc., how would this change bistability? Is it possible to alter enzyme abundance to prevent the low flux state or make a transition from low to high flux state?

Yes, indeed, each simulation is performed at a fixed *V_max_*, but its value can be changed to mimic the effect of gene expression. To illustrate that this indeed alters the bistability behaviour, we varied the *V_max_* of CPT1. The results were added to the section on regulation of CPT1 by malonyl-CoA.

• Analytic analysis

o Not ideal to start with a simplification before any result of the full model is presented

o I respect the effort to provide an analytic solution. However, the result “can in principle exhibit bistability” is rather weak in comparison to the extensive derivation shown in the supplement

o Suggestion: start with a general results and features of the model (see below) and provide then the analytic solution of the simplification

We followed these suggestions and swapped the first results of the full model with that of the simplified model.

• Possible additional analyses:

o dynamic of system at non steady state (oscillation? Time until steady state is reached? …)

o Overall reaction, elementary mode, energy flux/yield of metabolic system (with and without changes in this manuscript)

We added a time simulation in Figure 2C to show how the pathway behaviour depends on the initial conditions close to the unstable steady state. Flux ratios are relevant in the context of the addition of ACOT, as this introduces an extra degree of freedom, hence we added more information about the flux ratios in that section.

Minor

• Provide Mathematica Notebook files (.nb) also as PDF to make it easily accessible for readers with no Mathematica license

We added a pdf of the Mathematical Notebook files to make it accessible to non-Mathematica users.

• Line 60-61: Could you elaborate further, how FAS prevents substrate inhibition? Isn`t the retainment of intermediates a form or sign of substrate inhibition?

Probably, this is a matter of accurate phrasing. Indeed, the intermediate that is bound prevents a new substrate to enter the active site. The retainment of the intermediates prevents, however, inhibition of the reaction by a substrate that is *upstream* in the pathway. We clarified the sentence.

• Line 72: Is the strategy to artificially increase mFAO and energy turn over in general a therapeutic option without draw back? I could imagine the risk of cell damage and DNA mutagenesis by increased ROS production etc., please consider and discuss possible side effects.

We agree, any viable strategy should consider downstream pathways as well, to avoid accumulation of metabolites. This may explain why SCFA are so beneficial, as they have pleiotropic effects. We clarified the sentence.

• Line 74-81: I think some references could be added here to back the statements.

This is largely textbook information for which we added references to two comprehensive reviews (different references than before). For the statement about competition we added a separate reference.

• Line 145-146: the main derivation is found in the supplement; please add this as additional reference.

Done

• Line 159-160: It is not clear how the and when the palmitoyl-CoA concentration is changed. Is the concentration changed over time, simulation started with different initial conditions, …?

We clarified this in the text. When the palmitoyl-CoA concentration was stepwise increased or decreased, the steady-state of the previous palmitoyl-CoA concentration was taken as the initial condition for the next simulation.

• Line 234: “bistability originates from …” I think the statement that one could define the origin of bistability to one feature is too strong. The analysis shows that is a necessary condition as well as other parameters or parameter combination. I would advise to rephrase this and be aware in the discussion of results that there is no single origin of bistability in such models, but rather a combination of topology and kinetics.

We rephrased this more accurately (‘plays a pivotal role’). .

• Line 259-275: I understand that the NAD/NADH ratio is important. But, is the overall concentration of NAD+NADH as well an important factor for bistability? And while varying NAD/NADH ratio, was the sum NAD+NADH kept constant? Please elaborate.

Yes, indeed, the sum NAD^+^ + NADH was kept constant when the ratio was varied. We clarified this in the legend to Figure 5A. We added a simulation in which the sum was varied at the end of the same section.

• Line 300-301: “The maximum flux was the same” So, it is a competitive inhibitor?

Yes, it is competitive, we clarified this.

• Line 295-307: What are physiological expected concentrations of malonyl-CoA? The higher stretch of low and high-flux state would be physiologically unfavorable, or not? Could you elaborate on this?

This is a very important question, but not easy to answer. In the model the concentrations are expressed in the same units as *K_m_* and *K_i_* values, which are in µmol/L. The measured malonyl-CoA levels reported in the literature are typically expressed in nmoles per gram of tissue or as a concentration in a processed, often diluted sample. The latter are sufficient to assess differences between study groups, but do not give us true intracellular concentration in µmol/L. Pragmatically, we simulated malonyl-CoA concentrations in the range of the inhibition constant *K_i_* of CPT1 for malonyl-CoA (9.1 µM), such that there is an effect on the enzyme. Based on conversion factors of 112 mgProtein/gram rat liver (PMID: 16930941) and 2.2 x 10^-6^ L/mg protein for the cytosol (PMID: 23966849), and reported malonyl-CoA levels in the range of 0.4-15 nmol/gram liver (doi.org/10.1152/ajpendo.1994.267.1.E95; PMID: 29790582), this would be in the lower range of the in physiological concentration (~ 2-60 µM). This would be compatible with a fasted state, in which fatty-acids are typically high. We clarified this in the in the Discussion.

• Line 325: Which parameter p have been used to calculate the control coefficients shown in Figure 6? If all, was an average calculated?

The *V_max_* was varied. This was clarified in the corresponding section of the Results.

• P46Shc and Shc: Could you elaborate further on other functions of p46Shc and Shc in general (human, mice, mammals)? Are there any harmful side effects if this is depleted? It sounds too good to be true if a knock out of Shc solves the problem.

Indeed Shc has different splice forms and the here described effect is specific for the p46 splice variant. A recent review describes in important role of pShc46 in liver regeneration. We added this to the Discussion section.

• Influence of Acetate. In Figure 7 and in the text you show that there is no bistability with acetate = 3mM. Is there any concentration of acetate >0 where there is still bistability?

At low concentrations of acetate there is still bistability. We added a titration of acetate (Results and Supplemental information).

• Relation of results to diets and eating regimes: If I understand the results correctly, your results confirm the observation that fasting periods and less frequent meals per day is better. By big variations of fatty acid supply and oxidation supply, the risk of being stuck in the low flux state would be minimized, isn’t it?

Yes, this is plausible and it may relate our results to the effect of e.g. intermittent fasting regimes. On the cautious side, however, we have no direct experimental proof for the existence of this bistability in vivo, hence we can only speculate on the effect of diets.

Finally, we checked all the textual comments and suggestions for the figures below and made the suggested corrections and improvements. We particularly thank this reviewer for his/her thorough analysis of the manuscript.

• Line 544: the letter N is here used for the number of metabolites. Later in equation (5) it is used again, but I think here it represents the concentration of NADH/NAD.

This was corrected in the supplemental information.

Equation (4): There is no explanation of “sf_vmckat” and its meaning.

The parameter *sf_vmckatC4_* is a factor modifies *V_ma_*_x_ to make it specific for each chain length (in this case a C4 substrate). We clarified this.

• Line 568: “a few metabolic intermediates were set to be constant” Again, I think it is crucial to explicitly state which metabolites are external and constant to understand the steady state flux properly.

We clarified this in the legend to Figure 1 and in the Methods section.

• Line 617: a>0 is crucial and really trivial to see. E.g. if a=0, it is a quadratic function with maximal two steady state and hence no bistability. Please elaborate shortly, why a>0 is justified.

It is because a is based on multiplications and additions of only positive parameters. This was clarified.

• Figures:

• Figure 1, please add some indication of external (constant or conserved moieties) metabolites. Also elaborate this in the text. Otherwise, the steady state of the metabolic system is not understandable.

Indeed, this information was added to the legend of Figure 1 and in the Methods section.

• Figure 2: there is no Figure 2?

The numbering of the figures was corrected.

- Figure 4: Panels B and C are a bit confusing. It is not really clear what is shown by the lines (mathematically). Please indicate this in the caption or better find overall a better visualization. Are the plots on the right hand side of panel B and C for a fixed palmitoyl-CoA concentration?

Indeed the plots are for a fixed palmitoyl-CoA concentration, which has been added to the legend now. The model was split in a supply and a demand module with C4-ketoacyl-CoA as the linking metabolite. Then the steady-state fluxes of the supply and demand module were computed as a function of a fixed concentration of the linking metabolite. The intersection points indicate the steady states. This was described in the text, but has now also been clarified in the legend.

• Figure 5: For all panels I suggest to use a color gradient for the lines indicating the varied parameter value instead of the high number of colors. In the current version one cannot see intuitively the relation of increasing the parameter value and the corresponding lines. Further, in 5B consider to flip the ratio FAD/FADH2 to FADH2/FAD and use the same ratios as in 5A to have direct comparison of the impact on bistability.

These corrections were made.

• Figure 6: I would suggest to use letters (A,B) again for the subgraphs instead of left/right.

This was corrected as suggested.

• Figure 7: I suggest to rearrange the subpanels to A, B (first row) and C, D second row. I think this is the typical reading pattern across journals. Also check that font sizes of axis titles etc. are uniform, it looks a bit messy at the moment.

This was uniformized as suggested.

•

• References:

• Check overall citation formatting (species name italics, capitalization)

• [20] and [37] are duplicates

• Wording:

- Line 45, “network in -> of? Escherichia coli”
- Line 54, “loop is born” sounds not very scientific
- Line 87, “parameters for -> of? rat-liver”
- Line 159 “parameters [were] unchanged”
- Line 210: “was fixed and varied” sounds like a contradiction (constant over time, but varied initial concentration)
- Line 264: “[link to] control [of] cell metabolism”
- Line 268: “For [a] small value”
- Line 285: “Bistability existed” -> “exists”?
- Line 364-365: “activate the mFAO flux by […?] and”
- Line 431: rephrase “a stable state flux was found either at high or at low flux state” -> e.g. “a stable state flux with either low or high flux”
- Line 434: “de novo” italic?
- Line 442-444: Somehow this sentence is broken at “… coexist which state …”
- Line 498: “… higher FADH2 [concentration] …”
- Line 554: avoid “ … and … and … “

We thank the reviewer for this detailed editing. We made all the changes, but for simplicity we did not highlight them in the text.
